# Distributed Cognition and Process Management Enabling Individualized Translational Research: The NIH Undiagnosed Diseases Program Experience

**DOI:** 10.3389/fmed.2016.00039

**Published:** 2016-10-12

**Authors:** Amanda E. Links, David Draper, Elizabeth Lee, Jessica Guzman, Zaheer Valivullah, Valerie Maduro, Vlad Lebedev, Maxim Didenko, Garrick Tomlin, Michael Brudno, Marta Girdea, Sergiu Dumitriu, Melissa A. Haendel, Christopher J. Mungall, Damian Smedley, Harry Hochheiser, Andrew M. Arnold, Bert Coessens, Steven Verhoeven, William Bone, David Adams, Cornelius F. Boerkoel, William A. Gahl, Murat Sincan

**Affiliations:** ^1^NIH Undiagnosed Diseases Program, Common Fund, Office of the Director, National Institutes of Health (NIH), Bethesda, MD, USA; ^2^National Human Genome Research Institute, National Institutes of Health (NIH), Bethesda, MD, USA; ^3^RURO, Inc., Frederick, MD, USA; ^4^Centre for Computational Medicine Hospital for Sick Children, Toronto, ON, Canada; ^5^Department of Computer Science, University of Toronto, Toronto, ON, Canada; ^6^Department of Medical Informatics and Clinical Epidemiology, Oregon Health & Science University, Portland, OR, USA; ^7^Lawrence Berkeley National Laboratory, Division of Environmental Genomics and Systems Biology, Berkeley, CA, USA; ^8^Department of Clinical Pharmacology, Queen Mary University London, London, UK; ^9^Department of Biomedical Informatics and Intelligent Systems, University of Pittsburgh, Pittsburgh, PA, USA; ^10^Agilent Technologies, Inc., Santa Clara, CA, USA

**Keywords:** translational research, information system, ontology-based phenotyping, process management system, precision medicine

## Abstract

The National Institutes of Health Undiagnosed Diseases Program (NIH UDP) applies translational research systematically to diagnose patients with undiagnosed diseases. The challenge is to implement an information system enabling scalable translational research. The authors hypothesized that similar complex problems are resolvable through process management and the distributed cognition of communities. The team, therefore, built the NIH UDP integrated collaboration system (UDPICS) to form virtual collaborative multidisciplinary research networks or communities. UDPICS supports these communities through integrated process management, ontology-based phenotyping, biospecimen management, cloud-based genomic analysis, and an electronic laboratory notebook. UDPICS provided a mechanism for efficient, transparent, and scalable translational research and thereby addressed many of the complex and diverse research and logistical problems of the NIH UDP. Full definition of the strengths and deficiencies of UDPICS will require formal qualitative and quantitative usability and process improvement measurement.

## Introduction

Established in 2008, the National Institutes of Health (NIH) undiagnosed diseases program (UDP) provides answers to patients with conditions that have eluded diagnosis and advances medical knowledge about rare and common diseases (1). The NIH UDP implements translational research by integrating extensive phenotyping with genetic studies in a systematic fashion.

Translational research is a discipline within biomedical and public health research that aims to improve the health of individuals and the community by “translating” findings into diagnostic tools, therapies, policies, and education. The NIH has made translational research a priority, forming centers of translational research at its institutes and launching the Clinical and translational science award program in 2006. Translational research can be divided into five components^1^ (2). The first component, bedside to bench (T0), is the transfer of the medical physiological understanding of human diseases to the laboratory in a manner enabling application of scientific reductionism. The second component, bench to bedside (T1), is the transfer of new understandings of disease mechanisms gained in the laboratory into the development of new methods for diagnosis, therapy, and prevention of human disease. The third component (T2) builds on the clinical efficacy work conducted in T1 processes and includes phase 3 clinical trials as well as addressing questions about who benefits. The fourth component (T3) is the translation of results from clinical studies into clinical practice and health decision-making. The fifth component (T4) is the extension of T3 translation to education of practitioners, adoption promotion, policy re-evaluation, and outcomes assessment. In this context, the NIH UDP focuses on the first two components of translational research.

The NIH UDP admits approximately 100–120 families with different diseases annually and must systematically and scalably assess the evolutionary, developmental, and biochemical homeostases of each patient (3). Besides the sheer number of diseases, the challenges encountered by the NIH UDP include (1) a focus on individual patients, not cohorts; (2) data collection and aggregation; (3) uniform archiving of biospecimens with metadata; (4) process management; (5) secure data access; (6) generation and presentation of diagnostic hypotheses; (7) a forum for and recording of discussions, conclusions, and decisions; (8) formation of virtual villages of niche experts to focus on each patient; and (9) deposition of appropriate data into public databases.

These NIH UDP throughput and infrastructure requirements define a need for an intelligent scalable process management system. We hypothesized that integration of process management with distributed cognition (4) addresses the NIH UDP information system requirements. According to the theory of distributed cognition, a cognitive process is delimited by the functional relationships among the elements that participate in it, rather than by the spatial colocation of the elements, and is often characterized by (1) cognitive processes distributed across the members of a social group, (2) cognitive processes coordinating internal and external (material or environmental) structure, and (3) processes distributed through time (5). In this context, we reviewed some open source and commercial software packages (Table 1). Our review of open source and commercial software packages, including electronic data capture (EDC) systems, clinical trial management systems (CTMS), collaboration systems, and laboratory information management systems (LIMS), showed that none had all the required features or had the upper level production management needed for translational research as envisioned for the NIH UDP (6, 7). Similarly, evaluation of specialized systems such as EHR4CR^2^ and Taverna^3^ highlighted the strengths of each, but also positioned them hierarchically below the production system needed by the NIH UDP, i.e., the system required by the NIH UDP would link out to Taverna for required functions. Consequently, we decided address of the NIH UDP requirements and scalable T0 and T1 translational research required construction of a new system.

**Table 1:** **Comparison of the NIH UDP requirements to representative existing software**.

NIH UDP required features	UDPICS^a^	Representative alternative software							
		Genologics clarity LIMS^b^	Alfresco one	Atlassian’s confluence	Progeny suite	Exemplar knowledge management^b^	RedCap	OpenClinica	TransMed suite
Clinical data capture (i.e., phenotypes, demographics, visit information, etc.)	+	−	−	−	+	+	+	+	+
Pedigree tools	+	−	−	−	+	−	−	−	−
Research data capture	+	−	−	−	+	+	+	+	+
Individual patient-focused platform	+	NR	−	−	+	NR	−	+	−
Structured and unstructured data capabilities	+	+	−	+	+	+	+	+	+
File upload and management capabilities	+	NR	+	+	+	+	+	+	+
Internal biorepository (includes freezer designations, quality control measures, and metadata capture)	+	+	−	−	+	+	−	−	−
Process management (displays tasks completed, delinquent, and remaining)	+	+	+	+	+	+	−	−	−
Permission management	+	+	+	−	+	NR	+	+	+
Communication tools	+	−	+	+	−	+	−	−	−
Presentation of diagnostic hypotheses to researchers and clinical staff (differential diagnosis)	+	−	−	−	−	−	−	−	−
Easy deposition of quality data into public repositories	+	−	−	−	−	−	−	−	−
APIs for communication with external platforms	+	+	+	+	−	+	+	+	−
Accessible to global collaborators/team members	+	+	+	+	+	+	+	+	+
Minimal intrinsic data analysis tools that require maintenance	+	−	−	−	+	+	−	−	+

*^a^Undiagnosed diseases program integrated collaboration system*.

*^b^No demo available*.

*NR, not reported*.

## Defining the NIH UDP Translational Research Process Series

**Figure 1:**
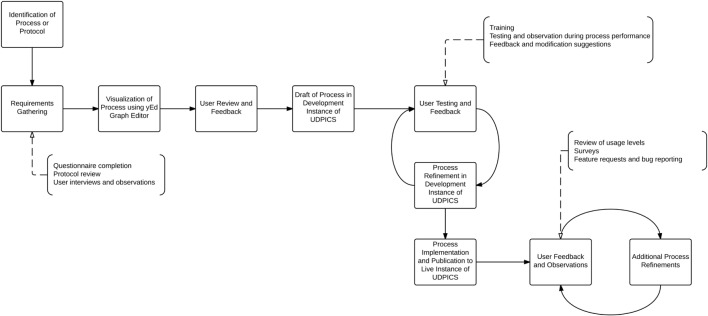
**Graphical representation of the process by which workflows were designed, implemented, and refined within UDPICS**.

The NIH UDP translational research process management is based on the hypothesis that translational research reduces to a series of tasks, outcomes, and decisions that can be captured in a searchable database. The secondary hypothesis is that standardized workflows are sufficiently flexible for testing scientific hypotheses and implementing research and clinical decisions. We designed, implemented, and refined the system as shown in Figure 1.

The processes and activities in the NIH UDP were categorized into three broad groups: clinical evaluation, agnostic screening for hypothesis generation, and hypothesis testing for elucidation of causation and biological mechanisms. We mapped the processes, tasks, and data elements of the NIH UDP by interviewing personnel (21 clinical staff members – 10 physicians, 6 nurse practitioners, and 5 administrative support staff – and 18 research staff members – laboratory manager, 6 laboratory researchers, 3 laboratory technicians, and 8 bioinformatics support staff) using a standardized questionnaire available as Data Sheet 1 in Supplementary Material. We supplemented interview information with analysis of protocols and operating procedures and observation of individuals executing their tasks.

We used the yEd Graph Editor (yEd Graph Editor, Version 3.10.1. yWorks, Germany) to define >80 workflows [see Data Sheet 2 in Supplementary Material, which lists all the workflows and their properties within UDP integrated collaboration system (UDPICS) in JavaScript object notation (JSON) format] and >2000 discrete data elements (see Data Sheet 3 in Supplementary Material, which lists all the user-defined fields or data elements and their properties in JSON format) in >100 data tables; these spanned patient recruitment through patient data disposition. This process also delineated three permission levels characterizing roles, interactions, and data access (see Table 1 in Supplementary Material, which defines the three permission levels for both workflows and data elements that characterize how users interact with the processes and data within UDPICS).

We built the UDPICS on the Limfinity^®^ framework (RURO, Inc., Frederick, MD, USA), a Ruby on Rails web application running on a Linux operating system. The Limfinity^®^ framework supports a metadata-based data model and process design as well as common requirements like account and login management, system security, and database searches as well as the use of multiple backend database platforms, including MySQL (Structured Query Language), Oracle, Microsoft SQL, and PostgreSQL. HTML5 (Hyper Text Markup Language), CSS (Cascading Style Sheets), and JavaScript are used to create dynamic and responsive user interfaces.

**Figure 2:**
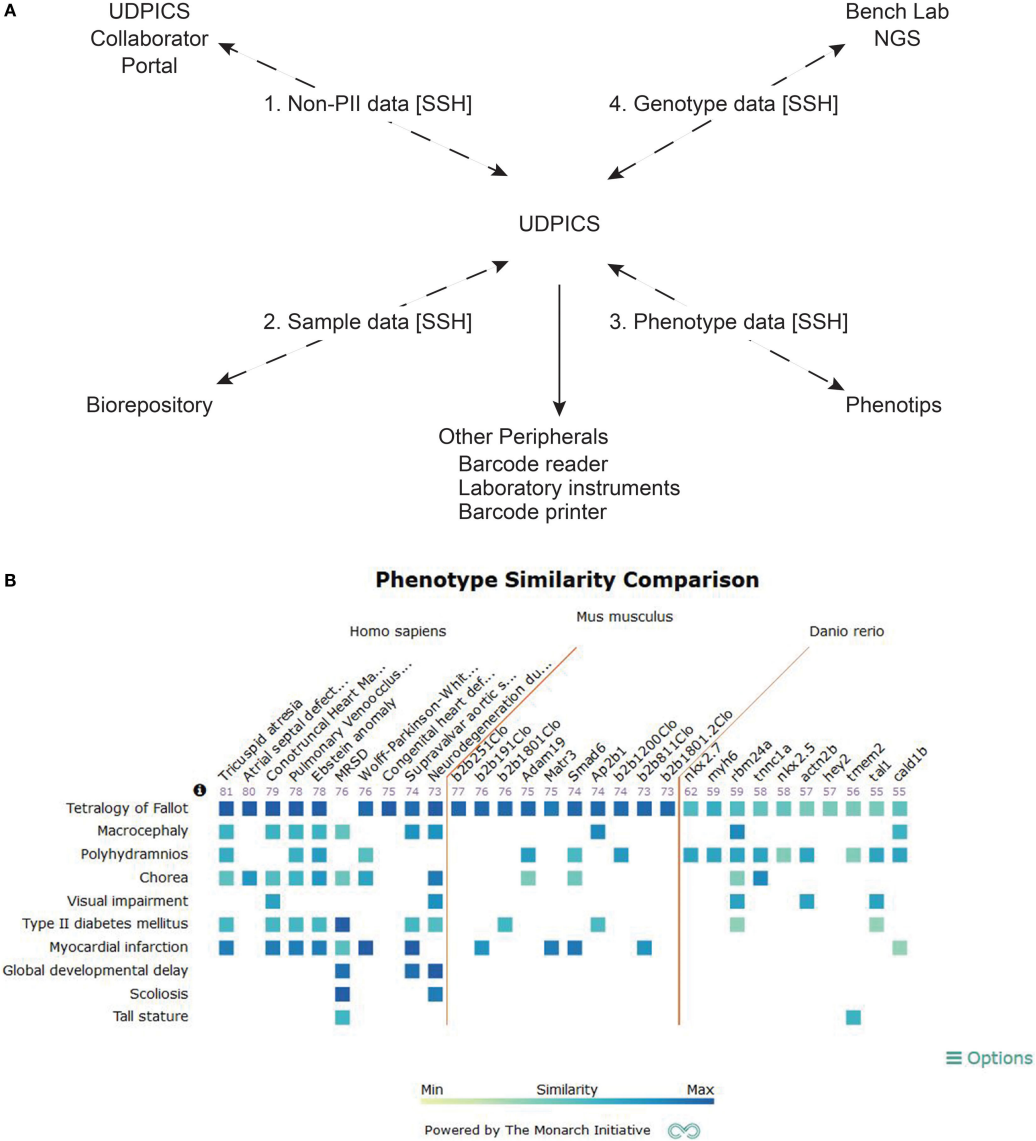
**Description of UDPICS architecture and screen shot of PhenoGrid user interface**. **(A)** Graphical display of all the components of UDPICS and the data that are distributed among those components. **(B)** Screenshot of the system showing PhenoGrid comparison of a patient’s phenotype data against published human diseases and established mutant *Mus musculus* and *Danio rerio* lines.

We implemented UDPICS in five stages over 3 years (Figure 2A). First, we defined patient recruitment and assessment workflows and transferred historical data. Second, we defined workflows for patient biospecimen processing, archiving, and metadata association and imported additional historical data. Third, we defined workflows for patient-focused research, including data transfer to and from collaborating external laboratories. Fourth, we implemented workflows for generation and characterization of patient-focused animal models. Last, we automated data deposition to repositories, such as the Database of Genotypes and Phenotypes (dbGaP). Upon implementation of each stage, users received individual training and wiki-based documentation. User requests for new features and bug reports were managed *via* a Redmine issue tracking system.^4^

## Scaling the NIH UDP Translational Research Process Series

After establishing workflows spanning NIH UDP research from recruitment through patient data deposition, our challenge was to facilitate the concurrent study of many diseases. We hypothesized that the distributed cognition of niche experts efficiently solves patient problems. Distributed cognition acknowledges that cognitive processes are distributed across environments and social groups, rather than confined to a place, individual, or group (4). Consequently, cognition is socially and culturally dependent and is embodied in the social group’s response to and the use of environment (4). A historical example of such a social group is a village, and with the advent of social media, village members can be drawn from the global population and assembled virtually. Both, physical and virtual villages require an organized structure, participation to maximize social capital, contribution of particular skills by each member to the community, communication within communal space for exchange of knowledge, and acceptance of the societal hierarchy. Incorporating these principles, UDPICS enabled methodical leveraging of global expertise to scale concurrent study of diseases (Figure 2A).

### Information Sharing and Common Language

#### Patient Medical History and Phenotype

Using the integrated web-based phenotyping application, PhenoTips (8), clinical phenotypes are captured in a standardized nomenclature, human phenotype ontology (HPO) (9). In contrast to the systematized nomenclature of medicine (SNOMED) or international classification of diseases (ICD), HPO is a formal ontology amenable to application of computerized algorithms for delineation of differential diagnoses and variant rankings (10, 11). UDPICS produces the HPO-dependent differential diagnosis with the OWLSim algorithm of Phenogrid (12) (Figure 2B) and the algorithm within PhenoTips (8).

#### Familial Structure

To generate pedigrees for segregation of disease within families, we incorporated a pedigree drawing tool based on Madeline (13). Derived PED files are used for downstream genomic studies.

#### Patient Sample Biorepository

To provide characterized biospecimens to the NIH UDP staff and collaborators, each specimen tracked within UDPICS is annotated with patient metrics, the date and time of collection, current medications, processing duration, and quality measures. UDPICS also tracks specimen storage location, use, and distribution.

#### Research Data

Undiagnosed diseases program integrated collaboration system contains sharable electronic laboratory notebooks (ELNs) for dissemination of research information. Each notebook is linked to a task, patient, or cohort. Each ELN is divided among the independent research groups working on a problem, and subdivided according to researchers in the group. Each note has three levels of electronic sign-off and locking: the performing researcher generates and signs the experimental note; the supervising principal investigator reviews and signs; and, if appropriate, the NIH UDP research manager reviews and signs.

### Communication Tools

#### Dashboards

**Figure 3:**
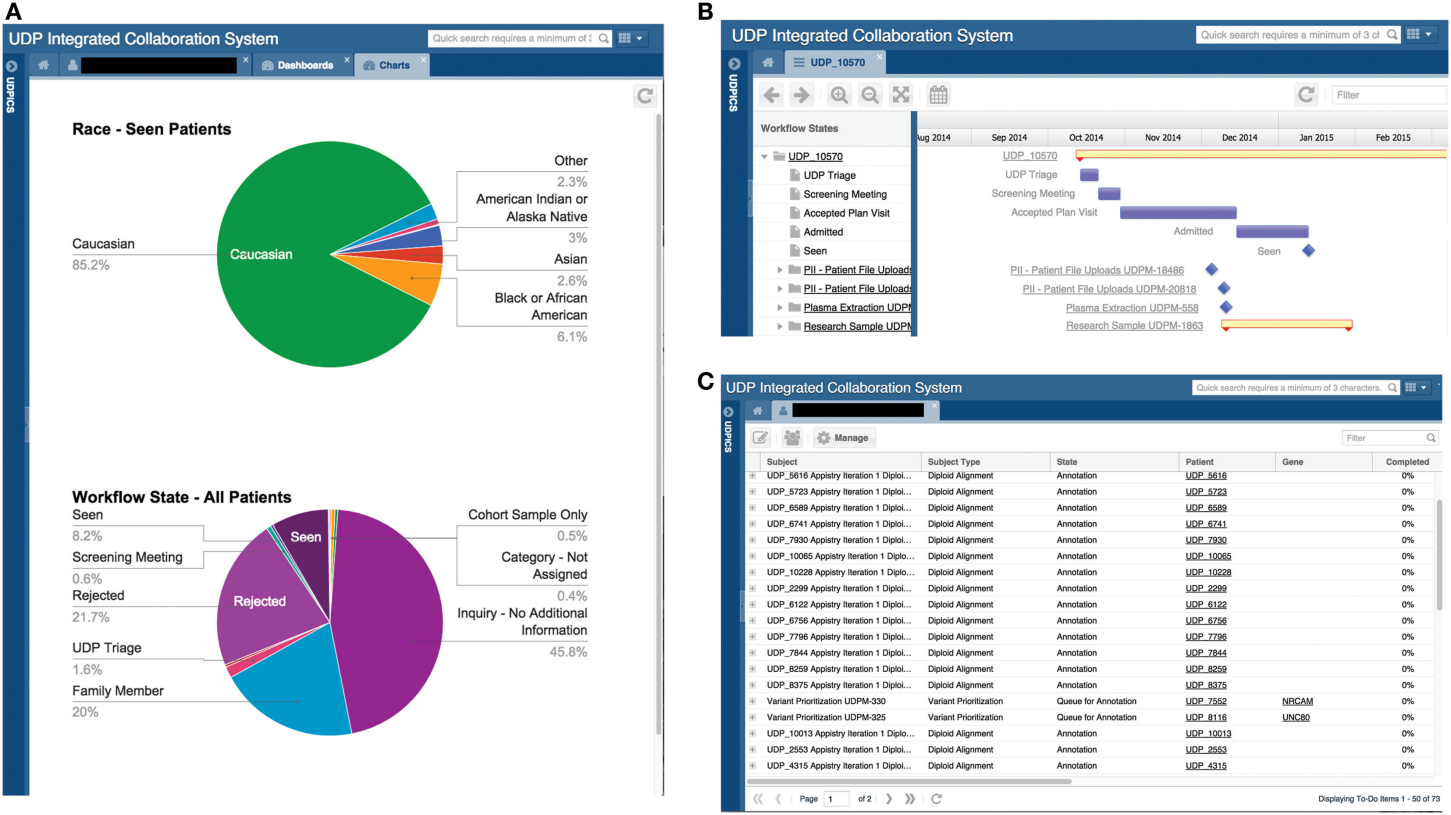
**Screen shots of user interfaces for possible dashboards, the Gantt chart, and the to-do list**. **(A)** Screenshot of the system showing two customized dashboards that display specific data on all or a subset of patients in a graphical format. Please note that the figures in this screenshot are examples, not accurate depictions of NIH UDP patient demographics. **(B)** Screenshot of the system showing a Gantt chart displaying tasks and processes associated with a patient and the progress of each. **(C)** Screenshot of a user’s to-do list showing the tasks assigned to the user, including the allowed duration and number of days overdue.

Undiagnosed diseases program integrated collaboration system contains customizable dashboards for displaying information. Examples include the number of patients in each workflow state, age distribution of patients, and current status of patient genomic studies (Figure 3A).

#### Gantt Chart

The Gantt chart provides a status overview on each workflow for a patient or project cohort. It displays progress and duration and serves as an active link to the details of each task (Figure 3B).

#### Lists

Undiagnosed diseases program integrated collaboration system contains two central lists: a to-do list and an activity list. The to-do list displays a user’s current tasks and the time for completion (Figure 3C). The activity list links to actions that the user chose to be notified of.

#### Comments

**Figure 4:**
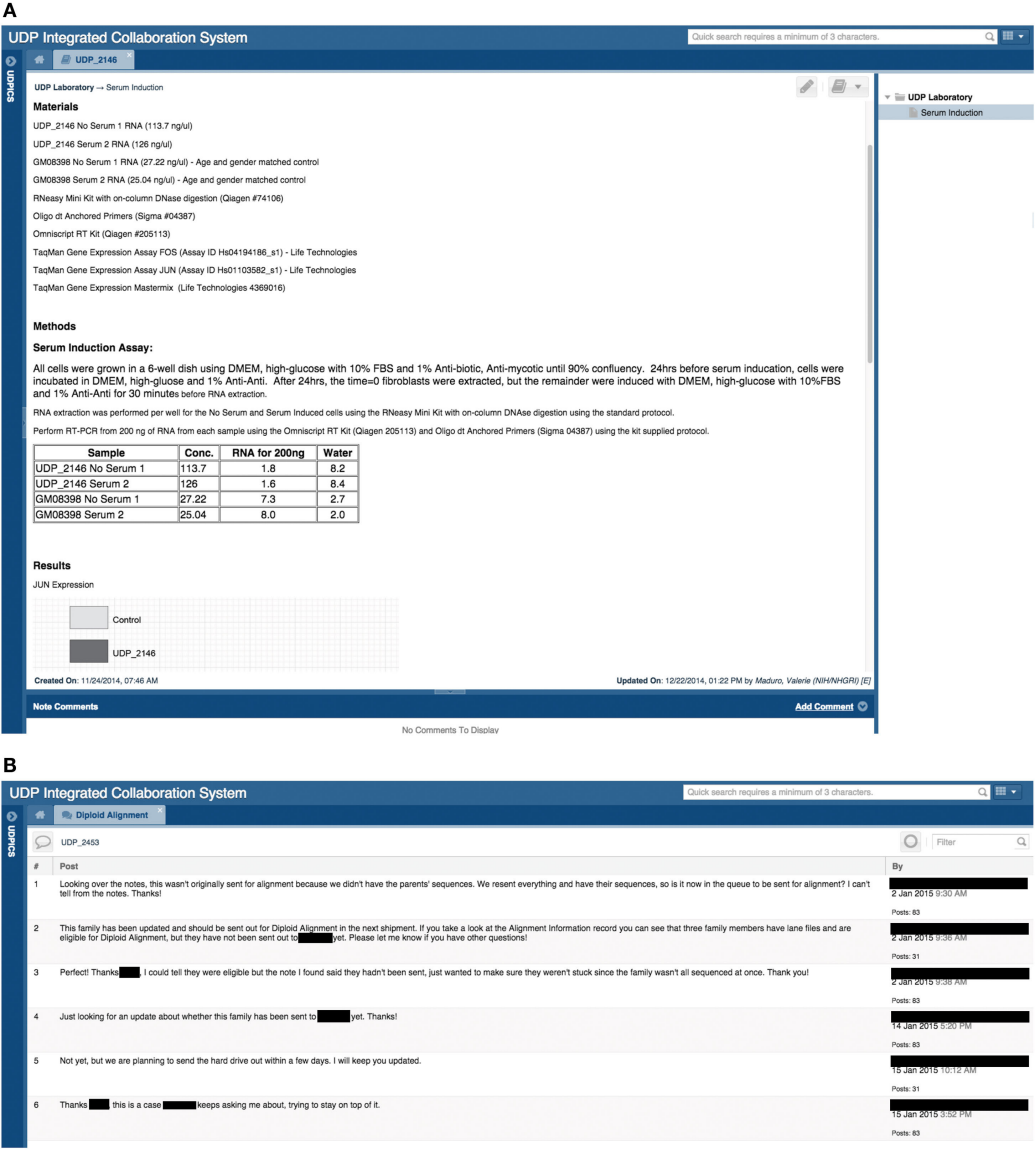
**Screen shots of user interfaces for a page in the electronic laboratory notebook (ELN) and of the Chat tool**. **(A)** Screenshot of a note in a patient’s ELN that outlines a hypothetical experiment performed by a researcher. **(B)** Screenshot of a chat regarding a specific patient and the communication between the clinical group and the bioinformatics group.

To facilitate discussion and participation, each UDPICS ELN note has a comment bar for supervisors and collaborators to provide feedback (Figure 4A). Viewing and commenting on ELN notes is controlled by permissions.

#### Chat

To capture discussion and decisions, UDPICS includes a chat feature for communication among internal and external users. Each chat, which can include multiple users, is embedded within a specific patient record or cohort project and can be further linked to a task or note (Figure 4B).

#### Email Notifications

Undiagnosed diseases program integrated collaboration system sends email alerts for user-defined issues of importance. These issues currently include quality control, task completion, and upload of specific reports.

## Udpics Improved Operational Efficiency

As a quantitative measure of system usefulness, we assessed time to accomplish three tasks: bulk shipment of samples from the biorepository, submission of data to databases, and exchange of data and reagents with collaborators. For bulk shipment of samples from 100 patients, the time to organize a report on plasma, urine, and cerebrospinal fluid specimens and associated metadata was approximately three workdays prior to UDPICS implementation; it was only 2 h after. For data submission to dbGaP, data assembly required extraction from multiple databases and paper files over three to five workdays before implementation of UDPICS and approximately one workday after. Assembly and processing of a material or intellectual transfer agreement application took three to five workdays and, upon approval, one to two workdays for the transfer of the material or data; after UDPICS implementation, data assembly and transfer occurred instantaneously with the only delay being the human approval process. Lastly, sharing of results among NIH personnel and collaborators previously required emails or phone calls over several days, whereas after implementation, UDPICS provided instantaneous knowledge of experiment status.

Anecdotal feedback from users has been positive. They particularly enjoy the improved transparency, accountability, and coherence of information. Clinical staff can now avoid mixed messages to patients, and collaborators have access to a complete data set.

## Discussion

The NIH UDP developed UDPICS, a web-based information and process management system, to support scalable translational research and, thereby, bridge the gap between the patient bedside and the research bench. Leveraging the principles of distributed cognition, this software solution addressed the needs of the NIH UDP through its focus on individual patients, data aggregation capabilities, archiving of quality biospecimens with associated metadata, efficient linking to other systems for data collection and analysis, robust process management, granular data permissions, transparent presentation of diagnostic hypotheses and decisions to researchers and clinical staff, support for virtual villages of geographically dispersed niche experts, and efficient transfer of data into public databases. Consequently, through the UDPICS ecosystem, the NIH UDP improved operating efficiency, standardized processes and data, and facilitated communication and information sharing among participants.

Although existing software tools had some overlapping functionality, the UDP’s needs exceeded the capabilities of existing platforms. Specifically, in addition to collecting research data in electronic format as for a traditional clinical trial, the NIH UDP required a comprehensive collaboration system that supported phenotyping, biospecimen management, native workflow integration, and model organism management. UDPICS, thus, aggregated many functions to achieve essential novel capabilities and validated the hypothesis that distributed cognition enables scalable translational research.

The theory of distributed cognition acknowledges that cognitive processes are distributed across various environments and social groups, rather than confined to one place, individual, or group (5). Consequently, cognition is socially and culturally dependent and is embodied in the social group’s response to and the use of their environment (4). Historically, humans have solved complex problems through the distribution of cognitive processes among a social group with a variety of skills, e.g., a village. With the advent of social media, village members can be drawn from the global population and assemble virtually. Distributed cognition within a virtual village, therefore, differs little from that of a physical village excepting the absence of physical proximity of the members. Both types of villages require an organized structure, participation to maximize social capital, contribution of particular skills by each member to the community, communication within communal space for exchange of knowledge, and acceptance of the societal hierarchy. By incorporating these principles, UDPICS enabled methodical leveraging of global expertise through formation of virtual villages and, thereby, provided the NIH UDP a platform for scalable translational research.

The implementation challenges faced by the NIH UDP for UDPICS were common to those observed for other enterprise software. Although there were technical challenges of transfer of data from legacy systems, security, and systems integration, the major challenges were organizational and human. UDPICS required a change in organizational culture for all participants within the NIH UDP, and, thus, its implementation required a holistic perspective of the entire process. To achieve this, the implementation required the commitment of senior management; the support of, ongoing communication with, and training of users; and alteration of workflows and interfaces to meet user needs. When users realized that the system contained all the data and processes required for performance of their tasks, they endorsed the system and the NIH UDP realized increased operating efficiency.

Undiagnosed diseases program integrated collaboration system has benefited the NIH UDP and its collaborators by providing a common information source, improving communication; enhancing decision-making and planning capabilities, integrating workflows, maintaining best practices, providing system-wide security, enabling real-time data access, defining regular, consistent data, and increasing data integrity, validity, and reliability. Having achieved the trust of its users, UDPICS became the nexus for the NIH UDP and its collaborators. We propose, therefore, that implementation of UDPICS or an equivalent system incorporating distributed cognition and process management will similarly benefit other programs conducting T0 and T1 translational research.

Undiagnosed diseases program integrated collaboration system supports static database requirements and fully integrates the complex dynamics of the research process. It also provides tools to automate the flow of information and ensure data quality, e.g., user task lists, email notifications, and full scriptability from the administrative interface. Also, the flexibility of the system design and the easy-to-use graphical interface enable the NIH UDP and its collaborators to access the information needed to complete their research projects and provide feedback to each other easily and efficiently.

This initial version of UDPICS requires further development and formal assessment. First, for security reasons UDPICS was developed as independent software; however, it would ideally connect to other databases such as the NIH Clinical Center electronic medical record rather than requiring manual re-entry of EMR data. Second, fully informed translational research requires participation of the patient and/or the caretakers; therefore, future development will include a portal through which patients can (1) provide self-phenotyping, e-consenting, and form completion; (2) access appointment calendars and research data; and (3) participate in the research process.

Full assessment of UDPICS’s benefit to the NIH UDP and other translational research programs requires formal qualitative and quantitative measures. Having such measures of its benefits will allow other institutions to judge the potential value of this software infrastructure for their personalized medicine programs.

In summary, the systematic implementation and incorporation of the principles of process management and distributed cognition within UDPICS has enabled scalable translational research through improved efficiency, quality control, and leveraging of global expertise within the NIH UDP. UDPICS has also addressed each of the challenges that the NIH UDP identified for such software. Much work remains, however, to enable patient participation and improve interaction with other biomedical research software systems.

## Author Contributions

DA, CB, WB, WG, and MS conceived the methodology. AL, DD, EL, JG, ZV, VM, and MS developed the system for use at the NIH UDP. VL, MD, and GT provided the Limfinity^®^ platform and support for development needs. MB, MG, and SD provided support for PhenoTips. AA, BC, SV, and WB provided support for Cartagenia Bench Lab NGS. HH and MH performed usability evaluation for UDPICS and PhenoTips. MH, CM, DS, and HH provided cross species phenotype comparison, ontological algorithms, and visualization from the Monarch Initiative. AL, CB, and MS wrote the manuscript. All authors read and approved the final manuscript.

## Conflict of Interest Statement

VL and MD are stakeholders in RURO, Inc.; the UDPICS infrastructure or framework can be viewed as Translational Science Informatics at https://ruro.com/solutions/featured-translational-science. GT and AL are employed by RURO, Inc. AA, BC, SV, and WB are employed by Agilent Technologies. The remaining authors declare no conflicts of interest. This article does not involve a research protocol requiring approval by the relevant institutional review board or ethics committee.

## Footnotes

^1^https://casemed.case.edu/ctsc/cores/documents/CTSC_Projects_and_Translational_Research_2010-0111%20Final.pdf

^2^http://www.ehr4cr.eu

^3^http://www.taverna.org.uk

^4^http://www.redmine.org, Redmine. 1.3.2.stable
