# Supplementary material for: Distributed Cognition and Process Management Enabling Individualized Translational Research: The NIH Undiagnosed Diseases Program Experience
Source: Front Med (Lausanne). 2016 Oct 12;3:39. doi: 10.3389/fmed.2016.00039 (PMC5060938; doi:10.3389/fmed.2016.00039)
Supplement: Supplementary file 2 [file data_sheet_1.pdf]

1. What is the beginning step?
2. Are there any pre-requisites to starting this step?
3. Is this step being performed on an existing patient/vial/record or is it a stand-alone task?
4. What information (and types of information) will be captured in this step?
5. How will this information be organized?
    a. All data collected in one sub-task?
    b. Data collected in stages/sub-tasks within the first step?
6. How long should this step take?
7. Who is responsible for this step?
    a. Owner – single individual ultimately responsible for the task being completed and data collected
    b. Manager(s) – one or more individuals who oversee the completion of the task
    c. Performer(s) – one or more individuals who perform the task to completion
        i. Is there a pre-defined structure to how performers are assigned?
        ii. Randomized assignment
        iii. Default performer(s)
8. Are there conditions for completion?
9. Does anyone need to be alerted or notified during this step?
    a. Task start
    b. Poor data quality
    c. Task completion
    d. Task overdue
    e. Collection of specific data
10. What is the next step?
    a. If more than one next step, what are the conditions for advancement to each of those steps?
    b. Does anyone need to be notified upon completion or poor data quality?
11. Are there specific conditions that need to be acted upon when completed?
    a. New record created
    b. Existing record advanced in a workflow
    c. Record stored as a biospecimen
