# Supplementary material for: Distributed Cognition and Process Management Enabling Individualized Translational Research: The NIH Undiagnosed Diseases Program Experience
Source: Front Med (Lausanne). 2016 Oct 12;3:39. doi: 10.3389/fmed.2016.00039 (PMC5060938; doi:10.3389/fmed.2016.00039)
Supplement: Supplementary file 3 [file data_sheet_2.doc]

[{

"name": "Sensitive Information Workflow",

"uuid": "UDPM-224",

"subject_type": "UDPM-276",

"state_defs": [

{

"name": "Sensitive Information",

"uuid": "UDPM-1111",

"owner": "UDPM-67",

"managers": "UDPM-27",

"performers": "UDPM-49",

"tools": [

{

"name": "Edit Information",

"uuid": "UDPM-1527",

"input_type": "UDPM-276",

"output_type": "UDPM-276",

"flags": 1,

"options": {

"udfs": [

"UDPM-6",

"UDPM-2912",

"UDPM-1301"

]

}

}

],

"show_options": {

"top": 180,

"left": 400

},

"end_task": true

}

],

"entry_point": "UDPM-1111"

},{

"name": "Cohort Files",

"uuid": "UDPM-225",

"subject_type": "UDPM-277",

"state_defs": [

{

"name": "Upload Cohort Files",

"uuid": "UDPM-1112",

"owner": "UDPM-67",

"managers": "UDPM-21",

"performers": "UDPM-21",

"show_options": {

"top": 180,

"left": 400

},

"end_task": true

}

],

"entry_point": "UDPM-1112",

"show_udfs": "UDPM-2812,UDPM-2032,UDPM-1649,UDPM-2374,UDPM-2894"

},{

"name": "Overnight Collaboration Shipment Workflow",

"uuid": "UDPM-222",

"subject_type": "UDPM-273",

"state_defs": [

{

"name": "Request Ordered",

"uuid": "UDPM-1097",

"owner": "UDPM-67",

"managers": "UDPM-49",

"performers": "UDPM-46",

"tools": [

{

"name": "Shipment Information",

"uuid": "UDPM-1498",

"input_type": "UDPM-273",

"output_type": "UDPM-273",

"flags": 1,

"options": {

"udfs": [

"UDPM-1389",

"UDPM-309"

]

}

}

],

"next_states": [

{

"state_name": "Sample Shipped",

"state_uuid": "UDPM-1098",

"btn_text": "Samples Shipped",

"btn_css": "rgb(13, 73, 123)",

"btn_scale": "large"

}

],

"show_options": {

"top": 126,

"left": 106

}

},

{

"name": "Sample Shipped",

"uuid": "UDPM-1098",

"owner": "UDPM-67",

"managers": "UDPM-49",

"performers": "UDPM-49",

"tools": [

{

"name": "Upload PII File",

"uuid": "UDPM-1534",

"input_type": "UDPM-273",

"output_type": "UDPM-172",

"flags": 0,

"options": {

"obj_type": "SubjectType"

}

},

{

"name": "Add Collaboration Report",

"uuid": "UDPM-1533",

"input_type": "UDPM-273",

"output_type": "UDPM-178",

"flags": 0,

"options": {

"obj_type": "SubjectType"

}

}

],

"show_options": {

"top": 187,

"left": 455

},

"end_task": true

}

],

"entry_point": "UDPM-1097",

"show_udfs": "UDPM-2489,UDPM-1472"

},{

"name": "Send Out Testing",

"uuid": "UDPM-226",

"subject_type": "UDPM-278",

"state_defs": [

{

"name": "Cancel Requisition",

"uuid": "UDPM-1119",

"owner": "UDPM-67",

"managers": "UDPM-40",

"performers": "UDPM-40",

"show_options": {

"top": 78,

"left": 774

},

"end_task": true

},

{

"name": "POTS",

"uuid": "UDPM-1114",

"owner": "UDPM-67",

"managers": "UDPM-40",

"performers": "UDPM-40",

"tools": [

{

"name": "POTS Information",

"uuid": "UDPM-1536",

"input_type": "UDPM-278",

"output_type": "UDPM-278",

"flags": 1,

"options": {

"udfs": [

"UDPM-1752",

"UDPM-1753"

]

}

}

],

"next_states": [

{

"state_name": "Ready for Shipment",

"state_uuid": "UDPM-1115",

"btn_text": "Entry Complete",

"btn_css": "rgb(13, 73, 123)",

"btn_scale": "large"

},

{

"state_name": "Send Out Testing",

"state_uuid": "UDPM-1113",

"btn_text": "Return to [redacted] for handling",

"btn_css": "rgb(13, 73, 123)",

"btn_scale": "large"

}

],

"em_performer_new_job": true,

"show_options": {

"top": 28,

"left": 467

}

},

{

"name": "Prepare Samples",

"uuid": "UDPM-1117",

"owner": "UDPM-67",

"managers": "UDPM-40",

"performers": "UDPM-40",

"next_states": [

{

"state_name": "Ready for Shipment",

"state_uuid": "UDPM-1115",

"btn_text": "Ready for Shipment",

"btn_css": "rgb(13, 73, 123)",

"btn_scale": "large"

},

{

"state_name": "Return Requisition",

"state_uuid": "UDPM-1118",

"btn_text": "Return Requisition to Requestor",

"btn_css": "rgb(13, 73, 123)",

"btn_scale": "large"

}

],

"show_options": {

"top": 311,

"left": 421

}

},

{

"name": "Ready for Shipment",

"uuid": "UDPM-1115",

"owner": "UDPM-67",

"managers": "UDPM-40",

"performers": "UDPM-40",

"tools": [

{

"name": "Tool Name",

"uuid": "UDPM-1537",

"input_type": "UDPM-278",

"output_type": "UDPM-278",

"description": "Tool Description",

"flags": 1,

"options": {

"udfs": [

"UDPM-1672",

"UDPM-309",

"UDPM-1515",

"UDPM-1797"

]

}

}

],

"next_states": [

{

"state_name": "Results Received",

"state_uuid": "UDPM-1116",

"btn_text": "Awaiting Results",

"btn_css": "rgb(13, 73, 123)",

"btn_scale": "large"

},

{

"state_name": "Prepare Samples",

"state_uuid": "UDPM-1117",

"btn_text": "To Lab for Sample Preparation",

"btn_css": "rgb(13, 73, 123)",

"btn_scale": "large"

}

],

"show_options": {

"top": 348,

"left": 49

}

},

{

"name": "Results Received",

"uuid": "UDPM-1116",

"owner": "UDPM-67",

"managers": "UDPM-40",

"performers": "UDPM-40",

"tools": [

{

"name": "Results Received",

"uuid": "UDPM-1538",

"input_type": "UDPM-278",

"output_type": "UDPM-278",

"flags": 1,

"options": {

"udfs": [

"UDPM-1754"

]

}

}

],

"show_options": {

"top": 534,

"left": 356

},

"end_task": true

},

{

"name": "Return Requisition",

"uuid": "UDPM-1118",

"owner": "UDPM-67",

"managers": "UDPM-40",

"performers": "UDPM-40",

"next_states": [

{

"state_name": "Cancel Requisition",

"state_uuid": "UDPM-1119",

"btn_text": "Cancel Requisition",

"btn_css": "rgb(13, 73, 123)",

"btn_scale": "large"

},

{

"state_name": "Prepare Samples",

"state_uuid": "UDPM-1117",

"btn_text": "Return to Sample Preparation",

"btn_css": "rgb(13, 73, 123)",

"btn_scale": "large"

}

],

"show_options": {

"top": 288,

"left": 754

}

},

{

"name": "Send Out Testing",

"uuid": "UDPM-1113",

"owner": "UDPM-67",

"managers": "UDPM-40",

"performers": "UDPM-40",

"tools": [

{

"name": "Send Out Information",

"uuid": "UDPM-1535",

"input_type": "UDPM-278",

"output_type": "UDPM-278",

"flags": 1,

"options": {

"udfs": [

"UDPM-415",

"UDPM-1672",

"UDPM-309"

]

}

}

],

"next_states": [

{

"state_name": "POTS",

"state_uuid": "UDPM-1114",

"btn_text": "To [redacted] for POTS processing",

"btn_css": "rgb(13, 73, 123)",

"btn_scale": "large"

},

{

"state_name": "Ready for Shipment",

"state_uuid": "UDPM-1115",

"btn_text": "POTS not Required",

"btn_css": "rgb(13, 73, 123)",

"btn_scale": "large"

}

],

"em_performer_new_job": true,

"show_options": {

"top": 2,

"left": 24

}

}

],

"entry_point": "UDPM-1113",

"ask_performer": true,

"show_udfs": "UDPM-6,UDPM-1696"

},{

"name": "Lymphoblast Tracking Workflow",

"uuid": "UDPM-221",

"subject_type": "UDPM-272",

"state_defs": [

{

"name": "Cell line received by UDP lab",

"uuid": "UDPM-1088",

"owner": "UDPM-67",

"managers": "UDPM-49",

"performers": "UDPM-22",

"tools": [

{

"name": "Send for Collaboration",

"uuid": "UDPM-1460",

"input_type": "UDPM-272",

"output_type": "UDPM-147",

"flags": 0,

"before_code": "collaborator = find_subject( :subject_type=>'Collaborators', :name=>'[redacted]')\n\nparams[:defaults] = {\n 'Collaborator Names'=> collaborator,\n 'Sample Types' => \"Lymphoblasts\"\n}\n\n ",

"options": {

"obj_type": "SubjectType"

}

}

],

"show_options": {

"top": 435,

"left": 565

},

"end_task": true

},

{

"name": "Cells Requested from Institution",

"uuid": "UDPM-1090",

"owner": "UDPM-67",

"managers": "UDPM-49",

"performers": "UDPM-22",

"next_states": [

{

"state_name": "Cell line received by UDP lab",

"state_uuid": "UDPM-1088",

"btn_text": "Cells received by UDP",

"btn_css": "rgb(13, 73, 123)",

"after_code": "subj.set_value(\"Date Received\", params['Date Received'])\nsubj.set_value(\"Obtained from\", params['Obtained from'])\nsubj.set_value(\"Passage\", params['Passage'])\nsubj.set_value(\"Cells In One Vial\", params['Cells In One Vial'])\n\n\nrequire_script 'lims_helper'\n\nvial_count = params['Number of Vials to Store']\nsubmit_to_fpro(subj, 'Lymphoblast', '7000000120', vial_count) do |s|\n s.set_value('Obtained From', subj.get_value('Obtained from'))\n s.set_value('Passage', subj.get_value('Passage'))\n s.set_value('Cells In One Vial', subj.get_value('Cells In One Vial'))\nend",

"btn_scale": "large",

"after_code_params": [

"UDPM-114",

"UDPM-1669",

"UDPM-1665",

"UDPM-1670",

"UDPM-1450"

]

}

],

"show_options": {

"top": 458,

"left": 168

}

},

{

"name": "Culture Failed",

"uuid": "UDPM-1093",

"owner": "UDPM-67",

"managers": "UDPM-49",

"performers": "UDPM-70",

"show_options": {

"top": 264,

"left": 521

},

"end_task": true

},

{

"name": "Request Entered",

"uuid": "UDPM-1084",

"owner": "UDPM-67",

"managers": "UDPM-49",

"performers": "UDPM-70",

"tools": [

{

"name": "Sample Information",

"uuid": "UDPM-1459",

"input_type": "UDPM-272",

"output_type": "UDPM-272",

"flags": 1,

"options": {

"udfs": [

"UDPM-6",

"UDPM-1385",

"UDPM-1010",

"UDPM-470",

"UDPM-1312"

]

}

}

],

"next_states": [

{

"state_name": "Sample Delivered to Order Processor",

"state_uuid": "UDPM-1087",

"btn_text": "Sample Delivered to Order Processor",

"btn_css": "rgb(13, 73, 123)",

"after_code": "self.next_state_performer = find_user('[redacted]')",

"btn_scale": "large"

}

],

"show_options": {

"top": 29,

"left": 43

}

},

{

"name": "Sample Delivered to Order Processor",

"uuid": "UDPM-1087",

"owner": "UDPM-67",

"managers": "UDPM-49",

"performers": "UDPM-70",

"next_states": [

{

"state_name": "Sample Shipped to Institution",

"state_uuid": "UDPM-1085",

"btn_text": "Sample Shipped to Institution",

"btn_css": "rgb(13, 73, 123)",

"after_code": "subj.set_value(\"Date Sent to Collaborator\", params['Date Sent to Collaborator'])\nsubj.set_value(\"Accession Number\", params['Accession Number'])\nself.next_state_performer = find_user('[redacted]')",

"btn_scale": "large",

"after_code_params": [

"UDPM-1389",

"UDPM-2869"

]

}

],

"em_performer_new_job": true,

"show_options": {

"top": 120,

"left": 274

}

},

{

"name": "Sample Shipped to Institution",

"uuid": "UDPM-1085",

"owner": "UDPM-67",

"managers": "UDPM-49",

"performers": "UDPM-70",

"duration": 90.0,

"next_states": [

{

"state_name": "Culture Failed",

"state_uuid": "UDPM-1093",

"btn_text": "Culture Failed",

"btn_css": "rgb(13, 73, 123)",

"after_code": "subj.set_value(\"Comments\", params['Comments'])",

"btn_scale": "large",

"after_code_params": [

"UDPM-5"

]

},

{

"state_name": "Cells Requested from Institution",

"state_uuid": "UDPM-1090",

"btn_text": "Vials Requested from Institution",

"btn_css": "rgb(13, 73, 123)",

"btn_scale": "large"

}

],

"em_performer_due_job": true,

"show_options": {

"top": 252,

"left": 32

}

}

],

"entry_point": "UDPM-1084",

"show_udfs": "UDPM-6,UDPM-1385,UDPM-470,UDPM-1010,UDPM-1312"

},{

"name": "MTA/ITA Amendments Workflow",

"uuid": "UDPM-223",

"subject_type": "UDPM-251",

"state_defs": [

{

"name": "Amended MTA/ITA",

"uuid": "UDPM-1109",

"owner": "UDPM-67",

"managers": "UDPM-45",

"performers": "UDPM-65",

"tools": [

{

"name": "Tool Name",

"uuid": "UDPM-1526",

"input_type": "UDPM-251",

"output_type": "UDPM-251",

"description": "Tool Description",

"flags": 1,

"options": {

"udfs": [

"UDPM-2032",

"UDPM-2693",

"UDPM-2694"

]

}

}

],

"next_states": [

{

"state_name": "Amendment Uploaded",

"state_uuid": "UDPM-1110",

"btn_text": "Upload Amendment",

"btn_css": "rgb(13, 73, 123)",

"btn_scale": "large"

}

],

"show_options": {

"top": 127,

"left": 326

}

},

{

"name": "Amendment Uploaded",

"uuid": "UDPM-1110",

"owner": "UDPM-67",

"managers": "UDPM-45",

"performers": "UDPM-65",

"show_options": {

"top": 301,

"left": 323

},

"end_task": true

}

],

"entry_point": "UDPM-1109"

},{

"name": "Blood Kit",

"uuid": "UDPM-19",

"subject_type": "UDPM-33",

"state_defs": [

{

"name": "Blood Kit Cancelled",

"uuid": "UDPM-356",

"owner": "UDPM-67",

"managers": "UDPM-9",

"performers": "UDPM-7",

"show_options": {

"top": 361,

"left": 154

},

"end_task": true

},

{

"name": "Blood Kit Prepared",

"uuid": "UDPM-351",

"owner": "UDPM-67",

"managers": "UDPM-9",

"performers": "UDPM-7",

"duration": 5.0,

"tools": [

{

"name": "Prepare Shipment",

"uuid": "UDPM-387",

"input_type": "UDPM-33",

"output_type": "UDPM-33",

"flags": 1,

"options": {

"udfs": [

"UDPM-6",

"UDPM-117",

"UDPM-118",

"UDPM-119",

"UDPM-51"

]

}

},

{

"name": "Print Label",

"uuid": "UDPM-763",

"input_type": "UDPM-33",

"output_type": "UDPM-33",

"description": "Print Kit Barcode Labels",

"flags": 2,

"options": {

"obj_type": "Printer",

"labels_count": 1,

"obj_name": "UDPM-4"

}

}

],

"next_states": [

{

"state_name": "Blood Kit Sent",

"state_uuid": "UDPM-353",

"btn_text": "Blood Kit Shipped",

"btn_css": "#456",

"after_code": "# Extra code here\n\nself.next_state_performer = find_user('[redacted]')\n#send_email(User.find_by_username('[redacted]'), find_email_template(\"Blood Kit Shipment\"), subj)",

"btn_scale": "large"

}

],

"show_options": {

"top": 48.991485595703125,

"left": 547.9886169433594

}

},

{

"name": "Blood Kit Processed",

"uuid": "UDPM-354",

"owner": "UDPM-67",

"managers": "UDPM-9",

"performers": "UDPM-7",

"show_options": {

"top": 187,

"left": 798.59375

},

"end_task": true

},

{

"name": "Blood Kit Requested",

"uuid": "UDPM-90",

"owner": "UDPM-67",

"managers": "UDPM-2",

"performers": "UDPM-46",

"duration": 5.0,

"tools": [

{

"name": "Phone Consent",

"uuid": "UDPM-98",

"input_type": "UDPM-33",

"output_type": "UDPM-33",

"flags": 1,

"options": {

"udfs": [

"UDPM-6",

"UDPM-2185",

"UDPM-419"

]

}

},

{

"name": "Print Labels",

"uuid": "UDPM-386",

"input_type": "UDPM-33",

"output_type": "UDPM-33",

"description": "Print Kit Barcode Labels",

"flags": 2,

"options": {

"obj_type": "Printer",

"labels_count": 1,

"obj_name": "UDPM-4"

}

},

{

"name": "Update Blood Kit Information",

"uuid": "UDPM-1113",

"input_type": "UDPM-33",

"output_type": "UDPM-33",

"flags": 1,

"options": {

"udfs": [

"UDPM-51",

"UDPM-2185",

"UDPM-1832",

"UDPM-5",

"UDPM-117",

"UDPM-120",

"UDPM-118",

"UDPM-119",

"UDPM-128",

"UDPM-114",

"UDPM-182",

"UDPM-419"

]

}

}

],

"next_states": [

{

"state_name": "Blood Kit Cancelled",

"state_uuid": "UDPM-356",

"btn_text": "Cancel",

"btn_css": "#456",

"after_code": "# Extra code here\nself.next_state_performer = User.curr_user\n",

"after_code_params": [

"UDPM-5"

]

},

{

"state_name": "Blood Kit Prepared",

"state_uuid": "UDPM-351",

"btn_text": "Prepare Blood Kit for Shipment",

"btn_css": "#456",

"after_code": "# Extra code here\nself.next_state_performer = find_user('[redacted]')"

},

{

"state_name": "Blood Kit Returned",

"state_uuid": "UDPM-951",

"btn_text": "Return Kit Request",

"btn_css": "#456",

"after_code": "send_email(subj.created_by, find_email_template(\"Kit Request Returned\"), subj)",

"before_code": "subj.set_value('Rationale/Reasoning', params['Rationale/Reasoning'])",

"btn_scale": "large",

"after_code_params": [

"UDPM-883"

]

}

],

"show_options": {

"top": 80,

"left": 35

}

},

{

"name": "Blood Kit Returned",

"uuid": "UDPM-951",

"owner": "UDPM-67",

"managers": "UDPM-9",

"performers": "UDPM-49",

"tools": [

{

"name": "Record Missing Information",

"uuid": "UDPM-1203",

"input_type": "UDPM-33",

"output_type": "UDPM-33",

"flags": 1,

"options": {

"udfs": [

"UDPM-51",

"UDPM-5",

"UDPM-182",

"UDPM-419"

]

}

}

],

"next_states": [

{

"state_name": "Blood Kit Requested",

"state_uuid": "UDPM-90",

"btn_text": "Request Blood Kit",

"btn_css": "#456"

}

],

"show_options": {

"top": 158.96875,

"left": 364

}

},

{

"name": "Blood Kit Sent",

"uuid": "UDPM-353",

"owner": "UDPM-67",

"managers": "UDPM-9",

"performers": "UDPM-7",

"tools": [

{

"name": "Blood Kit Received",

"uuid": "UDPM-777",

"input_type": "UDPM-33",

"output_type": "UDPM-86",

"description": "Prepare for Processing",

"flags": 0,

"after_code": "p = subj.get_value('Patient')\n if p\n recipients = []\n physician = p.get_value('Attending Physician')\n clinician = p.get_value('Primary Clinician')\n recipients << physician if physician\n recipients << clinician if clinician\n end\n\nif recipients.any?\n send_email(recipients, find_email_template(\"Notify Primary Care Clinician\"), subj)\nend\nsend_email(User.find_by_username('[redacted]'), find_email_template(\"Notify Primary Care Clinician\"), subj)\n",

"hide_button_if_not_condition": true,

"options": {

"obj_type": "SubjectType"

}

}

],

"next_states": [

{

"state_name": "Blood Kit Cancelled",

"state_uuid": "UDPM-356",

"btn_text": "Cancel",

"btn_css": "#456",

"after_code": "# Extra code here\n\nself.next_state_performer = find_user('[redacted]')",

"after_code_params": [

"UDPM-5"

]

},

{

"state_name": "Sample Sent to Collaborating Institution",

"state_uuid": "UDPM-687",

"btn_text": "Send Sample to Collaborating Institution for Lymphoblastoid Generation",

"btn_css": "#456",

"after_code": "# Extra code here\nself.next_state_performer = find_user('[redacted]')",

"btn_scale": "large",

"hide_button_if_not_condition": true,

"conditions": [

{

"name": "Blood Kit&rarr;ACD Tube for Lymphoblastoid Cell Line",

"condition": "Not Empty",

"value": "",

"message": "",

"subject_type": "UDPM-33",

"udf": "UDPM-1832"

}

]

},

{

"state_name": "Blood Kit Processed",

"state_uuid": "UDPM-354",

"btn_text": "Process Blood Kit",

"btn_css": "#456",

"after_code": "# Extra code here\nself.next_state_performer = find_user('[redacted]')",

"btn_scale": "large"

}

],

"show_options": {

"top": 249,

"left": 539

}

},

{

"name": "Sample Sent to Collaborating Institution",

"uuid": "UDPM-687",

"owner": "UDPM-67",

"managers": "UDPM-22",

"performers": "UDPM-4",

"tools": [

{

"name": "Request Lymphoblast Culture",

"uuid": "UDPM-1528",

"input_type": "UDPM-33",

"output_type": "UDPM-272",

"flags": 0,

"options": {

"obj_type": "SubjectType"

}

}

],

"show_options": {

"top": 411,

"left": 373

}

}

],

"entry_point": "UDPM-90",

"show_udfs": "UDPM-2185,UDPM-5,UDPM-1832"

},{

"name": "External Communication - DELETE",

"uuid": "UDPM-158",

"subject_type": "UDPM-190",

"state_defs": [

{

"name": "Initial Communication",

"uuid": "UDPM-744",

"owner": "UDPM-67",

"managers": "UDPM-9",

"performers": "UDPM-32",

"tools": [

{

"name": "Communication Information",

"uuid": "UDPM-908",

"input_type": "UDPM-190",

"output_type": "UDPM-190",

"flags": 1,

"options": {

"udfs": [

"UDPM-6",

"UDPM-1269",

"UDPM-191"

]

}

}

],

"next_states": [

{

"state_name": "Reply Information",

"state_uuid": "UDPM-745",

"btn_text": "Reply Received",

"btn_css": "#456",

"after_code_params": [

"UDPM-1956"

]

}

],

"show_options": {

"top": 163.97727966308594,

"left": 213.98574829101562

}

},

{

"name": "Reply Information",

"uuid": "UDPM-745",

"owner": "UDPM-67",

"managers": "UDPM-9",

"performers": "UDPM-32",

"show_options": {

"top": 206,

"left": 532

},

"end_task": true

}

],

"entry_point": "UDPM-744",

"ask_performer": true

},{

"name": "PII Patient File Uploads",

"uuid": "UDPM-146",

"subject_type": "UDPM-172",

"state_defs": [

{

"name": "Patient File(s) Upload",

"uuid": "UDPM-823",

"owner": "UDPM-67",

"managers": "UDPM-8",

"performers": "UDPM-5",

"tools": [

{

"name": "Notify Clinician",

"uuid": "UDPM-1028",

"input_type": "UDPM-172",

"output_type": "UDPM-172",

"flags": 5,

"options": {

"obj_type": "EmailTemplate",

"user_groups_ids": [

"UDPM-31"

],

"obj_name": "UDPM-3"

}

},

{

"name": "File Information",

"uuid": "UDPM-1024",

"input_type": "UDPM-172",

"output_type": "UDPM-172",

"flags": 1,

"options": {

"udfs": [

"UDPM-6",

"UDPM-1656",

"UDPM-1724",

"UDPM-1744",

"UDPM-1725",

"UDPM-2901",

"UDPM-1755",

"UDPM-2059"

]

}

}

],

"show_options": {

"top": 115,

"left": 261

},

"end_task": true

}

],

"entry_point": "UDPM-823",

"ask_performer": true,

"show_udfs": "UDPM-1724,UDPM-1744,UDPM-1656,UDPM-1725"

},{

"name": "Glycerol Stock Preparation",

"uuid": "UDPM-145",

"subject_type": "UDPM-171",

"state_defs": [

{

"name": "Prepare Stock",

"uuid": "UDPM-670",

"owner": "UDPM-67",

"managers": "UDPM-22",

"performers": "UDPM-21",

"tools": [

{

"name": "Glycerol Stock Information",

"uuid": "UDPM-825",

"input_type": "UDPM-171",

"output_type": "UDPM-171",

"flags": 1,

"options": {

"udfs": [

"UDPM-1701",

"UDPM-1656",

"UDPM-1332",

"UDPM-1933",

"UDPM-1907",

"UDPM-1669",

"UDPM-130",

"UDPM-1929",

"UDPM-2124",

"UDPM-1930",

"UDPM-1931",

"UDPM-2190",

"UDPM-1932",

"UDPM-5"

]

}

}

],

"next_states": [

{

"state_name": "Stored",

"state_uuid": "UDPM-671",

"btn_text": "Store Stock",

"btn_css": "#456",

"after_code": "require_script 'fpro_samples'\n\nvial_count = params['Number of Vials to Store']\npc = subj.get_value('Plasmid Construct')\npr = subj.get_value('Reagent Used')\n#evb = pc.get_value('Entry Vector Backbone')\n#dv = pc.get_value('Destination Vector')\nif User.curr_user.user_groups.map(&:name).join(',').include? \"Lab Personnel - Twinbrook\"\n submit_to_fpro(subj, 'Glycerol Stock', '223', vial_count) do |s|\n s.set_value('Bacteria Strain', subj.get_value('Bacteria Strain'))\n #s.set_value('Vector Information', pc.name)\n s.set_value('Insert Information', subj.get_value('Insert Information'))\n s.set_value('Species', subj.get_value('Glycerol Stock Species'))\n s.set_value('Obtained Date', subj.get_value('Obtained Date'))\n s.set_value('Obtained From', subj.get_value('Obtained from'))\n s.set_value('Growth Medium', subj.get_value('Growth Medium'))\n if pc\n s.set_value('Vector Information', pc.name)\n evb = pc.get_value('Entry Vector Backbone')\n dv = pc.get_value('Destination Vector')\n if evb\n s.set_value('Resistance Marker', evb.get_value('Resistance Marker'))\n elsif dv\n s.set_value('Resistance Marker', dv.get_value('Resistance Marker'))\n end\n elsif pr\n s.set_value('Vector Information', pr.name)\n s.set_value('Resistance Marker', pr.get_value('Resistance Marker'))\n end \n end\nelsif User.curr_user.user_groups.map(&:name).join(',').include? \"Lab Personnel - Building 50\"\n submit_to_fpro(subj, 'Glycerol Stock', '7000000622', vial_count) do |s|\n s.set_value('Bacteria Strain', subj.get_value('Bacteria Strain'))\n #s.set_value('Vector Information', pc.name)\n s.set_value('Insert Information', subj.get_value('Insert Information'))\n s.set_value('Species', subj.get_value('Glycerol Stock Species'))\n s.set_value('Obtained Date', subj.get_value('Obtained Date'))\n s.set_value('Obtained From', subj.get_value('Obtained from'))\n s.set_value('Growth Medium', subj.get_value('Growth Medium'))\n if pc\n s.set_value('Vector Information', pc.name)\n evb = pc.get_value('Entry Vector Backbone')\n dv = pc.get_value('Destination Vector')\n if evb\n s.set_value('Resistance Marker', evb.get_value('Resistance Marker'))\n elsif dv\n s.set_value('Resistance Marker', dv.get_value('Resistance Marker'))\n end\n elsif pr\n s.set_value('Vector Information', pr.name)\n s.set_value('Resistance Marker', pr.get_value('Resistance Marker'))\n end \n end\nelse\n raise(\"This script only allows users who belong to either Twinbrook Lab or Building 50 Lab, please contact the administrators\")\nend",

"btn_scale": "large",

"after_code_params": [

"UDPM-1450"

]

}

],

"show_options": {

"top": 60,

"left": 87.20001220703125

}

},

{

"name": "Stored",

"uuid": "UDPM-671",

"owner": "UDPM-67",

"managers": "UDPM-22",

"performers": "UDPM-21",

"show_options": {

"top": 242,

"left": 496.20001220703125

},

"end_task": true

}

],

"entry_point": "UDPM-670",

"ask_performer": true,

"show_udfs": "UDPM-2827,UDPM-2841,UDPM-2190,UDPM-1703,UDPM-1701,UDPM-130,UDPM-1332,UDPM-1929,UDPM-1933,UDPM-1656,UDPM-1473,UDPM-1932,UDPM-2895"

},{

"name": "Exome Analysis Results Workflow",

"uuid": "UDPM-154",

"subject_type": "UDPM-156",

"state_defs": [

{

"name": "Collaboration in Place",

"uuid": "UDPM-968",

"owner": "UDPM-67",

"managers": "UDPM-9",

"performers": "UDPM-49",

"tools": [

{

"name": "Request Material Transfer",

"uuid": "UDPM-1236",

"input_type": "UDPM-156",

"output_type": "UDPM-147",

"flags": 0,

"before_code": "gene = subj.get_value(\"Gene\")\n\nparams[:defaults] = {\n 'Gene for Collaboration'=> gene\n}",

"options": {

"obj_type": "SubjectType"

}

},

{

"name": "Request Zebrafish Model",

"uuid": "UDPM-1237",

"input_type": "UDPM-156",

"output_type": "UDPM-186",

"description": "[redacted] Lab",

"flags": 0,

"before_code": "gene = subj.get_value(\"Gene\")\nchr = subj.get_value(\"Chr\").delete('^0-9')\n\nparams[:defaults] = {\n 'Gene for Collaboration'=> gene,\n 'Chr'=> chr,\n}",

"options": {

"obj_type": "SubjectType"

}

},

{

"name": "Request Plasmid Construction",

"uuid": "UDPM-1241",

"input_type": "UDPM-156",

"output_type": "UDPM-193",

"description": "For collaboration",

"flags": 0,

"before_code": "gene = subj.get_value(\"Gene\")\n\nparams[:defaults] = {\n 'Gene'=> gene\n}",

"options": {

"obj_type": "SubjectType"

}

},

{

"name": "Request Information Transfer",

"uuid": "UDPM-1242",

"input_type": "UDPM-156",

"output_type": "UDPM-183",

"flags": 0,

"options": {

"obj_type": "SubjectType"

}

}

],

"show_options": {

"top": 158,

"left": 463.29998779296875

},

"end_task": true

},

{

"name": "Considered and Rejected",

"uuid": "UDPM-724",

"owner": "UDPM-67",

"managers": "UDPM-9",

"performers": "UDPM-2",

"show_options": {

"top": 152,

"left": 151.13330078125

},

"end_task": true

},

{

"name": "Consider for Research/Diagnosis",

"uuid": "UDPM-723",

"owner": "UDPM-67",

"managers": "UDPM-9",

"performers": "UDPM-2",

"tools": [

{

"name": "Request CLIA validation for the variant",

"uuid": "UDPM-983",

"input_type": "UDPM-156",

"output_type": "UDPM-167",

"flags": 0,

"options": {

"obj_type": "SubjectType"

}

},

{

"name": "Request Sanger Validation of Variant",

"uuid": "UDPM-1037",

"input_type": "UDPM-156",

"output_type": "UDPM-221",

"flags": 0,

"before_code": "gene = subj.get_value(\"Gene\")\nbuild = subj.get_value(\"Build\")\nchr = subj.get_value(\"Chr\").delete('^0-9')\nposition = subj.get_value(\"Position\")\nref = subj.get_value(\"Reference Allele\")\nvar = subj.get_value(\"Variant Allele\")\nseqChange = ref + '>' + var\ngenPos = 'chr' + chr + ':' + position\n\nparams[:defaults] = {\n 'Gene'=> gene,\n 'Build'=> build,\n 'Genomic Position'=> genPos,\n 'Sequence Change'=> seqChange\n}\n",

"options": {

"obj_type": "SubjectType"

}

},

{

"name": "Request Collaboration",

"uuid": "UDPM-1219",

"input_type": "UDPM-156",

"output_type": "UDPM-147",

"description": "Model organisms, etc",

"flags": 0,

"before_code": "gene = subj.get_value(\"Gene\")\n\nparams[:defaults] = {\n 'Gene for Collaboration'=> gene\n}",

"options": {

"obj_type": "SubjectType"

}

},

{

"name": "Request Zebrafish Model",

"uuid": "UDPM-1220",

"input_type": "UDPM-156",

"output_type": "UDPM-186",

"description": "[redacted] Lab",

"flags": 0,

"before_code": "gene = subj.get_value(\"Gene\")\nchr = subj.get_value(\"Chr\").delete('^0-9')\n\nparams[:defaults] = {\n 'Gene for Collaboration'=> gene,\n 'Chr'=> chr,\n}",

"options": {

"obj_type": "SubjectType"

}

}

],

"show_options": {

"top": 244,

"left": 463

},

"end_task": true

},

{

"name": "Glycosylation Variant",

"uuid": "UDPM-999",

"owner": "UDPM-67",

"managers": "UDPM-38",

"performers": "UDPM-38",

"show_options": {

"top": 261,

"left": 150.13333129882812

},

"end_task": true

},

{

"name": "Incidental Finding",

"uuid": "UDPM-954",

"owner": "UDPM-67",

"managers": "UDPM-9",

"performers": "UDPM-30",

"show_options": {

"top": 322,

"left": 152.26666259765625

},

"end_task": true

},

{

"name": "Reviewed, Possible Impact",

"uuid": "UDPM-965",

"owner": "UDPM-67",

"managers": "UDPM-9",

"performers": "UDPM-49",

"show_options": {

"top": 100,

"left": 149.933349609375

},

"end_task": true

},

{

"name": "RFA Variant",

"uuid": "UDPM-952",

"owner": "UDPM-67",

"managers": "UDPM-27",

"performers": "UDPM-30",

"show_options": {

"top": 378,

"left": 152.13333129882812

},

"end_task": true

},

{

"name": "Sequence Analysis Completed -- Pending Review",

"uuid": "UDPM-786",

"owner": "UDPM-67",

"managers": "UDPM-9",

"performers": "UDPM-30",

"tools": [

{

"name": "Consider for Research/Diagnosis",

"uuid": "UDPM-1508",

"input_type": "UDPM-156",

"output_type": "UDPM-156",

"flags": 6,

"before_code": "advance_workflow(\"Exome Analysis Results Workflow\",\"Consider for Research/Diagnosis\",subj)",

"no_conformation": true

},

{

"name": "Variant Information",

"uuid": "UDPM-1500",

"input_type": "UDPM-156",

"output_type": "UDPM-156",

"flags": 1,

"options": {

"udfs": [

"UDPM-2864",

"UDPM-2147",

"UDPM-1868",

"UDPM-1874",

"UDPM-2195",

"UDPM-1474",

"UDPM-2155",

"UDPM-2744",

"UDPM-1277",

"UDPM-1689",

"UDPM-1690",

"UDPM-1477",

"UDPM-1478",

"UDPM-1479",

"UDPM-1688",

"UDPM-1879",

"UDPM-1482",

"UDPM-1507",

"UDPM-1483",

"UDPM-1484",

"UDPM-1485",

"UDPM-1880",

"UDPM-1881",

"UDPM-1504",

"UDPM-1505",

"UDPM-1506",

"UDPM-1480"

]

}

}

],

"show_options": {

"top": 20,

"left": 154

},

"end_task": true

},

{

"name": "Variant involved in Diagnosis",

"uuid": "UDPM-953",

"owner": "UDPM-67",

"managers": "UDPM-9",

"performers": "UDPM-49",

"show_options": {

"top": 204,

"left": 149.933349609375

},

"end_task": true

}

],

"entry_point": "UDPM-786",

"ask_performer": true,

"show_udfs": "UDPM-2613,UDPM-2195,UDPM-2614,UDPM-1689,UDPM-1477,UDPM-1690,UDPM-1874,UDPM-2162,UDPM-2168,UDPM-2156,UDPM-2164,UDPM-2161,UDPM-2169,UDPM-1868,UDPM-2152,UDPM-2163,UDPM-2170,UDPM-2173,UDPM-2179,UDPM-2180,UDPM-2159,UDPM-2165,UDPM-2174,UDPM-2172,UDPM-2182,UDPM-2175,UDPM-2160,UDPM-2176,UDPM-2178,UDPM-2181,UDPM-2183,UDPM-2559,UDPM-2544,UDPM-1688,UDPM-1480,UDPM-1277,UDPM-2171,UDPM-2166,UDPM-2864,UDPM-2023,UDPM-2167,UDPM-2177"

},{

"name": "Generate iPS Workflow",

"uuid": "UDPM-27",

"subject_type": "UDPM-41",

"state_defs": [

{

"name": "Characterization",

"uuid": "UDPM-739",

"owner": "UDPM-67",

"managers": "UDPM-22",

"performers": "UDPM-43",

"duration": 2.0,

"tools": [

{

"name": "Characterization",

"uuid": "UDPM-907",

"input_type": "UDPM-41",

"output_type": "UDPM-41",

"flags": 1,

"options": {

"udfs": [

"UDPM-2004",

"UDPM-2005",

"UDPM-2006",

"UDPM-2007",

"UDPM-2009",

"UDPM-2011",

"UDPM-2010",

"UDPM-2008"

]

}

}

],

"next_states": [

{

"state_name": "Discard",

"state_uuid": "UDPM-743",

"btn_text": "Discard",

"btn_css": "#456",

"after_code": "subj.set_value('Rationale/Reasoning', params['Rationale/Reasoning'] )",

"after_code_params": [

"UDPM-883"

]

},

{

"state_name": "Embryoid Body Formation",

"state_uuid": "UDPM-741",

"btn_text": "Embryoid Body Formation",

"btn_css": "#456",

"after_code": "self.next_state_performer = User.curr_user",

"btn_scale": "large"

}

],

"show_options": {

"top": 743,

"left": 309

}

},

{

"name": "Discard",

"uuid": "UDPM-743",

"owner": "UDPM-67",

"managers": "UDPM-22",

"performers": "UDPM-43",

"show_options": {

"top": 348,

"left": 705

},

"end_task": true

},

{

"name": "Embryoid Body Formation",

"uuid": "UDPM-741",

"owner": "UDPM-67",

"managers": "UDPM-22",

"performers": "UDPM-43",

"duration": 7.0,

"tools": [

{

"name": "Formation Information",

"uuid": "UDPM-974",

"input_type": "UDPM-41",

"output_type": "UDPM-41",

"flags": 1,

"options": {

"udfs": [

"UDPM-2012",

"UDPM-2014"

]

}

}

],

"next_states": [

{

"state_name": "Discard",

"state_uuid": "UDPM-743",

"btn_text": "Discard",

"btn_css": "#456",

"after_code": "subj.set_value('Rationale/Reasoning', params['Rationale/Reasoning'] )",

"after_code_params": [

"UDPM-883"

]

},

{

"state_name": "Karyotyping",

"state_uuid": "UDPM-740",

"btn_text": "Begin Karyotyping",

"btn_css": "#456",

"after_code": "self.next_state_performer = User.curr_user"

}

],

"show_options": {

"top": 749,

"left": 676

}

},

{

"name": "Infection of Cells",

"uuid": "UDPM-735",

"owner": "UDPM-67",

"managers": "UDPM-22",

"performers": "UDPM-43",

"duration": 7.0,

"tools": [

{

"name": "General Culture Information",

"uuid": "UDPM-978",

"input_type": "UDPM-41",

"output_type": "UDPM-41",

"flags": 1,

"options": {

"udfs": [

"UDPM-458",

"UDPM-70",

"UDPM-1668",

"UDPM-5"

]

}

},

{

"name": "Infection Information",

"uuid": "UDPM-903",

"input_type": "UDPM-41",

"output_type": "UDPM-41",

"flags": 1,

"options": {

"udfs": [

"UDPM-2000",

"UDPM-1999",

"UDPM-1998"

]

}

}

],

"next_states": [

{

"state_name": "Prepare Feeder Cells",

"state_uuid": "UDPM-736",

"btn_text": "Begin Feeder Cell Preparation",

"btn_css": "#456",

"after_code": "self.next_state_performer = User.curr_user",

"btn_scale": "large",

"hide_button_if_not_condition": true,

"conditions": [

{

"name": "iPS Cells&rarr;Type of Cell",

"condition": "=",

"value": "Feeder-dependent iPS",

"message": "",

"subject_type": "UDPM-41",

"udf": "UDPM-458"

}

]

},

{

"state_name": "Discard",

"state_uuid": "UDPM-743",

"btn_text": "Discard",

"btn_css": "#456",

"after_code": "subj.set_value('Rationale/Reasoning', params['Rationale/Reasoning'] )",

"after_code_params": [

"UDPM-883"

]

},

{

"state_name": "Trypsinization of Cells",

"state_uuid": "UDPM-737",

"btn_text": "Trypsinize Cells",

"btn_css": "rgb(13, 73, 123)",

"btn_scale": "large",

"hide_button_if_not_condition": true,

"conditions": [

{

"name": "iPS Cells&rarr;Type of Cell",

"condition": "=",

"value": "Feeder-free iPS",

"message": "",

"subject_type": "UDPM-41",

"udf": "UDPM-458"

}

]

}

],

"show_options": {

"top": 10,

"left": 559

}

},

{

"name": "Karyotyping",

"uuid": "UDPM-740",

"owner": "UDPM-67",

"managers": "UDPM-22",

"performers": "UDPM-43",

"duration": 1.0,

"tools": [

{

"name": "Karyotyping Information",

"uuid": "UDPM-975",

"input_type": "UDPM-41",

"output_type": "UDPM-41",

"flags": 1,

"options": {

"udfs": [

"UDPM-2013",

"UDPM-2015"

]

}

}

],

"next_states": [

{

"state_name": "Discard",

"state_uuid": "UDPM-743",

"btn_text": "Discard",

"btn_css": "#456",

"after_code": "subj.set_value('Rationale/Reasoning', params['Rationale/Reasoning'] )",

"after_code_params": [

"UDPM-883"

]

},

{

"state_name": "Selection of Final Clones",

"state_uuid": "UDPM-742",

"btn_text": "Select Final Clones",

"btn_css": "#456",

"after_code": "self.next_state_performer = User.curr_user",

"btn_scale": "large"

}

],

"show_options": {

"top": 716,

"left": 1041

}

},

{

"name": "Passage",

"uuid": "UDPM-783",

"owner": "UDPM-67",

"managers": "UDPM-22",

"performers": "UDPM-43",

"tools": [

{

"name": "Passage Information",

"uuid": "UDPM-977",

"input_type": "UDPM-41",

"output_type": "UDPM-41",

"flags": 1,

"options": {

"udfs": [

"UDPM-1944",

"UDPM-1945",

"UDPM-1946",

"UDPM-1947"

]

}

}

],

"next_states": [

{

"state_name": "Discard",

"state_uuid": "UDPM-743",

"btn_text": "Discard",

"btn_css": "#456",

"after_code": "subj.set_value('Rationale/Reasoning', params['Rationale/Reasoning'] )",

"after_code_params": [

"UDPM-883"

]

},

{

"state_name": "Characterization",

"state_uuid": "UDPM-739",

"btn_text": "Freeze Final Passage and Characterize",

"btn_css": "#456",

"after_code": "self.next_state_performer = User.curr_user\n\nrequire_script 'lims_helper'\n\nsec = subj.get_value(\"Date of Second Passage\")\nthird = subj.get_value(\"Date of Third Passage\")\nfourth = subj.get_value(\"Date of Fourth Passage\")\nfifth = subj.get_value(\"Date of Fifth Passage\")\n\nvial_count = params['Number of Vials to Store']\nself.next_state_performer = User.curr_user\nsubmit_to_fpro(subj, 'iPS cells', '7000000123', vial_count) do |s|\n if fifth.present?\n s.set_value(\"Passage\", \"P5\")\n elsif fourth.present?\n s.set_value(\"Passage\", \"P4\")\n elsif third.present?\n s.set_value(\"Passage\", \"P3\")\n elsif sec.present?\n s.set_value(\"Passage\", \"P2\")\n else\n s.set_value(\"Passage\", \"P1\")\n end\nend\n",

"btn_scale": "large",

"after_code_params": [

"UDPM-1450"

]

}

],

"show_options": {

"top": 641.9829559326172,

"left": 72.9801025390625

}

},

{

"name": "Prepare Feeder Cells",

"uuid": "UDPM-736",

"owner": "UDPM-67",

"managers": "UDPM-22",

"performers": "UDPM-43",

"duration": 1.0,

"tools": [

{

"name": "Feeder Cell Information",

"uuid": "UDPM-904",

"input_type": "UDPM-41",

"output_type": "UDPM-41",

"flags": 1,

"options": {

"udfs": [

"UDPM-459",

"UDPM-1939",

"UDPM-2001"

]

}

}

],

"next_states": [

{

"state_name": "Trypsinization of Cells",

"state_uuid": "UDPM-737",

"btn_text": "Trypsinize Cells",

"btn_css": "#456",

"after_code": "self.next_state_performer = User.curr_user"

},

{

"state_name": "Discard",

"state_uuid": "UDPM-743",

"btn_text": "Discard",

"btn_css": "#456",

"after_code": "subj.set_value('Rationale/Reasoning', params['Rationale/Reasoning'] )",

"after_code_params": [

"UDPM-883"

]

}

],

"show_options": {

"top": 59.98863220214844,

"left": 92.99715042114258

}

},

{

"name": "Selection of Clones",

"uuid": "UDPM-738",

"owner": "UDPM-67",

"managers": "UDPM-22",

"performers": "UDPM-43",

"duration": 30.0,

"tools": [

{

"name": "Clone Selection",

"uuid": "UDPM-906",

"input_type": "UDPM-41",

"output_type": "UDPM-41",

"flags": 1,

"options": {

"udfs": [

"UDPM-1941",

"UDPM-2002",

"UDPM-2003",

"UDPM-1943"

]

}

}

],

"next_states": [

{

"state_name": "Passage",

"state_uuid": "UDPM-783",

"btn_text": "Freeze First Passage",

"btn_css": "#456",

"after_code": "self.next_state_performer = User.curr_user\n\nrequire_script 'lims_helper'\n\nvial_count = params['Number of Vials to Store']\nself.next_state_performer = User.curr_user\nsubmit_to_fpro(subj, 'iPS cells', '7000000123', vial_count) do |s|\n s.set_value(\"Passage\", 'P1')\nend\n",

"btn_scale": "large",

"after_code_params": [

"UDPM-1450"

]

},

{

"state_name": "Discard",

"state_uuid": "UDPM-743",

"btn_text": "Discard",

"btn_css": "#456",

"after_code": "subj.set_value('Rationale/Reasoning', params['Rationale/Reasoning'] )",

"after_code_params": [

"UDPM-883"

]

}

],

"show_options": {

"top": 462,

"left": 54

}

},

{

"name": "Selection of Final Clones",

"uuid": "UDPM-742",

"owner": "UDPM-67",

"managers": "UDPM-22",

"performers": "UDPM-43",

"tools": [

{

"name": "Final Clone Information",

"uuid": "UDPM-976",

"input_type": "UDPM-41",

"output_type": "UDPM-41",

"flags": 1,

"options": {

"udfs": [

"UDPM-1942"

]

}

}

],

"next_states": [

{

"state_name": "Discard",

"state_uuid": "UDPM-743",

"btn_text": "Discard",

"btn_css": "#456",

"after_code": "subj.set_value('Rationale/Reasoning', params['Rationale/Reasoning'] )",

"after_code_params": [

"UDPM-883"

]

},

{

"state_name": "Stored",

"state_uuid": "UDPM-378",

"btn_text": "Freeze Cells",

"btn_css": "#456",

"after_code": "require_script 'lims_helper'\nsubj.set_value('Passage', params['Passage'])\nvial_count = params['Number of Vials to Store']\nself.next_state_performer = User.curr_user\nsubmit_to_fpro(subj, 'iPS cells', '7000000123', vial_count) do |s|\n s.set_value('Passage', params['Passage'])\nend\n\n",

"btn_scale": "large",

"after_code_params": [

"UDPM-1450",

"UDPM-1665"

]

}

],

"show_options": {

"top": 497,

"left": 1122

}

},

{

"name": "Stored",

"uuid": "UDPM-378",

"owner": "UDPM-3",

"managers": "UDPM-22",

"performers": "UDPM-43",

"tools": [

{

"name": "Ship to Collaborator",

"uuid": "UDPM-1138",

"input_type": "UDPM-41",

"output_type": "UDPM-147",

"flags": 0,

"options": {

"obj_type": "SubjectType"

}

}

],

"show_options": {

"top": 308.8806610107422,

"left": 1216.8635559082031

},

"end_task": true

},

{

"name": "Trypsinization of Cells",

"uuid": "UDPM-737",

"owner": "UDPM-67",

"managers": "UDPM-22",

"performers": "UDPM-43",

"duration": 20.0,

"tools": [

{

"name": "Trypsinization Information",

"uuid": "UDPM-905",

"input_type": "UDPM-41",

"output_type": "UDPM-41",

"flags": 1,

"options": {

"udfs": [

"UDPM-1940"

]

}

}

],

"next_states": [

{

"state_name": "Discard",

"state_uuid": "UDPM-743",

"btn_text": "Discard",

"btn_css": "#456",

"after_code": "subj.set_value('Rationale/Reasoning', params['Rationale/Reasoning'] )",

"after_code_params": [

"UDPM-883"

]

},

{

"state_name": "Selection of Clones",

"state_uuid": "UDPM-738",

"btn_text": "Select Clones",

"btn_css": "#456",

"after_code": "self.next_state_performer = User.curr_user"

}

],

"show_options": {

"top": 251,

"left": 71

}

}

],

"entry_point": "UDPM-735",

"show_udfs": "UDPM-6,UDPM-463"

},{

"name": "Patient Visit Planning v3 - DELETE/RENAME",

"uuid": "UDPM-161",

"subject_type": "UDPM-192",

"state_defs": [

{

"name": "Accepted ",

"uuid": "UDPM-809",

"owner": "UDPM-67",

"managers": "UDPM-9",

"performers": "UDPM-20",

"duration": 21.0,

"next_states": [

{

"state_name": "Peds Scheduling",

"state_uuid": "UDPM-754",

"btn_text": "Peds Scheduling",

"btn_css": "#456",

"before_code": "# Script to run Before Transition is executed\nparams[:ask_next_performer] = true",

"hide_button_if_not_condition": true,

"conditions": [

{

"name": "Patient&rarr;Patient Case",

"condition": "=",

"value": "Pediatric",

"message": "",

"subject_type": "UDPM-1",

"udf": "UDPM-43"

}

]

},

{

"state_name": "Adult Scheduling",

"state_uuid": "UDPM-810",

"btn_text": "Adult Scheduling",

"btn_css": "#456",

"before_code": "# Script to run Before Transition is executed\nparams[:ask_next_performer] = true",

"hide_button_if_not_condition": true,

"conditions": [

{

"name": "Patient&rarr;Patient Case",

"condition": "=",

"value": "Adult",

"message": "",

"subject_type": "UDPM-1",

"udf": "UDPM-43"

}

]

}

],

"show_options": {

"top": 25,

"left": 164

}

},

{

"name": "Adult Scheduling",

"uuid": "UDPM-810",

"owner": "UDPM-67",

"managers": "UDPM-9",

"performers": "UDPM-20",

"duration": 21.0,

"tools": [

{

"name": "Create ATV",

"uuid": "UDPM-1010",

"input_type": "UDPM-192",

"output_type": "UDPM-146",

"flags": 0,

"options": {

"obj_type": "SubjectType"

}

},

{

"name": "Create - Send Accept Letter",

"uuid": "UDPM-1011",

"input_type": "UDPM-192",

"output_type": "UDPM-87",

"flags": 0,

"options": {

"obj_type": "SubjectType"

}

},

{

"name": "Create Task",

"uuid": "UDPM-1012",

"input_type": "UDPM-192",

"output_type": "UDPM-164",

"flags": 0,

"options": {

"obj_type": "SubjectType"

}

},

{

"name": "Checklist",

"uuid": "UDPM-1013",

"input_type": "UDPM-192",

"output_type": "UDPM-192",

"flags": 1,

"options": {

"udfs": [

"UDPM-6",

"UDPM-1110",

"UDPM-1162",

"UDPM-1350",

"UDPM-1360",

"UDPM-1159",

"UDPM-1358",

"UDPM-1362",

"UDPM-1367",

"UDPM-1369",

"UDPM-1371",

"UDPM-49",

"UDPM-1154",

"UDPM-1120",

"UDPM-1155",

"UDPM-136"

]

}

}

],

"next_states": [

{

"state_name": "Scheduling Complete",

"state_uuid": "UDPM-756",

"btn_text": "Scheduling Complete",

"btn_css": "#456",

"before_code": "# Script to run Before Transition is executed\nparams[:ask_next_performer] = true"

}

],

"show_options": {

"top": 4.9886322021484375,

"left": 646.9999694824219

}

},

{

"name": "Assessment Tools",

"uuid": "UDPM-758",

"owner": "UDPM-67",

"managers": "UDPM-9",

"performers": "UDPM-20",

"tools": [

{

"name": "Create Task",

"uuid": "UDPM-926",

"input_type": "UDPM-192",

"output_type": "UDPM-164",

"flags": 0,

"options": {

"obj_type": "SubjectType"

}

},

{

"name": "1 - Cardiology",

"uuid": "UDPM-934",

"input_type": "UDPM-192",

"output_type": "UDPM-192",

"flags": 1,

"options": {

"udfs": [

"UDPM-6",

"UDPM-1110",

"UDPM-1192",

"UDPM-1193",

"UDPM-1194",

"UDPM-1195",

"UDPM-1375",

"UDPM-1543"

]

}

},

{

"name": "2 - Neurological",

"uuid": "UDPM-935",

"input_type": "UDPM-192",

"output_type": "UDPM-192",

"flags": 1,

"options": {

"udfs": [

"UDPM-6",

"UDPM-1110",

"UDPM-1186",

"UDPM-1136",

"UDPM-1180",

"UDPM-1179",

"UDPM-1219",

"UDPM-1181",

"UDPM-1183",

"UDPM-1185",

"UDPM-1221",

"UDPM-1375",

"UDPM-1544"

]

}

},

{

"name": "3 - Nutrition",

"uuid": "UDPM-936",

"input_type": "UDPM-192",

"output_type": "UDPM-192",

"flags": 1,

"options": {

"udfs": [

"UDPM-6",

"UDPM-1110",

"UDPM-1191",

"UDPM-1194",

"UDPM-1188",

"UDPM-1190",

"UDPM-1545"

]

}

},

{

"name": "4 - Rehab",

"uuid": "UDPM-938",

"input_type": "UDPM-192",

"output_type": "UDPM-192",

"flags": 1,

"options": {

"udfs": [

"UDPM-6",

"UDPM-1110",

"UDPM-1196",

"UDPM-1220",

"UDPM-1198",

"UDPM-1200",

"UDPM-1548"

]

}

},

{

"name": "5 - Respiratory",

"uuid": "UDPM-939",

"input_type": "UDPM-192",

"output_type": "UDPM-192",

"flags": 1,

"options": {

"udfs": [

"UDPM-6",

"UDPM-1110",

"UDPM-1178",

"UDPM-1133",

"UDPM-1132",

"UDPM-1134",

"UDPM-1135",

"UDPM-1138",

"UDPM-1375",

"UDPM-1547"

]

}

},

{

"name": "6 - Safety & Equip.",

"uuid": "UDPM-940",

"input_type": "UDPM-192",

"output_type": "UDPM-192",

"flags": 1,

"options": {

"udfs": [

"UDPM-6",

"UDPM-1110",

"UDPM-1202",

"UDPM-1220",

"UDPM-1198",

"UDPM-1199",

"UDPM-1375",

"UDPM-1550"

]

}

},

{

"name": "7 - Consultation Request",

"uuid": "UDPM-941",

"input_type": "UDPM-192",

"output_type": "UDPM-192",

"flags": 1,

"options": {

"udfs": [

"UDPM-6",

"UDPM-1110",

"UDPM-1248",

"UDPM-945",

"UDPM-1229",

"UDPM-1230",

"UDPM-1231",

"UDPM-1232",

"UDPM-1233",

"UDPM-1234",

"UDPM-1117",

"UDPM-1549"

]

}

}

],

"next_states": [

{

"state_name": "Schedule - Imminent",

"state_uuid": "UDPM-757",

"btn_text": "Schedule - Imminent",

"btn_css": "#456",

"before_code": "# Script to run Before Transition is executed\nparams[:ask_next_performer] = true"

},

{

"state_name": "Scheduling Complete",

"state_uuid": "UDPM-756",

"btn_text": "Assessment Tools - Complete",

"btn_css": "#456",

"before_code": "# Script to run Before Transition is executed\nparams[:ask_next_performer] = true"

},

{

"state_name": "Schedule - Near",

"state_uuid": "UDPM-759",

"btn_text": "Schedule - Near",

"btn_css": "#456",

"before_code": "# Script to run Before Transition is executed\nparams[:ask_next_performer] = true"

},

{

"state_name": "Schedule - Far Out",

"state_uuid": "UDPM-755",

"btn_text": "Schedule - Far Out",

"btn_css": "#456",

"before_code": "# Script to run Before Transition is executed\nparams[:ask_next_performer] = true"

}

],

"show_options": {

"top": 615,

"left": 880

}

},

{

"name": "Peds Scheduling",

"uuid": "UDPM-754",

"owner": "UDPM-67",

"managers": "UDPM-9",

"performers": "UDPM-20",

"next_states": [

{

"state_name": "Schedule - Near",

"state_uuid": "UDPM-759",

"btn_text": "Schedule - Near",

"btn_css": "#456",

"before_code": "# Script to run Before Transition is executed\nparams[:ask_next_performer] = true"

},

{

"state_name": "Schedule - Far Out",

"state_uuid": "UDPM-755",

"btn_text": "Schedule - Far Out",

"btn_css": "#456",

"before_code": "# Script to run Before Transition is executed\nparams[:ask_next_performer] = true"

},

{

"state_name": "Schedule - Imminent",

"state_uuid": "UDPM-757",

"btn_text": "Schedule - Imminent",

"btn_css": "#456",

"before_code": "# Script to run Before Transition is executed\nparams[:ask_next_performer] = true"

}

],

"show_options": {

"top": 206,

"left": 147

}

},

{

"name": "Schedule - Far Out",

"uuid": "UDPM-755",

"owner": "UDPM-67",

"managers": "UDPM-9",

"performers": "UDPM-20",

"tools": [

{

"name": "Checklist",

"uuid": "UDPM-918",

"input_type": "UDPM-192",

"output_type": "UDPM-192",

"flags": 1,

"options": {

"udfs": [

"UDPM-6",

"UDPM-1110",

"UDPM-1561",

"UDPM-1613",

"UDPM-1614",

"UDPM-1632",

"UDPM-1630",

"UDPM-1368",

"UDPM-1584",

"UDPM-1563",

"UDPM-1162",

"UDPM-1163",

"UDPM-1143",

"UDPM-1350",

"UDPM-1126",

"UDPM-1360",

"UDPM-1159",

"UDPM-1358",

"UDPM-1359",

"UDPM-1361",

"UDPM-1160",

"UDPM-1364",

"UDPM-1362",

"UDPM-1366",

"UDPM-1367",

"UDPM-1369",

"UDPM-1370",

"UDPM-1371",

"UDPM-1372",

"UDPM-1157",

"UDPM-1130",

"UDPM-1111",

"UDPM-1153",

"UDPM-1161",

"UDPM-1148"

]

}

},

{

"name": "Create ATV",

"uuid": "UDPM-919",

"input_type": "UDPM-192",

"output_type": "UDPM-146",

"flags": 0,

"options": {

"obj_type": "SubjectType"

}

},

{

"name": "Create - Send Acceptance Letter",

"uuid": "UDPM-920",

"input_type": "UDPM-192",

"output_type": "UDPM-87",

"flags": 0,

"options": {

"obj_type": "SubjectType"

}

},

{

"name": "Create Task",

"uuid": "UDPM-921",

"input_type": "UDPM-192",

"output_type": "UDPM-164",

"flags": 0,

"options": {

"obj_type": "SubjectType"

}

}

],

"next_states": [

{

"state_name": "Scheduling Complete",

"state_uuid": "UDPM-756",

"btn_text": "Far Out Schedule - Complete",

"btn_css": "#456"

},

{

"state_name": "Schedule - Near",

"state_uuid": "UDPM-759",

"btn_text": "Schedule - Near",

"btn_css": "#456"

},

{

"state_name": "Schedule - Imminent",

"state_uuid": "UDPM-757",

"btn_text": "Schedule - Imminent",

"btn_css": "#456",

"before_code": "# Script to run Before Transition is executed\nparams[:ask_next_performer] = true"

},

{

"state_name": "Assessment Tools",

"state_uuid": "UDPM-758",

"btn_text": "Assessment Tools",

"btn_css": "#456",

"before_code": "# Script to run Before Transition is executed\nparams[:ask_next_performer] = true"

}

],

"show_options": {

"top": 118,

"left": 506

}

},

{

"name": "Schedule - Imminent",

"uuid": "UDPM-757",

"owner": "UDPM-67",

"managers": "UDPM-9",

"performers": "UDPM-20",

"tools": [

{

"name": "Checklist",

"uuid": "UDPM-932",

"input_type": "UDPM-192",

"output_type": "UDPM-192",

"flags": 1,

"options": {

"udfs": [

"UDPM-6",

"UDPM-1110",

"UDPM-1225",

"UDPM-1239",

"UDPM-1240",

"UDPM-1241",

"UDPM-1242",

"UDPM-1243",

"UDPM-1244",

"UDPM-1245",

"UDPM-1246",

"UDPM-1247",

"UDPM-191"

]

}

},

{

"name": "Create Task",

"uuid": "UDPM-925",

"input_type": "UDPM-192",

"output_type": "UDPM-164",

"flags": 0,

"options": {

"obj_type": "SubjectType"

}

}

],

"next_states": [

{

"state_name": "Scheduling Complete",

"state_uuid": "UDPM-756",

"btn_text": "Imminent Schedule - Complete",

"btn_css": "#456",

"before_code": "# Script to run Before Transition is executed\nparams[:ask_next_performer] = true"

},

{

"state_name": "Schedule - Far Out",

"state_uuid": "UDPM-755",

"btn_text": "Schedule - Far Out",

"btn_css": "#456",

"before_code": "# Script to run Before Transition is executed\nparams[:ask_next_performer] = true"

},

{

"state_name": "Schedule - Near",

"state_uuid": "UDPM-759",

"btn_text": "Schedule - Near",

"btn_css": "#456",

"before_code": "# Script to run Before Transition is executed\nparams[:ask_next_performer] = true"

},

{

"state_name": "Assessment Tools",

"state_uuid": "UDPM-758",

"btn_text": "Assessment Tools",

"btn_css": "#456",

"before_code": "# Script to run Before Transition is executed\nparams[:ask_next_performer] = true"

}

],

"show_options": {

"top": 731,

"left": 209

}

},

{

"name": "Schedule - Near",

"uuid": "UDPM-759",

"owner": "UDPM-67",

"managers": "UDPM-9",

"performers": "UDPM-20",

"tools": [

{

"name": "Checklist",

"uuid": "UDPM-933",

"input_type": "UDPM-192",

"output_type": "UDPM-192",

"flags": 1,

"options": {

"udfs": [

"UDPM-6",

"UDPM-1110",

"UDPM-1225",

"UDPM-191",

"UDPM-1621",

"UDPM-1634",

"UDPM-1635",

"UDPM-1595",

"UDPM-1592",

"UDPM-1612",

"UDPM-1610",

"UDPM-1637",

"UDPM-1591",

"UDPM-1623",

"UDPM-1609",

"UDPM-1567",

"UDPM-1611",

"UDPM-1570"

]

}

},

{

"name": "Create Task",

"uuid": "UDPM-927",

"input_type": "UDPM-192",

"output_type": "UDPM-164",

"flags": 0,

"options": {

"obj_type": "SubjectType"

}

}

],

"next_states": [

{

"state_name": "Schedule - Far Out",

"state_uuid": "UDPM-755",

"btn_text": "Scheduke - Far Out",

"btn_css": "#456"

},

{

"state_name": "Schedule - Imminent",

"state_uuid": "UDPM-757",

"btn_text": "Schedule - Imminent",

"btn_css": "#456",

"before_code": "# Script to run Before Transition is executed\nparams[:ask_next_performer] = true"

},

{

"state_name": "Assessment Tools",

"state_uuid": "UDPM-758",

"btn_text": "Assessment Tools",

"btn_css": "#456",

"before_code": "# Script to run Before Transition is executed\nparams[:ask_next_performer] = true"

},

{

"state_name": "Scheduling Complete",

"state_uuid": "UDPM-756",

"btn_text": "Near Schedule - Complete",

"btn_css": "#456",

"before_code": "# Script to run Before Transition is executed\nparams[:ask_next_performer] = true"

}

],

"show_options": {

"top": 352,

"left": 508

}

},

{

"name": "Scheduling Complete",

"uuid": "UDPM-756",

"owner": "UDPM-67",

"managers": "UDPM-9",

"performers": "UDPM-20",

"show_options": {

"top": 191,

"left": 1137

},

"end_task": true

}

],

"entry_point": "UDPM-809",

"ask_performer": true

},{

"name": "Patient Research Meeting",

"uuid": "UDPM-106",

"subject_type": "UDPM-127",

"state_defs": [

{

"name": "Case Closed",

"uuid": "UDPM-470",

"owner": "UDPM-67",

"managers": "UDPM-9",

"performers": "UDPM-9",

"show_options": {

"top": 38,

"left": 623

},

"end_task": true

},

{

"name": "Clinical Decision",

"uuid": "UDPM-469",

"owner": "UDPM-67",

"managers": "UDPM-9",

"performers": "UDPM-9",

"tools": [

{

"name": "Meeting Evaluation",

"uuid": "UDPM-757",

"input_type": "UDPM-127",

"output_type": "UDPM-127",

"flags": 1,

"options": {

"udfs": [

"UDPM-218"

]

}

},

{

"name": "Evalutate for SNP and Exome",

"uuid": "UDPM-687",

"input_type": "UDPM-127",

"output_type": "UDPM-82",

"description": "Disposition Task File",

"flags": 0,

"options": {

"obj_type": "SubjectType"

}

}

],

"next_states": [

{

"state_name": "Collaborate Out",

"state_uuid": "UDPM-1055",

"btn_text": "External Collaboration",

"btn_css": "rgb(13, 73, 123)",

"btn_scale": "large"

},

{

"state_name": "Internal Collaboration",

"state_uuid": "UDPM-1056",

"btn_text": "Internal Collaboration",

"btn_css": "rgb(13, 73, 123)",

"btn_scale": "large"

},

{

"state_name": "Case Closed",

"state_uuid": "UDPM-470",

"btn_text": "Close Case",

"btn_css": "#456",

"after_code": "subj.set_value('Rationale/Reasoning', params['Rationale/Reasoning'] )",

"after_code_params": [

"UDPM-883"

]

}

],

"show_options": {

"top": 84,

"left": 52

}

},

{

"name": "Collaborate Out",

"uuid": "UDPM-1055",

"owner": "UDPM-67",

"managers": "UDPM-9",

"performers": "UDPM-49",

"tools": [

{

"name": "Return to Clinical Decision",

"uuid": "UDPM-1543",

"input_type": "UDPM-127",

"output_type": "UDPM-127",

"flags": 6,

"after_code": "advance_workflow(\"Patient Research Meeting \",\"Clinical Decision \",subj)\n"

},

{

"name": "Request New MTA/ITA",

"uuid": "UDPM-1365",

"input_type": "UDPM-127",

"output_type": "UDPM-204",

"flags": 0,

"options": {

"obj_type": "SubjectType"

}

},

{

"name": "Request Material Transfer",

"uuid": "UDPM-1366",

"input_type": "UDPM-127",

"output_type": "UDPM-147",

"description": "Existing MTA in place",

"flags": 0,

"options": {

"obj_type": "SubjectType"

}

},

{

"name": "Request Information Transfer",

"uuid": "UDPM-1367",

"input_type": "UDPM-127",

"output_type": "UDPM-183",

"description": "Existing ITA in place",

"flags": 0,

"options": {

"obj_type": "SubjectType"

}

},

{

"name": "Justification for Collaboration",

"uuid": "UDPM-1371",

"input_type": "UDPM-127",

"output_type": "UDPM-127",

"flags": 1,

"options": {

"udfs": [

"UDPM-2130",

"UDPM-2773"

]

}

}

],

"show_options": {

"top": 427,

"left": 157

},

"end_task": true

},

{

"name": "Internal Collaboration",

"uuid": "UDPM-1056",

"owner": "UDPM-67",

"managers": "UDPM-9",

"performers": "UDPM-49",

"tools": [

{

"name": "Return to Clinical Decision",

"uuid": "UDPM-1542",

"input_type": "UDPM-127",

"output_type": "UDPM-127",

"flags": 6,

"after_code": "advance_workflow(\"Patient Research Meeting \",\"Clinical Decision \",subj)\n"

},

{

"name": "Initiate New Research Project",

"uuid": "UDPM-1364",

"input_type": "UDPM-127",

"output_type": "UDPM-218",

"flags": 0,

"options": {

"obj_type": "SubjectType"

}

},

{

"name": "Request Zebrafish Model",

"uuid": "UDPM-1369",

"input_type": "UDPM-127",

"output_type": "UDPM-186",

"flags": 0,

"options": {

"obj_type": "SubjectType"

}

},

{

"name": "Justification for Research",

"uuid": "UDPM-1370",

"input_type": "UDPM-127",

"output_type": "UDPM-127",

"flags": 1,

"options": {

"udfs": [

"UDPM-2130",

"UDPM-2773"

]

}

}

],

"show_options": {

"top": 282,

"left": 541

},

"end_task": true

}

],

"entry_point": "UDPM-469",

"show_udfs": "UDPM-5,UDPM-218"

},{

"name": "NIH Visit - DELETE",

"uuid": "UDPM-124",

"subject_type": "UDPM-140",

"state_defs": [

{

"name": "Consultation Complete",

"uuid": "UDPM-564",

"owner": "UDPM-67",

"managers": "UDPM-9",

"performers": "UDPM-2",

"duration": 14.0,

"next_states": [

{

"state_name": "NIH Visit",

"state_uuid": "UDPM-558",

"btn_text": "Return to NIH Visit",

"btn_css": "#456",

"before_code": "# Script to run Before Transition is executed\nparams[:ask_next_performer] = true"

},

{

"state_name": "NIH Visit Complete",

"state_uuid": "UDPM-686",

"btn_text": "NIH Visit Complete",

"btn_css": "#456",

"before_code": "# Script to run Before Transition is executed\nparams[:ask_next_performer] = true"

}

],

"show_options": {

"top": 519,

"left": 537

}

},

{

"name": "Message Follow Up Task",

"uuid": "UDPM-561",

"owner": "UDPM-67",

"managers": "UDPM-9",

"performers": "UDPM-2",

"duration": 10.0,

"tools": [

{

"name": "New Visit",

"uuid": "UDPM-635",

"input_type": "UDPM-140",

"output_type": "UDPM-139",

"description": "Schedule new visit",

"flags": 0,

"options": {

"obj_type": "SubjectType"

}

},

{

"name": "Send NP/PA/Attending note for Urgent Matter",

"uuid": "UDPM-636",

"input_type": "UDPM-140",

"output_type": "UDPM-140",

"description": "Page if Emergency",

"flags": 1,

"options": {

"udfs": [

"UDPM-6",

"UDPM-191"

]

}

},

{

"name": "Message Follow up Task",

"uuid": "UDPM-639",

"input_type": "UDPM-140",

"output_type": "UDPM-140",

"flags": 1,

"options": {

"udfs": [

"UDPM-1268",

"UDPM-1269",

"UDPM-1270",

"UDPM-1271",

"UDPM-1266",

"UDPM-1272"

]

}

}

],

"next_states": [

{

"state_name": "NIH Visit",

"state_uuid": "UDPM-558",

"btn_text": "Return to NIH Visit",

"btn_css": "#456",

"before_code": "# Script to run Before Transition is executed\nparams[:ask_next_performer] = true"

},

{

"state_name": "To Do",

"state_uuid": "UDPM-562",

"btn_text": "Create To Do",

"btn_css": "#456",

"after_code": "# Extra code here\n",

"before_code": "# Script to run Before Transition is executed\nparams[:ask_next_performer] = true"

}

],

"show_options": {

"top": 516,

"left": 255

}

},

{

"name": "NIH Visit",

"uuid": "UDPM-558",

"owner": "UDPM-67",

"managers": "UDPM-9",

"performers": "UDPM-2",

"duration": 10.0,

"tools": [

{

"name": "NIH Visit ",

"uuid": "UDPM-637",

"input_type": "UDPM-140",

"output_type": "UDPM-140",

"description": "Checklist",

"flags": 1,

"options": {

"udfs": [

"UDPM-6",

"UDPM-1502",

"UDPM-249",

"UDPM-1110",

"UDPM-1249",

"UDPM-1250",

"UDPM-1251",

"UDPM-1252",

"UDPM-1273"

]

}

},

{

"name": "Patient Research Meeting",

"uuid": "UDPM-686",

"input_type": "UDPM-140",

"output_type": "UDPM-127",

"flags": 0,

"options": {

"obj_type": "SubjectType"

}

},

{

"name": "Consult Request",

"uuid": "UDPM-923",

"input_type": "UDPM-140",

"output_type": "UDPM-180",

"flags": 0,

"options": {

"obj_type": "SubjectType"

}

}

],

"next_states": [

{

"state_name": "NIH Visit Complete",

"state_uuid": "UDPM-686",

"btn_text": "NIH Visit Complete",

"btn_css": "#456",

"after_code": "# Script to run After Transition is executed\npatient = subj.get_value('Patient')\npatient.set_value('Race', params['Race'] )\npatient.set_value('Ethnicity', params['Ethnicity'] )",

"before_code": "# Script to run Before Transition is executed\npatient = subj.get_value('Patient')\nif patient \n \tRace = patient.get_value('Race')\n Ethnicity = patient.get_value('Ethnicity')\n Attending = patient.get_value('Attending Physician')\n if not Race and not Ethnicity\n msg = \"Race and Ethicity information are not found in the patient data. Both are required for Exome analysis and dbGAP submission. Please try to enter them. <br> You may advance this process to the next state however Friday Meeting Notes will not advance without Race data <br> <b> Attending Physician: #{Attending}</b>\"\n params[:tool_message] = msg \n elsif Race and not Ethnicity\n msg = \"<b>Patient race : #{Race}</b> <br> Ethicity information is not found in the patient data. Ethnicity is required for Exome analysis and dbGAP submission. Please try to enter it. <br> You may advance this process to the next state however Friday Meeting Notes will not advance without Ethnicity data <br> <b> Attending Physician: #{Attending}</b>\"\n params[:tool_message] = msg \n params[:skip_udf] = {\n 'Race' =>true\n }\n elsif Ethnicity and not Race\n msg = \"<b>Patient Ethnicity : #{Ethnicity}</b> <br>Race information is not found in the patient data. Race is required for Exome analysis and dbGAP submission. Please try to enter it. <br> You may advance this process to the next state however Friday Meeting Notes will not advance without Race data <br> <b> Attending Physician: #{Attending}</b>\"\n params[:tool_message] = msg \n params[:skip_udf] = {\n 'Ethnicity' =>true\n }\n elsif Race and Ethnicity\n params[:skip_udf] = {\n 'Ethnicity' =>true,\n 'Race'=>true\n }\n end\nend\n",

"after_code_params": [

"UDPM-2202",

"UDPM-562"

]

},

{

"state_name": "Send Out LabTracking",

"state_uuid": "UDPM-560",

"btn_text": "Send Out Lab",

"btn_css": "#456",

"after_code": "# Extra code here\n",

"before_code": "# Script to run Before Transition is executed\nparams[:ask_next_performer] = true"

},

{

"state_name": "Seen",

"state_uuid": "UDPM-559",

"btn_text": "Clinical Follow up",

"btn_css": "#456",

"after_code": "# Extra code here\npatient = subj.get_value('Patient')\npatient.set_value('Race', params['Race'] )\npatient.set_value('Ethnicity', params['Ethnicity'] )\n\n",

"before_code": "# Script to run Before Transition is executed\npatient = subj.get_value('Patient')\nif patient \n Race = patient.get_value('Race')\n Ethnicity = patient.get_value('Ethnicity')\n Attending = patient.get_value('Attending Physician')\n if not Race and not Ethnicity\n msg = \"Race and Ethicity information are not found in the patient data. Both are required for Exome analysis and dbGAP submission. Please try to enter them. <br> You may advance this process to the next state however Friday Meeting Notes will not advance without Race data <br> <b> Attending Physician: #{Attending}</b>\"\n params[:tool_message] = msg \n elsif Race and not Ethnicity\n msg = \"<b>Patient race : #{Race}</b> <br> Ethicity information is not found in the patient data. Ethnicity is required for Exome analysis and dbGAP submission. Please try to enter it. <br> You may advance this process to the next state however Friday Meeting Notes will not advance without Ethnicity data <br> <b> Attending Physician: #{Attending}</b>\"\n params[:tool_message] = msg \n params[:skip_udf] = {\n 'Race' =>true\n }\n elsif Ethnicity and not Race\n msg = \"<b>Patient Ethnicity : #{Ethnicity}</b> <br>Race information is not found in the patient data. Race is required for Exome analysis and dbGAP submission. Please try to enter it. <br> You may advance this process to the next state however Friday Meeting Notes will not advance without Race data <br> <b> Attending Physician: #{Attending}</b>\"\n params[:tool_message] = msg \n params[:skip_udf] = {\n 'Ethnicity' =>true\n }\n elsif Race and Ethnicity\n params[:skip_udf] = {\n 'Ethnicity' =>true,\n 'Race'=>true\n }\n end\nend\nparams[:ask_next_performer] = true",

"after_code_params": [

"UDPM-2202",

"UDPM-562"

]

},

{

"state_name": "To Do",

"state_uuid": "UDPM-562",

"btn_text": "Create To Do",

"btn_css": "#456",

"after_code": "# Extra code here\n",

"before_code": "# Script to run Before Transition is executed\nparams[:ask_next_performer] = true"

}

],

"show_options": {

"top": 72,

"left": 210

}

},

{

"name": "NIH Visit Complete",

"uuid": "UDPM-686",

"owner": "UDPM-67",

"managers": "UDPM-9",

"performers": "UDPM-2",

"duration": 30.0,

"tools": [

{

"name": "Update Patient",

"uuid": "UDPM-1121",

"input_type": "UDPM-140",

"output_type": "UDPM-140",

"flags": 1,

"options": {

"udfs": [

"UDPM-545",

"UDPM-1252",

"UDPM-1273",

"UDPM-2314",

"UDPM-2313"

]

}

}

],

"em_owner_done_job": true,

"show_options": {

"top": 399,

"left": 888

},

"end_task": true

},

{

"name": "Seen",

"uuid": "UDPM-559",

"owner": "UDPM-67",

"managers": "UDPM-9",

"performers": "UDPM-2",

"duration": 30.0,

"tools": [

{

"name": "New Visit",

"uuid": "UDPM-633",

"input_type": "UDPM-140",

"output_type": "UDPM-139",

"description": "Schedule new visit",

"flags": 0,

"options": {

"obj_type": "SubjectType"

}

}

],

"next_states": [

{

"state_name": "To Do",

"state_uuid": "UDPM-562",

"btn_text": "Create To Do",

"btn_css": "#456",

"before_code": "# Script to run Before Transition is executed\nparams[:ask_next_performer] = true"

},

{

"state_name": "NIH Visit Complete",

"state_uuid": "UDPM-686",

"btn_text": "NIH Visit Complete",

"btn_css": "#456",

"after_code": "# Extra code here\nshow_message(\"This will mark the NIH visit complete, you may not be able to take this action back. Are you sure you want to advance the patient to this state?\")"

},

{

"state_name": "NIH Visit",

"state_uuid": "UDPM-558",

"btn_text": "Return to NIH Visit",

"btn_css": "#456",

"before_code": "# Script to run Before Transition is executed\nparams[:ask_next_performer] = true"

},

{

"state_name": "Send Out LabTracking",

"state_uuid": "UDPM-560",

"btn_text": "Send Out Lab",

"btn_css": "#456",

"after_code": "# Extra code here\n",

"before_code": "# Script to run Before Transition is executed\nparams[:ask_next_performer] = true"

},

{

"state_name": "Message Follow Up Task",

"state_uuid": "UDPM-561",

"btn_text": "Message Follow up",

"btn_css": "#456",

"after_code": "# Extra code here\n",

"before_code": "# Script to run Before Transition is executed\nparams[:ask_next_performer] = true"

}

],

"show_options": {

"top": 225,

"left": 1139

}

},

{

"name": "Send Out LabTracking",

"uuid": "UDPM-560",

"owner": "UDPM-67",

"managers": "UDPM-9",

"performers": "UDPM-5",

"duration": 30.0,

"tools": [

{

"name": "Send Out Lab",

"uuid": "UDPM-638",

"input_type": "UDPM-140",

"output_type": "UDPM-140",

"description": "Tracking",

"flags": 1,

"options": {

"udfs": [

"UDPM-6",

"UDPM-1120",

"UDPM-1253",

"UDPM-1254",

"UDPM-1255",

"UDPM-1256",

"UDPM-121",

"UDPM-1257",

"UDPM-1258",

"UDPM-1259",

"UDPM-1260",

"UDPM-1261",

"UDPM-1262",

"UDPM-573",

"UDPM-1263",

"UDPM-191",

"UDPM-1264",

"UDPM-1265",

"UDPM-1266",

"UDPM-1267"

]

}

}

],

"next_states": [

{

"state_name": "NIH Visit",

"state_uuid": "UDPM-558",

"btn_text": "Return to NIH Visit",

"btn_css": "#456",

"before_code": "# Script to run Before Transition is executed\nparams[:ask_next_performer] = true"

},

{

"state_name": "Message Follow Up Task",

"state_uuid": "UDPM-561",

"btn_text": "Message Follow up",

"btn_css": "#456",

"after_code": "# Extra code here\n",

"before_code": "# Script to run Before Transition is executed\nparams[:ask_next_performer] = true"

},

{

"state_name": "Consultation Complete",

"state_uuid": "UDPM-564",

"btn_text": "Complete",

"btn_css": "#456",

"after_code": "# Extra code here\ns",

"before_code": "# Script to run Before Transition is executed\nparams[:ask_next_performer] = true"

}

],

"show_options": {

"top": 323,

"left": 15

}

},

{

"name": "To Do",

"uuid": "UDPM-562",

"owner": "UDPM-67",

"managers": "UDPM-9",

"performers": "UDPM-20",

"duration": 10.0,

"tools": [

{

"name": "PIFUT",

"uuid": "UDPM-641",

"input_type": "UDPM-140",

"output_type": "UDPM-140",

"description": "Patient Issue Follow Up Task",

"flags": 1,

"options": {

"udfs": [

"UDPM-6",

"UDPM-1120",

"UDPM-48",

"UDPM-1235",

"UDPM-1236",

"UDPM-1237"

]

}

}

],

"next_states": [

{

"state_name": "NIH Visit",

"state_uuid": "UDPM-558",

"btn_text": "Return to NIH Visit",

"btn_css": "#456",

"before_code": "# Script to run Before Transition is executed\nparams[:ask_next_performer] = true"

}

],

"show_options": {

"top": 27,

"left": 681.5125122070312

}

}

],

"entry_point": "UDPM-558",

"ask_performer": true,

"show_udfs": "UDPM-545"

},{

"name": "Zebrafish Mutation Project Workflow",

"uuid": "UDPM-156",

"subject_type": "UDPM-186",

"state_defs": [

{

"name": "Case Closed",

"uuid": "UDPM-1099",

"owner": "UDPM-96",

"managers": "UDPM-36",

"performers": "UDPM-33",

"show_options": {

"top": 430.9658660888672,

"left": 1130.9829406738281

},

"end_task": true

},

{

"name": "CRISPR/Cas9 Design and Synthesis",

"uuid": "UDPM-730",

"owner": "UDPM-67",

"managers": "UDPM-36",

"performers": "UDPM-33",

"tools": [

{

"name": "1) CRISPR and Primer Design",

"uuid": "UDPM-1103",

"input_type": "UDPM-186",

"output_type": "UDPM-186",

"flags": 1,

"options": {

"udfs": [

"UDPM-1969",

"UDPM-2424",

"UDPM-2230",

"UDPM-2232",

"UDPM-2423",

"UDPM-2738"

]

}

},

{

"name": "3) RNA Synthesis",

"uuid": "UDPM-1144",

"input_type": "UDPM-186",

"output_type": "UDPM-186",

"description": "CRISPR and cas9",

"flags": 1,

"options": {

"udfs": [

"UDPM-2397",

"UDPM-2468",

"UDPM-2400",

"UDPM-2248",

"UDPM-2467",

"UDPM-2738"

]

}

},

{

"name": "2) Request Primer Order",

"uuid": "UDPM-1163",

"input_type": "UDPM-186",

"output_type": "UDPM-217",

"flags": 0,

"options": {

"obj_type": "SubjectType"

}

}

],

"next_states": [

{

"state_name": "Microinjection of zebrafish embryos",

"state_uuid": "UDPM-768",

"btn_text": "4) Begin Injection of Embryos",

"btn_css": "#456",

"after_code": "self.next_state_performer = User.curr_user"

}

],

"show_options": {

"top": 16.083328247070312,

"left": 494

}

},

{

"name": "Culture Healthy F0 Embryos",

"uuid": "UDPM-887",

"owner": "UDPM-67",

"managers": "UDPM-36",

"performers": "UDPM-33",

"duration": 90.0,

"tools": [

{

"name": "F0 Generation",

"uuid": "UDPM-1217",

"input_type": "UDPM-186",

"output_type": "UDPM-234",

"description": "Culture information",

"flags": 0,

"options": {

"obj_type": "SubjectType",

"do_not_open_subject": true

}

},

{

"name": "F0 Survival Information",

"uuid": "UDPM-1142",

"input_type": "UDPM-186",

"output_type": "UDPM-186",

"flags": 1,

"hide_button_if_not_condition": true,

"options": {

"udfs": [

"UDPM-2427"

]

}

}

],

"next_states": [

{

"state_name": "F0 Breeding",

"state_uuid": "UDPM-772",

"btn_text": "Begin F0 Breeding",

"btn_css": "#456",

"after_code": "self.next_state_performer = User.curr_user"

}

],

"show_options": {

"top": 254.57102966308594,

"left": 184.82101440429688

}

},

{

"name": "F0 Breeding",

"uuid": "UDPM-772",

"owner": "UDPM-67",

"managers": "UDPM-36",

"performers": "UDPM-33",

"tools": [

{

"name": "Perform In Situ Hybridization",

"uuid": "UDPM-1310",

"input_type": "UDPM-186",

"output_type": "UDPM-252",

"flags": 0,

"options": {

"obj_type": "SubjectType"

}

},

{

"name": "F0 Breeding Information",

"uuid": "UDPM-1215",

"input_type": "UDPM-186",

"output_type": "UDPM-233",

"flags": 0,

"options": {

"obj_type": "SubjectType",

"do_not_open_subject": true

}

}

],

"next_states": [

{

"state_name": "F1 Generation Culture",

"state_uuid": "UDPM-964",

"btn_text": "Grow F1 Generation",

"btn_css": "#456",

"after_code": "self.next_state_performer = User.curr_user"

}

],

"show_options": {

"top": 360.14488220214844,

"left": 33.8238525390625

}

},

{

"name": "F1 Breeding",

"uuid": "UDPM-889",

"owner": "UDPM-67",

"managers": "UDPM-36",

"performers": "UDPM-33",

"tools": [

{

"name": "Breeding Information",

"uuid": "UDPM-1146",

"input_type": "UDPM-186",

"output_type": "UDPM-186",

"description": "EZColony Integration needed",

"flags": 1,

"options": {

"udfs": [

"UDPM-2405",

"UDPM-2435"

]

}

},

{

"name": "Perform In Situ Hybridization",

"uuid": "UDPM-1309",

"input_type": "UDPM-186",

"output_type": "UDPM-252",

"flags": 0,

"options": {

"obj_type": "SubjectType"

}

}

],

"next_states": [

{

"state_name": "F2 Phenotyping",

"state_uuid": "UDPM-1100",

"btn_text": "Phenotype F2 Generation",

"btn_css": "rgb(13, 73, 123)",

"btn_scale": "large"

},

{

"state_name": "Case Closed",

"state_uuid": "UDPM-1099",

"btn_text": "Close Case",

"btn_css": "rgb(13, 73, 123)",

"btn_scale": "large",

"btn_tooltip": "No obvious phenotype detected"

}

],

"show_options": {

"top": 397.9971466064453,

"left": 862.9914855957031

}

},

{

"name": "F1 Generation Culture",

"uuid": "UDPM-964",

"owner": "UDPM-67",

"managers": "UDPM-36",

"performers": "UDPM-33",

"duration": 90.0,

"tools": [

{

"name": "Perform In situ Hybridization",

"uuid": "UDPM-1307",

"input_type": "UDPM-186",

"output_type": "UDPM-252",

"flags": 0,

"options": {

"obj_type": "SubjectType"

}

},

{

"name": "F1 Generation",

"uuid": "UDPM-1218",

"input_type": "UDPM-186",

"output_type": "UDPM-234",

"flags": 0,

"options": {

"obj_type": "SubjectType",

"do_not_open_subject": true

}

}

],

"next_states": [

{

"state_name": "F1 Generation Genotyping",

"state_uuid": "UDPM-888",

"btn_text": "Genotype F1 Generation",

"btn_css": "#456",

"after_code": "self.next_state_performer = User.curr_user"

}

],

"show_options": {

"top": 446.99998474121094,

"left": 216.99429321289062

}

},

{

"name": "F1 Generation Genotyping",

"uuid": "UDPM-888",

"owner": "UDPM-67",

"managers": "UDPM-36",

"performers": "UDPM-33",

"tools": [

{

"name": "Perform In Situ Hybridization",

"uuid": "UDPM-1308",

"input_type": "UDPM-186",

"output_type": "UDPM-252",

"flags": 0,

"options": {

"obj_type": "SubjectType"

}

},

{

"name": "Genotyping Information",

"uuid": "UDPM-1145",

"input_type": "UDPM-186",

"output_type": "UDPM-186",

"flags": 1,

"options": {

"udfs": [

"UDPM-2111",

"UDPM-2100",

"UDPM-2436",

"UDPM-2742"

]

}

}

],

"next_states": [

{

"state_name": "F1 Breeding",

"state_uuid": "UDPM-889",

"btn_text": "Begin F1 Breeding",

"btn_css": "#456",

"after_code": "self.next_state_performer = User.curr_user"

}

],

"show_options": {

"top": 429.98863220214844,

"left": 541.9971008300781

}

},

{

"name": "F2 Phenotyping",

"uuid": "UDPM-1100",

"owner": "UDPM-96",

"managers": "UDPM-36",

"performers": "UDPM-33",

"tools": [

{

"name": "Phenotype Information",

"uuid": "UDPM-1504",

"input_type": "UDPM-186",

"output_type": "UDPM-173",

"flags": 0,

"options": {

"obj_type": "SubjectType"

}

}

],

"next_states": [

{

"state_name": "Case Closed",

"state_uuid": "UDPM-1099",

"btn_text": "Close Case",

"btn_css": "rgb(13, 73, 123)",

"btn_scale": "large"

}

],

"show_options": {

"top": 274.9658966064453,

"left": 986.9516906738281

}

},

{

"name": "Microinjection of zebrafish embryos",

"uuid": "UDPM-768",

"owner": "UDPM-67",

"managers": "UDPM-36",

"performers": "UDPM-33",

"duration": 20.0,

"tools": [

{

"name": "Injection Information",

"uuid": "UDPM-1214",

"input_type": "UDPM-186",

"output_type": "UDPM-232",

"flags": 0,

"options": {

"obj_type": "SubjectType",

"do_not_open_subject": true

}

}

],

"next_states": [

{

"state_name": "T7 Endonuclease Assay",

"state_uuid": "UDPM-775",

"btn_text": "Perform T7 endonuclease assay",

"btn_css": "#456",

"after_code": "self.next_state_performer = User.curr_user"

},

{

"state_name": "Culture Healthy F0 Embryos",

"state_uuid": "UDPM-887",

"btn_text": "Culture Healthy Embryos",

"btn_css": "#456",

"after_code": "self.next_state_performer = User.curr_user"

}

],

"show_options": {

"top": 131,

"left": 564.25

}

},

{

"name": "Request for Zebrafish Mutation Project",

"uuid": "UDPM-886",

"owner": "UDPM-67",

"managers": "UDPM-36",

"performers": "UDPM-33",

"tools": [

{

"name": "Model Information",

"uuid": "UDPM-1141",

"input_type": "UDPM-186",

"output_type": "UDPM-186",

"flags": 1,

"options": {

"udfs": [

"UDPM-2032",

"UDPM-2130",

"UDPM-886",

"UDPM-1873",

"UDPM-2393",

"UDPM-2194"

]

}

}

],

"next_states": [

{

"state_name": "CRISPR/Cas9 Design and Synthesis",

"state_uuid": "UDPM-730",

"btn_text": "Design CRISPRs",

"btn_css": "#456",

"after_code": "self.next_state_performer = User.curr_user"

}

],

"show_options": {

"top": 100.99432373046875,

"left": 67.98861694335938

}

},

{

"name": "T7 Endonuclease Assay",

"uuid": "UDPM-775",

"owner": "UDPM-67",

"managers": "UDPM-36",

"performers": "UDPM-33",

"tools": [

{

"name": "Assay Information",

"uuid": "UDPM-994",

"input_type": "UDPM-186",

"output_type": "UDPM-186",

"flags": 1,

"options": {

"udfs": [

"UDPM-2403",

"UDPM-2077",

"UDPM-2425",

"UDPM-1347",

"UDPM-2244",

"UDPM-2079",

"UDPM-2341",

"UDPM-2740"

]

}

}

],

"next_states": [

{

"state_name": "Culture Healthy F0 Embryos",

"state_uuid": "UDPM-887",

"btn_text": "Culture Healthy Embryos",

"btn_css": "#456",

"after_code": "self.next_state_performer = User.curr_user"

}

],

"show_options": {

"top": 284.41668701171875,

"left": 575.6666259765625

}

}

],

"entry_point": "UDPM-886",

"ask_performer": true,

"show_udfs": "UDPM-2420,UDPM-2393,UDPM-2421,UDPM-2422,UDPM-1474,UDPM-2032,UDPM-2130,UDPM-2194"

},{

"name": "Medications",

"uuid": "UDPM-136",

"subject_type": "UDPM-142",

"state_defs": [

{

"name": "Medication",

"uuid": "UDPM-627",

"owner": "UDPM-67",

"managers": "UDPM-2",

"performers": "UDPM-18",

"duration": 5.0,

"tools": [

{

"name": "Edit Medication Information",

"uuid": "UDPM-778",

"input_type": "UDPM-142",

"output_type": "UDPM-142",

"flags": 1,

"options": {

"udfs": [

"UDPM-6",

"UDPM-1336",

"UDPM-1106",

"UDPM-1107",

"UDPM-1556"

]

}

}

],

"show_options": {

"top": 135,

"left": 103

},

"end_task": true

}

],

"entry_point": "UDPM-627",

"show_udfs": "UDPM-2151,UDPM-1106,UDPM-1107,UDPM-1336,UDPM-2150,UDPM-1556"

},{

"name": "Patient Visit Scheduling Tools - DELETE",

"uuid": "UDPM-138",

"subject_type": "UDPM-163",

"state_defs": [

{

"name": "Accepted",

"uuid": "UDPM-630",

"owner": "UDPM-67",

"managers": "UDPM-9",

"performers": "UDPM-20",

"duration": 10.0,

"next_states": [

{

"state_name": "Scheduling Imminent",

"state_uuid": "UDPM-631",

"btn_text": "Schedule - Imminent",

"btn_css": "#456",

"after_code": "self.next_state_performer = User.curr_user"

},

{

"state_name": "Scheduling - Far Out",

"state_uuid": "UDPM-632",

"btn_text": "Schedule - Far Out",

"btn_css": "#456",

"after_code": "self.next_state_performer = User.curr_user"

},

{

"state_name": "Scheduling - Near",

"state_uuid": "UDPM-635",

"btn_text": "Schedule - Near",

"btn_css": "#456",

"after_code": "self.next_state_performer = User.curr_user"

}

],

"show_options": {

"top": 30,

"left": 41

}

},

{

"name": "Assessment Tools",

"uuid": "UDPM-634",

"owner": "UDPM-67",

"managers": "UDPM-9",

"performers": "UDPM-20",

"duration": 30.0,

"tools": [

{

"name": "1 - Cardiovascular",

"uuid": "UDPM-788",

"input_type": "UDPM-163",

"output_type": "UDPM-163",

"flags": 1,

"options": {

"udfs": [

"UDPM-6",

"UDPM-1502",

"UDPM-1195",

"UDPM-1225",

"UDPM-1192",

"UDPM-1193",

"UDPM-1194",

"UDPM-1543",

"UDPM-1375"

]

}

},

{

"name": "7 - Consultation Request",

"uuid": "UDPM-789",

"input_type": "UDPM-163",

"output_type": "UDPM-163",

"flags": 1,

"options": {

"udfs": [

"UDPM-6",

"UDPM-1502",

"UDPM-1225",

"UDPM-945",

"UDPM-1229",

"UDPM-1230",

"UDPM-1231",

"UDPM-1232",

"UDPM-1233",

"UDPM-1234",

"UDPM-1117",

"UDPM-1549",

"UDPM-1248"

]

}

},

{

"name": "8 - Create PIFUT",

"uuid": "UDPM-790",

"input_type": "UDPM-163",

"output_type": "UDPM-164",

"flags": 0,

"options": {

"obj_type": "SubjectType"

}

},

{

"name": "2 - Neurological",

"uuid": "UDPM-791",

"input_type": "UDPM-163",

"output_type": "UDPM-163",

"flags": 1,

"options": {

"udfs": [

"UDPM-6",

"UDPM-1502",

"UDPM-1186",

"UDPM-1225",

"UDPM-1136",

"UDPM-1180",

"UDPM-1179",

"UDPM-1219",

"UDPM-1181",

"UDPM-1183",

"UDPM-1185",

"UDPM-1544",

"UDPM-1375"

]

}

},

{

"name": "3 - Nutrition",

"uuid": "UDPM-792",

"input_type": "UDPM-163",

"output_type": "UDPM-163",

"flags": 1,

"options": {

"udfs": [

"UDPM-6",

"UDPM-1502",

"UDPM-1191",

"UDPM-1225",

"UDPM-1187",

"UDPM-1194",

"UDPM-1188",

"UDPM-1190",

"UDPM-1545",

"UDPM-1248"

]

}

},

{

"name": "4 - Rehab",

"uuid": "UDPM-793",

"input_type": "UDPM-163",

"output_type": "UDPM-163",

"flags": 1,

"options": {

"udfs": [

"UDPM-6",

"UDPM-1502",

"UDPM-1633",

"UDPM-1225",

"UDPM-1196",

"UDPM-1220",

"UDPM-1198",

"UDPM-1653",

"UDPM-1548",

"UDPM-1248"

]

}

},

{

"name": "5 - Respiratory",

"uuid": "UDPM-794",

"input_type": "UDPM-163",

"output_type": "UDPM-163",

"flags": 1,

"options": {

"udfs": [

"UDPM-6",

"UDPM-1502",

"UDPM-1178",

"UDPM-1225",

"UDPM-1133",

"UDPM-1132",

"UDPM-1134",

"UDPM-1135",

"UDPM-1138",

"UDPM-1547",

"UDPM-1375"

]

}

},

{

"name": "6 - Safety & Equip.",

"uuid": "UDPM-795",

"input_type": "UDPM-163",

"output_type": "UDPM-163",

"flags": 1,

"options": {

"udfs": [

"UDPM-6",

"UDPM-1502",

"UDPM-1551",

"UDPM-1225",

"UDPM-1201",

"UDPM-1198",

"UDPM-1199",

"UDPM-1546",

"UDPM-1375"

]

}

}

],

"next_states": [

{

"state_name": "Scheduling - Far Out",

"state_uuid": "UDPM-632",

"btn_text": "Schedule - Far Out",

"btn_css": "#456",

"after_code": "self.next_state_performer = User.curr_user"

},

{

"state_name": "Scheduling Imminent",

"state_uuid": "UDPM-631",

"btn_text": "Schedule - Imminent",

"btn_css": "#456",

"after_code": "self.next_state_performer = User.curr_user"

},

{

"state_name": "Scheduling - Near",

"state_uuid": "UDPM-635",

"btn_text": "Schedule - Near",

"btn_css": "#456",

"after_code": "self.next_state_performer = User.curr_user"

},

{

"state_name": "Complete",

"state_uuid": "UDPM-633",

"btn_text": "Complete",

"btn_css": "#456",

"after_code": "self.next_state_performer = User.curr_user"

}

],

"show_options": {

"top": 294,

"left": 442.816650390625

}

},

{

"name": "Complete",

"uuid": "UDPM-633",

"owner": "UDPM-67",

"managers": "UDPM-9",

"performers": "UDPM-20",

"duration": 5.0,

"tools": [

{

"name": "NIH Visit",

"uuid": "UDPM-787",

"input_type": "UDPM-163",

"output_type": "UDPM-140",

"flags": 0,

"options": {

"obj_type": "SubjectType"

}

}

],

"show_options": {

"top": 101,

"left": 838.2666015625

},

"end_task": true

},

{

"name": "Scheduling - Far Out",

"uuid": "UDPM-632",

"owner": "UDPM-67",

"managers": "UDPM-9",

"performers": "UDPM-20",

"duration": 30.0,

"tools": [

{

"name": "Checklist",

"uuid": "UDPM-783",

"input_type": "UDPM-163",

"output_type": "UDPM-163",

"flags": 1,

"options": {

"udfs": [

"UDPM-6",

"UDPM-1561",

"UDPM-1562",

"UDPM-1613",

"UDPM-1614",

"UDPM-1632",

"UDPM-1630",

"UDPM-1368",

"UDPM-1584",

"UDPM-1563",

"UDPM-1155",

"UDPM-1162",

"UDPM-1163",

"UDPM-1242",

"UDPM-1143",

"UDPM-945",

"UDPM-1126",

"UDPM-1360",

"UDPM-1159",

"UDPM-1358",

"UDPM-1359",

"UDPM-1361",

"UDPM-1160",

"UDPM-1364",

"UDPM-1362",

"UDPM-1366",

"UDPM-1367",

"UDPM-1369",

"UDPM-1370",

"UDPM-1371",

"UDPM-1372",

"UDPM-1157",

"UDPM-1130",

"UDPM-1111",

"UDPM-1153",

"UDPM-1161",

"UDPM-1148",

"UDPM-1373"

]

}

},

{

"name": "Create ATV",

"uuid": "UDPM-784",

"input_type": "UDPM-163",

"output_type": "UDPM-146",

"flags": 0,

"options": {

"obj_type": "SubjectType"

}

},

{

"name": "Create Send/Acceptance Letter",

"uuid": "UDPM-785",

"input_type": "UDPM-163",

"output_type": "UDPM-137",

"flags": 0,

"options": {

"obj_type": "SubjectType"

}

},

{

"name": "PIFUT",

"uuid": "UDPM-786",

"input_type": "UDPM-163",

"output_type": "UDPM-164",

"flags": 0,

"options": {

"obj_type": "SubjectType"

}

}

],

"next_states": [

{

"state_name": "Scheduling Imminent",

"state_uuid": "UDPM-631",

"btn_text": "Schedule - Imminent",

"btn_css": "#456",

"after_code": "self.next_state_performer = User.curr_user"

},

{

"state_name": "Complete",

"state_uuid": "UDPM-633",

"btn_text": "Complete",

"btn_css": "#456",

"after_code": "self.next_state_performer = User.curr_user"

},

{

"state_name": "Assessment Tools",

"state_uuid": "UDPM-634",

"btn_text": "Assess Tools",

"btn_css": "#456",

"after_code": "self.next_state_performer = User.curr_user"

},

{

"state_name": "Scheduling - Near",

"state_uuid": "UDPM-635",

"btn_text": "Schedule - Near",

"btn_css": "#456",

"after_code": "self.next_state_performer = User.curr_user"

}

],

"show_options": {

"top": 26,

"left": 522

}

},

{

"name": "Scheduling Imminent",

"uuid": "UDPM-631",

"owner": "UDPM-67",

"managers": "UDPM-9",

"performers": "UDPM-20",

"duration": 10.0,

"tools": [

{

"name": "Create PIFUT",

"uuid": "UDPM-782",

"input_type": "UDPM-163",

"output_type": "UDPM-164",

"flags": 0,

"options": {

"obj_type": "SubjectType"

}

},

{

"name": "Checklist",

"uuid": "UDPM-781",

"input_type": "UDPM-163",

"output_type": "UDPM-163",

"flags": 1,

"options": {

"udfs": [

"UDPM-6",

"UDPM-1502",

"UDPM-1238",

"UDPM-1239",

"UDPM-1240",

"UDPM-1241",

"UDPM-1242",

"UDPM-1243",

"UDPM-1244",

"UDPM-1245",

"UDPM-1246",

"UDPM-1247",

"UDPM-1617"

]

}

}

],

"next_states": [

{

"state_name": "Scheduling - Far Out",

"state_uuid": "UDPM-632",

"btn_text": "Schedule - Far Out",

"btn_css": "#456",

"after_code": "self.next_state_performer = User.curr_user"

},

{

"state_name": "Assessment Tools",

"state_uuid": "UDPM-634",

"btn_text": "Assess Tools",

"btn_css": "#456",

"after_code": "self.next_state_performer = User.curr_user"

},

{

"state_name": "Scheduling - Near",

"state_uuid": "UDPM-635",

"btn_text": "Schedule - Near",

"btn_css": "#456",

"after_code": "# Extra code hereself.next_state_performer = User.curr_user"

},

{

"state_name": "Complete",

"state_uuid": "UDPM-633",

"btn_text": "Complete",

"btn_css": "#456"

}

],

"show_options": {

"top": 392,

"left": 68.816650390625

}

},

{

"name": "Scheduling - Near",

"uuid": "UDPM-635",

"owner": "UDPM-67",

"managers": "UDPM-9",

"performers": "UDPM-20",

"duration": 10.0,

"tools": [

{

"name": "Create PIFUT",

"uuid": "UDPM-797",

"input_type": "UDPM-163",

"output_type": "UDPM-164",

"flags": 0,

"options": {

"obj_type": "SubjectType"

}

},

{

"name": "Checklist",

"uuid": "UDPM-796",

"input_type": "UDPM-163",

"output_type": "UDPM-163",

"flags": 1,

"options": {

"udfs": [

"UDPM-6",

"UDPM-1502",

"UDPM-1225",

"UDPM-1621",

"UDPM-1634",

"UDPM-1635",

"UDPM-1595",

"UDPM-1636",

"UDPM-1612",

"UDPM-1610",

"UDPM-1637",

"UDPM-1591",

"UDPM-1638",

"UDPM-1609",

"UDPM-1567",

"UDPM-1611",

"UDPM-1570",

"UDPM-1375"

]

}

}

],

"next_states": [

{

"state_name": "Complete",

"state_uuid": "UDPM-633",

"btn_text": "Complete",

"btn_css": "#456",

"after_code": "self.next_state_performer = User.curr_user"

},

{

"state_name": "Scheduling Imminent",

"state_uuid": "UDPM-631",

"btn_text": "Schedule - Imminent",

"btn_css": "#456",

"after_code": "self.next_state_performer = User.curr_user"

},

{

"state_name": "Assessment Tools",

"state_uuid": "UDPM-634",

"btn_text": "Assess Tools",

"btn_css": "#456",

"after_code": "self.next_state_performer = User.curr_user"

},

{

"state_name": "Scheduling - Far Out",

"state_uuid": "UDPM-632",

"btn_text": "Schedule - Far Out",

"btn_css": "#456",

"after_code": "self.next_state_performer = User.curr_user"

}

],

"show_options": {

"top": 461,

"left": 645.9000244140625

}

}

],

"entry_point": "UDPM-630",

"ask_performer": true

},{

"name": "DNA Extraction Workflow",

"uuid": "UDPM-101",

"subject_type": "UDPM-122",

"state_defs": [

{

"name": "Archive DNA",

"uuid": "UDPM-445",

"owner": "UDPM-67",

"managers": "UDPM-22",

"performers": "UDPM-56",

"show_options": {

"top": 460,

"left": 337.2166748046875

},

"end_task": true

},

{

"name": "Cancel",

"uuid": "UDPM-496",

"owner": "UDPM-67",

"managers": "UDPM-22",

"performers": "UDPM-69",

"show_options": {

"top": 281,

"left": 433

},

"end_task": true

},

{

"name": "DNA in Transit to Twinbrook",

"uuid": "UDPM-604",

"owner": "UDPM-67",

"managers": "UDPM-22",

"performers": "UDPM-22",

"duration": 1.0,

"next_states": [

{

"state_name": "Temporary Storage at Twinbrook",

"state_uuid": "UDPM-1060",

"btn_text": "Receive at Twinbrook",

"btn_css": "rgb(13, 73, 123)",

"btn_scale": "large"

}

],

"show_options": {

"top": 125,

"left": 932.2166748046875

}

},

{

"name": "Prepare 96 well plate",

"uuid": "UDPM-497",

"owner": "UDPM-67",

"managers": "UDPM-22",

"performers": "UDPM-56",

"tools": [

{

"name": "PicoGreen Data",

"uuid": "UDPM-984",

"input_type": "UDPM-122",

"output_type": "UDPM-122",

"flags": 1,

"options": {

"udfs": [

"UDPM-2030"

]

}

},

{

"name": "Submit to FreezerPro",

"uuid": "UDPM-685",

"input_type": "UDPM-122",

"output_type": "UDPM-122",

"description": "Submit 12 DNA into 96 plate",

"flags": 6,

"before_code": "msg = '1) Open <a target=\"_blank\" href=\"'+gen_freezerpro_url+'\">FreezerPro</a> and select an empty 96 plate<br>2) Verify VisionMate is working (Check Status)<br>3) Press Yes to continue'\nparams[:tool_message] = msg\nparams[:show_progress] = true",

"after_code": "h = find_subjects do |qb|\n qb.add_subject_type('LIMS Helper')\nend\n\nh = h[0]\nh = create_subject('LIMS Helper') unless h\nsamples = h.get_value('DNA Extractions for FreezerPro')\nraise \"Need to have 12 samples before submit. You have #{samples.length}.\" if samples.length<12-1\n\nsamples << subj\nbox_data = prescan_box_in_freezerpro\ncreate_data_in_freezerpro do |fp_data|\n samples.each_with_index do |ns, idx|\n ns.set_value('FreezerPro Storage Link', box_data[\"box\"][\"barcode_tag\"])\n patient = ns.get_value('Patient')\n\n sp = fp_data.add_sample( patient.name )\n ('A'..'H').each do |row|\n bcoderead = box_data[\"barcodes\"].find{|el| el[\"Row\"] == row && el[\"Col\"] == idx+1}\n v = sp.add_vial( bcoderead['tubeBarcode'] )\n v['position'] = bcoderead['position']\n \n vial = create_subject('Vial') do |lv|\n lv.set_value('Patient', patient)\n lv.set_value('Vial of a Sample', ns)\n lv.set_value('Volume (uL)', ns.get_value('Volume (uL)'))\n end\n end\n \n sp.set_value('OD 230', ns.get_value('OD 230') )\n sp.set_value('OD 260', ns.get_value('OD 260') )\n sp.set_value('OD 280', ns.get_value('OD 280') )\n end\n fp_data.box_id = box_data[\"box\"][\"id\"]\n fp_data.sample_type = 'DNA'\nend\n\nupdate_box_view_in_freezerpro(box_data[\"box\"][\"id\"])"

}

],

"next_states": [

{

"state_name": "Archive DNA",

"state_uuid": "UDPM-445",

"btn_text": "Complete Archive Process",

"btn_css": "#456",

"after_code": "h = find_subjects do |qb|\n qb.add_subject_type('LIMS Helper')\nend\n\nh = h[0]\nh = create_subject('LIMS Helper') unless h\n\nsamples = h.get_value('DNA Extractions for FreezerPro')\nraise \"Submit to FreezerPro first.\" if samples.length==12-1\nsamples << subj\nh.set_value('DNA Extractions for FreezerPro', samples)"

}

],

"show_options": {

"top": 479,

"left": 685

}

},

{

"name": "Quantification of DNA",

"uuid": "UDPM-575",

"owner": "UDPM-67",

"managers": "UDPM-22",

"performers": "UDPM-69",

"duration": 1.0,

"tools": [

{

"name": "1) Processing and Extraction",

"uuid": "UDPM-814",

"input_type": "UDPM-122",

"output_type": "UDPM-122",

"flags": 1,

"options": {

"udfs": [

"UDPM-1017",

"UDPM-83",

"UDPM-1298",

"UDPM-360",

"UDPM-450"

]

}

},

{

"name": "2) Quantification Information",

"uuid": "UDPM-661",

"input_type": "UDPM-122",

"output_type": "UDPM-122",

"flags": 1,

"options": {

"udfs": [

"UDPM-451",

"UDPM-1977",

"UDPM-1016",

"UDPM-627",

"UDPM-628",

"UDPM-452"

]

}

}

],

"next_states": [

{

"state_name": "Store DNA for Transport",

"state_uuid": "UDPM-593",

"btn_text": "3) Temporarily Store for Transport",

"btn_css": "#456",

"after_code": "require_script 'lims_helper'\n\nif subj.get_value('Amount of DNA (ug)') < 10\n patient = subj.get_value('Patient')\n recipients = []\n recipients << find_user_group('Low DNA amount notification list')\n if patient\n physician = patient.get_value('Primary Clinician')\n if physician\n recipients << physician\n send_email(recipients , find_email_template('Low DNA Amount Alert'), subj)\n else\n send_email(recipients , find_email_template('Low DNA Amount Alert'), subj)\n end \n end\nend\n\nif subj.get_value('OD260/280') < 1.60\n patient = subj.get_value('Patient')\n recipients = []\n recipients << find_user_group('Low DNA amount notification list')\n if patient\n physician = patient.get_value('Primary Clinician')\n if physician\n recipients << physician\n send_email(recipients , find_email_template('Low DNA Quality Alert'), subj)\n else\n send_email(recipients , find_email_template('Low DNA Quality Alert'), subj)\n end\n end\nend\n\n#vial_count = params['Number of Vials to Store']\nself.next_state_performer = User.curr_user\nsubmit_to_fpro(subj, 'DNA', '7000000518', 1) do |s|\n \ts.set_value('OD 230', subj.get_value('OD 230'))\n s.set_value('OD 260', subj.get_value('OD 260'))\n s.set_value('OD 280', subj.get_value('OD 280'))\n s.set_value('Concentration (ng/ul)', subj.get_value('Concentration (ng/ul)'))\nend\n\n",

"btn_scale": "large",

"conditions": [

{

"name": "DNA Extraction&rarr;Concentration (ng/ul)",

"condition": "Not Empty",

"value": "",

"message": "Recording of Concetration, OD 230, OD 260, OD 280 and Volume are required to advance",

"subject_type": "UDPM-122",

"udf": "UDPM-1977"

},

{

"name": "DNA Extraction&rarr;OD 230",

"condition": "Not Empty",

"value": "",

"message": "Recording of Concetration, OD 230, OD 260, OD 280 and Volume are required to advance",

"subject_type": "UDPM-122",

"udf": "UDPM-1016"

},

{

"name": "DNA Extraction&rarr;OD 260",

"condition": "Not Empty",

"value": "",

"message": "Recording of Concetration, OD 230, OD 260, OD 280 and Volume are required to advance",

"subject_type": "UDPM-122",

"udf": "UDPM-627"

},

{

"name": "DNA Extraction&rarr;OD 280",

"condition": "Not Empty",

"value": "",

"message": "Recording of Concetration, OD 230, OD 260, OD 280 and Volume are required to advance",

"subject_type": "UDPM-122",

"udf": "UDPM-628"

},

{

"name": "DNA Extraction&rarr;Volume (uL)",

"condition": "Not Empty",

"value": "",

"message": "Recording of Concetration, OD 230, OD 260, OD 280 and Volume are required to advance",

"subject_type": "UDPM-122",

"udf": "UDPM-452"

}

]

},

{

"state_name": "Cancel",

"state_uuid": "UDPM-496",

"btn_text": "Discard Sample",

"btn_css": "#456",

"after_code": "# Extra code here\nsubj.set_value('Reason for Termination', params['Rationale/Reasoning'] )\n\n#sample = subj.get_value('Research Sample')",

"after_code_params": [

"UDPM-883"

]

}

],

"show_options": {

"top": 158,

"left": 111

}

},

{

"name": "Store DNA for Transport",

"uuid": "UDPM-593",

"owner": "UDPM-67",

"managers": "UDPM-22",

"performers": "UDPM-69",

"show_options": {

"top": 62,

"left": 569

},

"end_task": true

},

{

"name": "Temporary Storage at Twinbrook",

"uuid": "UDPM-1060",

"owner": "UDPM-67",

"managers": "UDPM-22",

"performers": "UDPM-56",

"show_options": {

"top": 328,

"left": 1020.2166748046875

},

"end_task": true

}

],

"entry_point": "UDPM-575",

"ask_performer": true

},{

"name": "UDP Inquiry",

"uuid": "UDPM-55",

"subject_type": "UDPM-61",

"state_defs": [

{

"name": "Enrolled (Existing Family)",

"uuid": "UDPM-180",

"owner": "UDPM-67",

"managers": "UDPM-9",

"performers": "UDPM-2",

"duration": 14.0,

"show_options": {

"top": 293.00001525878906,

"left": 461

},

"end_task": true

},

{

"name": "Enrolled (New Family)",

"uuid": "UDPM-179",

"owner": "UDPM-67",

"managers": "UDPM-9",

"performers": "UDPM-2",

"duration": 14.0,

"show_options": {

"top": 108,

"left": 640

},

"end_task": true

},

{

"name": "Indefinite Query Hold",

"uuid": "UDPM-178",

"owner": "UDPM-67",

"managers": "UDPM-9",

"performers": "UDPM-5",

"tools": [

{

"name": "Release Hold",

"uuid": "UDPM-1078",

"input_type": "UDPM-61",

"output_type": "UDPM-61",

"description": "Take case back to Inquiry Triage",

"flags": 6,

"after_code": "# Script to run After Tool is executed\nadvance_workflow(\"UDP Inquiry\",\"Inquiry Triage\", subj)\nself.next_state_performer = User.curr_user"

}

],

"show_options": {

"top": 313,

"left": 138

},

"end_task": true

},

{

"name": "Inquiry Triage",

"uuid": "UDPM-177",

"owner": "UDPM-67",

"managers": "UDPM-9",

"performers": "UDPM-49",

"duration": 14.0,

"tools": [

{

"name": "Edit Patient Information",

"uuid": "UDPM-299",

"input_type": "UDPM-61",

"output_type": "UDPM-61",

"flags": 1,

"options": {

"udfs": [

"UDPM-1847",

"UDPM-2",

"UDPM-616",

"UDPM-3",

"UDPM-1",

"UDPM-4",

"UDPM-56",

"UDPM-2202",

"UDPM-562",

"UDPM-184",

"UDPM-645",

"UDPM-1846",

"UDPM-739",

"UDPM-505",

"UDPM-731",

"UDPM-857",

"UDPM-528",

"UDPM-54",

"UDPM-1848",

"UDPM-1849",

"UDPM-1850",

"UDPM-1851",

"UDPM-1852",

"UDPM-1853",

"UDPM-1854",

"UDPM-188",

"UDPM-151",

"UDPM-1722",

"UDPM-43",

"UDPM-191"

]

}

}

],

"next_states": [

{

"state_name": "Enrolled (Existing Family)",

"state_uuid": "UDPM-180",

"btn_text": "Enroll into Existing Family",

"btn_css": "#456",

"after_code": "raise \"Please provide a valid family number\" unless params['Family']\n\nfamilyname = params['Family']\n\npatient = create_subject('Patient') do |p|\n p.set_value('Family', familyname)\n p.set_value('UDP Inquiry', subj)\n p.copy_properties_from(subj)\nend\n\nstart_workflow('Patient Workflow', patient)\nstate = patient.current_states[0]\nstate.proc_complete=50\nuser = find_user('[redacted]')\nstate.performer=user\nstate.save\n\nshow_message(\"The new UDP Number is successfully created, if you would like to continue working on the newly created UDP Subject, \n please click on the link UDP Patient\")",

"after_code_params": [

"UDPM-15"

]

},

{

"state_name": "Indefinite Query Hold",

"state_uuid": "UDPM-178",

"btn_text": "Place into Indefinite Query Hold",

"btn_css": "#456"

},

{

"state_name": "Enrolled (New Family)",

"state_uuid": "UDPM-179",

"btn_text": "Enroll into New Family",

"btn_css": "#456",

"after_code": "#raise \"Please provide a valid family number\" unless params[\"New Family Name\"]\n\n#familyname = params[\"New Family Name\"]\n#if (familyname =~ /UDP.d+/)\n# msg1 =\"Family UDP ID is <b> #{familyname}</b> <br>\"\n#else\n# msg1 = \"A new family with non standard family code #{familyname} was created. Please correct the Family ID to UDP_XXX format <br>\"\n#end\n#family = create_subject('Family', {:name=>familyname})\n\npatient = create_subject('Patient') do |p|\n p.set_value('UDP Inquiry', subj)\n p.copy_properties_from(subj)\nend\n\nfamilyName = patient.to_s.sub('_', '.')\nfamily = create_subject('Family', {:name=>familyName})\npatient.set_value('Family', family)\n\npedigree = family.get_value('Pedigree Tree')\nproband = pedigree.get_proband\n\nstart_workflow('Patient Workflow', patient)\nstate = patient.current_states[0]\nstate.proc_complete=50\nuser = find_user('[redacted]')\nstate.performer=user\nstate.save\n\n\nmsg =\"A new patient with the UDP number #{patient} has been created with the family number #{family}\"\nshow_message(msg)\n\n\n",

"btn_scale": "large"

}

],

"show_options": {

"top": 55,

"left": 119

}

}

],

"entry_point": "UDPM-177",

"ask_performer": true,

"show_udfs": "UDPM-1848,UDPM-1849,UDPM-1850,UDPM-1851,UDPM-1852,UDPM-1853,UDPM-1854,UDPM-562,UDPM-505,UDPM-528,UDPM-616,UDPM-3,UDPM-1,UDPM-2,UDPM-54,UDPM-184,UDPM-1847,UDPM-191,UDPM-52,UDPM-53,UDPM-739,UDPM-56,UDPM-731,UDPM-857,UDPM-11"

},{

"name": "Patient Material Management - DELETE",

"uuid": "UDPM-91",

"subject_type": "UDPM-32",

"state_defs": [

{

"name": "Received",

"uuid": "UDPM-365",

"owner": "UDPM-67",

"managers": "UDPM-9",

"performers": "UDPM-5",

"duration": 5.0,

"tools": [

{

"name": "Update Location",

"uuid": "UDPM-399",

"input_type": "UDPM-32",

"output_type": "UDPM-32",

"flags": 1,

"options": {

"udfs": [

"UDPM-114",

"UDPM-339",

"UDPM-5"

]

}

}

],

"show_options": {

"top": 97,

"left": 572

},

"end_task": true

},

{

"name": "Requested",

"uuid": "UDPM-377",

"owner": "UDPM-67",

"managers": "UDPM-9",

"performers": "UDPM-5",

"tools": [

{

"name": "Materials Rec'd Info",

"uuid": "UDPM-1156",

"input_type": "UDPM-32",

"output_type": "UDPM-32",

"flags": 1,

"options": {

"udfs": [

"UDPM-6",

"UDPM-438",

"UDPM-5",

"UDPM-441"

]

}

},

{

"name": "Return to UDP Triage",

"uuid": "UDPM-1155",

"input_type": "UDPM-32",

"output_type": "UDPM-1",

"flags": 0,

"options": {

"obj_type": "SubjectType"

}

}

],

"next_states": [

{

"state_name": "Received",

"state_uuid": "UDPM-365",

"btn_text": "Receive Material",

"btn_css": "#456",

"after_code": "# Extra code here\nsubj.set_value('Date Received', params['Date Received'])\nsubj.set_value('Location of Materials', params['Location of Materials'])\n\nself.next_state_performer = User.curr_user",

"after_code_params": [

"UDPM-114",

"UDPM-339"

]

}

],

"show_options": {

"top": 50.00001525878906,

"left": 62.000030517578125

}

}

],

"entry_point": "UDPM-377",

"ask_performer": true,

"show_udfs": "UDPM-438,UDPM-5"

},{

"name": "Sanger Interpretation Workflow",

"uuid": "UDPM-10",

"subject_type": "UDPM-22",

"state_defs": [

{

"name": "Complete Sanger Validation",

"uuid": "UDPM-784",

"owner": "UDPM-67",

"managers": "UDPM-22",

"performers": "UDPM-21",

"tools": [

{

"name": "Upload AB1 File",

"uuid": "UDPM-1277",

"input_type": "UDPM-22",

"output_type": "UDPM-250",

"flags": 0,

"options": {

"obj_type": "SubjectType"

}

},

{

"name": "Conclusion",

"uuid": "UDPM-980",

"input_type": "UDPM-22",

"output_type": "UDPM-22",

"flags": 1,

"options": {

"udfs": [

"UDPM-900",

"UDPM-901",

"UDPM-905",

"UDPM-1872",

"UDPM-2046"

]

}

}

],

"show_options": {

"top": 265,

"left": 321

},

"end_task": true

},

{

"name": "Preparation for Sequencing",

"uuid": "UDPM-47",

"owner": "UDPM-67",

"managers": "UDPM-22",

"performers": "UDPM-21",

"duration": 5.0,

"tools": [

{

"name": "PCR Amplification",

"uuid": "UDPM-998",

"input_type": "UDPM-22",

"output_type": "UDPM-22",

"flags": 1,

"options": {

"udfs": [

"UDPM-191",

"UDPM-893",

"UDPM-2515",

"UDPM-2419",

"UDPM-455",

"UDPM-1280",

"UDPM-1693"

]

}

}

],

"next_states": [

{

"state_name": "Sent for Validation",

"state_uuid": "UDPM-488",

"btn_text": "Send for Sequencing",

"btn_css": "#456",

"after_code": "self.next_state_performer = User.curr_user\nsubj.set_value('Date Sent for Sanger Validation', params['Date Sent for Sanger Validation'])",

"after_code_params": [

"UDPM-1166"

],

"hide_button_if_not_condition": true

}

],

"show_options": {

"top": 77,

"left": 69

}

},

{

"name": "Sent for Validation",

"uuid": "UDPM-488",

"owner": "UDPM-67",

"managers": "UDPM-22",

"performers": "UDPM-21",

"next_states": [

{

"state_name": "Complete Sanger Validation",

"state_uuid": "UDPM-784",

"btn_text": "Complete Sanger Validation",

"btn_css": "#456"

}

],

"show_options": {

"top": 106,

"left": 406

}

}

],

"entry_point": "UDPM-47",

"ask_performer": true,

"show_udfs": "UDPM-2448,UDPM-2594,UDPM-2593,UDPM-191,UDPM-1277"

},{

"name": "Cell Culture Workflow",

"uuid": "UDPM-26",

"subject_type": "UDPM-40",

"state_defs": [

{

"name": "Cell Culture Started",

"uuid": "UDPM-101",

"owner": "UDPM-67",

"managers": "UDPM-22",

"performers": "UDPM-26",

"duration": 30.0,

"tools": [

{

"name": "2) Mycoplasma Test Results",

"uuid": "UDPM-106",

"input_type": "UDPM-40",

"output_type": "UDPM-40",

"flags": 1,

"hide_button_if_not_condition": true,

"conditions": [

{

"name": "Cell Culture&rarr;Date Treatment Started",

"condition": "Empty",

"value": "",

"message": "",

"subject_type": "UDPM-40",

"udf": "UDPM-457"

}

],

"options": {

"udfs": [

"UDPM-337",

"UDPM-1042",

"UDPM-1666",

"UDPM-1667",

"UDPM-455",

"UDPM-5"

]

}

},

{

"name": "Generate iPS Cells",

"uuid": "UDPM-415",

"input_type": "UDPM-40",

"output_type": "UDPM-41",

"flags": 0,

"hide_button_if_not_condition": true,

"options": {

"obj_type": "SubjectType"

}

},

{

"name": "1) Additional Information",

"uuid": "UDPM-809",

"input_type": "UDPM-40",

"output_type": "UDPM-40",

"flags": 1,

"options": {

"udfs": [

"UDPM-1035",

"UDPM-415",

"UDPM-2561",

"UDPM-1669",

"UDPM-1665",

"UDPM-1332",

"UDPM-1670",

"UDPM-5",

"UDPM-2200",

"UDPM-2374"

]

}

},

{

"name": "Send for External Testing",

"uuid": "UDPM-811",

"input_type": "UDPM-40",

"output_type": "UDPM-147",

"description": "Collaborator Testing",

"flags": 0,

"after_code": "next_state = params[\"culture_workflow_states\"]\nadvance_workflow(\"Cell Culture Workflow\",'Shipped for Testing',subj)",

"options": {

"obj_type": "SubjectType"

}

},

{

"name": "Passage Cells",

"uuid": "UDPM-843",

"input_type": "UDPM-40",

"output_type": "UDPM-40",

"flags": 0,

"before_code": "f = subj.get_value(\"Passage\")\n \nparams[:defaults] = {\n 'Passage'=> f\n}\n",

"options": {

"obj_type": "SubjectType"

}

},

{

"name": "3) Transduction Information",

"uuid": "UDPM-844",

"input_type": "UDPM-40",

"output_type": "UDPM-40",

"flags": 1,

"before_code": "f = subj.get_value(\"Sample Type\")\n\nparams[:defaults] = {\n 'Cell Type'=> f\n}\n",

"options": {

"udfs": [

"UDPM-1833",

"UDPM-1834",

"UDPM-2502",

"UDPM-1999",

"UDPM-2503",

"UDPM-2374"

]

}

},

{

"name": "2) Retested Mycoplasma Results",

"uuid": "UDPM-845",

"input_type": "UDPM-40",

"output_type": "UDPM-40",

"flags": 1,

"hide_button_if_not_condition": true,

"conditions": [

{

"name": "Cell Culture&rarr;Date Treatment Started",

"condition": "Not Empty",

"value": "",

"message": "",

"subject_type": "UDPM-40",

"udf": "UDPM-457"

}

],

"options": {

"udfs": [

"UDPM-337",

"UDPM-1042",

"UDPM-1841",

"UDPM-1842",

"UDPM-1843"

]

}

},

{

"name": "Print Barcode",

"uuid": "UDPM-902",

"input_type": "UDPM-40",

"output_type": "UDPM-40",

"flags": 2,

"options": {

"obj_type": "Printer",

"labels_count": 1,

"obj_name": "UDPM-19"

}

},

{

"name": "Perform Assay",

"uuid": "UDPM-1221",

"input_type": "UDPM-40",

"output_type": "UDPM-225",

"flags": 0,

"after_code": "self.next_state_performer = User.curr_user",

"options": {

"obj_type": "SubjectType"

}

},

{

"name": "Send for Glycomics Testing",

"uuid": "UDPM-1315",

"input_type": "UDPM-40",

"output_type": "UDPM-244",

"flags": 0,

"options": {

"obj_type": "SubjectType"

}

},

{

"name": "Extract RNA",

"uuid": "UDPM-1359",

"input_type": "UDPM-40",

"output_type": "UDPM-43",

"flags": 0,

"options": {

"obj_type": "SubjectType"

}

}

],

"next_states": [

{

"state_name": "Shipped for Testing",

"state_uuid": "UDPM-648",

"btn_text": "Ready for Shipment",

"btn_css": "#456"

},

{

"state_name": "Discarded",

"state_uuid": "UDPM-447",

"btn_text": "Discard",

"btn_css": "#456",

"after_code": "# Extra code here\nsubj.set_value('Rationale/Reasoning', params['Rationale/Reasoning'] )\n\n#sample = subj.get_value('Research Sample')\n",

"after_code_params": [

"UDPM-883"

]

},

{

"state_name": "Treat Cell Line",

"state_uuid": "UDPM-372",

"btn_text": "Treat Cell Line",

"btn_css": "#456",

"after_code": "self.next_state_performer = User.curr_user",

"hide_button_if_not_condition": true,

"conditions": [

{

"name": "Cell Culture&rarr;Mycoplasma Contaminated",

"condition": "Not Empty",

"value": "",

"message": "",

"subject_type": "UDPM-40",

"udf": "UDPM-1667"

}

]

},

{

"state_name": "Culture Passaged",

"state_uuid": "UDPM-690",

"btn_text": "Culture Used",

"btn_css": "#456"

},

{

"state_name": "Stored",

"state_uuid": "UDPM-379",

"btn_text": "Freeze Cells",

"btn_css": "#456",

"after_code": "require_script 'culture_helper'\nvial_count = params['Number of Vials to Store']\nif User.curr_user.user_groups.map(&:name).join(',').include? \"Lab Personnel - Twinbrook\"\n if subj.get_value('Mycoplasma Free') == 'true' || subj.get_value('Mycoplasma Free after Treatment') == 'true'\n if subj.get_value('Storage Temp') == 'Cell Line (-150)'\n submit_to_fpro(subj, 'Skin Fibroblast', '7000000121', vial_count) do |s|\n end\n elsif subj.get_value('Storage Temp') == 'Cell Pellet (-80)'\n submit_to_fpro(subj, 'Cell Pellet', '7000000749', vial_count) do |s|\n end\n elsif subj.get_value('Storage Temp') == 'Culture Media (-80)'\n submit_to_fpro(subj, 'Culture media', '7000000749', vial_count) do |s|\n end\n else\n raise 'You must enter a storage temperature to proceed'\n end\n else\n raise \"Your cell line must be free of mycoplasma in order to freeze\"\n end\nelsif User.curr_user.user_groups.map(&:name).join(',').include? \"Lab Personnel - Building 50\"\n if subj.get_value('Mycoplasma Free') == 'true' || subj.get_value('Mycoplasma Free after Treatment') == 'true'\n if subj.get_value('Storage Temp') == 'Cell Line (-150)'\n submit_to_fpro(subj, 'Skin Fibroblast', '7000000417', vial_count) do |s|\n end\n elsif subj.get_value('Storage Temp') == 'Cell Pellet (-80)'\n submit_to_fpro(subj, 'Cell Pellet', '7000000623', vial_count) do |s|\n end\n elsif subj.get_value('Storage Temp') == 'Culture Media (-80)'\n submit_to_fpro(subj, 'Culture media', '7000000623', vial_count) do |s|\n end\n else\n raise 'You must enter a storage temperature to proceed'\n end\n else\n raise \"Your cell line must be free of mycoplasma in order to freeze\"\n end\nelse\n raise(\"This script only allows users who belong to either Twinbrook Lab or Building 50 Lab, please contact the administrators\")\nend",

"btn_scale": "large",

"after_code_params": [

"UDPM-1450"

],

"disable_button_if_not_condition": true

}

],

"show_options": {

"top": 50.66667175292969,

"left": 396

}

},

{

"name": "Culture Passaged",

"uuid": "UDPM-690",

"owner": "UDPM-67",

"managers": "UDPM-22",

"performers": "UDPM-26",

"show_options": {

"top": 425,

"left": 271

},

"end_task": true

},

{

"name": "Discarded",

"uuid": "UDPM-447",

"owner": "UDPM-67",

"managers": "UDPM-22",

"performers": "UDPM-26",

"show_options": {

"top": 406,

"left": 749

},

"end_task": true

},

{

"name": "Shipped for Testing",

"uuid": "UDPM-648",

"owner": "UDPM-67",

"managers": "UDPM-22",

"performers": "UDPM-26",

"show_options": {

"top": 337,

"left": 131

},

"end_task": true

},

{

"name": "Stored",

"uuid": "UDPM-379",

"owner": "UDPM-67",

"managers": "UDPM-22",

"performers": "UDPM-26",

"show_options": {

"top": 145,

"left": 890

},

"end_task": true

},

{

"name": "Treat Cell Line",

"uuid": "UDPM-372",

"owner": "UDPM-67",

"managers": "UDPM-22",

"performers": "UDPM-26",

"tools": [

{

"name": "Treat Cell Line",

"uuid": "UDPM-408",

"input_type": "UDPM-40",

"output_type": "UDPM-40",

"flags": 1,

"options": {

"udfs": [

"UDPM-457"

]

}

}

],

"next_states": [

{

"state_name": "Cell Culture Started",

"state_uuid": "UDPM-101",

"btn_text": "Retest for Mycoplasma",

"btn_css": "#456",

"after_code": "self.next_state_performer = User.curr_user"

},

{

"state_name": "Discarded",

"state_uuid": "UDPM-447",

"btn_text": "Discard",

"btn_css": "#456",

"after_code": "subj.set_value('Rationale/Reasoning', params['Rationale/Reasoning'] )",

"after_code_params": [

"UDPM-883"

]

}

],

"show_options": {

"top": 429,

"left": 454

}

}

],

"entry_point": "UDPM-101",

"ask_performer": true,

"show_udfs": "UDPM-2415,UDPM-415,UDPM-449"

},{

"name": "Plasma Extraction",

"uuid": "UDPM-39",

"subject_type": "UDPM-47",

"state_defs": [

{

"name": "Archive Plasma",

"uuid": "UDPM-413",

"owner": "UDPM-67",

"managers": "UDPM-22",

"performers": "UDPM-69",

"show_options": {

"top": 271,

"left": 508

},

"end_task": true

},

{

"name": "Discarded",

"uuid": "UDPM-672",

"owner": "UDPM-67",

"managers": "UDPM-22",

"performers": "UDPM-69",

"show_options": {

"top": 95,

"left": 511

},

"end_task": true

},

{

"name": "Plasma Extracted",

"uuid": "UDPM-577",

"owner": "UDPM-67",

"managers": "UDPM-22",

"performers": "UDPM-69",

"duration": 1.0,

"tools": [

{

"name": "1) Plasma Processing Information",

"uuid": "UDPM-665",

"input_type": "UDPM-47",

"output_type": "UDPM-47",

"flags": 1,

"options": {

"udfs": [

"UDPM-1017",

"UDPM-1298",

"UDPM-1024",

"UDPM-1020",

"UDPM-1332",

"UDPM-1021",

"UDPM-5"

]

}

}

],

"next_states": [

{

"state_name": "Archive Plasma",

"state_uuid": "UDPM-413",

"btn_text": "2) Archive Plasma",

"btn_css": "#456",

"after_code": "require_script 'lims_helper'\n\nvial_count = params['Number of Vials to Store']\nvial_volumes = params['Volume for the vials']\nself.next_state_performer = User.curr_user\nsubmit_to_fpro(subj, 'Blood Plasma/Serum', '7000000522', vial_count, vial_volumes) do |s|\n\ts.set_value('Description', subj.get_value('Comments'))\nend",

"btn_scale": "large",

"after_code_params": [

"UDPM-1450",

"UDPM-2664"

],

"conditions": [

{

"name": "Plasma Extraction&rarr;Volume (mL)",

"condition": "Not Empty",

"value": "",

"message": "Volume is required to advance",

"subject_type": "UDPM-47",

"udf": "UDPM-1332"

}

]

},

{

"state_name": "Discarded",

"state_uuid": "UDPM-672",

"btn_text": "Discard",

"btn_css": "#456",

"after_code": "subj.set_value('Rationale/Reasoning', params['Rationale/Reasoning'] )",

"after_code_params": [

"UDPM-883"

]

}

],

"show_options": {

"top": 226.99998474121094,

"left": 175

}

}

],

"entry_point": "UDPM-577",

"ask_performer": true

},{

"name": "Exome Analysis Workflow",

"uuid": "UDPM-48",

"subject_type": "UDPM-60",

"state_defs": [

{

"name": "Cancel",

"uuid": "UDPM-975",

"owner": "UDPM-67",

"managers": "UDPM-27",

"performers": "UDPM-30",

"show_options": {

"top": 248.99998474121094,

"left": 83.00003051757812

},

"end_task": true

},

{

"name": "Case Closed",

"uuid": "UDPM-788",

"owner": "UDPM-67",

"managers": "UDPM-27",

"performers": "UDPM-27",

"show_options": {

"top": 69,

"left": 508

},

"end_task": true

},

{

"name": "Exome Analysis",

"uuid": "UDPM-152",

"owner": "UDPM-67",

"managers": "UDPM-27",

"performers": "UDPM-30",

"duration": 14.0,

"tools": [

{

"name": "Upload Exomiser Files",

"uuid": "UDPM-1502",

"input_type": "UDPM-60",

"output_type": "UDPM-60",

"flags": 1,

"options": {

"udfs": [

"UDPM-2885",

"UDPM-2886",

"UDPM-2887",

"UDPM-2888",

"UDPM-2889",

"UDPM-2890",

"UDPM-2891"

]

}

},

{

"name": "Analysis Information",

"uuid": "UDPM-349",

"input_type": "UDPM-60",

"output_type": "UDPM-60",

"flags": 1,

"options": {

"udfs": [

"UDPM-1844",

"UDPM-1767",

"UDPM-2054",

"UDPM-2058",

"UDPM-2586",

"UDPM-1764",

"UDPM-380",

"UDPM-381",

"UDPM-382",

"UDPM-383",

"UDPM-384",

"UDPM-385",

"UDPM-386",

"UDPM-1277",

"UDPM-387",

"UDPM-174",

"UDPM-2562",

"UDPM-2563",

"UDPM-1866",

"UDPM-5"

]

}

},

{

"name": "Add Variant",

"uuid": "UDPM-886",

"input_type": "UDPM-60",

"output_type": "UDPM-156",

"flags": 0,

"options": {

"obj_type": "SubjectType"

}

}

],

"next_states": [

{

"state_name": "Cancel",

"state_uuid": "UDPM-975",

"btn_text": "Cancel Exome Analysis",

"btn_css": "#456",

"after_code": "subj.set_value(\"Rationale/Reasoning\", params['Rationale/Reasoning'])",

"btn_scale": "large",

"after_code_params": [

"UDPM-883"

]

},

{

"state_name": "Exome Analysis Complete",

"state_uuid": "UDPM-314",

"btn_text": "Exome Analysis Complete",

"btn_css": "#456",

"after_code": "p = subj.get_value('Patient')\n if p\n recipients = []\n physician = p.get_value('Attending Physician')\n clinician = p.get_value('Primary Clinician')\n recipients << physician if physician\n recipients << clinician if clinician\n recipients << User.find_by_username('[redacted]')\n end\n\nif recipients.any?\n send_email(recipients, find_email_template(\"Exome Analysis Complete\"), subj)\nend\n\natt = p.get_value('Attending Physician')\nself.next_state_performer = att",

"btn_scale": "large"

}

],

"show_options": {

"top": 52.850006103515625,

"left": 97.61663818359375

}

},

{

"name": "Exome Analysis Complete",

"uuid": "UDPM-314",

"owner": "UDPM-67",

"managers": "UDPM-9",

"performers": "UDPM-49",

"next_states": [

{

"state_name": "Review Complete",

"state_uuid": "UDPM-966",

"btn_text": "Analysis Review Complete",

"btn_css": "#456"

}

],

"show_options": {

"top": 219.85000610351562,

"left": 307.1333312988281

}

},

{

"name": "Review Complete",

"uuid": "UDPM-966",

"owner": "UDPM-67",

"managers": "UDPM-9",

"performers": "UDPM-49",

"show_options": {

"top": 369,

"left": 645

},

"end_task": true

}

],

"entry_point": "UDPM-152",

"ask_performer": true,

"show_udfs": "UDPM-1767,UDPM-5,UDPM-1844,UDPM-1277"

},{

"name": "Patient Documents Scan & Sort",

"uuid": "UDPM-92",

"subject_type": "UDPM-88",

"state_defs": [

{

"name": "Completed",

"uuid": "UDPM-376",

"owner": "UDPM-67",

"managers": "UDPM-9",

"performers": "UDPM-11",

"show_options": {

"top": 246.83333587646484,

"left": 751.9999694824219

},

"end_task": true

},

{

"name": "Document Scanning",

"uuid": "UDPM-375",

"owner": "UDPM-67",

"managers": "UDPM-9",

"performers": "UDPM-11",

"duration": 5.0,

"tools": [

{

"name": "Date Scanned",

"uuid": "UDPM-412",

"input_type": "UDPM-88",

"output_type": "UDPM-88",

"flags": 1,

"options": {

"udfs": [

"UDPM-221",

"UDPM-447"

]

}

}

],

"next_states": [

{

"state_name": "Completed",

"state_uuid": "UDPM-376",

"btn_text": "Complete",

"btn_css": "#456",

"after_code": "# Extra code here\n",

"before_code": "# Script to run Before Transition is executed\nparams[:ask_next_performer] = true"

}

],

"show_options": {

"top": 85.33333587646484,

"left": 502.9999694824219

}

},

{

"name": "Document Sorting",

"uuid": "UDPM-374",

"owner": "UDPM-67",

"managers": "UDPM-9",

"performers": "UDPM-11",

"duration": 5.0,

"tools": [

{

"name": "Date Sorted",

"uuid": "UDPM-411",

"input_type": "UDPM-88",

"output_type": "UDPM-88",

"flags": 1,

"options": {

"udfs": [

"UDPM-444",

"UDPM-447"

]

}

}

],

"next_states": [

{

"state_name": "Document Scanning",

"state_uuid": "UDPM-375",

"btn_text": "Proceed to Scanning",

"btn_css": "#456",

"after_code": "# Extra code here\n",

"before_code": "# Script to run Before Transition is executed\nparams[:ask_next_performer] = true"

}

],

"show_options": {

"top": 219.99999237060547,

"left": 279.6666564941406

}

},

{

"name": "Scan & Sort Requested",

"uuid": "UDPM-373",

"owner": "UDPM-67",

"managers": "UDPM-9",

"performers": "UDPM-11",

"duration": 3.0,

"next_states": [

{

"state_name": "Document Sorting",

"state_uuid": "UDPM-374",

"btn_text": "Start Sorting",

"btn_css": "#456",

"after_code": "# Extra code here\n",

"before_code": "# Script to run Before Transition is executed\nparams[:ask_next_performer] = true"

}

],

"show_options": {

"top": 47,

"left": 31

}

}

],

"entry_point": "UDPM-373",

"ask_performer": true

},{

"name": "CSF Workflow",

"uuid": "UDPM-95",

"subject_type": "UDPM-117",

"state_defs": [

{

"name": "Archive CSF",

"uuid": "UDPM-406",

"owner": "UDPM-67",

"managers": "UDPM-22",

"performers": "UDPM-69",

"show_options": {

"top": 188,

"left": 327

},

"end_task": true

},

{

"name": "Process CSF",

"uuid": "UDPM-576",

"owner": "UDPM-67",

"managers": "UDPM-22",

"performers": "UDPM-69",

"duration": 1.0,

"tools": [

{

"name": "1) Sample Information",

"uuid": "UDPM-663",

"input_type": "UDPM-117",

"output_type": "UDPM-117",

"flags": 1,

"options": {

"udfs": [

"UDPM-1017",

"UDPM-1014",

"UDPM-1005",

"UDPM-191",

"UDPM-1298",

"UDPM-1006",

"UDPM-1007",

"UDPM-1332"

]

}

}

],

"next_states": [

{

"state_name": "Archive CSF",

"state_uuid": "UDPM-406",

"btn_text": "2) Archive CSF",

"btn_css": "#456",

"after_code": "require_script 'lims_helper'\n\nvial_count = params['Number of Vials to Store']\nvial_volumes = params['Volume for the vials']\nself.next_state_performer = User.curr_user\nsubmit_to_fpro(subj, 'CSF', '7000000525', vial_count, vial_volumes)",

"btn_scale": "large",

"after_code_params": [

"UDPM-1450",

"UDPM-2664"

],

"conditions": [

{

"name": "CSF Processing&rarr;Volume (mL)",

"condition": "Not Empty",

"value": "",

"message": "Volume must be recorded to advance",

"subject_type": "UDPM-117",

"udf": "UDPM-1332"

}

]

}

],

"show_options": {

"top": 30,

"left": 105

}

}

],

"entry_point": "UDPM-576",

"ask_performer": true

},{

"name": "Serum Extraction",

"uuid": "UDPM-97",

"subject_type": "UDPM-119",

"state_defs": [

{

"name": "Archive Serum",

"uuid": "UDPM-416",

"owner": "UDPM-67",

"managers": "UDPM-22",

"performers": "UDPM-69",

"show_options": {

"top": 243,

"left": 506

},

"end_task": true

},

{

"name": "Cancel",

"uuid": "UDPM-994",

"owner": "UDPM-67",

"managers": "UDPM-22",

"performers": "UDPM-69",

"show_options": {

"top": 104,

"left": 330

},

"end_task": true

},

{

"name": "Receive Blood",

"uuid": "UDPM-578",

"owner": "UDPM-67",

"managers": "UDPM-22",

"performers": "UDPM-69",

"duration": 1.0,

"tools": [

{

"name": "Serum Extraction Information",

"uuid": "UDPM-668",

"input_type": "UDPM-119",

"output_type": "UDPM-119",

"flags": 1,

"options": {

"udfs": [

"UDPM-1298",

"UDPM-1017",

"UDPM-1024",

"UDPM-1027",

"UDPM-1332",

"UDPM-1025",

"UDPM-5"

]

}

}

],

"next_states": [

{

"state_name": "Archive Serum",

"state_uuid": "UDPM-416",

"btn_text": "Archive Serum",

"btn_css": "#456",

"after_code": "require_script 'lims_helper'\n\nvial_count = params['Number of Vials to Store']\nvial_volumes = params['Volume for the vials']\nself.next_state_performer = User.curr_user\nsubmit_to_fpro(subj, 'Blood Plasma/Serum', '7000000524', vial_count, vial_volumes) do |s|\n\ts.set_value('Description', subj.get_value('Comments'))\nend",

"btn_scale": "large",

"after_code_params": [

"UDPM-1450",

"UDPM-2664"

],

"conditions": [

{

"name": "Serum Extraction&rarr;Volume (mL)",

"condition": "Not Empty",

"value": "",

"message": "Volume is required to advance",

"subject_type": "UDPM-119",

"udf": "UDPM-1332"

}

]

},

{

"state_name": "Cancel",

"state_uuid": "UDPM-994",

"btn_text": "Discard Sample",

"btn_css": "#456",

"after_code": "subj.set_value('Rationale/Reasoning', params['Rationale/Reasoning'])",

"btn_scale": "large",

"after_code_params": [

"UDPM-883"

]

}

],

"show_options": {

"top": 247.9971466064453,

"left": 162.98861694335938

}

}

],

"entry_point": "UDPM-578",

"ask_performer": true

},{

"name": "Urine Workflow",

"uuid": "UDPM-98",

"subject_type": "UDPM-120",

"state_defs": [

{

"name": "Archive Urine",

"uuid": "UDPM-498",

"owner": "UDPM-67",

"managers": "UDPM-22",

"performers": "UDPM-69",

"show_options": {

"top": 318,

"left": 732

},

"end_task": true

},

{

"name": "Cancelled",

"uuid": "UDPM-980",

"owner": "UDPM-67",

"managers": "UDPM-22",

"performers": "UDPM-69",

"show_options": {

"top": 143,

"left": 471

},

"end_task": true

},

{

"name": "Receive Urine Sample",

"uuid": "UDPM-579",

"owner": "UDPM-67",

"managers": "UDPM-22",

"performers": "UDPM-69",

"duration": 1.0,

"tools": [

{

"name": "Processing Information",

"uuid": "UDPM-671",

"input_type": "UDPM-120",

"output_type": "UDPM-120",

"flags": 1,

"options": {

"udfs": [

"UDPM-1017",

"UDPM-1298",

"UDPM-1031",

"UDPM-1312",

"UDPM-1332",

"UDPM-1033"

]

}

}

],

"next_states": [

{

"state_name": "Archive Urine",

"state_uuid": "UDPM-498",

"btn_text": "Archive Urine",

"btn_css": "#456",

"after_code": "require_script 'lims_helper'\n\nvial_count = params['Number of Vials to Store']\nvial_volumes = params['Volume for the vials']\nself.next_state_performer = User.curr_user\nsubmit_to_fpro(subj, 'Urine', '7000000523', vial_count, vial_volumes)",

"btn_scale": "large",

"after_code_params": [

"UDPM-1450",

"UDPM-2664"

],

"conditions": [

{

"name": "Urine&rarr;Volume (mL)",

"condition": "Not Empty",

"value": "",

"message": "Volume is required to advance",

"subject_type": "UDPM-120",

"udf": "UDPM-1332"

}

]

},

{

"state_name": "Cancelled",

"state_uuid": "UDPM-980",

"btn_text": "Cancel",

"btn_css": "#456",

"after_code": "subj.set_value('Rationale/Reasoning', params['Rationale/Reasoning'])",

"btn_scale": "large",

"after_code_params": [

"UDPM-883"

]

}

],

"show_options": {

"top": 288,

"left": 204

}

}

],

"entry_point": "UDPM-579",

"ask_performer": true

},{

"name": "Antibody Storage",

"uuid": "UDPM-157",

"subject_type": "UDPM-189",

"state_defs": [

{

"name": "Antibody Information",

"uuid": "UDPM-732",

"owner": "UDPM-67",

"managers": "UDPM-22",

"performers": "UDPM-4",

"tools": [

{

"name": "Antibody Information",

"uuid": "UDPM-896",

"input_type": "UDPM-189",

"output_type": "UDPM-189",

"flags": 1,

"options": {

"udfs": [

"UDPM-6",

"UDPM-1332",

"UDPM-1669",

"UDPM-1907",

"UDPM-1791",

"UDPM-1792",

"UDPM-5",

"UDPM-1908",

"UDPM-1909",

"UDPM-1910",

"UDPM-1911",

"UDPM-1912",

"UDPM-1913",

"UDPM-1914",

"UDPM-1915",

"UDPM-1916",

"UDPM-1917",

"UDPM-1918"

]

}

}

],

"next_states": [

{

"state_name": "Stored",

"state_uuid": "UDPM-733",

"btn_text": "Store Antibody",

"btn_css": "#456",

"after_code": "require_script 'antibody_helper'\n\nvial_count = params['Number of Vials to Store']\nif User.curr_user.user_groups.map(&:name).join(',').include? \"Lab Personnel - Twinbrook\"\n if subj.get_value('Antibody Type') == 'Monoclonal Ab'\n submit_to_fpro(subj, 'Monoclonal Ab', 251, vial_count) do |s|\n end\n elsif subj.get_value('Antibody Type') == 'Polyclonal Ab'\n submit_to_fpro(subj,'Polyclonal Ab', 251, vial_count) do |s|\n end\n elsif subj.get_value('Antibody Type') == 'Fluorescent Ab'\n submit_to_fpro(subj,'Antibody', 251, vial_count) do |s|\n end\n else\n raise \"You must specify the antibody type before proceeding\"\n end\n \nelsif User.curr_user.user_groups.map(&:name).join(',').include? \"Lab Personnel - Building 50\"\n if subj.get_value('Antibody Type') == 'Monoclonal Ab'\n submit_to_fpro(subj, 'Monoclonal Ab', 7000000624, vial_count) do |s|\n end\n elsif subj.get_value('Antibody Type') == 'Polyclonal Ab'\n submit_to_fpro(subj,'Polyclonal Ab', 7000000624, vial_count) do |s|\n end\n elsif subj.get_value('Antibody Type') == 'Fluorescent Ab'\n submit_to_fpro(subj,'Antibody', 7000000624, vial_count) do |s|\n end\n else\n raise \"You must specify the antibody type before proceeding\"\n end\nelse\n raise(\"This script only allows users who belong to either Twinbrook Lab or Building 50 Lab, please contact the administrators\")\nend\n",

"after_code_params": [

"UDPM-1450"

]

}

],

"show_options": {

"top": 106,

"left": 268

}

},

{

"name": "Stored",

"uuid": "UDPM-733",

"owner": "UDPM-67",

"managers": "UDPM-22",

"performers": "UDPM-4",

"show_options": {

"top": 187,

"left": 721

},

"end_task": true

}

],

"entry_point": "UDPM-732",

"show_udfs": "UDPM-1791,UDPM-5,UDPM-1792"

},{

"name": "Consult Request",

"uuid": "UDPM-162",

"subject_type": "UDPM-180",

"state_defs": [

{

"name": "Consult Request",

"uuid": "UDPM-760",

"owner": "UDPM-67",

"managers": "UDPM-49",

"performers": "UDPM-49",

"duration": 30.0,

"tools": [

{

"name": "Consult Request Info",

"uuid": "UDPM-922",

"input_type": "UDPM-180",

"output_type": "UDPM-180",

"flags": 1,

"options": {

"udfs": [

"UDPM-6",

"UDPM-1229",

"UDPM-1230",

"UDPM-1231",

"UDPM-1232",

"UDPM-1233",

"UDPM-1838",

"UDPM-1234",

"UDPM-1248",

"UDPM-1549"

]

}

}

],

"next_states": [

{

"state_name": "Consult Request Complete",

"state_uuid": "UDPM-822",

"btn_text": "Consult Complete",

"btn_css": "#456",

"after_code": "self.next_state_performer = find_user('[redacted]')",

"before_code": "# Script to run Before Transition is executed\n#params[:ask_next_performer] = true",

"btn_scale": "large"

}

],

"em_performer_due_job": true,

"show_options": {

"top": 63,

"left": 46

}

},

{

"name": "Consult Request Complete",

"uuid": "UDPM-822",

"owner": "UDPM-67",

"managers": "UDPM-9",

"performers": "UDPM-31",

"duration": 14.0,

"show_options": {

"top": 180,

"left": 400

},

"end_task": true

}

],

"entry_point": "UDPM-760",

"ask_performer": true,

"show_udfs": "UDPM-1838,UDPM-1549,UDPM-1231,UDPM-1230,UDPM-1232,UDPM-1233,UDPM-1234,UDPM-1248,UDPM-1229"

},{

"name": "Friday SNP and Exome Meeting",

"uuid": "UDPM-79",

"subject_type": "UDPM-82",

"state_defs": [

{

"name": "Added to Queue for Submission",

"uuid": "UDPM-636",

"owner": "UDPM-6",

"managers": "UDPM-61",

"performers": "UDPM-49",

"tools": [

{

"name": "Add Gene List",

"uuid": "UDPM-1407",

"input_type": "UDPM-82",

"output_type": "UDPM-256",

"flags": 0,

"options": {

"obj_type": "SubjectType",

"do_not_open_subject": true

}

}

],

"show_options": {

"top": 270.7166748046875,

"left": 423.95001220703125

},

"end_task": true

},

{

"name": "Case Closed",

"uuid": "UDPM-448",

"owner": "UDPM-6",

"managers": "UDPM-61",

"performers": "UDPM-49",

"show_options": {

"top": 284.98333740234375,

"left": 5.316650390625

},

"end_task": true

},

{

"name": "Placed on Hold",

"uuid": "UDPM-601",

"owner": "UDPM-6",

"managers": "UDPM-61",

"performers": "UDPM-49",

"duration": 60.0,

"tools": [

{

"name": "Email Clinician to Complete PhenoTips",

"uuid": "UDPM-1525",

"input_type": "UDPM-82",

"output_type": "UDPM-82",

"flags": 6,

"after_code": "patient = subj.get_value('Patient')\natt = patient.get_value('Attending Physician')\npc = patient.get_value('Primary Clinician')\nattSign = patient.get_value('Phenotips Attending Sign Off')\npcSign = patient.get_value('Phenotips Primary Clinician Sign Off')\n\nif pc.present? and not pcSign.present?\n send_email(pc, find_email_template(\"Phenotips Sign-off Needed for Sequencing Submission\"), patient)\nend \nif att.present? and not attSign.present?\n send_email(att, find_email_template(\"Phenotips Sign-off Needed for Sequencing Submission\"), patient)\nend "

}

],

"next_states": [

{

"state_name": "Review Case for Exome & SNP",

"state_uuid": "UDPM-305",

"btn_text": "Reconsider Case",

"btn_css": "#456",

"after_code": "next_state_performer = User.find_by_username('[redacted]')\n"

}

],

"show_options": {

"top": 275.73333740234375,

"left": 164.98333740234375

}

},

{

"name": "Returned from Queue",

"uuid": "UDPM-1103",

"owner": "UDPM-6",

"managers": "UDPM-61",

"performers": "UDPM-2",

"tools": [

{

"name": "Add Gene List",

"uuid": "UDPM-1510",

"input_type": "UDPM-82",

"output_type": "UDPM-256",

"flags": 0,

"options": {

"obj_type": "SubjectType"

}

}

],

"next_states": [

{

"state_name": "Added to Queue for Submission",

"state_uuid": "UDPM-636",

"btn_text": "Place Back into Queue",

"btn_css": "rgb(13, 73, 123)",

"btn_scale": "large"

}

],

"em_owner_done_job": true,

"em_owner_pastdue_job": true,

"show_options": {

"top": 406.9666748046875,

"left": 364.98333740234375

}

},

{

"name": "Review Case for Exome & SNP",

"uuid": "UDPM-305",

"owner": "UDPM-6",

"managers": "UDPM-61",

"performers": "UDPM-49",

"tools": [

{

"name": "Email Clinician to Complete PhenoTips",

"uuid": "UDPM-1524",

"input_type": "UDPM-82",

"output_type": "UDPM-82",

"flags": 6,

"after_code": "patient = subj.get_value('Patient')\natt = patient.get_value('Attending Physician')\npc = patient.get_value('Primary Clinician')\nattSign = patient.get_value('Phenotips Attending Sign Off')\npcSign = patient.get_value('Phenotips Primary Clinician Sign Off')\n\nif pc.present? and not pcSign.present?\n send_email(pc, find_email_template(\"Phenotips Sign-off Needed for Sequencing Submission\"), patient)\nend \nif att.present? and not attSign.present?\n send_email(att, find_email_template(\"Phenotips Sign-off Needed for Sequencing Submission\"), patient)\nend "

},

{

"name": "Add Gene List",

"uuid": "UDPM-1361",

"input_type": "UDPM-82",

"output_type": "UDPM-256",

"flags": 0,

"options": {

"obj_type": "SubjectType"

}

},

{

"name": "Edit Task File",

"uuid": "UDPM-369",

"input_type": "UDPM-82",

"output_type": "UDPM-82",

"flags": 1,

"options": {

"udfs": [

"UDPM-325",

"UDPM-317",

"UDPM-356",

"UDPM-357",

"UDPM-2914",

"UDPM-1673",

"UDPM-1674",

"UDPM-2306",

"UDPM-342",

"UDPM-321"

]

}

}

],

"next_states": [

{

"state_name": "Case Closed",

"state_uuid": "UDPM-448",

"btn_text": "Close Case",

"btn_css": "#456"

},

{

"state_name": "Placed on Hold",

"state_uuid": "UDPM-601",

"btn_text": "Place Case on Hold",

"btn_css": "#456",

"after_code": "#next_state_performer = User.find_by_username('[redacted]')\np = subj.get_value('Patient')\natt = p.get_value('Attending Physician')\n if p\n recipients = []\n physician = p.get_value('Attending Physician')\n recipients << physician if physician\n end\n\nif recipients.any?\n self.next_state_performer = att\nelse\n next_state_performer = User.find_by_username('[redacted]')\nend"

},

{

"state_name": "Added to Queue for Submission",

"state_uuid": "UDPM-636",

"btn_text": "Add to Queue",

"btn_css": "#456",

"after_code": "patient = subj.get_value('Patient')\n\nif patient \n \tRace = patient.get_value('Race')\n Ethnicity = patient.get_value('Ethnicity')\n if (not Race or not Ethnicity ) and (not params['Race'] and not params['Ethnicity'])\n raise(\"Patient can't be added to the queue without Race and Ethnicity information\")\n elsif (not Race and not Ethnicity) and (params['Race'] and not params['Ethnicity'])\n raise(\"Patient can't be added to the queue without Ethnicity information\")\n patient.set_value('Race', params['Race'])\n elsif (not Race and not Ethnicity) and (params['Ethnicity'] and not params['Race'])\n raise(\"Patient can't be added to the queue without Race information\")\n patient.set_value('Ethnicity', params['Ethnicity'])\n elsif (not Race and Ethnicity ) and params['Race']\n patient.set_value('Race', params['Race'])\n elsif (Race and not Ethnicity ) and params['Ethnicity']\n patient.set_value('Ethnicity', params['Ethnicity'])\n elsif (not Race and not Ethnicity ) and (params['Race'] and params['Ethnicity'])\n patient.set_value('Race', params['Race'])\n patient.set_value('Ethnicity', params['Ethnicity'])\n end\nend\n\nsnpToSend = subj.get_value('SNPs To Send')\nexomeToSend = subj.get_value('Exomes To Send')\nsnpToSend.each do |snpPat|\n snpNumber = 2\n name = snpPat.to_s + ' SNP Chip Sequencing'\n snpSeq = find_subject( :subject_type=>'SNP Chip Sequencing', :name=>name)\n if snpSeq.present?\n name = name + ' ' + snpNumber.to_s\n snpSeq = find_subject( :subject_type=>'SNP Chip Sequencing', :name=>name)\n while snpSeq.present?\n snpNumber = snpNumber + 1 \n name = name.to_s[0..-2] + snpNumber.to_s\n #raise name\n snpSeq = find_subject( :subject_type=>'SNP Chip Sequencing', :name=>name)\n end\n newanalysis = create_subject('SNP Chip Sequencing', {:name=>name})\n newanalysis.set_value('Patient', snpPat)\n advance_workflow('SNP Chip Sequencing', 'SNP Chip Wait List', newanalysis)\n else\n newanalysis = create_subject('SNP Chip Sequencing', {:name=>name})\n newanalysis.set_value('Patient', snpPat)\n advance_workflow('SNP Chip Sequencing', 'SNP Chip Wait List', newanalysis)\n end\nend \n\nexomeToSend.each do |exPat|\n exNumber = 2\n name = exPat.to_s + ' Exome Sequencing'\n exSeq = find_subject( :subject_type=>'Exome Sequencing', :name=>name)\n if exSeq.present?\n name = name + ' ' + exNumber.to_s\n exSeq = find_subject( :subject_type=>'Exome Sequencing', :name=>name)\n while exSeq.present?\n exNumber = exNumber + 1 \n name = name.to_s[0..-2] + exNumber.to_s\n exSeq = find_subject( :subject_type=>'Exome Sequencing', :name=>name)\n end\n newanalysis = create_subject('Exome Sequencing', {:name=>name})\n newanalysis.set_value('Patient', exPat)\n advance_workflow('Exome Sequencing', 'Exome Wait List', newanalysis)\n else\n newanalysis = create_subject('Exome Sequencing', {:name=>name})\n newanalysis.set_value('Patient', exPat)\n advance_workflow('Exome Sequencing', 'Exome Wait List', newanalysis)\n end\nend \n\n#msgFinal = \"SNP Sequencings have been requested for: #{snpToSend.join(',')} <br> Exome Sequencings have been requested for: #{exomeToSend.join(',')}\"\n#show_message(msgFinal)",

"before_code": "patient = subj.get_value('Patient')\n\nrecipients = []\nrecipients << find_user_group('Phenotips Curators')\n\natt = patient.get_value('Attending Physician')\npc = patient.get_value('Primary Clinician')\nattSign = patient.get_value('PhenoTips Attending Sign Off')\npcSign = patient.get_value('PhenoTips Primary Clinician Sign Off')\nphenoReview = patient.get_value('PhenoTips Review')\n\nif not phenoReview.present?\n newPhenoReview = create_subject('PhenoTips Review')\n newPhenoReview.set_value('Patient',patient)\n if attSign.present? ^ pcSign.present?\n advance_workflow(\"PhenoTips Review\",\"First Review by Curators\",newPhenoReview)\n send_email(recipients, find_email_template(\"Ready for First Round of Curation\"), newPhenoReview)\n else \n start_workflow('PhenoTips Review', newPhenoReview)\n if pc.present? and not pcSign.present?\n \tsend_email(pc, find_email_template(\"PhenoTips Sign-off Needed for Sequencing Submission\"), patient)\n end \n if att.present? and not attSign.present?\n \tsend_email(att, find_email_template(\"PhenoTips Sign-off Needed for Sequencing Submission\"), patient)\n end \n end \nelsif phenoReview.present?\n if phenoReview.length < 2\n review = phenoReview.first\n state = review.states.map(&:name).last\n if attSign.present? ^ pcSign.present?\n if state == 'Initial Curation by Joie' \n elsif state == 'Review by Clinical Team'\n advance_workflow(\"PhenoTips Review\",\"First Review by Curators\",review)\n send_email(recipients, find_email_template(\"Ready for First Round of Curation\"), newPhenoReview)\n end\n elsif state == 'Initial Curation by [redacted]'\n else \n if pc.present? and not pcSign.present?\n \tsend_email(pc, find_email_template(\"PhenoTips Sign-off Needed for Sequencing Submission\"), patient)\n end \n if att.present? and not attSign.present?\n \tsend_email(att, find_email_template(\"PhenoTips Sign-off Needed for Sequencing Submission\"), patient)\n end \n end \n end\nend \n\n\nsnpToSend = subj.get_value('SNPs To Send')\nexomeToSend = subj.get_value('Exomes To Send')\nsnpToSend.each do |snpPat|\n phenotips = 0\n patient = find_subject( :subject_type=>'Patient', :name=>snpPat.to_s)\n consent = patient.get_value('Consents')\n dna = patient.get_value('Number of DNA Vials')\n proband = patient.get_value('Proband')\n attSign = patient.get_value('PhenoTips Attending Sign Off')\n pcSign = patient.get_value('PhenoTips Primary Clinician Sign Off')\n \n if proband == 'Yes'\n if attSign.present? or pcSign.present?\n phenotips = phenotips + 1\n end\n if not dna.present? and not consent.present? and phenotips < 1\n raise \"The Proband's consent forms are not documneted in UDPICS, there are no DNA samples available for the Proband, and a clinician has not signed off on the Proband's PhenoTips. The family cannot be added to the Queue.\" \n elsif not consent.present? and dna.present? and phenotips < 1\n raise \"The Proband's consent forms are not documneted in UDPICS and a clinician has not signed off on the Proband's PhenoTips. The family cannot be added to the Queue. \"\n elsif not consent.present? and dna.present? and phenotips > 0\n raise \"The Proband's consent forms are not documneted in UDPICS. The family cannot be added to the Queue. \"\n elsif consent.present? and not dna.present? and phenotips < 1\n raise \"There are no DNA samples available for the Proband and a clinician has not signed off on the Proband's PhenoTips. The family cannot be added to the Queue.\"\n elsif consent.present? and not dna.present? and phenotips > 0\n raise \"There are no DNA samples available for the Proband. The family cannot be added to the Queue.\"\n elsif consent.present? and dna.present? and phenotips < 1\n raise \"The proband's phenotype has not been confirmed by the Attending or the Primary Clinician. A clinican must sign off on the proband's PhenoTips before being added to the Queue.\"\n end\n else \n if not dna.present? and not consent.present? \n raise \"Consent forms for the family members to send are not documneted in UDPICS and not all family members to send have DNA samples available. The family cannot be added to the Queue.\" \n elsif not consent.present? and dna.present?\n raise \"Consent forms for the family members to send are not documneted in UDPICS. The family cannot be added to the Queue.\"\n elsif consent.present? and not dna.present?\n raise \"Not all family members to send have DNA samples available. The family cannot be added to the Queue.\"\n end \n end \nend \n\nexomeToSend.each do |exPat|\n exomeLen = exomeToSend.length\n phenotips = 0\n patient = find_subject( :subject_type=>'Patient', :name=>exPat.to_s)\n consent = patient.get_value('Consents')\n dna = patient.get_value('Number of DNA Vials')\n proband = patient.get_value('Proband')\n attSign = patient.get_value('PhenoTips Attending Sign Off')\n pcSign = patient.get_value('PhenoTips Primary Clinician Sign Off')\n \n if proband == 'Yes'\n if attSign.present? or pcSign.present?\n phenotips = phenotips + 1\n end\n if not dna.present? and not consent.present? and phenotips < 1\n raise \"The Proband's consent forms are not documneted in UDPICS, there are no DNA samples available for the Proband, and a clinician has not signed off on the Proband's PhenoTips. The family cannot be added to the Queue.\" \n elsif not consent.present? and dna.present? and phenotips < 1\n raise \"The Proband's consent forms are not documneted in UDPICS and a clinician has not signed off on the Proband's PhenoTips. The family cannot be added to the Queue. \"\n elsif not consent.present? and dna.present? and phenotips > 0\n raise \"The Proband's consent forms are not documneted in UDPICS. The family cannot be added to the Queue. \"\n elsif consent.present? and not dna.present? and phenotips < 1\n raise \"There are no DNA samples available for the Proband and a clinician has not signed off on the Proband's PhenoTips. The family cannot be added to the Queue.\"\n elsif consent.present? and not dna.present? and phenotips > 0\n raise \"There are no DNA samples available for the Proband. The family cannot be added to the Queue.\"\n elsif consent.present? and dna.present? and phenotips < 1\n raise \"The proband's phenotype has not been confirmed by the Attending or the Primary Clinician. A clinican must sign off on the proband's PhenoTips before being added to the Queue.\"\n end\n else \n if not dna.present? and not consent.present? \n raise \"Consent forms for the family members to send are not documneted in UDPICS and not all family members to send have DNA samples available. The family cannot be added to the Queue.\" \n elsif not consent.present? and dna.present?\n raise \"Consent forms for the family members to send are not documneted in UDPICS. The family cannot be added to the Queue.\"\n elsif consent.present? and not dna.present?\n raise \"Not all family members to send have DNA samples available. The family cannot be added to the Queue.\"\n end \n end \nend\n\npatient = subj.get_value('Patient')\nif patient \n \tRace = patient.get_value('Race')\n Ethnicity = patient.get_value('Ethnicity')\n Attending = patient.get_value('Attending Physician')\n \n if not Race and not Ethnicity\n msg = \"Race and Ethicity information are not found in the patient data. Both are required for Exome analysis and dbGaP submission. Please try to enter them. <br> You may not advance this process to the next state<br> <b> Attending Physician: #{Attending}</b>\"\n params[:tool_message] = msg \n \n elsif Race and not Ethnicity\n msg = \"<b>Patient race : #{Race}</b> <br> Ethicity information is not found in the patient data. Ethnicity is required for Exome analysis and dbGaP submission. Please try to enter it. <br> You may not advance this process to the next state without Ethnicity data <br> <b> Attending Physician: #{Attending}</b>\"\n params[:tool_message] = msg \n params[:skip_udf] = {\n 'Race' =>true\n }\n elsif Ethnicity and not Race\n msg = \"<b>Patient Ethnicity : #{Ethnicity}</b> <br>Race information is not found in the patient data. Race is required for Exome analysis and dbGaP submission. Please try to enter it. <br> You may not advance this process to the next state without Race data <br> <b> Attending Physician: #{Attending}</b>\"\n params[:tool_message] = msg \n params[:skip_udf] = {\n 'Ethnicity' =>true\n }\n elsif Race and Ethnicity\n params[:skip_udf] = {\n 'Ethnicity' =>true,\n 'Race'=>true\n }\n end\nend",

"btn_scale": "large",

"after_code_params": [

"UDPM-2202",

"UDPM-562"

]

}

],

"show_options": {

"top": 25,

"left": 50

}

}

],

"entry_point": "UDPM-305",

"show_udfs": "UDPM-325,UDPM-356,UDPM-357,UDPM-317,UDPM-2914"

},{

"name": "Consent Documentation",

"uuid": "UDPM-126",

"subject_type": "UDPM-141",

"state_defs": [

{

"name": "Consent Documentation",

"uuid": "UDPM-582",

"owner": "UDPM-67",

"managers": "UDPM-9",

"performers": "UDPM-10",

"duration": 10.0,

"show_options": {

"top": 50,

"left": 90.99996948242188

},

"end_task": true

}

],

"entry_point": "UDPM-582",

"show_udfs": "UDPM-2583,UDPM-2753,UDPM-2588,UDPM-182,UDPM-1311"

},{

"name": "RNA Workflow",

"uuid": "UDPM-30",

"subject_type": "UDPM-43",

"state_defs": [

{

"name": "Archive RNA",

"uuid": "UDPM-422",

"owner": "UDPM-67",

"managers": "UDPM-22",

"performers": "UDPM-24",

"show_options": {

"top": 297,

"left": 447

},

"end_task": true

},

{

"name": "Discard",

"uuid": "UDPM-641",

"owner": "UDPM-67",

"managers": "UDPM-22",

"performers": "UDPM-24",

"show_options": {

"top": 107,

"left": 414

},

"end_task": true

},

{

"name": "For Archive",

"uuid": "UDPM-1107",

"owner": "UDPM-57",

"managers": "UDPM-8",

"performers": "UDPM-8",

"show_options": {

"top": 180,

"left": 673

}

},

{

"name": "Process Sample",

"uuid": "UDPM-107",

"owner": "UDPM-67",

"managers": "UDPM-22",

"performers": "UDPM-4",

"duration": 1.0,

"tools": [

{

"name": "Extraction Information",

"uuid": "UDPM-1358",

"input_type": "UDPM-43",

"output_type": "UDPM-43",

"description": "From cell line",

"flags": 1,

"hide_button_if_not_condition": true,

"conditions": [

{

"name": "RNA Extraction&rarr;Specimen Type",

"condition": "=",

"value": "RNA->Blood",

"message": "",

"subject_type": "UDPM-43",

"udf": "UDPM-1298"

}

],

"options": {

"udfs": [

"UDPM-2757",

"UDPM-1298",

"UDPM-1048",

"UDPM-1043",

"UDPM-503"

]

}

},

{

"name": "Processing Information",

"uuid": "UDPM-555",

"input_type": "UDPM-43",

"output_type": "UDPM-43",

"description": "From blood",

"flags": 1,

"hide_button_if_not_condition": true,

"conditions": [

{

"name": "RNA Extraction&rarr;Specimen Type",

"condition": "=",

"value": "Cell Line",

"message": "",

"subject_type": "UDPM-43",

"udf": "UDPM-1298"

}

],

"options": {

"udfs": [

"UDPM-1298",

"UDPM-1017",

"UDPM-1040",

"UDPM-1302",

"UDPM-1048",

"UDPM-1043"

]

}

},

{

"name": "Quantification",

"uuid": "UDPM-556",

"input_type": "UDPM-43",

"output_type": "UDPM-43",

"flags": 1,

"options": {

"udfs": [

"UDPM-1331",

"UDPM-1016",

"UDPM-627",

"UDPM-628",

"UDPM-2759",

"UDPM-2763",

"UDPM-452"

]

}

}

],

"next_states": [

{

"state_name": "Discard",

"state_uuid": "UDPM-641",

"btn_text": "Discard RNA",

"btn_css": "#456",

"after_code": "subj.set_value('Rationale/Reasoning',params['Rationale/Reasoning'])",

"btn_scale": "large",

"after_code_params": [

"UDPM-883"

]

},

{

"state_name": "Archive RNA",

"state_uuid": "UDPM-422",

"btn_text": "Archive RNA",

"btn_css": "#456",

"after_code": "require_script 'lims_helper'\n\nvial_count = params['Number of Vials to Store']\nif User.curr_user.user_groups.map(&:name).join(',').include? \"Lab Personnel - Building 50\"\n submit_to_fpro(subj, 'RNA', '7000000482', vial_count) do |s|\n\ts.set_value('OD 230', subj.get_value('OD 230'))\n s.set_value('OD 260', subj.get_value('OD 260'))\n s.set_value('OD 280', subj.get_value('OD 280'))\n s.set_value('Concentration (ug/ml)', subj.get_value('Concentration (ug/ml)'))\n s.set_value('RIN Score', subj.get_value('RIN Score'))\n end\nelsif User.curr_user.user_groups.map(&:name).join(',').include? \"Lab Personnel - Twinbrook\"\n submit_to_fpro(subj, 'RNA', '7000000701', vial_count) do |s|\n\ts.set_value('OD 230', subj.get_value('OD 230'))\n s.set_value('OD 260', subj.get_value('OD 260'))\n s.set_value('OD 280', subj.get_value('OD 280'))\n s.set_value('Concentration (ug/ml)', subj.get_value('Concentration (ug/ml)'))\n s.set_value('RIN Score', subj.get_value('RIN Score'))\n end\nend",

"btn_scale": "large",

"after_code_params": [

"UDPM-1450"

],

"conditions": [

{

"name": "RNA Extraction&rarr;Concentration (ug/ml)",

"condition": "Not Empty",

"value": "",

"message": "Recording of Concentration, OD 230, OD 260, OD 280, and Volume are required to advance",

"subject_type": "UDPM-43",

"udf": "UDPM-1331"

},

{

"name": "RNA Extraction&rarr;OD 230",

"condition": "Not Empty",

"value": "",

"message": "Recording of Concentration, OD 230, OD 260, OD 280, and Volume are required to advance",

"subject_type": "UDPM-43",

"udf": "UDPM-1016"

},

{

"name": "RNA Extraction&rarr;OD 260",

"condition": "Not Empty",

"value": "",

"message": "Recording of Concentration, OD 230, OD 260, OD 280, and Volume are required to advance",

"subject_type": "UDPM-43",

"udf": "UDPM-627"

},

{

"name": "RNA Extraction&rarr;OD 280",

"condition": "Not Empty",

"value": "",

"message": "Recording of Concentration, OD 230, OD 260, OD 280, and Volume are required to advance",

"subject_type": "UDPM-43",

"udf": "UDPM-628"

},

{

"name": "RNA Extraction&rarr;Volume (uL)",

"condition": "Not Empty",

"value": "",

"message": "Recording of Concentration, OD 230, OD 260, OD 280, and Volume are required to advance",

"subject_type": "UDPM-43",

"udf": "UDPM-452"

}

]

}

],

"show_options": {

"top": 229,

"left": 89

}

}

],

"entry_point": "UDPM-107",

"ask_performer": true,

"show_udfs": "UDPM-2415,UDPM-2757,UDPM-449,UDPM-2943"

},{

"name": "Information Technology Transfer Workflow",

"uuid": "UDPM-153",

"subject_type": "UDPM-183",

"state_defs": [

{

"name": "Cancel",

"uuid": "UDPM-1002",

"owner": "UDPM-67",

"managers": "UDPM-27",

"performers": "UDPM-3",

"show_options": {

"top": 138,

"left": 626

},

"end_task": true

},

{

"name": "Collaboration Completed",

"uuid": "UDPM-716",

"owner": "UDPM-67",

"managers": "UDPM-27",

"performers": "UDPM-3",

"show_options": {

"top": 278,

"left": 349

},

"end_task": true

},

{

"name": "Ship Hard Drive",

"uuid": "UDPM-894",

"owner": "UDPM-67",

"managers": "UDPM-27",

"performers": "UDPM-46",

"tools": [

{

"name": "Shipment Information",

"uuid": "UDPM-1147",

"input_type": "UDPM-183",

"output_type": "UDPM-183",

"flags": 1,

"options": {

"udfs": [

"UDPM-309",

"UDPM-1797",

"UDPM-121"

]

}

}

],

"next_states": [

{

"state_name": "Waiting for Return Shipments",

"state_uuid": "UDPM-1001",

"btn_text": "Hard Drive Shipped",

"btn_css": "#456"

}

],

"show_options": {

"top": 253.9857635498047,

"left": 74.99429321289062

}

},

{

"name": "Transfer Information",

"uuid": "UDPM-714",

"owner": "UDPM-67",

"managers": "UDPM-27",

"performers": "UDPM-3",

"tools": [

{

"name": "File for Collaboration",

"uuid": "UDPM-879",

"input_type": "UDPM-183",

"output_type": "UDPM-184",

"flags": 0,

"options": {

"obj_type": "SubjectType",

"do_not_open_subject": true

}

},

{

"name": "Additional Information",

"uuid": "UDPM-992",

"input_type": "UDPM-183",

"output_type": "UDPM-183",

"flags": 1,

"options": {

"udfs": [

"UDPM-1417",

"UDPM-1393",

"UDPM-2141",

"UDPM-1699",

"UDPM-1861",

"UDPM-309",

"UDPM-1797",

"UDPM-2374",

"UDPM-2779"

]

}

},

{

"name": "File Received from Collaboration",

"uuid": "UDPM-1253",

"input_type": "UDPM-183",

"output_type": "UDPM-235",

"flags": 0,

"options": {

"obj_type": "SubjectType",

"do_not_open_subject": true

}

}

],

"next_states": [

{

"state_name": "Collaboration Completed",

"state_uuid": "UDPM-716",

"btn_text": "Data Transferred",

"btn_css": "#456"

},

{

"state_name": "Ship Hard Drive",

"state_uuid": "UDPM-894",

"btn_text": "Prepare Hard Drive for Shipment",

"btn_css": "#456",

"btn_scale": "large"

},

{

"state_name": "Cancel",

"state_uuid": "UDPM-1002",

"btn_text": "Cancel Collaboration",

"btn_css": "#456"

}

],

"em_performer_new_job": true,

"show_options": {

"top": 42,

"left": 44

}

},

{

"name": "Waiting for Return Shipments",

"uuid": "UDPM-1001",

"owner": "UDPM-67",

"managers": "UDPM-27",

"performers": "UDPM-3",

"tools": [

{

"name": "Files Received from Collaboration",

"uuid": "UDPM-1410",

"input_type": "UDPM-183",

"output_type": "UDPM-235",

"flags": 0,

"options": {

"obj_type": "SubjectType",

"do_not_open_subject": true

}

}

],

"next_states": [

{

"state_name": "Cancel",

"state_uuid": "UDPM-1002",

"btn_text": "Cancel Collaboration",

"btn_css": "#456"

},

{

"state_name": "Collaboration Completed",

"state_uuid": "UDPM-716",

"btn_text": "All Files Received by UDP",

"btn_css": "#456"

}

],

"show_options": {

"top": 582,

"left": 551

}

}

],

"entry_point": "UDPM-714",

"ask_performer": true,

"show_udfs": "UDPM-6,UDPM-309,UDPM-1393,UDPM-1797,UDPM-1699"

},{

"name": "Research Sample",

"uuid": "UDPM-88",

"subject_type": "UDPM-86",

"state_defs": [

{

"name": "Archive Sample",

"uuid": "UDPM-581",

"owner": "UDPM-67",

"managers": "UDPM-22",

"performers": "UDPM-69",

"show_options": {

"top": 36,

"left": 938

},

"end_task": true

},

{

"name": "Collected",

"uuid": "UDPM-357",

"owner": "UDPM-67",

"managers": "UDPM-8",

"performers": "UDPM-7",

"duration": 1.0,

"tools": [

{

"name": "Print Barcode",

"uuid": "UDPM-401",

"input_type": "UDPM-86",

"output_type": "UDPM-86",

"flags": 2,

"options": {

"obj_type": "Printer",

"labels_count": 1,

"obj_name": "UDPM-5"

}

},

{

"name": "Additional Information",

"uuid": "UDPM-672",

"input_type": "UDPM-86",

"output_type": "UDPM-86",

"description": "Blood",

"flags": 1,

"hide_button_if_not_condition": true,

"conditions": [

{

"name": "Research Sample&rarr;Research Sample Type",

"condition": "=",

"value": "Blood",

"subject_type": "UDPM-86",

"udf": "UDPM-1228"

}

],

"options": {

"udfs": [

"UDPM-1832",

"UDPM-470",

"UDPM-1299",

"UDPM-1310",

"UDPM-1301"

]

}

},

{

"name": "Additional Information",

"uuid": "UDPM-673",

"input_type": "UDPM-86",

"output_type": "UDPM-86",

"description": "Urine",

"flags": 1,

"hide_button_if_not_condition": true,

"conditions": [

{

"name": "Research Sample&rarr;Research Sample Type",

"condition": "=",

"value": "Urine",

"subject_type": "UDPM-86",

"udf": "UDPM-1228"

}

],

"options": {

"udfs": [

"UDPM-1299",

"UDPM-1300",

"UDPM-1309",

"UDPM-1301"

]

}

},

{

"name": "Additional Information",

"uuid": "UDPM-674",

"input_type": "UDPM-86",

"output_type": "UDPM-86",

"description": "CSF",

"flags": 1,

"hide_button_if_not_condition": true,

"conditions": [

{

"name": "Research Sample&rarr;Research Sample Type",

"condition": "=",

"value": "CSF",

"subject_type": "UDPM-86",

"udf": "UDPM-1228"

}

],

"options": {

"udfs": [

"UDPM-1299",

"UDPM-1300",

"UDPM-1304",

"UDPM-1305",

"UDPM-1306",

"UDPM-1307",

"UDPM-1308",

"UDPM-1301"

]

}

}

],

"next_states": [

{

"state_name": "Discarded",

"state_uuid": "UDPM-446",

"btn_text": "Discard Sample",

"btn_css": "#456",

"after_code": "# Extra code here\n\nsubj.get_value('Rationale/Reasoning', params['Rationale/Reasoning'] )",

"after_code_params": [

"UDPM-883"

]

},

{

"state_name": "Internal Lab",

"state_uuid": "UDPM-359",

"btn_text": "Transport to Lab for Processing",

"btn_css": "#456",

"after_code": "#patient = subj.get_value('Patient')\n#medications = patient.get_value('Medications')\n#blood_kit = subj.get_value('Blood Kit')\n#if medications.count == 0 and not blood_kit.present? \n #raise \"Medication List must be completed before research samples can be processed\"\n#end\n\n\nself.next_state_performer = find_user('[redacted]')\nsend_email(User.find_by_username('[redacted]'), find_email_template(\"Research Samples Collected\"), subj)\nsend_email(User.find_by_username('[redacted]'), find_email_template(\"Research Samples Collected\"), subj)",

"before_code": "params[:ask_next_performer] = true",

"btn_scale": "large"

}

],

"em_owner_pastdue_job": true,

"show_options": {

"top": 44,

"left": 64

}

},

{

"name": "Discarded",

"uuid": "UDPM-446",

"owner": "UDPM-67",

"managers": "UDPM-22",

"performers": "UDPM-69",

"show_options": {

"top": 368.66668701171875,

"left": 373.66668701171875

},

"end_task": true

},

{

"name": "Hold/Store",

"uuid": "UDPM-584",

"owner": "UDPM-67",

"managers": "UDPM-22",

"performers": "UDPM-69",

"duration": 7.0,

"next_states": [

{

"state_name": "Internal Lab",

"state_uuid": "UDPM-359",

"btn_text": "Return to Processing",

"btn_css": "#456",

"after_code": "self.next_state_performer = User.curr_user"

}

],

"em_owner_pastdue_job": true,

"show_options": {

"top": 202.99147033691406,

"left": 931.9885559082031

}

},

{

"name": "Internal Lab",

"uuid": "UDPM-359",

"owner": "UDPM-67",

"managers": "UDPM-22",

"performers": "UDPM-69",

"duration": 1.0,

"tools": [

{

"name": "Print",

"uuid": "UDPM-402",

"input_type": "UDPM-86",

"output_type": "UDPM-86",

"description": "Research Sample Barcode Label",

"flags": 2,

"options": {

"obj_type": "Printer",

"labels_count": 1,

"obj_name": "UDPM-5"

}

},

{

"name": "Begin DNA Extraction",

"uuid": "UDPM-403",

"input_type": "UDPM-86",

"output_type": "UDPM-122",

"flags": 0,

"hide_button_if_not_condition": true,

"conditions": [

{

"name": "Research Sample&rarr;Type of Blood Tube",

"condition": "!=",

"value": "PAX",

"message": "",

"subject_type": "UDPM-86",

"udf": "UDPM-470"

},

{

"name": "Research Sample&rarr;Type of Blood Tube",

"condition": "!=",

"value": "Serum (red)",

"message": "",

"subject_type": "UDPM-86",

"udf": "UDPM-470"

},

{

"name": "Research Sample&rarr;Research Sample Type",

"condition": "=",

"value": "Blood",

"message": "",

"subject_type": "UDPM-86",

"udf": "UDPM-1228"

},

{

"name": "Research Sample&rarr;Type of Blood Tube",

"condition": "!=",

"value": "Heparin (green)",

"message": "",

"subject_type": "UDPM-86",

"udf": "UDPM-470"

},

{

"name": "Research Sample&rarr;Type of Blood Tube",

"condition": "!=",

"value": "ACD (lymphoblastoid)",

"message": "",

"subject_type": "UDPM-86",

"udf": "UDPM-470"

}

],

"options": {

"obj_type": "SubjectType"

}

},

{

"name": "Begin Plasma Extraction",

"uuid": "UDPM-404",

"input_type": "UDPM-86",

"output_type": "UDPM-47",

"flags": 0,

"hide_button_if_not_condition": true,

"conditions": [

{

"name": "Research Sample&rarr;Type of Blood Tube",

"condition": "!=",

"value": "PAX",

"subject_type": "UDPM-86",

"udf": "UDPM-470"

},

{

"name": "Research Sample&rarr;Type of Blood Tube",

"condition": "!=",

"value": "Serum (red)",

"subject_type": "UDPM-86",

"udf": "UDPM-470"

},

{

"name": "Research Sample&rarr;Research Sample Type",

"condition": "=",

"value": "Blood",

"subject_type": "UDPM-86",

"udf": "UDPM-1228"

},

{

"name": "Research Sample&rarr;Type of Blood Tube",

"condition": "!=",

"value": "ACD (lymphoblastoid)",

"message": "",

"subject_type": "UDPM-86",

"udf": "UDPM-470"

}

],

"options": {

"obj_type": "SubjectType"

}

},

{

"name": "Begin Urine Processing",

"uuid": "UDPM-566",

"input_type": "UDPM-86",

"output_type": "UDPM-120",

"flags": 0,

"hide_button_if_not_condition": true,

"conditions": [

{

"name": "Research Sample&rarr;Research Sample Type",

"condition": "=",

"value": "Urine",

"subject_type": "UDPM-86",

"udf": "UDPM-1228"

}

],

"options": {

"obj_type": "SubjectType"

}

},

{

"name": "Begin Serum Extraction",

"uuid": "UDPM-567",

"input_type": "UDPM-86",

"output_type": "UDPM-119",

"flags": 0,

"before_code": "params[:defaults] = {\n 'Specimen Type' => \"Serum->Blood\"\n}",

"hide_button_if_not_condition": true,

"conditions": [

{

"name": "Research Sample&rarr;Type of Blood Tube",

"condition": "=",

"value": "Serum (red)",

"subject_type": "UDPM-86",

"udf": "UDPM-470"

}

],

"options": {

"obj_type": "SubjectType"

}

},

{

"name": "Begin CSF Processing",

"uuid": "UDPM-568",

"input_type": "UDPM-86",

"output_type": "UDPM-117",

"flags": 0,

"hide_button_if_not_condition": true,

"conditions": [

{

"name": "Research Sample&rarr;Research Sample Type",

"condition": "=",

"value": "CSF",

"subject_type": "UDPM-86",

"udf": "UDPM-1228"

}

],

"options": {

"obj_type": "SubjectType"

}

},

{

"name": "Begin RNA Extraction",

"uuid": "UDPM-577",

"input_type": "UDPM-86",

"output_type": "UDPM-43",

"flags": 0,

"hide_button_if_not_condition": true,

"conditions": [

{

"name": "Research Sample&rarr;Type of Blood Tube",

"condition": "=",

"value": "PAX",

"subject_type": "UDPM-86",

"udf": "UDPM-470"

}

],

"options": {

"obj_type": "SubjectType"

}

},

{

"name": "Receive Neurotransmitter",

"uuid": "UDPM-822",

"input_type": "UDPM-86",

"output_type": "UDPM-169",

"flags": 0,

"hide_button_if_not_condition": true,

"conditions": [

{

"name": "Research Sample&rarr;Research Sample Type",

"condition": "!=",

"value": "Blood",

"message": "",

"subject_type": "UDPM-86",

"udf": "UDPM-1228"

},

{

"name": "Research Sample&rarr;Research Sample Type",

"condition": "!=",

"value": "Skin Biopsy",

"message": "",

"subject_type": "UDPM-86",

"udf": "UDPM-1228"

},

{

"name": "Research Sample&rarr;Research Sample Type",

"condition": "!=",

"value": "Urine",

"message": "",

"subject_type": "UDPM-86",

"udf": "UDPM-1228"

},

{

"name": "Research Sample&rarr;Research Sample Type",

"condition": "!=",

"value": "Other",

"message": "",

"subject_type": "UDPM-86",

"udf": "UDPM-1228"

}

],

"options": {

"obj_type": "SubjectType"

}

},

{

"name": "Process Urine Pellet",

"uuid": "UDPM-823",

"input_type": "UDPM-86",

"output_type": "UDPM-170",

"flags": 0,

"hide_button_if_not_condition": true,

"conditions": [

{

"name": "Research Sample&rarr;Research Sample Type",

"condition": "=",

"value": "Urine",

"message": "",

"subject_type": "UDPM-86",

"udf": "UDPM-1228"

}

],

"options": {

"obj_type": "SubjectType"

}

},

{

"name": "Begin Buffy Coat Isolation",

"uuid": "UDPM-1194",

"input_type": "UDPM-86",

"output_type": "UDPM-219",

"flags": 0,

"hide_button_if_not_condition": true,

"conditions": [

{

"name": "Research Sample&rarr;Type of Blood Tube",

"condition": "!=",

"value": "PAX",

"message": "",

"subject_type": "UDPM-86",

"udf": "UDPM-470"

},

{

"name": "Research Sample&rarr;Type of Blood Tube",

"condition": "!=",

"value": "Serum (red)",

"message": "",

"subject_type": "UDPM-86",

"udf": "UDPM-470"

},

{

"name": "Research Sample&rarr;Research Sample Type",

"condition": "=",

"value": "Blood",

"message": "",

"subject_type": "UDPM-86",

"udf": "UDPM-1228"

},

{

"name": "Research Sample&rarr;Type of Blood Tube",

"condition": "!=",

"value": "ACD (lymphoblastoid)",

"message": "",

"subject_type": "UDPM-86",

"udf": "UDPM-470"

},

{

"name": "Research Sample&rarr;Type of Blood Tube",

"condition": "!=",

"value": "Heparin (green)",

"message": "",

"subject_type": "UDPM-86",

"udf": "UDPM-470"

}

],

"options": {

"obj_type": "SubjectType"

}

},

{

"name": "Begin Erythrocyte Isolation",

"uuid": "UDPM-1195",

"input_type": "UDPM-86",

"output_type": "UDPM-227",

"flags": 0,

"hide_button_if_not_condition": true,

"conditions": [

{

"name": "Research Sample&rarr;Type of Blood Tube",

"condition": "!=",

"value": "PAX",

"message": "",

"subject_type": "UDPM-86",

"udf": "UDPM-470"

},

{

"name": "Research Sample&rarr;Type of Blood Tube",

"condition": "!=",

"value": "Serum (red)",

"message": "",

"subject_type": "UDPM-86",

"udf": "UDPM-470"

},

{

"name": "Research Sample&rarr;Research Sample Type",

"condition": "=",

"value": "Blood",

"message": "",

"subject_type": "UDPM-86",

"udf": "UDPM-1228"

},

{

"name": "Research Sample&rarr;Type of Blood Tube",

"condition": "!=",

"value": "ACD (lymphoblastoid)",

"message": "",

"subject_type": "UDPM-86",

"udf": "UDPM-470"

},

{

"name": "Research Sample&rarr;Type of Blood Tube",

"condition": "!=",

"value": "Heparin (green)",

"message": "",

"subject_type": "UDPM-86",

"udf": "UDPM-470"

}

],

"options": {

"obj_type": "SubjectType"

}

},

{

"name": "Begin Platelet Isolation",

"uuid": "UDPM-1196",

"input_type": "UDPM-86",

"output_type": "UDPM-220",

"flags": 0,

"hide_button_if_not_condition": true,

"conditions": [

{

"name": "Research Sample&rarr;Platelet Isolation Requested",

"condition": "=",

"value": "Yes",

"message": "",

"subject_type": "UDPM-86",

"udf": "UDPM-2483"

}

],

"options": {

"obj_type": "SubjectType"

}

}

],

"next_states": [

{

"state_name": "Processed",

"state_uuid": "UDPM-366",

"btn_text": "Processing Complete",

"btn_css": "#456",

"hide_button_if_not_condition": true,

"conditions": [

{

"name": "Research Sample&rarr;Type of Blood Tube",

"condition": "!=",

"value": "ACD (lymphoblastoid)",

"message": "",

"subject_type": "UDPM-86",

"udf": "UDPM-470"

}

]

},

{

"state_name": "Archive Sample",

"state_uuid": "UDPM-581",

"btn_text": "Archive Sample",

"btn_css": "#456",

"after_code": "subj.set_value('Protocol Used', params['Protocol Used'])",

"btn_scale": "large",

"after_code_params": [

"UDPM-2775"

],

"hide_button_if_not_condition": true,

"conditions": [

{

"name": "Research Sample&rarr;Research Sample Type",

"condition": "=",

"value": "Other",

"subject_type": "UDPM-86",

"udf": "UDPM-1228"

}

]

},

{

"state_name": "Discarded",

"state_uuid": "UDPM-446",

"btn_text": "Discard Sample",

"btn_css": "#456",

"after_code": "# Extra code here\n\nsubj.set_value('Rationale/Reasoning', params['Rationale/Reasoning'] )",

"after_code_params": [

"UDPM-883"

]

},

{

"state_name": "Hold/Store",

"state_uuid": "UDPM-584",

"btn_text": "Hold/Store",

"btn_css": "#456",

"after_code": "self.next_state_performer = User.curr_user\n\nsubj.set_value('Temporary Storage Location', params['Temporary Storage Location'])\n",

"btn_scale": "large",

"after_code_params": [

"UDPM-453"

]

},

{

"state_name": "Prepare Sample for Culture",

"state_uuid": "UDPM-605",

"btn_text": "Plate Biopsy for Fibroblasts",

"btn_css": "#456",

"after_code": "subj.set_value('Date Culture Started', params['Date Culture Started'] )\nsubj.set_value('Biobanking Protocol', params['Biobanking Protocol'] )\nself.next_state_performer = User.curr_user",

"after_code_params": [

"UDPM-1035",

"UDPM-2415"

],

"hide_button_if_not_condition": true,

"conditions": [

{

"name": "Research Sample&rarr;Research Sample Type",

"condition": "=",

"value": "Skin Biopsy",

"subject_type": "UDPM-86",

"udf": "UDPM-1228"

}

]

}

],

"em_owner_pastdue_job": true,

"show_options": {

"top": 65,

"left": 394

}

},

{

"name": "Prepare Sample for Culture",

"uuid": "UDPM-605",

"owner": "UDPM-67",

"managers": "UDPM-22",

"performers": "UDPM-69",

"duration": 50.0,

"tools": [

{

"name": "Print",

"uuid": "UDPM-752",

"input_type": "UDPM-86",

"output_type": "UDPM-86",

"description": "Barcode for Plated Biopsy",

"flags": 2,

"options": {

"obj_type": "Printer",

"labels_count": 1,

"obj_name": "UDPM-18"

}

},

{

"name": "Transfer to New Flask",

"uuid": "UDPM-751",

"input_type": "UDPM-86",

"output_type": "UDPM-40",

"description": "Passage Cells",

"flags": 0,

"options": {

"obj_type": "SubjectType"

}

}

],

"next_states": [

{

"state_name": "Discarded",

"state_uuid": "UDPM-446",

"btn_text": "Discard Sample",

"btn_css": "#456",

"after_code": "subj.set_value('Rationale/Reasoning', params['Rationale/Reasoning'] )",

"after_code_params": [

"UDPM-883"

]

},

{

"state_name": "Processed",

"state_uuid": "UDPM-366",

"btn_text": "Sample Processed",

"btn_css": "#456"

}

],

"em_owner_pastdue_job": true,

"show_options": {

"top": 424,

"left": 584.9833374023438

}

},

{

"name": "Processed",

"uuid": "UDPM-366",

"owner": "UDPM-67",

"managers": "UDPM-22",

"performers": "UDPM-69",

"show_options": {

"top": 321,

"left": 869

},

"end_task": true

}

],

"entry_point": "UDPM-357",

"show_udfs": "UDPM-1669,UDPM-421,UDPM-191,UDPM-1303,UDPM-470,UDPM-2483,UDPM-1832"

},{

"name": "Destination Vector Construction",

"uuid": "UDPM-142",

"subject_type": "UDPM-168",

"state_defs": [

{

"name": "Cancelled",

"uuid": "UDPM-969",

"owner": "UDPM-67",

"managers": "UDPM-22",

"performers": "UDPM-21",

"show_options": {

"top": 313,

"left": 306

},

"end_task": true

},

{

"name": "Plasmid Construction Complete",

"uuid": "UDPM-688",

"owner": "UDPM-67",

"managers": "UDPM-22",

"performers": "UDPM-21",

"tools": [

{

"name": "Mutagenize Vector",

"uuid": "UDPM-1515",

"input_type": "UDPM-168",

"output_type": "UDPM-228",

"flags": 0,

"options": {

"obj_type": "SubjectType"

}

},

{

"name": "Begin Rescue",

"uuid": "UDPM-1271",

"input_type": "UDPM-168",

"output_type": "UDPM-247",

"flags": 0,

"options": {

"obj_type": "SubjectType"

}

},

{

"name": "Begin Glycerol Stock",

"uuid": "UDPM-1274",

"input_type": "UDPM-168",

"output_type": "UDPM-171",

"flags": 0,

"options": {

"obj_type": "SubjectType"

}

}

],

"show_options": {

"top": 371,

"left": 525

},

"end_task": true

},

{

"name": "Recombination into Destination Vector",

"uuid": "UDPM-683",

"owner": "UDPM-67",

"managers": "UDPM-22",

"performers": "UDPM-21",

"tools": [

{

"name": "Upload AB1 File",

"uuid": "UDPM-1448",

"input_type": "UDPM-168",

"output_type": "UDPM-250",

"flags": 0,

"options": {

"obj_type": "SubjectType",

"do_not_open_subject": true

}

},

{

"name": "Prepare MiniPrep",

"uuid": "UDPM-1279",

"input_type": "UDPM-168",

"output_type": "UDPM-249",

"flags": 0,

"options": {

"obj_type": "SubjectType"

}

},

{

"name": "Prepare Glycerol Stock",

"uuid": "UDPM-898",

"input_type": "UDPM-168",

"output_type": "UDPM-171",

"flags": 0,

"options": {

"obj_type": "SubjectType"

}

},

{

"name": "Destination Vector Information",

"uuid": "UDPM-901",

"input_type": "UDPM-168",

"output_type": "UDPM-168",

"flags": 1,

"options": {

"udfs": [

"UDPM-1656",

"UDPM-6",

"UDPM-2195",

"UDPM-1840",

"UDPM-1799",

"UDPM-1932",

"UDPM-1968",

"UDPM-2496",

"UDPM-1924",

"UDPM-1925",

"UDPM-1928",

"UDPM-1698",

"UDPM-901"

]

}

},

{

"name": "MaxiPrep Information",

"uuid": "UDPM-948",

"input_type": "UDPM-168",

"output_type": "UDPM-168",

"flags": 1,

"options": {

"udfs": [

"UDPM-1977",

"UDPM-1332",

"UDPM-1016",

"UDPM-627",

"UDPM-628"

]

}

},

{

"name": "Request Primer Order",

"uuid": "UDPM-1134",

"input_type": "UDPM-168",

"output_type": "UDPM-217",

"flags": 0,

"options": {

"obj_type": "SubjectType"

}

}

],

"next_states": [

{

"state_name": "Plasmid Construction Complete",

"state_uuid": "UDPM-688",

"btn_text": "Store MaxiPrep",

"btn_css": "#456",

"after_code": "require_script 'fpro_samples'\n\nvial_count = params['Number of Vials to Store']\ndv = subj.get_value('Destination Vector')\nev = subj.get_value('Entry Vector')\nif User.curr_user.user_groups.map(&:name).join(',').include? \"Lab Personnel - Twinbrook\"\n submit_to_fpro(subj, 'MaxiPrep DNA', '7000000404', vial_count) do |s|\n s.set_value('Vector Information', dv.name)\n s.set_value('Insert Information', ev.name)\n s.set_value('Resistance Marker', dv.get_value('Resistance Marker'))\n end\nelsif User.curr_user.user_groups.map(&:name).join(',').include? \"Lab Personnel - Building 50\"\n submit_to_fpro(subj, 'MaxiPrep DNA', '7000000476', vial_count) do |s|\n s.set_value('Vector Information', dv.name)\n s.set_value('Insert Information', ev.name)\n s.set_value('Resistance Marker', dv.get_value('Resistance Marker'))\n end\nelse\n raise(\"This script only allows users who belong to either Twinbrook Lab or Building 50 Lab, please contact the administrators\")\nend",

"after_code_params": [

"UDPM-1450"

]

},

{

"state_name": "Cancelled",

"state_uuid": "UDPM-969",

"btn_text": "Cancel",

"btn_css": "#456"

}

],

"show_options": {

"top": 84.99432373046875,

"left": 141.86077880859375

}

}

],

"entry_point": "UDPM-683",

"ask_performer": true,

"show_udfs": "UDPM-6,UDPM-2496,UDPM-1968"

},{

"name": "SNP Analysis Workflow",

"uuid": "UDPM-47",

"subject_type": "UDPM-59",

"state_defs": [

{

"name": "Analysis Cancelled",

"uuid": "UDPM-640",

"owner": "UDPM-67",

"managers": "UDPM-27",

"performers": "UDPM-3",

"show_options": {

"top": 638,

"left": 90

},

"end_task": true

},

{

"name": "Analysis on Hold",

"uuid": "UDPM-600",

"owner": "UDPM-67",

"managers": "UDPM-27",

"performers": "UDPM-3",

"show_options": {

"top": 54,

"left": 643

},

"end_task": true

},

{

"name": "Analysis Queue",

"uuid": "UDPM-1069",

"owner": "UDPM-67",

"managers": "UDPM-27",

"performers": "UDPM-3",

"duration": 30.0,

"next_states": [

{

"state_name": "Analysis on Hold",

"state_uuid": "UDPM-600",

"btn_text": "Place Analysis on Hold",

"btn_css": "rgb(13, 73, 123)",

"btn_scale": "large"

},

{

"state_name": "Generate SNP Report",

"state_uuid": "UDPM-312",

"btn_text": "Begin Analysis",

"btn_css": "rgb(13, 73, 123)",

"after_code": "subj.set_value('Analyst', params['Analyst'])\nself.next_state_performer = User.curr_user",

"btn_scale": "large",

"after_code_params": [

"UDPM-1767"

]

},

{

"state_name": "Analysis Cancelled",

"state_uuid": "UDPM-640",

"btn_text": "Cancel Analysis",

"btn_css": "rgb(13, 73, 123)",

"btn_scale": "large"

}

],

"show_options": {

"top": 283,

"left": 606

}

},

{

"name": "Complete SNP Analysis",

"uuid": "UDPM-138",

"owner": "UDPM-67",

"managers": "UDPM-27",

"performers": "UDPM-3",

"tools": [

{

"name": "Additional Notes",

"uuid": "UDPM-1248",

"input_type": "UDPM-59",

"output_type": "UDPM-59",

"flags": 1,

"options": {

"udfs": [

"UDPM-191"

]

}

}

],

"show_options": {

"top": 624,

"left": 953

},

"end_task": true

},

{

"name": "Generate SNP Report",

"uuid": "UDPM-312",

"owner": "UDPM-67",

"managers": "UDPM-27",

"performers": "UDPM-3",

"duration": 7.0,

"tools": [

{

"name": "SNP Analysis Information",

"uuid": "UDPM-365",

"input_type": "UDPM-59",

"output_type": "UDPM-59",

"flags": 1,

"options": {

"udfs": [

"UDPM-1277",

"UDPM-2145",

"UDPM-404",

"UDPM-1767",

"UDPM-2135",

"UDPM-1320",

"UDPM-2045",

"UDPM-1319",

"UDPM-1318",

"UDPM-397",

"UDPM-396",

"UDPM-1214",

"UDPM-864",

"UDPM-191"

]

}

},

{

"name": "Add Sensitive Family Information",

"uuid": "UDPM-1514",

"input_type": "UDPM-59",

"output_type": "UDPM-276",

"flags": 0,

"options": {

"obj_type": "SubjectType"

}

},

{

"name": "Upload Bedfile",

"uuid": "UDPM-1228",

"input_type": "UDPM-59",

"output_type": "UDPM-238",

"flags": 0,

"options": {

"obj_type": "SubjectType",

"do_not_open_subject": true

}

},

{

"name": "Record CNV/Homozygosity",

"uuid": "UDPM-1422",

"input_type": "UDPM-59",

"output_type": "UDPM-155",

"flags": 0,

"options": {

"obj_type": "SubjectType"

}

}

],

"next_states": [

{

"state_name": "Analysis on Hold",

"state_uuid": "UDPM-600",

"btn_text": "Place Analysis on Hold",

"btn_css": "#456",

"after_code": "subj.set_value('Rationale/Reasoning', params['Rationale/Reasoning'] )",

"after_code_params": [

"UDPM-883"

]

},

{

"state_name": "Analysis Cancelled",

"state_uuid": "UDPM-640",

"btn_text": "Cancel Analysis",

"btn_css": "rgb(13, 73, 123)",

"btn_scale": "large"

},

{

"state_name": "Complete SNP Analysis",

"state_uuid": "UDPM-138",

"btn_text": "Complete SNP Analysis",

"btn_css": "#456",

"after_code": "subj.set_value('Total Amount of Homozygosity that Segregates with Disease', params['Total Amount of Homozygosity that Segregates with Disease'])\n",

"before_code": "snp_report = subj.get_value('SOP SNP Report')\nbuild = subj.get_value('Build').to_s\nif not build.present?\n raise (\"Enter build of SNP Analysis.\")\nend\nsum = 0.0\nhzregion = 0\nif snp_report.present?\n length = []\n chromEnds = []\n cnvNumber = []\n build = subj.get_value('Build').to_s\n\n snp_report.each do |report|\n cnv_region = report.get_value('CNV Region')\n homo_region = report.get_value('Homozygous Region')\n link_region = report.get_value('Linkage Region')\n if cnv_region.present? \n cnv = report.get_value('Copy Number').to_i\n if not cnv_region.to_s.downcase.starts_with?('chr') \n raise (\"Check that the CNV, Homozygosity, and Linkage Regions are in the following format: 'chr1:123456-654321'\" ) \n end \n if not cnv_region.include?(':') or not cnv_region.include?('-')\n raise (\"Check that the CNV, Homozygosity, and Linkage Regions are in the following format: 'chr1:123456-654321'\" ) \n end\n cnv_region_components=cnv_region.split(/[:-]/)\n chrom = cnv_region_components[0].delete('^0-9').to_i \n chromStart = cnv_region_components[1].delete('^0-9').to_i\n chromEnd = cnv_region_components[2].delete('^0-9').to_i\n if cnv == 2 or cnv > 4\n cnvNumber << cnv_region\n end \n if build == 'HG19'\n len = chromEnd - chromStart\n sum = sum + len\n # raise (\"#{build}\")\n if chromStart > chromEnd\n length << cnv_region\n end \n if chrom == 1 and chromEnd > 249250622\n chromEnds << cnv_region\n end\n if chrom == 2 and chromEnd > 243199373\n chromEnds << cnv_region\n end\n if chrom == 3 and chromEnd > 198022430\n chromEnds << cnv_region\n end\n if chrom == 4 and chromEnd > 191154276\n chromEnds << cnv_region\n end\n if chrom == 5 and chromEnd > 180915260\n chromEnds << cnv_region\n end\n if chrom == 6 and chromEnd > 171115067\n chromEnds << cnv_region\n end\n if chrom == 7 and chromEnd > 159138663\n chromEnds << cnv_region\n end\n if chrom == 8 and chromEnd > 146364022\n chromEnds << cnv_region\n end\n if chrom == 9 and chromEnd > 141213431\n chromEnds << cnv_region\n end\n if chrom == 10 and chromEnd > 135534747\n chromEnds << cnv_region\n end\n if chrom == 11 and chromEnd > 135006516\n chromEnds << cnv_region\n end\n if chrom == 12 and chromEnd > 133851895\n chromEnds << cnv_region\n end\n if chrom == 13 and chromEnd > 115169878\n chromEnds << cnv_region\n end\n if chrom == 14 and chromEnd > 107349540\n chromEnds << cnv_region\n end\n if chrom == 15 and chromEnd > 102531392\n chromEnds << cnv_region\n end\n if chrom == 16 and chromEnd > 90354753\n chromEnds << cnv_region\n end\n if chrom == 17 and chromEnd > 81195210\n chromEnds << cnv_region\n end\n if chrom == 18 and chromEnd > 78077248\n chromEnds << cnv_region\n end\n if chrom == 19 and chromEnd > 59128983\n chromEnds << cnv_region\n end\n if chrom == 20 and chromEnd > 63025521\n chromEnds << cnv_region\n end\n if chrom == 21 and chromEnd > 48129895\n chromEnds << cnv_region\n end\n if chrom == 22 and chromEnd > 51304567\n chromEnds << cnv_region\n end\n end \n end \n if homo_region.present? \n hzregion = hzregion + 1\n homo_region_components=homo_region.split(/[:-]/)\n chrom = homo_region_components[0].delete('^0-9').to_i \n chromStart = homo_region_components[1].to_i\n chromEnd = homo_region_components[2].to_i\n if chromStart > chromEnd\n length << homo_region\n end \n if build == 'HG19'\n if chrom == 1 and chromEnd > 249250622\n chromEnds << homo_region\n end\n if chrom == 2 and chromEnd > 243199373\n chromEnds << homo_region\n end\n if chrom == 3 and chromEnd > 198022430\n chromEnds << homo_region\n end\n if chrom == 4 and chromEnd > 191154276\n chromEnds << homo_region\n end\n if chrom == 5 and chromEnd > 180915260\n chromEnds << homo_region\n end\n if chrom == 6 and chromEnd > 171115067\n chromEnds << homo_region\n end\n if chrom == 7 and chromEnd > 159138663\n chromEnds << homo_region\n end\n if chrom == 8 and chromEnd > 146364022\n chromEnds << homo_region\n end\n if chrom == 9 and chromEnd > 141213431\n chromEnds << homo_region\n end\n if chrom == 10 and chromEnd > 135534747\n chromEnds << homo_region\n end\n if chrom == 11 and chromEnd > 135006516\n chromEnds << homo_region\n end\n if chrom == 12 and chromEnd > 133851895\n chromEnds << homo_region\n end\n if chrom == 13 and chromEnd > 115169878\n chromEnds << homo_region\n end\n if chrom == 14 and chromEnd > 107349540\n chromEnds << homo_region\n end\n if chrom == 15 and chromEnd > 102531392\n chromEnds << homo_region\n end\n if chrom == 16 and chromEnd > 90354753\n chromEnds << homo_region\n end\n if chrom == 17 and chromEnd > 81195210\n chromEnds << homo_region\n end\n if chrom == 18 and chromEnd > 78077248\n chromEnds << homo_region\n end\n if chrom == 19 and chromEnd > 59128983\n chromEnds << homo_region\n end\n if chrom == 20 and chromEnd > 63025521\n chromEnds << homo_region\n end\n if chrom == 21 and chromEnd > 48129895\n chromEnds << homo_region\n end\n if chrom == 22 and chromEnd > 51304567\n chromEnds << homo_region\n end\n end\n end\n if link_region.present? \n link_region_components=link_region.split(/[:-]/)\n chrom = link_region_components[0].delete('^0-9').to_i \n chromStart = link_region_components[1].to_i\n chromEnd = link_region_components[2].to_i\n if chromStart > chromEnd\n length << link_region\n end \n if build == 'HG19'\n if chrom == 1 and chromEnd > 249250622\n chromEnds << link_region\n end\n if chrom == 2 and chromEnd > 243199373\n chromEnds << link_region\n end\n if chrom == 3 and chromEnd > 198022430\n chromEnds << link_region\n end\n if chrom == 4 and chromEnd > 191154276\n chromEnds << link_region\n end\n if chrom == 5 and chromEnd > 180915260\n chromEnds << link_region\n end\n if chrom == 6 and chromEnd > 171115067\n chromEnds << link_region\n end\n if chrom == 7 and chromEnd > 159138663\n chromEnds << link_region\n end\n if chrom == 8 and chromEnd > 146364022\n chromEnds << link_region\n end\n if chrom == 9 and chromEnd > 141213431\n chromEnds << link_region\n end\n if chrom == 10 and chromEnd > 135534747\n chromEnds << link_region\n end\n if chrom == 11 and chromEnd > 135006516\n chromEnds << link_region\n end\n if chrom == 12 and chromEnd > 133851895\n chromEnds << link_region\n end\n if chrom == 13 and chromEnd > 115169878\n chromEnds << link_region\n end\n if chrom == 14 and chromEnd > 107349540\n chromEnds << link_region\n end\n if chrom == 15 and chromEnd > 102531392\n chromEnds << link_region\n end\n if chrom == 16 and chromEnd > 90354753\n chromEnds << link_region\n end\n if chrom == 17 and chromEnd > 81195210\n chromEnds << link_region\n end\n if chrom == 18 and chromEnd > 78077248\n chromEnds << link_region\n end\n if chrom == 19 and chromEnd > 59128983\n chromEnds << link_region\n end\n if chrom == 20 and chromEnd > 63025521\n chromEnds << link_region\n end\n if chrom == 21 and chromEnd > 48129895\n chromEnds << link_region\n end\n if chrom == 22 and chromEnd > 51304567\n chromEnds << link_region\n end\n end \n end \n end \n if length.any? and chromEnds.any?\n to_message = length.join(', ')\n to_message2 = chromEnds.join(', ')\n raise (\"The end position of the chromosome is before the start of the chromosome: #{to_message} And the end position of the chromosome is longer than the length of the chromosome: #{to_message2}\") \n elsif length.any?\n to_message = length.join(', ')\n raise (\"The end position of the chromosome is before the start of the chromosome: #{to_message}\")\n elsif chromEnds.any?\n to_message2 = chromEnds.join(', ')\n raise (\"The end position of the chromosome is longer than the length of the chromosome: #{to_message2}\")\n end\n if cnvNumber.any?\n to_message3 = cnvNumber.join(', ')\n raise (\"You have an invalid CNV for: #{to_message3}\" ) \n end\nend \nif hzregion > 0\n if not subj.get_value('Total Amount of Homozygosity that Segregates with Disease').present?\n hz = (sum/3000000000 *100).round(3)\n hz_print = hz.to_s+'%'\n\n params[:defaults] = {\n 'Total Amount of Homozygosity that Segregates with Disease'=> hz_print\n }\n else \n params[:defaults] = {\n 'Total Amount of Homozygosity that Segregates with Disease'=> subj.get_value('Total Amount of Homozygosity that Segregates with Disease')\n }\n end\nelse\n if not subj.get_value('Total Amount of Homozygosity that Segregates with Disease').present?\n hz_print = '0%'\n params[:defaults] = {\n 'Total Amount of Homozygosity that Segregates with Disease'=> hz_print\n }\n else\n params[:defaults] = {\n 'Total Amount of Homozygosity that Segregates with Disease'=> subj.get_value('Total Amount of Homozygosity that Segregates with Disease')\n }\n end \nend\n",

"btn_scale": "large",

"after_code_params": [

"UDPM-2072"

]

}

],

"show_options": {

"top": 346.98863220214844,

"left": 888.9999084472656

}

},

{

"name": "PennCNV",

"uuid": "UDPM-310",

"owner": "UDPM-67",

"managers": "UDPM-27",

"performers": "UDPM-3",

"duration": 2.0,

"tools": [

{

"name": "PennCNV Information",

"uuid": "UDPM-364",

"input_type": "UDPM-59",

"output_type": "UDPM-59",

"flags": 1,

"options": {

"udfs": [

"UDPM-1687",

"UDPM-2145",

"UDPM-863",

"UDPM-154",

"UDPM-2116",

"UDPM-401"

]

}

}

],

"next_states": [

{

"state_name": "Analysis Queue",

"state_uuid": "UDPM-1069",

"btn_text": "Add to Analysis Queue",

"btn_css": "rgb(13, 73, 123)",

"btn_scale": "large"

},

{

"state_name": "Analysis on Hold",

"state_uuid": "UDPM-600",

"btn_text": "Place Analysis on Hold",

"btn_css": "#456",

"after_code": "subj.set_value('Rationale/Reasoning', params['Rationale/Reasoning'] )",

"after_code_params": [

"UDPM-883"

]

}

],

"show_options": {

"top": 353,

"left": 284

}

},

{

"name": "QC: Assess Data Quality",

"uuid": "UDPM-134",

"owner": "UDPM-67",

"managers": "UDPM-27",

"performers": "UDPM-3",

"tools": [

{

"name": "Chip Information",

"uuid": "UDPM-143",

"input_type": "UDPM-59",

"output_type": "UDPM-59",

"description": "Quality check",

"flags": 1,

"options": {

"udfs": [

"UDPM-868",

"UDPM-1277",

"UDPM-862",

"UDPM-1203"

]

}

}

],

"next_states": [

{

"state_name": "Analysis Queue",

"state_uuid": "UDPM-1069",

"btn_text": "Add to Analysis Queue",

"btn_css": "rgb(13, 73, 123)",

"btn_scale": "large"

},

{

"state_name": "PennCNV",

"state_uuid": "UDPM-310",

"btn_text": "Run PennCNV",

"btn_css": "#456",

"hide_button_if_not_condition": true,

"conditions": [

{

"name": "Patient&rarr;Affected_status",

"condition": "=",

"value": "Affected",

"message": "",

"subject_type": "UDPM-1",

"udf": "UDPM-11"

}

]

},

{

"state_name": "Analysis on Hold",

"state_uuid": "UDPM-600",

"btn_text": "Place Analysis on Hold",

"btn_css": "#456",

"after_code": "subj.set_value('Rationale/Reasoning', params['Rationale/Reasoning'] )",

"after_code_params": [

"UDPM-883"

]

},

{

"state_name": "Analysis Cancelled",

"state_uuid": "UDPM-640",

"btn_text": "Cancel Analysis",

"btn_css": "#456",

"after_code": "subj.set_value('Rationale/Reasoning', params['Rationale/Reasoning'] )",

"after_code_params": [

"UDPM-883"

]

}

],

"show_options": {

"top": 32,

"left": 71

}

}

],

"entry_point": "UDPM-134"

},{

"name": "Question to Primary Care /Family",

"uuid": "UDPM-86",

"subject_type": "UDPM-84",

"state_defs": [

{

"name": "Communication with Primary Care / Family",

"uuid": "UDPM-344",

"owner": "UDPM-67",

"managers": "UDPM-9",

"performers": "UDPM-2",

"duration": 5.0,

"tools": [

{

"name": "Notes to Self",

"uuid": "UDPM-378",

"input_type": "UDPM-84",

"output_type": "UDPM-84",

"flags": 1,

"options": {

"udfs": [

"UDPM-191"

]

}

},

{

"name": "Enter Answers",

"uuid": "UDPM-377",

"input_type": "UDPM-84",

"output_type": "UDPM-84",

"flags": 1,

"options": {

"udfs": [

"UDPM-411"

]

}

}

],

"next_states": [

{

"state_name": "Completed",

"state_uuid": "UDPM-345",

"btn_text": "Complete",

"btn_css": "#456",

"after_code": "# Extra code here\n",

"before_code": "# Script to run Before Transition is executed\nparams[:ask_next_performer] = true"

}

],

"show_options": {

"top": 56,

"left": 84

}

},

{

"name": "Completed",

"uuid": "UDPM-345",

"owner": "UDPM-67",

"managers": "UDPM-1",

"performers": "UDPM-2",

"show_options": {

"top": 311,

"left": 558

},

"end_task": true

}

],

"entry_point": "UDPM-344",

"ask_performer": true,

"show_udfs": "UDPM-184,UDPM-411,UDPM-51,UDPM-191"

},{

"name": "Rejection Letter",

"uuid": "UDPM-87",

"subject_type": "UDPM-85",

"state_defs": [

{

"name": "Completed",

"uuid": "UDPM-347",

"owner": "UDPM-67",

"managers": "UDPM-5",

"performers": "UDPM-5",

"show_options": {

"top": 180.99998474121094,

"left": 400

},

"end_task": true

},

{

"name": "Requested",

"uuid": "UDPM-346",

"owner": "UDPM-67",

"managers": "UDPM-49",

"performers": "UDPM-49",

"duration": 5.0,

"tools": [

{

"name": "Compose Rejection Letter",

"uuid": "UDPM-379",

"input_type": "UDPM-85",

"output_type": "UDPM-85",

"flags": 1,

"options": {

"udfs": [

"UDPM-412"

]

}

}

],

"next_states": [

{

"state_name": "Completed",

"state_uuid": "UDPM-347",

"btn_text": "Complete",

"btn_css": "#456",

"after_code": "# Extra code here\nself.next_state_performer = User.curr_user",

"before_code": "# Script to run Before Transition is executed\nparams[:ask_next_performer] = true"

}

],

"show_options": {

"top": 81,

"left": 48

}

}

],

"entry_point": "UDPM-346",

"ask_performer": true

},{

"name": "Collaborations",

"uuid": "UDPM-140",

"subject_type": "UDPM-147",

"state_defs": [

{

"name": "Amend MTA",

"uuid": "UDPM-1029",

"owner": "UDPM-67",

"managers": "UDPM-45",

"performers": "UDPM-65",

"duration": 14.0,

"tools": [

{

"name": "MTA Amendment",

"uuid": "UDPM-1314",

"input_type": "UDPM-147",

"output_type": "UDPM-251",

"flags": 0,

"options": {

"obj_type": "SubjectType",

"do_not_open_subject": true

}

}

],

"next_states": [

{

"state_name": "Request Material Transfer",

"state_uuid": "UDPM-674",

"btn_text": "MTA Amendment Complete",

"btn_css": "#456"

},

{

"state_name": "Prepare Samples for Shipment",

"state_uuid": "UDPM-642",

"btn_text": "MTA Amended/Prepare Samples",

"btn_css": "rgb(13, 73, 123)",

"btn_scale": "large"

}

],

"show_options": {

"top": 42,

"left": 526

}

},

{

"name": "Awaiting Further Information",

"uuid": "UDPM-950",

"owner": "UDPM-67",

"managers": "UDPM-9",

"performers": "UDPM-49",

"tools": [

{

"name": "Sample Information",

"uuid": "UDPM-1202",

"input_type": "UDPM-147",

"output_type": "UDPM-147",

"flags": 1,

"options": {

"udfs": [

"UDPM-2136",

"UDPM-1512",

"UDPM-1303",

"UDPM-2484",

"UDPM-1312",

"UDPM-2374"

]

}

}

],

"next_states": [

{

"state_name": "Prepare Samples for Shipment",

"state_uuid": "UDPM-642",

"btn_text": "Return to Sample Preparation",

"btn_css": "#456",

"after_code": "self.next_state_performer = find_user('[redacted]')",

"before_code": "params[:ask_next_performer] = true"

}

],

"show_options": {

"top": 583,

"left": 408

}

},

{

"name": "Collaboration Cancelled",

"uuid": "UDPM-681",

"owner": "UDPM-67",

"managers": "UDPM-9",

"performers": "UDPM-2",

"show_options": {

"top": 356.99998474121094,

"left": 9

},

"end_task": true

},

{

"name": "Collaboration Complete",

"uuid": "UDPM-646",

"owner": "UDPM-67",

"managers": "UDPM-9",

"performers": "UDPM-2",

"show_options": {

"top": 747.9971160888672,

"left": 80.99713134765625

},

"end_task": true

},

{

"name": "Hold for Batch",

"uuid": "UDPM-1019",

"owner": "UDPM-67",

"managers": "UDPM-22",

"performers": "UDPM-22",

"next_states": [

{

"state_name": "Prepare Samples for Shipment",

"state_uuid": "UDPM-642",

"btn_text": "Return to Preparation",

"btn_css": "#456",

"after_code": "params[:ask_next_performer] = true"

}

],

"show_options": {

"top": 602,

"left": 153

}

},

{

"name": "Interpret Results",

"uuid": "UDPM-645",

"owner": "UDPM-67",

"managers": "UDPM-9",

"performers": "UDPM-2",

"tools": [

{

"name": "Notes",

"uuid": "UDPM-1130",

"input_type": "UDPM-147",

"output_type": "UDPM-147",

"flags": 1,

"options": {

"udfs": [

"UDPM-2374"

]

}

}

],

"next_states": [

{

"state_name": "Collaboration Complete",

"state_uuid": "UDPM-646",

"btn_text": "Collaboration Complete",

"btn_css": "#456"

},

{

"state_name": "Upload Results",

"state_uuid": "UDPM-644",

"btn_text": "Add Additional Reports",

"btn_css": "#456"

}

],

"show_options": {

"top": 773,

"left": 479

}

},

{

"name": "Prepare Samples for Shipment",

"uuid": "UDPM-642",

"owner": "UDPM-67",

"managers": "UDPM-22",

"performers": "UDPM-48",

"tools": [

{

"name": "Collaboration Information",

"uuid": "UDPM-810",

"input_type": "UDPM-147",

"output_type": "UDPM-147",

"flags": 1,

"options": {

"udfs": [

"UDPM-6",

"UDPM-1417",

"UDPM-1512",

"UDPM-1303",

"UDPM-2130",

"UDPM-463",

"UDPM-2113",

"UDPM-2112",

"UDPM-2516",

"UDPM-2385",

"UDPM-1312",

"UDPM-503",

"UDPM-1694",

"UDPM-1486",

"UDPM-1399",

"UDPM-2374"

]

}

}

],

"next_states": [

{

"state_name": "Awaiting Further Information",

"state_uuid": "UDPM-950",

"btn_text": "Request Additional Information",

"btn_css": "#456",

"after_code": "subj.set_value('Rationale/Reasoning', params['Rationale/Reasoning'])\nsend_email(subj.created_by, find_email_template(\"Additional Sample Information Needed\"), subj)\nself.next_state_performer = subj.created_by",

"after_code_params": [

"UDPM-883"

]

},

{

"state_name": "Collaboration Cancelled",

"state_uuid": "UDPM-681",

"btn_text": "Cancel Collaboration",

"btn_css": "#456",

"after_code_params": [

"UDPM-883"

]

},

{

"state_name": "Sign Sample Shipment Letter",

"state_uuid": "UDPM-1021",

"btn_text": "Upload Unsigned Shipment Letter",

"btn_css": "#456",

"after_code": "#self.next_state_performer = User.curr_user\n\nself.next_state_performer = find_user('[redacted]')\n\nsubj.set_value('Shipment Letter', params['Shipment Letter'])\n\nsend_email(User.find_by_username('[redacted]'), find_email_template(\"Shipment Letter\"), subj)",

"btn_scale": "large",

"after_code_params": [

"UDPM-1797"

]

},

{

"state_name": "Hold for Batch",

"state_uuid": "UDPM-1019",

"btn_text": "Hold for Batch",

"btn_css": "#456"

}

],

"show_options": {

"top": 231,

"left": 221.00003051757812

}

},

{

"name": "Request Material Transfer",

"uuid": "UDPM-674",

"owner": "UDPM-67",

"managers": "UDPM-45",

"performers": "UDPM-45",

"tools": [

{

"name": "Information for Material Transfer Agreement",

"uuid": "UDPM-1016",

"input_type": "UDPM-147",

"output_type": "UDPM-147",

"flags": 1,

"options": {

"udfs": [

"UDPM-1417",

"UDPM-2141",

"UDPM-1699",

"UDPM-2136",

"UDPM-2489",

"UDPM-1512",

"UDPM-1303",

"UDPM-2484"

]

}

}

],

"next_states": [

{

"state_name": "Amend MTA",

"state_uuid": "UDPM-1029",

"btn_text": "Amend MTA",

"btn_css": "#456",

"after_code": "self.next_state_performer = find_user('[redacted]')\n\nrecipients = []\nrecipients << find_user_group('Consent Approval')\nsend_email(recipients, find_email_template(\"UDP MTA/ITA Amendment\"), subj)",

"btn_scale": "large"

},

{

"state_name": "Prepare Samples for Shipment",

"state_uuid": "UDPM-642",

"btn_text": "MTA Executed/Prepare Samples",

"btn_css": "#456",

"before_code": "internal = subj.get_value('Internal UDP Collaboration')\nmta = subj.get_value('Collaboration Project')\n#if internal.present? or mta.present?\n#else\nraise (\"MTA/ITA must be executed and uploaded or collaboration must be with an internal UDP collaborator.\" ) unless internal.present? or mta.present?\n#end\n\n\nparams[:ask_next_performer] = true",

"btn_scale": "large"

}

],

"em_performer_new_job": true,

"show_options": {

"top": 14,

"left": 41

}

},

{

"name": "Samples in Transit",

"uuid": "UDPM-649",

"owner": "UDPM-67",

"managers": "UDPM-22",

"performers": "UDPM-46",

"next_states": [

{

"state_name": "Samples Received in Bethesda",

"state_uuid": "UDPM-719",

"btn_text": "Samples Received by Support",

"btn_css": "#456",

"after_code": "self.next_state_performer = find_user('[redacted]')",

"btn_scale": "large"

}

],

"show_options": {

"top": 212,

"left": 1097

}

},

{

"name": "Samples Received in Bethesda",

"uuid": "UDPM-719",

"owner": "UDPM-67",

"managers": "UDPM-22",

"performers": "UDPM-4",

"tools": [

{

"name": "Additional Shipment Information",

"uuid": "UDPM-884",

"input_type": "UDPM-147",

"output_type": "UDPM-147",

"flags": 1,

"options": {

"udfs": [

"UDPM-1672",

"UDPM-1797",

"UDPM-309",

"UDPM-1515"

]

}

}

],

"next_states": [

{

"state_name": "Samples Shipped",

"state_uuid": "UDPM-720",

"btn_text": "Samples Shipped to Collaborator",

"btn_css": "#456",

"after_code": "self.next_state_performer = User.curr_user\n",

"btn_scale": "large"

}

],

"show_options": {

"top": 482,

"left": 1178.550048828125

}

},

{

"name": "Samples Shipped",

"uuid": "UDPM-720",

"owner": "UDPM-67",

"managers": "UDPM-22",

"performers": "UDPM-21",

"tools": [

{

"name": "Update Tracking Information",

"uuid": "UDPM-1149",

"input_type": "UDPM-147",

"output_type": "UDPM-147",

"flags": 1,

"options": {

"udfs": [

"UDPM-1672",

"UDPM-309",

"UDPM-1515"

]

}

}

],

"next_states": [

{

"state_name": "Upload Results",

"state_uuid": "UDPM-644",

"btn_text": "Samples Received by Collaborator",

"btn_css": "#456",

"after_code": "self.next_state_performer = find_user('[redacted]')"

}

],

"show_options": {

"top": 726,

"left": 1120.2333984375

}

},

{

"name": "Shipment Letter Signed",

"uuid": "UDPM-1022",

"owner": "UDPM-67",

"managers": "UDPM-48",

"performers": "UDPM-48",

"duration": 3.0,

"tools": [

{

"name": "Shipment Information",

"uuid": "UDPM-1406",

"input_type": "UDPM-147",

"output_type": "UDPM-147",

"flags": 1,

"options": {

"udfs": [

"UDPM-1672",

"UDPM-2679",

"UDPM-309",

"UDPM-1515"

]

}

}

],

"next_states": [

{

"state_name": "Samples in Transit",

"state_uuid": "UDPM-649",

"btn_text": "Courier Samples to Support for Shipment",

"btn_css": "#456",

"after_code": "subj.set_value('Date Sample Sent', params['Date Sample Sent'])",

"btn_scale": "large",

"after_code_params": [

"UDPM-1672"

]

},

{

"state_name": "Samples Shipped",

"state_uuid": "UDPM-720",

"btn_text": "Samples Shipped to Collaborator",

"btn_css": "#456",

"after_code": "self.next_state_performer = User.curr_user",

"btn_scale": "large"

}

],

"show_options": {

"top": 578,

"left": 756

}

},

{

"name": "Sign Sample Shipment Letter",

"uuid": "UDPM-1021",

"owner": "UDPM-67",

"managers": "UDPM-66",

"performers": "UDPM-66",

"duration": 3.0,

"next_states": [

{

"state_name": "Shipment Letter Signed",

"state_uuid": "UDPM-1022",

"btn_text": "Upload Signed Shipment Letter",

"btn_css": "#456",

"after_code": "subj.set_value('Signed Shipment Letter', params['Signed Shipment Letter'])\n\n#send_email(self.next_state_performer, find_email_template(\"Shipment Letter\"), subj)",

"before_code": "params[:ask_next_performer] = true",

"btn_scale": "large",

"after_code_params": [

"UDPM-2679"

]

}

],

"show_options": {

"top": 266.99998474121094,

"left": 650.0000305175781

}

},

{

"name": "Upload Results",

"uuid": "UDPM-644",

"owner": "UDPM-67",

"managers": "UDPM-22",

"performers": "UDPM-47",

"tools": [

{

"name": "Upload Report",

"uuid": "UDPM-841",

"input_type": "UDPM-147",

"output_type": "UDPM-178",

"flags": 0,

"options": {

"obj_type": "SubjectType",

"do_not_open_subject": true

}

}

],

"next_states": [

{

"state_name": "Interpret Results",

"state_uuid": "UDPM-645",

"btn_text": "Interpret Results",

"btn_css": "rgb(13, 73, 123)",

"btn_scale": "large"

}

],

"show_options": {

"top": 756.9914398193359,

"left": 828.9942932128906

}

}

],

"entry_point": "UDPM-674",

"show_udfs": "UDPM-1303,UDPM-2130,UDPM-1694,UDPM-2113"

},{

"name": "Neurotransmitter",

"uuid": "UDPM-143",

"subject_type": "UDPM-169",

"state_defs": [

{

"name": "Received",

"uuid": "UDPM-666",

"owner": "UDPM-67",

"managers": "UDPM-22",

"performers": "UDPM-69",

"next_states": [

{

"state_name": "Stored",

"state_uuid": "UDPM-667",

"btn_text": "Store",

"btn_css": "#456",

"after_code": "require_script 'lims_helper'\n\nvial_count = params['Number of Vials to Store']\nself.next_state_performer = User.curr_user\nsubmit_to_fpro(subj, 'Neurotransmitter', '7000000530', vial_count)",

"after_code_params": [

"UDPM-1450"

]

}

],

"show_options": {

"top": 86,

"left": 78

}

},

{

"name": "Stored",

"uuid": "UDPM-667",

"owner": "UDPM-67",

"managers": "UDPM-22",

"performers": "UDPM-69",

"show_options": {

"top": 180,

"left": 400

},

"end_task": true

}

],

"entry_point": "UDPM-666",

"ask_performer": true

},{

"name": "CLIA Validation Workflow",

"uuid": "UDPM-5",

"subject_type": "UDPM-15",

"state_defs": [

{

"name": "Awaiting Information",

"uuid": "UDPM-1042",

"owner": "UDPM-67",

"managers": "UDPM-9",

"performers": "UDPM-49",

"tools": [

{

"name": "Additional Information",

"uuid": "UDPM-1348",

"input_type": "UDPM-15",

"output_type": "UDPM-15",

"flags": 1,

"options": {

"udfs": [

"UDPM-2024",

"UDPM-2082",

"UDPM-191"

]

}

}

],

"next_states": [

{

"state_name": "Cancel Request",

"state_uuid": "UDPM-1043",

"btn_text": "Cancel Request",

"btn_css": "rgb(13, 73, 123)",

"btn_scale": "large"

},

{

"state_name": "Prepare Samples",

"state_uuid": "UDPM-855",

"btn_text": "Return to Sample Preparation",

"btn_css": "rgb(13, 73, 123)",

"after_code": "self.next_state_performer = find_user('[redacted]')",

"btn_scale": "large"

}

],

"show_options": {

"top": 401.96873474121094,

"left": 380.9630432128906

}

},

{

"name": "Cancel Request",

"uuid": "UDPM-1043",

"owner": "UDPM-67",

"managers": "UDPM-9",

"performers": "UDPM-49",

"show_options": {

"top": 291.96873474121094,

"left": 459.9800720214844

},

"end_task": true

},

{

"name": "Complete Validation",

"uuid": "UDPM-214",

"owner": "UDPM-67",

"managers": "UDPM-9",

"performers": "UDPM-71",

"duration": 5.0,

"show_options": {

"top": 671.5425872802734,

"left": 710.9686584472656

},

"end_task": true

},

{

"name": "Final CLIA Report Generation",

"uuid": "UDPM-19",

"owner": "UDPM-67",

"managers": "UDPM-9",

"performers": "UDPM-71",

"duration": 30.0,

"tools": [

{

"name": "CLIA Report Upload",

"uuid": "UDPM-19",

"input_type": "UDPM-15",

"output_type": "UDPM-15",

"flags": 1,

"options": {

"udfs": [

"UDPM-41",

"UDPM-129",

"UDPM-2207"

]

}

}

],

"next_states": [

{

"state_name": "Complete Validation",

"state_uuid": "UDPM-214",

"btn_text": "Complete Validation",

"btn_css": "#456"

}

],

"show_options": {

"top": 678.5568084716797,

"left": 353.9800720214844

}

},

{

"name": "POTS",

"uuid": "UDPM-854",

"owner": "UDPM-67",

"managers": "UDPM-40",

"performers": "UDPM-40",

"tools": [

{

"name": "POTS Entry",

"uuid": "UDPM-1085",

"input_type": "UDPM-15",

"output_type": "UDPM-15",

"flags": 1,

"options": {

"udfs": [

"UDPM-1752",

"UDPM-1753"

]

}

}

],

"next_states": [

{

"state_name": "Prepare Samples",

"state_uuid": "UDPM-855",

"btn_text": "POTS Entry Complete",

"btn_css": "#456",

"after_code": "self.next_state_performer = find_user('[redacted]')"

}

],

"em_performer_new_job": true,

"show_options": {

"top": 213.9630584716797,

"left": 62.9801025390625

}

},

{

"name": "Prepare Samples",

"uuid": "UDPM-855",

"owner": "UDPM-67",

"managers": "UDPM-22",

"performers": "UDPM-48",

"next_states": [

{

"state_name": "Awaiting Information",

"state_uuid": "UDPM-1042",

"btn_text": "Return to Requestor",

"btn_css": "rgb(13, 73, 123)",

"after_code": "subj.set_value('Rationale/Reasoning', params['Rationale/Reasoning'])\nsend_email(subj.created_by, find_email_template(\"CLIA Validation Return\"), subj)\nself.next_state_performer = subj.created_by",

"btn_scale": "large",

"after_code_params": [

"UDPM-883"

]

},

{

"state_name": "Send DNA for Validation",

"state_uuid": "UDPM-18",

"btn_text": "Send to Building 10 for Shipment",

"btn_css": "#456",

"before_code": "params[:ask_next_performer] = true"

}

],

"show_options": {

"top": 416.98008728027344,

"left": 60.988616943359375

}

},

{

"name": "Request Returned",

"uuid": "UDPM-1105",

"owner": "UDPM-67",

"managers": "UDPM-32",

"performers": "UDPM-49",

"next_states": [

{

"state_name": "Variant Information",

"state_uuid": "UDPM-211",

"btn_text": "Resend Request",

"btn_css": "rgb(13, 73, 123)",

"btn_scale": "large"

}

],

"em_performer_new_job": true,

"show_options": {

"top": 185.98333740234375,

"left": 353.9666748046875

}

},

{

"name": "Send DNA for Validation",

"uuid": "UDPM-18",

"owner": "UDPM-67",

"managers": "UDPM-9",

"performers": "UDPM-46",

"tools": [

{

"name": "Shipment Information",

"uuid": "UDPM-18",

"input_type": "UDPM-15",

"output_type": "UDPM-15",

"flags": 1,

"options": {

"udfs": [

"UDPM-2082",

"UDPM-309",

"UDPM-1515",

"UDPM-63"

]

}

}

],

"next_states": [

{

"state_name": "Final CLIA Report Generation",

"state_uuid": "UDPM-19",

"btn_text": "DNA Sent to Lab",

"btn_css": "#456",

"after_code": "self.next_state_performer = find_user('[redacted]')",

"btn_scale": "large"

}

],

"show_options": {

"top": 600.5596160888672,

"left": 79.98861694335938

}

},

{

"name": "Variant Information",

"uuid": "UDPM-211",

"owner": "UDPM-67",

"managers": "UDPM-32",

"performers": "UDPM-46",

"duration": 5.0,

"tools": [

{

"name": "Additional Information",

"uuid": "UDPM-1086",

"input_type": "UDPM-15",

"output_type": "UDPM-15",

"flags": 1,

"options": {

"udfs": [

"UDPM-2195",

"UDPM-1873",

"UDPM-1707",

"UDPM-2024",

"UDPM-2206",

"UDPM-2082"

]

}

}

],

"next_states": [

{

"state_name": "POTS",

"state_uuid": "UDPM-854",

"btn_text": "Request for POTS Processing",

"btn_css": "#456"

},

{

"state_name": "Request Returned",

"state_uuid": "UDPM-1105",

"btn_text": "Return Request to Sender",

"btn_css": "rgb(13, 73, 123)",

"after_code": "subj.set_value('Comments/Special Instructions', params['Comments/Special Instructions'])\nself.next_state_performer = subj.created_by",

"btn_scale": "large",

"after_code_params": [

"UDPM-1344"

]

}

],

"em_performer_new_job": true,

"show_options": {

"top": 22.991485595703125,

"left": 347.9573669433594

}

}

],

"entry_point": "UDPM-211",

"show_udfs": "UDPM-2082,UDPM-2024,UDPM-1696"

},{

"name": "Patient Review",

"uuid": "UDPM-83",

"subject_type": "UDPM-83",

"state_defs": [

{

"name": "Completed",

"uuid": "UDPM-336",

"owner": "UDPM-67",

"managers": "UDPM-9",

"performers": "UDPM-31",

"duration": 5.0,

"em_performer_due_job": true,

"show_options": {

"top": 179,

"left": 401

},

"end_task": true

},

{

"name": "Requested",

"uuid": "UDPM-335",

"owner": "UDPM-67",

"managers": "UDPM-9",

"performers": "UDPM-68",

"duration": 30.0,

"tools": [

{

"name": "Reviewer Notes",

"uuid": "UDPM-1313",

"input_type": "UDPM-83",

"output_type": "UDPM-83",

"flags": 1,

"options": {

"udfs": [

"UDPM-2335",

"UDPM-2294",

"UDPM-2293",

"UDPM-2069",

"UDPM-348",

"UDPM-191",

"UDPM-234",

"UDPM-236",

"UDPM-1497",

"UDPM-1496"

]

}

},

{

"name": "Reviewer's Assessment",

"uuid": "UDPM-1123",

"input_type": "UDPM-83",

"output_type": "UDPM-83",

"flags": 1,

"options": {

"udfs": [

"UDPM-2335",

"UDPM-2294",

"UDPM-2293",

"UDPM-348",

"UDPM-234",

"UDPM-236",

"UDPM-1497",

"UDPM-1496",

"UDPM-581",

"UDPM-4",

"UDPM-881",

"UDPM-730",

"UDPM-313",

"UDPM-2334",

"UDPM-52",

"UDPM-53",

"UDPM-51",

"UDPM-1569",

"UDPM-2321",

"UDPM-2322",

"UDPM-1438",

"UDPM-2323",

"UDPM-2324",

"UDPM-2325",

"UDPM-2326",

"UDPM-2327",

"UDPM-2328",

"UDPM-2329",

"UDPM-2330",

"UDPM-2331",

"UDPM-2332",

"UDPM-2333"

]

}

}

],

"next_states": [

{

"state_name": "Completed",

"state_uuid": "UDPM-336",

"btn_text": "Complete Review",

"btn_css": "#456",

"after_code": "self.next_state_performer = find_user('[redacted]')\n",

"before_code": "# Script to run Before Transition is executed\nparams[:ask_next_performer] = true",

"btn_scale": "large"

},

{

"state_name": "Withdrawn",

"state_uuid": "UDPM-337",

"btn_text": "Withdraw",

"btn_css": "#456",

"after_code": "# Extra code here\nself.next_state_performer = User.curr_user"

}

],

"em_performer_new_job": true,

"show_options": {

"top": 68,

"left": 90

}

},

{

"name": "Withdrawn",

"uuid": "UDPM-337",

"owner": "UDPM-67",

"managers": "UDPM-9",

"performers": "UDPM-2",

"show_options": {

"top": 363.99998474121094,

"left": 135

},

"end_task": true

}

],

"entry_point": "UDPM-335",

"ask_performer": true,

"show_udfs": "UDPM-2293,UDPM-2294,UDPM-2335,UDPM-234,UDPM-236,UDPM-348,UDPM-1496,UDPM-1497"

},{

"name": "Patient Workflow",

"uuid": "UDPM-53",

"subject_type": "UDPM-1",

"state_defs": [

{

"name": "Accepted - Patient Hold",

"uuid": "UDPM-197",

"owner": "UDPM-67",

"managers": "UDPM-49",

"performers": "UDPM-49",

"duration": 14.0,

"tools": [

{

"name": "Upload Non-PII Files",

"uuid": "UDPM-1469",

"input_type": "UDPM-1",

"output_type": "UDPM-1",

"flags": 6,

"before_code": "require_script 'non_pii_upload_helper'\nbefore_upload(params)\n",

"after_code": "require_script 'non_pii_upload_helper'\nafter_upload(params, subj)\n"

},

{

"name": "Upload PII Files",

"uuid": "UDPM-1470",

"input_type": "UDPM-1",

"output_type": "UDPM-1",

"flags": 6,

"before_code": "require_script 'pii_upload_helper'\nbefore_upload(params)\n",

"after_code": "require_script 'pii_upload_helper'\nafter_upload(params, subj)\n"

},

{

"name": "Add Diagnosis",

"uuid": "UDPM-969",

"input_type": "UDPM-1",

"output_type": "UDPM-185",

"flags": 0,

"options": {

"obj_type": "SubjectType"

}

},

{

"name": "PhenoTips Review",

"uuid": "UDPM-1031",

"input_type": "UDPM-1",

"output_type": "UDPM-205",

"flags": 0,

"options": {

"obj_type": "SubjectType"

}

},

{

"name": "Patient Update",

"uuid": "UDPM-1110",

"input_type": "UDPM-1",

"output_type": "UDPM-1",

"flags": 1,

"options": {

"udfs": [

"UDPM-2861",

"UDPM-2862",

"UDPM-2863",

"UDPM-2316",

"UDPM-1979",

"UDPM-1807",

"UDPM-1722",

"UDPM-43",

"UDPM-28",

"UDPM-56",

"UDPM-15",

"UDPM-4",

"UDPM-11",

"UDPM-1",

"UDPM-2",

"UDPM-616",

"UDPM-3",

"UDPM-580",

"UDPM-151",

"UDPM-1330",

"UDPM-485",

"UDPM-502",

"UDPM-2202",

"UDPM-562",

"UDPM-739",

"UDPM-505",

"UDPM-731",

"UDPM-857",

"UDPM-528",

"UDPM-730",

"UDPM-881",

"UDPM-52",

"UDPM-53",

"UDPM-184",

"UDPM-54",

"UDPM-190",

"UDPM-1654",

"UDPM-1655",

"UDPM-882",

"UDPM-1869",

"UDPM-109",

"UDPM-497",

"UDPM-499",

"UDPM-498",

"UDPM-2016",

"UDPM-48",

"UDPM-2290",

"UDPM-2204",

"UDPM-1495",

"UDPM-589",

"UDPM-484",

"UDPM-182",

"UDPM-613",

"UDPM-1492",

"UDPM-545"

]

}

},

{

"name": "Change State",

"uuid": "UDPM-1303",

"input_type": "UDPM-1",

"output_type": "UDPM-1",

"flags": 6,

"after_code": "# Script to run After Tool is executed\nnext_state = params[\"patient_workflow_states\"]\nadvance_workflow(\"Patient Workflow\",next_state,subj)\nshow_message(\"Patient is now successfully placed at #{next_state} state\")",

"after_code_params": [

"UDPM-2284"

]

},

{

"name": "Create Initial Clinical Note",

"uuid": "UDPM-1439",

"input_type": "UDPM-1",

"output_type": "UDPM-267",

"flags": 0,

"hide_button_if_not_condition": true,

"conditions": [

{

"name": "Patient&rarr;Clinical Notes",

"condition": "Empty",

"value": "",

"message": "",

"subject_type": "UDPM-1",

"udf": "UDPM-2840"

}

],

"options": {

"obj_type": "SubjectType"

}

}

],

"next_states": [

{

"state_name": "UDP Triage",

"state_uuid": "UDPM-159",

"btn_text": "UDP Triage",

"btn_css": "#456",

"after_code": "self.next_state_performer = User.curr_user",

"before_code": "# Script to run Before Transition is executed\nparams[:ask_next_performer] = true"

},

{

"state_name": "Accepted Plan Visit",

"state_uuid": "UDPM-164",

"btn_text": "Accept/Schedule",

"btn_css": "#456",

"after_code": "self.next_state_performer = User.curr_user",

"before_code": "# Script to run Before Transition is executed\nparams[:ask_next_performer] = true"

}

],

"show_options": {

"top": 589,

"left": 526.5166015625

}

},

{

"name": "Accepted Plan Visit",

"uuid": "UDPM-164",

"owner": "UDPM-67",

"managers": "UDPM-49",

"performers": "UDPM-49",

"duration": 7.0,

"tools": [

{

"name": "Upload PII Files",

"uuid": "UDPM-1466",

"input_type": "UDPM-1",

"output_type": "UDPM-1",

"flags": 6,

"before_code": "require_script 'pii_upload_helper'\nbefore_upload(params)\n",

"after_code": "require_script 'pii_upload_helper'\nafter_upload(params, subj)\n"

},

{

"name": "Upload Non-PII Files",

"uuid": "UDPM-1465",

"input_type": "UDPM-1",

"output_type": "UDPM-1",

"flags": 6,

"before_code": "require_script 'non_pii_upload_helper'\nbefore_upload(params)\n",

"after_code": "require_script 'non_pii_upload_helper'\nafter_upload(params, subj)\n"

},

{

"name": "Request Review",

"uuid": "UDPM-382",

"input_type": "UDPM-1",

"output_type": "UDPM-83",

"flags": 0,

"before_code": "f = subj.get_value(\"Notes (PII)\")\n\nparams[:defaults] = {\n 'Clinical Questions'=> f\n}\n",

"options": {

"obj_type": "SubjectType"

}

},

{

"name": "Add Diagnosis",

"uuid": "UDPM-970",

"input_type": "UDPM-1",

"output_type": "UDPM-185",

"flags": 0,

"options": {

"obj_type": "SubjectType"

}

},

{

"name": "Update Patient ",

"uuid": "UDPM-1017",

"input_type": "UDPM-1",

"output_type": "UDPM-1",

"flags": 1,

"options": {

"udfs": [

"UDPM-2861",

"UDPM-2862",

"UDPM-2863",

"UDPM-2316",

"UDPM-1979",

"UDPM-1807",

"UDPM-1722",

"UDPM-43",

"UDPM-28",

"UDPM-56",

"UDPM-15",

"UDPM-4",

"UDPM-11",

"UDPM-1",

"UDPM-2",

"UDPM-616",

"UDPM-3",

"UDPM-580",

"UDPM-151",

"UDPM-1330",

"UDPM-485",

"UDPM-502",

"UDPM-2202",

"UDPM-562",

"UDPM-739",

"UDPM-505",

"UDPM-731",

"UDPM-857",

"UDPM-528",

"UDPM-730",

"UDPM-881",

"UDPM-52",

"UDPM-53",

"UDPM-184",

"UDPM-54",

"UDPM-190",

"UDPM-1654",

"UDPM-1655",

"UDPM-882",

"UDPM-1869",

"UDPM-109",

"UDPM-497",

"UDPM-499",

"UDPM-498",

"UDPM-2016",

"UDPM-48",

"UDPM-2290",

"UDPM-2204",

"UDPM-1495",

"UDPM-589",

"UDPM-484",

"UDPM-182",

"UDPM-907",

"UDPM-2778",

"UDPM-613",

"UDPM-1492",

"UDPM-545"

]

}

},

{

"name": "PhenoTips Review",

"uuid": "UDPM-1035",

"input_type": "UDPM-1",

"output_type": "UDPM-205",

"flags": 0,

"options": {

"obj_type": "SubjectType"

}

},

{

"name": "Change State",

"uuid": "UDPM-1297",

"input_type": "UDPM-1",

"output_type": "UDPM-1",

"flags": 6,

"after_code": "# Script to run After Tool is executed\nnext_state = params[\"patient_workflow_states\"]\nadvance_workflow(\"Patient Workflow\",next_state,subj)\nshow_message(\"Patient is now successfully placed at #{next_state} state\")",

"after_code_params": [

"UDPM-2284"

]

},

{

"name": "Letter Request",

"uuid": "UDPM-1347",

"input_type": "UDPM-1",

"output_type": "UDPM-253",

"flags": 0,

"options": {

"obj_type": "SubjectType"

}

},

{

"name": "Create Initial Clinical Note",

"uuid": "UDPM-1440",

"input_type": "UDPM-1",

"output_type": "UDPM-267",

"flags": 0,

"hide_button_if_not_condition": true,

"conditions": [

{

"name": "Patient&rarr;Clinical Notes",

"condition": "Empty",

"value": "",

"message": "",

"subject_type": "UDPM-1",

"udf": "UDPM-2840"

}

],

"options": {

"obj_type": "SubjectType"

}

}

],

"next_states": [

{

"state_name": "Admitted",

"state_uuid": "UDPM-1030",

"btn_text": "Admitted",

"btn_css": "rgb(13, 73, 123)",

"btn_scale": "large"

}

],

"show_options": {

"top": 431.42498779296875,

"left": 778.3125

}

},

{

"name": "Admitted",

"uuid": "UDPM-1030",

"owner": "UDPM-67",

"managers": "UDPM-49",

"performers": "UDPM-49",

"tools": [

{

"name": "Update Patient",

"uuid": "UDPM-1341",

"input_type": "UDPM-1",

"output_type": "UDPM-1",

"flags": 1,

"options": {

"udfs": [

"UDPM-2861",

"UDPM-2862",

"UDPM-2863",

"UDPM-1807",

"UDPM-1722",

"UDPM-43",

"UDPM-28",

"UDPM-56",

"UDPM-15",

"UDPM-4",

"UDPM-11",

"UDPM-1",

"UDPM-2",

"UDPM-616",

"UDPM-3",

"UDPM-151",

"UDPM-1330",

"UDPM-502",

"UDPM-2202",

"UDPM-562",

"UDPM-739",

"UDPM-505",

"UDPM-731",

"UDPM-857",

"UDPM-528",

"UDPM-730",

"UDPM-881",

"UDPM-52",

"UDPM-53",

"UDPM-184",

"UDPM-54",

"UDPM-190",

"UDPM-1654",

"UDPM-1655",

"UDPM-882",

"UDPM-1869",

"UDPM-497",

"UDPM-499",

"UDPM-498",

"UDPM-2016",

"UDPM-48",

"UDPM-2290",

"UDPM-907",

"UDPM-2778",

"UDPM-545"

]

}

},

{

"name": "Upload Non-PII Files",

"uuid": "UDPM-1467",

"input_type": "UDPM-1",

"output_type": "UDPM-1",

"flags": 6,

"before_code": "require_script 'non_pii_upload_helper'\nbefore_upload(params)\n",

"after_code": "require_script 'non_pii_upload_helper'\nafter_upload(params, subj)\n"

},

{

"name": "Upload PII Files",

"uuid": "UDPM-1468",

"input_type": "UDPM-1",

"output_type": "UDPM-1",

"flags": 6,

"before_code": "require_script 'pii_upload_helper'\nbefore_upload(params)\n",

"after_code": "require_script 'pii_upload_helper'\nafter_upload(params, subj)\n"

},

{

"name": "Date of Admission",

"uuid": "UDPM-1334",

"input_type": "UDPM-1",

"output_type": "UDPM-1",

"flags": 1,

"options": {

"udfs": [

"UDPM-2733"

]

}

},

{

"name": "Add Diagnosis",

"uuid": "UDPM-1337",

"input_type": "UDPM-1",

"output_type": "UDPM-185",

"flags": 0,

"options": {

"obj_type": "SubjectType"

}

},

{

"name": "PhenoTips Review",

"uuid": "UDPM-1338",

"input_type": "UDPM-1",

"output_type": "UDPM-205",

"flags": 0,

"options": {

"obj_type": "SubjectType"

}

},

{

"name": "Radiology Review",

"uuid": "UDPM-1339",

"input_type": "UDPM-1",

"output_type": "UDPM-246",

"flags": 0,

"before_code": "f = subj.get_value(\"Notes (PII)\")\n\nparams[:defaults] = {\n 'Notes'=> f\n}\n",

"options": {

"obj_type": "SubjectType"

}

},

{

"name": "Letter Request",

"uuid": "UDPM-1340",

"input_type": "UDPM-1",

"output_type": "UDPM-253",

"flags": 0,

"options": {

"obj_type": "SubjectType"

}

},

{

"name": "Change State",

"uuid": "UDPM-1377",

"input_type": "UDPM-1",

"output_type": "UDPM-1",

"flags": 6,

"after_code": "# Script to run After Tool is executed\nnext_state = params[\"patient_workflow_states\"]\nadvance_workflow(\"Patient Workflow\",next_state,subj)\nshow_message(\"Patient is now successfully placed at #{next_state} state\")",

"after_code_params": [

"UDPM-2284"

]

},

{

"name": "Create Initial Clinical Note",

"uuid": "UDPM-1441",

"input_type": "UDPM-1",

"output_type": "UDPM-267",

"flags": 0,

"hide_button_if_not_condition": true,

"conditions": [

{

"name": "Patient&rarr;Clinical Notes",

"condition": "Empty",

"value": "",

"message": "",

"subject_type": "UDPM-1",

"udf": "UDPM-2840"

}

],

"options": {

"obj_type": "SubjectType"

}

}

],

"next_states": [

{

"state_name": "End of Visit Checklist",

"state_uuid": "UDPM-1031",

"btn_text": "Visit Checklist",

"btn_css": "rgb(13, 73, 123)",

"btn_scale": "large"

}

],

"show_options": {

"top": 664.9914398193359,

"left": 763.9914855957031

}

},

{

"name": "Category - Not Assigned",

"uuid": "UDPM-862",

"owner": "UDPM-67",

"managers": "UDPM-49",

"performers": "UDPM-49",

"tools": [

{

"name": "Upload PII Files",

"uuid": "UDPM-1485",

"input_type": "UDPM-1",

"output_type": "UDPM-1",

"flags": 6,

"before_code": "require_script 'pii_upload_helper'\nbefore_upload(params)",

"after_code": "require_script 'pii_upload_helper'\nafter_upload(params, subj)"

},

{

"name": "Upload Non-PII FIles",

"uuid": "UDPM-1486",

"input_type": "UDPM-1",

"output_type": "UDPM-1",

"flags": 6,

"before_code": "require_script 'non_pii_upload_helper'\nbefore_upload(params)\n",

"after_code": "require_script 'non_pii_upload_helper'\nafter_upload(params, subj)\n"

},

{

"name": "Change Workflow State",

"uuid": "UDPM-1095",

"input_type": "UDPM-1",

"output_type": "UDPM-1",

"flags": 6,

"after_code": "# Script to run After Tool is executed\nnext_state = params[\"patient_workflow_states\"]\nadvance_workflow(\"Patient Workflow\",next_state,subj)\nshow_message(\"Patient is now successfully placed at #{next_state} state\")",

"after_code_params": [

"UDPM-2284"

]

},

{

"name": "Add Diagnosis",

"uuid": "UDPM-1102",

"input_type": "UDPM-1",

"output_type": "UDPM-185",

"flags": 0,

"options": {

"obj_type": "SubjectType"

}

},

{

"name": "Update Patient",

"uuid": "UDPM-1107",

"input_type": "UDPM-1",

"output_type": "UDPM-1",

"flags": 1,

"options": {

"udfs": [

"UDPM-2861",

"UDPM-2862",

"UDPM-2863",

"UDPM-2316",

"UDPM-1979",

"UDPM-1807",

"UDPM-1722",

"UDPM-43",

"UDPM-28",

"UDPM-56",

"UDPM-15",

"UDPM-4",

"UDPM-11",

"UDPM-1",

"UDPM-2",

"UDPM-616",

"UDPM-3",

"UDPM-580",

"UDPM-151",

"UDPM-1330",

"UDPM-485",

"UDPM-502",

"UDPM-2202",

"UDPM-562",

"UDPM-739",

"UDPM-505",

"UDPM-731",

"UDPM-857",

"UDPM-528",

"UDPM-730",

"UDPM-881",

"UDPM-52",

"UDPM-53",

"UDPM-184",

"UDPM-54",

"UDPM-190",

"UDPM-1654",

"UDPM-1655",

"UDPM-882",

"UDPM-1869",

"UDPM-109",

"UDPM-497",

"UDPM-499",

"UDPM-498",

"UDPM-2016",

"UDPM-48",

"UDPM-2290",

"UDPM-2204",

"UDPM-1495",

"UDPM-589",

"UDPM-484",

"UDPM-2312",

"UDPM-613",

"UDPM-1492",

"UDPM-545"

]

}

},

{

"name": "PhenoTips Review",

"uuid": "UDPM-1229",

"input_type": "UDPM-1",

"output_type": "UDPM-205",

"flags": 0,

"options": {

"obj_type": "SubjectType",

"do_not_open_subject": true

}

},

{

"name": "Create Initial Clinical Note",

"uuid": "UDPM-1430",

"input_type": "UDPM-1",

"output_type": "UDPM-267",

"flags": 0,

"hide_button_if_not_condition": true,

"conditions": [

{

"name": "Patient&rarr;Clinical Notes",

"condition": "Empty",

"value": "",

"message": "",

"subject_type": "UDPM-1",

"udf": "UDPM-2840"

}

],

"options": {

"obj_type": "SubjectType"

}

}

],

"show_options": {

"top": 95,

"left": 708.11669921875

},

"end_task": true

},

{

"name": "Cohort Sample Only",

"uuid": "UDPM-956",

"owner": "UDPM-67",

"managers": "UDPM-49",

"performers": "UDPM-49",

"tools": [

{

"name": "Upload Non-PII Files",

"uuid": "UDPM-1487",

"input_type": "UDPM-1",

"output_type": "UDPM-1",

"flags": 6,

"before_code": "require_script 'non_pii_upload_helper'\nbefore_upload(params)",

"after_code": "require_script 'non_pii_upload_helper'\nafter_upload(params, subj)\n"

},

{

"name": "Upload PII Files",

"uuid": "UDPM-1488",

"input_type": "UDPM-1",

"output_type": "UDPM-1",

"flags": 6,

"before_code": "require_script 'pii_upload_helper'\nbefore_upload(params)\n",

"after_code": "require_script 'pii_upload_helper'\nafter_upload(params, subj)\n"

},

{

"name": "Request GSH/GSSH Study",

"uuid": "UDPM-1499",

"input_type": "UDPM-1",

"output_type": "UDPM-273",

"flags": 0,

"before_code": "collaborator = find_subject( :subject_type=>'Collaborators', :name=>'[redacted]')\n\nparams[:defaults] = {\n 'Collaborator Names'=> collaborator,\n 'Sample Types' => \"Urine\"\n}\n ",

"after_code": "recipients = []\nrecipients << find_user_group('Consent Approval')\nsend_email(recipients, find_email_template(\"UDP MTA/ITA Amendment\"), subj)\n\nnewAmendment = create_subject('MTA/ITA Amendments')\ncollaborator = find_subject( :subject_type=>'Collaboration Projects', :name=>'[redacted] MTA')\nnewAmendment.set_value('MTA/ITA Collaboration',collaborator)\nnewAmendment.set_value('Patients', subj)\nstart_workflow('MTA/ITA Amendments Workflow',newAmendment)",

"options": {

"obj_type": "SubjectType"

}

},

{

"name": "Cohort Information",

"uuid": "UDPM-1207",

"input_type": "UDPM-1",

"output_type": "UDPM-1",

"flags": 1,

"options": {

"udfs": [

"UDPM-2861",

"UDPM-2862",

"UDPM-2863",

"UDPM-2490",

"UDPM-2597",

"UDPM-2491",

"UDPM-2492",

"UDPM-2596",

"UDPM-2494",

"UDPM-2016",

"UDPM-48",

"UDPM-2290"

]

}

},

{

"name": "Change States",

"uuid": "UDPM-1223",

"input_type": "UDPM-1",

"output_type": "UDPM-1",

"flags": 6,

"after_code": "# Script to run After Tool is executed\nnext_state = params[\"patient_workflow_states\"]\nadvance_workflow(\"Patient Workflow\",next_state,subj)\nshow_message(\"Patient is now successfully placed at #{next_state} state\")",

"after_code_params": [

"UDPM-2284"

]

},

{

"name": "PhenoTips Review",

"uuid": "UDPM-1233",

"input_type": "UDPM-1",

"output_type": "UDPM-205",

"flags": 0,

"options": {

"obj_type": "SubjectType",

"do_not_open_subject": true

}

},

{

"name": "Add Diagnosis",

"uuid": "UDPM-1238",

"input_type": "UDPM-1",

"output_type": "UDPM-185",

"flags": 0,

"options": {

"obj_type": "SubjectType"

}

},

{

"name": "Create Initial Clinical Note",

"uuid": "UDPM-1429",

"input_type": "UDPM-1",

"output_type": "UDPM-267",

"flags": 0,

"hide_button_if_not_condition": true,

"conditions": [

{

"name": "Patient&rarr;Clinical Notes",

"condition": "Empty",

"value": "",

"message": "",

"subject_type": "UDPM-1",

"udf": "UDPM-2840"

}

],

"options": {

"obj_type": "SubjectType"

}

},

{

"name": "Request Glycomics",

"uuid": "UDPM-1454",

"input_type": "UDPM-1",

"output_type": "UDPM-244",

"flags": 0,

"options": {

"obj_type": "SubjectType",

"do_not_open_subject": true

}

},

{

"name": "Request Research",

"uuid": "UDPM-1455",

"input_type": "UDPM-1",

"output_type": "UDPM-271",

"flags": 0,

"before_code": "f = subj.get_value(\"UDP Cohort\")\n\nparams[:defaults] = {\n 'UDP Cohort'=> f\n}\n",

"after_code": "#subj.set_value('UDP Cohort', params['UDP Cohort'])",

"options": {

"obj_type": "SubjectType",

"do_not_open_subject": true

}

},

{

"name": "Request Fibroblast Testing",

"uuid": "UDPM-1456",

"input_type": "UDPM-1",

"output_type": "UDPM-167",

"flags": 0,

"before_code": "params[:defaults] = {\n 'Sample Type'=> 'Skin Fibroblast',\n 'Collaborating Center'=> find_subject(:subject_type=>'Collaborating Centers', :name=>'[redacted]')\n }",

"options": {

"obj_type": "SubjectType"

}

}

],

"show_options": {

"top": 20,

"left": 477.98333740234375

},

"end_task": true

},

{

"name": "End of Visit Checklist",

"uuid": "UDPM-1031",

"owner": "UDPM-67",

"managers": "UDPM-49",

"performers": "UDPM-49",

"tools": [

{

"name": "Checklist",

"uuid": "UDPM-1336",

"input_type": "UDPM-1",

"output_type": "UDPM-1",

"flags": 1,

"options": {

"udfs": [

"UDPM-2861",

"UDPM-2734"

]

}

},

{

"name": "Add Diagnosis",

"uuid": "UDPM-1342",

"input_type": "UDPM-1",

"output_type": "UDPM-185",

"flags": 0,

"options": {

"obj_type": "SubjectType"

}

},

{

"name": "PhenoTips Review",

"uuid": "UDPM-1343",

"input_type": "UDPM-1",

"output_type": "UDPM-205",

"flags": 0,

"options": {

"obj_type": "SubjectType"

}

},

{

"name": "Radiology Review",

"uuid": "UDPM-1344",

"input_type": "UDPM-1",

"output_type": "UDPM-246",

"flags": 0,

"before_code": "f = subj.get_value(\"Notes (PII)\")\n\nparams[:defaults] = {\n 'Notes'=> f\n}\n",

"options": {

"obj_type": "SubjectType"

}

},

{

"name": "Letter Request",

"uuid": "UDPM-1345",

"input_type": "UDPM-1",

"output_type": "UDPM-253",

"flags": 0,

"options": {

"obj_type": "SubjectType"

}

},

{

"name": "Create Initial Clinical Note",

"uuid": "UDPM-1442",

"input_type": "UDPM-1",

"output_type": "UDPM-267",

"flags": 0,

"hide_button_if_not_condition": true,

"conditions": [

{

"name": "Patient&rarr;Clinical Notes",

"condition": "Empty",

"value": "",

"message": "",

"subject_type": "UDPM-1",

"udf": "UDPM-2840"

}

],

"options": {

"obj_type": "SubjectType"

}

},

{

"name": "Upload Non-PII Files",

"uuid": "UDPM-1457",

"input_type": "UDPM-1",

"output_type": "UDPM-1",

"flags": 6,

"before_code": "require_script 'non_pii_upload_helper'\nbefore_upload(params)",

"after_code": "require_script 'non_pii_upload_helper'\nafter_upload(params, subj)"

},

{

"name": "Upload PII Files",

"uuid": "UDPM-1458",

"input_type": "UDPM-1",

"output_type": "UDPM-1",

"flags": 6,

"before_code": "require_script 'pii_upload_helper'\nbefore_upload(params)",

"after_code": "require_script 'pii_upload_helper'\nafter_upload(params, subj)"

}

],

"next_states": [

{

"state_name": "Seen",

"state_uuid": "UDPM-1025",

"btn_text": "Seen",

"btn_css": "rgb(13, 73, 123)",

"btn_scale": "large"

}

],

"show_options": {

"top": 579,

"left": 1099

}

},

{

"name": "Enrolled - Remote Workup - No UDP admission",

"uuid": "UDPM-603",

"owner": "UDPM-67",

"managers": "UDPM-49",

"performers": "UDPM-49",

"tools": [

{

"name": "Upload PII Files",

"uuid": "UDPM-1478",

"input_type": "UDPM-1",

"output_type": "UDPM-1",

"flags": 6,

"before_code": "require_script 'pii_upload_helper'\nbefore_upload(params)\n",

"after_code": "require_script 'pii_upload_helper'\nafter_upload(params, subj)\n"

},

{

"name": "Upload Non-PII Files",

"uuid": "UDPM-1477",

"input_type": "UDPM-1",

"output_type": "UDPM-1",

"flags": 6,

"before_code": "require_script 'non_pii_upload_helper'\nbefore_upload(params)\n",

"after_code": "require_script 'non_pii_upload_helper'\nafter_upload(params, subj)\n"

},

{

"name": "Update Patient",

"uuid": "UDPM-1018",

"input_type": "UDPM-1",

"output_type": "UDPM-1",

"flags": 1,

"options": {

"udfs": [

"UDPM-2861",

"UDPM-2862",

"UDPM-2863",

"UDPM-2316",

"UDPM-1979",

"UDPM-1807",

"UDPM-1722",

"UDPM-43",

"UDPM-28",

"UDPM-56",

"UDPM-15",

"UDPM-4",

"UDPM-11",

"UDPM-1",

"UDPM-2",

"UDPM-616",

"UDPM-3",

"UDPM-151",

"UDPM-1330",

"UDPM-485",

"UDPM-502",

"UDPM-2202",

"UDPM-562",

"UDPM-2117",

"UDPM-739",

"UDPM-505",

"UDPM-731",

"UDPM-857",

"UDPM-528",

"UDPM-730",

"UDPM-881",

"UDPM-52",

"UDPM-53",

"UDPM-184",

"UDPM-54",

"UDPM-190",

"UDPM-1654",

"UDPM-1655",

"UDPM-882",

"UDPM-1869",

"UDPM-109",

"UDPM-497",

"UDPM-499",

"UDPM-498",

"UDPM-2016",

"UDPM-48",

"UDPM-2290",

"UDPM-2204",

"UDPM-1495",

"UDPM-589",

"UDPM-484",

"UDPM-1225",

"UDPM-2059",

"UDPM-191",

"UDPM-182",

"UDPM-1497",

"UDPM-1496",

"UDPM-907",

"UDPM-2778",

"UDPM-613",

"UDPM-1492",

"UDPM-545"

]

}

},

{

"name": "Add Diagnosis",

"uuid": "UDPM-973",

"input_type": "UDPM-1",

"output_type": "UDPM-185",

"flags": 0,

"options": {

"obj_type": "SubjectType"

}

},

{

"name": "PhenoTips Review",

"uuid": "UDPM-1032",

"input_type": "UDPM-1",

"output_type": "UDPM-205",

"flags": 0,

"options": {

"obj_type": "SubjectType"

}

},

{

"name": "Create Initial Clinical Note",

"uuid": "UDPM-1433",

"input_type": "UDPM-1",

"output_type": "UDPM-267",

"flags": 0,

"hide_button_if_not_condition": true,

"conditions": [

{

"name": "Patient&rarr;Clinical Notes",

"condition": "Empty",

"value": "",

"message": "",

"subject_type": "UDPM-1",

"udf": "UDPM-2840"

}

],

"options": {

"obj_type": "SubjectType"

}

}

],

"next_states": [

{

"state_name": "Remote Workup Complete",

"state_uuid": "UDPM-856",

"btn_text": "Remote Workup Complete",

"btn_css": "#456"

}

],

"show_options": {

"top": 182.7166748046875,

"left": 421.5999755859375

}

},

{

"name": "Family Member",

"uuid": "UDPM-1024",

"owner": "UDPM-67",

"managers": "UDPM-49",

"performers": "UDPM-49",

"tools": [

{

"name": "Upload Non-PII Files",

"uuid": "UDPM-1481",

"input_type": "UDPM-1",

"output_type": "UDPM-1",

"flags": 6,

"before_code": "require_script 'non_pii_upload_helper'\nbefore_upload(params)\n",

"after_code": "require_script 'non_pii_upload_helper'\nafter_upload(params, subj)\n"

},

{

"name": "Upload PII Files",

"uuid": "UDPM-1482",

"input_type": "UDPM-1",

"output_type": "UDPM-1",

"flags": 6,

"before_code": "require_script 'pii_upload_helper'\nbefore_upload(params)\n",

"after_code": "require_script 'pii_upload_helper'\nafter_upload(params, subj)\n"

},

{

"name": "Update Patient",

"uuid": "UDPM-1293",

"input_type": "UDPM-1",

"output_type": "UDPM-1",

"flags": 1,

"options": {

"udfs": [

"UDPM-2861",

"UDPM-2862",

"UDPM-2863",

"UDPM-2316",

"UDPM-2490",

"UDPM-2597",

"UDPM-2491",

"UDPM-2492",

"UDPM-2596",

"UDPM-2493",

"UDPM-2494",

"UDPM-1807",

"UDPM-1722",

"UDPM-43",

"UDPM-28",

"UDPM-56",

"UDPM-15",

"UDPM-4",

"UDPM-11",

"UDPM-1",

"UDPM-2",

"UDPM-616",

"UDPM-3",

"UDPM-151",

"UDPM-1330",

"UDPM-502",

"UDPM-2202",

"UDPM-562",

"UDPM-739",

"UDPM-505",

"UDPM-731",

"UDPM-857",

"UDPM-528",

"UDPM-730",

"UDPM-881",

"UDPM-52",

"UDPM-53",

"UDPM-184",

"UDPM-54",

"UDPM-190",

"UDPM-1654",

"UDPM-1655",

"UDPM-882",

"UDPM-1869",

"UDPM-2016",

"UDPM-48",

"UDPM-2290",

"UDPM-2204",

"UDPM-1495",

"UDPM-589",

"UDPM-484",

"UDPM-1225",

"UDPM-2312",

"UDPM-2314",

"UDPM-2313",

"UDPM-2059",

"UDPM-191",

"UDPM-182",

"UDPM-1497",

"UDPM-1496",

"UDPM-218",

"UDPM-227",

"UDPM-1492",

"UDPM-1954",

"UDPM-545"

]

}

},

{

"name": "Change State",

"uuid": "UDPM-1292",

"input_type": "UDPM-1",

"output_type": "UDPM-1",

"flags": 6,

"after_code": "# Script to run After Tool is executed\nnext_state = params[\"patient_workflow_states\"]\nadvance_workflow(\"Patient Workflow\",next_state,subj)\nshow_message(\"Patient is now successfully placed at #{next_state} state\")",

"after_code_params": [

"UDPM-2284"

]

},

{

"name": "Create Initial Clinical Note",

"uuid": "UDPM-1432",

"input_type": "UDPM-1",

"output_type": "UDPM-267",

"flags": 0,

"options": {

"obj_type": "SubjectType"

}

}

],

"show_options": {

"top": 105,

"left": 991.75

},

"end_task": true

},

{

"name": "Inquiry - No Additional Information",

"uuid": "UDPM-958",

"owner": "UDPM-67",

"managers": "UDPM-49",

"performers": "UDPM-49",

"tools": [

{

"name": "Upload PII Files",

"uuid": "UDPM-1484",

"input_type": "UDPM-1",

"output_type": "UDPM-1",

"flags": 6,

"before_code": "require_script 'pii_upload_helper'\nbefore_upload(params)",

"after_code": "require_script 'pii_upload_helper'\nafter_upload(params, subj)"

},

{

"name": "Upload Non-PII FIles",

"uuid": "UDPM-1483",

"input_type": "UDPM-1",

"output_type": "UDPM-1",

"flags": 6,

"before_code": "require_script 'non_pii_upload_helper'\nbefore_upload(params)\n",

"after_code": "require_script 'non_pii_upload_helper'\nafter_upload(params, subj)\n"

},

{

"name": "Update Patient",

"uuid": "UDPM-1208",

"input_type": "UDPM-1",

"output_type": "UDPM-1",

"flags": 1,

"options": {

"udfs": [

"UDPM-2861",

"UDPM-2862",

"UDPM-2863",

"UDPM-2490",

"UDPM-2491",

"UDPM-2492",

"UDPM-2493",

"UDPM-2494",

"UDPM-1807",

"UDPM-1722",

"UDPM-43",

"UDPM-28",

"UDPM-56",

"UDPM-15",

"UDPM-4",

"UDPM-11",

"UDPM-1",

"UDPM-2",

"UDPM-616",

"UDPM-3",

"UDPM-580",

"UDPM-151",

"UDPM-1330",

"UDPM-485",

"UDPM-502",

"UDPM-2202",

"UDPM-562",

"UDPM-739",

"UDPM-505",

"UDPM-731",

"UDPM-857",

"UDPM-528",

"UDPM-730",

"UDPM-881",

"UDPM-52",

"UDPM-53",

"UDPM-184",

"UDPM-54",

"UDPM-190",

"UDPM-1654",

"UDPM-1655",

"UDPM-882",

"UDPM-1869",

"UDPM-2016",

"UDPM-48",

"UDPM-2290",

"UDPM-2059",

"UDPM-218",

"UDPM-227"

]

}

},

{

"name": "Change State",

"uuid": "UDPM-1209",

"input_type": "UDPM-1",

"output_type": "UDPM-1",

"flags": 6,

"after_code": "# Script to run After Tool is executed\nnext_state = params[\"patient_workflow_states\"]\nadvance_workflow(\"Patient Workflow\",next_state,subj)\nshow_message(\"Patient is now successfully placed at #{next_state} state\")",

"after_code_params": [

"UDPM-2284"

]

},

{

"name": "PhenoTips Review",

"uuid": "UDPM-1232",

"input_type": "UDPM-1",

"output_type": "UDPM-205",

"flags": 0,

"options": {

"obj_type": "SubjectType",

"do_not_open_subject": true

}

},

{

"name": "Create Initial Clinical Note",

"uuid": "UDPM-1431",

"input_type": "UDPM-1",

"output_type": "UDPM-267",

"flags": 0,

"hide_button_if_not_condition": true,

"conditions": [

{

"name": "Patient&rarr;Clinical Notes",

"condition": "Empty",

"value": "",

"message": "",

"subject_type": "UDPM-1",

"udf": "UDPM-2840"

}

],

"options": {

"obj_type": "SubjectType"

}

}

],

"show_options": {

"top": 23,

"left": 901.11669921875

},

"end_task": true

},

{

"name": "Referred - NIH Service",

"uuid": "UDPM-814",

"owner": "UDPM-67",

"managers": "UDPM-49",

"performers": "UDPM-49",

"tools": [

{

"name": "Update Patient",

"uuid": "UDPM-1106",

"input_type": "UDPM-1",

"output_type": "UDPM-1",

"flags": 1,

"options": {

"udfs": [

"UDPM-2861",

"UDPM-2862",

"UDPM-2863",

"UDPM-2316",

"UDPM-1979",

"UDPM-1807",

"UDPM-1722",

"UDPM-43",

"UDPM-28",

"UDPM-56",

"UDPM-15",

"UDPM-4",

"UDPM-11",

"UDPM-1",

"UDPM-2",

"UDPM-616",

"UDPM-3",

"UDPM-580",

"UDPM-151",

"UDPM-1330",

"UDPM-485",

"UDPM-502",

"UDPM-2202",

"UDPM-562",

"UDPM-739",

"UDPM-505",

"UDPM-731",

"UDPM-857",

"UDPM-528",

"UDPM-730",

"UDPM-881",

"UDPM-52",

"UDPM-53",

"UDPM-184",

"UDPM-54",

"UDPM-190",

"UDPM-1654",

"UDPM-1655",

"UDPM-882",

"UDPM-1869",

"UDPM-109",

"UDPM-497",

"UDPM-499",

"UDPM-498",

"UDPM-2016",

"UDPM-48",

"UDPM-2290",

"UDPM-2204",

"UDPM-1495",

"UDPM-589",

"UDPM-484",

"UDPM-2312",

"UDPM-182",

"UDPM-545"

]

}

},

{

"name": "Upload Non-PII Files",

"uuid": "UDPM-1491",

"input_type": "UDPM-1",

"output_type": "UDPM-1",

"flags": 6,

"before_code": "require_script 'non_pii_upload_helper'\nbefore_upload(params)\n",

"after_code": "require_script 'non_pii_upload_helper'\nafter_upload(params, subj)\n"

},

{

"name": "Upload PII Files",

"uuid": "UDPM-1492",

"input_type": "UDPM-1",

"output_type": "UDPM-1",

"flags": 6,

"before_code": "require_script 'pii_upload_helper'\nbefore_upload(params)",

"after_code": "require_script 'pii_upload_helper'\nafter_upload(params, subj)"

},

{

"name": "Change State",

"uuid": "UDPM-1075",

"input_type": "UDPM-1",

"output_type": "UDPM-1",

"description": "Changes Workflow State",

"flags": 6,

"after_code": "# Script to run After Tool is executed\nnext_state = params[\"patient_workflow_states\"]\nadvance_workflow(\"Patient Workflow\",next_state,subj)\nshow_message(\"Patient is now successfully placed at #{next_state} state\")",

"after_code_params": [

"UDPM-2284"

]

},

{

"name": "Create Task",

"uuid": "UDPM-1080",

"input_type": "UDPM-1",

"output_type": "UDPM-164",

"flags": 0,

"options": {

"obj_type": "SubjectType"

}

},

{

"name": "Add Diagnosis",

"uuid": "UDPM-1097",

"input_type": "UDPM-1",

"output_type": "UDPM-185",

"flags": 0,

"options": {

"obj_type": "SubjectType"

}

},

{

"name": "PhenoTips Review",

"uuid": "UDPM-1235",

"input_type": "UDPM-1",

"output_type": "UDPM-205",

"flags": 0,

"options": {

"obj_type": "SubjectType",

"do_not_open_subject": true

}

},

{

"name": "Create Initial Clinical Note",

"uuid": "UDPM-1427",

"input_type": "UDPM-1",

"output_type": "UDPM-267",

"flags": 0,

"hide_button_if_not_condition": true,

"conditions": [

{

"name": "Patient&rarr;Clinical Notes",

"condition": "Empty",

"value": "",

"message": "",

"subject_type": "UDPM-1",

"udf": "UDPM-2840"

}

],

"options": {

"obj_type": "SubjectType"

}

}

],

"show_options": {

"top": 62,

"left": 2.850006103515625

},

"end_task": true

},

{

"name": "Referred - Outside NIH",

"uuid": "UDPM-815",

"owner": "UDPM-67",

"managers": "UDPM-49",

"performers": "UDPM-49",

"tools": [

{

"name": "Upload PII Files",

"uuid": "UDPM-1490",

"input_type": "UDPM-1",

"output_type": "UDPM-1",

"flags": 6,

"before_code": "require_script 'pii_upload_helper'\nbefore_upload(params)\n",

"after_code": "require_script 'pii_upload_helper'\nafter_upload(params, subj)\n"

},

{

"name": "Upload Non-PII Files",

"uuid": "UDPM-1489",

"input_type": "UDPM-1",

"output_type": "UDPM-1",

"flags": 6,

"before_code": "require_script 'non_pii_upload_helper'\nbefore_upload(params)\n",

"after_code": "require_script 'non_pii_upload_helper'\nafter_upload(params, subj)\n"

},

{

"name": "Change State",

"uuid": "UDPM-1076",

"input_type": "UDPM-1",

"output_type": "UDPM-1",

"description": "Changes Workflow State",

"flags": 6,

"after_code": "# Script to run After Tool is executed\nnext_state = params[\"patient_workflow_states\"]\nadvance_workflow(\"Patient Workflow\",next_state,subj)\nshow_message(\"Patient is now successfully placed at #{next_state} state\")",

"after_code_params": [

"UDPM-2284"

]

},

{

"name": "Create Task",

"uuid": "UDPM-1081",

"input_type": "UDPM-1",

"output_type": "UDPM-164",

"flags": 0,

"options": {

"obj_type": "SubjectType"

}

},

{

"name": "Add Diagnosis",

"uuid": "UDPM-1101",

"input_type": "UDPM-1",

"output_type": "UDPM-185",

"flags": 0,

"options": {

"obj_type": "SubjectType"

}

},

{

"name": "Patient Update",

"uuid": "UDPM-1109",

"input_type": "UDPM-1",

"output_type": "UDPM-1",

"flags": 1,

"options": {

"udfs": [

"UDPM-2861",

"UDPM-2862",

"UDPM-2863",

"UDPM-2316",

"UDPM-1979",

"UDPM-1807",

"UDPM-1722",

"UDPM-43",

"UDPM-28",

"UDPM-56",

"UDPM-15",

"UDPM-4",

"UDPM-11",

"UDPM-1",

"UDPM-2",

"UDPM-616",

"UDPM-3",

"UDPM-580",

"UDPM-151",

"UDPM-1330",

"UDPM-485",

"UDPM-502",

"UDPM-2202",

"UDPM-562",

"UDPM-739",

"UDPM-505",

"UDPM-731",

"UDPM-857",

"UDPM-528",

"UDPM-730",

"UDPM-881",

"UDPM-52",

"UDPM-53",

"UDPM-184",

"UDPM-54",

"UDPM-190",

"UDPM-1654",

"UDPM-1655",

"UDPM-882",

"UDPM-1869",

"UDPM-109",

"UDPM-497",

"UDPM-499",

"UDPM-498",

"UDPM-2016",

"UDPM-48",

"UDPM-2290",

"UDPM-2204",

"UDPM-1495",

"UDPM-589",

"UDPM-484",

"UDPM-1225",

"UDPM-2312",

"UDPM-2059",

"UDPM-191",

"UDPM-182",

"UDPM-613",

"UDPM-1492",

"UDPM-545"

]

}

},

{

"name": "PhenoTips Review",

"uuid": "UDPM-1231",

"input_type": "UDPM-1",

"output_type": "UDPM-205",

"flags": 0,

"options": {

"obj_type": "SubjectType",

"do_not_open_subject": true

}

},

{

"name": "Create Initial Clinical Note",

"uuid": "UDPM-1428",

"input_type": "UDPM-1",

"output_type": "UDPM-267",

"flags": 0,

"hide_button_if_not_condition": true,

"conditions": [

{

"name": "Patient&rarr;Clinical Notes",

"condition": "Empty",

"value": "",

"message": "",

"subject_type": "UDPM-1",

"udf": "UDPM-2840"

}

],

"options": {

"obj_type": "SubjectType"

}

}

],

"show_options": {

"top": 13.716659545898438,

"left": 198.86666870117188

},

"end_task": true

},

{

"name": "Rejected",

"uuid": "UDPM-158",

"owner": "UDPM-67",

"managers": "UDPM-49",

"performers": "UDPM-5",

"tools": [

{

"name": "Upload PII File",

"uuid": "UDPM-1476",

"input_type": "UDPM-1",

"output_type": "UDPM-1",

"flags": 6,

"before_code": "require_script 'pii_upload_helper'\nbefore_upload(params)\n",

"after_code": "require_script 'pii_upload_helper'\nafter_upload(params, subj)\n\n"

},

{

"name": "Upload Non-PII Files",

"uuid": "UDPM-1475",

"input_type": "UDPM-1",

"output_type": "UDPM-1",

"flags": 6,

"before_code": "require_script 'non_pii_upload_helper'\nbefore_upload(params)\n",

"after_code": "require_script 'non_pii_upload_helper'\nafter_upload(params, subj)\n"

},

{

"name": "Rejection Notes",

"uuid": "UDPM-1060",

"input_type": "UDPM-1",

"output_type": "UDPM-1",

"flags": 1,

"options": {

"udfs": [

"UDPM-227"

]

}

},

{

"name": "Change State",

"uuid": "UDPM-1077",

"input_type": "UDPM-1",

"output_type": "UDPM-1",

"description": "Changes Workflow State",

"flags": 6,

"after_code": "# Script to run After Tool is executed\nnext_state = params[\"patient_workflow_states\"]\nadvance_workflow(\"Patient Workflow\",next_state,subj)\nshow_message(\"Patient is now successfully placed at #{next_state} state\")",

"after_code_params": [

"UDPM-2284"

]

},

{

"name": "Create Task",

"uuid": "UDPM-1082",

"input_type": "UDPM-1",

"output_type": "UDPM-164",

"flags": 0,

"options": {

"obj_type": "SubjectType"

}

},

{

"name": "Update Patient",

"uuid": "UDPM-1129",

"input_type": "UDPM-1",

"output_type": "UDPM-1",

"flags": 1,

"options": {

"udfs": [

"UDPM-2861",

"UDPM-2862",

"UDPM-2863",

"UDPM-2316",

"UDPM-1979",

"UDPM-1807",

"UDPM-1722",

"UDPM-43",

"UDPM-28",

"UDPM-56",

"UDPM-15",

"UDPM-4",

"UDPM-11",

"UDPM-1",

"UDPM-2",

"UDPM-616",

"UDPM-3",

"UDPM-580",

"UDPM-151",

"UDPM-1330",

"UDPM-485",

"UDPM-502",

"UDPM-2202",

"UDPM-562",

"UDPM-739",

"UDPM-505",

"UDPM-731",

"UDPM-857",

"UDPM-528",

"UDPM-730",

"UDPM-881",

"UDPM-52",

"UDPM-53",

"UDPM-184",

"UDPM-54",

"UDPM-190",

"UDPM-1654",

"UDPM-1655",

"UDPM-882",

"UDPM-1869",

"UDPM-109",

"UDPM-497",

"UDPM-499",

"UDPM-498",

"UDPM-2016",

"UDPM-48",

"UDPM-2290",

"UDPM-2204",

"UDPM-1495",

"UDPM-589",

"UDPM-484",

"UDPM-1225",

"UDPM-2312",

"UDPM-2314",

"UDPM-2313",

"UDPM-2059",

"UDPM-191",

"UDPM-182",

"UDPM-1497",

"UDPM-1496",

"UDPM-218",

"UDPM-227",

"UDPM-613",

"UDPM-1492",

"UDPM-1954",

"UDPM-545"

]

}

},

{

"name": "Create Initial Clinical Note",

"uuid": "UDPM-1437",

"input_type": "UDPM-1",

"output_type": "UDPM-267",

"flags": 0,

"hide_button_if_not_condition": true,

"conditions": [

{

"name": "Patient&rarr;Clinical Notes",

"condition": "Empty",

"value": "",

"message": "",

"subject_type": "UDPM-1",

"udf": "UDPM-2840"

}

],

"options": {

"obj_type": "SubjectType"

}

}

],

"show_options": {

"top": 657.2833251953125,

"left": 57.850006103515625

},

"end_task": true

},

{

"name": "Rejection Pending",

"uuid": "UDPM-157",

"owner": "UDPM-67",

"managers": "UDPM-49",

"performers": "UDPM-49",

"duration": 7.0,

"tools": [

{

"name": "Upload PII Files",

"uuid": "UDPM-1474",

"input_type": "UDPM-1",

"output_type": "UDPM-1",

"flags": 6,

"before_code": "require_script 'pii_upload_helper'\nbefore_upload(params)\n",

"after_code": "require_script 'pii_upload_helper'\nafter_upload(params, subj)\n"

},

{

"name": "Upload Non-PII Files",

"uuid": "UDPM-1473",

"input_type": "UDPM-1",

"output_type": "UDPM-1",

"flags": 6,

"before_code": "require_script 'non_pii_upload_helper'\nbefore_upload(params)\n",

"after_code": "require_script 'non_pii_upload_helper'\nafter_upload(params, subj)\n"

},

{

"name": "Compose Rejection Letter",

"uuid": "UDPM-380",

"input_type": "UDPM-1",

"output_type": "UDPM-85",

"flags": 0,

"options": {

"obj_type": "SubjectType"

}

},

{

"name": "Update Patient",

"uuid": "UDPM-1104",

"input_type": "UDPM-1",

"output_type": "UDPM-1",

"flags": 1,

"options": {

"udfs": [

"UDPM-2861",

"UDPM-2862",

"UDPM-2863",

"UDPM-2316",

"UDPM-1979",

"UDPM-1807",

"UDPM-1722",

"UDPM-43",

"UDPM-28",

"UDPM-56",

"UDPM-15",

"UDPM-4",

"UDPM-11",

"UDPM-1",

"UDPM-2",

"UDPM-616",

"UDPM-3",

"UDPM-580",

"UDPM-151",

"UDPM-1330",

"UDPM-485",

"UDPM-502",

"UDPM-2202",

"UDPM-562",

"UDPM-739",

"UDPM-505",

"UDPM-731",

"UDPM-857",

"UDPM-528",

"UDPM-730",

"UDPM-881",

"UDPM-52",

"UDPM-53",

"UDPM-184",

"UDPM-54",

"UDPM-190",

"UDPM-1654",

"UDPM-1655",

"UDPM-882",

"UDPM-1869",

"UDPM-109",

"UDPM-497",

"UDPM-499",

"UDPM-498",

"UDPM-2016",

"UDPM-48",

"UDPM-2290",

"UDPM-2204",

"UDPM-1495",

"UDPM-589",

"UDPM-484",

"UDPM-182",

"UDPM-613",

"UDPM-1492",

"UDPM-545"

]

}

},

{

"name": "Change State",

"uuid": "UDPM-1304",

"input_type": "UDPM-1",

"output_type": "UDPM-1",

"flags": 6,

"after_code": "# Script to run After Tool is executed\nnext_state = params[\"patient_workflow_states\"]\nadvance_workflow(\"Patient Workflow\",next_state,subj)\nshow_message(\"Patient is now successfully placed at #{next_state} state\")",

"after_code_params": [

"UDPM-2284"

]

},

{

"name": "Create Initial Clinical Note",

"uuid": "UDPM-1436",

"input_type": "UDPM-1",

"output_type": "UDPM-267",

"flags": 0,

"hide_button_if_not_condition": true,

"conditions": [

{

"name": "Patient&rarr;Clinical Notes",

"condition": "Empty",

"value": "",

"message": "",

"subject_type": "UDPM-1",

"udf": "UDPM-2840"

}

],

"options": {

"obj_type": "SubjectType"

}

}

],

"next_states": [

{

"state_name": "UDP Triage",

"state_uuid": "UDPM-159",

"btn_text": "Resurrect",

"btn_css": "#456",

"before_code": "# Script to run Before Transition is executed\nparams[:ask_next_performer] = true"

},

{

"state_name": "Rejected",

"state_uuid": "UDPM-158",

"btn_text": "Reject",

"btn_css": "#456"

}

],

"show_options": {

"top": 501.66668701171875,

"left": 15.850006103515625

}

},

{

"name": "Remote Workup Complete",

"uuid": "UDPM-856",

"owner": "UDPM-67",

"managers": "UDPM-49",

"performers": "UDPM-49",

"tools": [

{

"name": "Upload PII Files",

"uuid": "UDPM-1480",

"input_type": "UDPM-1",

"output_type": "UDPM-1",

"flags": 6,

"before_code": "require_script 'pii_upload_helper'\nbefore_upload(params)\n",

"after_code": "require_script 'pii_upload_helper'\nafter_upload(params, subj)\n"

},

{

"name": "Upload Non-PII Files",

"uuid": "UDPM-1479",

"input_type": "UDPM-1",

"output_type": "UDPM-1",

"flags": 6,

"before_code": "require_script 'non_pii_upload_helper'\nbefore_upload(params)\n",

"after_code": "require_script 'non_pii_upload_helper'\nafter_upload(params, subj)\n"

},

{

"name": "Patient Update",

"uuid": "UDPM-1111",

"input_type": "UDPM-1",

"output_type": "UDPM-1",

"flags": 1,

"options": {

"udfs": [

"UDPM-2861",

"UDPM-2862",

"UDPM-2863",

"UDPM-2316",

"UDPM-1979",

"UDPM-1807",

"UDPM-1722",

"UDPM-43",

"UDPM-28",

"UDPM-56",

"UDPM-15",

"UDPM-4",

"UDPM-11",

"UDPM-1",

"UDPM-2",

"UDPM-616",

"UDPM-3",

"UDPM-580",

"UDPM-151",

"UDPM-1330",

"UDPM-485",

"UDPM-502",

"UDPM-2202",

"UDPM-562",

"UDPM-739",

"UDPM-505",

"UDPM-731",

"UDPM-857",

"UDPM-528",

"UDPM-730",

"UDPM-881",

"UDPM-52",

"UDPM-53",

"UDPM-184",

"UDPM-54",

"UDPM-190",

"UDPM-1654",

"UDPM-1655",

"UDPM-882",

"UDPM-1869",

"UDPM-109",

"UDPM-497",

"UDPM-499",

"UDPM-498",

"UDPM-2016",

"UDPM-48",

"UDPM-2290",

"UDPM-2204",

"UDPM-1495",

"UDPM-589",

"UDPM-484",

"UDPM-182",

"UDPM-613",

"UDPM-1492",

"UDPM-545"

]

}

},

{

"name": "Change State",

"uuid": "UDPM-1087",

"input_type": "UDPM-1",

"output_type": "UDPM-1",

"description": "Changes Workflow State",

"flags": 6,

"after_code": "# Script to run After Tool is executed\nnext_state = params[\"patient_workflow_states\"]\nadvance_workflow(\"Patient Workflow\",next_state,subj)\nshow_message(\"Patient is now successfully placed at #{next_state} state\")",

"after_code_params": [

"UDPM-2284"

]

},

{

"name": "Add Diagnosis",

"uuid": "UDPM-1100",

"input_type": "UDPM-1",

"output_type": "UDPM-185",

"flags": 0,

"options": {

"obj_type": "SubjectType"

}

},

{

"name": "Create Initial Clinical Note",

"uuid": "UDPM-1434",

"input_type": "UDPM-1",

"output_type": "UDPM-267",

"flags": 0,

"hide_button_if_not_condition": true,

"conditions": [

{

"name": "Patient&rarr;Clinical Notes",

"condition": "Empty",

"value": "",

"message": "",

"subject_type": "UDPM-1",

"udf": "UDPM-2840"

}

],

"options": {

"obj_type": "SubjectType"

}

}

],

"show_options": {

"top": 206.4666748046875,

"left": 907.2166748046875

},

"end_task": true

},

{

"name": "Screening Meeting",

"uuid": "UDPM-163",

"owner": "UDPM-67",

"managers": "UDPM-49",

"performers": "UDPM-49",

"duration": 2.0,

"tools": [

{

"name": "Upload Non-PII FIles",

"uuid": "UDPM-1471",

"input_type": "UDPM-1",

"output_type": "UDPM-1",

"flags": 6,

"before_code": "require_script 'non_pii_upload_helper'\nbefore_upload(params)\n",

"after_code": "require_script 'non_pii_upload_helper'\nafter_upload(params, subj)\n"

},

{

"name": "Update Patient",

"uuid": "UDPM-1019",

"input_type": "UDPM-1",

"output_type": "UDPM-1",

"flags": 1,

"options": {

"udfs": [

"UDPM-2861",

"UDPM-2862",

"UDPM-2863",

"UDPM-2316",

"UDPM-1979",

"UDPM-1807",

"UDPM-1722",

"UDPM-43",

"UDPM-28",

"UDPM-56",

"UDPM-15",

"UDPM-4",

"UDPM-11",

"UDPM-1",

"UDPM-2",

"UDPM-616",

"UDPM-3",

"UDPM-580",

"UDPM-151",

"UDPM-1330",

"UDPM-485",

"UDPM-502",

"UDPM-2202",

"UDPM-562",

"UDPM-2117",

"UDPM-739",

"UDPM-505",

"UDPM-731",

"UDPM-857",

"UDPM-528",

"UDPM-730",

"UDPM-881",

"UDPM-52",

"UDPM-53",

"UDPM-184",

"UDPM-54",

"UDPM-190",

"UDPM-1654",

"UDPM-1655",

"UDPM-882",

"UDPM-1869",

"UDPM-109",

"UDPM-497",

"UDPM-499",

"UDPM-498",

"UDPM-2016",

"UDPM-48",

"UDPM-2290",

"UDPM-2204",

"UDPM-1495",

"UDPM-589",

"UDPM-484",

"UDPM-182",

"UDPM-907",

"UDPM-2778",

"UDPM-613",

"UDPM-1492",

"UDPM-1954",

"UDPM-545"

]

}

},

{

"name": "Upload PII Files",

"uuid": "UDPM-1472",

"input_type": "UDPM-1",

"output_type": "UDPM-1",

"flags": 6,

"before_code": "require_script 'pii_upload_helper'\nbefore_upload(params)\n",

"after_code": "require_script 'pii_upload_helper'\nafter_upload(params, subj)\n"

},

{

"name": "Question to Primary Care/Family",

"uuid": "UDPM-1547",

"input_type": "UDPM-1",

"output_type": "UDPM-84",

"flags": 0,

"options": {

"obj_type": "SubjectType"

}

},

{

"name": "Add Diagnosis",

"uuid": "UDPM-971",

"input_type": "UDPM-1",

"output_type": "UDPM-185",

"flags": 0,

"options": {

"obj_type": "SubjectType"

}

},

{

"name": "PhenoTips Review",

"uuid": "UDPM-1034",

"input_type": "UDPM-1",

"output_type": "UDPM-205",

"flags": 0,

"options": {

"obj_type": "SubjectType"

}

},

{

"name": "Request Review",

"uuid": "UDPM-1091",

"input_type": "UDPM-1",

"output_type": "UDPM-83",

"flags": 0,

"before_code": "f = subj.get_value(\"Notes (PII)\")\n\nparams[:defaults] = {\n 'Clinical Questions'=> f\n}\n",

"options": {

"obj_type": "SubjectType"

}

},

{

"name": "Change State",

"uuid": "UDPM-1305",

"input_type": "UDPM-1",

"output_type": "UDPM-1",

"flags": 6,

"after_code": "# Script to run After Tool is executed\nnext_state = params[\"patient_workflow_states\"]\nadvance_workflow(\"Patient Workflow\",next_state,subj)\nshow_message(\"Patient is now successfully placed at #{next_state} state\")",

"after_code_params": [

"UDPM-2284"

]

},

{

"name": "Radiology Review",

"uuid": "UDPM-1326",

"input_type": "UDPM-1",

"output_type": "UDPM-246",

"flags": 0,

"before_code": "f = subj.get_value(\"Notes (PII)\")\n\nparams[:defaults] = {\n 'Notes'=> f\n}\n",

"options": {

"obj_type": "SubjectType"

}

},

{

"name": "Letter Request",

"uuid": "UDPM-1346",

"input_type": "UDPM-1",

"output_type": "UDPM-253",

"flags": 0,

"options": {

"obj_type": "SubjectType"

}

},

{

"name": "Create Initial Clinical Note",

"uuid": "UDPM-1438",

"input_type": "UDPM-1",

"output_type": "UDPM-267",

"flags": 0,

"hide_button_if_not_condition": true,

"conditions": [

{

"name": "Patient&rarr;Clinical Notes",

"condition": "Empty",

"value": "",

"message": "",

"subject_type": "UDPM-1",

"udf": "UDPM-2840"

}

],

"options": {

"obj_type": "SubjectType"

}

}

],

"next_states": [

{

"state_name": "Seen",

"state_uuid": "UDPM-1025",

"btn_text": "Seen",

"btn_css": "#456"

},

{

"state_name": "Enrolled - Remote Workup - No UDP admission",

"state_uuid": "UDPM-603",

"btn_text": "Remote Accept - No Admit",

"btn_css": "#456",

"after_code": "self.next_state_performer = User.curr_user",

"before_code": "# Script to run Before Transition is executed\nparams[:ask_next_performer] = true"

},

{

"state_name": "Rejection Pending",

"state_uuid": "UDPM-157",

"btn_text": "Reject",

"btn_css": "#456",

"after_code": "self.next_state_performer = User.curr_user",

"before_code": "# Script to run Before Transition is executed\nparams[:ask_next_performer] = true"

},

{

"state_name": "Accepted Plan Visit",

"state_uuid": "UDPM-164",

"btn_text": "Accept/Schedule",

"btn_css": "#456",

"before_code": "# Script to run Before Transition is executed\nparams[:ask_next_performer] = true",

"btn_scale": "large"

}

],

"show_options": {

"top": 513.5333251953125,

"left": 262.683349609375

}

},

{

"name": "Seen",

"uuid": "UDPM-1025",

"owner": "UDPM-67",

"managers": "UDPM-49",

"performers": "UDPM-49",

"tools": [

{

"name": "Initial Seen Date",

"uuid": "UDPM-1404",

"input_type": "UDPM-1",

"output_type": "UDPM-1",

"flags": 1,

"options": {

"udfs": [

"UDPM-545"

]

}

},

{

"name": "Upload Non-PII Files",

"uuid": "UDPM-1463",

"input_type": "UDPM-1",

"output_type": "UDPM-1",

"flags": 6,

"before_code": "require_script 'non_pii_upload_helper'\nbefore_upload(params)",

"after_code": "require_script 'non_pii_upload_helper'\nbefore_upload(params)"

},

{

"name": "Update Patient",

"uuid": "UDPM-1374",

"input_type": "UDPM-1",

"output_type": "UDPM-1",

"flags": 1,

"options": {

"udfs": [

"UDPM-2861",

"UDPM-2862",

"UDPM-2863",

"UDPM-1807",

"UDPM-1722",

"UDPM-43",

"UDPM-28",

"UDPM-56",

"UDPM-15",

"UDPM-4",

"UDPM-11",

"UDPM-1",

"UDPM-2",

"UDPM-616",

"UDPM-3",

"UDPM-151",

"UDPM-1330",

"UDPM-502",

"UDPM-565",

"UDPM-2202",

"UDPM-562",

"UDPM-739",

"UDPM-505",

"UDPM-731",

"UDPM-857",

"UDPM-528",

"UDPM-730",

"UDPM-881",

"UDPM-52",

"UDPM-53",

"UDPM-184",

"UDPM-54",

"UDPM-190",

"UDPM-1654",

"UDPM-1655",

"UDPM-882",

"UDPM-1869",

"UDPM-2016",

"UDPM-48",

"UDPM-2290",

"UDPM-2204",

"UDPM-1495",

"UDPM-545",

"UDPM-2733"

]

}

},

{

"name": "Upload PII Files",

"uuid": "UDPM-1464",

"input_type": "UDPM-1",

"output_type": "UDPM-1",

"flags": 6,

"before_code": "require_script 'pii_upload_helper'\nbefore_upload(params)\n",

"after_code": "require_script 'pii_upload_helper'\nafter_upload(params, subj)\n"

},

{

"name": "Patient Research Meeting",

"uuid": "UDPM-1532",

"input_type": "UDPM-1",

"output_type": "UDPM-127",

"flags": 0,

"options": {

"obj_type": "SubjectType"

}

},

{

"name": "Change State",

"uuid": "UDPM-1301",

"input_type": "UDPM-1",

"output_type": "UDPM-1",

"flags": 6,

"after_code": "# Script to run After Tool is executed\nnext_state = params[\"patient_workflow_states\"]\nadvance_workflow(\"Patient Workflow\",next_state,subj)\nshow_message(\"Patient is now successfully placed at #{next_state} state\")",

"after_code_params": [

"UDPM-2284"

]

},

{

"name": "Add Diagnosis",

"uuid": "UDPM-1332",

"input_type": "UDPM-1",

"output_type": "UDPM-185",

"flags": 0,

"options": {

"obj_type": "SubjectType"

}

},

{

"name": "Radiology Review",

"uuid": "UDPM-1375",

"input_type": "UDPM-1",

"output_type": "UDPM-246",

"flags": 0,

"before_code": "f = subj.get_value(\"Notes (PII)\")\n\nparams[:defaults] = {\n 'Notes'=> f\n}\n",

"options": {

"obj_type": "SubjectType"

}

},

{

"name": "Request PhenoTips Review",

"uuid": "UDPM-1376",

"input_type": "UDPM-1",

"output_type": "UDPM-205",

"flags": 0,

"hide_button_if_not_condition": true,

"conditions": [

{

"name": "Patient&rarr;Phenotips Review",

"condition": "Empty",

"value": "",

"message": "",

"subject_type": "UDPM-1",

"udf": "UDPM-2662"

}

],

"options": {

"obj_type": "SubjectType",

"do_not_open_subject": true

}

},

{

"name": "Follow Up Visits",

"uuid": "UDPM-1403",

"input_type": "UDPM-1",

"output_type": "UDPM-264",

"flags": 0,

"options": {

"obj_type": "SubjectType"

}

},

{

"name": "Create Initial Clinical Note",

"uuid": "UDPM-1435",

"input_type": "UDPM-1",

"output_type": "UDPM-267",

"flags": 0,

"hide_button_if_not_condition": true,

"conditions": [

{

"name": "Patient&rarr;Clinical Notes",

"condition": "Empty",

"value": "",

"message": "",

"subject_type": "UDPM-1",

"udf": "UDPM-2840"

}

],

"options": {

"obj_type": "SubjectType"

}

}

],

"show_options": {

"top": 309,

"left": 1072

},

"end_task": true

},

{

"name": "UDP Triage",

"uuid": "UDPM-159",

"owner": "UDPM-67",

"managers": "UDPM-49",

"performers": "UDPM-49",

"duration": 21.0,

"tools": [

{

"name": "Upload PII Files",

"uuid": "UDPM-1461",

"input_type": "UDPM-1",

"output_type": "UDPM-1",

"flags": 6,

"before_code": "require_script 'pii_upload_helper'\nbefore_upload(params)",

"after_code": "require_script 'pii_upload_helper'\nafter_upload(params, subj)"

},

{

"name": "Update Patient",

"uuid": "UDPM-410",

"input_type": "UDPM-1",

"output_type": "UDPM-1",

"flags": 1,

"options": {

"udfs": [

"UDPM-2861",

"UDPM-2862",

"UDPM-2863",

"UDPM-2316",

"UDPM-1979",

"UDPM-2490",

"UDPM-1807",

"UDPM-1722",

"UDPM-43",

"UDPM-28",

"UDPM-56",

"UDPM-15",

"UDPM-4",

"UDPM-11",

"UDPM-1",

"UDPM-2",

"UDPM-616",

"UDPM-3",

"UDPM-580",

"UDPM-151",

"UDPM-1330",

"UDPM-485",

"UDPM-502",

"UDPM-2202",

"UDPM-562",

"UDPM-739",

"UDPM-505",

"UDPM-731",

"UDPM-857",

"UDPM-528",

"UDPM-730",

"UDPM-881",

"UDPM-52",

"UDPM-53",

"UDPM-645",

"UDPM-184",

"UDPM-54",

"UDPM-190",

"UDPM-1654",

"UDPM-1655",

"UDPM-882",

"UDPM-1869",

"UDPM-109",

"UDPM-497",

"UDPM-499",

"UDPM-498",

"UDPM-2016",

"UDPM-48",

"UDPM-2290",

"UDPM-2204",

"UDPM-1495",

"UDPM-589",

"UDPM-484",

"UDPM-2059",

"UDPM-182",

"UDPM-907",

"UDPM-2778",

"UDPM-613",

"UDPM-1492",

"UDPM-545"

]

}

},

{

"name": "Upload Non-PII Files",

"uuid": "UDPM-1462",

"input_type": "UDPM-1",

"output_type": "UDPM-1",

"flags": 6,

"before_code": "require_script 'non_pii_upload_helper'\nbefore_upload(params)",

"after_code": "require_script 'non_pii_upload_helper'\nafter_upload(params, subj)"

},

{

"name": "Patient Research Meeting",

"uuid": "UDPM-1531",

"input_type": "UDPM-1",

"output_type": "UDPM-127",

"flags": 0,

"options": {

"obj_type": "SubjectType"

}

},

{

"name": "Question to Primary Care/Family",

"uuid": "UDPM-1546",

"input_type": "UDPM-1",

"output_type": "UDPM-84",

"flags": 0,

"options": {

"obj_type": "SubjectType"

}

},

{

"name": "Request Review",

"uuid": "UDPM-371",

"input_type": "UDPM-1",

"output_type": "UDPM-83",

"flags": 0,

"before_code": "f = subj.get_value(\"Notes (PII)\")\n\nparams[:defaults] = {\n 'Clinical Questions'=> f\n}\n",

"options": {

"obj_type": "SubjectType"

}

},

{

"name": "Sort/Scan",

"uuid": "UDPM-413",

"input_type": "UDPM-1",

"output_type": "UDPM-88",

"flags": 0,

"options": {

"obj_type": "SubjectType"

}

},

{

"name": "PhenoTips Review",

"uuid": "UDPM-1030",

"input_type": "UDPM-1",

"output_type": "UDPM-205",

"flags": 0,

"options": {

"obj_type": "SubjectType"

}

},

{

"name": "Add Diagnosis",

"uuid": "UDPM-1094",

"input_type": "UDPM-1",

"output_type": "UDPM-185",

"flags": 0,

"options": {

"obj_type": "SubjectType"

}

},

{

"name": "Change State",

"uuid": "UDPM-1306",

"input_type": "UDPM-1",

"output_type": "UDPM-1",

"flags": 6,

"after_code": "# Script to run After Tool is executed\nnext_state = params[\"patient_workflow_states\"]\nadvance_workflow(\"Patient Workflow\",next_state,subj)\nshow_message(\"Patient is now successfully placed at #{next_state} state\")",

"after_code_params": [

"UDPM-2284"

]

},

{

"name": "Radiology Review",

"uuid": "UDPM-1316",

"input_type": "UDPM-1",

"output_type": "UDPM-246",

"flags": 0,

"before_code": "f = subj.get_value(\"Notes (PII)\")\n\nparams[:defaults] = {\n 'Notes'=> f\n}\n",

"options": {

"obj_type": "SubjectType"

}

},

{

"name": "Letter Request",

"uuid": "UDPM-1333",

"input_type": "UDPM-1",

"output_type": "UDPM-253",

"flags": 0,

"options": {

"obj_type": "SubjectType"

}

},

{

"name": "Create Initial Clinical Note",

"uuid": "UDPM-1426",

"input_type": "UDPM-1",

"output_type": "UDPM-267",

"description": "Use to set up clinical note",

"flags": 0,

"hide_button_if_not_condition": true,

"conditions": [

{

"name": "Patient&rarr;Clinical Notes",

"condition": "Empty",

"value": "",

"message": "",

"subject_type": "UDPM-1",

"udf": "UDPM-2840"

}

],

"options": {

"obj_type": "SubjectType",

"do_not_open_subject": true

}

}

],

"next_states": [

{

"state_name": "Category - Not Assigned",

"state_uuid": "UDPM-862",

"btn_text": "Category Unassigned",

"btn_css": "#456"

},

{

"state_name": "Screening Meeting",

"state_uuid": "UDPM-163",

"btn_text": "Screening Meeting",

"btn_css": "#456",

"after_code": "self.next_state_performer = find_user('[redacted]')",

"before_code": "# Script to run Before Transition is executed\nparams[:ask_next_performer] = true",

"hide_button_if_not_condition": true

},

{

"state_name": "Enrolled - Remote Workup - No UDP admission",

"state_uuid": "UDPM-603",

"btn_text": "Remote Accept - No Admit",

"btn_css": "#456",

"after_code": "self.next_state_performer = User.curr_user",

"before_code": "# Script to run Before Transition is executed\nparams[:ask_next_performer] = true"

},

{

"state_name": "Seen",

"state_uuid": "UDPM-1025",

"btn_text": "Seen",

"btn_css": "#456"

},

{

"state_name": "Rejection Pending",

"state_uuid": "UDPM-157",

"btn_text": "Reject",

"btn_css": "#456",

"before_code": "# Script to run Before Transition is executed\nparams[:ask_next_performer] = true"

},

{

"state_name": "Accepted Plan Visit",

"state_uuid": "UDPM-164",

"btn_text": "Accept/Schedule",

"btn_css": "#456",

"before_code": "# Script to run Before Transition is executed\nparams[:ask_next_performer] = true"

},

{

"state_name": "Referred - Outside NIH",

"state_uuid": "UDPM-815",

"btn_text": "Refer - Outside NIH",

"btn_css": "#456"

},

{

"state_name": "Referred - NIH Service",

"state_uuid": "UDPM-814",

"btn_text": "Refer - NIH",

"btn_css": "#456"

},

{

"state_name": "Cohort Sample Only",

"state_uuid": "UDPM-956",

"btn_text": "Cohort/Sample Management Only",

"btn_css": "#456",

"after_code": "subj.set_value('UDP Cohort Name', params['UDP Cohort Name'])",

"btn_scale": "large",

"after_code_params": [

"UDPM-2894"

],

"hide_button_if_not_condition": true,

"conditions": [

{

"name": "Patient&rarr;Cohort/Sample Management Only",

"condition": "=",

"value": "Yes",

"message": "",

"subject_type": "UDPM-1",

"udf": "UDPM-2490"

}

]

}

],

"show_options": {

"top": 264.7166748046875,

"left": 10.5

}

}

],

"entry_point": "UDPM-159",

"ask_performer": true

},{

"name": "Urine Pellet Workflow",

"uuid": "UDPM-144",

"subject_type": "UDPM-170",

"state_defs": [

{

"name": "Discard",

"uuid": "UDPM-957",

"owner": "UDPM-67",

"managers": "UDPM-22",

"performers": "UDPM-69",

"show_options": {

"top": 199,

"left": 122

},

"end_task": true

},

{

"name": "Processing",

"uuid": "UDPM-668",

"owner": "UDPM-67",

"managers": "UDPM-22",

"performers": "UDPM-69",

"duration": 1.0,

"next_states": [

{

"state_name": "Discard",

"state_uuid": "UDPM-957",

"btn_text": "Discard",

"btn_css": "#456",

"after_code": "subj.set_value('Rationale/Reasoning', params['Rationale/Reasoning'])",

"btn_scale": "large",

"after_code_params": [

"UDPM-883"

]

},

{

"state_name": "Stored",

"state_uuid": "UDPM-669",

"btn_text": "Store Pellet",

"btn_css": "#456",

"after_code": "require_script 'lims_helper'\n\nvial_count = params['Number of Vials to Store']\nself.next_state_performer = User.curr_user\nsubmit_to_fpro(subj, 'Urine Pellet', '7000000483', vial_count)",

"after_code_params": [

"UDPM-1450"

]

}

],

"show_options": {

"top": 55,

"left": 111

}

},

{

"name": "Stored",

"uuid": "UDPM-669",

"owner": "UDPM-67",

"managers": "UDPM-22",

"performers": "UDPM-69",

"show_options": {

"top": 57,

"left": 337

},

"end_task": true

}

],

"entry_point": "UDPM-668",

"ask_performer": true

},{

"name": "Exome Sequencing",

"uuid": "UDPM-135",

"subject_type": "UDPM-161",

"state_defs": [

{

"name": "Approved for Submission",

"uuid": "UDPM-766",

"owner": "UDPM-67",

"managers": "UDPM-44",

"performers": "UDPM-44",

"next_states": [

{

"state_name": "Cancelled",

"state_uuid": "UDPM-647",

"btn_text": "Cancel",

"btn_css": "#456",

"after_code_params": [

"UDPM-883"

]

},

{

"state_name": "Make DNA Aliquot",

"state_uuid": "UDPM-615",

"btn_text": "Prepare DNA for Shipment",

"btn_css": "#456",

"after_code": "self.next_state_performer = find_user('[redacted]')\n\nsubj.set_value(\"Sequencing center\", params['Sequencing center'])",

"btn_scale": "large",

"after_code_params": [

"UDPM-2039"

]

}

],

"show_options": {

"top": 396,

"left": 60

}

},

{

"name": "Cancelled",

"uuid": "UDPM-647",

"owner": "UDPM-67",

"managers": "UDPM-22",

"performers": "UDPM-25",

"show_options": {

"top": 117,

"left": 554

},

"end_task": true

},

{

"name": "DNA Ready for Shipment",

"uuid": "UDPM-616",

"owner": "UDPM-67",

"managers": "UDPM-42",

"performers": "UDPM-42",

"tools": [

{

"name": "Shipping Information",

"uuid": "UDPM-766",

"input_type": "UDPM-161",

"output_type": "UDPM-161",

"description": "DNA for Exome",

"flags": 1,

"options": {

"udfs": [

"UDPM-2035",

"UDPM-349",

"UDPM-191",

"UDPM-309"

]

}

}

],

"next_states": [

{

"state_name": "Cancelled",

"state_uuid": "UDPM-647",

"btn_text": "Cancel",

"btn_css": "#456",

"after_code_params": [

"UDPM-883"

]

},

{

"state_name": "DNA Sent for Exome",

"state_uuid": "UDPM-617",

"btn_text": "DNA Sent",

"btn_css": "#456",

"after_code": "subj.set_value('Date DNA Sent for Exome', params['Date DNA Sent for Exome'] )",

"after_code_params": [

"UDPM-349"

]

}

],

"em_performer_new_job": true,

"show_options": {

"top": 479,

"left": 639.2666625976562

}

},

{

"name": "DNA Received at Sequencing Center",

"uuid": "UDPM-793",

"owner": "UDPM-67",

"managers": "UDPM-27",

"performers": "UDPM-3",

"duration": 156.0,

"tools": [

{

"name": "Additional Information",

"uuid": "UDPM-993",

"input_type": "UDPM-161",

"output_type": "UDPM-161",

"description": "Axeq QC",

"flags": 1,

"options": {

"udfs": [

"UDPM-1381",

"UDPM-191",

"UDPM-2065",

"UDPM-2070",

"UDPM-2071",

"UDPM-2768",

"UDPM-2769"

]

}

}

],

"next_states": [

{

"state_name": "Cancelled",

"state_uuid": "UDPM-647",

"btn_text": "Cancel",

"btn_css": "rgb(13, 73, 123)",

"after_code": "subj.set_value(\"Rationale/Reasoning\", params['Rationale/Reasoning'])",

"btn_scale": "large",

"after_code_params": [

"UDPM-883"

]

},

{

"state_name": "Lane Files Received",

"state_uuid": "UDPM-767",

"btn_text": "Lane Files Received",

"btn_css": "#456"

}

],

"show_options": {

"top": 45.58332824707031,

"left": 886.816650390625

}

},

{

"name": "DNA Sent for Exome",

"uuid": "UDPM-617",

"owner": "UDPM-67",

"managers": "UDPM-27",

"performers": "UDPM-3",

"duration": 14.0,

"next_states": [

{

"state_name": "DNA Received at Sequencing Center",

"state_uuid": "UDPM-793",

"btn_text": "Samples Received at Sequencing Center",

"btn_css": "#456",

"after_code": "self.next_state_performer = find_user('[redacted]')",

"btn_scale": "large"

}

],

"show_options": {

"top": 314,

"left": 811.36669921875

}

},

{

"name": "Exome Wait List",

"uuid": "UDPM-614",

"owner": "UDPM-67",

"managers": "UDPM-44",

"performers": "UDPM-44",

"tools": [

{

"name": "Additional Information",

"uuid": "UDPM-764",

"input_type": "UDPM-161",

"output_type": "UDPM-161",

"flags": 1,

"options": {

"udfs": [

"UDPM-6",

"UDPM-2039"

]

}

}

],

"next_states": [

{

"state_name": "Hold for Decision",

"state_uuid": "UDPM-780",

"btn_text": "Place on Hold",

"btn_css": "#456",

"after_code_params": [

"UDPM-883"

]

},

{

"state_name": "Cancelled",

"state_uuid": "UDPM-647",

"btn_text": "Cancel",

"btn_css": "#456",

"after_code_params": [

"UDPM-883"

]

},

{

"state_name": "Approved for Submission",

"state_uuid": "UDPM-766",

"btn_text": "Approve for Submission",

"btn_css": "#456",

"after_code": "self.next_state_performer = find_user('[redacted]')",

"btn_scale": "large"

}

],

"show_options": {

"top": 16,

"left": 17

}

},

{

"name": "Hold for Decision",

"uuid": "UDPM-780",

"owner": "UDPM-67",

"managers": "UDPM-9",

"performers": "UDPM-2",

"duration": 30.0,

"next_states": [

{

"state_name": "Exome Wait List",

"state_uuid": "UDPM-614",

"btn_text": "Return to Wait List",

"btn_css": "#456"

},

{

"state_name": "Cancelled",

"state_uuid": "UDPM-647",

"btn_text": "Cancel",

"btn_css": "#456"

}

],

"show_options": {

"top": 192.9943084716797,

"left": 169.9801025390625

}

},

{

"name": "Lane Files Received",

"uuid": "UDPM-767",

"owner": "UDPM-67",

"managers": "UDPM-27",

"performers": "UDPM-3",

"tools": [

{

"name": "Create Exome Analysis",

"uuid": "UDPM-1544",

"input_type": "UDPM-161",

"output_type": "UDPM-60",

"flags": 0,

"options": {

"obj_type": "SubjectType"

}

},

{

"name": "Alignment Information",

"uuid": "UDPM-949",

"input_type": "UDPM-161",

"output_type": "UDPM-208",

"flags": 0,

"options": {

"obj_type": "SubjectType"

}

},

{

"name": "Lane File Information",

"uuid": "UDPM-950",

"input_type": "UDPM-161",

"output_type": "UDPM-161",

"flags": 1,

"options": {

"udfs": [

"UDPM-1681",

"UDPM-877",

"UDPM-371",

"UDPM-1958",

"UDPM-2873",

"UDPM-2874"

]

}

},

{

"name": "QC Results",

"uuid": "UDPM-1282",

"input_type": "UDPM-161",

"output_type": "UDPM-161",

"flags": 1,

"options": {

"udfs": [

"UDPM-2672",

"UDPM-2671",

"UDPM-2673",

"UDPM-2676",

"UDPM-2675"

]

}

},

{

"name": "Add Bioinformatics File",

"uuid": "UDPM-1423",

"input_type": "UDPM-161",

"output_type": "UDPM-235",

"flags": 0,

"options": {

"obj_type": "SubjectType"

}

}

],

"show_options": {

"top": 376.58331298828125,

"left": 1045.63330078125

},

"end_task": true

},

{

"name": "Make DNA Aliquot",

"uuid": "UDPM-615",

"owner": "UDPM-67",

"managers": "UDPM-22",

"performers": "UDPM-42",

"tools": [

{

"name": "1) DNA Information",

"uuid": "UDPM-765",

"input_type": "UDPM-161",

"output_type": "UDPM-161",

"flags": 1,

"options": {

"udfs": [

"UDPM-875",

"UDPM-451",

"UDPM-1382",

"UDPM-1342",

"UDPM-1378",

"UDPM-2040",

"UDPM-2041",

"UDPM-1384",

"UDPM-2482"

]

}

}

],

"next_states": [

{

"state_name": "Cancelled",

"state_uuid": "UDPM-647",

"btn_text": "Cancel",

"btn_css": "#456",

"after_code_params": [

"UDPM-883"

]

},

{

"state_name": "DNA Ready for Shipment",

"state_uuid": "UDPM-616",

"btn_text": "2) DNA Prepared",

"btn_css": "#456",

"after_code": "self.next_state_performer = find_user('[redacted]')"

}

],

"show_options": {

"top": 501.99998474121094,

"left": 367.2556457519531

}

}

],

"entry_point": "UDPM-614",

"ask_performer": true,

"show_udfs": "UDPM-1965"

},{

"name": "Requisition",

"uuid": "UDPM-141",

"subject_type": "UDPM-167",

"state_defs": [

{

"name": "Cancel Requisition",

"uuid": "UDPM-1077",

"owner": "UDPM-67",

"managers": "UDPM-9",

"performers": "UDPM-49",

"show_options": {

"top": 104,

"left": 687

},

"end_task": true

},

{

"name": "Complete",

"uuid": "UDPM-657",

"owner": "UDPM-67",

"managers": "UDPM-9",

"performers": "UDPM-31",

"duration": 30.0,

"show_options": {

"top": 434.984375,

"left": 690.984375

},

"end_task": true

},

{

"name": "POTS",

"uuid": "UDPM-652",

"owner": "UDPM-67",

"managers": "UDPM-9",

"performers": "UDPM-40",

"duration": 10.0,

"tools": [

{

"name": "POTS Entry",

"uuid": "UDPM-828",

"input_type": "UDPM-167",

"output_type": "UDPM-167",

"flags": 1,

"options": {

"udfs": [

"UDPM-1752",

"UDPM-1753"

]

}

}

],

"next_states": [

{

"state_name": "Ready for Shipment",

"state_uuid": "UDPM-821",

"btn_text": "Entry Complete",

"btn_css": "#456",

"after_code": "# Script to run After Transition is executed\nself.next_state_performer = find_user('[redacted]')"

}

],

"em_performer_new_job": true,

"show_options": {

"top": 46,

"left": 474

}

},

{

"name": "Prepare Samples",

"uuid": "UDPM-869",

"owner": "UDPM-67",

"managers": "UDPM-9",

"performers": "UDPM-54",

"duration": 10.0,

"next_states": [

{

"state_name": "Return Requisition",

"state_uuid": "UDPM-927",

"btn_text": "Return Requisition to Requestor",

"btn_css": "#456",

"after_code": "subj.set_value('Comments', params['Comments'])\nsend_email(subj.created_by, find_email_template(\"Requisition Return\"), subj)\nself.next_state_performer = subj.created_by",

"btn_scale": "large",

"after_code_params": [

"UDPM-5"

]

},

{

"state_name": "Ready for Shipment",

"state_uuid": "UDPM-821",

"btn_text": "Ready for Shipment",

"btn_css": "#456"

}

],

"show_options": {

"top": 273.421875,

"left": 588

}

},

{

"name": "Ready for Shipment",

"uuid": "UDPM-821",

"owner": "UDPM-67",

"managers": "UDPM-46",

"performers": "UDPM-46",

"tools": [

{

"name": "Shipment Information",

"uuid": "UDPM-1020",

"input_type": "UDPM-167",

"output_type": "UDPM-167",

"flags": 1,

"options": {

"udfs": [

"UDPM-1672",

"UDPM-309",

"UDPM-1515",

"UDPM-1797"

]

}

}

],

"next_states": [

{

"state_name": "Results Received",

"state_uuid": "UDPM-717",

"btn_text": "Requisition Submitted",

"btn_css": "rgb(13, 73, 123)",

"after_code": "self.next_state_performer = find_user('[redacted]')",

"btn_scale": "large"

},

{

"state_name": "Prepare Samples",

"state_uuid": "UDPM-869",

"btn_text": "To Laboratory for Sample Preparation",

"btn_css": "#456",

"btn_scale": "large"

}

],

"show_options": {

"top": 419,

"left": 185

}

},

{

"name": "Requisition Processing",

"uuid": "UDPM-651",

"owner": "UDPM-67",

"managers": "UDPM-9",

"performers": "UDPM-46",

"duration": 10.0,

"tools": [

{

"name": "Enter Requisition Details",

"uuid": "UDPM-837",

"input_type": "UDPM-167",

"output_type": "UDPM-167",

"flags": 1,

"options": {

"udfs": [

"UDPM-2082",

"UDPM-2142",

"UDPM-415",

"UDPM-1672"

]

}

}

],

"next_states": [

{

"state_name": "Ready for Shipment",

"state_uuid": "UDPM-821",

"btn_text": "POTS not Required",

"btn_css": "#456",

"after_code": "self.next_state_performer = find_user('[redacted]')",

"btn_scale": "large",

"conditions": [

{

"name": "Requisition&rarr;Requisition Document (Upload)",

"condition": "Not Empty",

"value": "",

"message": "The requisition form must be uploaded in order to proceed",

"subject_type": "UDPM-167",

"udf": "UDPM-2082"

}

]

},

{

"state_name": "POTS",

"state_uuid": "UDPM-652",

"btn_text": "Copy of Req to POTS processing",

"btn_css": "#456",

"after_code": "# Script to run After Transition is executed\nself.next_state_performer = find_user('[redacted]')",

"before_code": "# Script to run Before Transition is executed\nparams[:ask_next_performer] = true",

"btn_scale": "large",

"conditions": [

{

"name": "Requisition&rarr;Requisition Document (Upload)",

"condition": "Not Empty",

"value": "",

"message": "The requisition form must be uploaded in order to proceed",

"subject_type": "UDPM-167",

"udf": "UDPM-2082"

}

]

}

],

"em_performer_new_job": true,

"show_options": {

"top": 45,

"left": 44

}

},

{

"name": "Results Received",

"uuid": "UDPM-717",

"owner": "UDPM-67",

"managers": "UDPM-49",

"performers": "UDPM-2",

"duration": 180.0,

"tools": [

{

"name": "Upload PII Files",

"uuid": "UDPM-1541",

"input_type": "UDPM-167",

"output_type": "UDPM-172",

"flags": 0,

"options": {

"obj_type": "SubjectType"

}

},

{

"name": "Notify Attending",

"uuid": "UDPM-1025",

"input_type": "UDPM-167",

"output_type": "UDPM-167",

"description": "Use This Button",

"flags": 5,

"options": {

"obj_type": "EmailTemplate",

"user_groups_ids": [

"UDPM-31"

],

"obj_name": "UDPM-6"

}

}

],

"show_options": {

"top": 499,

"left": 510

},

"end_task": true

},

{

"name": "Return Requisition",

"uuid": "UDPM-927",

"owner": "UDPM-67",

"managers": "UDPM-9",

"performers": "UDPM-49",

"next_states": [

{

"state_name": "Cancel Requisition",

"state_uuid": "UDPM-1077",

"btn_text": "Cancel Requisition",

"btn_css": "rgb(13, 73, 123)",

"btn_scale": "large"

},

{

"state_name": "Prepare Samples",

"state_uuid": "UDPM-869",

"btn_text": "Return to Sample Preparation",

"btn_css": "#456",

"after_code": "self.next_state_performer = find_user('[redacted]')",

"btn_scale": "large"

}

],

"show_options": {

"top": 288,

"left": 933

}

}

],

"entry_point": "UDPM-651",

"show_udfs": "UDPM-415,UDPM-2082,UDPM-2142,UDPM-1696"

},{

"name": "Diploid Alignment Workflow",

"uuid": "UDPM-169",

"subject_type": "UDPM-209",

"state_defs": [

{

"name": "Alignment Cancelled",

"uuid": "UDPM-938",

"owner": "UDPM-67",

"managers": "UDPM-27",

"performers": "UDPM-3",

"show_options": {

"top": 389,

"left": 337.95001220703125

},

"end_task": true

},

{

"name": "Alignment Complete",

"uuid": "UDPM-829",

"owner": "UDPM-67",

"managers": "UDPM-27",

"performers": "UDPM-3",

"tools": [

{

"name": "Alignment Completion Information",

"uuid": "UDPM-1039",

"input_type": "UDPM-209",

"output_type": "UDPM-209",

"description": "VCF, BAM, etc",

"flags": 1,

"options": {

"udfs": [

"UDPM-1901",

"UDPM-1903",

"UDPM-2237",

"UDPM-168",

"UDPM-1859"

]

}

}

],

"next_states": [

{

"state_name": "Variant Calling",

"state_uuid": "UDPM-898",

"btn_text": "Call Variants",

"btn_css": "#456"

}

],

"show_options": {

"top": 137,

"left": 570.949951171875

}

},

{

"name": "Annotation",

"uuid": "UDPM-955",

"owner": "UDPM-67",

"managers": "UDPM-27",

"performers": "UDPM-3",

"next_states": [

{

"state_name": "Shipped to NIH UDP",

"state_uuid": "UDPM-937",

"btn_text": "Ship to NIH",

"btn_css": "#456"

}

],

"show_options": {

"top": 320,

"left": 916.949951171875

}

},

{

"name": "Beagle Run",

"uuid": "UDPM-895",

"owner": "UDPM-67",

"managers": "UDPM-27",

"performers": "UDPM-3",

"next_states": [

{

"state_name": "Iteration 1",

"state_uuid": "UDPM-896",

"btn_text": "Start Iteration 1",

"btn_css": "#456"

}

],

"show_options": {

"top": 5,

"left": 247.95001220703125

}

},

{

"name": "Iteration 1",

"uuid": "UDPM-896",

"owner": "UDPM-67",

"managers": "UDPM-27",

"performers": "UDPM-3",

"next_states": [

{

"state_name": "Iteration 2",

"state_uuid": "UDPM-944",

"btn_text": "Start Iteration 2",

"btn_css": "#456"

},

{

"state_name": "Alignment Complete",

"state_uuid": "UDPM-829",

"btn_text": "Finish Alignment",

"btn_css": "#456"

}

],

"show_options": {

"top": 26,

"left": 485.95001220703125

}

},

{

"name": "Iteration 2",

"uuid": "UDPM-944",

"owner": "UDPM-67",

"managers": "UDPM-27",

"performers": "UDPM-3",

"next_states": [

{

"state_name": "Alignment Complete",

"state_uuid": "UDPM-829",

"btn_text": "Finish Alignment",

"btn_css": "#456"

}

],

"show_options": {

"top": 21,

"left": 705.9000244140625

}

},

{

"name": "Not able to align",

"uuid": "UDPM-926",

"owner": "UDPM-67",

"managers": "UDPM-27",

"performers": "UDPM-60",

"tools": [

{

"name": "Failure Information",

"uuid": "UDPM-1178",

"input_type": "UDPM-209",

"output_type": "UDPM-209",

"flags": 1,

"options": {

"udfs": [

"UDPM-1903",

"UDPM-2460"

]

}

}

],

"next_states": [

{

"state_name": "Alignment Cancelled",

"state_uuid": "UDPM-938",

"btn_text": "Alignment Cancelled",

"btn_css": "#456"

},

{

"state_name": "Start Alignment",

"state_uuid": "UDPM-828",

"btn_text": "Start Alignment Again",

"btn_css": "#456"

}

],

"em_performer_new_job": true,

"show_options": {

"top": 208,

"left": 339.95001220703125

}

},

{

"name": "Received at NIH UDP",

"uuid": "UDPM-941",

"owner": "UDPM-67",

"managers": "UDPM-27",

"performers": "UDPM-3",

"show_options": {

"top": 468,

"left": 522.949951171875

},

"end_task": true

},

{

"name": "Shipped to Appistry",

"uuid": "UDPM-936",

"owner": "UDPM-67",

"managers": "UDPM-27",

"performers": "UDPM-3",

"tools": [

{

"name": "Shipment information",

"uuid": "UDPM-1193",

"input_type": "UDPM-209",

"output_type": "UDPM-209",

"flags": 1,

"options": {

"udfs": [

"UDPM-2236",

"UDPM-353",

"UDPM-1960",

"UDPM-2477",

"UDPM-1094",

"UDPM-1859"

]

}

}

],

"next_states": [

{

"state_name": "Start Alignment",

"state_uuid": "UDPM-828",

"btn_text": "Start Alignment",

"btn_css": "#456"

}

],

"show_options": {

"top": 384,

"left": 35.95001220703125

}

},

{

"name": "Shipped to NIH UDP",

"uuid": "UDPM-937",

"owner": "UDPM-67",

"managers": "UDPM-27",

"performers": "UDPM-3",

"next_states": [

{

"state_name": "Received at NIH UDP",

"state_uuid": "UDPM-941",

"btn_text": "Received at NIH UDP",

"btn_css": "#456"

}

],

"show_options": {

"top": 333,

"left": 650

}

},

{

"name": "Start Alignment",

"uuid": "UDPM-828",

"owner": "UDPM-67",

"managers": "UDPM-27",

"performers": "UDPM-3",

"tools": [

{

"name": "Alignment Information",

"uuid": "UDPM-1040",

"input_type": "UDPM-209",

"output_type": "UDPM-209",

"flags": 1,

"options": {

"udfs": [

"UDPM-2236",

"UDPM-353",

"UDPM-1960",

"UDPM-2477",

"UDPM-2238",

"UDPM-1094",

"UDPM-1859"

]

}

}

],

"next_states": [

{

"state_name": "Beagle Run",

"state_uuid": "UDPM-895",

"btn_text": "Start Beagle",

"btn_css": "#456"

},

{

"state_name": "Not able to align",

"state_uuid": "UDPM-926",

"btn_text": "Not able to align",

"btn_css": "#456"

}

],

"show_options": {

"top": 187,

"left": 101.95001220703125

}

},

{

"name": "Variant Calling",

"uuid": "UDPM-898",

"owner": "UDPM-67",

"managers": "UDPM-27",

"performers": "UDPM-3",

"next_states": [

{

"state_name": "Annotation",

"state_uuid": "UDPM-955",

"btn_text": "Annotate Variants",

"btn_css": "#456"

}

],

"show_options": {

"top": 203,

"left": 803.949951171875

}

}

],

"entry_point": "UDPM-936"

},{

"name": "Alignment Information Workflow",

"uuid": "UDPM-172",

"subject_type": "UDPM-208",

"state_defs": [

{

"name": "Alignment Information",

"uuid": "UDPM-838",

"owner": "UDPM-67",

"managers": "UDPM-27",

"performers": "UDPM-3",

"tools": [

{

"name": "Alignment Information",

"uuid": "UDPM-1062",

"input_type": "UDPM-208",

"output_type": "UDPM-208",

"flags": 1,

"options": {

"udfs": [

"UDPM-1976",

"UDPM-1958",

"UDPM-1935",

"UDPM-1974",

"UDPM-883",

"UDPM-2307",

"UDPM-2308",

"UDPM-2210",

"UDPM-2371",

"UDPM-2480",

"UDPM-2471",

"UDPM-5"

]

}

},

{

"name": "Create Diploid Alignment Record",

"uuid": "UDPM-1411",

"input_type": "UDPM-208",

"output_type": "UDPM-209",

"flags": 0,

"options": {

"obj_type": "SubjectType"

}

}

],

"show_options": {

"top": 119,

"left": 250

},

"end_task": true

}

],

"entry_point": "UDPM-838",

"show_udfs": "UDPM-6,UDPM-1976,UDPM-1974,UDPM-1935,UDPM-883"

},{

"name": "Genome Sequencing Workflow",

"uuid": "UDPM-173",

"subject_type": "UDPM-212",

"state_defs": [

{

"name": "Cancelled",

"uuid": "UDPM-842",

"owner": "UDPM-67",

"managers": "UDPM-22",

"performers": "UDPM-25",

"show_options": {

"top": 40,

"left": 694

},

"end_task": true

},

{

"name": "DNA Received at Sequencing Center",

"uuid": "UDPM-846",

"owner": "UDPM-67",

"managers": "UDPM-27",

"performers": "UDPM-3",

"duration": 156.0,

"tools": [

{

"name": "Additional Information",

"uuid": "UDPM-1064",

"input_type": "UDPM-212",

"output_type": "UDPM-212",

"description": "Axeq QC",

"flags": 1,

"options": {

"udfs": [

"UDPM-2065",

"UDPM-2070",

"UDPM-2071"

]

}

}

],

"next_states": [

{

"state_name": "Lane Files Received",

"state_uuid": "UDPM-847",

"btn_text": "Lane Files Received",

"btn_css": "#456"

}

],

"show_options": {

"top": 137,

"left": 1189

}

},

{

"name": "DNA Sent for Genome",

"uuid": "UDPM-845",

"owner": "UDPM-67",

"managers": "UDPM-22",

"performers": "UDPM-22",

"duration": 14.0,

"next_states": [

{

"state_name": "DNA Received at Sequencing Center",

"state_uuid": "UDPM-846",

"btn_text": "Samples Received at Sequencing Center",

"btn_css": "#456"

}

],

"show_options": {

"top": 65,

"left": 837

}

},

{

"name": "Genome Wait List",

"uuid": "UDPM-839",

"owner": "UDPM-67",

"managers": "UDPM-9",

"performers": "UDPM-44",

"tools": [

{

"name": "Additional Information",

"uuid": "UDPM-1067",

"input_type": "UDPM-212",

"output_type": "UDPM-212",

"flags": 1,

"options": {

"udfs": [

"UDPM-6",

"UDPM-2039"

]

}

}

],

"next_states": [

{

"state_name": "Cancelled",

"state_uuid": "UDPM-842",

"btn_text": "Cancel",

"btn_css": "#456"

},

{

"state_name": "Hold for Decision",

"state_uuid": "UDPM-841",

"btn_text": "Place on Hold",

"btn_css": "#456"

},

{

"state_name": "Preparation List",

"state_uuid": "UDPM-840",

"btn_text": "Add to Preparation List",

"btn_css": "#456"

}

],

"show_options": {

"top": 52,

"left": 74

}

},

{

"name": "Hold for Decision",

"uuid": "UDPM-841",

"owner": "UDPM-67",

"managers": "UDPM-9",

"performers": "UDPM-2",

"duration": 30.0,

"next_states": [

{

"state_name": "Genome Wait List",

"state_uuid": "UDPM-839",

"btn_text": "Return to Wait List",

"btn_css": "#456"

},

{

"state_name": "Cancelled",

"state_uuid": "UDPM-842",

"btn_text": "Cancel",

"btn_css": "#456"

}

],

"show_options": {

"top": 199,

"left": 290

}

},

{

"name": "Lane Files Received",

"uuid": "UDPM-847",

"owner": "UDPM-67",

"managers": "UDPM-27",

"performers": "UDPM-3",

"tools": [

{

"name": "Lane File Information",

"uuid": "UDPM-1063",

"input_type": "UDPM-212",

"output_type": "UDPM-212",

"flags": 1,

"options": {

"udfs": [

"UDPM-877",

"UDPM-371",

"UDPM-1958"

]

}

}

],

"show_options": {

"top": 387,

"left": 1340

},

"end_task": true

},

{

"name": "Make DNA Aliquot",

"uuid": "UDPM-843",

"owner": "UDPM-67",

"managers": "UDPM-22",

"performers": "UDPM-25",

"tools": [

{

"name": "DNA Information",

"uuid": "UDPM-1066",

"input_type": "UDPM-212",

"output_type": "UDPM-212",

"description": "Preparation",

"flags": 1,

"options": {

"udfs": [

"UDPM-875",

"UDPM-1342",

"UDPM-1378",

"UDPM-2040",

"UDPM-2041",

"UDPM-2042",

"UDPM-1344",

"UDPM-1384"

]

}

}

],

"next_states": [

{

"state_name": "Cancelled",

"state_uuid": "UDPM-842",

"btn_text": "Cancel",

"btn_css": "#456"

},

{

"state_name": "Prepare DNA for Shipment",

"state_uuid": "UDPM-844",

"btn_text": "DNA Prepared",

"btn_css": "#456"

}

],

"show_options": {

"top": 535,

"left": 543

}

},

{

"name": "Preparation List",

"uuid": "UDPM-840",

"owner": "UDPM-67",

"managers": "UDPM-9",

"performers": "UDPM-44",

"next_states": [

{

"state_name": "Make DNA Aliquot",

"state_uuid": "UDPM-843",

"btn_text": "Prepare DNA for Shipment",

"btn_css": "#456"

},

{

"state_name": "Cancelled",

"state_uuid": "UDPM-842",

"btn_text": "Cancel",

"btn_css": "#456"

}

],

"show_options": {

"top": 441,

"left": 214

}

},

{

"name": "Prepare DNA for Shipment",

"uuid": "UDPM-844",

"owner": "UDPM-67",

"managers": "UDPM-22",

"performers": "UDPM-25",

"tools": [

{

"name": "Shipping Information",

"uuid": "UDPM-1065",

"input_type": "UDPM-212",

"output_type": "UDPM-212",

"description": "DNA for Genome",

"flags": 1,

"options": {

"udfs": [

"UDPM-2039",

"UDPM-2281",

"UDPM-309"

]

}

}

],

"next_states": [

{

"state_name": "DNA Sent for Genome",

"state_uuid": "UDPM-845",

"btn_text": "DNA Sent",

"btn_css": "#456"

}

],

"show_options": {

"top": 547,

"left": 878

}

}

],

"entry_point": "UDPM-839"

},{

"name": "PhenoTips Review",

"uuid": "UDPM-168",

"subject_type": "UDPM-205",

"state_defs": [

{

"name": "Final Review by Attending Physician",

"uuid": "UDPM-826",

"owner": "UDPM-67",

"managers": "UDPM-49",

"performers": "UDPM-2",

"next_states": [

{

"state_name": "Final Review by Curators",

"state_uuid": "UDPM-1102",

"btn_text": "Ready for Final Curation",

"btn_css": "rgb(13, 73, 123)",

"before_code": "recipients = []\nrecipients << find_user_group('Phenotips Curators')\n\natt = subj.get_value('PhenoTips Attending Sign Off')\npc = subj.get_value('PhenoTips Primary Clinician Sign Off')\n\nif not att.present? and not pc.present?\n raise (\"Both the Attending the Primary Clinican must Sign-off on PhenoTips Record.\") \nelse \n send_email(recipients, find_email_template(\"Ready for Final Round of Curation\"), subj)\nend ",

"btn_scale": "large"

}

],

"em_performer_new_job": true,

"show_options": {

"top": 279,

"left": 121

}

},

{

"name": "Final Review by Curators",

"uuid": "UDPM-1102",

"owner": "UDPM-67",

"managers": "UDPM-49",

"performers": "UDPM-63",

"tools": [

{

"name": "Curation Notes",

"uuid": "UDPM-1506",

"input_type": "UDPM-205",

"output_type": "UDPM-205",

"flags": 1,

"options": {

"udfs": [

"UDPM-5"

]

}

}

],

"next_states": [

{

"state_name": "Record Complete",

"state_uuid": "UDPM-849",

"btn_text": "PhenoTips Record Complete",

"btn_css": "rgb(13, 73, 123)",

"after_code": "subj.set_value('Final Round of Curation Completed by', params['Final Round of Curation Completed by'])\n\nsubj.set_value('PhenoTips Curation Completed', params['PhenoTips Curation Completed'])\n",

"before_code": "subj.set_value('Final Round of Curation Completed by', User.curr_user)\n\nparams[:defaults] = {\n 'Final Round of Curation Completed by'=> User.curr_user,\n 'PhenoTips Curation Completed'=> Time.now\n}",

"btn_scale": "large",

"after_code_params": [

"UDPM-2900",

"UDPM-2899"

]

}

],

"show_options": {

"top": 423,

"left": 154

}

},

{

"name": "First Review by Curators",

"uuid": "UDPM-848",

"owner": "UDPM-67",

"managers": "UDPM-49",

"performers": "UDPM-63",

"tools": [

{

"name": "Curation Notes",

"uuid": "UDPM-1069",

"input_type": "UDPM-205",

"output_type": "UDPM-205",

"flags": 1,

"before_code": "c = subj.get_value(\"Comments\")\n\nparams[:defaults] = {\n 'Comments'=> c\n}",

"options": {

"udfs": [

"UDPM-5"

]

}

}

],

"next_states": [

{

"state_name": "Final Review by Attending Physician",

"state_uuid": "UDPM-826",

"btn_text": "Ready for Attending Review",

"btn_css": "#456",

"after_code": "subj.set_value('First Round of Curation Completed by', User.curr_user)\nsubj.set_value('First Round of Curation Completed on', params['First Round of Curation Completed on'])\n\np = subj.get_value('Patient')\natt = p.get_value('Attending Physician')\nif att\n self.next_state_performer = att\nelse\n self.next_state_performer = User.find_by_username('[redacted]')\nend",

"before_code": "params[:defaults] = {\n 'First Round of Curation Completed by'=> User.curr_user,\n 'First Round of Curation Completed on'=> Time.now\n}",

"btn_scale": "large",

"btn_tooltip": "Curation is complete",

"after_code_params": [

"UDPM-2916",

"UDPM-2898"

],

"hide_button_if_not_condition": true

}

],

"show_options": {

"top": 142.96875,

"left": 154.984375

}

},

{

"name": "Initial Data Entry by Support Team",

"uuid": "UDPM-1101",

"owner": "UDPM-67",

"managers": "UDPM-49",

"performers": "UDPM-63",

"tools": [

{

"name": "Add Curation Notes",

"uuid": "UDPM-1507",

"input_type": "UDPM-205",

"output_type": "UDPM-205",

"flags": 1,

"options": {

"udfs": [

"UDPM-5"

]

}

}

],

"next_states": [

{

"state_name": "Review by Clinical Team",

"state_uuid": "UDPM-825",

"btn_text": "Ready for Review by Clinical Team",

"btn_css": "rgb(13, 73, 123)",

"after_code": "subj.set_value('Initial Curation Completed by', User.curr_user)\nsubj.set_value('Initial Round of Curation Completed on', params['Initial Round of Curation Completed on'])\n\np = subj.get_value('Patient')\natt = p.get_value('Attending Physician')\nif att\n self.next_state_performer = att\nelse\n self.next_state_performer = User.find_by_username('[redacted]')\nend",

"before_code": "params[:defaults] = {\n 'Initial Round of Curation Completed on'=> User.curr_user,\n 'Initial Round of Curation Completed on'=> Time.now\n}",

"btn_scale": "large",

"after_code_params": [

"UDPM-2917"

]

}

],

"show_options": {

"top": 37,

"left": 574

}

},

{

"name": "Record Complete",

"uuid": "UDPM-849",

"owner": "UDPM-67",

"managers": "UDPM-9",

"performers": "UDPM-49",

"show_options": {

"top": 576,

"left": 182

},

"end_task": true

},

{

"name": "Review by Clinical Team",

"uuid": "UDPM-825",

"owner": "UDPM-67",

"managers": "UDPM-49",

"performers": "UDPM-2",

"tools": [

{

"name": "Ready for Curation",

"uuid": "UDPM-1505",

"input_type": "UDPM-205",

"output_type": "UDPM-205",

"flags": 6,

"before_code": "recipients = []\nrecipients << find_user_group('Phenotips Curators')\n\npat = subj.get_value('Patient')\n\natt1 = Property.find_by_display_name('PhenoTips Attending Sign Off')\natt = pat.get_value(att1)\npc1 = Property.find_by_display_name('PhenoTips Primary Clinician Sign Off')\npc = pat.get_value(pc1)\n\n#att = subj.get_value('Phenotips Attending Sign Off')\n#raise att.to_s\n#initial = subj.get_value('Phenotips Primary Clinician Sign Off')\n\nif att.present? and pc.present?\n advance_workflow(\"PhenoTips Review\",\"Final Review by Curators\",subj)\n send_email(recipients, find_email_template(\"Ready for Final Round of Curation\"), subj)\nelsif att.present? ^ pc.present?\n advance_workflow(\"PhenoTips Review\",\"First Review by Curators\",subj)\n send_email(recipients, find_email_template(\"Ready for First Round of Curation\"), subj)\nelse\n raise (\"Please Sign-off on PhenoTips Record.\") \nend ",

"no_conformation": true

}

],

"next_states": [

{

"state_name": "Initial Data Entry by Support Team",

"state_uuid": "UDPM-1101",

"btn_text": "Send to Support Team for Initial Data Entry",

"btn_css": "rgb(13, 73, 123)",

"after_code": "recipients = []\nrecipients << find_user_group('Phenotips Curators')\nsend_email(recipients, find_email_template(\"Needs Initial Curation\"), subj)",

"btn_scale": "large"

}

],

"show_options": {

"top": 33,

"left": 155

}

}

],

"entry_point": "UDPM-825",

"ask_performer": true,

"show_udfs": "UDPM-6"

},{

"name": "Buffy Coat Isolation Workflow",

"uuid": "UDPM-184",

"subject_type": "UDPM-219",

"state_defs": [

{

"name": "Cancel",

"uuid": "UDPM-1000",

"owner": "UDPM-67",

"managers": "UDPM-22",

"performers": "UDPM-69",

"show_options": {

"top": 213,

"left": 100

},

"end_task": true

},

{

"name": "Isolation",

"uuid": "UDPM-892",

"owner": "UDPM-67",

"managers": "UDPM-22",

"performers": "UDPM-69",

"next_states": [

{

"state_name": "Cancel",

"state_uuid": "UDPM-1000",

"btn_text": "Cancel",

"btn_css": "#456"

},

{

"state_name": "Store",

"state_uuid": "UDPM-893",

"btn_text": "Store",

"btn_css": "#456",

"after_code": "require_script 'lims_helper'\n\nvial_count = params['Number of Vials to Store']\nself.next_state_performer = User.curr_user\nsubmit_to_fpro(subj, 'Buffy Coat Pellet', '7000000616', vial_count)",

"after_code_params": [

"UDPM-1450"

]

}

],

"show_options": {

"top": 38,

"left": 59

}

},

{

"name": "Store",

"uuid": "UDPM-893",

"owner": "UDPM-67",

"managers": "UDPM-22",

"performers": "UDPM-69",

"show_options": {

"top": 163,

"left": 304

},

"end_task": true

}

],

"entry_point": "UDPM-892",

"show_udfs": "UDPM-1332"

},{

"name": "Platelet Isolation Workflow",

"uuid": "UDPM-185",

"subject_type": "UDPM-220",

"state_defs": [

{

"name": "Platelet Isolation",

"uuid": "UDPM-890",

"owner": "UDPM-67",

"managers": "UDPM-22",

"performers": "UDPM-24",

"next_states": [

{

"state_name": "Stored",

"state_uuid": "UDPM-891",

"btn_text": "Store",

"btn_css": "#456",

"after_code": "require_script 'lims_helper'\n\nvial_count = params['Number of Vials to Store']\nself.next_state_performer = User.curr_user\nsubmit_to_fpro(subj, 'Platelet Pellet', vial_count)",

"after_code_params": [

"UDPM-1450"

]

}

],

"show_options": {

"top": 121,

"left": 201

}

},

{

"name": "Stored",

"uuid": "UDPM-891",

"owner": "UDPM-67",

"managers": "UDPM-22",

"performers": "UDPM-24",

"show_options": {

"top": 267,

"left": 411

},

"end_task": true

}

],

"entry_point": "UDPM-890"

},{

"name": "Glycome Workflow",

"uuid": "UDPM-188",

"subject_type": "UDPM-224",

"state_defs": [

{

"name": "Additional Glycome Tests",

"uuid": "UDPM-924",

"owner": "UDPM-67",

"managers": "UDPM-38",

"performers": "UDPM-38",

"tools": [

{

"name": "Perform Assay",

"uuid": "UDPM-1182",

"input_type": "UDPM-224",

"output_type": "UDPM-225",

"flags": 0,

"options": {

"obj_type": "SubjectType"

}

},

{

"name": "Initiate Collaboration",

"uuid": "UDPM-1183",

"input_type": "UDPM-224",

"output_type": "UDPM-147",

"flags": 0,

"options": {

"obj_type": "SubjectType"

}

},

{

"name": "Plasmid Construction",

"uuid": "UDPM-1184",

"input_type": "UDPM-224",

"output_type": "UDPM-193",

"flags": 0,

"options": {

"obj_type": "SubjectType"

}

},

{

"name": "Add Glycome Short List",

"uuid": "UDPM-1186",

"input_type": "UDPM-224",

"output_type": "UDPM-60",

"description": "SOP Exome Anlaysis",

"flags": 0,

"options": {

"obj_type": "SubjectType"

}

},

{

"name": "Send for Sanger Sequencing",

"uuid": "UDPM-1188",

"input_type": "UDPM-224",

"output_type": "UDPM-221",

"flags": 0,

"options": {

"obj_type": "SubjectType"

}

},

{

"name": "Culture Fibrobasts",

"uuid": "UDPM-1204",

"input_type": "UDPM-224",

"output_type": "UDPM-40",

"flags": 0,

"options": {

"obj_type": "SubjectType"

}

},

{

"name": "Add Notes",

"uuid": "UDPM-1226",

"input_type": "UDPM-224",

"output_type": "UDPM-224",

"flags": 1,

"options": {

"udfs": [

"UDPM-2632",

"UDPM-2532",

"UDPM-2533",

"UDPM-2534",

"UDPM-2535",

"UDPM-2536",

"UDPM-2746",

"UDPM-2747",

"UDPM-2855",

"UDPM-2462"

]

}

},

{

"name": "Rescue Cell Line",

"uuid": "UDPM-1269",

"input_type": "UDPM-224",

"output_type": "UDPM-247",

"description": "Fibroblast and HELA",

"flags": 0,

"options": {

"obj_type": "SubjectType"

}

}

],

"next_states": [

{

"state_name": "Glycome Analysis Complete",

"state_uuid": "UDPM-922",

"btn_text": "Glycome Analysis Complete",

"btn_css": "#456",

"after_code": "subj.set_value('Final Glycome Results Summary', params['Final Glycome Results Summary'])",

"after_code_params": [

"UDPM-2466"

]

},

{

"state_name": "Cancel",

"state_uuid": "UDPM-934",

"btn_text": "Cancel Glycome Pipeline",

"btn_css": "#456",

"after_code": "subj.set_value('Rationale for Cancellation',params['Rationale/Reasoning'])",

"after_code_params": [

"UDPM-883"

]

}

],

"show_options": {

"top": 351,

"left": 275

}

},

{

"name": "Cancel",

"uuid": "UDPM-934",

"owner": "UDPM-67",

"managers": "UDPM-38",

"performers": "UDPM-38",

"show_options": {

"top": 114,

"left": 61

},

"end_task": true

},

{

"name": "Glycome Analysis Complete",

"uuid": "UDPM-922",

"owner": "UDPM-67",

"managers": "UDPM-38",

"performers": "UDPM-38",

"show_options": {

"top": 362,

"left": 712

},

"end_task": true

},

{

"name": "Glycome Testing",

"uuid": "UDPM-920",

"owner": "UDPM-67",

"managers": "UDPM-38",

"performers": "UDPM-38",

"tools": [

{

"name": "Add Glycome Short List",

"uuid": "UDPM-1166",

"input_type": "UDPM-224",

"output_type": "UDPM-60",

"description": "SOP Exome Analysis",

"flags": 0,

"hide_button_if_not_condition": true,

"options": {

"obj_type": "SubjectType"

}

},

{

"name": "Culture Fibrobasts",

"uuid": "UDPM-1167",

"input_type": "UDPM-224",

"output_type": "UDPM-40",

"flags": 0,

"options": {

"obj_type": "SubjectType"

}

},

{

"name": "Send for Sanger Sequencing",

"uuid": "UDPM-1169",

"input_type": "UDPM-224",

"output_type": "UDPM-221",

"flags": 0,

"options": {

"obj_type": "SubjectType"

}

},

{

"name": "Add Notes",

"uuid": "UDPM-1225",

"input_type": "UDPM-224",

"output_type": "UDPM-224",

"flags": 1,

"options": {

"udfs": [

"UDPM-2632",

"UDPM-2532",

"UDPM-2533",

"UDPM-2534",

"UDPM-2535",

"UDPM-2536",

"UDPM-2746",

"UDPM-2747",

"UDPM-2855",

"UDPM-2462"

]

}

}

],

"next_states": [

{

"state_name": "Glycome Analysis Complete",

"state_uuid": "UDPM-922",

"btn_text": "Glycome Analysis Complete",

"btn_css": "#456",

"after_code": "subj.set_value('Final Glycome Results Summary', params['Final Glycome Results Summary'])",

"after_code_params": [

"UDPM-2466"

]

},

{

"state_name": "Additional Glycome Tests",

"state_uuid": "UDPM-924",

"btn_text": "Additional Glycome Analyses",

"btn_css": "#456",

"after_code": "self.next_state_performer = User.curr_user",

"no_conformation": true

},

{

"state_name": "Cancel",

"state_uuid": "UDPM-934",

"btn_text": "Cancel Glycome Pipeline",

"btn_css": "#456",

"after_code": "subj.set_value('Rationale for Cancellation',params['Rationale/Reasoning'])",

"after_code_params": [

"UDPM-883"

]

}

],

"show_options": {

"top": 123,

"left": 695

}

},

{

"name": "Patient for Glycome Analysis",

"uuid": "UDPM-929",

"owner": "UDPM-67",

"managers": "UDPM-38",

"performers": "UDPM-38",

"duration": 365.0,

"tools": [

{

"name": "Evaluate Glycome Results",

"uuid": "UDPM-1256",

"input_type": "UDPM-224",

"output_type": "UDPM-224",

"flags": 1,

"options": {

"udfs": [

"UDPM-2532",

"UDPM-2533",

"UDPM-2534",

"UDPM-2535",

"UDPM-2536",

"UDPM-2746",

"UDPM-2747",

"UDPM-2855",

"UDPM-2462"

]

}

}

],

"next_states": [

{

"state_name": "Additional Glycome Tests",

"state_uuid": "UDPM-924",

"btn_text": "Normal Glycome Results",

"btn_css": "#456",

"after_code": "subj.set_value('Glycome Results Flag', 'Normal')"

},

{

"state_name": "Glycome Testing",

"state_uuid": "UDPM-920",

"btn_text": "Abnormal Glycome Results",

"btn_css": "#456",

"after_code": "subj.set_value('Glycome Results Flag', 'Abnormal')\n\nself.next_state_performer = User.curr_user\nrecipients = []\nrecipients << find_user('[redacted]')\nsend_email(recipients, find_email_template('Abnormal Glycome Alert'), subj)"

},

{

"state_name": "Cancel",

"state_uuid": "UDPM-934",

"btn_text": "Cancel Glycome Pipeline",

"btn_css": "#456",

"after_code": "subj.set_value('Rationale for Cancellation',params['Rationale/Reasoning'])",

"after_code_params": [

"UDPM-883"

]

}

],

"show_options": {

"top": 10,

"left": 320

}

}

],

"entry_point": "UDPM-929",

"ask_performer": true

},{

"name": "Assay Workflow",

"uuid": "UDPM-189",

"subject_type": "UDPM-225",

"state_defs": [

{

"name": "Assay Complete",

"uuid": "UDPM-932",

"owner": "UDPM-67",

"managers": "UDPM-22",

"performers": "UDPM-21",

"show_options": {

"top": 228,

"left": 277

},

"end_task": true

},

{

"name": "Perform Assay",

"uuid": "UDPM-931",

"owner": "UDPM-67",

"managers": "UDPM-22",

"performers": "UDPM-21",

"duration": 50.0,

"tools": [

{

"name": "Perform Assay",

"uuid": "UDPM-1181",

"input_type": "UDPM-225",

"output_type": "UDPM-225",

"description": "and Upload Results",

"flags": 1,

"options": {

"udfs": [

"UDPM-2457",

"UDPM-67",

"UDPM-2459",

"UDPM-2458",

"UDPM-2473",

"UDPM-2474",

"UDPM-2475",

"UDPM-2472",

"UDPM-2374",

"UDPM-1649"

]

}

}

],

"next_states": [

{

"state_name": "Assay Complete",

"state_uuid": "UDPM-932",

"btn_text": "Assay Complete",

"btn_css": "#456",

"after_code": "raise \"You must summarize the data before continuing\" unless subj.get_value('Summary of Results').present?\nself.next_state_performer = User.curr_user",

"conditions": [

{

"name": "Assay&rarr;Assay Type",

"condition": "Not Empty",

"value": "",

"message": "No Assay Type Recorded",

"subject_type": "UDPM-225",

"udf": "UDPM-2457"

},

{

"name": "Assay&rarr;Date Completed",

"condition": "Not Empty",

"value": "",

"message": "Record \"Date Completed\" and upload results files for assay.",

"subject_type": "UDPM-225",

"udf": "UDPM-2459"

}

]

}

],

"show_options": {

"top": 78,

"left": 99

}

}

],

"entry_point": "UDPM-931",

"ask_performer": true,

"show_udfs": "UDPM-67,UDPM-2374"

},{

"name": "Erythrocyte Pellet Workflow",

"uuid": "UDPM-190",

"subject_type": "UDPM-227",

"state_defs": [

{

"name": "Extraction",

"uuid": "UDPM-939",

"owner": "UDPM-67",

"managers": "UDPM-22",

"performers": "UDPM-69",

"duration": 1.0,

"next_states": [

{

"state_name": "Stored",

"state_uuid": "UDPM-940",

"btn_text": "Store",

"btn_css": "#456",

"after_code": "require_script 'lims_helper'\n\nvial_count = params['Number of Vials to Store']\nself.next_state_performer = User.curr_user\nsubmit_to_fpro(subj, 'Erythrocyte Pellet', '7000000617', vial_count)",

"after_code_params": [

"UDPM-1450"

]

}

],

"show_options": {

"top": 70,

"left": 161

}

},

{

"name": "Stored",

"uuid": "UDPM-940",

"owner": "UDPM-67",

"managers": "UDPM-22",

"performers": "UDPM-69",

"show_options": {

"top": 248,

"left": 355

},

"end_task": true

}

],

"entry_point": "UDPM-939",

"show_udfs": "UDPM-1332"

},{

"name": "Rescue Cell Culture Workflow",

"uuid": "UDPM-201",

"subject_type": "UDPM-247",

"state_defs": [

{

"name": "Cell Culture Started",

"uuid": "UDPM-1003",

"owner": "UDPM-67",

"managers": "UDPM-22",

"performers": "UDPM-26",

"tools": [

{

"name": "Send for Collaboration",

"uuid": "UDPM-1260",

"input_type": "UDPM-247",

"output_type": "UDPM-147",

"flags": 0,

"after_code": "next_state = params[\"culture_workflow_states\"]\nadvance_workflow(\"Rescue Cell Culture Workflow\",'Shipped for Testing',subj)",

"options": {

"obj_type": "SubjectType"

}

},

{

"name": "Transduction Information",

"uuid": "UDPM-1261",

"input_type": "UDPM-247",

"output_type": "UDPM-247",

"flags": 1,

"options": {

"udfs": [

"UDPM-1833",

"UDPM-1834",

"UDPM-1999",

"UDPM-2503"

]

}

},

{

"name": "Retested Mycoplasma Results",

"uuid": "UDPM-1262",

"input_type": "UDPM-247",

"output_type": "UDPM-247",

"flags": 1,

"hide_button_if_not_condition": true,

"conditions": [

{

"name": "Rescue Cell Culture&rarr;Date Treatment Started",

"condition": "Not Empty",

"value": "",

"message": "",

"subject_type": "UDPM-247",

"udf": "UDPM-457"

},

{

"name": "Rescue Cell Culture&rarr;Date Culture Started",

"condition": "Empty",

"value": "",

"message": "",

"subject_type": "UDPM-247",

"udf": "UDPM-1035"

}

],

"options": {

"udfs": [

"UDPM-1035",

"UDPM-1668",

"UDPM-457",

"UDPM-1841",

"UDPM-1842",

"UDPM-1843"

]

}

},

{

"name": "Passage Cells",

"uuid": "UDPM-1263",

"input_type": "UDPM-247",

"output_type": "UDPM-247",

"flags": 0,

"options": {

"obj_type": "SubjectType"

}

},

{

"name": "Mycoplasma Test Results",

"uuid": "UDPM-1264",

"input_type": "UDPM-247",

"output_type": "UDPM-247",

"flags": 1,

"options": {

"udfs": [

"UDPM-337",

"UDPM-1666",

"UDPM-1667",

"UDPM-455"

]

}

},

{

"name": "Culture Information",

"uuid": "UDPM-1265",

"input_type": "UDPM-247",

"output_type": "UDPM-247",

"flags": 1,

"options": {

"udfs": [

"UDPM-1833",

"UDPM-1035",

"UDPM-415",

"UDPM-2561",

"UDPM-2656",

"UDPM-1665",

"UDPM-2200",

"UDPM-1332",

"UDPM-1670",

"UDPM-2937",

"UDPM-2936"

]

}

},

{

"name": "Extract RNA",

"uuid": "UDPM-1360",

"input_type": "UDPM-247",

"output_type": "UDPM-43",

"flags": 0,

"options": {

"obj_type": "SubjectType"

}

}

],

"next_states": [

{

"state_name": "Shipped for Testing",

"state_uuid": "UDPM-1008",

"btn_text": "Culture Shipped",

"btn_css": "#456"

},

{

"state_name": "Discarded",

"state_uuid": "UDPM-1005",

"btn_text": "Discard",

"btn_css": "#456"

},

{

"state_name": "Culture Passaged",

"state_uuid": "UDPM-1007",

"btn_text": "Culture Used",

"btn_css": "#456"

},

{

"state_name": "Treat Cell Line",

"state_uuid": "UDPM-1006",

"btn_text": "Treat Cell Line",

"btn_css": "#456",

"btn_scale": "large",

"hide_button_if_not_condition": true,

"conditions": [

{

"name": "Rescue Cell Culture&rarr;Mycoplasma Contaminated",

"condition": "Not Empty",

"value": "",

"message": "",

"subject_type": "UDPM-247",

"udf": "UDPM-1667"

}

]

},

{

"state_name": "Stored",

"state_uuid": "UDPM-1004",

"btn_text": "Freeze Cells",

"btn_css": "#456",

"after_code": "require_script 'rescue_helper'\nvial_count = params['Number of Vials to Store']\nif User.curr_user.user_groups.map(&:name).join(',').include? \"Lab Personnel - Twinbrook\"\n if subj.get_value('Mycoplasma Free') == 'true' || subj.get_value('Mycoplasma Free after Treatment') == 'true'\n if subj.get_value('Storage Temp') == 'Cell Line (-150)'\n if subj.get_value('Cell Type') == 'Rescued Fibroblast'\n submit_to_fpro(subj, 'Rescued Fibroblast', '7000000121', vial_count) do |s|\n end\n elsif subj.get_value('Sample Type') == 'Rescued HELA'\n submit_to_fpro(subj, 'Rescued HELA', '7000000122', vial_count) do |s| \n end\n end\n elsif subj.get_value('Storage Temp') == 'Cell Pellet (-80)'\n submit_to_fpro(subj, 'Cell Pellet', '257', vial_count) do |s|\n end\n elsif subj.get_value('Storage Temp') == 'Culture Media (-80)'\n submit_to_fpro(subj, 'Culture media', '257', vial_count) do |s|\n end\n else\n raise 'You must enter a storage temperature to proceed'\n end\n else\n raise \"Your cell line must be free of mycoplasma in order to freeze\"\n end\nelsif User.curr_user.user_groups.map(&:name).join(',').include? \"Lab Personnel - Building 50\"\n if subj.get_value('Mycoplasma Free') == 'true' || subj.get_value('Mycoplasma Free after Treatment') == 'true'\n if subj.get_value('Storage Temp') == 'Cell Line (-150)'\n if subj.get_value('Cell Type') == 'Rescued Fibroblast'\n submit_to_fpro(subj, 'Rescued Fibroblast', '7000000417', vial_count) do |s|\n end\n elsif subj.get_value('Sample Type') == 'Rescued HELA'\n submit_to_fpro(subj, 'Rescued HELA', '7000000417', vial_count) do |s| \n end\n end\n elsif subj.get_value('Storage Temp') == 'Cell Pellet (-80)'\n submit_to_fpro(subj, 'Cell Pellet', '7000000623', vial_count) do |s|\n end\n elsif subj.get_value('Storage Temp') == 'Culture Media (-80)'\n submit_to_fpro(subj, 'Culture media', '7000000623', vial_count) do |s|\n end\n else\n raise 'You must enter a storage temperature to proceed'\n end\n else\n raise \"Your cell line must be free of mycoplasma in order to freeze\"\n end\nelse\n raise(\"This script only allows users who belong to either Twinbrook Lab or Building 50 Lab, please contact the administrators\")\nend",

"btn_scale": "large",

"after_code_params": [

"UDPM-1450"

]

}

],

"show_options": {

"top": 73,

"left": 284

}

},

{

"name": "Culture Passaged",

"uuid": "UDPM-1007",

"owner": "UDPM-67",

"managers": "UDPM-22",

"performers": "UDPM-26",

"show_options": {

"top": 441,

"left": 301

},

"end_task": true

},

{

"name": "Discarded",

"uuid": "UDPM-1005",

"owner": "UDPM-67",

"managers": "UDPM-22",

"performers": "UDPM-26",

"show_options": {

"top": 279,

"left": 638

},

"end_task": true

},

{

"name": "Shipped for Testing",

"uuid": "UDPM-1008",

"owner": "UDPM-67",

"managers": "UDPM-22",

"performers": "UDPM-26",

"show_options": {

"top": 293,

"left": 61

},

"end_task": true

},

{

"name": "Stored",

"uuid": "UDPM-1004",

"owner": "UDPM-67",

"managers": "UDPM-22",

"performers": "UDPM-26",

"show_options": {

"top": 121,

"left": 695

},

"end_task": true

},

{

"name": "Treat Cell Line",

"uuid": "UDPM-1006",

"owner": "UDPM-67",

"managers": "UDPM-22",

"performers": "UDPM-26",

"next_states": [

{

"state_name": "Cell Culture Started",

"state_uuid": "UDPM-1003",

"btn_text": "Retest for Mycoplasma",

"btn_css": "#456"

}

],

"show_options": {

"top": 352,

"left": 454

}

}

],

"entry_point": "UDPM-1003",

"ask_performer": true,

"show_udfs": "UDPM-415,UDPM-1665,UDPM-6,UDPM-2561,UDPM-2656,UDPM-1035"

},{

"name": "Hard Drive Tracking",

"uuid": "UDPM-202",

"subject_type": "UDPM-248",

"state_defs": [

{

"name": "Hard Drive In House",

"uuid": "UDPM-1009",

"owner": "UDPM-67",

"managers": "UDPM-27",

"performers": "UDPM-3",

"tools": [

{

"name": "Information",

"uuid": "UDPM-1266",

"input_type": "UDPM-248",

"output_type": "UDPM-248",

"flags": 1,

"options": {

"udfs": [

"UDPM-1420",

"UDPM-1656",

"UDPM-2658",

"UDPM-2659",

"UDPM-2660",

"UDPM-2374"

]

}

}

],

"next_states": [

{

"state_name": "Hard Drive in Transit",

"state_uuid": "UDPM-1010",

"btn_text": "Ship Hard Drive",

"btn_css": "#456"

},

{

"state_name": "No Longer in Use",

"state_uuid": "UDPM-1012",

"btn_text": "No longer in use",

"btn_css": "#456"

}

],

"show_options": {

"top": 109,

"left": 149

}

},

{

"name": "Hard Drive in Transit",

"uuid": "UDPM-1010",

"owner": "UDPM-67",

"managers": "UDPM-27",

"performers": "UDPM-3",

"next_states": [

{

"state_name": "No Longer in Use",

"state_uuid": "UDPM-1012",

"btn_text": "No longer in use",

"btn_css": "#456"

},

{

"state_name": "Hard Drive In House",

"state_uuid": "UDPM-1009",

"btn_text": "Hard Drive Received In House",

"btn_css": "#456",

"btn_scale": "large"

},

{

"state_name": "Hard Drive out for Collaboration",

"state_uuid": "UDPM-1011",

"btn_text": "Hard Drive Received by Collaborator",

"btn_css": "#456",

"btn_scale": "large"

}

],

"show_options": {

"top": 105,

"left": 475

}

},

{

"name": "Hard Drive out for Collaboration",

"uuid": "UDPM-1011",

"owner": "UDPM-67",

"managers": "UDPM-27",

"performers": "UDPM-3",

"tools": [

{

"name": "Information",

"uuid": "UDPM-1267",

"input_type": "UDPM-248",

"output_type": "UDPM-248",

"flags": 1,

"options": {

"udfs": [

"UDPM-1420",

"UDPM-1656",

"UDPM-2658",

"UDPM-2659",

"UDPM-2660",

"UDPM-2374"

]

}

}

],

"next_states": [

{

"state_name": "Hard Drive in Transit",

"state_uuid": "UDPM-1010",

"btn_text": "Ship Hard Drive",

"btn_css": "#456"

},

{

"state_name": "No Longer in Use",

"state_uuid": "UDPM-1012",

"btn_text": "No longer in use",

"btn_css": "#456"

}

],

"show_options": {

"top": 365,

"left": 566

}

},

{

"name": "No Longer in Use",

"uuid": "UDPM-1012",

"owner": "UDPM-67",

"managers": "UDPM-27",

"performers": "UDPM-3",

"show_options": {

"top": 393,

"left": 179

},

"end_task": true

}

],

"entry_point": "UDPM-1009",

"show_udfs": "UDPM-2658,UDPM-2660,UDPM-1420,UDPM-1656,UDPM-2659,UDPM-2374"

},{

"name": "MiniPrep Storage",

"uuid": "UDPM-203",

"subject_type": "UDPM-249",

"state_defs": [

{

"name": "MiniPrep Information",

"uuid": "UDPM-1015",

"owner": "UDPM-67",

"managers": "UDPM-22",

"performers": "UDPM-21",

"tools": [

{

"name": "Information",

"uuid": "UDPM-1276",

"input_type": "UDPM-249",

"output_type": "UDPM-249",

"flags": 1,

"options": {

"udfs": [

"UDPM-6",

"UDPM-1977",

"UDPM-1332",

"UDPM-1016",

"UDPM-627",

"UDPM-628",

"UDPM-5",

"UDPM-1907"

]

}

}

],

"next_states": [

{

"state_name": "Stored",

"state_uuid": "UDPM-1016",

"btn_text": "Store",

"btn_css": "#456",

"after_code": "require_script 'fpro_samples'\n\nvial_count = params['Number of Vials to Store']\n\nif User.curr_user.user_groups.map(&:name).join(',').include? \"Lab Personnel - Twinbrook\"\n submit_to_fpro(subj, 'MiniPrep DNA', '7000000605', vial_count) do |s|\n end\nelsif User.curr_user.user_groups.map(&:name).join(',').include? \"Lab Personnel - Building 50\"\n submit_to_fpro(subj, 'MiniPrep DNA', '7000000477', vial_count) do |s|\n end\nelse\n raise(\"This script only allows users who belong to either Twinbrook Lab or Building 50 Lab, please contact the administrators\")\nend",

"after_code_params": [

"UDPM-1450"

]

}

],

"show_options": {

"top": 133,

"left": 167

}

},

{

"name": "Stored",

"uuid": "UDPM-1016",

"owner": "UDPM-67",

"managers": "UDPM-22",

"performers": "UDPM-21",

"show_options": {

"top": 241.9943084716797,

"left": 488.9914855957031

},

"end_task": true

}

],

"entry_point": "UDPM-1015",

"ask_performer": true,

"show_udfs": "UDPM-6,UDPM-5,UDPM-627,UDPM-628,UDPM-1016,UDPM-1332,UDPM-1907,UDPM-1977"

},{

"name": "Collaborators",

"uuid": "UDPM-204",

"subject_type": "UDPM-149",

"state_defs": [

{

"name": "Contact Information",

"uuid": "UDPM-1020",

"owner": "UDPM-67",

"managers": "UDPM-22",

"performers": "UDPM-22",

"tools": [

{

"name": "Update Information",

"uuid": "UDPM-1284",

"input_type": "UDPM-149",

"output_type": "UDPM-149",

"flags": 1,

"options": {

"udfs": [

"UDPM-1696",

"UDPM-1407",

"UDPM-1408",

"UDPM-1409",

"UDPM-1717",

"UDPM-1410",

"UDPM-1392",

"UDPM-1692"

]

}

}

],

"show_options": {

"top": 180,

"left": 399

},

"end_task": true

}

],

"entry_point": "UDPM-1020",

"show_udfs": "UDPM-1410,UDPM-1409,UDPM-1407,UDPM-1408,UDPM-1696"

},{

"name": "Zebrafish Breeding",

"uuid": "UDPM-206",

"subject_type": "UDPM-233",

"state_defs": [

{

"name": "Breeding Information",

"uuid": "UDPM-1026",

"owner": "UDPM-67",

"managers": "UDPM-36",

"performers": "UDPM-33",

"tools": [

{

"name": "Information",

"uuid": "UDPM-1295",

"input_type": "UDPM-233",

"output_type": "UDPM-233",

"flags": 1,

"options": {

"udfs": [

"UDPM-2438",

"UDPM-1972",

"UDPM-2434",

"UDPM-2741",

"UDPM-2404",

"UDPM-2394",

"UDPM-2433",

"UDPM-2432",

"UDPM-2251",

"UDPM-2514"

]

}

}

],

"show_options": {

"top": 74,

"left": 155

},

"end_task": true

}

],

"entry_point": "UDPM-1026",

"show_udfs": "UDPM-2514,UDPM-2404,UDPM-2434,UDPM-2251,UDPM-2394,UDPM-2432,UDPM-2433,UDPM-1972"

},{

"name": "Bioinformatics SOP Editing",

"uuid": "UDPM-207",

"subject_type": "UDPM-240",

"state_defs": [

{

"name": "SOP Upload",

"uuid": "UDPM-1028",

"owner": "UDPM-67",

"managers": "UDPM-27",

"performers": "UDPM-3",

"tools": [

{

"name": "SOP Information",

"uuid": "UDPM-1299",

"input_type": "UDPM-240",

"output_type": "UDPM-240",

"flags": 1,

"options": {

"udfs": [

"UDPM-2102",

"UDPM-649",

"UDPM-2585"

]

}

}

],

"show_options": {

"top": 124,

"left": 191

},

"end_task": true

}

],

"entry_point": "UDPM-1028",

"show_udfs": "UDPM-2102,UDPM-2585,UDPM-649"

},{

"name": "Bedfiles Workflow",

"uuid": "UDPM-215",

"subject_type": "UDPM-238",

"state_defs": [

{

"name": "Bedfiles",

"uuid": "UDPM-1068",

"owner": "UDPM-67",

"managers": "UDPM-27",

"performers": "UDPM-3",

"tools": [

{

"name": "Edit Bedfile Information",

"uuid": "UDPM-1405",

"input_type": "UDPM-238",

"output_type": "UDPM-238",

"flags": 1,

"options": {

"udfs": [

"UDPM-6",

"UDPM-1500",

"UDPM-2566",

"UDPM-1277",

"UDPM-2565",

"UDPM-5"

]

}

}

],

"show_options": {

"top": 91,

"left": 249

},

"end_task": true

}

],

"entry_point": "UDPM-1068",

"show_udfs": "UDPM-6"

},{

"name": "Clone Workflow",

"uuid": "UDPM-216",

"subject_type": "UDPM-266",

"state_defs": [

{

"name": "Cancel",

"uuid": "UDPM-1072",

"owner": "UDPM-67",

"managers": "UDPM-22",

"performers": "UDPM-21",

"show_options": {

"top": 346,

"left": 163

},

"end_task": true

},

{

"name": "Enter Clone Information",

"uuid": "UDPM-1070",

"owner": "UDPM-67",

"managers": "UDPM-22",

"performers": "UDPM-21",

"tools": [

{

"name": "Edit Information",

"uuid": "UDPM-1503",

"input_type": "UDPM-266",

"output_type": "UDPM-266",

"flags": 1,

"options": {

"udfs": [

"UDPM-1656",

"UDPM-2195",

"UDPM-1800",

"UDPM-1961",

"UDPM-1966",

"UDPM-1922",

"UDPM-1303",

"UDPM-1165",

"UDPM-1174",

"UDPM-1698",

"UDPM-2380"

]

}

},

{

"name": "Request Primer Order",

"uuid": "UDPM-1424",

"input_type": "UDPM-266",

"output_type": "UDPM-217",

"flags": 0,

"before_code": "s = subj.get_value(\"Sequencing Primers\")\n\ntable = \"<html>\n<table border='1'>\n\n<tr><td>Primer Name</td><td>Sequence</td><td>Amount</td></tr>\n<tr><td></td><td>#{s}</td><td></td></tr>\n<tr><td></td><td></td><td></td></tr>\n</table>\n</html>\"\n\nparams[:defaults] = {\n 'Requester'=> User.curr_user,\n 'Primer ordering'=> table\n}\n",

"options": {

"obj_type": "SubjectType"

}

},

{

"name": "Prepare Entry Vector",

"uuid": "UDPM-1412",

"input_type": "UDPM-266",

"output_type": "UDPM-193",

"flags": 0,

"before_code": "insert_name = subj.get_value(\"Insert Name\")\ngene = subj.get_value(\"Gene\")\ndes = subj.get_value(\"Description\")\n\nparams[:defaults] = {\n 'Insert Name'=> insert_name,\n 'Gene' => gene,\n 'Description' => des\n}",

"options": {

"obj_type": "SubjectType"

}

},

{

"name": "Prepare Glycerol Stock",

"uuid": "UDPM-1413",

"input_type": "UDPM-266",

"output_type": "UDPM-171",

"flags": 0,

"before_code": "des = subj.get_value(\"Description\")\n\nparams[:defaults] = {\n 'Description' => des\n}",

"options": {

"obj_type": "SubjectType"

}

},

{

"name": "Prepare MiniPrep",

"uuid": "UDPM-1419",

"input_type": "UDPM-266",

"output_type": "UDPM-249",

"flags": 0,

"options": {

"obj_type": "SubjectType"

}

},

{

"name": "MaxiPrep Information",

"uuid": "UDPM-1425",

"input_type": "UDPM-266",

"output_type": "UDPM-266",

"flags": 1,

"options": {

"udfs": [

"UDPM-1977",

"UDPM-1332",

"UDPM-1016",

"UDPM-627",

"UDPM-628"

]

}

}

],

"next_states": [

{

"state_name": "Cancel",

"state_uuid": "UDPM-1072",

"btn_text": "Cancel",

"btn_css": "rgb(13, 73, 123)",

"btn_scale": "large"

},

{

"state_name": "Stored MaxiPrep",

"state_uuid": "UDPM-1071",

"btn_text": "Store MaxiPrep",

"btn_css": "rgb(13, 73, 123)",

"after_code": "require_script 'fpro_samples'\n\nvial_count = params['Number of Vials to Store']\n#evb = subj.get_value('Entry Vector Backbone')\nif User.curr_user.user_groups.map(&:name).join(',').include? \"Lab Personnel - Twinbrook\"\n submit_to_fpro(subj, 'MaxiPrep DNA', '255', vial_count) do |s|\n #s.set_value('Vector Information', evb.name)\n s.set_value('Insert Information', subj.get_value('Insert Name'))\n #s.set_value('Resistance Marker', evb.get_value('Resistance Marker'))\n end\nelsif User.curr_user.user_groups.map(&:name).join(',').include? \"Lab Personnel - Building 50\"\n submit_to_fpro(subj, 'MaxiPrep DNA', '7000000476', vial_count) do |s|\n #s.set_value('Vector Information', evb.name)\n s.set_value('Insert Information', subj.get_value('Insert Name'))\n #s.set_value('Resistance Marker', evb.get_value('Resistance Marker'))\n end\nelse\n raise(\"This script only allows users who belong to either Twinbrook Lab or Building 50 Lab, please contact the administrators\")\nend",

"btn_scale": "large",

"after_code_params": [

"UDPM-1450"

]

}

],

"show_options": {

"top": 86,

"left": 112

}

},

{

"name": "Stored MaxiPrep",

"uuid": "UDPM-1071",

"owner": "UDPM-67",

"managers": "UDPM-22",

"performers": "UDPM-21",

"tools": [

{

"name": "Prepare Entry Vector",

"uuid": "UDPM-1415",

"input_type": "UDPM-266",

"output_type": "UDPM-193",

"flags": 0,

"before_code": "insert_name = subj.get_value(\"Insert Name\")\ngene = subj.get_value(\"Gene\")\ndes = subj.get_value(\"Description\")\n\nparams[:defaults] = {\n 'Insert Name'=> insert_name,\n 'Gene' => gene,\n 'Description' => des\n}",

"options": {

"obj_type": "SubjectType"

}

},

{

"name": "Prepare Glycerol Stock",

"uuid": "UDPM-1416",

"input_type": "UDPM-266",

"output_type": "UDPM-171",

"flags": 0,

"before_code": "des = subj.get_value(\"Description\")\n\nparams[:defaults] = {\n 'Description' => des\n}",

"options": {

"obj_type": "SubjectType"

}

},

{

"name": "Prepare MiniPrep",

"uuid": "UDPM-1420",

"input_type": "UDPM-266",

"output_type": "UDPM-249",

"flags": 0,

"options": {

"obj_type": "SubjectType"

}

}

],

"show_options": {

"top": 93,

"left": 525

},

"end_task": true

}

],

"entry_point": "UDPM-1070",

"ask_performer": true,

"show_udfs": "UDPM-2195,UDPM-1922,UDPM-1698,UDPM-1165,UDPM-1174,UDPM-1303,UDPM-1656,UDPM-2380,UDPM-1800,UDPM-1966,UDPM-1961"

},{

"name": "Clinical Notes",

"uuid": "UDPM-217",

"subject_type": "UDPM-267",

"state_defs": [

{

"name": "Clinical Notes",

"uuid": "UDPM-1076",

"owner": "UDPM-67",

"managers": "UDPM-49",

"performers": "UDPM-5",

"tools": [

{

"name": "Update Clinical Notes",

"uuid": "UDPM-1443",

"input_type": "UDPM-267",

"output_type": "UDPM-267",

"flags": 1,

"options": {

"udfs": [

"UDPM-2829",

"UDPM-2312",

"UDPM-2831",

"UDPM-2832",

"UDPM-2833",

"UDPM-2834",

"UDPM-2835",

"UDPM-2842",

"UDPM-2836",

"UDPM-2843",

"UDPM-2837",

"UDPM-2838",

"UDPM-2839"

]

}

}

],

"show_options": {

"top": 180,

"left": 400

},

"end_task": true

}

],

"entry_point": "UDPM-1076",

"show_udfs": "UDPM-6,UDPM-2829,UDPM-2839,UDPM-2831,UDPM-2842,UDPM-2832,UDPM-2834,UDPM-2835,UDPM-2843,UDPM-2833,UDPM-2836,UDPM-2837,UDPM-2838,UDPM-2312"

},{

"name": "Plasmid Reagent Workflow",

"uuid": "UDPM-218",

"subject_type": "UDPM-177",

"state_defs": [

{

"name": "Information",

"uuid": "UDPM-1078",

"owner": "UDPM-67",

"managers": "UDPM-22",

"performers": "UDPM-4",

"tools": [

{

"name": "Create MiniPrep",

"uuid": "UDPM-1444",

"input_type": "UDPM-177",

"output_type": "UDPM-249",

"flags": 0,

"options": {

"obj_type": "SubjectType"

}

},

{

"name": "Edit Reagent Information",

"uuid": "UDPM-1445",

"input_type": "UDPM-177",

"output_type": "UDPM-177",

"flags": 1,

"options": {

"udfs": [

"UDPM-1769",

"UDPM-1768",

"UDPM-1778",

"UDPM-1770",

"UDPM-1798",

"UDPM-1791",

"UDPM-1792",

"UDPM-1776",

"UDPM-1182",

"UDPM-901",

"UDPM-1773",

"UDPM-1774",

"UDPM-1775",

"UDPM-1803",

"UDPM-1804"

]

}

},

{

"name": "Create MaxiPrep",

"uuid": "UDPM-1447",

"input_type": "UDPM-177",

"output_type": "UDPM-269",

"flags": 0,

"options": {

"obj_type": "SubjectType"

}

},

{

"name": "Make Glycerol Stock",

"uuid": "UDPM-1452",

"input_type": "UDPM-177",

"output_type": "UDPM-171",

"flags": 0,

"options": {

"obj_type": "SubjectType"

}

}

],

"show_options": {

"top": 151,

"left": 345

},

"end_task": true

}

],

"entry_point": "UDPM-1078",

"ask_performer": true

},{

"name": "MaxiPrep Workflow",

"uuid": "UDPM-219",

"subject_type": "UDPM-269",

"state_defs": [

{

"name": "MaxiPrep Information",

"uuid": "UDPM-1079",

"owner": "UDPM-67",

"managers": "UDPM-22",

"performers": "UDPM-4",

"tools": [

{

"name": "MaxiPrep Information",

"uuid": "UDPM-1446",

"input_type": "UDPM-269",

"output_type": "UDPM-269",

"flags": 1,

"options": {

"udfs": [

"UDPM-2841",

"UDPM-452",

"UDPM-1977",

"UDPM-1016",

"UDPM-627",

"UDPM-628"

]

}

}

],

"next_states": [

{

"state_name": "Stored",

"state_uuid": "UDPM-1080",

"btn_text": "Store",

"btn_css": "rgb(13, 73, 123)",

"after_code": "require_script 'fpro_samples'\n\nvial_count = params['Number of Vials to Store']\npr = subj.get_value('Reagent Used')\nif User.curr_user.user_groups.map(&:name).join(',').include? \"Lab Personnel - Twinbrook\"\n submit_to_fpro(subj, 'MaxiPrep DNA', '7000000404', vial_count) do |s|\n s.name = subj.name #changed from 'pr.name'\n s.set_value('Resistance Marker', pr.get_value('Resistance Marker'))\n end\nelsif User.curr_user.user_groups.map(&:name).join(',').include? \"Lab Personnel - Building 50\"\n submit_to_fpro(subj, 'MaxiPrep DNA', '7000000476', vial_count) do |s|\n s.name = subj.name #changed from 'pr.name'\n s.set_value('Resistance Marker', pr.get_value('Resistance Marker'))\n end\nelse\n raise(\"This script only allows users who belong to either Twinbrook Lab or Building 50 Lab, please contact the administrators\")\nend\n",

"btn_scale": "large",

"after_code_params": [

"UDPM-1450"

],

"conditions": [

{

"name": "MaxiPrep Storage&rarr;Concentration (ng/ul)",

"condition": "Not Empty",

"value": "",

"message": "Volume and concentration must be entered in order to store",

"subject_type": "UDPM-269",

"udf": "UDPM-1977"

},

{

"name": "MaxiPrep Storage&rarr;Volume (uL)",

"condition": "Not Empty",

"value": "",

"message": "Volume and concentration must be entered in order to store",

"subject_type": "UDPM-269",

"udf": "UDPM-452"

}

]

}

],

"show_options": {

"top": 91,

"left": 109

}

},

{

"name": "Stored",

"uuid": "UDPM-1080",

"owner": "UDPM-67",

"managers": "UDPM-22",

"performers": "UDPM-4",

"show_options": {

"top": 236,

"left": 440

},

"end_task": true

}

],

"entry_point": "UDPM-1079",

"ask_performer": true

},{

"name": "Transcriptome Workflow",

"uuid": "UDPM-211",

"subject_type": "UDPM-255",

"state_defs": [

{

"name": "Cancelled",

"uuid": "UDPM-1053",

"owner": "UDPM-67",

"managers": "UDPM-22",

"performers": "UDPM-21",

"show_options": {

"top": 218,

"left": 76

},

"end_task": true

},

{

"name": "Files Returned",

"uuid": "UDPM-1052",

"owner": "UDPM-67",

"managers": "UDPM-22",

"performers": "UDPM-21",

"tools": [

{

"name": "Upload to Patient Bioinformatics Files",

"uuid": "UDPM-1539",

"input_type": "UDPM-255",

"output_type": "UDPM-235",

"flags": 0,

"options": {

"obj_type": "SubjectType"

}

},

{

"name": "File Information",

"uuid": "UDPM-1356",

"input_type": "UDPM-255",

"output_type": "UDPM-255",

"flags": 1,

"options": {

"udfs": [

"UDPM-1578",

"UDPM-2871",

"UDPM-371",

"UDPM-168",

"UDPM-2927",

"UDPM-2758"

]

}

}

],

"show_options": {

"top": 291,

"left": 166

},

"end_task": true

},

{

"name": "Prepare Samples for Shipment",

"uuid": "UDPM-1049",

"owner": "UDPM-67",

"managers": "UDPM-22",

"performers": "UDPM-21",

"tools": [

{

"name": "Shipping Information",

"uuid": "UDPM-1355",

"input_type": "UDPM-255",

"output_type": "UDPM-255",

"flags": 1,

"options": {

"udfs": [

"UDPM-1515",

"UDPM-1797",

"UDPM-121",

"UDPM-2679"

]

}

},

{

"name": "Sample Information",

"uuid": "UDPM-1354",

"input_type": "UDPM-255",

"output_type": "UDPM-255",

"flags": 1,

"options": {

"udfs": [

"UDPM-6",

"UDPM-2195",

"UDPM-2761",

"UDPM-1040",

"UDPM-2759",

"UDPM-1977",

"UDPM-452",

"UDPM-1016",

"UDPM-627",

"UDPM-628",

"UDPM-1959"

]

}

}

],

"next_states": [

{

"state_name": "Cancelled",

"state_uuid": "UDPM-1053",

"btn_text": "Cancelled",

"btn_css": "rgb(13, 73, 123)",

"after_code": "subj.set_value('Rationale/Reasoning',params['Rationale/Reasoning'])",

"btn_scale": "large",

"after_code_params": [

"UDPM-883"

]

},

{

"state_name": "Samples Sent to Sequencing Center",

"state_uuid": "UDPM-1050",

"btn_text": "Samples Shipped",

"btn_css": "rgb(13, 73, 123)",

"after_code": "self.next_state_performer = find_user('[redacted]')",

"btn_scale": "large"

}

],

"show_options": {

"top": 48,

"left": 67

}

},

{

"name": "Samples Received at Sequencing Center",

"uuid": "UDPM-1051",

"owner": "UDPM-67",

"managers": "UDPM-22",

"performers": "UDPM-21",

"duration": 70.0,

"tools": [

{

"name": "Upload Analysis Report",

"uuid": "UDPM-1496",

"input_type": "UDPM-255",

"output_type": "UDPM-255",

"flags": 1,

"options": {

"udfs": [

"UDPM-2871"

]

}

},

{

"name": "QC Information",

"uuid": "UDPM-1451",

"input_type": "UDPM-255",

"output_type": "UDPM-255",

"description": "Quality Check Information",

"flags": 1,

"options": {

"udfs": [

"UDPM-2071",

"UDPM-2070",

"UDPM-2768",

"UDPM-2769"

]

}

}

],

"next_states": [

{

"state_name": "Files Returned",

"state_uuid": "UDPM-1052",

"btn_text": "FastQ Files Received",

"btn_css": "rgb(13, 73, 123)",

"after_code": "self.next_state_performer = find_user('[redacted]')",

"btn_scale": "large"

},

{

"state_name": "Cancelled",

"state_uuid": "UDPM-1053",

"btn_text": "Cancelled",

"btn_css": "rgb(13, 73, 123)",

"after_code": "subj.set_value('Rationale/Reasoning',params['Rationale/Reasoning'])",

"btn_scale": "large",

"after_code_params": [

"UDPM-883"

]

}

],

"show_options": {

"top": 289,

"left": 443

}

},

{

"name": "Samples Sent to Sequencing Center",

"uuid": "UDPM-1050",

"owner": "UDPM-67",

"managers": "UDPM-22",

"performers": "UDPM-21",

"duration": 14.0,

"tools": [

{

"name": "Update Shipment Information",

"uuid": "UDPM-1357",

"input_type": "UDPM-255",

"output_type": "UDPM-255",

"flags": 1,

"options": {

"udfs": [

"UDPM-1515",

"UDPM-1797",

"UDPM-121",

"UDPM-2679"

]

}

}

],

"next_states": [

{

"state_name": "Samples Received at Sequencing Center",

"state_uuid": "UDPM-1051",

"btn_text": "Samples Received at Sequencing Center",

"btn_css": "rgb(13, 73, 123)",

"after_code": "self.next_state_performer = find_user('[redacted]')",

"btn_scale": "large"

},

{

"state_name": "Cancelled",

"state_uuid": "UDPM-1053",

"btn_text": "Cancelled",

"btn_css": "rgb(13, 73, 123)",

"after_code": "subj.set_value('Rationale/Reasoning',params['Rationale/Reasoning'])",

"btn_scale": "large",

"after_code_params": [

"UDPM-883"

]

}

],

"show_options": {

"top": 124,

"left": 466

}

}

],

"entry_point": "UDPM-1049",

"show_udfs": "UDPM-2195,UDPM-6,UDPM-2761,UDPM-1040"

},{

"name": "Radiology Review",

"uuid": "UDPM-205",

"subject_type": "UDPM-246",

"state_defs": [

{

"name": "Complete",

"uuid": "UDPM-1121",

"owner": "UDPM-7",

"managers": "UDPM-64",

"performers": "UDPM-31",

"show_options": {

"top": 282,

"left": 622

},

"end_task": true

},

{

"name": "Edit Review",

"uuid": "UDPM-1120",

"owner": "UDPM-7",

"managers": "UDPM-64",

"performers": "UDPM-64",

"tools": [

{

"name": "Edit Review",

"uuid": "UDPM-1548",

"input_type": "UDPM-246",

"output_type": "UDPM-246",

"flags": 1,

"options": {

"udfs": [

"UDPM-2688",

"UDPM-2646",

"UDPM-2687",

"UDPM-2644",

"UDPM-916",

"UDPM-2647",

"UDPM-2686",

"UDPM-2648"

]

}

}

],

"next_states": [

{

"state_name": "Complete",

"state_uuid": "UDPM-1121",

"btn_text": "Complete",

"btn_css": "rgb(13, 73, 123)",

"btn_scale": "large"

}

],

"show_options": {

"top": 245,

"left": 349

}

},

{

"name": "Radiology Review",

"uuid": "UDPM-1023",

"owner": "UDPM-67",

"managers": "UDPM-64",

"performers": "UDPM-64",

"tools": [

{

"name": "Edit Review",

"uuid": "UDPM-1290",

"input_type": "UDPM-246",

"output_type": "UDPM-246",

"flags": 1,

"options": {

"udfs": [

"UDPM-6",

"UDPM-2688",

"UDPM-2646",

"UDPM-2687",

"UDPM-2644",

"UDPM-916",

"UDPM-2647",

"UDPM-2686",

"UDPM-2648"

]

}

}

],

"next_states": [

{

"state_name": "Edit Review",

"state_uuid": "UDPM-1120",

"btn_text": "Action",

"btn_css": "rgb(13, 73, 123)"

}

],

"show_options": {

"top": 55,

"left": 50

}

}

],

"entry_point": "UDPM-1023",

"ask_performer": true,

"show_udfs": "UDPM-6,UDPM-916,UDPM-2644,UDPM-2646,UDPM-2686,UDPM-2647,UDPM-2648,UDPM-2688,UDPM-2687"

},{

"name": "Letters",

"uuid": "UDPM-209",

"subject_type": "UDPM-253",

"state_defs": [

{

"name": "Complete",

"uuid": "UDPM-1034",

"owner": "UDPM-67",

"managers": "UDPM-49",

"performers": "UDPM-49",

"show_options": {

"top": 213,

"left": 474

},

"end_task": true

},

{

"name": "Hold - Pending Receipt of Missing Records",

"uuid": "UDPM-1035",

"owner": "UDPM-67",

"managers": "UDPM-49",

"performers": "UDPM-49",

"tools": [

{

"name": "Information Hold Update",

"uuid": "UDPM-1327",

"input_type": "UDPM-253",

"output_type": "UDPM-253",

"flags": 1,

"options": {

"udfs": [

"UDPM-2708",

"UDPM-2707"

]

}

}

],

"next_states": [

{

"state_name": "Complete",

"state_uuid": "UDPM-1034",

"btn_text": "Complete",

"btn_css": "#456",

"after_code": "# Script to run After Transition is executed",

"before_code": "# Script to run Before Transition is executed"

}

],

"show_options": {

"top": 349,

"left": 430

}

},

{

"name": "Information Received",

"uuid": "UDPM-1036",

"owner": "UDPM-67",

"managers": "UDPM-49",

"performers": "UDPM-49",

"tools": [

{

"name": "Upload PII Files",

"uuid": "UDPM-1540",

"input_type": "UDPM-253",

"output_type": "UDPM-172",

"flags": 0,

"options": {

"obj_type": "SubjectType"

}

},

{

"name": "Receive Information",

"uuid": "UDPM-1328",

"input_type": "UDPM-253",

"output_type": "UDPM-253",

"flags": 1,

"options": {

"udfs": [

"UDPM-1991",

"UDPM-114",

"UDPM-2708",

"UDPM-2707"

]

}

}

],

"next_states": [

{

"state_name": "Hold - Pending Receipt of Missing Records",

"state_uuid": "UDPM-1035",

"btn_text": "Hold for Incomplete Information",

"btn_css": "#456",

"after_code": "# Script to run After Transition is executed",

"before_code": "# Script to run Before Transition is executed\nparams[:ask_next_performer] = true",

"btn_scale": "large"

},

{

"state_name": "Complete",

"state_uuid": "UDPM-1034",

"btn_text": "Complete - No Further Action Required",

"btn_css": "rgb(13, 73, 123)",

"btn_scale": "large"

}

],

"show_options": {

"top": 404,

"left": 67

}

},

{

"name": "Letter Request",

"uuid": "UDPM-1037",

"owner": "UDPM-67",

"managers": "UDPM-49",

"performers": "UDPM-49",

"tools": [

{

"name": "Requested Letter Information",

"uuid": "UDPM-1329",

"input_type": "UDPM-253",

"output_type": "UDPM-253",

"flags": 1,

"options": {

"udfs": [

"UDPM-6",

"UDPM-2711",

"UDPM-438",

"UDPM-2749",

"UDPM-2723",

"UDPM-2722",

"UDPM-2707"

]

}

}

],

"next_states": [

{

"state_name": "Letter Sent",

"state_uuid": "UDPM-1038",

"btn_text": "Send Letter",

"btn_css": "#456",

"after_code": "# Script to run After Transition is executed\nsubj.set_value('Letter - Date Sent', params['Letter - Date Sent'])\nsubj.set_value('Upload Letter', params['Upload Letter'])\nsubj.set_value('Communication Notes', params['Communication Notes'])",

"before_code": "# Script to run Before Transition is executed\nparams[:ask_next_performer] = true",

"btn_scale": "large",

"after_code_params": [

"UDPM-214",

"UDPM-2709",

"UDPM-2707"

]

}

],

"show_options": {

"top": 33,

"left": 53

}

},

{

"name": "Letter Sent",

"uuid": "UDPM-1038",

"owner": "UDPM-67",

"managers": "UDPM-49",

"performers": "UDPM-49",

"next_states": [

{

"state_name": "Complete",

"state_uuid": "UDPM-1034",

"btn_text": "Complete",

"btn_css": "rgb(13, 73, 123)",

"btn_scale": "large"

},

{

"state_name": "Information Received",

"state_uuid": "UDPM-1036",

"btn_text": "Information Received",

"btn_css": "rgb(13, 73, 123)",

"before_code": "params[:ask_next_performer] = true",

"btn_scale": "large"

}

],

"show_options": {

"top": 188,

"left": 67

}

}

],

"entry_point": "UDPM-1037",

"ask_performer": true,

"show_udfs": "UDPM-438,UDPM-2722,UDPM-2711,UDPM-2749,UDPM-2723,UDPM-214"

},{

"name": "Primer Ordering",

"uuid": "UDPM-182",

"subject_type": "UDPM-217",

"state_defs": [

{

"name": "Cancel Order",

"uuid": "UDPM-1054",

"owner": "UDPM-67",

"managers": "UDPM-22",

"performers": "UDPM-4",

"show_options": {

"top": 266,

"left": 150

},

"end_task": true

},

{

"name": "Primers Ordered",

"uuid": "UDPM-882",

"owner": "UDPM-67",

"managers": "UDPM-22",

"performers": "UDPM-21",

"duration": 14.0,

"next_states": [

{

"state_name": "Primers Received",

"state_uuid": "UDPM-883",

"btn_text": "Primers Received",

"btn_css": "#456",

"after_code": "subj.set_value('Date Received', params[\"Date Received\"])",

"btn_scale": "large",

"after_code_params": [

"UDPM-114"

]

}

],

"show_options": {

"top": 45,

"left": 470

}

},

{

"name": "Primers Received",

"uuid": "UDPM-883",

"owner": "UDPM-67",

"managers": "UDPM-22",

"performers": "UDPM-21",

"show_options": {

"top": 143.99998474121094,

"left": 791.0000305175781

},

"end_task": true

},

{

"name": "Request Primer Order",

"uuid": "UDPM-881",

"owner": "UDPM-67",

"managers": "UDPM-8",

"performers": "UDPM-41",

"tools": [

{

"name": "Design Information",

"uuid": "UDPM-1133",

"input_type": "UDPM-217",

"output_type": "UDPM-217",

"flags": 1,

"options": {

"udfs": [

"UDPM-2381",

"UDPM-2383",

"UDPM-2382",

"UDPM-1344"

]

}

}

],

"next_states": [

{

"state_name": "Cancel Order",

"state_uuid": "UDPM-1054",

"btn_text": "Cancel Order",

"btn_css": "rgb(13, 73, 123)",

"btn_scale": "large"

},

{

"state_name": "Primers Ordered",

"state_uuid": "UDPM-882",

"btn_text": "Primers Ordered",

"btn_css": "#456",

"after_code": "#send_email(subj.created_by, find_email_template(\"Primers Ordered\"), subj)\nself.next_state_performer = subj.created_by\n#subj.set_value('Primers Ordered', params['Primers Ordered'])\nsubj.set_value('Date Ordered', params['Date Ordered'])\n\np = subj.get_value('Primers Ordered')\n\n#if p == 'Yes'\nsend_email(subj.created_by, find_email_template(\"Primers Ordered\"), subj) \n#end",

"btn_scale": "large",

"after_code_params": [

"UDPM-1472"

]

}

],

"show_options": {

"top": 44,

"left": 66

}

}

],

"entry_point": "UDPM-881",

"show_udfs": "UDPM-6,UDPM-2382,UDPM-1344,UDPM-2124"

},{

"name": "Vector Mutagenesis Workflow",

"uuid": "UDPM-191",

"subject_type": "UDPM-228",

"state_defs": [

{

"name": "Cancelled",

"uuid": "UDPM-971",

"owner": "UDPM-67",

"managers": "UDPM-22",

"performers": "UDPM-21",

"show_options": {

"top": 254,

"left": 174

},

"end_task": true

},

{

"name": "Mutagenesis",

"uuid": "UDPM-942",

"owner": "UDPM-67",

"managers": "UDPM-22",

"performers": "UDPM-21",

"tools": [

{

"name": "Mutagenesis Information",

"uuid": "UDPM-1199",

"input_type": "UDPM-228",

"output_type": "UDPM-228",

"flags": 1,

"options": {

"udfs": [

"UDPM-2569",

"UDPM-1802",

"UDPM-2419",

"UDPM-2481",

"UDPM-2537",

"UDPM-455",

"UDPM-1280",

"UDPM-1693",

"UDPM-5"

]

}

},

{

"name": "MaxiPrep Information",

"uuid": "UDPM-1200",

"input_type": "UDPM-228",

"output_type": "UDPM-228",

"flags": 1,

"options": {

"udfs": [

"UDPM-1977",

"UDPM-1332",

"UDPM-1016",

"UDPM-627",

"UDPM-628"

]

}

},

{

"name": "Upload AB1 Files",

"uuid": "UDPM-1278",

"input_type": "UDPM-228",

"output_type": "UDPM-250",

"flags": 0,

"options": {

"obj_type": "SubjectType"

}

},

{

"name": "Create MiniPrep",

"uuid": "UDPM-1280",

"input_type": "UDPM-228",

"output_type": "UDPM-249",

"flags": 0,

"options": {

"obj_type": "SubjectType"

}

},

{

"name": "Create Glycerol Stock",

"uuid": "UDPM-1281",

"input_type": "UDPM-228",

"output_type": "UDPM-171",

"flags": 0,

"options": {

"obj_type": "SubjectType"

}

}

],

"next_states": [

{

"state_name": "Store MaxiPrep",

"state_uuid": "UDPM-943",

"btn_text": "Store MaxiPrep",

"btn_css": "#456",

"after_code": "require_script 'fpro_samples'\n\nvial_count = params['Number of Vials to Store']\nev = subj.get_value('Entry Vector')\nevb = ev.get_value('Entry Vector Backbone')\nif User.curr_user.user_groups.map(&:name).join(',').include? \"Lab Personnel - Twinbrook\"\n submit_to_fpro(subj, 'MaxiPrep DNA', '7000000404', vial_count) do |s|\n s.set_value('Concentration (ng/ul)', subj.get_value('Concentration (ng/ul)'))\n s.set_value('Vector Information', evb.name)\n s.set_value('Created By', ev.get_value('Plasmid made by'))\n s.set_value('Insert Information', ev.get_value('Insert Name'))\n end\nelsif User.curr_user.user_groups.map(&:name).join(',').include? \"Lab Personnel - Building 50\"\n submit_to_fpro(subj, 'MaxiPrep DNA', '7000000476', vial_count) do |s|\n s.set_value('Concentration (ng/ul)', subj.get_value('Concentration (ng/ul)'))\n s.set_value('Vector Information', evb.name)\n s.set_value('Created By', ev.get_value('Plasmid made by'))\n s.set_value('Insert Information', ev.get_value('Insert Name'))\n end\nelse\n raise(\"This script only allows users who belong to either Twinbrook Lab or Building 50 Lab, please contact the administrators\")\nend",

"btn_scale": "large",

"after_code_params": [

"UDPM-1450"

]

},

{

"state_name": "Cancelled",

"state_uuid": "UDPM-971",

"btn_text": "Cancel",

"btn_css": "#456",

"after_code": "subj.set_value('Rationale/Reasoning', params['Rationale/Reasoning'])",

"btn_scale": "large",

"after_code_params": [

"UDPM-883"

]

}

],

"show_options": {

"top": 76,

"left": 166.00001525878906

}

},

{

"name": "Store MaxiPrep",

"uuid": "UDPM-943",

"owner": "UDPM-67",

"managers": "UDPM-22",

"performers": "UDPM-21",

"tools": [

{

"name": "Prepare Glycerol Stock",

"uuid": "UDPM-1197",

"input_type": "UDPM-228",

"output_type": "UDPM-171",

"flags": 0,

"options": {

"obj_type": "SubjectType"

}

},

{

"name": "Begin Destination Vector",

"uuid": "UDPM-1198",

"input_type": "UDPM-228",

"output_type": "UDPM-168",

"flags": 0,

"options": {

"obj_type": "SubjectType"

}

},

{

"name": "Begin New Mutagenesis",

"uuid": "UDPM-1222",

"input_type": "UDPM-228",

"output_type": "UDPM-228",

"flags": 0,

"options": {

"obj_type": "SubjectType"

}

}

],

"show_options": {

"top": 200,

"left": 492.00001525878906

},

"end_task": true

}

],

"entry_point": "UDPM-942",

"ask_performer": true,

"show_udfs": "UDPM-6,UDPM-5,UDPM-1925,UDPM-1968"

},{

"name": "PhenomeCentral Submission Workflow",

"uuid": "UDPM-181",

"subject_type": "UDPM-216",

"state_defs": [

{

"name": "Cancel Submission",

"uuid": "UDPM-880",

"owner": "UDPM-67",

"managers": "UDPM-9",

"performers": "UDPM-49",

"show_options": {

"top": 260,

"left": 225

},

"end_task": true

},

{

"name": "Data for Submission",

"uuid": "UDPM-878",

"owner": "UDPM-67",

"managers": "UDPM-9",

"performers": "UDPM-55",

"tools": [

{

"name": "Data information",

"uuid": "UDPM-1408",

"input_type": "UDPM-216",

"output_type": "UDPM-216",

"flags": 1,

"options": {

"udfs": [

"UDPM-2504",

"UDPM-2505",

"UDPM-2377",

"UDPM-2680",

"UDPM-2681",

"UDPM-2378",

"UDPM-2374"

]

}

}

],

"next_states": [

{

"state_name": "Cancel Submission",

"state_uuid": "UDPM-880",

"btn_text": "Cancel",

"btn_css": "#456"

},

{

"state_name": "Ready for Submission",

"state_uuid": "UDPM-879",

"btn_text": "Ready for Submission",

"btn_css": "#456",

"after_code": "subj.set_value('Date Submitted to PhenomeCentral', params['Date Submitted to PhenomeCentral'])\n",

"btn_scale": "large",

"after_code_params": [

"UDPM-2376"

]

}

],

"show_options": {

"top": 92,

"left": 18

}

},

{

"name": "Ready for Submission",

"uuid": "UDPM-879",

"owner": "UDPM-67",

"managers": "UDPM-9",

"performers": "UDPM-3",

"next_states": [

{

"state_name": "Submitted",

"state_uuid": "UDPM-877",

"btn_text": "Data Submitted",

"btn_css": "#456"

},

{

"state_name": "Cancel Submission",

"state_uuid": "UDPM-880",

"btn_text": "Cancel",

"btn_css": "#456"

}

],

"show_options": {

"top": 99,

"left": 437

}

},

{

"name": "Submitted",

"uuid": "UDPM-877",

"owner": "UDPM-67",

"managers": "UDPM-9",

"performers": "UDPM-49",

"tools": [

{

"name": "Updated Phenotype",

"uuid": "UDPM-1287",

"input_type": "UDPM-216",

"output_type": "UDPM-216",

"flags": 1,

"options": {

"udfs": [

"UDPM-2680",

"UDPM-2681"

]

}

}

],

"show_options": {

"top": 296,

"left": 651

},

"end_task": true

}

],

"entry_point": "UDPM-878"

},{

"name": "Diagnosis Workflow",

"uuid": "UDPM-178",

"subject_type": "UDPM-185",

"state_defs": [

{

"name": "Diagnosis",

"uuid": "UDPM-863",

"owner": "UDPM-67",

"managers": "UDPM-9",

"performers": "UDPM-49",

"tools": [

{

"name": "Diagnosis Information",

"uuid": "UDPM-1096",

"input_type": "UDPM-185",

"output_type": "UDPM-185",

"flags": 1,

"options": {

"udfs": [

"UDPM-1875",

"UDPM-2710",

"UDPM-2408",

"UDPM-2406",

"UDPM-2411",

"UDPM-2184",

"UDPM-2292",

"UDPM-2409",

"UDPM-2410",

"UDPM-2412",

"UDPM-2413",

"UDPM-2295",

"UDPM-191",

"UDPM-2374"

]

}

}

],

"show_options": {

"top": 179,

"left": 400

},

"end_task": true

}

],

"entry_point": "UDPM-863",

"show_udfs": "UDPM-2292,UDPM-2184,UDPM-2408,UDPM-2411,UDPM-2413,UDPM-2412,UDPM-2295,UDPM-2410,UDPM-2409,UDPM-2374,UDPM-2913"

},{

"name": "Gene List Workflow",

"uuid": "UDPM-214",

"subject_type": "UDPM-256",

"state_defs": [

{

"name": "Gene List",

"uuid": "UDPM-1067",

"owner": "UDPM-67",

"managers": "UDPM-30",

"performers": "UDPM-30",

"tools": [

{

"name": "Edit Gene List",

"uuid": "UDPM-1402",

"input_type": "UDPM-256",

"output_type": "UDPM-256",

"flags": 1,

"options": {

"udfs": [

"UDPM-2124",

"UDPM-2774",

"UDPM-2764",

"UDPM-540"

]

}

}

],

"show_options": {

"top": 68,

"left": 199

},

"end_task": true

}

],

"entry_point": "UDPM-1067",

"show_udfs": "UDPM-2764,UDPM-2774,UDPM-540,UDPM-2124"

},{

"name": "ChIP-Seq Workflow",

"uuid": "UDPM-210",

"subject_type": "UDPM-254",

"state_defs": [

{

"name": "Antibody Validation",

"uuid": "UDPM-1045",

"owner": "UDPM-67",

"managers": "UDPM-22",

"performers": "UDPM-21",

"tools": [

{

"name": "Validation Information",

"uuid": "UDPM-1351",

"input_type": "UDPM-254",

"output_type": "UDPM-254",

"flags": 1,

"options": {

"udfs": [

"UDPM-2755",

"UDPM-2756",

"UDPM-2939"

]

}

}

],

"next_states": [

{

"state_name": "Send for ChIP-Seq",

"state_uuid": "UDPM-1046",

"btn_text": "Sent for ChIP-Seq",

"btn_css": "rgb(13, 73, 123)",

"btn_scale": "large"

},

{

"state_name": "Sequencing Cancelled",

"state_uuid": "UDPM-1048",

"btn_text": "Cancelled",

"btn_css": "rgb(13, 73, 123)",

"after_code": "subj.set_value('Rationale/Reasoning',params['Rationale/Reasoning'])",

"btn_scale": "large",

"after_code_params": [

"UDPM-883"

]

}

],

"show_options": {

"top": 200,

"left": 740

}

},

{

"name": "Files Returned",

"uuid": "UDPM-1047",

"owner": "UDPM-67",

"managers": "UDPM-22",

"performers": "UDPM-21",

"tools": [

{

"name": "Results Summary",

"uuid": "UDPM-1523",

"input_type": "UDPM-254",

"output_type": "UDPM-254",

"flags": 1,

"options": {

"udfs": [

"UDPM-2925"

]

}

},

{

"name": "File Upload",

"uuid": "UDPM-1522",

"input_type": "UDPM-254",

"output_type": "UDPM-254",

"flags": 1,

"options": {

"udfs": [

"UDPM-371",

"UDPM-168",

"UDPM-2927",

"UDPM-2928",

"UDPM-2929",

"UDPM-2930",

"UDPM-2931",

"UDPM-2926",

"UDPM-2934"

]

}

}

],

"show_options": {

"top": 412.9943084716797,

"left": 172.99429321289062

},

"end_task": true

},

{

"name": "Queue for Shipment",

"uuid": "UDPM-1044",

"owner": "UDPM-67",

"managers": "UDPM-22",

"performers": "UDPM-21",

"tools": [

{

"name": "Shipping Information",

"uuid": "UDPM-1350",

"input_type": "UDPM-254",

"output_type": "UDPM-254",

"flags": 1,

"options": {

"udfs": [

"UDPM-1959",

"UDPM-2754",

"UDPM-2035",

"UDPM-1515",

"UDPM-309",

"UDPM-121"

]

}

},

{

"name": "Sample Information",

"uuid": "UDPM-1349",

"input_type": "UDPM-254",

"output_type": "UDPM-254",

"flags": 1,

"options": {

"udfs": [

"UDPM-6",

"UDPM-2195",

"UDPM-2502",

"UDPM-503",

"UDPM-2667",

"UDPM-2920",

"UDPM-2921",

"UDPM-2922",

"UDPM-1312",

"UDPM-2815",

"UDPM-2935",

"UDPM-883",

"UDPM-2923",

"UDPM-2924"

]

}

}

],

"next_states": [

{

"state_name": "Antibody Validation",

"state_uuid": "UDPM-1045",

"btn_text": "Sent for Antibody Validation",

"btn_css": "rgb(13, 73, 123)",

"btn_scale": "large"

},

{

"state_name": "Send for ChIP-Seq",

"state_uuid": "UDPM-1046",

"btn_text": "Sent for ChIP-Seq",

"btn_css": "rgb(13, 73, 123)",

"btn_scale": "large"

},

{

"state_name": "Sequencing Cancelled",

"state_uuid": "UDPM-1048",

"btn_text": "Cancelled",

"btn_css": "rgb(13, 73, 123)",

"after_code": "subj.set_value('Rationale/Reasoning',params['Rationale/Reasoning'])",

"btn_scale": "large",

"after_code_params": [

"UDPM-883"

]

}

],

"show_options": {

"top": 88,

"left": 196

}

},

{

"name": "Send for ChIP-Seq",

"uuid": "UDPM-1046",

"owner": "UDPM-67",

"managers": "UDPM-22",

"performers": "UDPM-21",

"tools": [

{

"name": "Report Information",

"uuid": "UDPM-1352",

"input_type": "UDPM-254",

"output_type": "UDPM-254",

"flags": 1,

"options": {

"udfs": [

"UDPM-1744",

"UDPM-121"

]

}

}

],

"next_states": [

{

"state_name": "Sequencing Cancelled",

"state_uuid": "UDPM-1048",

"btn_text": "Cancelled",

"btn_css": "rgb(13, 73, 123)",

"after_code": "subj.set_value('Rationale/Reasoning',params['Rationale/Reasoning'])",

"btn_scale": "large",

"after_code_params": [

"UDPM-883"

]

},

{

"state_name": "Files Returned",

"state_uuid": "UDPM-1047",

"btn_text": "Report Files Returned",

"btn_css": "rgb(13, 73, 123)",

"btn_scale": "large"

}

],

"show_options": {

"top": 419,

"left": 492

}

},

{

"name": "Sequencing Cancelled",

"uuid": "UDPM-1048",

"owner": "UDPM-67",

"managers": "UDPM-22",

"performers": "UDPM-21",

"show_options": {

"top": 277,

"left": 20

},

"end_task": true

}

],

"entry_point": "UDPM-1044"

},{

"name": "Genome Amplification Workflow",

"uuid": "UDPM-212",

"subject_type": "UDPM-258",

"state_defs": [

{

"name": "Amplification Process",

"uuid": "UDPM-1057",

"owner": "UDPM-67",

"managers": "UDPM-22",

"performers": "UDPM-54",

"tools": [

{

"name": "Quantification Information",

"uuid": "UDPM-1373",

"input_type": "UDPM-258",

"output_type": "UDPM-258",

"flags": 1,

"options": {

"udfs": [

"UDPM-2425",

"UDPM-1977",

"UDPM-1016",

"UDPM-627",

"UDPM-628",

"UDPM-452"

]

}

},

{

"name": "Amplification Information",

"uuid": "UDPM-1372",

"input_type": "UDPM-258",

"output_type": "UDPM-258",

"flags": 1,

"options": {

"udfs": [

"UDPM-6",

"UDPM-2775",

"UDPM-2776",

"UDPM-2777",

"UDPM-2794",

"UDPM-1042",

"UDPM-2425"

]

}

}

],

"next_states": [

{

"state_name": "Store DNA",

"state_uuid": "UDPM-1059",

"btn_text": "Store DNA",

"btn_css": "rgb(13, 73, 123)",

"after_code": "require_script 'lims_helper'\n\n#vial_count = params['Number of Vials to Store']\nif User.curr_user.user_groups.map(&:name).join(',').include? \"Lab Personnel - Building 50\"\n submit_to_fpro(subj, 'DNA', '7000000518', 1) do |s|\n \ts.set_value('OD 230', subj.get_value('OD 230'))\n s.set_value('OD 260', subj.get_value('OD 260'))\n s.set_value('OD 280', subj.get_value('OD 280'))\n s.set_value('Concentration (ng/ul)', subj.get_value('Concentration (ng/ul)'))\n end\nelsif User.curr_user.user_groups.map(&:name).join(',').include? \"Lab Personnel - Twinbrook\"\n submit_to_fpro(subj, 'DNA', '7000000427', 1) do |s|\n \ts.set_value('OD 230', subj.get_value('OD 230'))\n s.set_value('OD 260', subj.get_value('OD 260'))\n s.set_value('OD 280', subj.get_value('OD 280'))\n s.set_value('Concentration (ng/ul)', subj.get_value('Concentration (ng/ul)'))\n end\nend",

"btn_scale": "large",

"conditions": [

{

"name": "Genome Amplification&rarr;Concentration (ng/ul)",

"condition": "Not Empty",

"value": "",

"message": "Recording of Concentration, OD 230, OD 260, OD 280, and Volume are required to advance",

"subject_type": "UDPM-258",

"udf": "UDPM-1977"

},

{

"name": "Genome Amplification&rarr;OD 230",

"condition": "Not Empty",

"value": "",

"message": "Recording of Concentration, OD 230, OD 260, OD 280, and Volume are required to advance",

"subject_type": "UDPM-258",

"udf": "UDPM-1016"

},

{

"name": "Genome Amplification&rarr;OD 260",

"condition": "Not Empty",

"value": "",

"message": "Recording of Concentration, OD 230, OD 260, OD 280, and Volume are required to advance",

"subject_type": "UDPM-258",

"udf": "UDPM-627"

},

{

"name": "Genome Amplification&rarr;OD 280",

"condition": "Not Empty",

"value": "",

"message": "Recording of Concentration, OD 230, OD 260, OD 280, and Volume are required to advance",

"subject_type": "UDPM-258",

"udf": "UDPM-628"

},

{

"name": "Genome Amplification&rarr;Volume (uL)",

"condition": "Not Empty",

"value": "",

"message": "Recording of Concentration, OD 230, OD 260, OD 280, and Volume are required to advance",

"subject_type": "UDPM-258",

"udf": "UDPM-452"

}

]

},

{

"state_name": "Cancel",

"state_uuid": "UDPM-1058",

"btn_text": "Cancel/Discard",

"btn_css": "rgb(13, 73, 123)",

"after_code": "subj.set_value('Rationale/Reasoning',params['Rationale/Reasoning'])",

"btn_scale": "large",

"after_code_params": [

"UDPM-883"

]

}

],

"show_options": {

"top": 205,

"left": 78

}

},

{

"name": "Cancel",

"uuid": "UDPM-1058",

"owner": "UDPM-67",

"managers": "UDPM-22",

"performers": "UDPM-54",

"show_options": {

"top": 71,

"left": 367

},

"end_task": true

},

{

"name": "Store DNA",

"uuid": "UDPM-1059",

"owner": "UDPM-67",

"managers": "UDPM-22",

"performers": "UDPM-54",

"show_options": {

"top": 245,

"left": 444

},

"end_task": true

}

],

"entry_point": "UDPM-1057",

"show_udfs": "UDPM-6,UDPM-2775,UDPM-2776"

},{

"name": "Photography Release/Consent",

"uuid": "UDPM-197",

"subject_type": "UDPM-242",

"state_defs": [

{

"name": "Photography Release",

"uuid": "UDPM-979",

"owner": "UDPM-67",

"managers": "UDPM-49",

"performers": "UDPM-2",

"tools": [

{

"name": "Edit Consent Information",

"uuid": "UDPM-1545",

"input_type": "UDPM-242",

"output_type": "UDPM-242",

"flags": 1,

"options": {

"udfs": [

"UDPM-6",

"UDPM-2615",

"UDPM-2616"

]

}

}

],

"show_options": {

"top": 180,

"left": 400

},

"end_task": true

}

],

"entry_point": "UDPM-979",

"show_udfs": "UDPM-2615,UDPM-2616,UDPM-6"

},{

"name": "Cohort Research Request Workflow",

"uuid": "UDPM-220",

"subject_type": "UDPM-271",

"state_defs": [

{

"name": "All Requests Assigned",

"uuid": "UDPM-1083",

"owner": "UDPM-67",

"managers": "UDPM-22",

"performers": "UDPM-54",

"show_options": {

"top": 325,

"left": 73

},

"end_task": true

},

{

"name": "Request Cancelled",

"uuid": "UDPM-1092",

"owner": "UDPM-67",

"managers": "UDPM-54",

"performers": "UDPM-49",

"show_options": {

"top": 66,

"left": 566

},

"end_task": true

},

{

"name": "Request Created",

"uuid": "UDPM-1082",

"owner": "UDPM-67",

"managers": "UDPM-22",

"performers": "UDPM-54",

"tools": [

{

"name": "Begin Collaboration",

"uuid": "UDPM-1495",

"input_type": "UDPM-271",

"output_type": "UDPM-147",

"flags": 0,

"options": {

"obj_type": "SubjectType"

}

},

{

"name": "Perform Assay",

"uuid": "UDPM-1494",

"input_type": "UDPM-271",

"output_type": "UDPM-225",

"flags": 0,

"options": {

"obj_type": "SubjectType"

}

}

],

"next_states": [

{

"state_name": "All Requests Assigned",

"state_uuid": "UDPM-1083",

"btn_text": "All Requests Assigned",

"btn_css": "rgb(13, 73, 123)",

"btn_scale": "large"

},

{

"state_name": "Request Returned",

"state_uuid": "UDPM-1091",

"btn_text": "Return Request to Sender",

"btn_css": "rgb(13, 73, 123)",

"after_code": "subj.set_value('Comments/Special Instructions', params['Comments/Special Instructions'])\nself.next_state_performer = subj.created_by",

"btn_scale": "large",

"after_code_params": [

"UDPM-1344"

]

}

],

"show_options": {

"top": 58,

"left": 79

}

},

{

"name": "Request Returned",

"uuid": "UDPM-1091",

"owner": "UDPM-67",

"managers": "UDPM-54",

"performers": "UDPM-49",

"duration": 2.0,

"next_states": [

{

"state_name": "Request Cancelled",

"state_uuid": "UDPM-1092",

"btn_text": "Cancel Request",

"btn_css": "rgb(13, 73, 123)",

"btn_scale": "large"

},

{

"state_name": "Request Created",

"state_uuid": "UDPM-1082",

"btn_text": "Resend Request",

"btn_css": "rgb(13, 73, 123)",

"before_code": "#f = subj.get_value(\"Comments\")\n#r = subj.get_value(\"Reverse Primer\")\n\n#params[:defaults] = {\n# 'Requester'=> User.curr_user,\n# 'Primer ordering'=> table,\n #'Primer Design File'=> x\n#}",

"btn_scale": "large"

}

],

"em_performer_new_job": true,

"show_options": {

"top": 345,

"left": 369

}

}

],

"entry_point": "UDPM-1082",

"show_udfs": "UDPM-6,UDPM-2810,UDPM-1301,UDPM-2856,UDPM-2857,UDPM-2894"

},{

"name": "Vials",

"uuid": "UDPM-194",

"subject_type": "UDPM-152",

"state_defs": [

{

"name": "Vial Stored",

"uuid": "UDPM-959",

"owner": "UDPM-67",

"managers": "UDPM-22",

"performers": "UDPM-4",

"tools": [

{

"name": "Begin DNA Amplification",

"uuid": "UDPM-1513",

"input_type": "UDPM-152",

"output_type": "UDPM-258",

"flags": 0,

"hide_button_if_not_condition": true,

"conditions": [

{

"name": "Vial&rarr;Sample Type",

"condition": "=",

"value": "DNA",

"message": "",

"subject_type": "UDPM-152",

"udf": "UDPM-415"

}

],

"options": {

"obj_type": "SubjectType"

}

},

{

"name": "Print Barcode",

"uuid": "UDPM-1211",

"input_type": "UDPM-152",

"output_type": "UDPM-152",

"flags": 2,

"options": {

"obj_type": "Printer",

"labels_count": 1,

"obj_name": "UDPM-16"

}

}

],

"show_options": {

"top": 106.99147033691406,

"left": 191.98861694335938

},

"end_task": true

}

],

"entry_point": "UDPM-959"

},{

"name": "Entry Vector Workflow",

"uuid": "UDPM-163",

"subject_type": "UDPM-193",

"state_defs": [

{

"name": "Amplification of cDNA",

"uuid": "UDPM-761",

"owner": "UDPM-67",

"managers": "UDPM-22",

"performers": "UDPM-21",

"tools": [

{

"name": "Request Primer Order",

"uuid": "UDPM-1135",

"input_type": "UDPM-193",

"output_type": "UDPM-217",

"flags": 0,

"options": {

"obj_type": "SubjectType"

}

},

{

"name": "Amplification Information",

"uuid": "UDPM-946",

"input_type": "UDPM-193",

"output_type": "UDPM-193",

"flags": 1,

"options": {

"udfs": [

"UDPM-1957",

"UDPM-1926",

"UDPM-2419",

"UDPM-455",

"UDPM-1693"

]

}

}

],

"next_states": [

{

"state_name": "Transformation",

"state_uuid": "UDPM-762",

"btn_text": "Begin Transformation",

"btn_css": "#456",

"after_code": "self.next_state_performer=User.curr_user",

"btn_scale": "large"

},

{

"state_name": "Cancelled",

"state_uuid": "UDPM-970",

"btn_text": "Cancel",

"btn_css": "#456",

"after_code": "subj.set_value('Rationale/Reasoning', params['Rationale/Reasoning'])",

"btn_scale": "large",

"after_code_params": [

"UDPM-883"

]

}

],

"show_options": {

"top": 84,

"left": 96

}

},

{

"name": "Cancelled",

"uuid": "UDPM-970",

"owner": "UDPM-67",

"managers": "UDPM-22",

"performers": "UDPM-21",

"show_options": {

"top": 305,

"left": 333

},

"end_task": true

},

{

"name": "Store MaxiPrep",

"uuid": "UDPM-765",

"owner": "UDPM-67",

"managers": "UDPM-22",

"performers": "UDPM-21",

"tools": [

{

"name": "Store MaxiPrep",

"uuid": "UDPM-1493",

"input_type": "UDPM-193",

"output_type": "UDPM-269",

"flags": 0,

"options": {

"obj_type": "SubjectType"

}

},

{

"name": "Prepare Destination Vector",

"uuid": "UDPM-943",

"input_type": "UDPM-193",

"output_type": "UDPM-168",

"flags": 0,

"before_code": "gene = subj.get_value(\"Gene\")\ndes = subj.get_value(\"Description\")\n\nparams[:defaults] = {\n 'Gene' => gene,\n 'Description' => des\n}",

"options": {

"obj_type": "SubjectType"

}

},

{

"name": "Prepare Glycerol Stock",

"uuid": "UDPM-952",

"input_type": "UDPM-193",

"output_type": "UDPM-171",

"flags": 0,

"before_code": "gene = subj.get_value(\"Gene\")\ndes = subj.get_value(\"Description\")\n\nparams[:defaults] = {\n 'Genes' => gene,\n 'Description' => des\n}",

"options": {

"obj_type": "SubjectType"

}

},

{

"name": "Mutagenize Vector",

"uuid": "UDPM-1205",

"input_type": "UDPM-193",

"output_type": "UDPM-228",

"flags": 0,

"options": {

"obj_type": "SubjectType"

}

},

{

"name": "Store MiniPrep",

"uuid": "UDPM-1450",

"input_type": "UDPM-193",

"output_type": "UDPM-249",

"flags": 0,

"options": {

"obj_type": "SubjectType"

}

}

],

"show_options": {

"top": 293.9943084716797,

"left": 756.9971008300781

},

"end_task": true

},

{

"name": "Transformation",

"uuid": "UDPM-762",

"owner": "UDPM-67",

"managers": "UDPM-22",

"performers": "UDPM-21",

"tools": [

{

"name": "Prepare Glycerol Stock",

"uuid": "UDPM-928",

"input_type": "UDPM-193",

"output_type": "UDPM-171",

"flags": 0,

"options": {

"obj_type": "SubjectType"

}

},

{

"name": "Entry Vector Information",

"uuid": "UDPM-929",

"input_type": "UDPM-193",

"output_type": "UDPM-193",

"flags": 1,

"options": {

"udfs": [

"UDPM-1840",

"UDPM-1799",

"UDPM-1777",

"UDPM-1769",

"UDPM-1923",

"UDPM-1927",

"UDPM-1962",

"UDPM-5"

]

}

},

{

"name": "MaxiPrep Information",

"uuid": "UDPM-945",

"input_type": "UDPM-193",

"output_type": "UDPM-193",

"flags": 1,

"options": {

"udfs": [

"UDPM-1977",

"UDPM-1332",

"UDPM-1016",

"UDPM-627",

"UDPM-628"

]

}

},

{

"name": "Request Primer Order",

"uuid": "UDPM-1136",

"input_type": "UDPM-193",

"output_type": "UDPM-217",

"flags": 0,

"options": {

"obj_type": "SubjectType"

}

},

{

"name": "Mutagenize Vector",

"uuid": "UDPM-1201",

"input_type": "UDPM-193",

"output_type": "UDPM-228",

"flags": 0,

"options": {

"obj_type": "SubjectType"

}

},

{

"name": "Prepare MiniPrep",

"uuid": "UDPM-1275",

"input_type": "UDPM-193",

"output_type": "UDPM-249",

"flags": 0,

"options": {

"obj_type": "SubjectType"

}

},

{

"name": "Upload AB1 File",

"uuid": "UDPM-1449",

"input_type": "UDPM-193",

"output_type": "UDPM-250",

"flags": 0,

"options": {

"obj_type": "SubjectType",

"do_not_open_subject": true

}

}

],

"next_states": [

{

"state_name": "Store MaxiPrep",

"state_uuid": "UDPM-765",

"btn_text": "Store MaxiPrep",

"btn_css": "#456",

"after_code": "require_script 'fpro_samples'\n\nvial_count = params['Number of Vials to Store']\nevb = subj.get_value('Entry Vector Backbone')\nif User.curr_user.user_groups.map(&:name).join(',').include? \"Lab Personnel - Twinbrook\"\n submit_to_fpro(subj, 'MaxiPrep DNA', '7000000404', vial_count) do |s|\n s.set_value('Vector Information', evb.name)\n s.set_value('Insert Information', subj.get_value('Insert Name'))\n s.set_value('Resistance Marker', evb.get_value('Resistance Marker'))\n end\nelsif User.curr_user.user_groups.map(&:name).join(',').include? \"Lab Personnel - Building 50\"\n submit_to_fpro(subj, 'MaxiPrep DNA', '7000000476', vial_count) do |s|\n s.set_value('Vector Information', evb.name)\n s.set_value('Insert Information', subj.get_value('Insert Name'))\n s.set_value('Resistance Marker', evb.get_value('Resistance Marker'))\n end\nelse\n raise(\"This script only allows users who belong to either Twinbrook Lab or Building 50 Lab, please contact the administrators\")\nend",

"btn_scale": "large",

"after_code_params": [

"UDPM-1450"

]

},

{

"state_name": "Cancelled",

"state_uuid": "UDPM-970",

"btn_text": "Cancel",

"btn_css": "#456",

"after_code": "subj.set_value('Rationale/Reasoning', params['Rationale/Reasoning'])",

"btn_scale": "large",

"after_code_params": [

"UDPM-883"

]

}

],

"show_options": {

"top": 160,

"left": 473

}

}

],

"entry_point": "UDPM-761",

"ask_performer": true,

"show_udfs": "UDPM-6,UDPM-2419,UDPM-1693,UDPM-455,UDPM-1957,UDPM-1656,UDPM-1926"

},{

"name": "Sanger Sequencing Workflow",

"uuid": "UDPM-186",

"subject_type": "UDPM-221",

"state_defs": [

{

"name": "Design Primers",

"uuid": "UDPM-900",

"owner": "UDPM-67",

"managers": "UDPM-22",

"performers": "UDPM-21",

"tools": [

{

"name": "2) Order Primers",

"uuid": "UDPM-1152",

"input_type": "UDPM-221",

"output_type": "UDPM-217",

"flags": 0,

"before_code": "# Script to run Before Tool is executed\nf = subj.get_value(\"Forward Primer\")\nr = subj.get_value(\"Reverse Primer\")\n#v = subj.get_value(\"Primers\")\n#x = subj.get_value(\"Primer 3 Output File\")\n#a = v.split('\\n')\ntable = \"<html>\n<table>\n<tr><td>Primer Name</td><td>Sequence</td><td>Amount</td></tr>\n<tr><td></td><td>#{f}</td><td></td></tr>\n<tr><td></td><td>#{r}</td><td></td></tr>\n</table>\n</html>\"\nparams[:defaults] = {\n 'Requester'=> User.curr_user,\n 'Primer ordering'=> table,\n #'Primer Design File'=> x\n}\n",

"after_code": "# Script to run After Tool is executed\nsubj.set_value('Primers Requested', 'Yes')\n\nx = subj.get_value(\"Primer 3 Output File\")\nparams[:new_subj].set_value('Primer Design File', x)\n",

"conditions": [

{

"name": "Sanger Sequencing Information&rarr;Primers Requested",

"condition": "Empty",

"value": "",

"message": "",

"subject_type": "UDPM-221",

"udf": "UDPM-2595"

}

],

"options": {

"obj_type": "SubjectType"

}

},

{

"name": "1) Primer Information",

"uuid": "UDPM-1151",

"input_type": "UDPM-221",

"output_type": "UDPM-221",

"flags": 1,

"conditions": [

{

"name": "Sanger Sequencing Information&rarr;Primer 3 Output File",

"condition": "Empty",

"value": "",

"message": "",

"subject_type": "UDPM-221",

"udf": "UDPM-891"

}

],

"options": {

"udfs": [

"UDPM-889",

"UDPM-2593",

"UDPM-2594",

"UDPM-891",

"UDPM-191"

]

}

}

],

"next_states": [

{

"state_name": "Primers Designed",

"state_uuid": "UDPM-921",

"btn_text": "3) Primers Received",

"btn_css": "#456"

}

],

"show_options": {

"top": 226,

"left": 393

}

},

{

"name": "Primers Designed",

"uuid": "UDPM-921",

"owner": "UDPM-67",

"managers": "UDPM-22",

"performers": "UDPM-21",

"tools": [

{

"name": "Begin Amplification",

"uuid": "UDPM-1164",

"input_type": "UDPM-221",

"output_type": "UDPM-22",

"flags": 0,

"before_code": "# Script to run Before Tool is executed\nb = subj.get_value(\"Build\")\nf = subj.get_value(\"Forward Primer\")\nr = subj.get_value(\"Reverse Primer\")\n\nparams[:defaults] = {\n 'Build'=> b,\n 'Forward Primer' => f,\n 'Reverse Primer' => r\n}",

"options": {

"obj_type": "SubjectType"

}

}

],

"show_options": {

"top": 372,

"left": 543

},

"end_task": true

},

{

"name": "Variant Information",

"uuid": "UDPM-899",

"owner": "UDPM-67",

"managers": "UDPM-22",

"performers": "UDPM-32",

"duration": 1.0,

"tools": [

{

"name": "Variant Information",

"uuid": "UDPM-1150",

"input_type": "UDPM-221",

"output_type": "UDPM-221",

"flags": 1,

"options": {

"udfs": [

"UDPM-2195",

"UDPM-2258",

"UDPM-1277",

"UDPM-886",

"UDPM-304",

"UDPM-191"

]

}

}

],

"next_states": [

{

"state_name": "Design Primers",

"state_uuid": "UDPM-900",

"btn_text": "Design Primers",

"btn_css": "#456",

"before_code": "params[:ask_next_performer] = true"

}

],

"show_options": {

"top": 60,

"left": 152

}

}

],

"entry_point": "UDPM-899",

"ask_performer": true,

"show_udfs": "UDPM-2195,UDPM-2258,UDPM-304,UDPM-191,UDPM-886,UDPM-1277"

},{

"name": "Collaboration Projects Workflow",

"uuid": "UDPM-175",

"subject_type": "UDPM-204",

"state_defs": [

{

"name": "Collaboration Project Complete",

"uuid": "UDPM-859",

"owner": "UDPM-67",

"managers": "UDPM-32",

"performers": "UDPM-32",

"tools": [

{

"name": "Request Collaboration",

"uuid": "UDPM-1516",

"input_type": "UDPM-204",

"output_type": "UDPM-147",

"flags": 0,

"options": {

"obj_type": "SubjectType"

}

},

{

"name": "Upload MTA/ITA Amendment",

"uuid": "UDPM-1300",

"input_type": "UDPM-204",

"output_type": "UDPM-251",

"flags": 0,

"options": {

"obj_type": "SubjectType"

}

}

],

"show_options": {

"top": 481,

"left": 450.88330078125

},

"end_task": true

},

{

"name": "MTA/ITA Approved by IRB",

"uuid": "UDPM-998",

"owner": "UDPM-67",

"managers": "UDPM-45",

"performers": "UDPM-45",

"tools": [

{

"name": "Add New Collaborating Center",

"uuid": "UDPM-1286",

"input_type": "UDPM-204",

"output_type": "UDPM-166",

"description": "Institution or lab",

"flags": 0,

"options": {

"obj_type": "SubjectType"

}

},

{

"name": "Add New Collaborator",

"uuid": "UDPM-1285",

"input_type": "UDPM-204",

"output_type": "UDPM-149",

"flags": 0,

"options": {

"obj_type": "SubjectType"

}

}

],

"next_states": [

{

"state_name": "Collaboration Project Complete",

"state_uuid": "UDPM-859",

"btn_text": "MTA/ITA Executed",

"btn_css": "#456",

"after_code": "send_email(subj.created_by, find_email_template(\"MTA_ITA executed notification\"), subj)"

}

],

"em_performer_new_job": true,

"show_options": {

"top": 339,

"left": 464.33331298828125

}

},

{

"name": "MTA Request Cancelled",

"uuid": "UDPM-1081",

"owner": "UDPM-67",

"managers": "UDPM-45",

"performers": "UDPM-45",

"tools": [

{

"name": "Reasoning for Cancellation",

"uuid": "UDPM-1453",

"input_type": "UDPM-204",

"output_type": "UDPM-204",

"flags": 1,

"options": {

"udfs": [

"UDPM-883"

]

}

}

],

"show_options": {

"top": 189,

"left": 708

},

"end_task": true

},

{

"name": "Request MTA/ITA",

"uuid": "UDPM-857",

"owner": "UDPM-67",

"managers": "UDPM-45",

"performers": "UDPM-45",

"tools": [

{

"name": "MTA/ITA Information",

"uuid": "UDPM-1089",

"input_type": "UDPM-204",

"output_type": "UDPM-204",

"flags": 1,

"options": {

"udfs": [

"UDPM-1417",

"UDPM-2702",

"UDPM-2704",

"UDPM-2703",

"UDPM-1699",

"UDPM-2136",

"UDPM-2032",

"UDPM-1399",

"UDPM-2130",

"UDPM-2113",

"UDPM-2700",

"UDPM-2701"

]

}

}

],

"next_states": [

{

"state_name": "Request Sent to IRB for Approval",

"state_uuid": "UDPM-973",

"btn_text": "Submit to IRB for Approval",

"btn_css": "#456",

"after_code": "self.next_state_performer = find_user('[redacted]')\n#send_email(User.find_by_username('[redacted]'), find_email_template(\"UDP MTA/ITA Request\"), subj)\n\nrecipients = []\nrecipients << find_user_group('Consent Approval')\nsend_email(recipients, find_email_template(\"UDP MTA/ITA Request\"), subj)\n",

"btn_scale": "large"

}

],

"em_performer_new_job": true,

"show_options": {

"top": 41,

"left": 103

}

},

{

"name": "Request Sent to IRB for Approval",

"uuid": "UDPM-973",

"owner": "UDPM-67",

"managers": "UDPM-45",

"performers": "UDPM-65",

"tools": [

{

"name": "Patients without Proper Consents",

"uuid": "UDPM-1258",

"input_type": "UDPM-204",

"output_type": "UDPM-204",

"flags": 1,

"options": {

"udfs": [

"UDPM-2651",

"UDPM-2059"

]

}

}

],

"next_states": [

{

"state_name": "Upload MTA/ITA",

"state_uuid": "UDPM-1027",

"btn_text": "All Consents Approved",

"btn_css": "#456",

"after_code": "next_state_performer = User.find_by_username('[redacted]')",

"btn_scale": "large"

},

{

"state_name": "Reviewed by IRB - Revisions Required",

"state_uuid": "UDPM-997",

"btn_text": "Revisions Required",

"btn_css": "#456",

"after_code": "self.next_state_performer = find_user('[redacted]')\n#send_email(User.find_by_username('[redacted]'), find_email_template(\"UDP MTA/ITA Request\"), subj)\n\nrecipients = []\nrecipients << find_user_group('MTA/ITA Managers')\nsend_email(recipients, find_email_template(\"MTA/ITA Patient Consents Not Approved by IRB\"), subj)",

"btn_scale": "large"

}

],

"show_options": {

"top": 38,

"left": 437.4166259765625

}

},

{

"name": "Reviewed by IRB - Revisions Required",

"uuid": "UDPM-997",

"owner": "UDPM-67",

"managers": "UDPM-45",

"performers": "UDPM-45",

"tools": [

{

"name": "Edit Patients for MTA/ITA",

"uuid": "UDPM-1259",

"input_type": "UDPM-204",

"output_type": "UDPM-204",

"flags": 1,

"options": {

"udfs": [

"UDPM-2032"

]

}

}

],

"next_states": [

{

"state_name": "Request Sent to IRB for Approval",

"state_uuid": "UDPM-973",

"btn_text": "Submit Revisions to IRB",

"btn_css": "#456",

"after_code": "self.next_state_performer = find_user('[redacted]')\n#send_email(User.find_by_username('[redacted]'), find_email_template(\"UDP MTA/ITA Request\"), subj)\n\nrecipients = []\nrecipients << find_user_group('Consent Approval')\nsend_email(recipients, find_email_template(\"UDP MTA/ITA Request Re-submit\"), subj)",

"btn_scale": "large"

}

],

"show_options": {

"top": 42,

"left": 824.6500244140625

}

},

{

"name": "Upload MTA/ITA",

"uuid": "UDPM-1027",

"owner": "UDPM-67",

"managers": "UDPM-45",

"performers": "UDPM-65",

"tools": [

{

"name": "Upload MTA/ITA",

"uuid": "UDPM-1298",

"input_type": "UDPM-204",

"output_type": "UDPM-204",

"flags": 1,

"options": {

"udfs": [

"UDPM-2129",

"UDPM-2128",

"UDPM-2059"

]

}

}

],

"next_states": [

{

"state_name": "MTA/ITA Approved by IRB",

"state_uuid": "UDPM-998",

"btn_text": "MTA/ITA Complete",

"btn_css": "#456",

"after_code": "self.next_state_performer = find_user('[redacted]')",

"btn_scale": "large"

}

],

"show_options": {

"top": 194,

"left": 490

}

}

],

"entry_point": "UDPM-857",

"show_udfs": "UDPM-2701,UDPM-2703,UDPM-2704,UDPM-2032,UDPM-2130,UDPM-1399"

},{

"name": "Patient Follow Up Visit",

"uuid": "UDPM-213",

"subject_type": "UDPM-264",

"state_defs": [

{

"name": "Admitted",

"uuid": "UDPM-1063",

"owner": "UDPM-67",

"managers": "UDPM-49",

"performers": "UDPM-49",

"tools": [

{

"name": "Add Diagnosis",

"uuid": "UDPM-1378",

"input_type": "UDPM-264",

"output_type": "UDPM-185",

"flags": 0,

"options": {

"obj_type": "SubjectType"

}

},

{

"name": "Date of Admission",

"uuid": "UDPM-1379",

"input_type": "UDPM-264",

"output_type": "UDPM-264",

"flags": 1,

"options": {

"udfs": [

"UDPM-2733"

]

}

},

{

"name": "Letter Request",

"uuid": "UDPM-1380",

"input_type": "UDPM-264",

"output_type": "UDPM-253",

"flags": 0,

"options": {

"obj_type": "SubjectType"

}

},

{

"name": "PhenoTips Review",

"uuid": "UDPM-1381",

"input_type": "UDPM-264",

"output_type": "UDPM-205",

"flags": 0,

"options": {

"obj_type": "SubjectType"

}

},

{

"name": "Radiology Review",

"uuid": "UDPM-1382",

"input_type": "UDPM-264",

"output_type": "UDPM-246",

"flags": 0,

"options": {

"obj_type": "SubjectType"

}

},

{

"name": "Change State",

"uuid": "UDPM-1399",

"input_type": "UDPM-264",

"output_type": "UDPM-264",

"flags": 6,

"after_code": "# Script to run After Tool is executed\nnext_state = params[\"Follow_up_patient_workflow_states\"]\nadvance_workflow(\"Patient Follow Up Visit\",next_state,subj)\nshow_message(\"Patient is now successfully placed at #{next_state} state\")\n",

"after_code_params": [

"UDPM-2789"

]

}

],

"next_states": [

{

"state_name": "End of Visit Checklist",

"state_uuid": "UDPM-1064",

"btn_text": "Visit Checklist",

"btn_css": "rgb(35, 31, 32)",

"btn_scale": "large"

}

],

"show_options": {

"top": 224,

"left": 84

}

},

{

"name": "End of Visit Checklist",

"uuid": "UDPM-1064",

"owner": "UDPM-67",

"managers": "UDPM-49",

"performers": "UDPM-49",

"tools": [

{

"name": "Add Diagnosis",

"uuid": "UDPM-1384",

"input_type": "UDPM-264",

"output_type": "UDPM-185",

"flags": 0,

"options": {

"obj_type": "SubjectType"

}

},

{

"name": "Checklist",

"uuid": "UDPM-1385",

"input_type": "UDPM-264",

"output_type": "UDPM-264",

"flags": 1,

"options": {

"udfs": [

"UDPM-2734"

]

}

},

{

"name": "Letter Request",

"uuid": "UDPM-1386",

"input_type": "UDPM-264",

"output_type": "UDPM-253",

"flags": 0,

"options": {

"obj_type": "SubjectType"

}

},

{

"name": "PhenoTips Review",

"uuid": "UDPM-1387",

"input_type": "UDPM-264",

"output_type": "UDPM-205",

"flags": 0,

"options": {

"obj_type": "SubjectType"

}

},

{

"name": "Radiology Review",

"uuid": "UDPM-1388",

"input_type": "UDPM-264",

"output_type": "UDPM-246",

"flags": 0,

"options": {

"obj_type": "SubjectType"

}

},

{

"name": "Change State",

"uuid": "UDPM-1400",

"input_type": "UDPM-264",

"output_type": "UDPM-264",

"flags": 6,

"after_code": "# Script to run After Tool is executed\nnext_state = params[\"Follow_up_patient_workflow_states\"]\nadvance_workflow(\"Patient Follow Up Visit\",next_state,subj)\nshow_message(\"Patient is now successfully placed at #{next_state} state\")\n",

"after_code_params": [

"UDPM-2789"

]

}

],

"next_states": [

{

"state_name": "Seen",

"state_uuid": "UDPM-1065",

"btn_text": "Seen",

"btn_css": "rgb(35, 31, 32)",

"btn_scale": "large"

}

],

"show_options": {

"top": 128,

"left": 395

}

},

{

"name": "Plan Visit",

"uuid": "UDPM-1066",

"owner": "UDPM-67",

"managers": "UDPM-49",

"performers": "UDPM-49",

"tools": [

{

"name": "Add Diagnosis",

"uuid": "UDPM-1392",

"input_type": "UDPM-264",

"output_type": "UDPM-185",

"flags": 0,

"options": {

"obj_type": "SubjectType"

}

},

{

"name": "Letter Request",

"uuid": "UDPM-1393",

"input_type": "UDPM-264",

"output_type": "UDPM-253",

"flags": 0,

"options": {

"obj_type": "SubjectType"

}

},

{

"name": "PhenoTips Review",

"uuid": "UDPM-1394",

"input_type": "UDPM-264",

"output_type": "UDPM-205",

"flags": 0,

"options": {

"obj_type": "SubjectType"

}

},

{

"name": "Request Review",

"uuid": "UDPM-1395",

"input_type": "UDPM-264",

"output_type": "UDPM-83",

"flags": 0,

"options": {

"obj_type": "SubjectType"

}

},

{

"name": "Change State",

"uuid": "UDPM-1398",

"input_type": "UDPM-264",

"output_type": "UDPM-264",

"flags": 6,

"after_code": "# Script to run After Tool is executed\nnext_state = params[\"Follow_up_patient_workflow_states\"]\nadvance_workflow(\"Patient Follow Up Visit\",next_state,subj)\nshow_message(\"Patient is now successfully placed at #{next_state} state\")\n",

"after_code_params": [

"UDPM-2789"

]

}

],

"next_states": [

{

"state_name": "Admitted",

"state_uuid": "UDPM-1063",

"btn_text": "Admit",

"btn_css": "rgb(35, 31, 32)",

"btn_scale": "large"

}

],

"show_options": {

"top": 33,

"left": 42

}

},

{

"name": "Seen",

"uuid": "UDPM-1065",

"owner": "UDPM-67",

"managers": "UDPM-49",

"performers": "UDPM-49",

"tools": [

{

"name": "Add Diagnosis",

"uuid": "UDPM-1389",

"input_type": "UDPM-264",

"output_type": "UDPM-185",

"flags": 0,

"options": {

"obj_type": "SubjectType"

}

},

{

"name": "Date Seen",

"uuid": "UDPM-1390",

"input_type": "UDPM-264",

"output_type": "UDPM-264",

"flags": 1,

"options": {

"udfs": [

"UDPM-545"

]

}

},

{

"name": "Change State",

"uuid": "UDPM-1401",

"input_type": "UDPM-264",

"output_type": "UDPM-264",

"flags": 6,

"after_code": "# Script to run After Tool is executed\nnext_state = params[\"Follow_up_patient_workflow_states\"]\nadvance_workflow(\"Patient Follow Up Visit\",next_state,subj)\nshow_message(\"Patient is now successfully placed at #{next_state} state\")\n",

"after_code_params": [

"UDPM-2789"

]

}

],

"show_options": {

"top": 239,

"left": 725

},

"end_task": true

}

],

"entry_point": "UDPM-1066",

"ask_performer": true

},{

"name": "dbGaP Submission Workflow",

"uuid": "UDPM-174",

"subject_type": "UDPM-213",

"state_defs": [

{

"name": "Cancelled Submission",

"uuid": "UDPM-870",

"owner": "UDPM-67",

"managers": "UDPM-8",

"performers": "UDPM-59",

"show_options": {

"top": 220,

"left": 105

},

"end_task": true

},

{

"name": "Data for Submission",

"uuid": "UDPM-850",

"owner": "UDPM-67",

"managers": "UDPM-8",

"performers": "UDPM-59",

"tools": [

{

"name": "dbGaP Submission",

"uuid": "UDPM-1084",

"input_type": "UDPM-213",

"output_type": "UDPM-213",

"flags": 1,

"options": {

"udfs": [

"UDPM-6",

"UDPM-2286",

"UDPM-2287",

"UDPM-5"

]

}

}

],

"next_states": [

{

"state_name": "Cancelled Submission",

"state_uuid": "UDPM-870",

"btn_text": "Cancel Submission",

"btn_css": "#456"

},

{

"state_name": "Ready for Submission",

"state_uuid": "UDPM-851",

"btn_text": "Ready for Submission",

"btn_css": "#456",

"after_code": "patient = subj.get_value('Patient')\nproband = patient.get_value('Proband')\n#phenotips = patient.get_value('PhenoTips Complete')\nif proband == 'Yes' and not phenotips \n raise \"Proband PhenoTips must be complete to submit.\"\nend",

"conditions": [

{

"name": "Patient&rarr;Consents",

"condition": "Not Empty",

"value": "",

"message": "Cannot submit to dbGaP wihtout documented Consent Form, Race, and Ethnicity.",

"subject_type": "UDPM-1",

"udf": "UDPM-1390"

},

{

"name": "Patient&rarr;Race",

"condition": "Not Empty",

"value": "",

"message": "Cannot submit to dbGaP wihtout documented Consent Form, Race, and Ethnicity.",

"subject_type": "UDPM-1",

"udf": "UDPM-2202"

},

{

"name": "Patient&rarr;Ethnicity",

"condition": "Not Empty",

"value": "",

"message": "Cannot submit to dbGaP wihtout documented Consent Form, Race, and Ethnicity.",

"subject_type": "UDPM-1",

"udf": "UDPM-562"

}

]

}

],

"em_performer_new_job": true,

"em_owner_done_job": true,

"show_options": {

"top": 53,

"left": 147

}

},

{

"name": "Editing for Resubmission",

"uuid": "UDPM-871",

"owner": "UDPM-67",

"managers": "UDPM-8",

"performers": "UDPM-59",

"next_states": [

{

"state_name": "Final Submission Accepted to dbGaP",

"state_uuid": "UDPM-853",

"btn_text": "Passed dbGaP Quality Control",

"btn_css": "#456",

"after_code": "self.next_state_performer = User.curr_user\n\npatient = subj.get_value('Patient')\n if patient\n recipients = []\n physician = patient.get_value('Attending Physician')\n clinician = patient.get_value('Primary Clinician')\n recipients << physician if physician\n recipients << clinician if clinician\n end\n\nif recipients.any?\n send_email(recipients, find_email_template(\"dbGaP Data Submitted\"), subj) \nend"

},

{

"state_name": "Cancelled Submission",

"state_uuid": "UDPM-870",

"btn_text": "Cancel Submission",

"btn_css": "#456"

}

],

"show_options": {

"top": 386,

"left": 424

}

},

{

"name": "Final Submission Accepted to dbGaP",

"uuid": "UDPM-853",

"owner": "UDPM-67",

"managers": "UDPM-8",

"performers": "UDPM-59",

"show_options": {

"top": 370,

"left": 870.1500244140625

},

"end_task": true

},

{

"name": "Ready for Submission",

"uuid": "UDPM-851",

"owner": "UDPM-67",

"managers": "UDPM-59",

"performers": "UDPM-59",

"next_states": [

{

"state_name": "Cancelled Submission",

"state_uuid": "UDPM-870",

"btn_text": "Cancel Submission",

"btn_css": "#456"

},

{

"state_name": "Submitted",

"state_uuid": "UDPM-852",

"btn_text": "Submit",

"btn_css": "#456",

"after_code": "subj.set_value('Date Submitted to dbGaP', params['Date Submitted to dbGaP'])\nself.next_state_performer = User.curr_user",

"after_code_params": [

"UDPM-1099"

]

}

],

"show_options": {

"top": 156,

"left": 452

}

},

{

"name": "Submitted",

"uuid": "UDPM-852",

"owner": "UDPM-67",

"managers": "UDPM-8",

"performers": "UDPM-59",

"duration": 1.0,

"next_states": [

{

"state_name": "Editing for Resubmission",

"state_uuid": "UDPM-871",

"btn_text": "Failed dbGaP Quality Control",

"btn_css": "#456",

"after_code": "self.next_state_performer = User.curr_user"

},

{

"state_name": "Final Submission Accepted to dbGaP",

"state_uuid": "UDPM-853",

"btn_text": "Passed dbGaP Quality Control",

"btn_css": "#456",

"after_code": "#self.next_state_performer = User.curr_user\n\n#patient = subj.get_value('Patient')\n# if patient\n# recipients = []\n# physician = patient.get_value('Attending Physician')\n# clinician = patient.get_value('Primary Clinician')\n# recipients << physician if physician\n# recipients << clinician if clinician\n# end\n\n#if recipients.any?\n# send_email(recipients, find_email_template(\"dbGaP Data Submitted\"), subj) \n#end",

"btn_scale": "large"

}

],

"em_owner_pastdue_job": true,

"show_options": {

"top": 239,

"left": 692.9833374023438

}

},

{

"name": "Waiting for Phenotips to be Completed",

"uuid": "UDPM-960",

"owner": "UDPM-3",

"managers": "UDPM-8",

"performers": "UDPM-59",

"show_options": {

"top": 800,

"left": 988

}

}

],

"entry_point": "UDPM-850",

"show_udfs": "UDPM-2286,UDPM-2287,UDPM-5"

},{

"name": "Cohort VCF Pipeline",

"uuid": "UDPM-164",

"subject_type": "UDPM-195",

"state_defs": [

{

"name": "Annotation of Cohort VCF",

"uuid": "UDPM-790",

"owner": "UDPM-67",

"managers": "UDPM-27",

"performers": "UDPM-3",

"tools": [

{

"name": "Annotated VCF Information",

"uuid": "UDPM-990",

"input_type": "UDPM-195",

"output_type": "UDPM-195",

"flags": 1,

"options": {

"udfs": [

"UDPM-2050",

"UDPM-2047",

"UDPM-2052",

"UDPM-995",

"UDPM-994",

"UDPM-385",

"UDPM-377",

"UDPM-1894",

"UDPM-2048"

]

}

}

],

"next_states": [

{

"state_name": "Completion of Annotated Cohort VCF",

"state_uuid": "UDPM-791",

"btn_text": "Complete Annotation",

"btn_css": "#456"

}

],

"show_options": {

"top": 141,

"left": 489

}

},

{

"name": "Completion of Annotated Cohort VCF",

"uuid": "UDPM-791",

"owner": "UDPM-67",

"managers": "UDPM-27",

"performers": "UDPM-3",

"show_options": {

"top": 377,

"left": 215

},

"end_task": true

},

{

"name": "Receive/Create Cohort VCF",

"uuid": "UDPM-789",

"owner": "UDPM-67",

"managers": "UDPM-27",

"performers": "UDPM-3",

"tools": [

{

"name": "VCF Information",

"uuid": "UDPM-989",

"input_type": "UDPM-195",

"output_type": "UDPM-195",

"flags": 1,

"options": {

"udfs": [

"UDPM-2032",

"UDPM-15",

"UDPM-2051",

"UDPM-1656",

"UDPM-2049",

"UDPM-1094",

"UDPM-2053"

]

}

}

],

"next_states": [

{

"state_name": "Annotation of Cohort VCF",

"state_uuid": "UDPM-790",

"btn_text": "Begin Annotation",

"btn_css": "#456"

}

],

"show_options": {

"top": 57,

"left": 64

}

}

],

"entry_point": "UDPM-789",

"show_udfs": "UDPM-2051,UDPM-2032,UDPM-1656,UDPM-15"

},{

"name": "SNP Chip Sequencing",

"uuid": "UDPM-166",

"subject_type": "UDPM-203",

"state_defs": [

{

"name": "Cancelled",

"uuid": "UDPM-804",

"owner": "UDPM-67",

"managers": "UDPM-9",

"performers": "UDPM-2",

"show_options": {

"top": 440,

"left": 66

},

"end_task": true

},

{

"name": "Data Received and Imported",

"uuid": "UDPM-807",

"owner": "UDPM-67",

"managers": "UDPM-27",

"performers": "UDPM-3",

"tools": [

{

"name": "GenomeStudio Information",

"uuid": "UDPM-1189",

"input_type": "UDPM-203",

"output_type": "UDPM-203",

"flags": 1,

"options": {

"udfs": [

"UDPM-868",

"UDPM-2461"

]

}

},

{

"name": "Begin SNP Analysis",

"uuid": "UDPM-1009",

"input_type": "UDPM-203",

"output_type": "UDPM-59",

"flags": 0,

"options": {

"obj_type": "SubjectType"

}

}

],

"show_options": {

"top": 248,

"left": 758

},

"end_task": true

},

{

"name": "Hold",

"uuid": "UDPM-803",

"owner": "UDPM-67",

"managers": "UDPM-9",

"performers": "UDPM-44",

"duration": 30.0,

"next_states": [

{

"state_name": "Cancelled",

"state_uuid": "UDPM-804",

"btn_text": "Cancel",

"btn_css": "#456"

},

{

"state_name": "SNP Chip Wait List",

"state_uuid": "UDPM-801",

"btn_text": "Return to Wait List",

"btn_css": "#456"

}

],

"show_options": {

"top": 270,

"left": 52

}

},

{

"name": "Import Raw Data from Core Lab",

"uuid": "UDPM-806",

"owner": "UDPM-67",

"managers": "UDPM-27",

"performers": "UDPM-3",

"duration": 7.0,

"tools": [

{

"name": "IDAT Files",

"uuid": "UDPM-1008",

"input_type": "UDPM-203",

"output_type": "UDPM-203",

"flags": 1,

"options": {

"udfs": [

"UDPM-390",

"UDPM-391",

"UDPM-2149",

"UDPM-1277"

]

}

}

],

"next_states": [

{

"state_name": "Data Received and Imported",

"state_uuid": "UDPM-807",

"btn_text": "Data Received and Imported",

"btn_css": "#456"

},

{

"state_name": "Cancelled",

"state_uuid": "UDPM-804",

"btn_text": "Cancel",

"btn_css": "rgb(13, 73, 123)",

"btn_scale": "large"

}

],

"show_options": {

"top": 451,

"left": 526

}

},

{

"name": "Prepare DNA for Shipment",

"uuid": "UDPM-802",

"owner": "UDPM-67",

"managers": "UDPM-44",

"performers": "UDPM-42",

"duration": 7.0,

"tools": [

{

"name": "Shipment Information",

"uuid": "UDPM-1007",

"input_type": "UDPM-203",

"output_type": "UDPM-203",

"flags": 1,

"options": {

"udfs": [

"UDPM-861",

"UDPM-71"

]

}

},

{

"name": "DNA Preparation",

"uuid": "UDPM-1006",

"input_type": "UDPM-203",

"output_type": "UDPM-203",

"flags": 1,

"options": {

"udfs": [

"UDPM-451",

"UDPM-1346",

"UDPM-1347",

"UDPM-1345",

"UDPM-2426",

"UDPM-455"

]

}

}

],

"next_states": [

{

"state_name": "SNP Chip Sent",

"state_uuid": "UDPM-805",

"btn_text": "DNA Sent for SNP Chip",

"btn_css": "#456"

},

{

"state_name": "Cancelled",

"state_uuid": "UDPM-804",

"btn_text": "Cancel",

"btn_css": "rgb(13, 73, 123)",

"btn_scale": "large"

}

],

"show_options": {

"top": 29,

"left": 448

}

},

{

"name": "SNP Chip Sent",

"uuid": "UDPM-805",

"owner": "UDPM-67",

"managers": "UDPM-27",

"performers": "UDPM-3",

"duration": 10.0,

"next_states": [

{

"state_name": "Import Raw Data from Core Lab",

"state_uuid": "UDPM-806",

"btn_text": "Data Ready for Transfer",

"btn_css": "#456"

},

{

"state_name": "Cancelled",

"state_uuid": "UDPM-804",

"btn_text": "Cancel",

"btn_css": "rgb(13, 73, 123)",

"btn_scale": "large"

}

],

"show_options": {

"top": 264,

"left": 478

}

},

{

"name": "SNP Chip Wait List",

"uuid": "UDPM-801",

"owner": "UDPM-67",

"managers": "UDPM-9",

"performers": "UDPM-44",

"tools": [

{

"name": "Approve for SNP Submission",

"uuid": "UDPM-1005",

"input_type": "UDPM-203",

"output_type": "UDPM-203",

"flags": 6,

"after_code": "unless subj.get_value('Approved for SNP Submission')\n last_snp_sub = find_subjects do |qb|\n qb.add_subject_type('SNP submission')\n qb.order = 'id desc'\n qb.limit = 1\n end\n\n sub = last_snp_sub[0]\n sub = create_subject('SNP submission') unless sub.present? \n snps = sub.get_value('snp_submission')\n unless snps.size < 48\n sub = create_subject('SNP submission')\n snps = []\n end\n snps << subj\n sub.set_value('snp_submission', snps)\n subj.set_value('Approved for SNP Submission', 'yes')\nend",

"hide_button_if_not_condition": true,

"conditions": [

{

"name": "SNP Chip Sequencing&rarr;Approved for SNP Submission",

"condition": "Empty",

"value": "",

"message": "",

"subject_type": "UDPM-203",

"udf": "UDPM-1349"

}

]

}

],

"next_states": [

{

"state_name": "Prepare DNA for Shipment",

"state_uuid": "UDPM-802",

"btn_text": "Prepare for Shipment",

"btn_css": "#456",

"after_code": "self.next_state_performer = find_user('[redacted]')"

},

{

"state_name": "Hold",

"state_uuid": "UDPM-803",

"btn_text": "Place on Hold",

"btn_css": "#456"

}

],

"show_options": {

"top": 39,

"left": 66

}

}

],

"entry_point": "UDPM-801"

},{

"name": "Research Project Workflow",

"uuid": "UDPM-183",

"subject_type": "UDPM-218",

"state_defs": [

{

"name": "Project Closed",

"uuid": "UDPM-885",

"owner": "UDPM-67",

"managers": "UDPM-22",

"performers": "UDPM-57",

"show_options": {

"top": 106.99716186523438,

"left": 377.9971008300781

},

"end_task": true

},

{

"name": "Project in Progress",

"uuid": "UDPM-884",

"owner": "UDPM-67",

"managers": "UDPM-22",

"performers": "UDPM-57",

"tools": [

{

"name": "Cohort Research Request",

"uuid": "UDPM-1501",

"input_type": "UDPM-218",

"output_type": "UDPM-271",

"flags": 0,

"before_code": "#x = subj.get_value(subj.name)\n#\n#params[:defaults] = {\n# 'UDP Cohort'=> x #subj.name\n#}",

"options": {

"obj_type": "SubjectType"

}

},

{

"name": "Upload Cohort File",

"uuid": "UDPM-1529",

"input_type": "UDPM-218",

"output_type": "UDPM-277",

"flags": 0,

"before_code": "p = subj.get_value(\"Patients\")\n\nparams[:defaults] = {\n 'Patients'=> p\n}",

"options": {

"obj_type": "SubjectType"

}

},

{

"name": "Sanger Sequencing",

"uuid": "UDPM-1139",

"input_type": "UDPM-218",

"output_type": "UDPM-22",

"flags": 0,

"options": {

"obj_type": "SubjectType"

}

},

{

"name": "Project Information",

"uuid": "UDPM-1140",

"input_type": "UDPM-218",

"output_type": "UDPM-218",

"flags": 1,

"options": {

"udfs": [

"UDPM-2032",

"UDPM-2195",

"UDPM-2374"

]

}

}

],

"show_options": {

"top": 69,

"left": 112

},

"end_task": true

}

],

"entry_point": "UDPM-884",

"show_udfs": "UDPM-2195,UDPM-2032"

},{

"name": "Non - PII Patient File Uploads",

"uuid": "UDPM-165",

"subject_type": "UDPM-201",

"state_defs": [

{

"name": "Patient File Uploads",

"uuid": "UDPM-799",

"owner": "UDPM-67",

"managers": "UDPM-9",

"performers": "UDPM-5",

"tools": [

{

"name": "Enter File Upload Information",

"uuid": "UDPM-1002",

"input_type": "UDPM-201",

"output_type": "UDPM-201",

"flags": 1,

"options": {

"udfs": [

"UDPM-6",

"UDPM-1656",

"UDPM-1724",

"UDPM-1744",

"UDPM-1755",

"UDPM-191"

]

}

},

{

"name": "Notify Clinical Managers",

"uuid": "UDPM-1001",

"input_type": "UDPM-201",

"output_type": "UDPM-201",

"flags": 5,

"options": {

"obj_type": "EmailTemplate",

"user_groups_ids": [

"UDPM-9"

],

"obj_name": "UDPM-3"

}

}

],

"show_options": {

"top": 62.99998474121094,

"left": 113

},

"end_task": true

}

],

"entry_point": "UDPM-799",

"show_udfs": "UDPM-1724,UDPM-1744,UDPM-191,UDPM-1755,UDPM-1656"

},{

"name": "Request Glycomics Collaboration",

"uuid": "UDPM-199",

"subject_type": "UDPM-244",

"state_defs": [

{

"name": "Cancel",

"uuid": "UDPM-989",

"owner": "UDPM-67",

"managers": "UDPM-38",

"performers": "UDPM-62",

"show_options": {

"top": 124,

"left": 641

},

"end_task": true

},

{

"name": "Complete Glycomics Collaboration",

"uuid": "UDPM-1014",

"owner": "UDPM-67",

"managers": "UDPM-38",

"performers": "UDPM-62",

"show_options": {

"top": 762,

"left": 682

},

"end_task": true

},

{

"name": "Glycomics Collaboration Request - Prepare Sample",

"uuid": "UDPM-988",

"owner": "UDPM-67",

"managers": "UDPM-38",

"performers": "UDPM-62",

"tools": [

{

"name": "Sample Information",

"uuid": "UDPM-1255",

"input_type": "UDPM-244",

"output_type": "UDPM-244",

"flags": 1,

"options": {

"udfs": [

"UDPM-2624",

"UDPM-2625",

"UDPM-2626",

"UDPM-2628",

"UDPM-2629",

"UDPM-2822",

"UDPM-2823",

"UDPM-1512",

"UDPM-463",

"UDPM-1312",

"UDPM-2374"

]

}

}

],

"next_states": [

{

"state_name": "Sign Sample Shipment Letter",

"state_uuid": "UDPM-1017",

"btn_text": "Upload Unsigned Shipment Letter",

"btn_css": "#456",

"after_code": "self.next_state_performer = find_user('[redacted]')\n\nsubj.set_value('Shipment Letter', params['Shipment Letter'])\n\nsend_email(User.find_by_username('[redacted]'), find_email_template(\"-omics Shipment Letter\"), subj)\n\nsend_email(User.find_by_username('[redacted]'), find_email_template(\"-omics Shipment Letter\"), subj)\n\n\n",

"btn_scale": "large",

"after_code_params": [

"UDPM-1797"

]

},

{

"state_name": "Cancel",

"state_uuid": "UDPM-989",

"btn_text": "Cancel Request",

"btn_css": "#456"

}

],

"show_options": {

"top": 34,

"left": 42

}

},

{

"name": "Prepare Fibroblasts",

"uuid": "UDPM-1013",

"owner": "UDPM-67",

"managers": "UDPM-38",

"performers": "UDPM-62",

"tools": [

{

"name": "Begin Culture",

"uuid": "UDPM-1272",

"input_type": "UDPM-244",

"output_type": "UDPM-40",

"flags": 0,

"options": {

"obj_type": "SubjectType"

}

}

],

"next_states": [

{

"state_name": "Sign Sample Shipment Letter",

"state_uuid": "UDPM-1017",

"btn_text": "Upload Unsigned Shipment Letter",

"btn_css": "#456",

"after_code": "self.next_state_performer = find_user('[redacted]')\n\nsubj.set_value('Shipment Letter', params['Shipment Letter'])\n\nsend_email(User.find_by_username('[redacted]'), find_email_template(\"-omics Shipment Letter\"), subj)",

"before_code": "#params[:ask_next_performer] = true",

"btn_scale": "large",

"after_code_params": [

"UDPM-1797"

]

},

{

"state_name": "Cancel",

"state_uuid": "UDPM-989",

"btn_text": "Cancel",

"btn_css": "#456"

}

],

"show_options": {

"top": 413,

"left": 782

}

},

{

"name": "Reports Uploaded",

"uuid": "UDPM-993",

"owner": "UDPM-67",

"managers": "UDPM-38",

"performers": "UDPM-38",

"tools": [

{

"name": "Additional Information",

"uuid": "UDPM-1273",

"input_type": "UDPM-244",

"output_type": "UDPM-244",

"flags": 1,

"options": {

"udfs": [

"UDPM-2628",

"UDPM-2629",

"UDPM-2374"

]

}

}

],

"next_states": [

{

"state_name": "Prepare Fibroblasts",

"state_uuid": "UDPM-1013",

"btn_text": "Prepare Fibroblasts",

"btn_css": "#456",

"after_code": "self.next_state_performer = User.curr_user"

},

{

"state_name": "Complete Glycomics Collaboration",

"state_uuid": "UDPM-1014",

"btn_text": "Complete Glycomics Testing",

"btn_css": "#456"

}

],

"show_options": {

"top": 689,

"left": 405

}

},

{

"name": "Samples Received",

"uuid": "UDPM-990",

"owner": "UDPM-67",

"managers": "UDPM-38",

"performers": "UDPM-62",

"tools": [

{

"name": "Upload Report",

"uuid": "UDPM-1257",

"input_type": "UDPM-244",

"output_type": "UDPM-178",

"flags": 0,

"options": {

"obj_type": "SubjectType"

}

},

{

"name": "Analyze Results",

"uuid": "UDPM-1250",

"input_type": "UDPM-244",

"output_type": "UDPM-224",

"description": "Add as a Glycome Patient",

"flags": 0,

"options": {
[truncated: 2,512 more chars]
