# Supplementary material for: Distributed Cognition and Process Management Enabling Individualized Translational Research: The NIH Undiagnosed Diseases Program Experience
Source: Front Med (Lausanne). 2016 Oct 12;3:39. doi: 10.3389/fmed.2016.00039 (PMC5060938; doi:10.3389/fmed.2016.00039)
Supplement: Supplementary file 4 [file data_sheet_3.doc]

Ôªø{

"Total": 2124,

"Result Type": "UserFields",

"UserFields": [

{

"id": 2167,

"obj_id": 2167,

"display_name": "1K Frequency",

"name": "1K Frequency",

"type": "Text Field",

"values": "",

"created_at": "01/17/2014",

"updated_at": "10/30/2014",

"created_by": [redacted],

"searchable_quick": true,

"searchable_advanced": true,

"searchable_batch": true,

"searchable": "Quick,Advanced,Batch",

"pii": "No",

"qtip": "",

"used_by": "Exome Analysis Results",

"used_by_display": "<a href='#' onClick='remoteJS({name:\"dialogs/AddSubjectTypeDlg\", obj_id:156 });'>Exome Analysis Results</a>"

},

{

"id": 910,

"obj_id": 910,

"display_name": "2nd Reviewer",

"name": "2nd Reviewer",

"type": "Text Field",

"values": "",

"created_at": "05/10/2013",

"updated_at": "10/30/2014",

"created_by": [redacted],

"searchable_quick": true,

"searchable_advanced": true,

"searchable_batch": true,

"searchable": "Quick,Advanced,Batch",

"pii": "No",

"qtip": "",

"used_by": "Sharepoint Legacy - Pediatric Patient, Sharepoint Legacy - Adult Patient Status",

"used_by_display": "<a href='#' onClick='remoteJS({name:\"dialogs/AddSubjectTypeDlg\", obj_id:114 });'>Sharepoint Legacy - Pediatric Patient</a>, <a href='#' onClick='remoteJS({name:\"dialogs/AddSubjectTypeDlg\", obj_id:115 });'>Sharepoint Legacy - Adult Patient Status</a>"

},

{

"id": 937,

"obj_id": 937,

"display_name": "3rd Review Date",

"name": "3rd Review Date",

"type": "Date",

"values": "",

"created_at": "05/10/2013",

"updated_at": "10/30/2014",

"created_by": [redacted],

"searchable_quick": true,

"searchable_advanced": true,

"searchable_batch": true,

"searchable": "Quick,Advanced,Batch",

"pii": "No",

"qtip": "",

"used_by": "Sharepoint Legacy - Adult Patient Status",

"used_by_display": "<a href='#' onClick='remoteJS({name:\"dialogs/AddSubjectTypeDlg\", obj_id:115 });'>Sharepoint Legacy - Adult Patient Status</a>"

},

{

"id": 938,

"obj_id": 938,

"display_name": "3rd Reviewer",

"name": "3rd Reviewer",

"type": "Text Field",

"values": "",

"created_at": "05/10/2013",

"updated_at": "10/30/2014",

"created_by": [redacted],

"searchable_quick": true,

"searchable_advanced": true,

"searchable_batch": true,

"searchable": "Quick,Advanced,Batch",

"pii": "No",

"qtip": "",

"used_by": "Sharepoint Legacy - Adult Patient Status",

"used_by_display": "<a href='#' onClick='remoteJS({name:\"dialogs/AddSubjectTypeDlg\", obj_id:115 });'>Sharepoint Legacy - Adult Patient Status</a>"

},

{

"id": 939,

"obj_id": 939,

"display_name": "3rd Reviewer Return Date",

"name": "3rd Reviewer Return Date",

"type": "Date",

"values": "",

"created_at": "05/10/2013",

"updated_at": "10/30/2014",

"created_by": [redacted],

"searchable_quick": true,

"searchable_advanced": true,

"searchable_batch": true,

"searchable": "Quick,Advanced,Batch",

"pii": "No",

"qtip": "",

"used_by": "Sharepoint Legacy - Adult Patient Status",

"used_by_display": "<a href='#' onClick='remoteJS({name:\"dialogs/AddSubjectTypeDlg\", obj_id:115 });'>Sharepoint Legacy - Adult Patient Status</a>"

},

{

"id": 1931,

"obj_id": 1931,

"display_name": "3'-Tags",

"name": "3'-Tags",

"type": "List",

"values": "EGFP, FLAG, GST, HA, HIS, HSV, None, PYO, V5, VSV-G, c-MYC, DDK",

"created_at": "11/11/2013",

"updated_at": "10/30/2014",

"created_by": [redacted],

"searchable_quick": true,

"searchable_advanced": true,

"searchable_batch": true,

"searchable": "Quick,Advanced,Batch",

"pii": "No",

"qtip": "",

"used_by": "Glycerol Stock",

"used_by_display": "<a href='#' onClick='remoteJS({name:\"dialogs/AddSubjectTypeDlg\", obj_id:171 });'>Glycerol Stock</a>"

},

{

"id": 1930,

"obj_id": 1930,

"display_name": "5'-Tags",

"name": "5'-Tags",

"type": "List",

"values": "EGFP, FLAG, GST, HA, HIS, HS, None, PYO, V5, VSV-G, c-MYC, DDK",

"created_at": "11/11/2013",

"updated_at": "10/30/2014",

"created_by": [redacted],

"searchable_quick": true,

"searchable_advanced": true,

"searchable_batch": true,

"searchable": "Quick,Advanced,Batch",

"pii": "No",

"qtip": "",

"used_by": "Glycerol Stock",

"used_by_display": "<a href='#' onClick='remoteJS({name:\"dialogs/AddSubjectTypeDlg\", obj_id:171 });'>Glycerol Stock</a>"

},

{

"id": 2541,

"obj_id": 2541,

"display_name": "AA_MAF(%)",

"name": "AA_MAF(%)",

"type": "Text Field",

"values": "",

"created_at": "04/21/2014",

"updated_at": "10/30/2014",

"created_by": [redacted],

"searchable_quick": true,

"searchable_advanced": true,

"searchable_batch": true,

"searchable": "Quick,Advanced,Batch",

"pii": "No",

"qtip": "",

"used_by": "Exome Analysis Results",

"used_by_display": "<a href='#' onClick='remoteJS({name:\"dialogs/AddSubjectTypeDlg\", obj_id:156 });'>Exome Analysis Results</a>"

},

{

"id": 2668,

"obj_id": 2668,

"display_name": "AB1 File",

"name": "AB1 File",

"type": "File",

"values": "",

"created_at": "06/11/2014",

"updated_at": "10/30/2014",

"created_by": [redacted],

"searchable_quick": true,

"searchable_advanced": true,

"searchable_batch": true,

"searchable": "Quick,Advanced,Batch",

"pii": "No",

"qtip": "",

"used_by": "AB1 Sequence Files",

"used_by_display": "<a href='#' onClick='remoteJS({name:\"dialogs/AddSubjectTypeDlg\", obj_id:250 });'>AB1 Sequence Files</a>"

},

{

"id": 2845,

"obj_id": 2845,

"display_name": "AB1 Files",

"name": "AB1 Files",

"type": "Subject Back Ref",

"values": "",

"created_at": "09/24/2014",

"updated_at": "10/30/2014",

"created_by": [redacted],

"searchable_quick": true,

"searchable_advanced": true,

"searchable_batch": true,

"searchable": "Quick,Advanced,Batch",

"pii": "No",

"qtip": "",

"used_by": "Sanger Interpretation, Destination Vector Construction, Entry Vector, Mutagenized Vectors",

"used_by_display": "<a href='#' onClick='remoteJS({name:\"dialogs/AddSubjectTypeDlg\", obj_id:22 });'>Sanger Interpretation</a>, <a href='#' onClick='remoteJS({name:\"dialogs/AddSubjectTypeDlg\", obj_id:168 });'>Destination Vector Construction</a>, <a href='#' onClick='remoteJS({name:\"dialogs/AddSubjectTypeDlg\", obj_id:193 });'>Entry Vector</a>, <a href='#' onClick='remoteJS({name:\"dialogs/AddSubjectTypeDlg\", obj_id:228 });'>Mutagenized Vectors</a>"

},

{

"id": 2808,

"obj_id": 2808,

"display_name": "Abnormal",

"name": "Abnormal",

"type": "Text Field",

"values": "",

"created_at": "08/28/2014",

"updated_at": "10/30/2014",

"created_by": [redacted],

"searchable_quick": false,

"searchable_advanced": true,

"searchable_batch": false,

"searchable": "Advanced",

"pii": "No",

"qtip": "Yes/No determined from Phenote",

"used_by": "Zebrafish Phenotype, Drosophila Phenotypes",

"used_by_display": "<a href='#' onClick='remoteJS({name:\"dialogs/AddSubjectTypeDlg\", obj_id:173 });'>Zebrafish Phenotype</a>, <a href='#' onClick='remoteJS({name:\"dialogs/AddSubjectTypeDlg\", obj_id:265 });'>Drosophila Phenotypes</a>"

},

{

"id": 2456,

"obj_id": 2456,

"display_name": "Abnormal Glycome Results Flag",

"name": "Abnormal Glycome Results Flag",

"type": "Formula",

"values": "",

"created_at": "03/25/2014",

"updated_at": "03/27/2014",

"created_by": [redacted],

"searchable_quick": false,

"searchable_advanced": null,

"searchable_batch": null,

"searchable": "",

"pii": "No",

"qtip": null,

"used_by": "",

"used_by_display": ""

},

{

"id": 1987,

"obj_id": 1987,

"display_name": "Accept",

"name": "Accept",

"type": "HTML",

"values": "",

"created_at": "11/20/2013",

"updated_at": "06/10/2014",

"created_by": [redacted],

"searchable_quick": false,

"searchable_advanced": null,

"searchable_batch": null,

"searchable": "",

"pii": "Yes",

"qtip": "",

"used_by": "Patient Letter",

"used_by_display": "<a href='#' onClick='remoteJS({name:\"dialogs/AddSubjectTypeDlg\", obj_id:87 });'>Patient Letter</a>"

},

{

"id": 1561,

"obj_id": 1561,

"display_name": "Acceptance Letter Sent",

"name": "Acceptance Letter Sent",

"type": "Radio Buttons",

"values": "Yes, No",

"created_at": "08/12/2013",

"updated_at": "10/30/2014",

"created_by": [redacted],

"searchable_quick": true,

"searchable_advanced": true,

"searchable_batch": true,

"searchable": "Quick,Advanced,Batch",

"pii": "No",

"qtip": "",

"used_by": "Patient Visit Tools, Patient Visit Planning for Version C",

"used_by_display": "<a href='#' onClick='remoteJS({name:\"dialogs/AddSubjectTypeDlg\", obj_id:163 });'>Patient Visit Tools</a>, <a href='#' onClick='remoteJS({name:\"dialogs/AddSubjectTypeDlg\", obj_id:192 });'>Patient Visit Planning for Version C</a>"

},

{

"id": 1562,

"obj_id": 1562,

"display_name": "Acceptance Letter Uploaded to LIMS",

"name": "Acceptance Letter Uploaded to LIMS",

"type": "Radio Buttons",

"values": "Yes, No",

"created_at": "08/12/2013",

"updated_at": "10/30/2014",

"created_by": [redacted],

"searchable_quick": true,

"searchable_advanced": true,

"searchable_batch": true,

"searchable": "Quick,Advanced,Batch",

"pii": "No",

"qtip": "",

"used_by": "Patient Visit Tools, Patient Visit Planning for Version C",

"used_by_display": "<a href='#' onClick='remoteJS({name:\"dialogs/AddSubjectTypeDlg\", obj_id:163 });'>Patient Visit Tools</a>, <a href='#' onClick='remoteJS({name:\"dialogs/AddSubjectTypeDlg\", obj_id:192 });'>Patient Visit Planning for Version C</a>"

},

{

"id": 2718,

"obj_id": 2718,

"display_name": "Accept Ltr",

"name": "Accept Ltr",

"type": "Text Field",

"values": "",

"created_at": "07/18/2014",

"updated_at": "10/30/2014",

"created_by": [redacted],

"searchable_quick": true,

"searchable_advanced": true,

"searchable_batch": true,

"searchable": "Quick,Advanced,Batch",

"pii": "No",

"qtip": "",

"used_by": "",

"used_by_display": ""

},

{

"id": 1988,

"obj_id": 1988,

"display_name": "Accept/Refer",

"name": "Accept/Refer",

"type": "HTML",

"values": "",

"created_at": "11/20/2013",

"updated_at": "06/10/2014",

"created_by": [redacted],

"searchable_quick": false,

"searchable_advanced": null,

"searchable_batch": null,

"searchable": "",

"pii": "Yes",

"qtip": "",

"used_by": "Patient Letter",

"used_by_display": "<a href='#' onClick='remoteJS({name:\"dialogs/AddSubjectTypeDlg\", obj_id:87 });'>Patient Letter</a>"

},

{

"id": 2719,

"obj_id": 2719,

"display_name": "Accept/Reference Ltr",

"name": "Accept/Reference Ltr",

"type": "Text Field",

"values": "",

"created_at": "07/18/2014",

"updated_at": "10/30/2014",

"created_by": [redacted],

"searchable_quick": true,

"searchable_advanced": true,

"searchable_batch": true,

"searchable": "Quick,Advanced,Batch",

"pii": "No",

"qtip": "",

"used_by": "",

"used_by_display": ""

},

{

"id": 475,

"obj_id": 475,

"display_name": "Access Group Id List",

"name": "Access Group Id List",

"type": "Text Field",

"values": "",

"created_at": "05/07/2013",

"updated_at": "11/03/2014",

"created_by": [redacted],

"searchable_quick": true,

"searchable_advanced": true,

"searchable_batch": true,

"searchable": "Quick,Advanced,Batch",

"pii": "No",

"qtip": "",

"used_by": "Labmatrix Legacy - Communications",

"used_by_display": "<a href='#' onClick='remoteJS({name:\"dialogs/AddSubjectTypeDlg\", obj_id:92 });'>Labmatrix Legacy - Communications</a>"

},

{

"id": 2869,

"obj_id": 2869,

"display_name": "Accession Number",

"name": "Accession Number",

"type": "Text Field",

"values": "",

"created_at": "10/30/2014",

"updated_at": "11/03/2014",

"created_by": [redacted],

"searchable_quick": true,

"searchable_advanced": true,

"searchable_batch": true,

"searchable": "Quick,Advanced,Batch",

"pii": "No",

"qtip": "",

"used_by": "Lymphoblast Culture Request",

"used_by_display": "<a href='#' onClick='remoteJS({name:\"dialogs/AddSubjectTypeDlg\", obj_id:272 });'>Lymphoblast Culture Request</a>"

},

{

"id": 2309,

"obj_id": 2309,

"display_name": "Account Acess Approval Notes",

"name": "Account Acess Approval Notes",

"type": "Text Area",

"values": "",

"created_at": "02/27/2014",

"updated_at": "11/03/2014",

"created_by": [redacted],

"searchable_quick": false,

"searchable_advanced": false,

"searchable_batch": false,

"searchable": "",

"pii": "No",

"qtip": "",

"used_by": "",

"used_by_display": ""

},

{

"id": 1832,

"obj_id": 1832,

"display_name": "ACD Tube for Lymphoblastoid Cell Line",

"name": "ACD Tube for Lymphoblastoid Cell Line",

"type": "CheckBox",

"values": "",

"created_at": "10/30/2013",

"updated_at": "11/03/2014",

"created_by": [redacted],

"searchable_quick": true,

"searchable_advanced": true,

"searchable_batch": true,

"searchable": "Quick,Advanced,Batch",

"pii": "No",

"qtip": "Acid-Citrate-Dextrose (yellow top) tube to be sent to building 35 for lymphoblastoid cell line development",

"used_by": "Blood Kit, Research Sample",

"used_by_display": "<a href='#' onClick='remoteJS({name:\"dialogs/AddSubjectTypeDlg\", obj_id:33 });'>Blood Kit</a>, <a href='#' onClick='remoteJS({name:\"dialogs/AddSubjectTypeDlg\", obj_id:86 });'>Research Sample</a>"

},

{

"id": 476,

"obj_id": 476,

"display_name": "Acronym",

"name": "Acronym",

"type": "Text Field",

"values": "",

"created_at": "05/07/2013",

"updated_at": "11/03/2014",

"created_by": [redacted],

"searchable_quick": true,

"searchable_advanced": true,

"searchable_batch": true,

"searchable": "Quick,Advanced,Batch",

"pii": "No",

"qtip": "",

"used_by": "Labmatrix Legacy - Organization Contacts",

"used_by_display": "<a href='#' onClick='remoteJS({name:\"dialogs/AddSubjectTypeDlg\", obj_id:94 });'>Labmatrix Legacy - Organization Contacts</a>"

},

{

"id": 1398,

"obj_id": 1398,

"display_name": "Active?",

"name": "Active?",

"type": "Choice",

"values": "Yes, No",

"created_at": "07/08/2013",

"updated_at": "11/03/2014",

"created_by": [redacted],

"searchable_quick": true,

"searchable_advanced": true,

"searchable_batch": true,

"searchable": "Quick,Advanced,Batch",

"pii": "No",

"qtip": "",

"used_by": "",

"used_by_display": ""

},

{

"id": 1861,

"obj_id": 1861,

"display_name": "Active Collaboration",

"name": "Active Collaboration",

"type": "Choice",

"values": "Yes, No",

"created_at": "11/01/2013",

"updated_at": "11/03/2014",

"created_by": [redacted],

"searchable_quick": true,

"searchable_advanced": true,

"searchable_batch": true,

"searchable": "Quick,Advanced,Batch",

"pii": "No",

"qtip": "",

"used_by": "Information Technology Collaboration",

"used_by_display": "<a href='#' onClick='remoteJS({name:\"dialogs/AddSubjectTypeDlg\", obj_id:183 });'>Information Technology Collaboration</a>"

},

{

"id": 2928,

"obj_id": 2928,

"display_name": "Active Region Analysis Report",

"name": "Active Region Analysis Report",

"type": "File",

"values": "",

"created_at": "12/08/2014",

"updated_at": "12/08/2014",

"created_by": [redacted],

"searchable_quick": false,

"searchable_advanced": true,

"searchable_batch": false,

"searchable": "Advanced",

"pii": "No",

"qtip": "",

"used_by": "ChIP-Seq",

"used_by_display": "<a href='#' onClick='remoteJS({name:\"dialogs/AddSubjectTypeDlg\", obj_id:254 });'>ChIP-Seq</a>"

},

{

"id": 2310,

"obj_id": 2310,

"display_name": "Activities Requested",

"name": "Activities Requested",

"type": "Text Area",

"values": "",

"created_at": "02/27/2014",

"updated_at": "12/10/2014",

"created_by": [redacted],

"searchable_quick": false,

"searchable_advanced": false,

"searchable_batch": false,

"searchable": "",

"pii": "No",

"qtip": "What activities do you want to perform in the system",

"used_by": "",

"used_by_display": ""

},

{

"id": 1563,

"obj_id": 1563,

"display_name": "Added to Calendar",

"name": "Added to Calendar",

"type": "Radio Buttons",

"values": "Yes, No",

"created_at": "08/12/2013",

"updated_at": "11/03/2014",

"created_by": [redacted],

"searchable_quick": true,

"searchable_advanced": true,

"searchable_batch": true,

"searchable": "Quick,Advanced,Batch",

"pii": "No",

"qtip": "",

"used_by": "Patient Visit Tools, Patient Visit Planning for Version C",

"used_by_display": "<a href='#' onClick='remoteJS({name:\"dialogs/AddSubjectTypeDlg\", obj_id:163 });'>Patient Visit Tools</a>, <a href='#' onClick='remoteJS({name:\"dialogs/AddSubjectTypeDlg\", obj_id:192 });'>Patient Visit Planning for Version C</a>"

},

{

"id": 2038,

"obj_id": 2038,

"display_name": "Additional Address",

"name": "Additional Address",

"type": "Text Area",

"values": "",

"created_at": "12/02/2013",

"updated_at": "11/03/2014",

"created_by": [redacted],

"searchable_quick": false,

"searchable_advanced": false,

"searchable_batch": false,

"searchable": "",

"pii": "No",

"qtip": "",

"used_by": "Sequencing Centers",

"used_by_display": "<a href='#' onClick='remoteJS({name:\"dialogs/AddSubjectTypeDlg\", obj_id:196 });'>Sequencing Centers</a>"

},

{

"id": 2366,

"obj_id": 2366,

"display_name": "Additional Breeding Done",

"name": "Additional Breeding Done",

"type": "Choice",

"values": "yes, no",

"created_at": "03/05/2014",

"updated_at": "11/03/2014",

"created_by": [redacted],

"searchable_quick": true,

"searchable_advanced": true,

"searchable_batch": true,

"searchable": "Quick,Advanced,Batch",

"pii": "No",

"qtip": "",

"used_by": "Zebrafish Mutation Project",

"used_by_display": "<a href='#' onClick='remoteJS({name:\"dialogs/AddSubjectTypeDlg\", obj_id:186 });'>Zebrafish Mutation Project</a>"

},

{

"id": 1440,

"obj_id": 1440,

"display_name": "Additional comments",

"name": "Additional comments",

"type": "Text Area",

"values": "",

"created_at": "07/10/2013",

"updated_at": "11/03/2014",

"created_by": [redacted],

"searchable_quick": false,

"searchable_advanced": false,

"searchable_batch": false,

"searchable": "",

"pii": "No",

"qtip": "",

"used_by": "",

"used_by_display": ""

},

{

"id": 2123,

"obj_id": 2123,

"display_name": "Additional Contact Information",

"name": "Additional Contact Information",

"type": "Text Field",

"values": "",

"created_at": "12/20/2013",

"updated_at": "11/03/2014",

"created_by": [redacted],

"searchable_quick": true,

"searchable_advanced": true,

"searchable_batch": true,

"searchable": "Quick,Advanced,Batch",

"pii": "Yes",

"qtip": "",

"used_by": "Patient, Patient Follow Up Visit",

"used_by_display": "<a href='#' onClick='remoteJS({name:\"dialogs/AddSubjectTypeDlg\", obj_id:1 });'>Patient</a>, <a href='#' onClick='remoteJS({name:\"dialogs/AddSubjectTypeDlg\", obj_id:264 });'>Patient Follow Up Visit</a>"

},

{

"id": 1887,

"obj_id": 1887,

"display_name": "Additional Frequency Information",

"name": "Additional Frequency Information",

"type": "Text Area",

"values": "",

"created_at": "11/07/2013",

"updated_at": "11/03/2014",

"created_by": [redacted],

"searchable_quick": false,

"searchable_advanced": false,

"searchable_batch": false,

"searchable": "",

"pii": "No",

"qtip": "",

"used_by": "Exome Analysis Results",

"used_by_display": "<a href='#' onClick='remoteJS({name:\"dialogs/AddSubjectTypeDlg\", obj_id:156 });'>Exome Analysis Results</a>"

},

{

"id": 1280,

"obj_id": 1280,

"display_name": "Additional Gel Images",

"name": "Additional Gel Images",

"type": "File",

"values": "",

"created_at": "06/04/2013",

"updated_at": "11/03/2014",

"created_by": [redacted],

"searchable_quick": true,

"searchable_advanced": true,

"searchable_batch": true,

"searchable": "Quick,Advanced,Batch",

"pii": "No",

"qtip": "",

"used_by": "Sanger Interpretation, Destination Vector Construction, Entry Vector, Mutagenized Vectors",

"used_by_display": "<a href='#' onClick='remoteJS({name:\"dialogs/AddSubjectTypeDlg\", obj_id:22 });'>Sanger Interpretation</a>, <a href='#' onClick='remoteJS({name:\"dialogs/AddSubjectTypeDlg\", obj_id:168 });'>Destination Vector Construction</a>, <a href='#' onClick='remoteJS({name:\"dialogs/AddSubjectTypeDlg\", obj_id:193 });'>Entry Vector</a>, <a href='#' onClick='remoteJS({name:\"dialogs/AddSubjectTypeDlg\", obj_id:228 });'>Mutagenized Vectors</a>"

},

{

"id": 1874,

"obj_id": 1874,

"display_name": "Additional IGV View",

"name": "Additional IGV View",

"type": "File",

"values": "",

"created_at": "11/07/2013",

"updated_at": "11/03/2014",

"created_by": [redacted],

"searchable_quick": true,

"searchable_advanced": true,

"searchable_batch": true,

"searchable": "Quick,Advanced,Batch",

"pii": "No",

"qtip": "",

"used_by": "Exome Analysis Results",

"used_by_display": "<a href='#' onClick='remoteJS({name:\"dialogs/AddSubjectTypeDlg\", obj_id:156 });'>Exome Analysis Results</a>"

},

{

"id": 2700,

"obj_id": 2700,

"display_name": "Additional MTA/ITA Details",

"name": "Additional MTA/ITA Details",

"type": "Text Area",

"values": "",

"created_at": "06/27/2014",

"updated_at": "11/03/2014",

"created_by": [redacted],

"searchable_quick": false,

"searchable_advanced": false,

"searchable_batch": false,

"searchable": "",

"pii": "No",

"qtip": "",

"used_by": "Collaboration Projects",

"used_by_display": "<a href='#' onClick='remoteJS({name:\"dialogs/AddSubjectTypeDlg\", obj_id:204 });'>Collaboration Projects</a>"

},

{

"id": 1301,

"obj_id": 1301,

"display_name": "Additional Notes",

"name": "Additional Notes",

"type": "Text Area",

"values": "",

"created_at": "06/10/2013",

"updated_at": "10/29/2014",

"created_by": [redacted],

"searchable_quick": false,

"searchable_advanced": false,

"searchable_batch": false,

"searchable": "",

"pii": "No",

"qtip": "",

"used_by": "Research Sample, Cohort Research Request, Sensitive Family Relationship Information",

"used_by_display": "<a href='#' onClick='remoteJS({name:\"dialogs/AddSubjectTypeDlg\", obj_id:86 });'>Research Sample</a>, <a href='#' onClick='remoteJS({name:\"dialogs/AddSubjectTypeDlg\", obj_id:271 });'>Cohort Research Request</a>, <a href='#' onClick='remoteJS({name:\"dialogs/AddSubjectTypeDlg\", obj_id:276 });'>Sensitive Family Relationship Information</a>"

},

{

"id": 2494,

"obj_id": 2494,

"display_name": "Additional Protocol Name",

"name": "Additional Protocol Name",

"type": "Text Field",

"values": "",

"created_at": "04/07/2014",

"updated_at": "04/15/2014",

"created_by": [redacted],

"searchable_quick": true,

"searchable_advanced": true,

"searchable_batch": true,

"searchable": "Quick,Advanced,Batch",

"pii": "No",

"qtip": "Name of Additional Protcol patient is enrolled on",

"used_by": "Patient, Patient Follow Up Visit",

"used_by_display": "<a href='#' onClick='remoteJS({name:\"dialogs/AddSubjectTypeDlg\", obj_id:1 });'>Patient</a>, <a href='#' onClick='remoteJS({name:\"dialogs/AddSubjectTypeDlg\", obj_id:264 });'>Patient Follow Up Visit</a>"

},

{

"id": 2291,

"obj_id": 2291,

"display_name": "Additional Publication",

"name": "Additional Publication",

"type": "File",

"values": "",

"created_at": "02/24/2014",

"updated_at": "03/31/2014",

"created_by": [redacted],

"searchable_quick": true,

"searchable_advanced": true,

"searchable_batch": true,

"searchable": "Quick,Advanced,Batch",

"pii": "No",

"qtip": "PDF of published paper regarding the diagnosis",

"used_by": "Diagnosis",

"used_by_display": "<a href='#' onClick='remoteJS({name:\"dialogs/AddSubjectTypeDlg\", obj_id:185 });'>Diagnosis</a>"

},

{

"id": 2473,

"obj_id": 2473,

"display_name": "Additional Results File",

"name": "Additional Results File",

"type": "File",

"values": "",

"created_at": "03/31/2014",

"updated_at": "11/03/2014",

"created_by": [redacted],

"searchable_quick": true,

"searchable_advanced": true,

"searchable_batch": false,

"searchable": "Quick,Advanced",

"pii": "No",

"qtip": "",

"used_by": "Assay",

"used_by_display": "<a href='#' onClick='remoteJS({name:\"dialogs/AddSubjectTypeDlg\", obj_id:225 });'>Assay</a>"

},

{

"id": 2474,

"obj_id": 2474,

"display_name": "Additional Results File 2",

"name": "Additional Results File 2",

"type": "File",

"values": "",

"created_at": "03/31/2014",

"updated_at": "11/03/2014",

"created_by": [redacted],

"searchable_quick": true,

"searchable_advanced": true,

"searchable_batch": false,

"searchable": "Quick,Advanced",

"pii": "No",

"qtip": "",

"used_by": "Assay",

"used_by_display": "<a href='#' onClick='remoteJS({name:\"dialogs/AddSubjectTypeDlg\", obj_id:225 });'>Assay</a>"

},

{

"id": 2475,

"obj_id": 2475,

"display_name": "Additional Results File 3",

"name": "Additional Results File 3",

"type": "File",

"values": "",

"created_at": "03/31/2014",

"updated_at": "11/03/2014",

"created_by": [redacted],

"searchable_quick": true,

"searchable_advanced": true,

"searchable_batch": false,

"searchable": "Quick,Advanced",

"pii": "No",

"qtip": "",

"used_by": "Assay",

"used_by_display": "<a href='#' onClick='remoteJS({name:\"dialogs/AddSubjectTypeDlg\", obj_id:225 });'>Assay</a>"

},

{

"id": 2295,

"obj_id": 2295,

"display_name": "Additional Supporting File",

"name": "Additional Supporting File",

"type": "File",

"values": "",

"created_at": "02/24/2014",

"updated_at": "11/03/2014",

"created_by": [redacted],

"searchable_quick": true,

"searchable_advanced": true,

"searchable_batch": false,

"searchable": "Quick,Advanced",

"pii": "Yes",

"qtip": "",

"used_by": "Exome Analysis Results, Diagnosis",

"used_by_display": "<a href='#' onClick='remoteJS({name:\"dialogs/AddSubjectTypeDlg\", obj_id:156 });'>Exome Analysis Results</a>, <a href='#' onClick='remoteJS({name:\"dialogs/AddSubjectTypeDlg\", obj_id:185 });'>Diagnosis</a>"

},

{

"id": 2463,

"obj_id": 2463,

"display_name": "Additional Testing and Reasoning",

"name": "Additional Testing and Reasoning",

"type": "Text Area",

"values": "",

"created_at": "03/27/2014",

"updated_at": "11/03/2014",

"created_by": [redacted],

"searchable_quick": false,

"searchable_advanced": false,

"searchable_batch": false,

"searchable": "",

"pii": "No",

"qtip": "",

"used_by": "Glycome Patient",

"used_by_display": "<a href='#' onClick='remoteJS({name:\"dialogs/AddSubjectTypeDlg\", obj_id:224 });'>Glycome Patient</a>"

},

{

"id": 51,

"obj_id": 51,

"display_name": "Address",

"name": "Address",

"type": "Text Field",

"values": "",

"created_at": "02/05/2013",

"updated_at": "11/03/2014",

"created_by": [redacted],

"searchable_quick": true,

"searchable_advanced": true,

"searchable_batch": false,

"searchable": "Quick,Advanced",

"pii": "Yes",

"qtip": "",

"used_by": "Blood Kit, Patient Review, Question to Primary Care /Family",

"used_by_display": "<a href='#' onClick='remoteJS({name:\"dialogs/AddSubjectTypeDlg\", obj_id:33 });'>Blood Kit</a>, <a href='#' onClick='remoteJS({name:\"dialogs/AddSubjectTypeDlg\", obj_id:83 });'>Patient Review</a>, <a href='#' onClick='remoteJS({name:\"dialogs/AddSubjectTypeDlg\", obj_id:84 });'>Question to Primary Care /Family</a>"

},

{

"id": 477,

"obj_id": 477,

"display_name": "Address - City",

"name": "Address - City",

"type": "Text Field",

"values": "",

"created_at": "05/07/2013",

"updated_at": "11/03/2014",

"created_by": [redacted],

"searchable_quick": true,

"searchable_advanced": true,

"searchable_batch": false,

"searchable": "Quick,Advanced",

"pii": "Yes",

"qtip": "",

"used_by": "Labmatrix Legacy - Persons Contact, Patient Visit Tools",

"used_by_display": "<a href='#' onClick='remoteJS({name:\"dialogs/AddSubjectTypeDlg\", obj_id:95 });'>Labmatrix Legacy - Persons Contact</a>, <a href='#' onClick='remoteJS({name:\"dialogs/AddSubjectTypeDlg\", obj_id:163 });'>Patient Visit Tools</a>"

},

{

"id": 478,

"obj_id": 478,

"display_name": "Address - Country",

"name": "Address - Country",

"type": "Text Field",

"values": "",

"created_at": "05/07/2013",

"updated_at": "11/03/2014",

"created_by": [redacted],

"searchable_quick": true,

"searchable_advanced": true,

"searchable_batch": false,

"searchable": "Quick,Advanced",

"pii": "Yes",

"qtip": "",

"used_by": "Labmatrix Legacy - Persons Contact, Patient Visit Tools",

"used_by_display": "<a href='#' onClick='remoteJS({name:\"dialogs/AddSubjectTypeDlg\", obj_id:95 });'>Labmatrix Legacy - Persons Contact</a>, <a href='#' onClick='remoteJS({name:\"dialogs/AddSubjectTypeDlg\", obj_id:163 });'>Patient Visit Tools</a>"

},

{

"id": 2703,

"obj_id": 2703,

"display_name": "Address of New Collaborator",

"name": "Address of New Collaborator",

"type": "Text Field",

"values": "",

"created_at": "06/27/2014",

"updated_at": "11/03/2014",

"created_by": [redacted],

"searchable_quick": true,

"searchable_advanced": true,

"searchable_batch": false,

"searchable": "Quick,Advanced",

"pii": "No",

"qtip": "",

"used_by": "Collaboration Projects",

"used_by_display": "<a href='#' onClick='remoteJS({name:\"dialogs/AddSubjectTypeDlg\", obj_id:204 });'>Collaboration Projects</a>"

},

{

"id": 479,

"obj_id": 479,

"display_name": "Address - State/Province",

"name": "Address - State/Province",

"type": "Text Field",

"values": "",

"created_at": "05/07/2013",

"updated_at": "11/03/2014",

"created_by": [redacted],

"searchable_quick": true,

"searchable_advanced": true,

"searchable_batch": false,

"searchable": "Quick,Advanced",

"pii": "Yes",

"qtip": "",

"used_by": "Labmatrix Legacy - Persons Contact, Patient Visit Tools",

"used_by_display": "<a href='#' onClick='remoteJS({name:\"dialogs/AddSubjectTypeDlg\", obj_id:95 });'>Labmatrix Legacy - Persons Contact</a>, <a href='#' onClick='remoteJS({name:\"dialogs/AddSubjectTypeDlg\", obj_id:163 });'>Patient Visit Tools</a>"

},

{

"id": 480,

"obj_id": 480,

"display_name": "Address - Street Address",

"name": "Address - Street Address",

"type": "Text Field",

"values": "",

"created_at": "05/07/2013",

"updated_at": "11/03/2014",

"created_by": [redacted],

"searchable_quick": true,

"searchable_advanced": true,

"searchable_batch": false,

"searchable": "Quick,Advanced",

"pii": "Yes",

"qtip": "",

"used_by": "Labmatrix Legacy - Persons Contact, Patient Visit Tools",

"used_by_display": "<a href='#' onClick='remoteJS({name:\"dialogs/AddSubjectTypeDlg\", obj_id:95 });'>Labmatrix Legacy - Persons Contact</a>, <a href='#' onClick='remoteJS({name:\"dialogs/AddSubjectTypeDlg\", obj_id:163 });'>Patient Visit Tools</a>"

},

{

"id": 2117,

"obj_id": 2117,

"display_name": "Address Type",

"name": "Address Type",

"type": "Text Field",

"values": "",

"created_at": "12/18/2013",

"updated_at": "11/03/2014",

"created_by": [redacted],

"searchable_quick": true,

"searchable_advanced": true,

"searchable_batch": false,

"searchable": "Quick,Advanced",

"pii": "Yes",

"qtip": "",

"used_by": "Patient, Patient Follow Up Visit",

"used_by_display": "<a href='#' onClick='remoteJS({name:\"dialogs/AddSubjectTypeDlg\", obj_id:1 });'>Patient</a>, <a href='#' onClick='remoteJS({name:\"dialogs/AddSubjectTypeDlg\", obj_id:264 });'>Patient Follow Up Visit</a>"

},

{

"id": 483,

"obj_id": 483,

"display_name": "Address - Type",

"name": "Address - Type",

"type": "Text Field",

"values": "",

"created_at": "05/07/2013",

"updated_at": "11/03/2014",

"created_by": [redacted],

"searchable_quick": true,

"searchable_advanced": true,

"searchable_batch": false,

"searchable": "Quick,Advanced",

"pii": "Yes",

"qtip": "",

"used_by": "Labmatrix Legacy - Persons Contact",

"used_by_display": "<a href='#' onClick='remoteJS({name:\"dialogs/AddSubjectTypeDlg\", obj_id:95 });'>Labmatrix Legacy - Persons Contact</a>"

},

{

"id": 481,

"obj_id": 481,

"display_name": "Address - Zip/Postal Code",

"name": "Address - Zip/Postal Code",

"type": "Text Field",

"values": "",

"created_at": "05/07/2013",

"updated_at": "11/03/2014",

"created_by": [redacted],

"searchable_quick": true,

"searchable_advanced": true,

"searchable_batch": false,

"searchable": "Quick,Advanced",

"pii": "Yes",

"qtip": "",

"used_by": "Labmatrix Legacy - Persons Contact",

"used_by_display": "<a href='#' onClick='remoteJS({name:\"dialogs/AddSubjectTypeDlg\", obj_id:95 });'>Labmatrix Legacy - Persons Contact</a>"

},

{

"id": 482,

"obj_id": 482,

"display_name": "Address - Zip/Postal Code Ext.",

"name": "Address - Zip/Postal Code Ext.",

"type": "Text Field",

"values": "",

"created_at": "05/07/2013",

"updated_at": "11/03/2014",

"created_by": [redacted],

"searchable_quick": true,

"searchable_advanced": true,

"searchable_batch": false,

"searchable": "Quick,Advanced",

"pii": "Yes",

"qtip": "",

"used_by": "Labmatrix Legacy - Persons Contact",

"used_by_display": "<a href='#' onClick='remoteJS({name:\"dialogs/AddSubjectTypeDlg\", obj_id:95 });'>Labmatrix Legacy - Persons Contact</a>"

},

{

"id": 940,

"obj_id": 940,

"display_name": "Admit Physician",

"name": "Admit Physician",

"type": "Text Field",

"values": "",

"created_at": "05/10/2013",

"updated_at": "11/03/2014",

"created_by": [redacted],

"searchable_quick": true,

"searchable_advanced": true,

"searchable_batch": false,

"searchable": "Quick,Advanced",

"pii": "No",

"qtip": "",

"used_by": "Sharepoint Legacy - Adult Patient Status",

"used_by_display": "<a href='#' onClick='remoteJS({name:\"dialogs/AddSubjectTypeDlg\", obj_id:115 });'>Sharepoint Legacy - Adult Patient Status</a>"

},

{

"id": 484,

"obj_id": 484,

"display_name": "Adoption Status",

"name": "Adoption Status",

"type": "Text Field",

"values": "",

"created_at": "05/07/2013",

"updated_at": "11/03/2014",

"created_by": [redacted],

"searchable_quick": true,

"searchable_advanced": true,

"searchable_batch": false,

"searchable": "Quick,Advanced",

"pii": "Yes",

"qtip": "",

"used_by": "Patient, Labmatrix Legacy - Subjects, Patient Follow Up Visit",

"used_by_display": "<a href='#' onClick='remoteJS({name:\"dialogs/AddSubjectTypeDlg\", obj_id:1 });'>Patient</a>, <a href='#' onClick='remoteJS({name:\"dialogs/AddSubjectTypeDlg\", obj_id:112 });'>Labmatrix Legacy - Subjects</a>, <a href='#' onClick='remoteJS({name:\"dialogs/AddSubjectTypeDlg\", obj_id:264 });'>Patient Follow Up Visit</a>"

},

{

"id": 328,

"obj_id": 328,

"display_name": "AD Score",

"name": "AD Score",

"type": "Choice",

"values": "1, 2, 3, 4, 5",

"created_at": "04/08/2013",

"updated_at": "11/03/2014",

"created_by": [redacted],

"searchable_quick": true,

"searchable_advanced": true,

"searchable_batch": false,

"searchable": "Quick,Advanced",

"pii": "No",

"qtip": "",

"used_by": "Friday Meeting Notes, Sharepoint Legacy - Friday Meeting Notes",

"used_by_display": "<a href='#' onClick='remoteJS({name:\"dialogs/AddSubjectTypeDlg\", obj_id:82 });'>Friday Meeting Notes</a>, <a href='#' onClick='remoteJS({name:\"dialogs/AddSubjectTypeDlg\", obj_id:154 });'>Sharepoint Legacy - Friday Meeting Notes</a>"

},

{

"id": 1808,

"obj_id": 1808,

"display_name": "Adult UDP Entry - Labmatrix Legacy",

"name": "Adult UDP Entry - Labmatrix Legacy",

"type": "Subject Back Ref",

"values": "",

"created_at": "10/29/2013",

"updated_at": "11/03/2014",

"created_by": [redacted],

"searchable_quick": true,

"searchable_advanced": true,

"searchable_batch": false,

"searchable": "Quick,Advanced",

"pii": "No",

"qtip": "",

"used_by": "Patient, Patient Follow Up Visit",

"used_by_display": "<a href='#' onClick='remoteJS({name:\"dialogs/AddSubjectTypeDlg\", obj_id:1 });'>Patient</a>, <a href='#' onClick='remoteJS({name:\"dialogs/AddSubjectTypeDlg\", obj_id:264 });'>Patient Follow Up Visit</a>"

},

{

"id": 11,

"obj_id": 11,

"display_name": "Affected_status",

"name": "Affected_status",

"type": "Choice",

"values": "Affected, Unaffected, Unknown, Possibly Affected, Not Assessed",

"created_at": "01/28/2013",

"updated_at": "12/16/2014",

"created_by": [redacted],

"searchable_quick": false,

"searchable_advanced": true,

"searchable_batch": false,

"searchable": "Advanced",

"pii": "No",

"qtip": "",

"used_by": "Labmatrix Legacy - SNP Chip Non UDP, Labmatrix Legacy - SNP Chip, Patient Follow Up Visit, UDP Inquiry, Patient",

"used_by_display": "<a href='#' onClick='remoteJS({name:\"dialogs/AddSubjectTypeDlg\", obj_id:99 });'>Labmatrix Legacy - SNP Chip Non UDP</a>, <a href='#' onClick='remoteJS({name:\"dialogs/AddSubjectTypeDlg\", obj_id:105 });'>Labmatrix Legacy - SNP Chip</a>, <a href='#' onClick='remoteJS({name:\"dialogs/AddSubjectTypeDlg\", obj_id:264 });'>Patient Follow Up Visit</a>, <a href='#' onClick='remoteJS({name:\"dialogs/AddSubjectTypeDlg\", obj_id:61 });'>UDP Inquiry</a>, <a href='#' onClick='remoteJS({name:\"dialogs/AddSubjectTypeDlg\", obj_id:1 });'>Patient</a>"

},

{

"id": 455,

"obj_id": 455,

"display_name": "Agarose Gel Image",

"name": "Agarose Gel Image",

"type": "File",

"values": "",

"created_at": "04/28/2013",

"updated_at": "11/03/2014",

"created_by": [redacted],

"searchable_quick": false,

"searchable_advanced": true,

"searchable_batch": false,

"searchable": "Advanced",

"pii": "No",

"qtip": "Proof of Contamination",

"used_by": "Mutagenized Vectors, Destination Vector Construction, Rescue Cell Culture, SNP Chip Sequencing, SNP Analysis, Cell Culture, Sanger Interpretation, Entry Vector, Collaborations",

"used_by_display": "<a href='#' onClick='remoteJS({name:\"dialogs/AddSubjectTypeDlg\", obj_id:228 });'>Mutagenized Vectors</a>, <a href='#' onClick='remoteJS({name:\"dialogs/AddSubjectTypeDlg\", obj_id:168 });'>Destination Vector Construction</a>, <a href='#' onClick='remoteJS({name:\"dialogs/AddSubjectTypeDlg\", obj_id:247 });'>Rescue Cell Culture</a>, <a href='#' onClick='remoteJS({name:\"dialogs/AddSubjectTypeDlg\", obj_id:203 });'>SNP Chip Sequencing</a>, <a href='#' onClick='remoteJS({name:\"dialogs/AddSubjectTypeDlg\", obj_id:59 });'>SNP Analysis</a>, <a href='#' onClick='remoteJS({name:\"dialogs/AddSubjectTypeDlg\", obj_id:40 });'>Cell Culture</a>, <a href='#' onClick='remoteJS({name:\"dialogs/AddSubjectTypeDlg\", obj_id:22 });'>Sanger Interpretation</a>, <a href='#' onClick='remoteJS({name:\"dialogs/AddSubjectTypeDlg\", obj_id:193 });'>Entry Vector</a>, <a href='#' onClick='remoteJS({name:\"dialogs/AddSubjectTypeDlg\", obj_id:147 });'>Collaborations</a>"

},

{

"id": 1843,

"obj_id": 1843,

"display_name": "Agarose Gel Image after Mycoplasma Treatment",

"name": "Agarose Gel Image after Mycoplasma Treatment",

"type": "File",

"values": "",

"created_at": "11/01/2013",

"updated_at": "11/03/2014",

"created_by": [redacted],

"searchable_quick": false,

"searchable_advanced": true,

"searchable_batch": false,

"searchable": "Advanced",

"pii": "No",

"qtip": "",

"used_by": "Cell Culture, Rescue Cell Culture",

"used_by_display": "<a href='#' onClick='remoteJS({name:\"dialogs/AddSubjectTypeDlg\", obj_id:40 });'>Cell Culture</a>, <a href='#' onClick='remoteJS({name:\"dialogs/AddSubjectTypeDlg\", obj_id:247 });'>Rescue Cell Culture</a>"

},

{

"id": 151,

"obj_id": 151,

"display_name": "Age",

"name": "Age",

"type": "Formula",

"values": "",

"created_at": "02/15/2013",

"updated_at": "11/05/2014",

"created_by": [redacted],

"searchable_quick": false,

"searchable_advanced": true,

"searchable_batch": false,

"searchable": "Advanced",

"pii": "No",

"qtip": null,

"used_by": "Labmatrix Legacy - Peds Sharepoint Seen, Labmatrix Legacy - Peds Sharepoint Seen List, Patient Follow Up Visit, Sharepoint Legacy - Pediatric Patient, Sharepoint Legacy - Adult Patient Status, Patient, UDP Inquiry",

"used_by_display": "<a href='#' onClick='remoteJS({name:\"dialogs/AddSubjectTypeDlg\", obj_id:97 });'>Labmatrix Legacy - Peds Sharepoint Seen</a>, <a href='#' onClick='remoteJS({name:\"dialogs/AddSubjectTypeDlg\", obj_id:98 });'>Labmatrix Legacy - Peds Sharepoint Seen List</a>, <a href='#' onClick='remoteJS({name:\"dialogs/AddSubjectTypeDlg\", obj_id:264 });'>Patient Follow Up Visit</a>, <a href='#' onClick='remoteJS({name:\"dialogs/AddSubjectTypeDlg\", obj_id:114 });'>Sharepoint Legacy - Pediatric Patient</a>, <a href='#' onClick='remoteJS({name:\"dialogs/AddSubjectTypeDlg\", obj_id:115 });'>Sharepoint Legacy - Adult Patient Status</a>, <a href='#' onClick='remoteJS({name:\"dialogs/AddSubjectTypeDlg\", obj_id:1 });'>Patient</a>, <a href='#' onClick='remoteJS({name:\"dialogs/AddSubjectTypeDlg\", obj_id:61 });'>UDP Inquiry</a>"

},

{

"id": 485,

"obj_id": 485,

"display_name": "Age at Death",

"name": "Age at Death",

"type": "Number",

"values": "",

"created_at": "05/07/2013",

"updated_at": "11/03/2014",

"created_by": [redacted],

"searchable_quick": false,

"searchable_advanced": true,

"searchable_batch": false,

"searchable": "Advanced",

"pii": "Yes",

"qtip": "",

"used_by": "Patient, Labmatrix Legacy - Subjects, Patient Follow Up Visit",

"used_by_display": "<a href='#' onClick='remoteJS({name:\"dialogs/AddSubjectTypeDlg\", obj_id:1 });'>Patient</a>, <a href='#' onClick='remoteJS({name:\"dialogs/AddSubjectTypeDlg\", obj_id:112 });'>Labmatrix Legacy - Subjects</a>, <a href='#' onClick='remoteJS({name:\"dialogs/AddSubjectTypeDlg\", obj_id:264 });'>Patient Follow Up Visit</a>"

},

{

"id": 909,

"obj_id": 909,

"display_name": "Age in Days",

"name": "Age in Days",

"type": "Text Field",

"values": "",

"created_at": "05/10/2013",

"updated_at": "11/03/2014",

"created_by": [redacted],

"searchable_quick": false,

"searchable_advanced": true,

"searchable_batch": false,

"searchable": "Advanced",

"pii": "Yes",

"qtip": "",

"used_by": "Sharepoint Legacy - Pediatric Patient, Sharepoint Legacy - Adult Patient Status",

"used_by_display": "<a href='#' onClick='remoteJS({name:\"dialogs/AddSubjectTypeDlg\", obj_id:114 });'>Sharepoint Legacy - Pediatric Patient</a>, <a href='#' onClick='remoteJS({name:\"dialogs/AddSubjectTypeDlg\", obj_id:115 });'>Sharepoint Legacy - Adult Patient Status</a>"

},

{

"id": 1794,

"obj_id": 1794,

"display_name": "Age in Labmatrix",

"name": "Age in Labmatrix",

"type": "Text Field",

"values": "",

"created_at": "10/25/2013",

"updated_at": "11/03/2014",

"created_by": [redacted],

"searchable_quick": false,

"searchable_advanced": true,

"searchable_batch": false,

"searchable": "Advanced",

"pii": "No",

"qtip": "",

"used_by": "Labmatrix Legacy - Subjects",

"used_by_display": "<a href='#' onClick='remoteJS({name:\"dialogs/AddSubjectTypeDlg\", obj_id:112 });'>Labmatrix Legacy - Subjects</a>"

},

{

"id": 487,

"obj_id": 487,

"display_name": "Age in Years",

"name": "Age in Years",

"type": "Number",

"values": "",

"created_at": "05/07/2013",

"updated_at": "11/03/2014",

"created_by": [redacted],

"searchable_quick": false,

"searchable_advanced": true,

"searchable_batch": false,

"searchable": "Advanced",

"pii": "Yes",

"qtip": "",

"used_by": "Labmatrix Legacy - Peds Sharepoint Seen, Labmatrix Legacy - Peds Sharepoint Seen List, Sharepoint Legacy - Pediatric Patient",

"used_by_display": "<a href='#' onClick='remoteJS({name:\"dialogs/AddSubjectTypeDlg\", obj_id:97 });'>Labmatrix Legacy - Peds Sharepoint Seen</a>, <a href='#' onClick='remoteJS({name:\"dialogs/AddSubjectTypeDlg\", obj_id:98 });'>Labmatrix Legacy - Peds Sharepoint Seen List</a>, <a href='#' onClick='remoteJS({name:\"dialogs/AddSubjectTypeDlg\", obj_id:114 });'>Sharepoint Legacy - Pediatric Patient</a>"

},

{

"id": 2204,

"obj_id": 2204,

"display_name": "Age of Penetrance",

"name": "Age of Penetrance",

"type": "Choice",

"values": "Unknown, Congenital onset, Embryonal onset, Fetal onset, Neonatal onset, Infantile onset, Childhood onset, Juvenile onset, Adult onset, Young adult onset, Middle age onset, Late onset",

"created_at": "02/04/2014",

"updated_at": "11/03/2014",

"created_by": [redacted],

"searchable_quick": true,

"searchable_advanced": true,

"searchable_batch": true,

"searchable": "Quick,Advanced,Batch",

"pii": "Yes",

"qtip": "Global age of onset from Phenotips",

"used_by": "Patient, Phenotype, Patient Follow Up Visit",

"used_by_display": "<a href='#' onClick='remoteJS({name:\"dialogs/AddSubjectTypeDlg\", obj_id:1 });'>Patient</a>, <a href='#' onClick='remoteJS({name:\"dialogs/AddSubjectTypeDlg\", obj_id:160 });'>Phenotype</a>, <a href='#' onClick='remoteJS({name:\"dialogs/AddSubjectTypeDlg\", obj_id:264 });'>Patient Follow Up Visit</a>"

},

{

"id": 2023,

"obj_id": 2023,

"display_name": "Alamut Analysis",

"name": "Alamut Analysis",

"type": "File",

"values": "",

"created_at": "11/25/2013",

"updated_at": "11/03/2014",

"created_by": [redacted],

"searchable_quick": true,

"searchable_advanced": true,

"searchable_batch": true,

"searchable": "Quick,Advanced,Batch",

"pii": "No",

"qtip": "Image of alleles in Alamut",

"used_by": "Sanger Interpretation, Exome Analysis Results",

"used_by_display": "<a href='#' onClick='remoteJS({name:\"dialogs/AddSubjectTypeDlg\", obj_id:22 });'>Sanger Interpretation</a>, <a href='#' onClick='remoteJS({name:\"dialogs/AddSubjectTypeDlg\", obj_id:156 });'>Exome Analysis Results</a>"

},

{

"id": 995,

"obj_id": 995,

"display_name": "Alamut-HT Annotations Added",

"name": "Alamut-HT Annotations Added",

"type": "CheckBox",

"values": "",

"created_at": "05/10/2013",

"updated_at": "11/03/2014",

"created_by": [redacted],

"searchable_quick": true,

"searchable_advanced": true,

"searchable_batch": true,

"searchable": "Quick,Advanced,Batch",

"pii": "No",

"qtip": "AlamutAnnotation_afterANNOVAR.py",

"used_by": "Cohort Variant Call Format File",

"used_by_display": "<a href='#' onClick='remoteJS({name:\"dialogs/AddSubjectTypeDlg\", obj_id:195 });'>Cohort Variant Call Format File</a>"

},

{

"id": 488,

"obj_id": 488,

"display_name": "Alias_sample_ID",

"name": "Alias_sample_ID",

"type": "Text Field",

"values": "",

"created_at": "05/07/2013",

"updated_at": "09/19/2013",

"created_by": [redacted],

"searchable_quick": false,

"searchable_advanced": null,

"searchable_batch": null,

"searchable": "",

"pii": "No",

"qtip": "",

"used_by": "Labmatrix Legacy - SNP Chip Non UDP, Labmatrix Legacy - SNP Chip",

"used_by_display": "<a href='#' onClick='remoteJS({name:\"dialogs/AddSubjectTypeDlg\", obj_id:99 });'>Labmatrix Legacy - SNP Chip Non UDP</a>, <a href='#' onClick='remoteJS({name:\"dialogs/AddSubjectTypeDlg\", obj_id:105 });'>Labmatrix Legacy - SNP Chip</a>"

},

{

"id": 2237,

"obj_id": 2237,

"display_name": "Aligned VCF File",

"name": "Aligned VCF File",

"type": "File",

"values": "",

"created_at": "02/07/2014",

"updated_at": "11/04/2014",

"created_by": [redacted],

"searchable_quick": false,

"searchable_advanced": true,

"searchable_batch": false,

"searchable": "Advanced",

"pii": "No",

"qtip": "",

"used_by": "Diploid Alignment",

"used_by_display": "<a href='#' onClick='remoteJS({name:\"dialogs/AddSubjectTypeDlg\", obj_id:209 });'>Diploid Alignment</a>"

},

{

"id": 2927,

"obj_id": 2927,

"display_name": "Aligner Used",

"name": "Aligner Used",

"type": "Choice",

"values": "Bowtie, BWA, NovoAlign, STAR, TopHat",

"created_at": "12/08/2014",

"updated_at": "12/08/2014",

"created_by": [redacted],

"searchable_quick": false,

"searchable_advanced": true,

"searchable_batch": false,

"searchable": "Advanced",

"pii": "No",

"qtip": "",

"used_by": "ChIP-Seq, Transcriptome",

"used_by_display": "<a href='#' onClick='remoteJS({name:\"dialogs/AddSubjectTypeDlg\", obj_id:254 });'>ChIP-Seq</a>, <a href='#' onClick='remoteJS({name:\"dialogs/AddSubjectTypeDlg\", obj_id:255 });'>Transcriptome</a>"

},

{

"id": 2176,

"obj_id": 2176,

"display_name": "Align GVGD",

"name": "Align GVGD",

"type": "Text Field",

"values": "",

"created_at": "01/17/2014",

"updated_at": "11/04/2014",

"created_by": [redacted],

"searchable_quick": false,

"searchable_advanced": true,

"searchable_batch": false,

"searchable": "Advanced",

"pii": "No",

"qtip": "",

"used_by": "Exome Analysis Results",

"used_by_display": "<a href='#' onClick='remoteJS({name:\"dialogs/AddSubjectTypeDlg\", obj_id:156 });'>Exome Analysis Results</a>"

},

{

"id": 2177,

"obj_id": 2177,

"display_name": "Align GVGD Numbers",

"name": "Align GVGD Numbers",

"type": "Text Field",

"values": "",

"created_at": "01/17/2014",

"updated_at": "11/04/2014",

"created_by": [redacted],

"searchable_quick": false,

"searchable_advanced": true,

"searchable_batch": false,

"searchable": "Advanced",

"pii": "No",

"qtip": "",

"used_by": "Exome Analysis Results",

"used_by_display": "<a href='#' onClick='remoteJS({name:\"dialogs/AddSubjectTypeDlg\", obj_id:156 });'>Exome Analysis Results</a>"

},

{

"id": 2528,

"obj_id": 2528,

"display_name": "Aligning Center",

"name": "Aligning Center",

"type": "Text Field",

"values": "",

"created_at": "04/15/2014",

"updated_at": "11/04/2014",

"created_by": [redacted],

"searchable_quick": false,

"searchable_advanced": true,

"searchable_batch": false,

"searchable": "Advanced",

"pii": "No",

"qtip": "",

"used_by": "Patient Bioinformatics Files",

"used_by_display": "<a href='#' onClick='remoteJS({name:\"dialogs/AddSubjectTypeDlg\", obj_id:235 });'>Patient Bioinformatics Files</a>"

},

{

"id": 2454,

"obj_id": 2454,

"display_name": "Alignment",

"name": "Alignment",

"type": "Text Field",

"values": "",

"created_at": "03/25/2014",

"updated_at": "11/04/2014",

"created_by": [redacted],

"searchable_quick": false,

"searchable_advanced": true,

"searchable_batch": false,

"searchable": "Advanced",

"pii": "No",

"qtip": "",

"used_by": "dbGaP VCFs",

"used_by_display": "<a href='#' onClick='remoteJS({name:\"dialogs/AddSubjectTypeDlg\", obj_id:207 });'>dbGaP VCFs</a>"

},

{

"id": 2031,

"obj_id": 2031,

"display_name": "Alignment Collaborator Files",

"name": "Alignment Collaborator Files",

"type": "Subject Back Ref",

"values": "",

"created_at": "11/26/2013",

"updated_at": "11/04/2014",

"created_by": [redacted],

"searchable_quick": false,

"searchable_advanced": true,

"searchable_batch": false,

"searchable": "Advanced",

"pii": "No",

"qtip": "",

"used_by": "Information Technology Collaboration, Diploid Alignment",

"used_by_display": "<a href='#' onClick='remoteJS({name:\"dialogs/AddSubjectTypeDlg\", obj_id:183 });'>Information Technology Collaboration</a>, <a href='#' onClick='remoteJS({name:\"dialogs/AddSubjectTypeDlg\", obj_id:209 });'>Diploid Alignment</a>"

},

{

"id": 2315,

"obj_id": 2315,

"display_name": "Alignment Information",

"name": "Alignment Information",

"type": "Subject Back Ref",

"values": "",

"created_at": "03/04/2014",

"updated_at": "11/04/2014",

"created_by": [redacted],

"searchable_quick": false,

"searchable_advanced": true,

"searchable_batch": false,

"searchable": "Advanced",

"pii": "No",

"qtip": "",

"used_by": "Patient, Family, Patient Follow Up Visit",

"used_by_display": "<a href='#' onClick='remoteJS({name:\"dialogs/AddSubjectTypeDlg\", obj_id:1 });'>Patient</a>, <a href='#' onClick='remoteJS({name:\"dialogs/AddSubjectTypeDlg\", obj_id:2 });'>Family</a>, <a href='#' onClick='remoteJS({name:\"dialogs/AddSubjectTypeDlg\", obj_id:264 });'>Patient Follow Up Visit</a>"

},

{

"id": 1094,

"obj_id": 1094,

"display_name": "Alignment Location",

"name": "Alignment Location",

"type": "Choice",

"values": "UDP, Toronto, Appistry, NISC, Axeq, NY Genome",

"created_at": "05/17/2013",

"updated_at": "11/04/2014",

"created_by": [redacted],

"searchable_quick": false,

"searchable_advanced": true,

"searchable_batch": false,

"searchable": "Advanced",

"pii": "No",

"qtip": "",

"used_by": "Cohort Variant Call Format File, Diploid Alignment, Genome Sequencing",

"used_by_display": "<a href='#' onClick='remoteJS({name:\"dialogs/AddSubjectTypeDlg\", obj_id:195 });'>Cohort Variant Call Format File</a>, <a href='#' onClick='remoteJS({name:\"dialogs/AddSubjectTypeDlg\", obj_id:209 });'>Diploid Alignment</a>, <a href='#' onClick='remoteJS({name:\"dialogs/AddSubjectTypeDlg\", obj_id:212 });'>Genome Sequencing</a>"

},

{

"id": 1903,

"obj_id": 1903,

"display_name": "Alignment Notes",

"name": "Alignment Notes",

"type": "Text Area",

"values": "",

"created_at": "11/08/2013",

"updated_at": "11/04/2014",

"created_by": [redacted],

"searchable_quick": false,

"searchable_advanced": false,

"searchable_batch": false,

"searchable": "",

"pii": "No",

"qtip": "",

"used_by": "Diploid Alignment",

"used_by_display": "<a href='#' onClick='remoteJS({name:\"dialogs/AddSubjectTypeDlg\", obj_id:209 });'>Diploid Alignment</a>"

},

{

"id": 2529,

"obj_id": 2529,

"display_name": "Alignment Type",

"name": "Alignment Type",

"type": "Text Field",

"values": "",

"created_at": "04/15/2014",

"updated_at": "11/04/2014",

"created_by": [redacted],

"searchable_quick": false,

"searchable_advanced": true,

"searchable_batch": false,

"searchable": "Advanced",

"pii": "No",

"qtip": "",

"used_by": "Patient Bioinformatics Files",

"used_by_display": "<a href='#' onClick='remoteJS({name:\"dialogs/AddSubjectTypeDlg\", obj_id:235 });'>Patient Bioinformatics Files</a>"

},

{

"id": 2614,

"obj_id": 2614,

"display_name": "Allele Type",

"name": "Allele Type",

"type": "Choice",

"values": "Monoallelic, Biallelic, Hemizygous, Biallelic/Monoallelic, Unknown",

"created_at": "05/12/2014",

"updated_at": "11/04/2014",

"created_by": [redacted],

"searchable_quick": false,

"searchable_advanced": true,

"searchable_batch": false,

"searchable": "Advanced",

"pii": "No",

"qtip": "",

"used_by": "Exome Analysis Results",

"used_by_display": "<a href='#' onClick='remoteJS({name:\"dialogs/AddSubjectTypeDlg\", obj_id:156 });'>Exome Analysis Results</a>"

},

{

"id": 2325,

"obj_id": 2325,

"display_name": "Allergies:",

"name": "Allergies:",

"type": "Text Area",

"values": "",

"created_at": "03/04/2014",

"updated_at": "11/04/2014",

"created_by": [redacted],

"searchable_quick": false,

"searchable_advanced": false,

"searchable_batch": false,

"searchable": "",

"pii": "Yes",

"qtip": "",

"used_by": "Patient Review",

"used_by_display": "<a href='#' onClick='remoteJS({name:\"dialogs/AddSubjectTypeDlg\", obj_id:83 });'>Patient Review</a>"

},

{

"id": 982,

"obj_id": 982,

"display_name": "All Families File Used",

"name": "All Families File Used",

"type": "Text Field",

"values": "",

"created_at": "05/10/2013",

"updated_at": "11/04/2014",

"created_by": [redacted],

"searchable_quick": false,

"searchable_advanced": true,

"searchable_batch": false,

"searchable": "Advanced",

"pii": "Yes",

"qtip": "Starting all families file to update",

"used_by": "Varsifter Files",

"used_by_display": "<a href='#' onClick='remoteJS({name:\"dialogs/AddSubjectTypeDlg\", obj_id:198 });'>Varsifter Files</a>"

},

{

"id": 2019,

"obj_id": 2019,

"display_name": "All_freq_refallele",

"name": "All_freq_refallele",

"type": "Number",

"values": "",

"created_at": "11/22/2013",

"updated_at": "11/04/2014",

"created_by": [redacted],

"searchable_quick": false,

"searchable_advanced": false,

"searchable_batch": false,

"searchable": "",

"pii": "No",

"qtip": "",

"used_by": "Exome Analysis Results",

"used_by_display": "<a href='#' onClick='remoteJS({name:\"dialogs/AddSubjectTypeDlg\", obj_id:156 });'>Exome Analysis Results</a>"

},

{

"id": 996,

"obj_id": 996,

"display_name": "Frequencies Recalculated - DELETE",

"name": "Frequencies Recalculated - DELETE",

"type": "CheckBox",

"values": "",

"created_at": "05/10/2013",

"updated_at": "06/06/2014",

"created_by": [redacted],

"searchable_quick": false,

"searchable_advanced": null,

"searchable_batch": null,

"searchable": "",

"pii": "No",

"qtip": "RedoCounts_all.py",

"used_by": "",

"used_by_display": ""

},

{

"id": 2020,

"obj_id": 2020,

"display_name": "All_freq_varallele",

"name": "All_freq_varallele",

"type": "Number",

"values": "",

"created_at": "11/22/2013",

"updated_at": "11/22/2013",

"created_by": [redacted],

"searchable_quick": false,

"searchable_advanced": null,

"searchable_batch": null,

"searchable": "",

"pii": "No",

"qtip": "",

"used_by": "Exome Analysis Results",

"used_by_display": "<a href='#' onClick='remoteJS({name:\"dialogs/AddSubjectTypeDlg\", obj_id:156 });'>Exome Analysis Results</a>"

},

{

"id": 2540,

"obj_id": 2540,

"display_name": "All_MAF",

"name": "All_MAF",

"type": "Text Field",

"values": "",

"created_at": "04/21/2014",

"updated_at": "12/10/2014",

"created_by": [redacted],

"searchable_quick": false,

"searchable_advanced": false,

"searchable_batch": false,

"searchable": "",

"pii": "No",

"qtip": "",

"used_by": "Exome Analysis Results",

"used_by_display": "<a href='#' onClick='remoteJS({name:\"dialogs/AddSubjectTypeDlg\", obj_id:156 });'>Exome Analysis Results</a>"

},

{

"id": 2599,

"obj_id": 2599,

"display_name": "Alternate Allele",

"name": "Alternate Allele",

"type": "Text Field",

"values": "",

"created_at": "05/08/2014",

"updated_at": "12/11/2014",

"created_by": [redacted],

"searchable_quick": false,

"searchable_advanced": true,

"searchable_batch": false,

"searchable": "Advanced",

"pii": "No",

"qtip": "",

"used_by": "",

"used_by_display": ""

},

{

"id": 2694,

"obj_id": 2694,

"display_name": "Amendment Documentation",

"name": "Amendment Documentation",

"type": "File",

"values": "",

"created_at": "06/25/2014",

"updated_at": "11/04/2014",

"created_by": [redacted],

"searchable_quick": false,

"searchable_advanced": true,

"searchable_batch": false,

"searchable": "Advanced",

"pii": "No",

"qtip": "",

"used_by": "MTA/ITA Amendments",

"used_by_display": "<a href='#' onClick='remoteJS({name:\"dialogs/AddSubjectTypeDlg\", obj_id:251 });'>MTA/ITA Amendments</a>"

},

{

"id": 2698,

"obj_id": 2698,

"display_name": "Amendment TEST",

"name": "Amendment TEST",

"type": "Subject",

"values": "",

"created_at": "06/25/2014",

"updated_at": "06/25/2014",

"created_by": [redacted],

"searchable_quick": false,

"searchable_advanced": null,

"searchable_batch": null,

"searchable": "",

"pii": "No",

"qtip": "",

"used_by": "",

"used_by_display": ""

},

{

"id": 2677,

"obj_id": 2677,

"display_name": "Amendment to Agreement",

"name": "Amendment to Agreement",

"type": "File",

"values": "",

"created_at": "06/16/2014",

"updated_at": "11/04/2014",

"created_by": [redacted],

"searchable_quick": false,

"searchable_advanced": true,

"searchable_batch": false,

"searchable": "Advanced",

"pii": "No",

"qtip": "",

"used_by": "Collaboration Projects",

"used_by_display": "<a href='#' onClick='remoteJS({name:\"dialogs/AddSubjectTypeDlg\", obj_id:204 });'>Collaboration Projects</a>"

},

{

"id": 2183,

"obj_id": 2183,

"display_name": "Amino Acid Change",

"name": "Amino Acid Change",

"type": "Text Field",

"values": "",

"created_at": "01/22/2014",

"updated_at": "11/04/2014",

"created_by": [redacted],

"searchable_quick": false,

"searchable_advanced": true,

"searchable_batch": false,

"searchable": "Advanced",

"pii": "No",

"qtip": "Protein level (p.AA#AA)",

"used_by": "Exome Analysis Results",

"used_by_display": "<a href='#' onClick='remoteJS({name:\"dialogs/AddSubjectTypeDlg\", obj_id:156 });'>Exome Analysis Results</a>"

},

{

"id": 2109,

"obj_id": 2109,

"display_name": "Amino Acid Designation",

"name": "Amino Acid Designation",

"type": "Text Area",

"values": "",

"created_at": "12/17/2013",

"updated_at": "11/04/2014",

"created_by": [redacted],

"searchable_quick": false,

"searchable_advanced": false,

"searchable_batch": false,

"searchable": "",

"pii": "No",

"qtip": "",

"used_by": "Zebrafish Mutation Project",

"used_by_display": "<a href='#' onClick='remoteJS({name:\"dialogs/AddSubjectTypeDlg\", obj_id:186 });'>Zebrafish Mutation Project</a>"

},

{

"id": 2422,

"obj_id": 2422,

"display_name": "Amino Acid Sequence",

"name": "Amino Acid Sequence",

"type": "Text Area",

"values": "",

"created_at": "03/20/2014",

"updated_at": "11/04/2014",

"created_by": [redacted],

"searchable_quick": false,

"searchable_advanced": false,

"searchable_batch": false,

"searchable": "",

"pii": "No",

"qtip": "",

"used_by": "Zebrafish Mutation Project",

"used_by_display": "<a href='#' onClick='remoteJS({name:\"dialogs/AddSubjectTypeDlg\", obj_id:186 });'>Zebrafish Mutation Project</a>"

},

{

"id": 1978,

"obj_id": 1978,

"display_name": "Amount of DNA (ug)",

"name": "Amount of DNA (ug)",

"type": "Formula",

"values": "",

"created_at": "11/19/2013",

"updated_at": "02/14/2014",

"created_by": [redacted],

"searchable_quick": false,

"searchable_advanced": null,

"searchable_batch": null,

"searchable": "",

"pii": "No",

"qtip": null,

"used_by": "DNA Extraction",

"used_by_display": "<a href='#' onClick='remoteJS({name:\"dialogs/AddSubjectTypeDlg\", obj_id:122 });'>DNA Extraction</a>"

},

{

"id": 2024,

"obj_id": 2024,

"display_name": "Amount of DNA(ug)",

"name": "Amount of DNA(ug)",

"type": "Text Field",

"values": "",

"created_at": "11/25/2013",

"updated_at": "11/04/2014",

"created_by": [redacted],

"searchable_quick": false,

"searchable_advanced": true,

"searchable_batch": false,

"searchable": "Advanced",

"pii": "No",

"qtip": "For CLIA validation",

"used_by": "CLIA Validation",

"used_by_display": "<a href='#' onClick='remoteJS({name:\"dialogs/AddSubjectTypeDlg\", obj_id:15 });'>CLIA Validation</a>"

},

{

"id": 2777,

"obj_id": 2777,

"display_name": "Amount of DNA Used for Amplification",

"name": "Amount of DNA Used for Amplification",

"type": "Text Field",

"values": "",

"created_at": "08/04/2014",

"updated_at": "08/04/2014",

"created_by": [redacted],

"searchable_quick": false,

"searchable_advanced": false,

"searchable_batch": false,

"searchable": "",

"pii": "No",

"qtip": "",

"used_by": "Genome Amplification",

"used_by_display": "<a href='#' onClick='remoteJS({name:\"dialogs/AddSubjectTypeDlg\", obj_id:258 });'>Genome Amplification</a>"

},

{

"id": 2794,

"obj_id": 2794,

"display_name": "Amount of Plasma/Serum for Amplification",

"name": "Amount of Plasma/Serum for Amplification",

"type": "Text Field",

"values": "",

"created_at": "08/28/2014",

"updated_at": "08/28/2014",

"created_by": [redacted],

"searchable_quick": false,

"searchable_advanced": false,

"searchable_batch": false,

"searchable": "",

"pii": "No",

"qtip": "",

"used_by": "Genome Amplification",

"used_by_display": "<a href='#' onClick='remoteJS({name:\"dialogs/AddSubjectTypeDlg\", obj_id:258 });'>Genome Amplification</a>"

},

{

"id": 2425,

"obj_id": 2425,

"display_name": "Amplification Gel Image",

"name": "Amplification Gel Image",

"type": "File",

"values": "",

"created_at": "03/20/2014",

"updated_at": "03/20/2014",

"created_by": [redacted],

"searchable_quick": false,

"searchable_advanced": null,

"searchable_batch": null,

"searchable": "",

"pii": "No",

"qtip": "",

"used_by": "Zebrafish Mutation Project, Genome Amplification",

"used_by_display": "<a href='#' onClick='remoteJS({name:\"dialogs/AddSubjectTypeDlg\", obj_id:186 });'>Zebrafish Mutation Project</a>, <a href='#' onClick='remoteJS({name:\"dialogs/AddSubjectTypeDlg\", obj_id:258 });'>Genome Amplification</a>"

},

{

"id": 2042,

"obj_id": 2042,

"display_name": "Analysis",

"name": "Analysis",

"type": "Choice",

"values": "Basic, Standard, Advanced, Premium",

"created_at": "12/02/2013",

"updated_at": "12/14/2013",

"created_by": [redacted],

"searchable_quick": false,

"searchable_advanced": null,

"searchable_batch": null,

"searchable": "",

"pii": "No",

"qtip": "Axeq Submission Form",

"used_by": "Exome Sequencing, Genome Sequencing",

"used_by_display": "<a href='#' onClick='remoteJS({name:\"dialogs/AddSubjectTypeDlg\", obj_id:161 });'>Exome Sequencing</a>, <a href='#' onClick='remoteJS({name:\"dialogs/AddSubjectTypeDlg\", obj_id:212 });'>Genome Sequencing</a>"

},

{

"id": 2792,

"obj_id": 2792,

"display_name": "Analysis Pipeline",

"name": "Analysis Pipeline",

"type": "Text Field",

"values": "",

"created_at": "08/28/2014",

"updated_at": "08/28/2014",

"created_by": [redacted],

"searchable_quick": false,

"searchable_advanced": false,

"searchable_batch": false,

"searchable": "",

"pii": "No",

"qtip": "Method used for analysis",

"used_by": "Exome Analysis",

"used_by_display": "<a href='#' onClick='remoteJS({name:\"dialogs/AddSubjectTypeDlg\", obj_id:60 });'>Exome Analysis</a>"

},

{

"id": 2871,

"obj_id": 2871,

"display_name": "Analysis Report",

"name": "Analysis Report",

"type": "File",

"values": "",

"created_at": "11/03/2014",

"updated_at": "11/03/2014",

"created_by": [redacted],

"searchable_quick": false,

"searchable_advanced": true,

"searchable_batch": false,

"searchable": "Advanced",

"pii": "No",

"qtip": "",

"used_by": "Transcriptome",

"used_by_display": "<a href='#' onClick='remoteJS({name:\"dialogs/AddSubjectTypeDlg\", obj_id:255 });'>Transcriptome</a>"

},

{

"id": 2791,

"obj_id": 2791,

"display_name": "Analysis Status",

"name": "Analysis Status",

"type": "Text Field",

"values": "",

"created_at": "08/25/2014",

"updated_at": "11/13/2014",

"created_by": [redacted],

"searchable_quick": false,

"searchable_advanced": true,

"searchable_batch": false,

"searchable": "Advanced",

"pii": "No",

"qtip": "",

"used_by": "Exome Analysis",

"used_by_display": "<a href='#' onClick='remoteJS({name:\"dialogs/AddSubjectTypeDlg\", obj_id:60 });'>Exome Analysis</a>"

},

{

"id": 1767,

"obj_id": 1767,

"display_name": "Analyst",

"name": "Analyst",

"type": "Text Field",

"values": "",

"created_at": "10/22/2013",

"updated_at": "11/13/2014",

"created_by": [redacted],

"searchable_quick": true,

"searchable_advanced": true,

"searchable_batch": true,

"searchable": "Quick,Advanced,Batch",

"pii": "No",

"qtip": "",

"used_by": "SNP Analysis, Exome Analysis, Independent SNP Analysis",

"used_by_display": "<a href='#' onClick='remoteJS({name:\"dialogs/AddSubjectTypeDlg\", obj_id:59 });'>SNP Analysis</a>, <a href='#' onClick='remoteJS({name:\"dialogs/AddSubjectTypeDlg\", obj_id:60 });'>Exome Analysis</a>, <a href='#' onClick='remoteJS({name:\"dialogs/AddSubjectTypeDlg\", obj_id:215 });'>Independent SNP Analysis</a>"

},

{

"id": 329,

"obj_id": 329,

"display_name": "Analyze Exome for",

"name": "Analyze Exome for",

"type": "List",

"values": "New Dominant, AR Homozygous, AR Compound Het, Gene List, SNP Correlation, X-Linkage",

"created_at": "04/08/2013",

"updated_at": "11/13/2014",

"created_by": [redacted],

"searchable_quick": false,

"searchable_advanced": true,

"searchable_batch": false,

"searchable": "Advanced",

"pii": "No",

"qtip": "",

"used_by": "Friday Meeting Notes",

"used_by_display": "<a href='#' onClick='remoteJS({name:\"dialogs/AddSubjectTypeDlg\", obj_id:82 });'>Friday Meeting Notes</a>"

},

{

"id": 343,

"obj_id": 343,

"display_name": "Analyze SNP for",

"name": "Analyze SNP for",

"type": "List",

"values": "SNP CNV, SNP Parentage, SNP Mosaicism, SNP Linkage Bed, SNP Homozygosity",

"created_at": "04/11/2013",

"updated_at": "11/13/2014",

"created_by": [redacted],

"searchable_quick": false,

"searchable_advanced": true,

"searchable_batch": false,

"searchable": "Advanced",

"pii": "No",

"qtip": "",

"used_by": "Friday Meeting Notes",

"used_by_display": "<a href='#' onClick='remoteJS({name:\"dialogs/AddSubjectTypeDlg\", obj_id:82 });'>Friday Meeting Notes</a>"

},

{

"id": 489,

"obj_id": 489,

"display_name": "Anatomy/Cell Type",

"name": "Anatomy/Cell Type",

"type": "Text Field",

"values": "",

"created_at": "05/07/2013",

"updated_at": "11/13/2014",

"created_by": [redacted],

"searchable_quick": false,

"searchable_advanced": true,

"searchable_batch": false,

"searchable": "Advanced",

"pii": "No",

"qtip": "",

"used_by": "Labmatrix Legacy - Biomaterials",

"used_by_display": "<a href='#' onClick='remoteJS({name:\"dialogs/AddSubjectTypeDlg\", obj_id:91 });'>Labmatrix Legacy - Biomaterials</a>"

},

{

"id": 2050,

"obj_id": 2050,

"display_name": "Annotated Cohort File",

"name": "Annotated Cohort File",

"type": "File",

"values": "",

"created_at": "12/04/2013",

"updated_at": "11/13/2014",

"created_by": [redacted],

"searchable_quick": false,

"searchable_advanced": true,

"searchable_batch": false,

"searchable": "Advanced",

"pii": "No",

"qtip": "",

"used_by": "Cohort Variant Call Format File",

"used_by_display": "<a href='#' onClick='remoteJS({name:\"dialogs/AddSubjectTypeDlg\", obj_id:195 });'>Cohort Variant Call Format File</a>"

},

{

"id": 2054,

"obj_id": 2054,

"display_name": "Annotated Cohort VCF",

"name": "Annotated Cohort VCF",

"type": "Subjects",

"values": "",

"created_at": "12/04/2013",

"updated_at": "11/13/2014",

"created_by": [redacted],

"searchable_quick": false,

"searchable_advanced": true,

"searchable_batch": false,

"searchable": "Advanced",

"pii": "No",

"qtip": "",

"used_by": "Exome Analysis",

"used_by_display": "<a href='#' onClick='remoteJS({name:\"dialogs/AddSubjectTypeDlg\", obj_id:60 });'>Exome Analysis</a>"

},

{

"id": 998,

"obj_id": 998,

"display_name": "Annotated File Merged with All Families File - DELETE",

"name": "Annotated File Merged with All Families File - DELETE",

"type": "CheckBox",

"values": "",

"created_at": "05/10/2013",

"updated_at": "11/13/2014",

"created_by": [redacted],

"searchable_quick": false,

"searchable_advanced": true,

"searchable_batch": false,

"searchable": "Advanced",

"pii": "No",

"qtip": "AddInAnnotatedVS.py",

"used_by": "",

"used_by_display": ""

},

{

"id": 1485,

"obj_id": 1485,

"display_name": "Annotations",

"name": "Annotations",

"type": "Text Area",

"values": "",

"created_at": "07/22/2013",

"updated_at": "11/13/2014",

"created_by": [redacted],

"searchable_quick": false,

"searchable_advanced": false,

"searchable_batch": false,

"searchable": "",

"pii": "No",

"qtip": "",

"used_by": "Exome Analysis Results",

"used_by_display": "<a href='#' onClick='remoteJS({name:\"dialogs/AddSubjectTypeDlg\", obj_id:156 });'>Exome Analysis Results</a>"

},

{

"id": 994,

"obj_id": 994,

"display_name": "ANNOVAR Annotations Added",

"name": "ANNOVAR Annotations Added",

"type": "CheckBox",

"values": "",

"created_at": "05/10/2013",

"updated_at": "11/13/2014",

"created_by": [redacted],

"searchable_quick": false,

"searchable_advanced": true,

"searchable_batch": false,

"searchable": "Advanced",

"pii": "No",

"qtip": "ANNOVARAnnotation.py",

"used_by": "Cohort Variant Call Format File",

"used_by_display": "<a href='#' onClick='remoteJS({name:\"dialogs/AddSubjectTypeDlg\", obj_id:195 });'>Cohort Variant Call Format File</a>"

},

{

"id": 490,

"obj_id": 490,

"display_name": "Anonymous",

"name": "Anonymous",

"type": "Text Field",

"values": "",

"created_at": "05/07/2013",

"updated_at": "11/13/2014",

"created_by": [redacted],

"searchable_quick": false,

"searchable_advanced": true,

"searchable_batch": false,

"searchable": "Advanced",

"pii": "No",

"qtip": "",

"used_by": "Labmatrix Legacy - Subjects",

"used_by_display": "<a href='#' onClick='remoteJS({name:\"dialogs/AddSubjectTypeDlg\", obj_id:112 });'>Labmatrix Legacy - Subjects</a>"

},

{

"id": 411,

"obj_id": 411,

"display_name": "Answers",

"name": "Answers",

"type": "Text Area",

"values": "",

"created_at": "04/16/2013",

"updated_at": "11/13/2014",

"created_by": [redacted],

"searchable_quick": false,

"searchable_advanced": false,

"searchable_batch": false,

"searchable": "",

"pii": "No",

"qtip": "",

"used_by": "Question to Primary Care /Family",

"used_by_display": "<a href='#' onClick='remoteJS({name:\"dialogs/AddSubjectTypeDlg\", obj_id:84 });'>Question to Primary Care /Family</a>"

},

{

"id": 1703,

"obj_id": 1703,

"display_name": "Antibiotic Resistance",

"name": "Antibiotic Resistance",

"type": "Choice",

"values": "Yes, No",

"created_at": "09/25/2013",

"updated_at": "11/13/2014",

"created_by": [redacted],

"searchable_quick": false,

"searchable_advanced": true,

"searchable_batch": false,

"searchable": "Advanced",

"pii": "No",

"qtip": "",

"used_by": "Glycerol Stock",

"used_by_display": "<a href='#' onClick='remoteJS({name:\"dialogs/AddSubjectTypeDlg\", obj_id:171 });'>Glycerol Stock</a>"

},

{

"id": 2922,

"obj_id": 2922,

"display_name": "Antibody Catalog Number",

"name": "Antibody Catalog Number",

"type": "Text Field",

"values": "",

"created_at": "12/08/2014",

"updated_at": "12/08/2014",

"created_by": [redacted],

"searchable_quick": false,

"searchable_advanced": true,

"searchable_batch": false,

"searchable": "Advanced",

"pii": "No",

"qtip": "",

"used_by": "ChIP-Seq",

"used_by_display": "<a href='#' onClick='remoteJS({name:\"dialogs/AddSubjectTypeDlg\", obj_id:254 });'>ChIP-Seq</a>"

},

{

"id": 2920,

"obj_id": 2920,

"display_name": "Antibody Epitope",

"name": "Antibody Epitope",

"type": "Text Field",

"values": "",

"created_at": "12/08/2014",

"updated_at": "12/08/2014",

"created_by": [redacted],

"searchable_quick": false,

"searchable_advanced": true,

"searchable_batch": false,

"searchable": "Advanced",

"pii": "No",

"qtip": "",

"used_by": "ChIP-Seq",

"used_by_display": "<a href='#' onClick='remoteJS({name:\"dialogs/AddSubjectTypeDlg\", obj_id:254 });'>ChIP-Seq</a>"

},

{

"id": 2921,

"obj_id": 2921,

"display_name": "Antibody Manufacturer",

"name": "Antibody Manufacturer",

"type": "Text Field",

"values": "",

"created_at": "12/08/2014",

"updated_at": "12/08/2014",

"created_by": [redacted],

"searchable_quick": false,

"searchable_advanced": true,

"searchable_batch": false,

"searchable": "Advanced",

"pii": "No",

"qtip": "",

"used_by": "ChIP-Seq",

"used_by_display": "<a href='#' onClick='remoteJS({name:\"dialogs/AddSubjectTypeDlg\", obj_id:254 });'>ChIP-Seq</a>"

},

{

"id": 2667,

"obj_id": 2667,

"display_name": "Antibody Type",

"name": "Antibody Type",

"type": "Choice",

"values": "Monoclonal Ab, Polyclonal Ab, Fluorescent Ab",

"created_at": "06/11/2014",

"updated_at": "11/13/2014",

"created_by": [redacted],

"searchable_quick": false,

"searchable_advanced": true,

"searchable_batch": false,

"searchable": "Advanced",

"pii": "No",

"qtip": "",

"used_by": "Antibody, ChIP-Seq",

"used_by_display": "<a href='#' onClick='remoteJS({name:\"dialogs/AddSubjectTypeDlg\", obj_id:189 });'>Antibody</a>, <a href='#' onClick='remoteJS({name:\"dialogs/AddSubjectTypeDlg\", obj_id:254 });'>ChIP-Seq</a>"

},

{

"id": 2755,

"obj_id": 2755,

"display_name": "Antibody Validation",

"name": "Antibody Validation",

"type": "Choice",

"values": "Yes, No",

"created_at": "08/01/2014",

"updated_at": "11/13/2014",

"created_by": [redacted],

"searchable_quick": false,

"searchable_advanced": true,

"searchable_batch": false,

"searchable": "Advanced",

"pii": "No",

"qtip": "",

"used_by": "ChIP-Seq",

"used_by_display": "<a href='#' onClick='remoteJS({name:\"dialogs/AddSubjectTypeDlg\", obj_id:254 });'>ChIP-Seq</a>"

},

{

"id": 2939,

"obj_id": 2939,

"display_name": "Antibody Validation Services Report",

"name": "Antibody Validation Services Report",

"type": "File",

"values": "",

"created_at": "12/09/2014",

"updated_at": "12/09/2014",

"created_by": [redacted],

"searchable_quick": false,

"searchable_advanced": true,

"searchable_batch": false,

"searchable": "Advanced",

"pii": "No",

"qtip": "",

"used_by": "ChIP-Seq",

"used_by_display": "<a href='#' onClick='remoteJS({name:\"dialogs/AddSubjectTypeDlg\", obj_id:254 });'>ChIP-Seq</a>"

},

{

"id": 1257,

"obj_id": 1257,

"display_name": "Anticipated time to return",

"name": "Anticipated time to return",

"type": "Text Field",

"values": "",

"created_at": "06/03/2013",

"updated_at": "11/13/2014",

"created_by": [redacted],

"searchable_quick": false,

"searchable_advanced": true,

"searchable_batch": false,

"searchable": "Advanced",

"pii": "No",

"qtip": "Optional",

"used_by": "NIH Visit",

"used_by_display": "<a href='#' onClick='remoteJS({name:\"dialogs/AddSubjectTypeDlg\", obj_id:140 });'>NIH Visit</a>"

},

{

"id": 1198,

"obj_id": 1198,

"display_name": "Any Compound Medicines?",

"name": "Any Compound Medicines?",

"type": "Radio Buttons",

"values": "Yes, No, Unassessed",

"created_at": "05/29/2013",

"updated_at": "11/13/2014",

"created_by": [redacted],

"searchable_quick": false,

"searchable_advanced": true,

"searchable_batch": false,

"searchable": "Advanced",

"pii": "No",

"qtip": "",

"used_by": "Patient Visit Tools, Patient Visit Planning for Version C",

"used_by_display": "<a href='#' onClick='remoteJS({name:\"dialogs/AddSubjectTypeDlg\", obj_id:163 });'>Patient Visit Tools</a>, <a href='#' onClick='remoteJS({name:\"dialogs/AddSubjectTypeDlg\", obj_id:192 });'>Patient Visit Planning for Version C</a>"

},

{

"id": 1220,

"obj_id": 1220,

"display_name": "Any implanted devices not yet discussed?",

"name": "Any implanted devices not yet discussed?",

"type": "Radio Buttons",

"values": "Yes, No, Not Assessed",

"created_at": "05/31/2013",

"updated_at": "11/13/2014",

"created_by": [redacted],

"searchable_quick": false,

"searchable_advanced": true,

"searchable_batch": false,

"searchable": "Advanced",

"pii": "No",

"qtip": "",

"used_by": "Patient Visit Tools, Patient Visit Planning for Version C",

"used_by_display": "<a href='#' onClick='remoteJS({name:\"dialogs/AddSubjectTypeDlg\", obj_id:163 });'>Patient Visit Tools</a>, <a href='#' onClick='remoteJS({name:\"dialogs/AddSubjectTypeDlg\", obj_id:192 });'>Patient Visit Planning for Version C</a>"

},

{

"id": 1142,

"obj_id": 1142,

"display_name": "Any Implants?",

"name": "Any Implants?",

"type": "Text Area",

"values": "",

"created_at": "05/28/2013",

"updated_at": "11/13/2014",

"created_by": [redacted],

"searchable_quick": false,

"searchable_advanced": false,

"searchable_batch": false,

"searchable": "",

"pii": "No",

"qtip": "Pace Maker, Deep Brain Stimulator. Shunt, Central Line (Details)",

"used_by": "Patient Visit Tools, Patient Visit Planning for Version C",

"used_by_display": "<a href='#' onClick='remoteJS({name:\"dialogs/AddSubjectTypeDlg\", obj_id:163 });'>Patient Visit Tools</a>, <a href='#' onClick='remoteJS({name:\"dialogs/AddSubjectTypeDlg\", obj_id:192 });'>Patient Visit Planning for Version C</a>"

},

{

"id": 1361,

"obj_id": 1361,

"display_name": "Any other clinical procedure that need advance notice?",

"name": "Any other clinical procedure that need advance notice?",

"type": "Text Field",

"values": "",

"created_at": "06/24/2013",

"updated_at": "11/13/2014",

"created_by": [redacted],

"searchable_quick": false,

"searchable_advanced": true,

"searchable_batch": false,

"searchable": "Advanced",

"pii": "No",

"qtip": "",

"used_by": "Patient Visit Tools, Patient Visit Planning for Version C",

"used_by_display": "<a href='#' onClick='remoteJS({name:\"dialogs/AddSubjectTypeDlg\", obj_id:163 });'>Patient Visit Tools</a>, <a href='#' onClick='remoteJS({name:\"dialogs/AddSubjectTypeDlg\", obj_id:192 });'>Patient Visit Planning for Version C</a>"

},

{

"id": 1201,

"obj_id": 1201,

"display_name": "Any Other implanted devices not yet discussed?",

"name": "Any Other implanted devices not yet discussed?",

"type": "Radio Buttons",

"values": "Yes, No, Unassessed",

"created_at": "05/29/2013",

"updated_at": "11/13/2014",

"created_by": [redacted],

"searchable_quick": false,

"searchable_advanced": true,

"searchable_batch": false,

"searchable": "Advanced",

"pii": "No",

"qtip": "DBS, shunt, vagal nerve stimulator, screws/plates/staples/cochleear implant etc. If so create PIFUT for NP/PA/MD, consider changing Safety Status to moderate or high",

"used_by": "Patient Visit Tools, Patient Visit Planning for Version C",

"used_by_display": "<a href='#' onClick='remoteJS({name:\"dialogs/AddSubjectTypeDlg\", obj_id:163 });'>Patient Visit Tools</a>, <a href='#' onClick='remoteJS({name:\"dialogs/AddSubjectTypeDlg\", obj_id:192 });'>Patient Visit Planning for Version C</a>"

},

{

"id": 1140,

"obj_id": 1140,

"display_name": "Any Other Medical Equipment",

"name": "Any Other Medical Equipment",

"type": "Text Area",

"values": "",

"created_at": "05/28/2013",

"updated_at": "11/13/2014",

"created_by": [redacted],

"searchable_quick": false,

"searchable_advanced": false,

"searchable_batch": false,

"searchable": "",

"pii": "No",

"qtip": "Please list",

"used_by": "Patient Visit Tools, Patient Visit Planning for Version C",

"used_by_display": "<a href='#' onClick='remoteJS({name:\"dialogs/AddSubjectTypeDlg\", obj_id:163 });'>Patient Visit Tools</a>, <a href='#' onClick='remoteJS({name:\"dialogs/AddSubjectTypeDlg\", obj_id:192 });'>Patient Visit Planning for Version C</a>"

},

{

"id": 1190,

"obj_id": 1190,

"display_name": "Any Special Formulas being Used?",

"name": "Any Special Formulas being Used?",

"type": "Radio Buttons",

"values": "Yes, No, Unassessed",

"created_at": "05/29/2013",

"updated_at": "11/13/2014",

"created_by": [redacted],

"searchable_quick": false,

"searchable_advanced": true,

"searchable_batch": false,

"searchable": "Advanced",

"pii": "No",

"qtip": "If so, create PIFUT to arrange for formular to be available during NIH visit",

"used_by": "Patient Visit Tools, Patient Visit Planning for Version C",

"used_by_display": "<a href='#' onClick='remoteJS({name:\"dialogs/AddSubjectTypeDlg\", obj_id:163 });'>Patient Visit Tools</a>, <a href='#' onClick='remoteJS({name:\"dialogs/AddSubjectTypeDlg\", obj_id:192 });'>Patient Visit Planning for Version C</a>"

},

{

"id": 2460,

"obj_id": 2460,

"display_name": "Appistry comments",

"name": "Appistry comments",

"type": "Text Area",

"values": "",

"created_at": "03/26/2014",

"updated_at": "11/13/2014",

"created_by": [redacted],

"searchable_quick": false,

"searchable_advanced": false,

"searchable_batch": false,

"searchable": "",

"pii": "No",

"qtip": "",

"used_by": "Diploid Alignment",

"used_by_display": "<a href='#' onClick='remoteJS({name:\"dialogs/AddSubjectTypeDlg\", obj_id:209 });'>Diploid Alignment</a>"

},

{

"id": 2480,

"obj_id": 2480,

"display_name": "Appistry Expected Shipment Date",

"name": "Appistry Expected Shipment Date",

"type": "Date",

"values": "",

"created_at": "03/31/2014",

"updated_at": "11/13/2014",

"created_by": [redacted],

"searchable_quick": false,

"searchable_advanced": true,

"searchable_batch": false,

"searchable": "Advanced",

"pii": "No",

"qtip": "",

"used_by": "Alignment Information",

"used_by_display": "<a href='#' onClick='remoteJS({name:\"dialogs/AddSubjectTypeDlg\", obj_id:208 });'>Alignment Information</a>"

},

{

"id": 2301,

"obj_id": 2301,

"display_name": "Approved ",

"name": "Approved ",

"type": "Radio Buttons",

"values": "Yes, No",

"created_at": "02/26/2014",

"updated_at": "11/13/2014",

"created_by": [redacted],

"searchable_quick": false,

"searchable_advanced": true,

"searchable_batch": false,

"searchable": "Advanced",

"pii": "No",

"qtip": "",

"used_by": "",

"used_by_display": ""

},

{

"id": 1349,

"obj_id": 1349,

"display_name": "Approved for SNP Submission",

"name": "Approved for SNP Submission",

"type": "CheckBox",

"values": "",

"created_at": "06/21/2013",

"updated_at": "10/23/2014",

"created_by": [redacted],

"searchable_quick": false,

"searchable_advanced": true,

"searchable_batch": false,

"searchable": "Advanced",

"pii": "No",

"qtip": "",

"used_by": "SNP Analysis, SNP Chip Sequencing",

"used_by_display": "<a href='#' onClick='remoteJS({name:\"dialogs/AddSubjectTypeDlg\", obj_id:59 });'>SNP Analysis</a>, <a href='#' onClick='remoteJS({name:\"dialogs/AddSubjectTypeDlg\", obj_id:203 });'>SNP Chip Sequencing</a>"

},

{

"id": 1219,

"obj_id": 1219,

"display_name": "Are Emergency Medication used?",

"name": "Are Emergency Medication used?",

"type": "Radio Buttons",

"values": "Yes, No, Unassessed",

"created_at": "05/31/2013",

"updated_at": "11/13/2014",

"created_by": [redacted],

"searchable_quick": false,

"searchable_advanced": true,

"searchable_batch": false,

"searchable": "Advanced",

"pii": "No",

"qtip": "",

"used_by": "Patient Visit Tools, Patient Visit Planning for Version C",

"used_by_display": "<a href='#' onClick='remoteJS({name:\"dialogs/AddSubjectTypeDlg\", obj_id:163 });'>Patient Visit Tools</a>, <a href='#' onClick='remoteJS({name:\"dialogs/AddSubjectTypeDlg\", obj_id:192 });'>Patient Visit Planning for Version C</a>"

},

{

"id": 323,

"obj_id": 323,

"display_name": "AR Pedigree Score",

"name": "AR Pedigree Score",

"type": "Choice",

"values": "1, 2, 3, 4, 5",

"created_at": "04/08/2013",

"updated_at": "11/13/2014",

"created_by": [redacted],

"searchable_quick": false,

"searchable_advanced": true,

"searchable_batch": false,

"searchable": "Advanced",

"pii": "No",

"qtip": "",

"used_by": "Friday Meeting Notes, Sharepoint Legacy - Friday Meeting Notes",

"used_by_display": "<a href='#' onClick='remoteJS({name:\"dialogs/AddSubjectTypeDlg\", obj_id:82 });'>Friday Meeting Notes</a>, <a href='#' onClick='remoteJS({name:\"dialogs/AddSubjectTypeDlg\", obj_id:154 });'>Sharepoint Legacy - Friday Meeting Notes</a>"

},

{

"id": 1368,

"obj_id": 1368,

"display_name": "Ascertain parent's marital status",

"name": "Ascertain parent's marital status",

"type": "Radio Buttons",

"values": "Yes, No, Complete",

"created_at": "06/24/2013",

"updated_at": "11/13/2014",

"created_by": [redacted],

"searchable_quick": false,

"searchable_advanced": true,

"searchable_batch": false,

"searchable": "Advanced",

"pii": "Yes",

"qtip": "If patient under 18",

"used_by": "Patient Visit Tools, Patient Visit Planning for Version C",

"used_by_display": "<a href='#' onClick='remoteJS({name:\"dialogs/AddSubjectTypeDlg\", obj_id:163 });'>Patient Visit Tools</a>, <a href='#' onClick='remoteJS({name:\"dialogs/AddSubjectTypeDlg\", obj_id:192 });'>Patient Visit Planning for Version C</a>"

},

{

"id": 1144,

"obj_id": 1144,

"display_name": "Ask patient/parents for signed note from home Physician",

"name": "Ask patient/parents for signed note from home Physician",

"type": "CheckBox",

"values": "",

"created_at": "05/28/2013",

"updated_at": "11/13/2014",

"created_by": [redacted],

"searchable_quick": false,

"searchable_advanced": true,

"searchable_batch": false,

"searchable": "Advanced",

"pii": "No",

"qtip": "Note to cldar patient for travel",

"used_by": "Patient Visit Tools, Patient Visit Planning for Version C",

"used_by_display": "<a href='#' onClick='remoteJS({name:\"dialogs/AddSubjectTypeDlg\", obj_id:163 });'>Patient Visit Tools</a>, <a href='#' onClick='remoteJS({name:\"dialogs/AddSubjectTypeDlg\", obj_id:192 });'>Patient Visit Planning for Version C</a>"

},

{

"id": 1143,

"obj_id": 1143,

"display_name": "Ask patient/parents to bring:",

"name": "Ask patient/parents to bring:",

"type": "CheckBox",

"values": "",

"created_at": "05/28/2013",

"updated_at": "09/19/2013",

"created_by": [redacted],

"searchable_quick": false,

"searchable_advanced": null,

"searchable_batch": null,

"searchable": "",

"pii": "No",

"qtip": "Emergency Supplies, Routine Medical Supplies, Medications, Favorite Toys, Attachment Objects, Special Formula",

"used_by": "Patient Visit Tools, Patient Visit Planning for Version C",

"used_by_display": "<a href='#' onClick='remoteJS({name:\"dialogs/AddSubjectTypeDlg\", obj_id:163 });'>Patient Visit Tools</a>, <a href='#' onClick='remoteJS({name:\"dialogs/AddSubjectTypeDlg\", obj_id:192 });'>Patient Visit Planning for Version C</a>"

},

{

"id": 2810,

"obj_id": 2810,

"display_name": "Assay",

"name": "Assay",

"type": "Text Field",

"values": "",

"created_at": "08/28/2014",

"updated_at": "11/13/2014",

"created_by": [redacted],

"searchable_quick": false,

"searchable_advanced": true,

"searchable_batch": false,

"searchable": "Advanced",

"pii": "No",

"qtip": "Assay from Phenote",

"used_by": "Zebrafish Phenotype, Drosophila Phenotypes, Cohort Research Request",

"used_by_display": "<a href='#' onClick='remoteJS({name:\"dialogs/AddSubjectTypeDlg\", obj_id:173 });'>Zebrafish Phenotype</a>, <a href='#' onClick='remoteJS({name:\"dialogs/AddSubjectTypeDlg\", obj_id:265 });'>Drosophila Phenotypes</a>, <a href='#' onClick='remoteJS({name:\"dialogs/AddSubjectTypeDlg\", obj_id:271 });'>Cohort Research Request</a>"

},

{

"id": 2740,

"obj_id": 2740,

"display_name": "Assay Notes",

"name": "Assay Notes",

"type": "Text Area",

"values": "",

"created_at": "07/18/2014",

"updated_at": "11/13/2014",

"created_by": [redacted],

"searchable_quick": false,

"searchable_advanced": false,

"searchable_batch": false,

"searchable": "",

"pii": "No",

"qtip": "",

"used_by": "Zebrafish Mutation Project",

"used_by_display": "<a href='#' onClick='remoteJS({name:\"dialogs/AddSubjectTypeDlg\", obj_id:186 });'>Zebrafish Mutation Project</a>"

},

{

"id": 2465,

"obj_id": 2465,

"display_name": "Assays",

"name": "Assays",

"type": "Subject Back Ref",

"values": "",

"created_at": "03/28/2014",

"updated_at": "11/13/2014",

"created_by": [redacted],

"searchable_quick": false,

"searchable_advanced": true,

"searchable_batch": false,

"searchable": "Advanced",

"pii": "No",

"qtip": "",

"used_by": "Patient, Patient Follow Up Visit",

"used_by_display": "<a href='#' onClick='remoteJS({name:\"dialogs/AddSubjectTypeDlg\", obj_id:1 });'>Patient</a>, <a href='#' onClick='remoteJS({name:\"dialogs/AddSubjectTypeDlg\", obj_id:264 });'>Patient Follow Up Visit</a>"

},

{

"id": 2457,

"obj_id": 2457,

"display_name": "Assay Type",

"name": "Assay Type",

"type": "Choice",

"values": "Western Blot, qPCR, Functional Assay, Lysotracker Assay, Lectin Stain, Organelle Stain, UPR-stress, Seahorse",

"created_at": "03/26/2014",

"updated_at": "10/23/2014",

"created_by": [redacted],

"searchable_quick": false,

"searchable_advanced": true,

"searchable_batch": false,

"searchable": "Advanced",

"pii": "No",

"qtip": "",

"used_by": "Assay",

"used_by_display": "<a href='#' onClick='remoteJS({name:\"dialogs/AddSubjectTypeDlg\", obj_id:225 });'>Assay</a>"

},

{

"id": 2329,

"obj_id": 2329,

"display_name": "Assessment:",

"name": "Assessment:",

"type": "Text Area",

"values": "",

"created_at": "03/04/2014",

"updated_at": "11/13/2014",

"created_by": [redacted],

"searchable_quick": false,

"searchable_advanced": false,

"searchable_batch": false,

"searchable": "",

"pii": "Yes",

"qtip": "",

"used_by": "Patient Review",

"used_by_display": "<a href='#' onClick='remoteJS({name:\"dialogs/AddSubjectTypeDlg\", obj_id:83 });'>Patient Review</a>"

},

{

"id": 49,

"obj_id": 49,

"display_name": "Assigned Clinician",

"name": "Assigned Clinician",

"type": "User",

"values": "",

"created_at": "02/04/2013",

"updated_at": "11/13/2014",

"created_by": [redacted],

"searchable_quick": false,

"searchable_advanced": true,

"searchable_batch": false,

"searchable": "Advanced",

"pii": "No",

"qtip": "Clinician to perform Phone Interview",

"used_by": "Patient Visit Tools, Patient Visit Planning for Version C",

"used_by_display": "<a href='#' onClick='remoteJS({name:\"dialogs/AddSubjectTypeDlg\", obj_id:163 });'>Patient Visit Tools</a>, <a href='#' onClick='remoteJS({name:\"dialogs/AddSubjectTypeDlg\", obj_id:192 });'>Patient Visit Planning for Version C</a>"

},

{

"id": 1290,

"obj_id": 1290,

"display_name": "Assigned Tasks",

"name": "Assigned Tasks",

"type": "Subject Back Ref",

"values": "",

"created_at": "06/05/2013",

"updated_at": "01/23/2015",

"created_by": [redacted],

"searchable_quick": true,

"searchable_advanced": true,

"searchable_batch": true,

"searchable": "Quick,Advanced,Batch",

"pii": "No",

"qtip": "",

"used_by": "Patient Visit Planning for Version C",

"used_by_display": "<a href='#' onClick='remoteJS({name:\"dialogs/AddSubjectTypeDlg\", obj_id:192 });'>Patient Visit Planning for Version C</a>"

},

{

"id": 1237,

"obj_id": 1237,

"display_name": "Assign task to",

"name": "Assign task to",

"type": "Choice",

"values": [redacted],

"created_at": "06/03/2013",

"updated_at": "01/23/2015",

"created_by": [redacted],

"searchable_quick": true,

"searchable_advanced": true,

"searchable_batch": true,

"searchable": "Quick,Advanced,Batch",

"pii": "Yes",

"qtip": "",

"used_by": "NIH Visit, Patient Visit Tools, Patient Visit Planning for Version C",

"used_by_display": "<a href='#' onClick='remoteJS({name:\"dialogs/AddSubjectTypeDlg\", obj_id:140 });'>NIH Visit</a>, <a href='#' onClick='remoteJS({name:\"dialogs/AddSubjectTypeDlg\", obj_id:163 });'>Patient Visit Tools</a>, <a href='#' onClick='remoteJS({name:\"dialogs/AddSubjectTypeDlg\", obj_id:192 });'>Patient Visit Planning for Version C</a>"

},

{

"id": 2955,

"obj_id": 2955,

"display_name": "Associated Glycomics Results",

"name": "Associated Glycomics Results",

"type": "Subjects",

"values": "",

"created_at": "01/13/2015",

"updated_at": "01/13/2015",

"created_by": [redacted],

"searchable_quick": false,

"searchable_advanced": null,

"searchable_batch": null,

"searchable": "",

"pii": "No",

"qtip": "Associated Results for Invoice",

"used_by": "Invoice Information",

"used_by_display": "<a href='#' onClick='remoteJS({name:\"dialogs/AddSubjectTypeDlg\", obj_id:270 });'>Invoice Information</a>"

},

{

"id": 2956,

"obj_id": 2956,

"display_name": "Associated Glycomics Results [2]",

"name": "Associated Glycomics Results [2]",

"type": "Subjects",

"values": "",

"created_at": "01/13/2015",

"updated_at": "01/13/2015",

"created_by": [redacted],

"searchable_quick": false,

"searchable_advanced": null,

"searchable_batch": null,

"searchable": "",

"pii": "No",

"qtip": "Associated Results for Invoice",

"used_by": "",

"used_by_display": ""

},

{

"id": 2957,

"obj_id": 2957,

"display_name": "Associated Glycomics Results [3]",

"name": "Associated Glycomics Results [3]",

"type": "Subjects",

"values": "",

"created_at": "01/13/2015",

"updated_at": "01/13/2015",

"created_by": [redacted],

"searchable_quick": false,

"searchable_advanced": null,

"searchable_batch": null,

"searchable": "",

"pii": "No",

"qtip": "Associated Results for Invoice",

"used_by": "",

"used_by_display": ""

},

{

"id": 2958,

"obj_id": 2958,

"display_name": "Associated Glycomics Results [4]",

"name": "Associated Glycomics Results [4]",

"type": "Subjects",

"values": "",

"created_at": "01/13/2015",

"updated_at": "01/13/2015",

"created_by": [redacted],

"searchable_quick": false,

"searchable_advanced": null,

"searchable_batch": null,

"searchable": "",

"pii": "No",

"qtip": "Associated Results for Invoice",

"used_by": "",

"used_by_display": ""

},

{

"id": 2959,

"obj_id": 2959,

"display_name": "Associated Glycomics Results [5]",

"name": "Associated Glycomics Results [5]",

"type": "Subjects",

"values": "",

"created_at": "01/13/2015",

"updated_at": "01/13/2015",

"created_by": [redacted],

"searchable_quick": false,

"searchable_advanced": null,

"searchable_batch": null,

"searchable": "",

"pii": "No",

"qtip": "Associated Results for Invoice",

"used_by": "",

"used_by_display": ""

},

{

"id": 2849,

"obj_id": 2849,

"display_name": "Associated Invoice Report",

"name": "Associated Invoice Report",

"type": "Subject",

"values": "",

"created_at": "09/24/2014",

"updated_at": "11/13/2014",

"created_by": [redacted],

"searchable_quick": true,

"searchable_advanced": true,

"searchable_batch": true,

"searchable": "Quick,Advanced,Batch",

"pii": "No",

"qtip": "",

"used_by": "",

"used_by_display": ""

},

{

"id": 2953,

"obj_id": 2953,

"display_name": "Associated -omics Results",

"name": "Associated -omics Results",

"type": "Subjects of Several Types",

"values": "",

"created_at": "01/13/2015",

"updated_at": "01/13/2015",

"created_by": [redacted],

"searchable_quick": false,

"searchable_advanced": null,

"searchable_batch": null,

"searchable": "",

"pii": "No",

"qtip": "",

"used_by": "",

"used_by_display": ""

},

{

"id": 2975,

"obj_id": 2975,

"display_name": "Association of Gene with Human Disease",

"name": "Association of Gene with Human Disease",

"type": "Text Area",

"values": "",

"created_at": "01/22/2015",

"updated_at": "01/22/2015",

"created_by": [redacted],

"searchable_quick": false,

"searchable_advanced": false,

"searchable_batch": false,

"searchable": "",

"pii": "No",

"qtip": "",

"used_by": "Variant Prioritization",

"used_by_display": "<a href='#' onClick='remoteJS({name:\"dialogs/AddSubjectTypeDlg\", obj_id:279 });'>Variant Prioritization</a>"

},

{

"id": 2332,

"obj_id": 2332,

"display_name": "At NIH should have:",

"name": "At NIH should have:",

"type": "Text Area",

"values": "",

"created_at": "03/04/2014",

"updated_at": "11/13/2014",

"created_by": [redacted],

"searchable_quick": false,

"searchable_advanced": false,

"searchable_batch": false,

"searchable": "",

"pii": "Yes",

"qtip": "",

"used_by": "Patient Review",

"used_by_display": "<a href='#' onClick='remoteJS({name:\"dialogs/AddSubjectTypeDlg\", obj_id:83 });'>Patient Review</a>"

},

{

"id": 491,

"obj_id": 491,

"display_name": "Attachment Id",

"name": "Attachment Id",

"type": "Text Field",

"values": "",

"created_at": "05/07/2013",

"updated_at": "11/13/2014",

"created_by": [redacted],

"searchable_quick": false,

"searchable_advanced": true,

"searchable_batch": false,

"searchable": "Advanced",

"pii": "No",

"qtip": "",

"used_by": "Labmatrix Legacy - Communications",

"used_by_display": "<a href='#' onClick='remoteJS({name:\"dialogs/AddSubjectTypeDlg\", obj_id:92 });'>Labmatrix Legacy - Communications</a>"

},

{

"id": 492,

"obj_id": 492,

"display_name": "Attachments",

"name": "Attachments",

"type": "Text Area",

"values": "",

"created_at": "05/07/2013",

"updated_at": "11/13/2014",

"created_by": [redacted],

"searchable_quick": false,

"searchable_advanced": false,

"searchable_batch": false,

"searchable": "",

"pii": "Yes",

"qtip": "",

"used_by": "Labmatrix Legacy - Example Record, Labmatrix Legacy - Test Order Records, Labmatrix Legacy - Todo Form, Labmatrix Legacy - Send Out Tests, Labmatrix Legacy - Adult UDP Entry",

"used_by_display": "<a href='#' onClick='remoteJS({name:\"dialogs/AddSubjectTypeDlg\", obj_id:102 });'>Labmatrix Legacy - Example Record</a>, <a href='#' onClick='remoteJS({name:\"dialogs/AddSubjectTypeDlg\", obj_id:106 });'>Labmatrix Legacy - Test Order Records</a>, <a href='#' onClick='remoteJS({name:\"dialogs/AddSubjectTypeDlg\", obj_id:107 });'>Labmatrix Legacy - Todo Form</a>, <a href='#' onClick='remoteJS({name:\"dialogs/AddSubjectTypeDlg\", obj_id:104 });'>Labmatrix Legacy - Send Out Tests</a>, <a href='#' onClick='remoteJS({name:\"dialogs/AddSubjectTypeDlg\", obj_id:101 });'>Labmatrix Legacy - Adult UDP Entry</a>"

},

{

"id": 1266,

"obj_id": 1266,

"display_name": "Attachments (optional)",

"name": "Attachments (optional)",

"type": "Radio Buttons",

"values": "Yes, No",

"created_at": "06/03/2013",

"updated_at": "11/13/2014",

"created_by": [redacted],

"searchable_quick": false,

"searchable_advanced": true,

"searchable_batch": false,

"searchable": "Advanced",

"pii": "No",

"qtip": "Scan into patient&#x27;s file (requisition, ressults etc)",

"used_by": "NIH Visit",

"used_by_display": "<a href='#' onClick='remoteJS({name:\"dialogs/AddSubjectTypeDlg\", obj_id:140 });'>NIH Visit</a>"

},

{

"id": 249,

"obj_id": 249,

"display_name": "Attending",

"name": "Attending",

"type": "Choice",

"values": [redacted],

"created_at": "03/28/2013",

"updated_at": "11/13/2014",

"created_by": [redacted],

"searchable_quick": true,

"searchable_advanced": true,

"searchable_batch": true,

"searchable": "Quick,Advanced,Batch",

"pii": "No",

"qtip": "",

"used_by": "Labmatrix Legacy - Peds Sharepoint Seen, Labmatrix Legacy - Peds Sharepoint Seen List, Sharepoint Legacy - Pediatric Patient, Sharepoint Legacy - Adult Patient Status",

"used_by_display": "<a href='#' onClick='remoteJS({name:\"dialogs/AddSubjectTypeDlg\", obj_id:97 });'>Labmatrix Legacy - Peds Sharepoint Seen</a>, <a href='#' onClick='remoteJS({name:\"dialogs/AddSubjectTypeDlg\", obj_id:98 });'>Labmatrix Legacy - Peds Sharepoint Seen List</a>, <a href='#' onClick='remoteJS({name:\"dialogs/AddSubjectTypeDlg\", obj_id:114 });'>Sharepoint Legacy - Pediatric Patient</a>, <a href='#' onClick='remoteJS({name:\"dialogs/AddSubjectTypeDlg\", obj_id:115 });'>Sharepoint Legacy - Adult Patient Status</a>"

},

{

"id": 1267,

"obj_id": 1267,

"display_name": "Attending/NP/PA aware of results",

"name": "Attending/NP/PA aware of results",

"type": "Radio Buttons",

"values": "Yes, No",

"created_at": "06/03/2013",

"updated_at": "11/13/2014",

"created_by": [redacted],

"searchable_quick": false,

"searchable_advanced": true,

"searchable_batch": false,

"searchable": "Advanced",

"pii": "No",

"qtip": "",

"used_by": "NIH Visit",

"used_by_display": "<a href='#' onClick='remoteJS({name:\"dialogs/AddSubjectTypeDlg\", obj_id:140 });'>NIH Visit</a>"

},

{

"id": 2016,

"obj_id": 2016,

"display_name": "Attending Physician",

"name": "Attending Physician",

"type": "User",

"values": "",

"created_at": "11/20/2013",

"updated_at": "11/07/2014",

"created_by": [redacted],

"searchable_quick": true,

"searchable_advanced": true,

"searchable_batch": true,

"searchable": "Quick,Advanced,Batch",

"pii": "No",

"qtip": "",

"used_by": "Patient, Patient Follow Up Visit",

"used_by_display": "<a href='#' onClick='remoteJS({name:\"dialogs/AddSubjectTypeDlg\", obj_id:1 });'>Patient</a>, <a href='#' onClick='remoteJS({name:\"dialogs/AddSubjectTypeDlg\", obj_id:264 });'>Patient Follow Up Visit</a>"

},

{

"id": 1463,

"obj_id": 1463,

"display_name": "Attending Physician (Sharepoint)",

"name": "Attending Physician (Sharepoint)",

"type": "Text Field",

"values": "",

"created_at": "07/18/2013",

"updated_at": "11/13/2014",

"created_by": [redacted],

"searchable_quick": false,

"searchable_advanced": true,

"searchable_batch": false,

"searchable": "Advanced",

"pii": "No",

"qtip": "Sharepoint Legacy Field",

"used_by": "Sharepoint Legacy - Friday Meeting Notes",

"used_by_display": "<a href='#' onClick='remoteJS({name:\"dialogs/AddSubjectTypeDlg\", obj_id:154 });'>Sharepoint Legacy - Friday Meeting Notes</a>"

},

{

"id": 493,

"obj_id": 493,

"display_name": "Attribute 1",

"name": "Attribute 1",

"type": "Text Field",

"values": "",

"created_at": "05/07/2013",

"updated_at": "11/13/2014",

"created_by": [redacted],

"searchable_quick": false,

"searchable_advanced": true,

"searchable_batch": false,

"searchable": "Advanced",

"pii": "Yes",

"qtip": "",

"used_by": "Patient, Labmatrix Legacy - Subjects, Patient Follow Up Visit",

"used_by_display": "<a href='#' onClick='remoteJS({name:\"dialogs/AddSubjectTypeDlg\", obj_id:1 });'>Patient</a>, <a href='#' onClick='remoteJS({name:\"dialogs/AddSubjectTypeDlg\", obj_id:112 });'>Labmatrix Legacy - Subjects</a>, <a href='#' onClick='remoteJS({name:\"dialogs/AddSubjectTypeDlg\", obj_id:264 });'>Patient Follow Up Visit</a>"

},

{

"id": 494,

"obj_id": 494,

"display_name": "Attribute 2",

"name": "Attribute 2",

"type": "Text Field",

"values": "",

"created_at": "05/07/2013",

"updated_at": "11/13/2014",

"created_by": [redacted],

"searchable_quick": false,

"searchable_advanced": true,

"searchable_batch": false,

"searchable": "Advanced",

"pii": "Yes",

"qtip": "",

"used_by": "Patient, Labmatrix Legacy - Subjects, Patient Follow Up Visit",

"used_by_display": "<a href='#' onClick='remoteJS({name:\"dialogs/AddSubjectTypeDlg\", obj_id:1 });'>Patient</a>, <a href='#' onClick='remoteJS({name:\"dialogs/AddSubjectTypeDlg\", obj_id:112 });'>Labmatrix Legacy - Subjects</a>, <a href='#' onClick='remoteJS({name:\"dialogs/AddSubjectTypeDlg\", obj_id:264 });'>Patient Follow Up Visit</a>"

},

{

"id": 1371,

"obj_id": 1371,

"display_name": "ATV's created",

"name": "ATV's created",

"type": "Radio Buttons",

"values": "Yes, No, Partial, Complete",

"created_at": "06/24/2013",

"updated_at": "11/13/2014",

"created_by": [redacted],

"searchable_quick": false,

"searchable_advanced": true,

"searchable_batch": false,

"searchable": "Advanced",

"pii": "No",

"qtip": "One for each person traveling",

"used_by": "Patient Visit Tools, Patient Visit Planning for Version C",

"used_by_display": "<a href='#' onClick='remoteJS({name:\"dialogs/AddSubjectTypeDlg\", obj_id:163 });'>Patient Visit Tools</a>, <a href='#' onClick='remoteJS({name:\"dialogs/AddSubjectTypeDlg\", obj_id:192 });'>Patient Visit Planning for Version C</a>"

},

{

"id": 1567,

"obj_id": 1567,

"display_name": "Audiology and/or ABR",

"name": "Audiology and/or ABR",

"type": "Radio Buttons",

"values": "Yes, No",

"created_at": "08/12/2013",

"updated_at": "11/13/2014",

"created_by": [redacted],

"searchable_quick": false,

"searchable_advanced": true,

"searchable_batch": false,

"searchable": "Advanced",

"pii": "No",

"qtip": "",

"used_by": "Patient Visit Tools, Patient Visit Planning for Version C",

"used_by_display": "<a href='#' onClick='remoteJS({name:\"dialogs/AddSubjectTypeDlg\", obj_id:163 });'>Patient Visit Tools</a>, <a href='#' onClick='remoteJS({name:\"dialogs/AddSubjectTypeDlg\", obj_id:192 });'>Patient Visit Planning for Version C</a>"

},

{

"id": 2686,

"obj_id": 2686,

"display_name": "Available_Studies_Radiology",

"name": "Available_Studies_Radiology",

"type": "Choice",

"values": "Studies Verified as Available in PACS, Studies Available in PACS/None for this Review Type, PACS +Unloadable and/or Plain Film Only, No Studies Available",

"created_at": "06/19/2014",

"updated_at": "10/31/2014",

"created_by": [redacted],

"searchable_quick": false,

"searchable_advanced": true,

"searchable_batch": false,

"searchable": "Advanced",

"pii": "Yes",

"qtip": "",

"used_by": "Radiology Review",

"used_by_display": "<a href='#' onClick='remoteJS({name:\"dialogs/AddSubjectTypeDlg\", obj_id:246 });'>Radiology Review</a>"

},

{

"id": 1380,

"obj_id": 1380,

"display_name": "Average Fragment Size",

"name": "Average Fragment Size",

"type": "Text Field",

"values": "",

"created_at": "06/24/2013",

"updated_at": "11/13/2014",

"created_by": [redacted],

"searchable_quick": false,

"searchable_advanced": true,

"searchable_batch": false,

"searchable": "Advanced",

"pii": "No",

"qtip": "for ChIP-Seq of other pre-sheared samples",

"used_by": "Exome Sequencing",

"used_by_display": "<a href='#' onClick='remoteJS({name:\"dialogs/AddSubjectTypeDlg\", obj_id:161 });'>Exome Sequencing</a>"

},

{

"id": 2873,

"obj_id": 2873,

"display_name": "Axeq Analysis File",

"name": "Axeq Analysis File",

"type": "File",

"values": "",

"created_at": "11/07/2014",

"updated_at": "11/12/2014",

"created_by": [redacted],

"searchable_quick": false,

"searchable_advanced": true,

"searchable_batch": false,

"searchable": "Advanced",

"pii": "No",

"qtip": "",

"used_by": "Exome Sequencing",

"used_by_display": "<a href='#' onClick='remoteJS({name:\"dialogs/AddSubjectTypeDlg\", obj_id:161 });'>Exome Sequencing</a>"

},

{

"id": 2874,

"obj_id": 2874,

"display_name": "Axeq SNP and INDEL file",

"name": "Axeq SNP and INDEL file",

"type": "File",

"values": "",

"created_at": "11/07/2014",

"updated_at": "11/12/2014",

"created_by": [redacted],

"searchable_quick": false,

"searchable_advanced": true,

"searchable_batch": false,

"searchable": "Advanced",

"pii": "No",

"qtip": "Returned from Axeq with each sample sequencing data",

"used_by": "Exome Sequencing",

"used_by_display": "<a href='#' onClick='remoteJS({name:\"dialogs/AddSubjectTypeDlg\", obj_id:161 });'>Exome Sequencing</a>"

},

{

"id": 2797,

"obj_id": 2797,

"display_name": "Background",

"name": "Background",

"type": "Text Field",

"values": "",

"created_at": "08/28/2014",

"updated_at": "11/13/2014",

"created_by": [redacted],

"searchable_quick": false,

"searchable_advanced": true,

"searchable_batch": false,

"searchable": "Advanced",

"pii": "No",

"qtip": "Background from Phenote",

"used_by": "Zebrafish Phenotype, Drosophila Phenotypes",

"used_by_display": "<a href='#' onClick='remoteJS({name:\"dialogs/AddSubjectTypeDlg\", obj_id:173 });'>Zebrafish Phenotype</a>, <a href='#' onClick='remoteJS({name:\"dialogs/AddSubjectTypeDlg\", obj_id:265 });'>Drosophila Phenotypes</a>"

},

{

"id": 1932,

"obj_id": 1932,

"display_name": "Bacterial Resistance",

"name": "Bacterial Resistance",

"type": "List",

"values": "None, Amoxicillin, Ampicillin, Canavanine, Carbenicillin, Chloramphenicol, Doxycycline, Erythromycin, Kanamycin, Neomycine/G418, Penicillin, Rifampicin, Spectinimycin, Streptomycin, Tetrocycline, Other, see comments",

"created_at": "11/11/2013",

"updated_at": "11/13/2014",

"created_by": [redacted],

"searchable_quick": false,

"searchable_advanced": true,

"searchable_batch": false,

"searchable": "Advanced",

"pii": "No",

"qtip": "",

"used_by": "Destination Vector Construction, Glycerol Stock",

"used_by_display": "<a href='#' onClick='remoteJS({name:\"dialogs/AddSubjectTypeDlg\", obj_id:168 });'>Destination Vector Construction</a>, <a href='#' onClick='remoteJS({name:\"dialogs/AddSubjectTypeDlg\", obj_id:171 });'>Glycerol Stock</a>"

},

{

"id": 1182,

"obj_id": 1182,

"display_name": "Bacterial Strain",

"name": "Bacterial Strain",

"type": "Text Field",

"values": "",

"created_at": "05/29/2013",

"updated_at": "11/13/2014",

"created_by": [redacted],

"searchable_quick": false,

"searchable_advanced": true,

"searchable_batch": false,

"searchable": "Advanced",

"pii": "No",

"qtip": "",

"used_by": "Glycerol Stock, Plasmid Reagents",

"used_by_display": "<a href='#' onClick='remoteJS({name:\"dialogs/AddSubjectTypeDlg\", obj_id:171 });'>Glycerol Stock</a>, <a href='#' onClick='remoteJS({name:\"dialogs/AddSubjectTypeDlg\", obj_id:177 });'>Plasmid Reagents</a>"

},

{

"id": 2190,

"obj_id": 2190,

"display_name": "Bacteria Strain",

"name": "Bacteria Strain",

"type": "Choice",

"values": "TOP10, DH5a, MACH1, Stbl3, ccdb survival, Stbl4, BL21A1",

"created_at": "01/27/2014",

"updated_at": "11/13/2014",

"created_by": [redacted],

"searchable_quick": false,

"searchable_advanced": true,

"searchable_batch": false,

"searchable": "Advanced",

"pii": "No",

"qtip": "",

"used_by": "Glycerol Stock",

"used_by_display": "<a href='#' onClick='remoteJS({name:\"dialogs/AddSubjectTypeDlg\", obj_id:171 });'>Glycerol Stock</a>"

},

{

"id": 1924,

"obj_id": 1924,

"display_name": "Bacteria Transformed for Destination Vector Creation",

"name": "Bacteria Transformed for Destination Vector Creation",

"type": "Text Field",

"values": "",

"created_at": "11/11/2013",

"updated_at": "11/13/2014",

"created_by": [redacted],

"searchable_quick": false,

"searchable_advanced": true,

"searchable_batch": false,

"searchable": "Advanced",

"pii": "No",

"qtip": "",

"used_by": "Destination Vector Construction",

"used_by_display": "<a href='#' onClick='remoteJS({name:\"dialogs/AddSubjectTypeDlg\", obj_id:168 });'>Destination Vector Construction</a>"

},

{

"id": 1923,

"obj_id": 1923,

"display_name": "Bacteria Transformed for pENTR Vector Creation",

"name": "Bacteria Transformed for pENTR Vector Creation",

"type": "Text Field",

"values": "",

"created_at": "11/11/2013",

"updated_at": "11/13/2014",

"created_by": [redacted],

"searchable_quick": false,

"searchable_advanced": true,

"searchable_batch": false,

"searchable": "Advanced",

"pii": "No",

"qtip": "",

"used_by": "Destination Vector Construction, Entry Vector",

"used_by_display": "<a href='#' onClick='remoteJS({name:\"dialogs/AddSubjectTypeDlg\", obj_id:168 });'>Destination Vector Construction</a>, <a href='#' onClick='remoteJS({name:\"dialogs/AddSubjectTypeDlg\", obj_id:193 });'>Entry Vector</a>"

},

{

"id": 168,

"obj_id": 168,

"display_name": "BAM File Location",

"name": "BAM File Location",

"type": "Text Field",

"values": "",

"created_at": "02/21/2013",

"updated_at": "11/13/2014",

"created_by": [redacted],

"searchable_quick": false,

"searchable_advanced": true,

"searchable_batch": false,

"searchable": "Advanced",

"pii": "No",

"qtip": "Path to BAM file",

"used_by": "ChIP-Seq, Exome Analysis, Transcriptome, Diploid Alignment",

"used_by_display": "<a href='#' onClick='remoteJS({name:\"dialogs/AddSubjectTypeDlg\", obj_id:254 });'>ChIP-Seq</a>, <a href='#' onClick='remoteJS({name:\"dialogs/AddSubjectTypeDlg\", obj_id:60 });'>Exome Analysis</a>, <a href='#' onClick='remoteJS({name:\"dialogs/AddSubjectTypeDlg\", obj_id:255 });'>Transcriptome</a>, <a href='#' onClick='remoteJS({name:\"dialogs/AddSubjectTypeDlg\", obj_id:209 });'>Diploid Alignment</a>"

},

{

"id": 1976,

"obj_id": 1976,

"display_name": "BAM File Present",

"name": "BAM File Present",

"type": "Choice",

"values": "Yes, No, Unknown",

"created_at": "11/19/2013",

"updated_at": "11/13/2014",

"created_by": [redacted],

"searchable_quick": false,

"searchable_advanced": true,

"searchable_batch": false,

"searchable": "Advanced",

"pii": "No",

"qtip": "",

"used_by": "Exome Sequencing, Alignment Information",

"used_by_display": "<a href='#' onClick='remoteJS({name:\"dialogs/AddSubjectTypeDlg\", obj_id:161 });'>Exome Sequencing</a>, <a href='#' onClick='remoteJS({name:\"dialogs/AddSubjectTypeDlg\", obj_id:208 });'>Alignment Information</a>"

},

{

"id": 74,

"obj_id": 74,

"display_name": "Barcode",

"name": "Barcode",

"type": "Text Field",

"values": "",

"created_at": "02/06/2013",

"updated_at": "09/19/2013",

"created_by": [redacted],

"searchable_quick": false,

"searchable_advanced": null,

"searchable_batch": null,

"searchable": "",

"pii": "No",

"qtip": "Barcode created for project",

"used_by": "Labmatrix Legacy - Biomaterials",

"used_by_display": "<a href='#' onClick='remoteJS({name:\"dialogs/AddSubjectTypeDlg\", obj_id:91 });'>Labmatrix Legacy - Biomaterials</a>"

},

{

"id": 2382,

"obj_id": 2382,

"display_name": "Batch Design Requested",

"name": "Batch Design Requested",

"type": "Choice",

"values": "Yes, No",

"created_at": "03/11/2014",

"updated_at": "11/13/2014",

"created_by": [redacted],

"searchable_quick": false,

"searchable_advanced": true,

"searchable_batch": false,

"searchable": "Advanced",

"pii": "No",

"qtip": "ONLY for large numbers of primer requests",

"used_by": "Primer Order",

"used_by_display": "<a href='#' onClick='remoteJS({name:\"dialogs/AddSubjectTypeDlg\", obj_id:217 });'>Primer Order</a>"

},

{

"id": 1900,

"obj_id": 1900,

"display_name": "BEAGLE Version Used",

"name": "BEAGLE Version Used",

"type": "Text Field",

"values": "",

"created_at": "11/08/2013",

"updated_at": "11/13/2014",

"created_by": [redacted],

"searchable_quick": false,

"searchable_advanced": true,

"searchable_batch": false,

"searchable": "Advanced",

"pii": "No",

"qtip": "",

"used_by": "Phased and Imputed VCF",

"used_by_display": "<a href='#' onClick='remoteJS({name:\"dialogs/AddSubjectTypeDlg\", obj_id:188 });'>Phased and Imputed VCF</a>"

},

{

"id": 2633,

"obj_id": 2633,

"display_name": "Bedfiles",

"name": "Bedfiles",

"type": "Subject Back Ref",

"values": "",

"created_at": "05/20/2014",

"updated_at": "05/20/2014",

"created_by": [redacted],

"searchable_quick": false,

"searchable_advanced": null,

"searchable_batch": null,

"searchable": "",

"pii": "No",

"qtip": "",

"used_by": "SNP Analysis",

"used_by_display": "<a href='#' onClick='remoteJS({name:\"dialogs/AddSubjectTypeDlg\", obj_id:59 });'>SNP Analysis</a>"

},

{

"id": 2824,

"obj_id": 2824,

"display_name": "Bedfile Type",

"name": "Bedfile Type",

"type": "List",

"values": "Deletion, Linkage, Homozygosity",

"created_at": "09/10/2014",

"updated_at": "11/11/2014",

"created_by": [redacted],

"searchable_quick": false,

"searchable_advanced": true,

"searchable_batch": false,

"searchable": "Advanced",

"pii": "No",

"qtip": "",

"used_by": "Exome Analysis Results",

"used_by_display": "<a href='#' onClick='remoteJS({name:\"dialogs/AddSubjectTypeDlg\", obj_id:156 });'>Exome Analysis Results</a>"

},

{

"id": 2565,

"obj_id": 2565,

"display_name": "Bedfile Upload",

"name": "Bedfile Upload",

"type": "File",

"values": "",

"created_at": "04/25/2014",

"updated_at": "04/25/2014",

"created_by": [redacted],

"searchable_quick": false,

"searchable_advanced": null,

"searchable_batch": null,

"searchable": "",

"pii": "No",

"qtip": "",

"used_by": "Bedfiles",

"used_by_display": "<a href='#' onClick='remoteJS({name:\"dialogs/AddSubjectTypeDlg\", obj_id:238 });'>Bedfiles</a>"

},

{

"id": 1735,

"obj_id": 1735,

"display_name": "Behaviour Analysis Description",

"name": "Behaviour Analysis Description",

"type": "Text Area",

"values": "",

"created_at": "10/17/2013",

"updated_at": "11/13/2014",

"created_by": [redacted],

"searchable_quick": false,

"searchable_advanced": false,

"searchable_batch": false,

"searchable": "",

"pii": "No",

"qtip": "",

"used_by": "",

"used_by_display": ""

},

{

"id": 1734,

"obj_id": 1734,

"display_name": "Behaviour Analysis Stage",

"name": "Behaviour Analysis Stage",

"type": "Choice",

"values": "Hatching, Long-Pec (48 hr), Hatching, Pec-Fin (60 hr), Larval, Protruding Mouth (72 hr), Larval, Day 4, Larval, Day 5, Larval, Day 6, Larval, Day 7, Larval, Day 8, Larval, Day 9, Larval, Day 10, Larval, Day 11, Larval, Day 12, Larval, Day 13, Larval, Day 14, Larval, Days 15-20, Larval, Days 21-29, Juvenile, Days 30-44, Juvenile, Days 45-89, Adult, Days 90+, Other, Unknown",

"created_at": "10/17/2013",

"updated_at": "11/13/2014",

"created_by": [redacted],

"searchable_quick": false,

"searchable_advanced": true,

"searchable_batch": false,

"searchable": "Advanced",

"pii": "No",

"qtip": "",

"used_by": "",

"used_by_display": ""

},

{

"id": 1742,

"obj_id": 1742,

"display_name": "Behaviour Analysis Stage, Other",

"name": "Behaviour Analysis Stage, Other",

"type": "Text Field",

"values": "",

"created_at": "10/17/2013",

"updated_at": "11/13/2014",

"created_by": [redacted],

"searchable_quick": false,

"searchable_advanced": true,

"searchable_batch": false,

"searchable": "Advanced",

"pii": "No",

"qtip": "",

"used_by": "",

"used_by_display": ""

},

{

"id": 495,

"obj_id": 495,

"display_name": "Best_index_samples_table",

"name": "Best_index_samples_table",

"type": "Text Area",

"values": "",

"created_at": "05/07/2013",

"updated_at": "11/13/2014",

"created_by": [redacted],

"searchable_quick": false,

"searchable_advanced": false,

"searchable_batch": false,

"searchable": "",

"pii": "No",

"qtip": "",

"used_by": "Labmatrix Legacy - SNP Chip Non UDP, Labmatrix Legacy - SNP Chip",

"used_by_display": "<a href='#' onClick='remoteJS({name:\"dialogs/AddSubjectTypeDlg\", obj_id:99 });'>Labmatrix Legacy - SNP Chip Non UDP</a>, <a href='#' onClick='remoteJS({name:\"dialogs/AddSubjectTypeDlg\", obj_id:105 });'>Labmatrix Legacy - SNP Chip</a>"

},

{

"id": 2415,

"obj_id": 2415,

"display_name": "Biobanking Protocol",

"name": "Biobanking Protocol",

"type": "Subject",

"values": "",

"created_at": "03/18/2014",

"updated_at": "03/18/2014",

"created_by": [redacted],

"searchable_quick": true,

"searchable_advanced": true,

"searchable_batch": true,

"searchable": "Quick,Advanced,Batch",

"pii": "No",

"qtip": "",

"used_by": "DNA Extraction, Cell Culture, RNA Extraction, Plasma Extraction, Urine Pellet, Urine, Serum Extraction, CSF Processing",

"used_by_display": "<a href='#' onClick='remoteJS({name:\"dialogs/AddSubjectTypeDlg\", obj_id:122 });'>DNA Extraction</a>, <a href='#' onClick='remoteJS({name:\"dialogs/AddSubjectTypeDlg\", obj_id:40 });'>Cell Culture</a>, <a href='#' onClick='remoteJS({name:\"dialogs/AddSubjectTypeDlg\", obj_id:43 });'>RNA Extraction</a>, <a href='#' onClick='remoteJS({name:\"dialogs/AddSubjectTypeDlg\", obj_id:47 });'>Plasma Extraction</a>, <a href='#' onClick='remoteJS({name:\"dialogs/AddSubjectTypeDlg\", obj_id:170 });'>Urine Pellet</a>, <a href='#' onClick='remoteJS({name:\"dialogs/AddSubjectTypeDlg\", obj_id:120 });'>Urine</a>, <a href='#' onClick='remoteJS({name:\"dialogs/AddSubjectTypeDlg\", obj_id:119 });'>Serum Extraction</a>, <a href='#' onClick='remoteJS({name:\"dialogs/AddSubjectTypeDlg\", obj_id:117 });'>CSF Processing</a>"

},

{

"id": 2409,

"obj_id": 2409,

"display_name": "Biochemical CLIA Validation",

"name": "Biochemical CLIA Validation",

"type": "File",

"values": "",

"created_at": "03/14/2014",

"updated_at": "12/10/2014",

"created_by": [redacted],

"searchable_quick": false,

"searchable_advanced": true,

"searchable_batch": false,

"searchable": "Advanced",

"pii": "Yes",

"qtip": "",

"used_by": "Diagnosis",

"used_by_display": "<a href='#' onClick='remoteJS({name:\"dialogs/AddSubjectTypeDlg\", obj_id:185 });'>Diagnosis</a>"

},

{

"id": 2933,

"obj_id": 2933,

"display_name": "Bioinformatics File",

"name": "Bioinformatics File",

"type": "Subject",

"values": "",

"created_at": "12/08/2014",

"updated_at": "12/08/2014",

"created_by": [redacted],

"searchable_quick": false,

"searchable_advanced": true,

"searchable_batch": false,

"searchable": "Advanced",

"pii": "No",

"qtip": "",

"used_by": "",

"used_by_display": ""

},

{

"id": 2685,

"obj_id": 2685,

"display_name": "Bioinformatics Files",

"name": "Bioinformatics Files",

"type": "Subject Back Ref",

"values": "",

"created_at": "06/18/2014",

"updated_at": "06/18/2014",

"created_by": [redacted],

"searchable_quick": false,

"searchable_advanced": null,

"searchable_batch": null,

"searchable": "",

"pii": "No",

"qtip": "",

"used_by": "Patient, Patient Follow Up Visit",

"used_by_display": "<a href='#' onClick='remoteJS({name:\"dialogs/AddSubjectTypeDlg\", obj_id:1 });'>Patient</a>, <a href='#' onClick='remoteJS({name:\"dialogs/AddSubjectTypeDlg\", obj_id:264 });'>Patient Follow Up Visit</a>"

},

{

"id": 2567,

"obj_id": 2567,

"display_name": "Bioinformatics Files Received",

"name": "Bioinformatics Files Received",

"type": "Subject Back Ref",

"values": "",

"created_at": "04/28/2014",

"updated_at": "06/18/2014",

"created_by": [redacted],

"searchable_quick": false,

"searchable_advanced": null,

"searchable_batch": null,

"searchable": "",

"pii": "No",

"qtip": "",

"used_by": "Information Technology Collaboration",

"used_by_display": "<a href='#' onClick='remoteJS({name:\"dialogs/AddSubjectTypeDlg\", obj_id:183 });'>Information Technology Collaboration</a>"

},

{

"id": 496,

"obj_id": 496,

"display_name": "Biomaterial ID",

"name": "Biomaterial ID",

"type": "Number",

"values": "",

"created_at": "05/07/2013",

"updated_at": "11/13/2014",

"created_by": [redacted],

"searchable_quick": false,

"searchable_advanced": true,

"searchable_batch": false,

"searchable": "Advanced",

"pii": "No",

"qtip": "",

"used_by": "Labmatrix Legacy - Biomaterials, Labmatrix Legacy - Workflows",

"used_by_display": "<a href='#' onClick='remoteJS({name:\"dialogs/AddSubjectTypeDlg\", obj_id:91 });'>Labmatrix Legacy - Biomaterials</a>, <a href='#' onClick='remoteJS({name:\"dialogs/AddSubjectTypeDlg\", obj_id:113 });'>Labmatrix Legacy - Workflows</a>"

},

{

"id": 1810,

"obj_id": 1810,

"display_name": "Biomaterials - Labmatrix Legacy",

"name": "Biomaterials - Labmatrix Legacy",

"type": "Subject Back Ref",

"values": "",

"created_at": "10/29/2013",

"updated_at": "10/29/2013",

"created_by": [redacted],

"searchable_quick": false,

"searchable_advanced": null,

"searchable_batch": null,

"searchable": "",

"pii": "No",

"qtip": "",

"used_by": "Patient, Patient Follow Up Visit",

"used_by_display": "<a href='#' onClick='remoteJS({name:\"dialogs/AddSubjectTypeDlg\", obj_id:1 });'>Patient</a>, <a href='#' onClick='remoteJS({name:\"dialogs/AddSubjectTypeDlg\", obj_id:264 });'>Patient Follow Up Visit</a>"

},

{

"id": 2322,

"obj_id": 2322,

"display_name": "Birth:",

"name": "Birth:",

"type": "Text Area",

"values": "",

"created_at": "03/04/2014",

"updated_at": "11/13/2014",

"created_by": [redacted],

"searchable_quick": false,

"searchable_advanced": false,

"searchable_batch": false,

"searchable": "",

"pii": "Yes",

"qtip": "",

"used_by": "Patient Review",

"used_by_display": "<a href='#' onClick='remoteJS({name:\"dialogs/AddSubjectTypeDlg\", obj_id:83 });'>Patient Review</a>"

},

{

"id": 497,

"obj_id": 497,

"display_name": "Birth Place - City",

"name": "Birth Place - City",

"type": "Text Field",

"values": "",

"created_at": "05/07/2013",

"updated_at": "11/13/2014",

"created_by": [redacted],

"searchable_quick": false,

"searchable_advanced": true,

"searchable_batch": false,

"searchable": "Advanced",

"pii": "Yes",

"qtip": "",

"used_by": "Patient, Labmatrix Legacy - Subjects, Patient Follow Up Visit",

"used_by_display": "<a href='#' onClick='remoteJS({name:\"dialogs/AddSubjectTypeDlg\", obj_id:1 });'>Patient</a>, <a href='#' onClick='remoteJS({name:\"dialogs/AddSubjectTypeDlg\", obj_id:112 });'>Labmatrix Legacy - Subjects</a>, <a href='#' onClick='remoteJS({name:\"dialogs/AddSubjectTypeDlg\", obj_id:264 });'>Patient Follow Up Visit</a>"

},

{

"id": 498,

"obj_id": 498,

"display_name": "Birth Place - Country",

"name": "Birth Place - Country",

"type": "Text Field",

"values": "",

"created_at": "05/07/2013",

"updated_at": "11/13/2014",

"created_by": [redacted],

"searchable_quick": false,

"searchable_advanced": true,

"searchable_batch": false,

"searchable": "Advanced",

"pii": "Yes",

"qtip": "",

"used_by": "Patient, Labmatrix Legacy - Subjects, Patient Follow Up Visit",

"used_by_display": "<a href='#' onClick='remoteJS({name:\"dialogs/AddSubjectTypeDlg\", obj_id:1 });'>Patient</a>, <a href='#' onClick='remoteJS({name:\"dialogs/AddSubjectTypeDlg\", obj_id:112 });'>Labmatrix Legacy - Subjects</a>, <a href='#' onClick='remoteJS({name:\"dialogs/AddSubjectTypeDlg\", obj_id:264 });'>Patient Follow Up Visit</a>"

},

{

"id": 499,

"obj_id": 499,

"display_name": "Birth Place - State/Province",

"name": "Birth Place - State/Province",

"type": "Text Field",

"values": "",

"created_at": "05/07/2013",

"updated_at": "11/13/2014",

"created_by": [redacted],

"searchable_quick": false,

"searchable_advanced": true,

"searchable_batch": false,

"searchable": "Advanced",

"pii": "Yes",

"qtip": "",

"used_by": "Patient, Labmatrix Legacy - Subjects, Patient Follow Up Visit",

"used_by_display": "<a href='#' onClick='remoteJS({name:\"dialogs/AddSubjectTypeDlg\", obj_id:1 });'>Patient</a>, <a href='#' onClick='remoteJS({name:\"dialogs/AddSubjectTypeDlg\", obj_id:112 });'>Labmatrix Legacy - Subjects</a>, <a href='#' onClick='remoteJS({name:\"dialogs/AddSubjectTypeDlg\", obj_id:264 });'>Patient Follow Up Visit</a>"

},

{

"id": 2589,

"obj_id": 2589,

"display_name": "Blank consent form",

"name": "Blank consent form",

"type": "File",

"values": "",

"created_at": "05/02/2014",

"updated_at": "05/02/2014",

"created_by": [redacted],

"searchable_quick": false,

"searchable_advanced": null,

"searchable_batch": null,

"searchable": "",

"pii": "No",

"qtip": "",

"used_by": "Consent Versions",

"used_by_display": "<a href='#' onClick='remoteJS({name:\"dialogs/AddSubjectTypeDlg\", obj_id:239 });'>Consent Versions</a>"

},

{

"id": 2571,

"obj_id": 2571,

"display_name": "Blood collection and sharing",

"name": "Blood collection and sharing",

"type": "Choice",

"values": "Yes, No",

"created_at": "05/01/2014",

"updated_at": "11/13/2014",

"created_by": [redacted],

"searchable_quick": false,

"searchable_advanced": true,

"searchable_batch": false,

"searchable": "Advanced",

"pii": "No",

"qtip": "",

"used_by": "Consent Versions",

"used_by_display": "<a href='#' onClick='remoteJS({name:\"dialogs/AddSubjectTypeDlg\", obj_id:239 });'>Consent Versions</a>"

},

{

"id": 1251,

"obj_id": 1251,

"display_name": "Blood Draws for Available Family Members",

"name": "Blood Draws for Available Family Members",

"type": "Radio Buttons",

"values": "Yes, No",

"created_at": "06/03/2013",

"updated_at": "09/19/2013",

"created_by": [redacted],

"searchable_quick": false,

"searchable_advanced": null,

"searchable_batch": null,

"searchable": "",

"pii": "No",

"qtip": "",

"used_by": "NIH Visit",

"used_by_display": "<a href='#' onClick='remoteJS({name:\"dialogs/AddSubjectTypeDlg\", obj_id:140 });'>NIH Visit</a>"

},

{

"id": 421,

"obj_id": 421,

"display_name": "Blood Kit",

"name": "Blood Kit",

"type": "Subject",

"values": "",

"created_at": "04/17/2013",

"updated_at": "10/29/2014",

"created_by": [redacted],

"searchable_quick": false,

"searchable_advanced": true,

"searchable_batch": false,

"searchable": "Advanced",

"pii": "No",

"qtip": "",

"used_by": "Research Sample, DNA Extraction, Serum Extraction, Plasma Extraction, RNA Extraction",

"used_by_display": "<a href='#' onClick='remoteJS({name:\"dialogs/AddSubjectTypeDlg\", obj_id:86 });'>Research Sample</a>, <a href='#' onClick='remoteJS({name:\"dialogs/AddSubjectTypeDlg\", obj_id:122 });'>DNA Extraction</a>, <a href='#' onClick='remoteJS({name:\"dialogs/AddSubjectTypeDlg\", obj_id:119 });'>Serum Extraction</a>, <a href='#' onClick='remoteJS({name:\"dialogs/AddSubjectTypeDlg\", obj_id:47 });'>Plasma Extraction</a>, <a href='#' onClick='remoteJS({name:\"dialogs/AddSubjectTypeDlg\", obj_id:43 });'>RNA Extraction</a>"

},

{

"id": 2081,

"obj_id": 2081,

"display_name": "Blood Kits",

"name": "Blood Kits",

"type": "Subject Back Ref",

"values": "",

"created_at": "12/12/2013",

"updated_at": "10/29/2014",

"created_by": [redacted],

"searchable_quick": false,

"searchable_advanced": true,

"searchable_batch": false,

"searchable": "Advanced",

"pii": "No",

"qtip": "",

"used_by": "Patient, Patient Follow Up Visit",

"used_by_display": "<a href='#' onClick='remoteJS({name:\"dialogs/AddSubjectTypeDlg\", obj_id:1 });'>Patient</a>, <a href='#' onClick='remoteJS({name:\"dialogs/AddSubjectTypeDlg\", obj_id:264 });'>Patient Follow Up Visit</a>"

},

{

"id": 500,

"obj_id": 500,

"display_name": "Blood_kit_sent",

"name": "Blood_kit_sent",

"type": "Text Field",

"values": "",

"created_at": "05/07/2013",

"updated_at": "12/10/2014",

"created_by": [redacted],

"searchable_quick": false,

"searchable_advanced": true,

"searchable_batch": false,

"searchable": "Advanced",

"pii": "No",

"qtip": "",

"used_by": "Labmatrix Legacy - FEDEX Specimen Kit Tracking",

"used_by_display": "<a href='#' onClick='remoteJS({name:\"dialogs/AddSubjectTypeDlg\", obj_id:103 });'>Labmatrix Legacy - FEDEX Specimen Kit Tracking</a>"

},

{

"id": 1024,

"obj_id": 1024,

"display_name": "Blood Sample Spun Down",

"name": "Blood Sample Spun Down",

"type": "CheckBox",

"values": "",

"created_at": "05/14/2013",

"updated_at": "09/19/2013",

"created_by": [redacted],

"searchable_quick": false,

"searchable_advanced": null,

"searchable_batch": null,

"searchable": "",

"pii": "No",

"qtip": "980g for 10 mins @ 18-20C",

"used_by": "Plasma Extraction, Serum Extraction, DNA Extraction",

"used_by_display": "<a href='#' onClick='remoteJS({name:\"dialogs/AddSubjectTypeDlg\", obj_id:47 });'>Plasma Extraction</a>, <a href='#' onClick='remoteJS({name:\"dialogs/AddSubjectTypeDlg\", obj_id:119 });'>Serum Extraction</a>, <a href='#' onClick='remoteJS({name:\"dialogs/AddSubjectTypeDlg\", obj_id:122 });'>DNA Extraction</a>"

},

{

"id": 1505,

"obj_id": 1505,

"display_name": "BLOSUM62 Score",

"name": "BLOSUM62 Score",

"type": "Text Field",

"values": "",

"created_at": "07/29/2013",

"updated_at": "07/29/2013",

"created_by": [redacted],

"searchable_quick": false,

"searchable_advanced": null,

"searchable_batch": null,

"searchable": "",

"pii": "No",

"qtip": "",

"used_by": "Exome Analysis Results",

"used_by_display": "<a href='#' onClick='remoteJS({name:\"dialogs/AddSubjectTypeDlg\", obj_id:156 });'>Exome Analysis Results</a>"

},

{

"id": 2107,

"obj_id": 2107,

"display_name": "Bp Inserted/Deleted",

"name": "Bp Inserted/Deleted",

"type": "Text Area",

"values": "",

"created_at": "12/17/2013",

"updated_at": "01/06/2014",

"created_by": [redacted],

"searchable_quick": false,

"searchable_advanced": null,

"searchable_batch": null,

"searchable": "",

"pii": "No",

"qtip": "",

"used_by": "Zebrafish Mutation Project",

"used_by_display": "<a href='#' onClick='remoteJS({name:\"dialogs/AddSubjectTypeDlg\", obj_id:186 });'>Zebrafish Mutation Project</a>"

},

{

"id": 2741,

"obj_id": 2741,

"display_name": "Breeding Notes",

"name": "Breeding Notes",

"type": "Text Area",

"values": "",

"created_at": "07/18/2014",

"updated_at": "07/18/2014",

"created_by": [redacted],

"searchable_quick": false,

"searchable_advanced": false,

"searchable_batch": false,

"searchable": "",

"pii": "No",

"qtip": "",

"used_by": "Zebrafish Mutation Project, Zebrafish Breeding Information",

"used_by_display": "<a href='#' onClick='remoteJS({name:\"dialogs/AddSubjectTypeDlg\", obj_id:186 });'>Zebrafish Mutation Project</a>, <a href='#' onClick='remoteJS({name:\"dialogs/AddSubjectTypeDlg\", obj_id:233 });'>Zebrafish Breeding Information</a>"

},

{

"id": 2040,

"obj_id": 2040,

"display_name": "Buffer",

"name": "Buffer",

"type": "Choice",

"values": "TE, Water",

"created_at": "12/02/2013",

"updated_at": "11/13/2014",

"created_by": [redacted],

"searchable_quick": false,

"searchable_advanced": true,

"searchable_batch": false,

"searchable": "Advanced",

"pii": "No",

"qtip": "Axeq Submission Form",

"used_by": "Exome Sequencing, Genome Sequencing",

"used_by_display": "<a href='#' onClick='remoteJS({name:\"dialogs/AddSubjectTypeDlg\", obj_id:161 });'>Exome Sequencing</a>, <a href='#' onClick='remoteJS({name:\"dialogs/AddSubjectTypeDlg\", obj_id:212 });'>Genome Sequencing</a>"

},

{

"id": 2499,

"obj_id": 2499,

"display_name": "Buffy Coat Pellets",

"name": "Buffy Coat Pellets",

"type": "Subject Back Ref",

"values": "",

"created_at": "04/09/2014",

"updated_at": "10/29/2014",

"created_by": [redacted],

"searchable_quick": false,

"searchable_advanced": true,

"searchable_batch": false,

"searchable": "Advanced",

"pii": "No",

"qtip": "",

"used_by": "Research Sample",

"used_by_display": "<a href='#' onClick='remoteJS({name:\"dialogs/AddSubjectTypeDlg\", obj_id:86 });'>Research Sample</a>"

},

{

"id": 1277,

"obj_id": 1277,

"display_name": "Build",

"name": "Build",

"type": "Choice",

"values": "HG18, HG19, HG38",

"created_at": "06/04/2013",

"updated_at": "11/13/2014",

"created_by": [redacted],

"searchable_quick": false,

"searchable_advanced": true,

"searchable_batch": false,

"searchable": "Advanced",

"pii": "No",

"qtip": "",

"used_by": "SNP Chip Sequencing, Sanger Interpretation, Variant Prioritization, SNP Analysis, Bedfiles, Sanger Sequencing Information, Exome Analysis Results, Exome Analysis",

"used_by_display": "<a href='#' onClick='remoteJS({name:\"dialogs/AddSubjectTypeDlg\", obj_id:203 });'>SNP Chip Sequencing</a>, <a href='#' onClick='remoteJS({name:\"dialogs/AddSubjectTypeDlg\", obj_id:22 });'>Sanger Interpretation</a>, <a href='#' onClick='remoteJS({name:\"dialogs/AddSubjectTypeDlg\", obj_id:279 });'>Variant Prioritization</a>, <a href='#' onClick='remoteJS({name:\"dialogs/AddSubjectTypeDlg\", obj_id:59 });'>SNP Analysis</a>, <a href='#' onClick='remoteJS({name:\"dialogs/AddSubjectTypeDlg\", obj_id:238 });'>Bedfiles</a>, <a href='#' onClick='remoteJS({name:\"dialogs/AddSubjectTypeDlg\", obj_id:221 });'>Sanger Sequencing Information</a>, <a href='#' onClick='remoteJS({name:\"dialogs/AddSubjectTypeDlg\", obj_id:156 });'>Exome Analysis Results</a>, <a href='#' onClick='remoteJS({name:\"dialogs/AddSubjectTypeDlg\", obj_id:60 });'>Exome Analysis</a>"

},

{

"id": 2314,

"obj_id": 2314,

"display_name": "[redacted] Review Needed",

"name": "[redacted] Review Needed",

"type": "Choice",

"values": "Yes, No",

"created_at": "02/28/2014",

"updated_at": "02/28/2014",

"created_by": [redacted],

"searchable_quick": true,

"searchable_advanced": true,

"searchable_batch": true,

"searchable": "Quick,Advanced,Batch",

"pii": "No",

"qtip": "",

"used_by": "Patient, NIH Visit, Patient Follow Up Visit",

"used_by_display": "<a href='#' onClick='remoteJS({name:\"dialogs/AddSubjectTypeDlg\", obj_id:1 });'>Patient</a>, <a href='#' onClick='remoteJS({name:\"dialogs/AddSubjectTypeDlg\", obj_id:140 });'>NIH Visit</a>, <a href='#' onClick='remoteJS({name:\"dialogs/AddSubjectTypeDlg\", obj_id:264 });'>Patient Follow Up Visit</a>"

},

{

"id": 2990,

"obj_id": 2990,

"display_name": "CADD score",

"name": "CADD score",

"type": "Number",

"values": "",

"created_at": "01/23/2015",

"updated_at": "01/23/2015",

"created_by": [redacted],

"searchable_quick": false,

"searchable_advanced": true,

"searchable_batch": false,

"searchable": "Advanced",

"pii": "No",

"qtip": "",

"used_by": "Exome Analysis Results",

"used_by_display": "<a href='#' onClick='remoteJS({name:\"dialogs/AddSubjectTypeDlg\", obj_id:156 });'>Exome Analysis Results</a>"

},

{

"id": 2296,

"obj_id": 2296,

"display_name": "CADD Score",

"name": "CADD Score",

"type": "Text Field",

"values": "",

"created_at": "02/26/2014",

"updated_at": "11/13/2014",

"created_by": [redacted],

"searchable_quick": false,

"searchable_advanced": true,

"searchable_batch": false,

"searchable": "Advanced",

"pii": "No",

"qtip": "Combined annotation dependent deletion score",

"used_by": "Exome Analysis Results",

"used_by_display": "<a href='#' onClick='remoteJS({name:\"dialogs/AddSubjectTypeDlg\", obj_id:156 });'>Exome Analysis Results</a>"

},

{

"id": 2527,

"obj_id": 2527,

"display_name": "Capture Kit",

"name": "Capture Kit",

"type": "Text Field",

"values": "",

"created_at": "04/15/2014",

"updated_at": "11/13/2014",

"created_by": [redacted],

"searchable_quick": false,

"searchable_advanced": true,

"searchable_batch": false,

"searchable": "Advanced",

"pii": "No",

"qtip": "",

"used_by": "Patient Bioinformatics Files",

"used_by_display": "<a href='#' onClick='remoteJS({name:\"dialogs/AddSubjectTypeDlg\", obj_id:235 });'>Patient Bioinformatics Files</a>"

},

{

"id": 1543,

"obj_id": 1543,

"display_name": "Cardio Notes",

"name": "Cardio Notes",

"type": "Text Area",

"values": "",

"created_at": "08/07/2013",

"updated_at": "06/10/2014",

"created_by": [redacted],

"searchable_quick": false,

"searchable_advanced": null,

"searchable_batch": null,

"searchable": "",

"pii": "Yes",

"qtip": "",

"used_by": "Patient Visit Tools, Patient Visit Planning for Version C",

"used_by_display": "<a href='#' onClick='remoteJS({name:\"dialogs/AddSubjectTypeDlg\", obj_id:163 });'>Patient Visit Tools</a>, <a href='#' onClick='remoteJS({name:\"dialogs/AddSubjectTypeDlg\", obj_id:192 });'>Patient Visit Planning for Version C</a>"

},

{

"id": 1195,

"obj_id": 1195,

"display_name": "Cardiovascular Assessment Complete?",

"name": "Cardiovascular Assessment Complete?",

"type": "Radio Buttons",

"values": "Yes, No",

"created_at": "05/29/2013",

"updated_at": "09/19/2013",

"created_by": [redacted],

"searchable_quick": false,

"searchable_advanced": null,

"searchable_batch": null,

"searchable": "",

"pii": "No",

"qtip": "",

"used_by": "Patient Visit Tools, Patient Visit Planning for Version C",

"used_by_display": "<a href='#' onClick='remoteJS({name:\"dialogs/AddSubjectTypeDlg\", obj_id:163 });'>Patient Visit Tools</a>, <a href='#' onClick='remoteJS({name:\"dialogs/AddSubjectTypeDlg\", obj_id:192 });'>Patient Visit Planning for Version C</a>"

},

{

"id": 501,

"obj_id": 501,

"display_name": "Carrier Tracking Number",

"name": "Carrier Tracking Number",

"type": "Text Field",

"values": "",

"created_at": "05/07/2013",

"updated_at": "11/13/2014",

"created_by": [redacted],

"searchable_quick": false,

"searchable_advanced": true,

"searchable_batch": false,

"searchable": "Advanced",

"pii": "No",

"qtip": "",

"used_by": "Labmatrix Legacy - Communications",

"used_by_display": "<a href='#' onClick='remoteJS({name:\"dialogs/AddSubjectTypeDlg\", obj_id:92 });'>Labmatrix Legacy - Communications</a>"

},

{

"id": 2467,

"obj_id": 2467,

"display_name": "Cas9 mRNA Concentration (ng/ul)",

"name": "Cas9 mRNA Concentration (ng/ul)",

"type": "Text Field",

"values": "",

"created_at": "03/28/2014",

"updated_at": "11/13/2014",

"created_by": [redacted],

"searchable_quick": false,

"searchable_advanced": true,

"searchable_batch": false,

"searchable": "Advanced",

"pii": "No",

"qtip": "",

"used_by": "Zebrafish Mutation Project",

"used_by_display": "<a href='#' onClick='remoteJS({name:\"dialogs/AddSubjectTypeDlg\", obj_id:186 });'>Zebrafish Mutation Project</a>"

},

{

"id": 2398,

"obj_id": 2398,

"display_name": "Cas9 mRNA Concentration (ng/uL)",

"name": "Cas9 mRNA Concentration (ng/uL)",

"type": "Number",

"values": "",

"created_at": "03/14/2014",

"updated_at": "03/14/2014",

"created_by": [redacted],

"searchable_quick": false,

"searchable_advanced": null,

"searchable_batch": null,

"searchable": "",

"pii": "No",

"qtip": "",

"used_by": "Zebrafish Mutation Project",

"used_by_display": "<a href='#' onClick='remoteJS({name:\"dialogs/AddSubjectTypeDlg\", obj_id:186 });'>Zebrafish Mutation Project</a>"

},

{

"id": 2248,

"obj_id": 2248,

"display_name": "Cas9 mRNA Synthesized",

"name": "Cas9 mRNA Synthesized",

"type": "Choice",

"values": "Yes, No",

"created_at": "02/10/2014",

"updated_at": "11/13/2014",

"created_by": [redacted],

"searchable_quick": false,

"searchable_advanced": true,

"searchable_batch": false,

"searchable": "Advanced",

"pii": "No",

"qtip": "",

"used_by": "Zebrafish Mutation Project",

"used_by_display": "<a href='#' onClick='remoteJS({name:\"dialogs/AddSubjectTypeDlg\", obj_id:186 });'>Zebrafish Mutation Project</a>"

},

{

"id": 2400,

"obj_id": 2400,

"display_name": "Cas9 Plasmid",

"name": "Cas9 Plasmid",

"type": "Text Field",

"values": "",

"created_at": "03/14/2014",

"updated_at": "12/23/2014",

"created_by": [redacted],

"searchable_quick": true,

"searchable_advanced": true,

"searchable_batch": true,

"searchable": "Quick,Advanced,Batch",

"pii": "No",

"qtip": "Plasmid used for mRNA synthesis",

"used_by": "Zebrafish Mutation Project",

"used_by_display": "<a href='#' onClick='remoteJS({name:\"dialogs/AddSubjectTypeDlg\", obj_id:186 });'>Zebrafish Mutation Project</a>"

},

{

"id": 1792,

"obj_id": 1792,

"display_name": "Catalog Number",

"name": "Catalog Number",

"type": "Text Field",

"values": "",

"created_at": "10/24/2013",

"updated_at": "10/24/2013",

"created_by": [redacted],

"searchable_quick": false,

"searchable_advanced": null,

"searchable_batch": null,

"searchable": "",

"pii": "No",

"qtip": "",

"used_by": "iPS Cells, Plasmid Reagents, Antibody",

"used_by_display": "<a href='#' onClick='remoteJS({name:\"dialogs/AddSubjectTypeDlg\", obj_id:41 });'>iPS Cells</a>, <a href='#' onClick='remoteJS({name:\"dialogs/AddSubjectTypeDlg\", obj_id:177 });'>Plasmid Reagents</a>, <a href='#' onClick='remoteJS({name:\"dialogs/AddSubjectTypeDlg\", obj_id:189 });'>Antibody</a>"

},

{

"id": 1235,

"obj_id": 1235,

"display_name": "Category",

"name": "Category",

"type": "Choice",

"values": "Acceptance Letter, Review, Respiratory, Neurological, Nutritional, Cardiovascular, Rehabilitation and Mobility, Other",

"created_at": "06/03/2013",

"updated_at": "07/26/2013",

"created_by": [redacted],

"searchable_quick": false,

"searchable_advanced": null,

"searchable_batch": null,

"searchable": "",

"pii": "No",

"qtip": "",

"used_by": "NIH Visit, Patient Visit Tools, Patient Visit Planning for Version C",

"used_by_display": "<a href='#' onClick='remoteJS({name:\"dialogs/AddSubjectTypeDlg\", obj_id:140 });'>NIH Visit</a>, <a href='#' onClick='remoteJS({name:\"dialogs/AddSubjectTypeDlg\", obj_id:163 });'>Patient Visit Tools</a>, <a href='#' onClick='remoteJS({name:\"dialogs/AddSubjectTypeDlg\", obj_id:192 });'>Patient Visit Planning for Version C</a>"

},

{

"id": 2942,

"obj_id": 2942,

"display_name": "Category Type",

"name": "Category Type",

"type": "Text Field",

"values": "",

"created_at": "12/10/2014",

"updated_at": "12/10/2014",

"created_by": [redacted],

"searchable_quick": true,

"searchable_advanced": true,

"searchable_batch": true,

"searchable": "Quick,Advanced,Batch",

"pii": "No",

"qtip": "",

"used_by": "",

"used_by_display": ""

},

{

"id": 502,

"obj_id": 502,

"display_name": "Cause of Death",

"name": "Cause of Death",

"type": "Text Field",

"values": "",

"created_at": "05/07/2013",

"updated_at": "11/13/2014",

"created_by": [redacted],

"searchable_quick": false,

"searchable_advanced": true,

"searchable_batch": false,

"searchable": "Advanced",

"pii": "Yes",

"qtip": "",

"used_by": "Patient, Labmatrix Legacy - Subjects, Patient Follow Up Visit",

"used_by_display": "<a href='#' onClick='remoteJS({name:\"dialogs/AddSubjectTypeDlg\", obj_id:1 });'>Patient</a>, <a href='#' onClick='remoteJS({name:\"dialogs/AddSubjectTypeDlg\", obj_id:112 });'>Labmatrix Legacy - Subjects</a>, <a href='#' onClick='remoteJS({name:\"dialogs/AddSubjectTypeDlg\", obj_id:264 });'>Patient Follow Up Visit</a>"

},

{

"id": 1961,

"obj_id": 1961,

"display_name": "cDNA Sequence File",

"name": "cDNA Sequence File",

"type": "File",

"values": "",

"created_at": "11/14/2013",

"updated_at": "06/05/2014",

"created_by": [redacted],

"searchable_quick": false,

"searchable_advanced": null,

"searchable_batch": null,

"searchable": "",

"pii": "No",

"qtip": "From vendor",

"used_by": "Destination Vector Construction, Entry Vector, Clone",

"used_by_display": "<a href='#' onClick='remoteJS({name:\"dialogs/AddSubjectTypeDlg\", obj_id:168 });'>Destination Vector Construction</a>, <a href='#' onClick='remoteJS({name:\"dialogs/AddSubjectTypeDlg\", obj_id:193 });'>Entry Vector</a>, <a href='#' onClick='remoteJS({name:\"dialogs/AddSubjectTypeDlg\", obj_id:266 });'>Clone</a>"

},

{

"id": 1966,

"obj_id": 1966,

"display_name": "cDNA Species",

"name": "cDNA Species",

"type": "Choice",

"values": "Human, Yeast, Drosophila, Zebrafish, Mouse",

"created_at": "11/17/2013",

"updated_at": "11/13/2014",

"created_by": [redacted],

"searchable_quick": false,

"searchable_advanced": true,

"searchable_batch": false,

"searchable": "Advanced",

"pii": "No",

"qtip": "",

"used_by": "Entry Vector, Clone",

"used_by_display": "<a href='#' onClick='remoteJS({name:\"dialogs/AddSubjectTypeDlg\", obj_id:193 });'>Entry Vector</a>, <a href='#' onClick='remoteJS({name:\"dialogs/AddSubjectTypeDlg\", obj_id:266 });'>Clone</a>"

},

{

"id": 1480,

"obj_id": 1480,

"display_name": "CD Pred Score",

"name": "CD Pred Score",

"type": "Text Field",

"values": "",

"created_at": "07/22/2013",

"updated_at": "11/13/2014",

"created_by": [redacted],

"searchable_quick": false,

"searchable_advanced": true,

"searchable_batch": false,

"searchable": "Advanced",

"pii": "No",

"qtip": "",

"used_by": "Exome Analysis Results",

"used_by_display": "<a href='#' onClick='remoteJS({name:\"dialogs/AddSubjectTypeDlg\", obj_id:156 });'>Exome Analysis Results</a>"

},

{

"id": 503,

"obj_id": 503,

"display_name": "Cell Count",

"name": "Cell Count",

"type": "Text Field",

"values": "",

"created_at": "05/07/2013",

"updated_at": "11/13/2014",

"created_by": [redacted],

"searchable_quick": false,

"searchable_advanced": true,

"searchable_batch": false,

"searchable": "Advanced",

"pii": "No",

"qtip": "",

"used_by": "Labmatrix Legacy - Biomaterials, Collaborations, ChIP-Seq, RNA Extraction",

"used_by_display": "<a href='#' onClick='remoteJS({name:\"dialogs/AddSubjectTypeDlg\", obj_id:91 });'>Labmatrix Legacy - Biomaterials</a>, <a href='#' onClick='remoteJS({name:\"dialogs/AddSubjectTypeDlg\", obj_id:147 });'>Collaborations</a>, <a href='#' onClick='remoteJS({name:\"dialogs/AddSubjectTypeDlg\", obj_id:254 });'>ChIP-Seq</a>, <a href='#' onClick='remoteJS({name:\"dialogs/AddSubjectTypeDlg\", obj_id:43 });'>RNA Extraction</a>"

},

{

"id": 504,

"obj_id": 504,

"display_name": "Cell Count Units",

"name": "Cell Count Units",

"type": "Text Field",

"values": "",

"created_at": "05/07/2013",

"updated_at": "11/13/2014",

"created_by": [redacted],

"searchable_quick": false,

"searchable_advanced": true,

"searchable_batch": false,

"searchable": "Advanced",

"pii": "No",

"qtip": "",

"used_by": "Labmatrix Legacy - Biomaterials",

"used_by_display": "<a href='#' onClick='remoteJS({name:\"dialogs/AddSubjectTypeDlg\", obj_id:91 });'>Labmatrix Legacy - Biomaterials</a>"

},

{

"id": 2757,

"obj_id": 2757,

"display_name": "Cell culture",

"name": "Cell culture",

"type": "Subject",

"values": "",

"created_at": "08/01/2014",

"updated_at": "08/04/2014",

"created_by": [redacted],

"searchable_quick": false,

"searchable_advanced": false,

"searchable_batch": false,

"searchable": "",

"pii": "No",

"qtip": "",

"used_by": "RNA Extraction",

"used_by_display": "<a href='#' onClick='remoteJS({name:\"dialogs/AddSubjectTypeDlg\", obj_id:43 });'>RNA Extraction</a>"

},

{

"id": 2531,

"obj_id": 2531,

"display_name": "Cell Culture",

"name": "Cell Culture",

"type": "Subject Back Ref",

"values": "",

"created_at": "04/15/2014",

"updated_at": "04/15/2014",

"created_by": [redacted],

"searchable_quick": false,

"searchable_advanced": null,

"searchable_batch": null,

"searchable": "",

"pii": "No",

"qtip": "",

"used_by": "Patient, ChIP-Seq, Patient Follow Up Visit",

"used_by_display": "<a href='#' onClick='remoteJS({name:\"dialogs/AddSubjectTypeDlg\", obj_id:1 });'>Patient</a>, <a href='#' onClick='remoteJS({name:\"dialogs/AddSubjectTypeDlg\", obj_id:254 });'>ChIP-Seq</a>, <a href='#' onClick='remoteJS({name:\"dialogs/AddSubjectTypeDlg\", obj_id:264 });'>Patient Follow Up Visit</a>"

},

{

"id": 1670,

"obj_id": 1670,

"display_name": "Cells In One Vial",

"name": "Cells In One Vial",

"type": "Text Field",

"values": "",

"created_at": "08/26/2013",

"updated_at": "11/13/2014",

"created_by": [redacted],

"searchable_quick": false,

"searchable_advanced": true,

"searchable_batch": false,

"searchable": "Advanced",

"pii": "No",

"qtip": "",

"used_by": "Cell Culture, Rescue Cell Culture, Lymphoblast Culture Request",

"used_by_display": "<a href='#' onClick='remoteJS({name:\"dialogs/AddSubjectTypeDlg\", obj_id:40 });'>Cell Culture</a>, <a href='#' onClick='remoteJS({name:\"dialogs/AddSubjectTypeDlg\", obj_id:247 });'>Rescue Cell Culture</a>, <a href='#' onClick='remoteJS({name:\"dialogs/AddSubjectTypeDlg\", obj_id:272 });'>Lymphoblast Culture Request</a>"

},

{

"id": 2502,

"obj_id": 2502,

"display_name": "Cell Type",

"name": "Cell Type",

"type": "Choice",

"values": "Skin Fibroblast, Rescued Fibroblast, Rescued HELA, iPS cells, Culture media, Melanocyte, Osteoblast, HELA Cells, HEK 293 Cells, CHO, MEF, SH-SY5Y, Lymphoblast, NTERA2 cells, JURKAT, Cell Pellet",

"created_at": "04/09/2014",

"updated_at": "11/13/2014",

"created_by": [redacted],

"searchable_quick": false,

"searchable_advanced": true,

"searchable_batch": false,

"searchable": "Advanced",

"pii": "No",

"qtip": "",

"used_by": "Cell Culture, Rescue Cell Culture, ChIP-Seq",

"used_by_display": "<a href='#' onClick='remoteJS({name:\"dialogs/AddSubjectTypeDlg\", obj_id:40 });'>Cell Culture</a>, <a href='#' onClick='remoteJS({name:\"dialogs/AddSubjectTypeDlg\", obj_id:247 });'>Rescue Cell Culture</a>, <a href='#' onClick='remoteJS({name:\"dialogs/AddSubjectTypeDlg\", obj_id:254 });'>ChIP-Seq</a>"

},

{

"id": 2285,

"obj_id": 2285,

"display_name": "Center for CLIA Validation",

"name": "Center for CLIA Validation",

"type": "Subject",

"values": "",

"created_at": "02/20/2014",

"updated_at": "02/20/2014",

"created_by": [redacted],

"searchable_quick": true,

"searchable_advanced": true,

"searchable_batch": true,

"searchable": "Quick,Advanced,Batch",

"pii": "No",

"qtip": "Where DNA is sent for validation",

"used_by": "",

"used_by_display": ""

},

{

"id": 2545,

"obj_id": 2545,

"display_name": "CG#",

"name": "CG#",

"type": "Text Field",

"values": "",

"created_at": "04/21/2014",

"updated_at": "09/15/2014",

"created_by": [redacted],

"searchable_quick": null,

"searchable_advanced": null,

"searchable_batch": null,

"searchable": "",

"pii": "No",

"qtip": null,

"used_by": "Drosophila Study",

"used_by_display": "<a href='#' onClick='remoteJS({name:\"dialogs/AddSubjectTypeDlg\", obj_id:236 });'>Drosophila Study</a>"

},

{

"id": 1304,

"obj_id": 1304,

"display_name": "Changes in Volume/Sequence of Collection Tubes",

"name": "Changes in Volume/Sequence of Collection Tubes",

"type": "Choice",

"values": "Yes, No",

"created_at": "06/10/2013",

"updated_at": "10/29/2014",

"created_by": [redacted],

"searchable_quick": false,

"searchable_advanced": true,

"searchable_batch": false,

"searchable": "Advanced",

"pii": "No",

"qtip": "",

"used_by": "Research Sample",

"used_by_display": "<a href='#' onClick='remoteJS({name:\"dialogs/AddSubjectTypeDlg\", obj_id:86 });'>Research Sample</a>"

},

{

"id": 2734,

"obj_id": 2734,

"display_name": "Checklist Notes",

"name": "Checklist Notes",

"type": "Text Area",

"values": "",

"created_at": "07/18/2014",

"updated_at": "07/18/2014",

"created_by": [redacted],

"searchable_quick": false,

"searchable_advanced": false,

"searchable_batch": false,

"searchable": "",

"pii": "No",

"qtip": "",

"used_by": "Patient, Patient Follow Up Visit",

"used_by_display": "<a href='#' onClick='remoteJS({name:\"dialogs/AddSubjectTypeDlg\", obj_id:1 });'>Patient</a>, <a href='#' onClick='remoteJS({name:\"dialogs/AddSubjectTypeDlg\", obj_id:264 });'>Patient Follow Up Visit</a>"

},

{

"id": 1374,

"obj_id": 1374,

"display_name": "Check review for suggested tests",

"name": "Check review for suggested tests",

"type": "Radio Buttons",

"values": "Yes, No",

"created_at": "06/24/2013",

"updated_at": "09/19/2013",

"created_by": [redacted],

"searchable_quick": false,

"searchable_advanced": null,

"searchable_batch": null,

"searchable": "",

"pii": "No",

"qtip": "",

"used_by": "Patient Visit Tools, Patient Visit Planning for Version C",

"used_by_display": "<a href='#' onClick='remoteJS({name:\"dialogs/AddSubjectTypeDlg\", obj_id:163 });'>Patient Visit Tools</a>, <a href='#' onClick='remoteJS({name:\"dialogs/AddSubjectTypeDlg\", obj_id:192 });'>Patient Visit Planning for Version C</a>"

},

{

"id": 1569,

"obj_id": 1569,

"display_name": "Chief Complaint and Problem List",

"name": "Chief Complaint and Problem List",

"type": "Text Area",

"values": "",

"created_at": "08/12/2013",

"updated_at": "06/10/2014",

"created_by": [redacted],

"searchable_quick": false,

"searchable_advanced": null,

"searchable_batch": null,

"searchable": "",

"pii": "Yes",

"qtip": "",

"used_by": "Patient Review",

"used_by_display": "<a href='#' onClick='remoteJS({name:\"dialogs/AddSubjectTypeDlg\", obj_id:83 });'>Patient Review</a>"

},

{

"id": 2934,

"obj_id": 2934,

"display_name": "ChIP-Seq Services Report",

"name": "ChIP-Seq Services Report",

"type": "File",

"values": "",

"created_at": "12/08/2014",

"updated_at": "12/09/2014",

"created_by": [redacted],

"searchable_quick": false,

"searchable_advanced": true,

"searchable_batch": false,

"searchable": "Advanced",

"pii": "No",

"qtip": "",

"used_by": "ChIP-Seq",

"used_by_display": "<a href='#' onClick='remoteJS({name:\"dialogs/AddSubjectTypeDlg\", obj_id:254 });'>ChIP-Seq</a>"

},

{

"id": 2127,

"obj_id": 2127,

"display_name": "Chip Sequencing",

"name": "Chip Sequencing",

"type": "Subject",

"values": "",

"created_at": "12/30/2013",

"updated_at": "10/29/2014",

"created_by": [redacted],

"searchable_quick": false,

"searchable_advanced": true,

"searchable_batch": true,

"searchable": "Advanced,Batch",

"pii": "No",

"qtip": "Record with correct set, chip type, etc",

"used_by": "SNP Analysis, Phased and Imputed VCF, GenomeStudio Files",

"used_by_display": "<a href='#' onClick='remoteJS({name:\"dialogs/AddSubjectTypeDlg\", obj_id:59 });'>SNP Analysis</a>, <a href='#' onClick='remoteJS({name:\"dialogs/AddSubjectTypeDlg\", obj_id:188 });'>Phased and Imputed VCF</a>, <a href='#' onClick='remoteJS({name:\"dialogs/AddSubjectTypeDlg\", obj_id:202 });'>GenomeStudio Files</a>"

},

{

"id": 1387,

"obj_id": 1387,

"display_name": "Chip Type",

"name": "Chip Type",

"type": "Choice",

"values": "1Mduo, OmniQuad1M, OmniExpress12, OmniExpressExome, OmniExpressv1.2, OmniExpressExome1.1B, OmniExpressExome1.2A",

"created_at": "06/27/2013",

"updated_at": "11/13/2014",

"created_by": [redacted],

"searchable_quick": false,

"searchable_advanced": true,

"searchable_batch": false,

"searchable": "Advanced",

"pii": "No",

"qtip": "",

"used_by": "SNP Analysis, SNP Chip Sequencing",

"used_by_display": "<a href='#' onClick='remoteJS({name:\"dialogs/AddSubjectTypeDlg\", obj_id:59 });'>SNP Analysis</a>, <a href='#' onClick='remoteJS({name:\"dialogs/AddSubjectTypeDlg\", obj_id:203 });'>SNP Chip Sequencing</a>"

},

{

"id": 1474,

"obj_id": 1474,

"display_name": "Chr",

"name": "Chr",

"type": "Text Field",

"values": "",

"created_at": "07/22/2013",

"updated_at": "11/19/2014",

"created_by": [redacted],

"searchable_quick": false,

"searchable_advanced": true,

"searchable_batch": false,

"searchable": "Advanced",

"pii": "No",

"qtip": "",

"used_by": "Exome Analysis Results, Zebrafish Mutation Project, Variant Prioritization",

"used_by_display": "<a href='#' onClick='remoteJS({name:\"dialogs/AddSubjectTypeDlg\", obj_id:156 });'>Exome Analysis Results</a>, <a href='#' onClick='remoteJS({name:\"dialogs/AddSubjectTypeDlg\", obj_id:186 });'>Zebrafish Mutation Project</a>, <a href='#' onClick='remoteJS({name:\"dialogs/AddSubjectTypeDlg\", obj_id:279 });'>Variant Prioritization</a>"

},

{

"id": 2046,

"obj_id": 2046,

"display_name": "Chromatogram Image",

"name": "Chromatogram Image",

"type": "File",

"values": "",

"created_at": "12/03/2013",

"updated_at": "12/03/2013",

"created_by": [redacted],

"searchable_quick": false,

"searchable_advanced": null,

"searchable_batch": null,

"searchable": "",

"pii": "No",

"qtip": "",

"used_by": "Sanger Interpretation",

"used_by_display": "<a href='#' onClick='remoteJS({name:\"dialogs/AddSubjectTypeDlg\", obj_id:22 });'>Sanger Interpretation</a>"

},

{

"id": 2613,

"obj_id": 2613,

"display_name": "Chromosome Type",

"name": "Chromosome Type",

"type": "Choice",

"values": "Autosomal, X-linked, Holandric, Mitochondrial, Unmapped",

"created_at": "05/12/2014",

"updated_at": "09/25/2014",

"created_by": [redacted],

"searchable_quick": true,

"searchable_advanced": true,

"searchable_batch": true,

"searchable": "Quick,Advanced,Batch",

"pii": "No",

"qtip": "",

"used_by": "Exome Analysis Results",

"used_by_display": "<a href='#' onClick='remoteJS({name:\"dialogs/AddSubjectTypeDlg\", obj_id:156 });'>Exome Analysis Results</a>"

},

{

"id": 505,

"obj_id": 505,

"display_name": "City",

"name": "City",

"type": "Text Field",

"values": "",

"created_at": "05/07/2013",

"updated_at": "12/11/2014",

"created_by": [redacted],

"searchable_quick": true,

"searchable_advanced": true,

"searchable_batch": true,

"searchable": "Quick,Advanced,Batch",

"pii": "Yes",

"qtip": "",

"used_by": "Labmatrix Legacy - Organization Contacts, Patient Follow Up Visit, UDP Inquiry, Patient",

"used_by_display": "<a href='#' onClick='remoteJS({name:\"dialogs/AddSubjectTypeDlg\", obj_id:94 });'>Labmatrix Legacy - Organization Contacts</a>, <a href='#' onClick='remoteJS({name:\"dialogs/AddSubjectTypeDlg\", obj_id:264 });'>Patient Follow Up Visit</a>, <a href='#' onClick='remoteJS({name:\"dialogs/AddSubjectTypeDlg\", obj_id:61 });'>UDP Inquiry</a>, <a href='#' onClick='remoteJS({name:\"dialogs/AddSubjectTypeDlg\", obj_id:1 });'>Patient</a>"

},

{

"id": 506,

"obj_id": 506,

"display_name": "Class",

"name": "Class",

"type": "Text Field",

"values": "",

"created_at": "05/07/2013",

"updated_at": "09/19/2013",

"created_by": [redacted],

"searchable_quick": false,

"searchable_advanced": null,

"searchable_batch": null,

"searchable": "",

"pii": "No",

"qtip": "",

"used_by": "Labmatrix Legacy - Biomaterials",

"used_by_display": "<a href='#' onClick='remoteJS({name:\"dialogs/AddSubjectTypeDlg\", obj_id:91 });'>Labmatrix Legacy - Biomaterials</a>"

},

{

"id": 1242,

"obj_id": 1242,

"display_name": "Clearance to Travel by home team verified",

"name": "Clearance to Travel by home team verified",

"type": "Radio Buttons",

"values": "Yes, No",

"created_at": "06/03/2013",

"updated_at": "09/19/2013",

"created_by": [redacted],

"searchable_quick": false,

"searchable_advanced": null,

"searchable_batch": null,

"searchable": "",

"pii": "No",

"qtip": "",

"used_by": "Patient Visit Tools, Patient Visit Planning for Version C",

"used_by_display": "<a href='#' onClick='remoteJS({name:\"dialogs/AddSubjectTypeDlg\", obj_id:163 });'>Patient Visit Tools</a>, <a href='#' onClick='remoteJS({name:\"dialogs/AddSubjectTypeDlg\", obj_id:192 });'>Patient Visit Planning for Version C</a>"

},

{

"id": 2370,

"obj_id": 2370,

"display_name": "CLIA Back Reference",

"name": "CLIA Back Reference",

"type": "Subject Back Ref",

"values": "",

"created_at": "03/06/2014",

"updated_at": "11/24/2014",

"created_by": [redacted],

"searchable_quick": false,

"searchable_advanced": true,

"searchable_batch": false,

"searchable": "Advanced",

"pii": "No",

"qtip": "",

"used_by": "",

"used_by_display": ""

},

{

"id": 41,

"obj_id": 41,

"display_name": "CLIA/Sanger Validation Report",

"name": "CLIA/Sanger Validation Report",

"type": "File",

"values": "",

"created_at": "02/04/2013",

"updated_at": "11/24/2014",

"created_by": [redacted],

"searchable_quick": false,

"searchable_advanced": true,

"searchable_batch": false,

"searchable": "Advanced",

"pii": "Yes",

"qtip": "Final report for either CLIA or Sanger validation",

"used_by": "CLIA Validation",

"used_by_display": "<a href='#' onClick='remoteJS({name:\"dialogs/AddSubjectTypeDlg\", obj_id:15 });'>CLIA Validation</a>"

},

{

"id": 2446,

"obj_id": 2446,

"display_name": "CLIA Validation",

"name": "CLIA Validation",

"type": "Subject Back Ref",

"values": "",

"created_at": "03/25/2014",

"updated_at": "12/08/2014",

"created_by": [redacted],

"searchable_quick": false,

"searchable_advanced": true,

"searchable_batch": false,

"searchable": "Advanced",

"pii": "No",

"qtip": "",

"used_by": "Patient, Exome Analysis Results, UCSC Gene List, Patient Follow Up Visit",

"used_by_display": "<a href='#' onClick='remoteJS({name:\"dialogs/AddSubjectTypeDlg\", obj_id:1 });'>Patient</a>, <a href='#' onClick='remoteJS({name:\"dialogs/AddSubjectTypeDlg\", obj_id:156 });'>Exome Analysis Results</a>, <a href='#' onClick='remoteJS({name:\"dialogs/AddSubjectTypeDlg\", obj_id:199 });'>UCSC Gene List</a>, <a href='#' onClick='remoteJS({name:\"dialogs/AddSubjectTypeDlg\", obj_id:264 });'>Patient Follow Up Visit</a>"

},

{

"id": 1755,

"obj_id": 1755,

"display_name": "Clinical Interpretation of Test Results",

"name": "Clinical Interpretation of Test Results",

"type": "Text Area",

"values": "",

"created_at": "10/21/2013",

"updated_at": "01/23/2014",

"created_by": [redacted],

"searchable_quick": false,

"searchable_advanced": null,

"searchable_batch": null,

"searchable": "",

"pii": "Yes",

"qtip": "",

"used_by": "Requisition, PII - Patient File Uploads, Non - PII Patient File Uploads",

"used_by_display": "<a href='#' onClick='remoteJS({name:\"dialogs/AddSubjectTypeDlg\", obj_id:167 });'>Requisition</a>, <a href='#' onClick='remoteJS({name:\"dialogs/AddSubjectTypeDlg\", obj_id:172 });'>PII - Patient File Uploads</a>, <a href='#' onClick='remoteJS({name:\"dialogs/AddSubjectTypeDlg\", obj_id:201 });'>Non - PII Patient File Uploads</a>"

},

{

"id": 2840,

"obj_id": 2840,

"display_name": "Clinical Notes",

"name": "Clinical Notes",

"type": "Subject Back Ref",

"values": "",

"created_at": "09/15/2014",

"updated_at": "12/17/2014",

"created_by": [redacted],

"searchable_quick": true,

"searchable_advanced": true,

"searchable_batch": true,

"searchable": "Quick,Advanced,Batch",

"pii": "No",

"qtip": "",

"used_by": "Patient",

"used_by_display": "<a href='#' onClick='remoteJS({name:\"dialogs/AddSubjectTypeDlg\", obj_id:1 });'>Patient</a>"

},

{

"id": 234,

"obj_id": 234,

"display_name": "Clinical Questions",

"name": "Clinical Questions",

"type": "Text Area",

"values": "",

"created_at": "03/27/2013",

"updated_at": "06/10/2014",

"created_by": [redacted],

"searchable_quick": false,

"searchable_advanced": null,

"searchable_batch": null,

"searchable": "",

"pii": "Yes",

"qtip": "",

"used_by": "Patient Review",

"used_by_display": "<a href='#' onClick='remoteJS({name:\"dialogs/AddSubjectTypeDlg\", obj_id:83 });'>Patient Review</a>"

},

{

"id": 335,

"obj_id": 335,

"display_name": "Clinical Worksheet",

"name": "Clinical Worksheet",

"type": "HTML",

"values": "",

"created_at": "04/09/2013",

"updated_at": "09/16/2014",

"created_by": [redacted],

"searchable_quick": false,

"searchable_advanced": false,

"searchable_batch": false,

"searchable": "",

"pii": "Yes",

"qtip": "",

"used_by": "Patient, Patient Follow Up Visit",

"used_by_display": "<a href='#' onClick='remoteJS({name:\"dialogs/AddSubjectTypeDlg\", obj_id:1 });'>Patient</a>, <a href='#' onClick='remoteJS({name:\"dialogs/AddSubjectTypeDlg\", obj_id:264 });'>Patient Follow Up Visit</a>"

},

{

"id": 1502,

"obj_id": 1502,

"display_name": "Clinician (Primary)",

"name": "Clinician (Primary)",

"type": "Choice",

"values": [redacted],

"created_at": "07/29/2013",

"updated_at": "11/13/2014",

"created_by": [redacted],

"searchable_quick": false,

"searchable_advanced": true,

"searchable_batch": false,

"searchable": "Advanced",

"pii": "No",

"qtip": "",

"used_by": "Patient Visit Planning for Version C",

"used_by_display": "<a href='#' onClick='remoteJS({name:\"dialogs/AddSubjectTypeDlg\", obj_id:192 });'>Patient Visit Planning for Version C</a>"

},

{

"id": 2543,

"obj_id": 2543,

"display_name": "ClinSeq_maf",

"name": "ClinSeq_maf",

"type": "Text Field",

"values": "",

"created_at": "04/21/2014",

"updated_at": "04/21/2014",

"created_by": [redacted],

"searchable_quick": false,

"searchable_advanced": null,

"searchable_batch": null,

"searchable": "",

"pii": "No",

"qtip": "",

"used_by": "Exome Analysis Results",

"used_by_display": "<a href='#' onClick='remoteJS({name:\"dialogs/AddSubjectTypeDlg\", obj_id:156 });'>Exome Analysis Results</a>"

},

{

"id": 1915,

"obj_id": 1915,

"display_name": "Clone",

"name": "Clone",

"type": "Text Field",

"values": "",

"created_at": "11/08/2013",

"updated_at": "11/13/2014",

"created_by": [redacted],

"searchable_quick": false,

"searchable_advanced": true,

"searchable_batch": false,

"searchable": "Advanced",

"pii": "No",

"qtip": "",

"used_by": "Antibody",

"used_by_display": "<a href='#' onClick='remoteJS({name:\"dialogs/AddSubjectTypeDlg\", obj_id:189 });'>Antibody</a>"

},

{

"id": 2826,

"obj_id": 2826,

"display_name": "Clone Used",

"name": "Clone Used",

"type": "Subject",

"values": "",

"created_at": "09/11/2014",

"updated_at": "09/11/2014",

"created_by": [redacted],

"searchable_quick": false,

"searchable_advanced": false,

"searchable_batch": false,

"searchable": "",

"pii": "No",

"qtip": "",

"used_by": "Entry Vector",

"used_by_display": "<a href='#' onClick='remoteJS({name:\"dialogs/AddSubjectTypeDlg\", obj_id:193 });'>Entry Vector</a>"

},

{

"id": 1799,

"obj_id": 1799,

"display_name": "Cloning Method",

"name": "Cloning Method",

"type": "Choice",

"values": "TOPO, TOPO-TA, Recombination, Gateway Recombination, Restriction Digest",

"created_at": "10/28/2013",

"updated_at": "10/30/2014",

"created_by": [redacted],

"searchable_quick": true,

"searchable_advanced": true,

"searchable_batch": true,

"searchable": "Quick,Advanced,Batch",

"pii": "No",

"qtip": "",

"used_by": "Destination Vector Construction, Entry Vector",

"used_by_display": "<a href='#' onClick='remoteJS({name:\"dialogs/AddSubjectTypeDlg\", obj_id:168 });'>Destination Vector Construction</a>, <a href='#' onClick='remoteJS({name:\"dialogs/AddSubjectTypeDlg\", obj_id:193 });'>Entry Vector</a>"

},

{

"id": 1697,

"obj_id": 1697,

"display_name": "Cloning Primers",

"name": "Cloning Primers",

"type": "Text Area",

"values": "",

"created_at": "09/25/2013",

"updated_at": "09/25/2013",

"created_by": [redacted],

"searchable_quick": false,

"searchable_advanced": null,

"searchable_batch": null,

"searchable": "",

"pii": "No",

"qtip": "",

"used_by": "Destination Vector Construction",

"used_by_display": "<a href='#' onClick='remoteJS({name:\"dialogs/AddSubjectTypeDlg\", obj_id:168 });'>Destination Vector Construction</a>"

},

{

"id": 919,

"obj_id": 919,

"display_name": "Closeout Letter Date",

"name": "Closeout Letter Date",

"type": "Date",

"values": "",

"created_at": "05/10/2013",

"updated_at": "09/19/2013",

"created_by": [redacted],

"searchable_quick": false,

"searchable_advanced": null,

"searchable_batch": null,

"searchable": "",

"pii": "No",

"qtip": "",

"used_by": "Sharepoint Legacy - Pediatric Patient, Sharepoint Legacy - Adult Patient Status",

"used_by_display": "<a href='#' onClick='remoteJS({name:\"dialogs/AddSubjectTypeDlg\", obj_id:114 });'>Sharepoint Legacy - Pediatric Patient</a>, <a href='#' onClick='remoteJS({name:\"dialogs/AddSubjectTypeDlg\", obj_id:115 });'>Sharepoint Legacy - Adult Patient Status</a>"

},

{

"id": 918,

"obj_id": 918,

"display_name": "Closeout Letter Status",

"name": "Closeout Letter Status",

"type": "Text Field",

"values": "",

"created_at": "05/10/2013",

"updated_at": "11/13/2014",

"created_by": [redacted],

"searchable_quick": false,

"searchable_advanced": true,

"searchable_batch": false,

"searchable": "Advanced",

"pii": "No",

"qtip": "",

"used_by": "Sharepoint Legacy - Pediatric Patient, Sharepoint Legacy - Adult Patient Status",

"used_by_display": "<a href='#' onClick='remoteJS({name:\"dialogs/AddSubjectTypeDlg\", obj_id:114 });'>Sharepoint Legacy - Pediatric Patient</a>, <a href='#' onClick='remoteJS({name:\"dialogs/AddSubjectTypeDlg\", obj_id:115 });'>Sharepoint Legacy - Adult Patient Status</a>"

},

{

"id": 917,

"obj_id": 917,

"display_name": "Closeout Meeting",

"name": "Closeout Meeting",

"type": "Text Field",

"values": "",

"created_at": "05/10/2013",

"updated_at": "09/19/2013",

"created_by": [redacted],

"searchable_quick": false,

"searchable_advanced": null,

"searchable_batch": null,

"searchable": "",

"pii": "No",

"qtip": "",

"used_by": "Sharepoint Legacy - Pediatric Patient, Sharepoint Legacy - Adult Patient Status",

"used_by_display": "<a href='#' onClick='remoteJS({name:\"dialogs/AddSubjectTypeDlg\", obj_id:114 });'>Sharepoint Legacy - Pediatric Patient</a>, <a href='#' onClick='remoteJS({name:\"dialogs/AddSubjectTypeDlg\", obj_id:115 });'>Sharepoint Legacy - Adult Patient Status</a>"

},

{

"id": 920,

"obj_id": 920,

"display_name": "Closeout Planned",

"name": "Closeout Planned",

"type": "Date",

"values": "",

"created_at": "05/10/2013",

"updated_at": "09/19/2013",

"created_by": [redacted],

"searchable_quick": false,

"searchable_advanced": null,

"searchable_batch": null,

"searchable": "",

"pii": "No",

"qtip": "",

"used_by": "Sharepoint Legacy - Pediatric Patient, Sharepoint Legacy - Adult Patient Status",

"used_by_display": "<a href='#' onClick='remoteJS({name:\"dialogs/AddSubjectTypeDlg\", obj_id:114 });'>Sharepoint Legacy - Pediatric Patient</a>, <a href='#' onClick='remoteJS({name:\"dialogs/AddSubjectTypeDlg\", obj_id:115 });'>Sharepoint Legacy - Adult Patient Status</a>"

},

{

"id": 914,

"obj_id": 914,

"display_name": "CNS Imaging",

"name": "CNS Imaging",

"type": "Text Field",

"values": "",

"created_at": "05/10/2013",

"updated_at": "09/19/2013",

"created_by": [redacted],

"searchable_quick": false,

"searchable_advanced": null,

"searchable_batch": null,

"searchable": "",

"pii": "No",

"qtip": "",

"used_by": "Sharepoint Legacy - Pediatric Patient, Sharepoint Legacy - Adult Patient Status",

"used_by_display": "<a href='#' onClick='remoteJS({name:\"dialogs/AddSubjectTypeDlg\", obj_id:114 });'>Sharepoint Legacy - Pediatric Patient</a>, <a href='#' onClick='remoteJS({name:\"dialogs/AddSubjectTypeDlg\", obj_id:115 });'>Sharepoint Legacy - Adult Patient Status</a>"

},

{

"id": 2964,

"obj_id": 2964,

"display_name": "CNS KD climbing 20 DAE (Female)",

"name": "CNS KD climbing 20 DAE (Female)",

"type": "Text Area",

"values": "",

"created_at": "01/20/2015",

"updated_at": "01/20/2015",

"created_by": [redacted],

"searchable_quick": false,

"searchable_advanced": false,

"searchable_batch": false,

"searchable": "",

"pii": "No",

"qtip": "CNS KD with C155",

"used_by": "Drosophila Study",

"used_by_display": "<a href='#' onClick='remoteJS({name:\"dialogs/AddSubjectTypeDlg\", obj_id:236 });'>Drosophila Study</a>"

},

{

"id": 2553,

"obj_id": 2553,

"display_name": "CNS KD climbing 20 DAE (Male)",

"name": "CNS KD climbing 20 DAE (Male)",

"type": "Text Area",

"values": "",

"created_at": "04/21/2014",

"updated_at": "01/20/2015",

"created_by": [redacted],

"searchable_quick": false,

"searchable_advanced": false,

"searchable_batch": false,

"searchable": "",

"pii": "No",

"qtip": "CNS KD with C155",

"used_by": "Drosophila Study",

"used_by_display": "<a href='#' onClick='remoteJS({name:\"dialogs/AddSubjectTypeDlg\", obj_id:236 });'>Drosophila Study</a>"

},

{

"id": 2965,

"obj_id": 2965,

"display_name": "CNS KD climbing 2 DAE (Female)",

"name": "CNS KD climbing 2 DAE (Female)",

"type": "Text Area",

"values": "",

"created_at": "01/20/2015",

"updated_at": "01/20/2015",

"created_by": [redacted],

"searchable_quick": false,

"searchable_advanced": false,

"searchable_batch": false,

"searchable": "",

"pii": "No",

"qtip": "CNS KD with C155",

"used_by": "Drosophila Study",

"used_by_display": "<a href='#' onClick='remoteJS({name:\"dialogs/AddSubjectTypeDlg\", obj_id:236 });'>Drosophila Study</a>"

},

{

"id": 2552,

"obj_id": 2552,

"display_name": "CNS KD climbing 2 DAE (Male)",

"name": "CNS KD climbing 2 DAE (Male)",

"type": "Text Area",

"values": "",

"created_at": "04/21/2014",

"updated_at": "01/20/2015",

"created_by": [redacted],

"searchable_quick": false,

"searchable_advanced": false,

"searchable_batch": false,

"searchable": "",

"pii": "No",

"qtip": "CNS KD with C155",

"used_by": "Drosophila Study",

"used_by_display": "<a href='#' onClick='remoteJS({name:\"dialogs/AddSubjectTypeDlg\", obj_id:236 });'>Drosophila Study</a>"

},

{

"id": 1464,

"obj_id": 1464,

"display_name": "CNV Region",

"name": "CNV Region",

"type": "Text Field",

"values": "",

"created_at": "07/18/2013",

"updated_at": "11/13/2014",

"created_by": [redacted],

"searchable_quick": false,

"searchable_advanced": true,

"searchable_batch": false,

"searchable": "Advanced",

"pii": "No",

"qtip": "chr:position",

"used_by": "SNP Report",

"used_by_display": "<a href='#' onClick='remoteJS({name:\"dialogs/AddSubjectTypeDlg\", obj_id:155 });'>SNP Report</a>"

},

{

"id": 507,

"obj_id": 507,

"display_name": "CNV_Review",

"name": "CNV_Review",

"type": "Text Area",

"values": "",

"created_at": "05/07/2013",

"updated_at": "09/19/2013",

"created_by": [redacted],

"searchable_quick": false,

"searchable_advanced": null,

"searchable_batch": null,

"searchable": "",

"pii": "No",

"qtip": "",

"used_by": "Labmatrix Legacy - SNP Chip Non UDP, Labmatrix Legacy - SNP Chip",

"used_by_display": "<a href='#' onClick='remoteJS({name:\"dialogs/AddSubjectTypeDlg\", obj_id:99 });'>Labmatrix Legacy - SNP Chip Non UDP</a>, <a href='#' onClick='remoteJS({name:\"dialogs/AddSubjectTypeDlg\", obj_id:105 });'>Labmatrix Legacy - SNP Chip</a>"

},

{

"id": 508,

"obj_id": 508,

"display_name": "CNV_Review_Date",

"name": "CNV_Review_Date",

"type": "Date",

"values": "",

"created_at": "05/07/2013",

"updated_at": "09/19/2013",

"created_by": [redacted],

"searchable_quick": false,

"searchable_advanced": null,

"searchable_batch": null,

"searchable": "",

"pii": "No",

"qtip": "",

"used_by": "Labmatrix Legacy - SNP Chip Non UDP, Labmatrix Legacy - SNP Chip",

"used_by_display": "<a href='#' onClick='remoteJS({name:\"dialogs/AddSubjectTypeDlg\", obj_id:99 });'>Labmatrix Legacy - SNP Chip Non UDP</a>, <a href='#' onClick='remoteJS({name:\"dialogs/AddSubjectTypeDlg\", obj_id:105 });'>Labmatrix Legacy - SNP Chip</a>"

},

{

"id": 509,

"obj_id": 509,

"display_name": "CNV_Reviewer",

"name": "CNV_Reviewer",

"type": "Text Field",

"values": "",

"created_at": "05/07/2013",

"updated_at": "11/13/2014",

"created_by": [redacted],

"searchable_quick": false,

"searchable_advanced": true,

"searchable_batch": false,

"searchable": "Advanced",

"pii": "No",

"qtip": "",

"used_by": "Labmatrix Legacy - SNP Chip Non UDP, Labmatrix Legacy - SNP Chip",

"used_by_display": "<a href='#' onClick='remoteJS({name:\"dialogs/AddSubjectTypeDlg\", obj_id:99 });'>Labmatrix Legacy - SNP Chip Non UDP</a>, <a href='#' onClick='remoteJS({name:\"dialogs/AddSubjectTypeDlg\", obj_id:105 });'>Labmatrix Legacy - SNP Chip</a>"

},

{

"id": 2152,

"obj_id": 2152,

"display_name": "CNV Tolerant",

"name": "CNV Tolerant",

"type": "Text Field",

"values": "",

"created_at": "01/17/2014",

"updated_at": "01/17/2014",

"created_by": [redacted],

"searchable_quick": false,

"searchable_advanced": null,

"searchable_batch": null,

"searchable": "",

"pii": "No",

"qtip": "",

"used_by": "Exome Analysis Results",

"used_by_display": "<a href='#' onClick='remoteJS({name:\"dialogs/AddSubjectTypeDlg\", obj_id:156 });'>Exome Analysis Results</a>"

},

{

"id": 2182,

"obj_id": 2182,

"display_name": "CNV Tolerant Gene",

"name": "CNV Tolerant Gene",

"type": "Text Field",

"values": "",

"created_at": "01/17/2014",

"updated_at": "01/17/2014",

"created_by": [redacted],

"searchable_quick": false,

"searchable_advanced": null,

"searchable_batch": null,

"searchable": "",

"pii": "No",

"qtip": "",

"used_by": "Exome Analysis Results",

"used_by_display": "<a href='#' onClick='remoteJS({name:\"dialogs/AddSubjectTypeDlg\", obj_id:156 });'>Exome Analysis Results</a>"

},

{

"id": 1997,

"obj_id": 1997,

"display_name": "Cohort",

"name": "Cohort",

"type": "Text Area",

"values": "",

"created_at": "11/20/2013",

"updated_at": "11/20/2013",

"created_by": [redacted],

"searchable_quick": false,

"searchable_advanced": null,

"searchable_batch": null,

"searchable": "",

"pii": "No",

"qtip": "",

"used_by": "",

"used_by_display": ""

},

{

"id": 2049,

"obj_id": 2049,

"display_name": "Cohort Genotyped in Same Run",

"name": "Cohort Genotyped in Same Run",

"type": "Choice",

"values": "Yes, No",

"created_at": "12/04/2013",

"updated_at": "12/04/2013",

"created_by": [redacted],

"searchable_quick": false,

"searchable_advanced": null,

"searchable_batch": null,

"searchable": "",

"pii": "No",

"qtip": "",

"used_by": "Cohort Variant Call Format File",

"used_by_display": "<a href='#' onClick='remoteJS({name:\"dialogs/AddSubjectTypeDlg\", obj_id:195 });'>Cohort Variant Call Format File</a>"

},

{

"id": 2491,

"obj_id": 2491,

"display_name": "Cohort/Project Name",

"name": "Cohort/Project Name",

"type": "List",

"values": [redacted],

"created_at": "04/07/2014",

"updated_at": "01/15/2015",

"created_by": [redacted],

"searchable_quick": true,

"searchable_advanced": true,

"searchable_batch": true,

"searchable": "Quick,Advanced,Batch",

"pii": "Yes",

"qtip": "Select from drop down list. If not on list please notify UDPICS Admins",

"used_by": "Patient, Patient Follow Up Visit",

"used_by_display": "<a href='#' onClick='remoteJS({name:\"dialogs/AddSubjectTypeDlg\", obj_id:1 });'>Patient</a>, <a href='#' onClick='remoteJS({name:\"dialogs/AddSubjectTypeDlg\", obj_id:264 });'>Patient Follow Up Visit</a>"

},

{

"id": 2492,

"obj_id": 2492,

"display_name": "Cohort/Project Specimen or Tests",

"name": "Cohort/Project Specimen or Tests",

"type": "Text Area",

"values": "",

"created_at": "04/07/2014",

"updated_at": "04/15/2014",

"created_by": [redacted],

"searchable_quick": false,

"searchable_advanced": null,

"searchable_batch": null,

"searchable": "",

"pii": "No",

"qtip": "Please specify type of Specimen(s) required",

"used_by": "Patient, Patient Follow Up Visit",

"used_by_display": "<a href='#' onClick='remoteJS({name:\"dialogs/AddSubjectTypeDlg\", obj_id:1 });'>Patient</a>, <a href='#' onClick='remoteJS({name:\"dialogs/AddSubjectTypeDlg\", obj_id:264 });'>Patient Follow Up Visit</a>"

},

{

"id": 2876,

"obj_id": 2876,

"display_name": "Cohort Research Requests",

"name": "Cohort Research Requests",

"type": "Subject Back Ref",

"values": "",

"created_at": "11/10/2014",

"updated_at": "11/10/2014",

"created_by": [redacted],

"searchable_quick": false,

"searchable_advanced": null,

"searchable_batch": null,

"searchable": "",

"pii": "No",

"qtip": "",

"used_by": "Patient",

"used_by_display": "<a href='#' onClick='remoteJS({name:\"dialogs/AddSubjectTypeDlg\", obj_id:1 });'>Patient</a>"

},

{

"id": 2490,

"obj_id": 2490,

"display_name": "Cohort/Sample Management Only",

"name": "Cohort/Sample Management Only",

"type": "Choice",

"values": "Yes, No",

"created_at": "04/07/2014",

"updated_at": "06/10/2014",

"created_by": [redacted],

"searchable_quick": true,

"searchable_advanced": true,

"searchable_batch": true,

"searchable": "Quick,Advanced,Batch",

"pii": "Yes",

"qtip": "",

"used_by": "Patient, Patient Follow Up Visit",

"used_by_display": "<a href='#' onClick='remoteJS({name:\"dialogs/AddSubjectTypeDlg\", obj_id:1 });'>Patient</a>, <a href='#' onClick='remoteJS({name:\"dialogs/AddSubjectTypeDlg\", obj_id:264 });'>Patient Follow Up Visit</a>"

},

{

"id": 2051,

"obj_id": 2051,

"display_name": "Cohort VCF",

"name": "Cohort VCF",

"type": "File",

"values": "",

"created_at": "12/04/2013",

"updated_at": "12/04/2013",

"created_by": [redacted],

"searchable_quick": false,

"searchable_advanced": null,

"searchable_batch": null,

"searchable": "",

"pii": "No",

"qtip": "Unannotated",

"used_by": "Cohort Variant Call Format File",

"used_by_display": "<a href='#' onClick='remoteJS({name:\"dialogs/AddSubjectTypeDlg\", obj_id:195 });'>Cohort Variant Call Format File</a>"

},

{

"id": 2220,

"obj_id": 2220,

"display_name": "Co-injection of cas9 mRNA and sgRNA completed",

"name": "Co-injection of cas9 mRNA and sgRNA completed",

"type": "Choice",

"values": "Yes, No",

"created_at": "02/07/2014",

"updated_at": "03/14/2014",

"created_by": [redacted],

"searchable_quick": false,

"searchable_advanced": null,

"searchable_batch": null,

"searchable": "",

"pii": "No",

"qtip": "",

"used_by": "Zebrafish Mutation Project",

"used_by_display": "<a href='#' onClick='remoteJS({name:\"dialogs/AddSubjectTypeDlg\", obj_id:186 });'>Zebrafish Mutation Project</a>"

},

{

"id": 1696,

"obj_id": 1696,

"display_name": "Collaborating Center",

"name": "Collaborating Center",

"type": "Subject",

"values": "",

"created_at": "09/25/2013",

"updated_at": "12/09/2014",

"created_by": [redacted],

"searchable_quick": false,

"searchable_advanced": true,

"searchable_batch": false,

"searchable": "Advanced",

"pii": "No",

"qtip": "Collaborating Center, Outside Lab/Institution",

"used_by": "CLIA Validation, Collaborators, Requisition, Send Out Testing",

"used_by_display": "<a href='#' onClick='remoteJS({name:\"dialogs/AddSubjectTypeDlg\", obj_id:15 });'>CLIA Validation</a>, <a href='#' onClick='remoteJS({name:\"dialogs/AddSubjectTypeDlg\", obj_id:149 });'>Collaborators</a>, <a href='#' onClick='remoteJS({name:\"dialogs/AddSubjectTypeDlg\", obj_id:167 });'>Requisition</a>, <a href='#' onClick='remoteJS({name:\"dialogs/AddSubjectTypeDlg\", obj_id:278 });'>Send Out Testing</a>"

},

{

"id": 1718,

"obj_id": 1718,

"display_name": "Collaborating Department",

"name": "Collaborating Department",

"type": "Text Field",

"values": "",

"created_at": "09/26/2013",

"updated_at": "11/13/2014",

"created_by": [redacted],

"searchable_quick": false,

"searchable_advanced": true,

"searchable_batch": false,

"searchable": "Advanced",

"pii": "No",

"qtip": "",

"used_by": "Collaborating Centers",

"used_by_display": "<a href='#' onClick='remoteJS({name:\"dialogs/AddSubjectTypeDlg\", obj_id:166 });'>Collaborating Centers</a>"

},

{

"id": 1406,

"obj_id": 1406,

"display_name": "Collaborating Institution",

"name": "Collaborating Institution",

"type": "Text Field",

"values": "",

"created_at": "07/08/2013",

"updated_at": "11/13/2014",

"created_by": [redacted],

"searchable_quick": false,

"searchable_advanced": true,

"searchable_batch": false,

"searchable": "Advanced",

"pii": "No",

"qtip": "",

"used_by": "Collaborators, Collaborating Centers, Collaboration Projects",

"used_by_display": "<a href='#' onClick='remoteJS({name:\"dialogs/AddSubjectTypeDlg\", obj_id:149 });'>Collaborators</a>, <a href='#' onClick='remoteJS({name:\"dialogs/AddSubjectTypeDlg\", obj_id:166 });'>Collaborating Centers</a>, <a href='#' onClick='remoteJS({name:\"dialogs/AddSubjectTypeDlg\", obj_id:204 });'>Collaboration Projects</a>"

},

{

"id": 1779,

"obj_id": 1779,

"display_name": "Collaboration",

"name": "Collaboration",

"type": "Subject",

"values": "",

"created_at": "10/23/2013",

"updated_at": "10/23/2013",

"created_by": [redacted],

"searchable_quick": true,

"searchable_advanced": true,

"searchable_batch": true,

"searchable": "Quick,Advanced,Batch",

"pii": "No",

"qtip": "",

"used_by": "Metabolomics Results, Glycomics Results, Collaboration Reports",

"used_by_display": "<a href='#' onClick='remoteJS({name:\"dialogs/AddSubjectTypeDlg\", obj_id:174 });'>Metabolomics Results</a>, <a href='#' onClick='remoteJS({name:\"dialogs/AddSubjectTypeDlg\", obj_id:176 });'>Glycomics Results</a>, <a href='#' onClick='remoteJS({name:\"dialogs/AddSubjectTypeDlg\", obj_id:178 });'>Collaboration Reports</a>"

},

{

"id": 2141,

"obj_id": 2141,

"display_name": "Collaboration Project",

"name": "Collaboration Project",

"type": "Subject",

"values": "",

"created_at": "01/13/2014",

"updated_at": "02/21/2014",

"created_by": [redacted],

"searchable_quick": true,

"searchable_advanced": true,

"searchable_batch": true,

"searchable": "Quick,Advanced,Batch",

"pii": "No",

"qtip": "MTA/ITA associated with specific collaboration",

"used_by": "Collaborations, Information Technology Collaboration, Invoice Information",

"used_by_display": "<a href='#' onClick='remoteJS({name:\"dialogs/AddSubjectTypeDlg\", obj_id:147 });'>Collaborations</a>, <a href='#' onClick='remoteJS({name:\"dialogs/AddSubjectTypeDlg\", obj_id:183 });'>Information Technology Collaboration</a>, <a href='#' onClick='remoteJS({name:\"dialogs/AddSubjectTypeDlg\", obj_id:270 });'>Invoice Information</a>"

},

{

"id": 2384,

"obj_id": 2384,

"display_name": "Collaboration Reports",

"name": "Collaboration Reports",

"type": "Subject Back Ref",

"values": "",

"created_at": "03/11/2014",

"updated_at": "03/11/2014",

"created_by": [redacted],

"searchable_quick": false,

"searchable_advanced": null,

"searchable_batch": null,

"searchable": "",

"pii": "No",

"qtip": "",

"used_by": "Patient, Glycomics Collaborations, Patient Follow Up Visit",

"used_by_display": "<a href='#' onClick='remoteJS({name:\"dialogs/AddSubjectTypeDlg\", obj_id:1 });'>Patient</a>, <a href='#' onClick='remoteJS({name:\"dialogs/AddSubjectTypeDlg\", obj_id:244 });'>Glycomics Collaborations</a>, <a href='#' onClick='remoteJS({name:\"dialogs/AddSubjectTypeDlg\", obj_id:264 });'>Patient Follow Up Visit</a>"

},

{

"id": 1511,

"obj_id": 1511,

"display_name": "Collaborations",

"name": "Collaborations",

"type": "Subject Back Ref",

"values": "",

"created_at": "07/30/2013",

"updated_at": "03/24/2014",

"created_by": [redacted],

"searchable_quick": false,

"searchable_advanced": null,

"searchable_batch": null,

"searchable": "",

"pii": "No",

"qtip": "Material transfer collaborations",

"used_by": "Collaborators, Rescue Cell Culture, UCSC Gene List, Cell Culture, Patient Follow Up Visit, Patient",

"used_by_display": "<a href='#' onClick='remoteJS({name:\"dialogs/AddSubjectTypeDlg\", obj_id:149 });'>Collaborators</a>, <a href='#' onClick='remoteJS({name:\"dialogs/AddSubjectTypeDlg\", obj_id:247 });'>Rescue Cell Culture</a>, <a href='#' onClick='remoteJS({name:\"dialogs/AddSubjectTypeDlg\", obj_id:199 });'>UCSC Gene List</a>, <a href='#' onClick='remoteJS({name:\"dialogs/AddSubjectTypeDlg\", obj_id:40 });'>Cell Culture</a>, <a href='#' onClick='remoteJS({name:\"dialogs/AddSubjectTypeDlg\", obj_id:264 });'>Patient Follow Up Visit</a>, <a href='#' onClick='remoteJS({name:\"dialogs/AddSubjectTypeDlg\", obj_id:1 });'>Patient</a>"

},

{

"id": 1393,

"obj_id": 1393,

"display_name": "Collaboration Start Date",

"name": "Collaboration Start Date",

"type": "Date",

"values": "",

"created_at": "07/08/2013",

"updated_at": "09/19/2013",

"created_by": [redacted],

"searchable_quick": false,

"searchable_advanced": null,

"searchable_batch": null,

"searchable": "",

"pii": "No",

"qtip": "",

"used_by": "Information Technology Collaboration",

"used_by_display": "<a href='#' onClick='remoteJS({name:\"dialogs/AddSubjectTypeDlg\", obj_id:183 });'>Information Technology Collaboration</a>"

},

{

"id": 2973,

"obj_id": 2973,

"display_name": "Collaboration Status",

"name": "Collaboration Status",

"type": "Choice",

"values": "Available, Available with Restrictions, Not-Available",

"created_at": "01/22/2015",

"updated_at": "01/22/2015",

"created_by": [redacted],

"searchable_quick": false,

"searchable_advanced": true,

"searchable_batch": false,

"searchable": "Advanced",

"pii": "No",

"qtip": "Is this variant availible for collaboration?",

"used_by": "Variant Prioritization",

"used_by_display": "<a href='#' onClick='remoteJS({name:\"dialogs/AddSubjectTypeDlg\", obj_id:279 });'>Variant Prioritization</a>"

},

{

"id": 2974,

"obj_id": 2974,

"display_name": "Collaboration Status Notes",

"name": "Collaboration Status Notes",

"type": "Text Area",

"values": "",

"created_at": "01/22/2015",

"updated_at": "01/22/2015",

"created_by": [redacted],

"searchable_quick": false,

"searchable_advanced": false,

"searchable_batch": false,

"searchable": "",

"pii": "No",

"qtip": "",

"used_by": "Variant Prioritization",

"used_by_display": "<a href='#' onClick='remoteJS({name:\"dialogs/AddSubjectTypeDlg\", obj_id:279 });'>Variant Prioritization</a>"

},

{

"id": 1407,

"obj_id": 1407,

"display_name": "Collaborator Address",

"name": "Collaborator Address",

"type": "Text Area",

"values": "",

"created_at": "07/08/2013",

"updated_at": "11/13/2014",

"created_by": [redacted],

"searchable_quick": false,

"searchable_advanced": false,

"searchable_batch": false,

"searchable": "",

"pii": "Yes",

"qtip": "",

"used_by": "Collaborators, Collaborating Centers",

"used_by_display": "<a href='#' onClick='remoteJS({name:\"dialogs/AddSubjectTypeDlg\", obj_id:149 });'>Collaborators</a>, <a href='#' onClick='remoteJS({name:\"dialogs/AddSubjectTypeDlg\", obj_id:166 });'>Collaborating Centers</a>"

},

{

"id": 1410,

"obj_id": 1410,

"display_name": "Collaborator Contact Person",

"name": "Collaborator Contact Person",

"type": "Text Field",

"values": "",

"created_at": "07/08/2013",

"updated_at": "11/13/2014",

"created_by": [redacted],

"searchable_quick": false,

"searchable_advanced": true,

"searchable_batch": false,

"searchable": "Advanced",

"pii": "Yes",

"qtip": "",

"used_by": "Collaborators",

"used_by_display": "<a href='#' onClick='remoteJS({name:\"dialogs/AddSubjectTypeDlg\", obj_id:149 });'>Collaborators</a>"

},

{

"id": 1409,

"obj_id": 1409,

"display_name": "Collaborator Email",

"name": "Collaborator Email",

"type": "Text Field",

"values": "",

"created_at": "07/08/2013",

"updated_at": "06/10/2014",

"created_by": [redacted],

"searchable_quick": false,

"searchable_advanced": null,

"searchable_batch": null,

"searchable": "",

"pii": "Yes",

"qtip": "",

"used_by": "Collaborators",

"used_by_display": "<a href='#' onClick='remoteJS({name:\"dialogs/AddSubjectTypeDlg\", obj_id:149 });'>Collaborators</a>"

},

{

"id": 1691,

"obj_id": 1691,

"display_name": "Collaborator Lab",

"name": "Collaborator Lab",

"type": "List",

"values": [redacted],

"created_at": "09/11/2013",

"updated_at": "09/11/2013",

"created_by": [redacted],

"searchable_quick": false,

"searchable_advanced": null,

"searchable_batch": null,

"searchable": "",

"pii": "No",

"qtip": "",

"used_by": "",

"used_by_display": ""

},

{

"id": 1720,

"obj_id": 1720,

"display_name": "Collaborator Name",

"name": "Collaborator Name",

"type": "Subject Back Ref",

"values": "",

"created_at": "09/30/2013",

"updated_at": "09/30/2013",

"created_by": [redacted],

"searchable_quick": false,

"searchable_advanced": null,

"searchable_batch": null,

"searchable": "",

"pii": "No",

"qtip": "",

"used_by": "Collaborating Centers, Information Technology Collaboration Files",

"used_by_display": "<a href='#' onClick='remoteJS({name:\"dialogs/AddSubjectTypeDlg\", obj_id:166 });'>Collaborating Centers</a>, <a href='#' onClick='remoteJS({name:\"dialogs/AddSubjectTypeDlg\", obj_id:184 });'>Information Technology Collaboration Files</a>"

},

{

"id": 1417,

"obj_id": 1417,

"display_name": "Collaborator Names",

"name": "Collaborator Names",

"type": "Subject",

"values": "",

"created_at": "07/09/2013",

"updated_at": "11/07/2014",

"created_by": [redacted],

"searchable_quick": false,

"searchable_advanced": true,

"searchable_batch": false,

"searchable": "Advanced",

"pii": "No",

"qtip": "",

"used_by": "Collaborations, Information Technology Collaboration, Collaboration Projects, Alignment Collaboration Files, Overnight Collaboration Shipment",

"used_by_display": "<a href='#' onClick='remoteJS({name:\"dialogs/AddSubjectTypeDlg\", obj_id:147 });'>Collaborations</a>, <a href='#' onClick='remoteJS({name:\"dialogs/AddSubjectTypeDlg\", obj_id:183 });'>Information Technology Collaboration</a>, <a href='#' onClick='remoteJS({name:\"dialogs/AddSubjectTypeDlg\", obj_id:204 });'>Collaboration Projects</a>, <a href='#' onClick='remoteJS({name:\"dialogs/AddSubjectTypeDlg\", obj_id:194 });'>Alignment Collaboration Files</a>, <a href='#' onClick='remoteJS({name:\"dialogs/AddSubjectTypeDlg\", obj_id:273 });'>Overnight Collaboration Shipment</a>"

},

{

"id": 1392,

"obj_id": 1392,

"display_name": "Collaborator - Other",

"name": "Collaborator - Other",

"type": "Text Field",

"values": "",

"created_at": "07/08/2013",

"updated_at": "08/21/2013",

"created_by": [redacted],

"searchable_quick": false,

"searchable_advanced": null,

"searchable_batch": null,

"searchable": "",

"pii": "No",

"qtip": "",

"used_by": "Collaborators",

"used_by_display": "<a href='#' onClick='remoteJS({name:\"dialogs/AddSubjectTypeDlg\", obj_id:149 });'>Collaborators</a>"

},

{

"id": 1408,

"obj_id": 1408,

"display_name": "Collaborator Phone",

"name": "Collaborator Phone",

"type": "Text Field",

"values": "",

"created_at": "07/08/2013",

"updated_at": "07/19/2013",

"created_by": [redacted],

"searchable_quick": false,

"searchable_advanced": null,

"searchable_batch": null,

"searchable": "",

"pii": "Yes",

"qtip": "",

"used_by": "Collaborators",

"used_by_display": "<a href='#' onClick='remoteJS({name:\"dialogs/AddSubjectTypeDlg\", obj_id:149 });'>Collaborators</a>"

},

{

"id": 1692,

"obj_id": 1692,

"display_name": "Collaborator Role",

"name": "Collaborator Role",

"type": "Choice",

"values": "PI-Lab Chief, Post-doc, Technician",

"created_at": "09/11/2013",

"updated_at": "09/11/2013",

"created_by": [redacted],

"searchable_quick": false,

"searchable_advanced": null,

"searchable_batch": null,

"searchable": "",

"pii": "No",

"qtip": "Used to determine permissions (PI, Post-doc, tech ...)",

"used_by": "Collaborators",

"used_by_display": "<a href='#' onClick='remoteJS({name:\"dialogs/AddSubjectTypeDlg\", obj_id:149 });'>Collaborators</a>"

},

{

"id": 510,

"obj_id": 510,

"display_name": "Collected By ID",

"name": "Collected By ID",

"type": "Number",

"values": "",

"created_at": "05/07/2013",

"updated_at": "09/19/2013",

"created_by": [redacted],

"searchable_quick": false,

"searchable_advanced": null,

"searchable_batch": null,

"searchable": "",

"pii": "No",

"qtip": "",

"used_by": "Labmatrix Legacy - Example Record, Labmatrix Legacy - Send Out Tests, Labmatrix Legacy - Adult UDP Entry, Labmatrix Legacy - Test Order Records, Labmatrix Legacy - Todo Form",

"used_by_display": "<a href='#' onClick='remoteJS({name:\"dialogs/AddSubjectTypeDlg\", obj_id:102 });'>Labmatrix Legacy - Example Record</a>, <a href='#' onClick='remoteJS({name:\"dialogs/AddSubjectTypeDlg\", obj_id:104 });'>Labmatrix Legacy - Send Out Tests</a>, <a href='#' onClick='remoteJS({name:\"dialogs/AddSubjectTypeDlg\", obj_id:101 });'>Labmatrix Legacy - Adult UDP Entry</a>, <a href='#' onClick='remoteJS({name:\"dialogs/AddSubjectTypeDlg\", obj_id:106 });'>Labmatrix Legacy - Test Order Records</a>, <a href='#' onClick='remoteJS({name:\"dialogs/AddSubjectTypeDlg\", obj_id:107 });'>Labmatrix Legacy - Todo Form</a>"

},

{

"id": 418,

"obj_id": 418,

"display_name": "Collected Samples",

"name": "Collected Samples",

"type": "Subject Back Ref",

"values": "",

"created_at": "04/17/2013",

"updated_at": "08/19/2013",

"created_by": [redacted],

"searchable_quick": false,

"searchable_advanced": null,

"searchable_batch": null,

"searchable": "",

"pii": "No",

"qtip": "",

"used_by": "Blood Kit",

"used_by_display": "<a href='#' onClick='remoteJS({name:\"dialogs/AddSubjectTypeDlg\", obj_id:33 });'>Blood Kit</a>"

},

{

"id": 511,

"obj_id": 511,

"display_name": "Collection_date",

"name": "Collection_date",

"type": "Date",

"values": "",

"created_at": "05/07/2013",

"updated_at": "09/19/2013",

"created_by": [redacted],

"searchable_quick": false,

"searchable_advanced": null,

"searchable_batch": null,

"searchable": "",

"pii": "No",

"qtip": "",

"used_by": "Labmatrix Legacy - Send Out Tests",

"used_by_display": "<a href='#' onClick='remoteJS({name:\"dialogs/AddSubjectTypeDlg\", obj_id:104 });'>Labmatrix Legacy - Send Out Tests</a>"

},

{

"id": 1385,

"obj_id": 1385,

"display_name": "Collection Date",

"name": "Collection Date",

"type": "Date",

"values": "",

"created_at": "06/26/2013",

"updated_at": "10/29/2014",

"created_by": [redacted],

"searchable_quick": false,

"searchable_advanced": true,

"searchable_batch": false,

"searchable": "Advanced",

"pii": "No",

"qtip": "",

"used_by": "Research Sample, Lymphoblast Culture Request",

"used_by_display": "<a href='#' onClick='remoteJS({name:\"dialogs/AddSubjectTypeDlg\", obj_id:86 });'>Research Sample</a>, <a href='#' onClick='remoteJS({name:\"dialogs/AddSubjectTypeDlg\", obj_id:272 });'>Lymphoblast Culture Request</a>"

},

{

"id": 1297,

"obj_id": 1297,

"display_name": "Collection Date & Time",

"name": "Collection Date & Time",

"type": "Date / Time",

"values": "",

"created_at": "06/10/2013",

"updated_at": "09/19/2013",

"created_by": [redacted],

"searchable_quick": false,

"searchable_advanced": null,

"searchable_batch": null,

"searchable": "",

"pii": "No",

"qtip": "",

"used_by": "Research Sample",

"used_by_display": "<a href='#' onClick='remoteJS({name:\"dialogs/AddSubjectTypeDlg\", obj_id:86 });'>Research Sample</a>"

},

{

"id": 1010,

"obj_id": 1010,

"display_name": "Collection Time",

"name": "Collection Time",

"type": "Text Field",

"values": "",

"created_at": "05/13/2013",

"updated_at": "10/29/2014",

"created_by": [redacted],

"searchable_quick": false,

"searchable_advanced": true,

"searchable_batch": false,

"searchable": "Advanced",

"pii": "No",

"qtip": "",

"used_by": "Research Sample, Lymphoblast Culture Request",

"used_by_display": "<a href='#' onClick='remoteJS({name:\"dialogs/AddSubjectTypeDlg\", obj_id:86 });'>Research Sample</a>, <a href='#' onClick='remoteJS({name:\"dialogs/AddSubjectTypeDlg\", obj_id:272 });'>Lymphoblast Culture Request</a>"

},

{

"id": 1302,

"obj_id": 1302,

"display_name": "Column Used",

"name": "Column Used",

"type": "Choice",

"values": "PAXgene shredder column, 5Prime Preclear columns",

"created_at": "06/10/2013",

"updated_at": "09/19/2013",

"created_by": [redacted],

"searchable_quick": false,

"searchable_advanced": null,

"searchable_batch": null,

"searchable": "",

"pii": "No",

"qtip": "For RNA extraction",

"used_by": "RNA Extraction",

"used_by_display": "<a href='#' onClick='remoteJS({name:\"dialogs/AddSubjectTypeDlg\", obj_id:43 });'>RNA Extraction</a>"

},

{

"id": 5,

"obj_id": 5,

"display_name": "Comments",

"name": "Comments",

"type": "Text Area",

"values": "",

"created_at": "01/28/2013",

"updated_at": "08/08/2013",

"created_by": [redacted],

"searchable_quick": false,

"searchable_advanced": null,

"searchable_batch": null,

"searchable": "",

"pii": "No",

"qtip": "",

"used_by": "Family, Patient Medical Material, Sequencing Centers, Labmatrix Legacy - FEDEX Specimen Kit Tracking, Labmatrix Legacy - Send Out Tests, Drosophila Reports, PhenoTips Review, Rescue Cell Culture, Patient Research Meeting, Phenotype, iPS Cells, Blood Kit, Varsifter Files, Phased and Imputed VCF, Send Out Testing, GenomeStudio Files, Drosophila Phenotypes, Independent SNP Analysis, Collaboration Reports, Alignment Information, SNP Report, Vial, Requisition, Neurotransmitter, Plasma Extraction, Serum Extraction, Bedfiles, Urine Pellet, SNP Analysis, dbGaP Submission, Cell Culture, Zebrafish Phenotype, Entry Vector, MiniPrep DNA, Antibody, Genome Amplification, Glycerol Stock, Mutagenized Vectors, Exome Analysis, Cohort Research Request, Lymphoblast Culture Request",

"used_by_display": "<a href='#' onClick='remoteJS({name:\"dialogs/AddSubjectTypeDlg\", obj_id:2 });'>Family</a>, <a href='#' onClick='remoteJS({name:\"dialogs/AddSubjectTypeDlg\", obj_id:32 });'>Patient Medical Material</a>, <a href='#' onClick='remoteJS({name:\"dialogs/AddSubjectTypeDlg\", obj_id:196 });'>Sequencing Centers</a>, <a href='#' onClick='remoteJS({name:\"dialogs/AddSubjectTypeDlg\", obj_id:103 });'>Labmatrix Legacy - FEDEX Specimen Kit Tracking</a>, <a href='#' onClick='remoteJS({name:\"dialogs/AddSubjectTypeDlg\", obj_id:104 });'>Labmatrix Legacy - Send Out Tests</a>, <a href='#' onClick='remoteJS({name:\"dialogs/AddSubjectTypeDlg\", obj_id:237 });'>Drosophila Reports</a>, <a href='#' onClick='remoteJS({name:\"dialogs/AddSubjectTypeDlg\", obj_id:205 });'>PhenoTips Review</a>, <a href='#' onClick='remoteJS({name:\"dialogs/AddSubjectTypeDlg\", obj_id:247 });'>Rescue Cell Culture</a>, <a href='#' onClick='remoteJS({name:\"dialogs/AddSubjectTypeDlg\", obj_id:127 });'>Patient Research Meeting</a>, <a href='#' onClick='remoteJS({name:\"dialogs/AddSubjectTypeDlg\", obj_id:160 });'>Phenotype</a>, <a href='#' onClick='remoteJS({name:\"dialogs/AddSubjectTypeDlg\", obj_id:41 });'>iPS Cells</a>, <a href='#' onClick='remoteJS({name:\"dialogs/AddSubjectTypeDlg\", obj_id:33 });'>Blood Kit</a>, <a href='#' onClick='remoteJS({name:\"dialogs/AddSubjectTypeDlg\", obj_id:198 });'>Varsifter Files</a>, <a href='#' onClick='remoteJS({name:\"dialogs/AddSubjectTypeDlg\", obj_id:188 });'>Phased and Imputed VCF</a>, <a href='#' onClick='remoteJS({name:\"dialogs/AddSubjectTypeDlg\", obj_id:278 });'>Send Out Testing</a>, <a href='#' onClick='remoteJS({name:\"dialogs/AddSubjectTypeDlg\", obj_id:202 });'>GenomeStudio Files</a>, <a href='#' onClick='remoteJS({name:\"dialogs/AddSubjectTypeDlg\", obj_id:265 });'>Drosophila Phenotypes</a>, <a href='#' onClick='remoteJS({name:\"dialogs/AddSubjectTypeDlg\", obj_id:215 });'>Independent SNP Analysis</a>, <a href='#' onClick='remoteJS({name:\"dialogs/AddSubjectTypeDlg\", obj_id:178 });'>Collaboration Reports</a>, <a href='#' onClick='remoteJS({name:\"dialogs/AddSubjectTypeDlg\", obj_id:208 });'>Alignment Information</a>, <a href='#' onClick='remoteJS({name:\"dialogs/AddSubjectTypeDlg\", obj_id:155 });'>SNP Report</a>, <a href='#' onClick='remoteJS({name:\"dialogs/AddSubjectTypeDlg\", obj_id:152 });'>Vial</a>, <a href='#' onClick='remoteJS({name:\"dialogs/AddSubjectTypeDlg\", obj_id:167 });'>Requisition</a>, <a href='#' onClick='remoteJS({name:\"dialogs/AddSubjectTypeDlg\", obj_id:169 });'>Neurotransmitter</a>, <a href='#' onClick='remoteJS({name:\"dialogs/AddSubjectTypeDlg\", obj_id:47 });'>Plasma Extraction</a>, <a href='#' onClick='remoteJS({name:\"dialogs/AddSubjectTypeDlg\", obj_id:119 });'>Serum Extraction</a>, <a href='#' onClick='remoteJS({name:\"dialogs/AddSubjectTypeDlg\", obj_id:238 });'>Bedfiles</a>, <a href='#' onClick='remoteJS({name:\"dialogs/AddSubjectTypeDlg\", obj_id:170 });'>Urine Pellet</a>, <a href='#' onClick='remoteJS({name:\"dialogs/AddSubjectTypeDlg\", obj_id:59 });'>SNP Analysis</a>, <a href='#' onClick='remoteJS({name:\"dialogs/AddSubjectTypeDlg\", obj_id:213 });'>dbGaP Submission</a>, <a href='#' onClick='remoteJS({name:\"dialogs/AddSubjectTypeDlg\", obj_id:40 });'>Cell Culture</a>, <a href='#' onClick='remoteJS({name:\"dialogs/AddSubjectTypeDlg\", obj_id:173 });'>Zebrafish Phenotype</a>, <a href='#' onClick='remoteJS({name:\"dialogs/AddSubjectTypeDlg\", obj_id:193 });'>Entry Vector</a>, <a href='#' onClick='remoteJS({name:\"dialogs/AddSubjectTypeDlg\", obj_id:249 });'>MiniPrep DNA</a>, <a href='#' onClick='remoteJS({name:\"dialogs/AddSubjectTypeDlg\", obj_id:189 });'>Antibody</a>, <a href='#' onClick='remoteJS({name:\"dialogs/AddSubjectTypeDlg\", obj_id:258 });'>Genome Amplification</a>, <a href='#' onClick='remoteJS({name:\"dialogs/AddSubjectTypeDlg\", obj_id:171 });'>Glycerol Stock</a>, <a href='#' onClick='remoteJS({name:\"dialogs/AddSubjectTypeDlg\", obj_id:228 });'>Mutagenized Vectors</a>, <a href='#' onClick='remoteJS({name:\"dialogs/AddSubjectTypeDlg\", obj_id:60 });'>Exome Analysis</a>, <a href='#' onClick='remoteJS({name:\"dialogs/AddSubjectTypeDlg\", obj_id:271 });'>Cohort Research Request</a>, <a href='#' onClick='remoteJS({name:\"dialogs/AddSubjectTypeDlg\", obj_id:272 });'>Lymphoblast Culture Request</a>"

},

{

"id": 1344,

"obj_id": 1344,

"display_name": "Comments/Special Instructions",

"name": "Comments/Special Instructions",

"type": "Text Field",

"values": "",

"created_at": "06/21/2013",

"updated_at": "09/19/2013",

"created_by": [redacted],

"searchable_quick": false,

"searchable_advanced": null,

"searchable_batch": null,

"searchable": "",

"pii": "No",

"qtip": "",

"used_by": "Genome Sequencing, Exome Sequencing, Primer Order, SNP Chip Sequencing, Glycomics Collaborations, Cohort Research Request",

"used_by_display": "<a href='#' onClick='remoteJS({name:\"dialogs/AddSubjectTypeDlg\", obj_id:212 });'>Genome Sequencing</a>, <a href='#' onClick='remoteJS({name:\"dialogs/AddSubjectTypeDlg\", obj_id:161 });'>Exome Sequencing</a>, <a href='#' onClick='remoteJS({name:\"dialogs/AddSubjectTypeDlg\", obj_id:217 });'>Primer Order</a>, <a href='#' onClick='remoteJS({name:\"dialogs/AddSubjectTypeDlg\", obj_id:203 });'>SNP Chip Sequencing</a>, <a href='#' onClick='remoteJS({name:\"dialogs/AddSubjectTypeDlg\", obj_id:244 });'>Glycomics Collaborations</a>, <a href='#' onClick='remoteJS({name:\"dialogs/AddSubjectTypeDlg\", obj_id:271 });'>Cohort Research Request</a>"

},

{

"id": 512,

"obj_id": 512,

"display_name": "Communication Id",

"name": "Communication Id",

"type": "Number",

"values": "",

"created_at": "05/07/2013",

"updated_at": "09/19/2013",

"created_by": [redacted],

"searchable_quick": false,

"searchable_advanced": null,

"searchable_batch": null,

"searchable": "",

"pii": "No",

"qtip": "",

"used_by": "Labmatrix Legacy - Communications",

"used_by_display": "<a href='#' onClick='remoteJS({name:\"dialogs/AddSubjectTypeDlg\", obj_id:92 });'>Labmatrix Legacy - Communications</a>"

},

{

"id": 2707,

"obj_id": 2707,

"display_name": "Communication Notes",

"name": "Communication Notes",

"type": "Text Area",

"values": "",

"created_at": "07/17/2014",

"updated_at": "07/17/2014",

"created_by": [redacted],

"searchable_quick": false,

"searchable_advanced": false,

"searchable_batch": false,

"searchable": "",

"pii": "Yes",

"qtip": "",

"used_by": "Letters",

"used_by_display": "<a href='#' onClick='remoteJS({name:\"dialogs/AddSubjectTypeDlg\", obj_id:253 });'>Letters</a>"

},

{

"id": 1811,

"obj_id": 1811,

"display_name": "Communications - Labmatrix Legacy",

"name": "Communications - Labmatrix Legacy",

"type": "Subject Back Ref",

"values": "",

"created_at": "10/29/2013",

"updated_at": "10/29/2013",

"created_by": [redacted],

"searchable_quick": false,

"searchable_advanced": null,

"searchable_batch": null,

"searchable": "",

"pii": "No",

"qtip": "",

"used_by": "Patient, Patient Follow Up Visit",

"used_by_display": "<a href='#' onClick='remoteJS({name:\"dialogs/AddSubjectTypeDlg\", obj_id:1 });'>Patient</a>, <a href='#' onClick='remoteJS({name:\"dialogs/AddSubjectTypeDlg\", obj_id:264 });'>Patient Follow Up Visit</a>"

},

{

"id": 513,

"obj_id": 513,

"display_name": "completed_date",

"name": "completed_date",

"type": "Date",

"values": "",

"created_at": "05/07/2013",

"updated_at": "09/19/2013",

"created_by": [redacted],

"searchable_quick": false,

"searchable_advanced": null,

"searchable_batch": null,

"searchable": "",

"pii": "No",

"qtip": "",

"used_by": "Labmatrix Legacy - Todo Form",

"used_by_display": "<a href='#' onClick='remoteJS({name:\"dialogs/AddSubjectTypeDlg\", obj_id:107 });'>Labmatrix Legacy - Todo Form</a>"

},

{

"id": 1249,

"obj_id": 1249,

"display_name": "Complete Family History",

"name": "Complete Family History",

"type": "Radio Buttons",

"values": "Yes, No",

"created_at": "06/03/2013",

"updated_at": "09/19/2013",

"created_by": [redacted],

"searchable_quick": false,

"searchable_advanced": null,

"searchable_batch": null,

"searchable": "",

"pii": "No",

"qtip": "",

"used_by": "NIH Visit",

"used_by_display": "<a href='#' onClick='remoteJS({name:\"dialogs/AddSubjectTypeDlg\", obj_id:140 });'>NIH Visit</a>"

},

{

"id": 1570,

"obj_id": 1570,

"display_name": "Complete History Obtained",

"name": "Complete History Obtained",

"type": "Radio Buttons",

"values": "Yes, No",

"created_at": "08/12/2013",

"updated_at": "08/12/2013",

"created_by": [redacted],

"searchable_quick": false,

"searchable_advanced": null,

"searchable_batch": null,

"searchable": "",

"pii": "No",

"qtip": "",

"used_by": "Patient Visit Tools, Patient Visit Planning for Version C",

"used_by_display": "<a href='#' onClick='remoteJS({name:\"dialogs/AddSubjectTypeDlg\", obj_id:163 });'>Patient Visit Tools</a>, <a href='#' onClick='remoteJS({name:\"dialogs/AddSubjectTypeDlg\", obj_id:192 });'>Patient Visit Planning for Version C</a>"

},

{

"id": 382,

"obj_id": 382,

"display_name": "Compound Heterozygous",

"name": "Compound Heterozygous",

"type": "CheckBox",

"values": "",

"created_at": "04/15/2013",

"updated_at": "09/19/2013",

"created_by": [redacted],

"searchable_quick": false,

"searchable_advanced": null,

"searchable_batch": null,

"searchable": "",

"pii": "No",

"qtip": "Boolean filter",

"used_by": "Exome Analysis",

"used_by_display": "<a href='#' onClick='remoteJS({name:\"dialogs/AddSubjectTypeDlg\", obj_id:60 });'>Exome Analysis</a>"

},

{

"id": 2887,

"obj_id": 2887,

"display_name": "Compound Heterozygous Exomiser Output file",

"name": "Compound Heterozygous Exomiser Output file",

"type": "File",

"values": "",

"created_at": "11/12/2014",

"updated_at": "11/12/2014",

"created_by": [redacted],

"searchable_quick": false,

"searchable_advanced": true,

"searchable_batch": false,

"searchable": "Advanced",

"pii": "No",

"qtip": "",

"used_by": "Exome Analysis",

"used_by_display": "<a href='#' onClick='remoteJS({name:\"dialogs/AddSubjectTypeDlg\", obj_id:60 });'>Exome Analysis</a>"

},

{

"id": 2879,

"obj_id": 2879,

"display_name": "Compound Heterozygous Exomiser Output File",

"name": "Compound Heterozygous Exomiser Output File",

"type": "Text Field",

"values": "",

"created_at": "11/11/2014",

"updated_at": "11/11/2014",

"created_by": [redacted],

"searchable_quick": false,

"searchable_advanced": true,

"searchable_batch": false,

"searchable": "Advanced",

"pii": "No",

"qtip": "",

"used_by": "",

"used_by_display": ""

},

{

"id": 1784,

"obj_id": 1784,

"display_name": "CompoundName",

"name": "CompoundName",

"type": "Text Field",

"values": "",

"created_at": "10/23/2013",

"updated_at": "10/28/2014",

"created_by": [redacted],

"searchable_quick": false,

"searchable_advanced": true,

"searchable_batch": false,

"searchable": "Advanced",

"pii": "No",

"qtip": "",

"used_by": "Metabolomics Results, Glycomics Results",

"used_by_display": "<a href='#' onClick='remoteJS({name:\"dialogs/AddSubjectTypeDlg\", obj_id:174 });'>Metabolomics Results</a>, <a href='#' onClick='remoteJS({name:\"dialogs/AddSubjectTypeDlg\", obj_id:176 });'>Glycomics Results</a>"

},

{

"id": 1782,

"obj_id": 1782,

"display_name": "CompoundNameID",

"name": "CompoundNameID",

"type": "Text Field",

"values": "",

"created_at": "10/23/2013",

"updated_at": "10/28/2014",

"created_by": [redacted],

"searchable_quick": false,

"searchable_advanced": true,

"searchable_batch": false,

"searchable": "Advanced",

"pii": "No",

"qtip": "",

"used_by": "Metabolomics Results, Glycomics Results",

"used_by_display": "<a href='#' onClick='remoteJS({name:\"dialogs/AddSubjectTypeDlg\", obj_id:174 });'>Metabolomics Results</a>, <a href='#' onClick='remoteJS({name:\"dialogs/AddSubjectTypeDlg\", obj_id:176 });'>Glycomics Results</a>"

},

{

"id": 2517,

"obj_id": 2517,

"display_name": "CompoundNameID, m/z",

"name": "CompoundNameID, m/z",

"type": "Text Field",

"values": "",

"created_at": "04/15/2014",

"updated_at": "10/28/2014",

"created_by": [redacted],

"searchable_quick": false,

"searchable_advanced": true,

"searchable_batch": false,

"searchable": "Advanced",

"pii": "No",

"qtip": "",

"used_by": "Glycomics Results",

"used_by_display": "<a href='#' onClick='remoteJS({name:\"dialogs/AddSubjectTypeDlg\", obj_id:176 });'>Glycomics Results</a>"

},

{

"id": 514,

"obj_id": 514,

"display_name": "Concentration",

"name": "Concentration",

"type": "Text Area",

"values": "",

"created_at": "05/07/2013",

"updated_at": "09/19/2013",

"created_by": [redacted],

"searchable_quick": false,

"searchable_advanced": null,

"searchable_batch": null,

"searchable": "",

"pii": "No",

"qtip": "",

"used_by": "Labmatrix Legacy - Biomaterials",

"used_by_display": "<a href='#' onClick='remoteJS({name:\"dialogs/AddSubjectTypeDlg\", obj_id:91 });'>Labmatrix Legacy - Biomaterials</a>"

},

{

"id": 1977,

"obj_id": 1977,

"display_name": "Concentration (ng/ul)",

"name": "Concentration (ng/ul)",

"type": "Number",

"values": "",

"created_at": "11/19/2013",

"updated_at": "10/23/2014",

"created_by": [redacted],

"searchable_quick": false,

"searchable_advanced": true,

"searchable_batch": false,

"searchable": "Advanced",

"pii": "No",

"qtip": "",

"used_by": "DNA Extraction, MiniPrep DNA, Entry Vector, Destination Vector Construction, Clone, MaxiPrep Storage, Genome Amplification, Transcriptome, Mutagenized Vectors, Zebrafish Mutation Project",

"used_by_display": "<a href='#' onClick='remoteJS({name:\"dialogs/AddSubjectTypeDlg\", obj_id:122 });'>DNA Extraction</a>, <a href='#' onClick='remoteJS({name:\"dialogs/AddSubjectTypeDlg\", obj_id:249 });'>MiniPrep DNA</a>, <a href='#' onClick='remoteJS({name:\"dialogs/AddSubjectTypeDlg\", obj_id:193 });'>Entry Vector</a>, <a href='#' onClick='remoteJS({name:\"dialogs/AddSubjectTypeDlg\", obj_id:168 });'>Destination Vector Construction</a>, <a href='#' onClick='remoteJS({name:\"dialogs/AddSubjectTypeDlg\", obj_id:266 });'>Clone</a>, <a href='#' onClick='remoteJS({name:\"dialogs/AddSubjectTypeDlg\", obj_id:269 });'>MaxiPrep Storage</a>, <a href='#' onClick='remoteJS({name:\"dialogs/AddSubjectTypeDlg\", obj_id:258 });'>Genome Amplification</a>, <a href='#' onClick='remoteJS({name:\"dialogs/AddSubjectTypeDlg\", obj_id:255 });'>Transcriptome</a>, <a href='#' onClick='remoteJS({name:\"dialogs/AddSubjectTypeDlg\", obj_id:228 });'>Mutagenized Vectors</a>, <a href='#' onClick='remoteJS({name:\"dialogs/AddSubjectTypeDlg\", obj_id:186 });'>Zebrafish Mutation Project</a>"

},

{

"id": 361,

"obj_id": 361,

"display_name": "Concentration (ng/uL)",

"name": "Concentration (ng/uL)",

"type": "Text Field",

"values": "",

"created_at": "04/15/2013",

"updated_at": "10/23/2014",

"created_by": [redacted],

"searchable_quick": false,

"searchable_advanced": true,

"searchable_batch": false,

"searchable": "Advanced",

"pii": "No",

"qtip": "DNA",

"used_by": "Destination Vector Construction, Vial, Entry Vector, In situ Hybridization",

"used_by_display": "<a href='#' onClick='remoteJS({name:\"dialogs/AddSubjectTypeDlg\", obj_id:168 });'>Destination Vector Construction</a>, <a href='#' onClick='remoteJS({name:\"dialogs/AddSubjectTypeDlg\", obj_id:152 });'>Vial</a>, <a href='#' onClick='remoteJS({name:\"dialogs/AddSubjectTypeDlg\", obj_id:193 });'>Entry Vector</a>, <a href='#' onClick='remoteJS({name:\"dialogs/AddSubjectTypeDlg\", obj_id:252 });'>In situ Hybridization</a>"

},

{

"id": 2402,

"obj_id": 2402,

"display_name": "Concentration of cas9 mRNA Injected",

"name": "Concentration of cas9 mRNA Injected",

"type": "Text Field",

"values": "",

"created_at": "03/14/2014",

"updated_at": "03/14/2014",

"created_by": [redacted],

"searchable_quick": false,

"searchable_advanced": null,

"searchable_batch": null,

"searchable": "",

"pii": "No",

"qtip": "",

"used_by": "Zebrafish Mutation Project, Zebrafish Injections",

"used_by_display": "<a href='#' onClick='remoteJS({name:\"dialogs/AddSubjectTypeDlg\", obj_id:186 });'>Zebrafish Mutation Project</a>, <a href='#' onClick='remoteJS({name:\"dialogs/AddSubjectTypeDlg\", obj_id:232 });'>Zebrafish Injections</a>"

},

{

"id": 2401,

"obj_id": 2401,

"display_name": "Concentration of sgRNA Injected",

"name": "Concentration of sgRNA Injected",

"type": "Text Field",

"values": "",

"created_at": "03/14/2014",

"updated_at": "03/14/2014",

"created_by": [redacted],

"searchable_quick": false,

"searchable_advanced": null,

"searchable_batch": null,

"searchable": "",

"pii": "No",

"qtip": "",

"used_by": "Zebrafish Mutation Project, Zebrafish Injections",

"used_by_display": "<a href='#' onClick='remoteJS({name:\"dialogs/AddSubjectTypeDlg\", obj_id:186 });'>Zebrafish Mutation Project</a>, <a href='#' onClick='remoteJS({name:\"dialogs/AddSubjectTypeDlg\", obj_id:232 });'>Zebrafish Injections</a>"

},

{

"id": 1331,

"obj_id": 1331,

"display_name": "Concentration (ug/ml)",

"name": "Concentration (ug/ml)",

"type": "Number",

"values": "",

"created_at": "06/19/2013",

"updated_at": "10/23/2014",

"created_by": [redacted],

"searchable_quick": false,

"searchable_advanced": true,

"searchable_batch": false,

"searchable": "Advanced",

"pii": "No",

"qtip": "RNA",

"used_by": "RNA Extraction, Vial",

"used_by_display": "<a href='#' onClick='remoteJS({name:\"dialogs/AddSubjectTypeDlg\", obj_id:43 });'>RNA Extraction</a>, <a href='#' onClick='remoteJS({name:\"dialogs/AddSubjectTypeDlg\", obj_id:152 });'>Vial</a>"

},

{

"id": 515,

"obj_id": 515,

"display_name": "Concentration Units",

"name": "Concentration Units",

"type": "Text Field",

"values": "",

"created_at": "05/07/2013",

"updated_at": "09/19/2013",

"created_by": [redacted],

"searchable_quick": false,

"searchable_advanced": null,

"searchable_batch": null,

"searchable": "",

"pii": "No",

"qtip": "",

"used_by": "Labmatrix Legacy - Biomaterials",

"used_by_display": "<a href='#' onClick='remoteJS({name:\"dialogs/AddSubjectTypeDlg\", obj_id:91 });'>Labmatrix Legacy - Biomaterials</a>"

},

{

"id": 1872,

"obj_id": 1872,

"display_name": "Conclusion",

"name": "Conclusion",

"type": "Text Area",

"values": "",

"created_at": "11/07/2013",

"updated_at": "11/07/2013",

"created_by": [redacted],

"searchable_quick": false,

"searchable_advanced": null,

"searchable_batch": null,

"searchable": "",

"pii": "No",

"qtip": "",

"used_by": "Sanger Interpretation",

"used_by_display": "<a href='#' onClick='remoteJS({name:\"dialogs/AddSubjectTypeDlg\", obj_id:22 });'>Sanger Interpretation</a>"

},

{

"id": 516,

"obj_id": 516,

"display_name": "Confirmed",

"name": "Confirmed",

"type": "Text Field",

"values": "",

"created_at": "05/07/2013",

"updated_at": "11/13/2014",

"created_by": [redacted],

"searchable_quick": false,

"searchable_advanced": true,

"searchable_batch": false,

"searchable": "Advanced",

"pii": "No",

"qtip": "",

"used_by": "Labmatrix Legacy - Phenotype Legacy Files",

"used_by_display": "<a href='#' onClick='remoteJS({name:\"dialogs/AddSubjectTypeDlg\", obj_id:96 });'>Labmatrix Legacy - Phenotype Legacy Files</a>"

},

{

"id": 1174,

"obj_id": 1174,

"display_name": "Confirmed No Mutation in cDNA",

"name": "Confirmed No Mutation in cDNA",

"type": "Choice",

"values": "Yes, No",

"created_at": "05/29/2013",

"updated_at": "11/13/2014",

"created_by": [redacted],

"searchable_quick": false,

"searchable_advanced": true,

"searchable_batch": false,

"searchable": "Advanced",

"pii": "No",

"qtip": "",

"used_by": "Entry Vector, Clone",

"used_by_display": "<a href='#' onClick='remoteJS({name:\"dialogs/AddSubjectTypeDlg\", obj_id:193 });'>Entry Vector</a>, <a href='#' onClick='remoteJS({name:\"dialogs/AddSubjectTypeDlg\", obj_id:266 });'>Clone</a>"

},

{

"id": 2045,

"obj_id": 2045,

"display_name": "Confirmed Offspring Relationships",

"name": "Confirmed Offspring Relationships",

"type": "Choice",

"values": "Yes, No, Not Checked",

"created_at": "12/02/2013",

"updated_at": "11/13/2014",

"created_by": [redacted],

"searchable_quick": false,

"searchable_advanced": true,

"searchable_batch": false,

"searchable": "Advanced",

"pii": "No",

"qtip": "",

"used_by": "SNP Analysis",

"used_by_display": "<a href='#' onClick='remoteJS({name:\"dialogs/AddSubjectTypeDlg\", obj_id:59 });'>SNP Analysis</a>"

},

{

"id": 1320,

"obj_id": 1320,

"display_name": "Confirmed Parental Relationships",

"name": "Confirmed Parental Relationships",

"type": "Choice",

"values": "Yes, No, Not Checked",

"created_at": "06/14/2013",

"updated_at": "11/13/2014",

"created_by": [redacted],

"searchable_quick": false,

"searchable_advanced": true,

"searchable_batch": false,

"searchable": "Advanced",

"pii": "No",

"qtip": "",

"used_by": "SNP Analysis",

"used_by_display": "<a href='#' onClick='remoteJS({name:\"dialogs/AddSubjectTypeDlg\", obj_id:59 });'>SNP Analysis</a>"

},

{

"id": 517,

"obj_id": 517,

"display_name": "Consanguinity",

"name": "Consanguinity",

"type": "Text Area",

"values": "",

"created_at": "05/07/2013",

"updated_at": "09/19/2013",

"created_by": [redacted],

"searchable_quick": false,

"searchable_advanced": null,

"searchable_batch": null,

"searchable": "",

"pii": "No",

"qtip": "",

"used_by": "Labmatrix Legacy - Subject Familial Association",

"used_by_display": "<a href='#' onClick='remoteJS({name:\"dialogs/AddSubjectTypeDlg\", obj_id:100 });'>Labmatrix Legacy - Subject Familial Association</a>"

},

{

"id": 1491,

"obj_id": 1491,

"display_name": "Consanguinity Suspected",

"name": "Consanguinity Suspected",

"type": "Choice",

"values": "Yes, No, Unknown",

"created_at": "07/24/2013",

"updated_at": "07/24/2013",

"created_by": [redacted],

"searchable_quick": false,

"searchable_advanced": null,

"searchable_batch": null,

"searchable": "",

"pii": "No",

"qtip": "",

"used_by": "Friday Meeting Notes",

"used_by_display": "<a href='#' onClick='remoteJS({name:\"dialogs/AddSubjectTypeDlg\", obj_id:82 });'>Friday Meeting Notes</a>"

},

{

"id": 518,

"obj_id": 518,

"display_name": "Consent Form Name",

"name": "Consent Form Name",

"type": "Text Area",

"values": "",

"created_at": "05/07/2013",

"updated_at": "10/29/2014",

"created_by": [redacted],

"searchable_quick": false,

"searchable_advanced": false,

"searchable_batch": false,

"searchable": "",

"pii": "Yes",

"qtip": "",

"used_by": "Labmatrix Legacy - IRB Protocols",

"used_by_display": "<a href='#' onClick='remoteJS({name:\"dialogs/AddSubjectTypeDlg\", obj_id:93 });'>Labmatrix Legacy - IRB Protocols</a>"

},

{

"id": 423,

"obj_id": 423,

"display_name": "Consent Form Present",

"name": "Consent Form Present",

"type": "Choice",

"values": "Yes, No",

"created_at": "04/18/2013",

"updated_at": "10/29/2014",

"created_by": [redacted],

"searchable_quick": false,

"searchable_advanced": true,

"searchable_batch": false,

"searchable": "Advanced",

"pii": "No",

"qtip": "",

"used_by": "Research Sample",

"used_by_display": "<a href='#' onClick='remoteJS({name:\"dialogs/AddSubjectTypeDlg\", obj_id:86 });'>Research Sample</a>"

},

{

"id": 128,

"obj_id": 128,

"display_name": "Consent Forwarded for Scanning",

"name": "Consent Forwarded for Scanning",

"type": "CheckBox",

"values": "",

"created_at": "02/12/2013",

"updated_at": "09/19/2013",

"created_by": [redacted],

"searchable_quick": false,

"searchable_advanced": null,

"searchable_batch": null,

"searchable": "",

"pii": "No",

"qtip": "",

"used_by": "Blood Kit",

"used_by_display": "<a href='#' onClick='remoteJS({name:\"dialogs/AddSubjectTypeDlg\", obj_id:33 });'>Blood Kit</a>"

},

{

"id": 519,

"obj_id": 519,

"display_name": "Consent Id",

"name": "Consent Id",

"type": "Number",

"values": "",

"created_at": "05/07/2013",

"updated_at": "09/19/2013",

"created_by": [redacted],

"searchable_quick": false,

"searchable_advanced": null,

"searchable_batch": null,

"searchable": "",

"pii": "No",

"qtip": "",

"used_by": "Labmatrix Legacy - IRB Protocols",

"used_by_display": "<a href='#' onClick='remoteJS({name:\"dialogs/AddSubjectTypeDlg\", obj_id:93 });'>Labmatrix Legacy - IRB Protocols</a>"

},

{

"id": 1401,

"obj_id": 1401,

"display_name": "Consent Obtain and Scanned Into Lims",

"name": "Consent Obtain and Scanned Into Lims",

"type": "Radio Buttons",

"values": "Yes, No",

"created_at": "07/08/2013",

"updated_at": "09/19/2013",

"created_by": [redacted],

"searchable_quick": false,

"searchable_advanced": null,

"searchable_batch": null,

"searchable": "",

"pii": "No",

"qtip": "",

"used_by": "",

"used_by_display": ""

},

{

"id": 1390,

"obj_id": 1390,

"display_name": "Consents",

"name": "Consents",

"type": "Subject Back Ref",

"values": "",

"created_at": "07/02/2013",

"updated_at": "06/10/2014",

"created_by": [redacted],

"searchable_quick": true,

"searchable_advanced": true,

"searchable_batch": true,

"searchable": "Quick,Advanced,Batch",

"pii": "Yes",

"qtip": "",

"used_by": "Patient, Family, Patient Follow Up Visit",

"used_by_display": "<a href='#' onClick='remoteJS({name:\"dialogs/AddSubjectTypeDlg\", obj_id:1 });'>Patient</a>, <a href='#' onClick='remoteJS({name:\"dialogs/AddSubjectTypeDlg\", obj_id:2 });'>Family</a>, <a href='#' onClick='remoteJS({name:\"dialogs/AddSubjectTypeDlg\", obj_id:264 });'>Patient Follow Up Visit</a>"

},

{

"id": 520,

"obj_id": 520,

"display_name": "Consent Status",

"name": "Consent Status",

"type": "Text Field",

"values": "",

"created_at": "05/07/2013",

"updated_at": "09/19/2013",

"created_by": [redacted],

"searchable_quick": false,

"searchable_advanced": null,

"searchable_batch": null,

"searchable": "",

"pii": "No",

"qtip": "",

"used_by": "Labmatrix Legacy - IRB Protocols",

"used_by_display": "<a href='#' onClick='remoteJS({name:\"dialogs/AddSubjectTypeDlg\", obj_id:93 });'>Labmatrix Legacy - IRB Protocols</a>"

},

{

"id": 521,

"obj_id": 521,

"display_name": "Consent Status Date",

"name": "Consent Status Date",

"type": "Date",

"values": "",

"created_at": "05/07/2013",

"updated_at": "06/10/2014",

"created_by": [redacted],

"searchable_quick": false,

"searchable_advanced": null,

"searchable_batch": null,

"searchable": "",

"pii": "Yes",

"qtip": "",

"used_by": "Labmatrix Legacy - IRB Protocols",

"used_by_display": "<a href='#' onClick='remoteJS({name:\"dialogs/AddSubjectTypeDlg\", obj_id:93 });'>Labmatrix Legacy - IRB Protocols</a>"

},

{

"id": 2814,

"obj_id": 2814,

"display_name": "Consent Text for Data Sharing",

"name": "Consent Text for Data Sharing",

"type": "File",

"values": "",

"created_at": "09/02/2014",

"updated_at": "09/02/2014",

"created_by": [redacted],

"searchable_quick": false,

"searchable_advanced": false,

"searchable_batch": false,

"searchable": "",

"pii": "No",

"qtip": "File with the wording from the consents about data sharing",

"used_by": "Consent Versions",

"used_by_display": "<a href='#' onClick='remoteJS({name:\"dialogs/AddSubjectTypeDlg\", obj_id:239 });'>Consent Versions</a>"

},

{

"id": 2582,

"obj_id": 2582,

"display_name": "Consent type",

"name": "Consent type",

"type": "Choice",

"values": "Standard, Family, Minor, Normal",

"created_at": "05/01/2014",

"updated_at": "05/02/2014",

"created_by": [redacted],

"searchable_quick": false,

"searchable_advanced": null,

"searchable_batch": null,

"searchable": "",

"pii": "No",

"qtip": "",

"used_by": "Consent Versions",

"used_by_display": "<a href='#' onClick='remoteJS({name:\"dialogs/AddSubjectTypeDlg\", obj_id:239 });'>Consent Versions</a>"

},

{

"id": 1229,

"obj_id": 1229,

"display_name": "Consultant",

"name": "Consultant",

"type": "Text Field",

"values": "",

"created_at": "05/31/2013",

"updated_at": "07/19/2013",

"created_by": [redacted],

"searchable_quick": false,

"searchable_advanced": null,

"searchable_batch": null,

"searchable": "",

"pii": "Yes",

"qtip": "",

"used_by": "NIH Visit, Patient Visit Tools, Consult Request, Patient Visit Planning for Version C",

"used_by_display": "<a href='#' onClick='remoteJS({name:\"dialogs/AddSubjectTypeDlg\", obj_id:140 });'>NIH Visit</a>, <a href='#' onClick='remoteJS({name:\"dialogs/AddSubjectTypeDlg\", obj_id:163 });'>Patient Visit Tools</a>, <a href='#' onClick='remoteJS({name:\"dialogs/AddSubjectTypeDlg\", obj_id:180 });'>Consult Request</a>, <a href='#' onClick='remoteJS({name:\"dialogs/AddSubjectTypeDlg\", obj_id:192 });'>Patient Visit Planning for Version C</a>"

},

{

"id": 2729,

"obj_id": 2729,

"display_name": "Consultant notes Ltr",

"name": "Consultant notes Ltr",

"type": "Text Field",

"values": "",

"created_at": "07/18/2014",

"updated_at": "07/18/2014",

"created_by": [redacted],

"searchable_quick": true,

"searchable_advanced": true,

"searchable_batch": true,

"searchable": "Quick,Advanced,Batch",

"pii": "No",

"qtip": "",

"used_by": "",

"used_by_display": ""

},

{

"id": 1232,

"obj_id": 1232,

"display_name": "Consultation Question:",

"name": "Consultation Question:",

"type": "Text Area",

"values": "",

"created_at": "05/31/2013",

"updated_at": "09/19/2013",

"created_by": [redacted],

"searchable_quick": false,

"searchable_advanced": null,

"searchable_batch": null,

"searchable": "",

"pii": "No",

"qtip": "",

"used_by": "NIH Visit, Patient Visit Tools, Consult Request, Patient Visit Planning for Version C",

"used_by_display": "<a href='#' onClick='remoteJS({name:\"dialogs/AddSubjectTypeDlg\", obj_id:140 });'>NIH Visit</a>, <a href='#' onClick='remoteJS({name:\"dialogs/AddSubjectTypeDlg\", obj_id:163 });'>Patient Visit Tools</a>, <a href='#' onClick='remoteJS({name:\"dialogs/AddSubjectTypeDlg\", obj_id:180 });'>Consult Request</a>, <a href='#' onClick='remoteJS({name:\"dialogs/AddSubjectTypeDlg\", obj_id:192 });'>Patient Visit Planning for Version C</a>"

},

{

"id": 1837,

"obj_id": 1837,

"display_name": "Consultation Request",

"name": "Consultation Request",

"type": "Subject Back Ref",

"values": "",

"created_at": "10/31/2013",

"updated_at": "12/08/2014",

"created_by": [redacted],

"searchable_quick": false,

"searchable_advanced": false,

"searchable_batch": false,

"searchable": "",

"pii": "No",

"qtip": "",

"used_by": "Patient, Patient Follow Up Visit",

"used_by_display": "<a href='#' onClick='remoteJS({name:\"dialogs/AddSubjectTypeDlg\", obj_id:1 });'>Patient</a>, <a href='#' onClick='remoteJS({name:\"dialogs/AddSubjectTypeDlg\", obj_id:264 });'>Patient Follow Up Visit</a>"

},

{

"id": 1248,

"obj_id": 1248,

"display_name": "Consultation Request Complete",

"name": "Consultation Request Complete",

"type": "Radio Buttons",

"values": "Yes, No",

"created_at": "06/03/2013",

"updated_at": "09/19/2013",

"created_by": [redacted],

"searchable_quick": false,

"searchable_advanced": null,

"searchable_batch": null,

"searchable": "",

"pii": "No",

"qtip": "",

"used_by": "NIH Visit, Patient Visit Tools, Consult Request, Patient Visit Planning for Version C",

"used_by_display": "<a href='#' onClick='remoteJS({name:\"dialogs/AddSubjectTypeDlg\", obj_id:140 });'>NIH Visit</a>, <a href='#' onClick='remoteJS({name:\"dialogs/AddSubjectTypeDlg\", obj_id:163 });'>Patient Visit Tools</a>, <a href='#' onClick='remoteJS({name:\"dialogs/AddSubjectTypeDlg\", obj_id:180 });'>Consult Request</a>, <a href='#' onClick='remoteJS({name:\"dialogs/AddSubjectTypeDlg\", obj_id:192 });'>Patient Visit Planning for Version C</a>"

},

{

"id": 1836,

"obj_id": 1836,

"display_name": "Consultation Request(s) Entered in LIMS",

"name": "Consultation Request(s) Entered in LIMS",

"type": "Radio Buttons",

"values": "Yes, No",

"created_at": "10/31/2013",

"updated_at": "10/31/2013",

"created_by": [redacted],

"searchable_quick": true,

"searchable_advanced": true,

"searchable_batch": true,

"searchable": "Quick,Advanced,Batch",

"pii": "No",

"qtip": "",

"used_by": "NIH Visit",

"used_by_display": "<a href='#' onClick='remoteJS({name:\"dialogs/AddSubjectTypeDlg\", obj_id:140 });'>NIH Visit</a>"

},

{

"id": 1234,

"obj_id": 1234,

"display_name": "Consultation Scheduled",

"name": "Consultation Scheduled",

"type": "Radio Buttons",

"values": "Yes, No",

"created_at": "05/31/2013",

"updated_at": "09/19/2013",

"created_by": [redacted],

"searchable_quick": false,

"searchable_advanced": null,

"searchable_batch": null,

"searchable": "",

"pii": "No",

"qtip": "",

"used_by": "NIH Visit, Patient Visit Tools, Consult Request, Patient Visit Planning for Version C",

"used_by_display": "<a href='#' onClick='remoteJS({name:\"dialogs/AddSubjectTypeDlg\", obj_id:140 });'>NIH Visit</a>, <a href='#' onClick='remoteJS({name:\"dialogs/AddSubjectTypeDlg\", obj_id:163 });'>Patient Visit Tools</a>, <a href='#' onClick='remoteJS({name:\"dialogs/AddSubjectTypeDlg\", obj_id:180 });'>Consult Request</a>, <a href='#' onClick='remoteJS({name:\"dialogs/AddSubjectTypeDlg\", obj_id:192 });'>Patient Visit Planning for Version C</a>"

},

{

"id": 1233,

"obj_id": 1233,

"display_name": "Consultation Summary Sent",

"name": "Consultation Summary Sent",

"type": "Radio Buttons",

"values": "Yes, No",

"created_at": "05/31/2013",

"updated_at": "09/19/2013",

"created_by": [redacted],

"searchable_quick": false,

"searchable_advanced": null,

"searchable_batch": null,

"searchable": "",

"pii": "No",

"qtip": "",

"used_by": "NIH Visit, Patient Visit Tools, Consult Request, Patient Visit Planning for Version C",

"used_by_display": "<a href='#' onClick='remoteJS({name:\"dialogs/AddSubjectTypeDlg\", obj_id:140 });'>NIH Visit</a>, <a href='#' onClick='remoteJS({name:\"dialogs/AddSubjectTypeDlg\", obj_id:163 });'>Patient Visit Tools</a>, <a href='#' onClick='remoteJS({name:\"dialogs/AddSubjectTypeDlg\", obj_id:180 });'>Consult Request</a>, <a href='#' onClick='remoteJS({name:\"dialogs/AddSubjectTypeDlg\", obj_id:192 });'>Patient Visit Planning for Version C</a>"

},

{

"id": 1549,

"obj_id": 1549,

"display_name": "Consult Notes",

"name": "Consult Notes",

"type": "Text Area",

"values": "",

"created_at": "08/07/2013",

"updated_at": "06/10/2014",

"created_by": [redacted],

"searchable_quick": false,

"searchable_advanced": null,

"searchable_batch": null,

"searchable": "",

"pii": "Yes",

"qtip": "",

"used_by": "Patient Visit Tools, Consult Request, Patient Visit Planning for Version C",

"used_by_display": "<a href='#' onClick='remoteJS({name:\"dialogs/AddSubjectTypeDlg\", obj_id:163 });'>Patient Visit Tools</a>, <a href='#' onClick='remoteJS({name:\"dialogs/AddSubjectTypeDlg\", obj_id:180 });'>Consult Request</a>, <a href='#' onClick='remoteJS({name:\"dialogs/AddSubjectTypeDlg\", obj_id:192 });'>Patient Visit Planning for Version C</a>"

},

{

"id": 2834,

"obj_id": 2834,

"display_name": "Consult Notes:",

"name": "Consult Notes:",

"type": "Text Area",

"values": "",

"created_at": "09/12/2014",

"updated_at": "09/12/2014",

"created_by": [redacted],

"searchable_quick": false,

"searchable_advanced": false,

"searchable_batch": false,

"searchable": "",

"pii": "Yes",

"qtip": "",

"used_by": "Clinical Notes",

"used_by_display": "<a href='#' onClick='remoteJS({name:\"dialogs/AddSubjectTypeDlg\", obj_id:267 });'>Clinical Notes</a>"

},

{

"id": 1963,

"obj_id": 1963,

"display_name": "Consult Request",

"name": "Consult Request",

"type": "Subject Back Ref",

"values": "",

"created_at": "11/14/2013",

"updated_at": "12/08/2014",

"created_by": [redacted],

"searchable_quick": false,

"searchable_advanced": false,

"searchable_batch": false,

"searchable": "",

"pii": "No",

"qtip": "",

"used_by": "Patient, Patient Follow Up Visit",

"used_by_display": "<a href='#' onClick='remoteJS({name:\"dialogs/AddSubjectTypeDlg\", obj_id:1 });'>Patient</a>, <a href='#' onClick='remoteJS({name:\"dialogs/AddSubjectTypeDlg\", obj_id:264 });'>Patient Follow Up Visit</a>"

},

{

"id": 1806,

"obj_id": 1806,

"display_name": "Contact",

"name": "Contact",

"type": "Text Field",

"values": "",

"created_at": "10/29/2013",

"updated_at": "06/09/2014",

"created_by": [redacted],

"searchable_quick": false,

"searchable_advanced": null,

"searchable_batch": null,

"searchable": "",

"pii": "Yes",

"qtip": "",

"used_by": "Labmatrix Legacy - Persons Contact",

"used_by_display": "<a href='#' onClick='remoteJS({name:\"dialogs/AddSubjectTypeDlg\", obj_id:95 });'>Labmatrix Legacy - Persons Contact</a>"

},

{

"id": 190,

"obj_id": 190,

"display_name": "Contact #",

"name": "Contact #",

"type": "Text Field",

"values": "",

"created_at": "03/20/2013",

"updated_at": "07/19/2013",

"created_by": [redacted],

"searchable_quick": false,

"searchable_advanced": null,

"searchable_batch": null,

"searchable": "",

"pii": "Yes",

"qtip": "",

"used_by": "Patient, Patient Follow Up Visit",

"used_by_display": "<a href='#' onClick='remoteJS({name:\"dialogs/AddSubjectTypeDlg\", obj_id:1 });'>Patient</a>, <a href='#' onClick='remoteJS({name:\"dialogs/AddSubjectTypeDlg\", obj_id:264 });'>Patient Follow Up Visit</a>"

},

{

"id": 2588,

"obj_id": 2588,

"display_name": "Contact about additional genetic findings",

"name": "Contact about additional genetic findings",

"type": "Choice",

"values": "Yes, No, Not completed",

"created_at": "05/02/2014",

"updated_at": "05/02/2014",

"created_by": [redacted],

"searchable_quick": false,

"searchable_advanced": null,

"searchable_batch": null,

"searchable": "",

"pii": "No",

"qtip": "",

"used_by": "Consent Signed",

"used_by_display": "<a href='#' onClick='remoteJS({name:\"dialogs/AddSubjectTypeDlg\", obj_id:141 });'>Consent Signed</a>"

},

{

"id": 522,

"obj_id": 522,

"display_name": "Contact Access Group Id List",

"name": "Contact Access Group Id List",

"type": "Text Area",

"values": "",

"created_at": "05/07/2013",

"updated_at": "09/19/2013",

"created_by": [redacted],

"searchable_quick": false,

"searchable_advanced": null,

"searchable_batch": null,

"searchable": "",

"pii": "No",

"qtip": "",

"used_by": "Labmatrix Legacy - Communications",

"used_by_display": "<a href='#' onClick='remoteJS({name:\"dialogs/AddSubjectTypeDlg\", obj_id:92 });'>Labmatrix Legacy - Communications</a>"

},

{

"id": 1852,

"obj_id": 1852,

"display_name": "Contact City",

"name": "Contact City",

"type": "Text Field",

"values": "",

"created_at": "11/01/2013",

"updated_at": "11/01/2013",

"created_by": [redacted],

"searchable_quick": true,

"searchable_advanced": true,

"searchable_batch": true,

"searchable": "Quick,Advanced,Batch",

"pii": "Yes",

"qtip": "",

"used_by": "UDP Inquiry",

"used_by_display": "<a href='#' onClick='remoteJS({name:\"dialogs/AddSubjectTypeDlg\", obj_id:61 });'>UDP Inquiry</a>"

},

{

"id": 1848,

"obj_id": 1848,

"display_name": "Contact Email",

"name": "Contact Email",

"type": "Text Field",

"values": "",

"created_at": "11/01/2013",

"updated_at": "11/01/2013",

"created_by": [redacted],

"searchable_quick": true,

"searchable_advanced": true,

"searchable_batch": true,

"searchable": "Quick,Advanced,Batch",

"pii": "Yes",

"qtip": "",

"used_by": "UDP Inquiry",

"used_by_display": "<a href='#' onClick='remoteJS({name:\"dialogs/AddSubjectTypeDlg\", obj_id:61 });'>UDP Inquiry</a>"

},

{

"id": 1163,

"obj_id": 1163,

"display_name": "Contact Family with list of medical records still needed",

"name": "Contact Family with list of medical records still needed",

"type": "CheckBox",

"values": "",

"created_at": "05/28/2013",

"updated_at": "09/19/2013",

"created_by": [redacted],

"searchable_quick": false,

"searchable_advanced": null,

"searchable_batch": null,

"searchable": "",

"pii": "No",

"qtip": "",

"used_by": "Patient Visit Tools, Patient Visit Planning for Version C",

"used_by_display": "<a href='#' onClick='remoteJS({name:\"dialogs/AddSubjectTypeDlg\", obj_id:163 });'>Patient Visit Tools</a>, <a href='#' onClick='remoteJS({name:\"dialogs/AddSubjectTypeDlg\", obj_id:192 });'>Patient Visit Planning for Version C</a>"

},

{

"id": 523,

"obj_id": 523,

"display_name": "Contact Id",

"name": "Contact Id",

"type": "Number",

"values": "",

"created_at": "05/07/2013",

"updated_at": "07/19/2013",

"created_by": [redacted],

"searchable_quick": false,

"searchable_advanced": null,

"searchable_batch": null,

"searchable": "",

"pii": "Yes",

"qtip": "",

"used_by": "Labmatrix Legacy - Communications",

"used_by_display": "<a href='#' onClick='remoteJS({name:\"dialogs/AddSubjectTypeDlg\", obj_id:92 });'>Labmatrix Legacy - Communications</a>"

},

{

"id": 430,

"obj_id": 430,

"display_name": "Contact Info",

"name": "Contact Info",

"type": "Text Area",

"values": "",

"created_at": "04/24/2013",

"updated_at": "07/19/2013",

"created_by": [redacted],

"searchable_quick": false,

"searchable_advanced": null,

"searchable_batch": null,

"searchable": "",

"pii": "Yes",

"qtip": "",

"used_by": "Question to Primary Care /Family",

"used_by_display": "<a href='#' onClick='remoteJS({name:\"dialogs/AddSubjectTypeDlg\", obj_id:84 });'>Question to Primary Care /Family</a>"

},

{

"id": 313,

"obj_id": 313,

"display_name": "Contact Information",

"name": "Contact Information",

"type": "Text Field",

"values": "",

"created_at": "04/05/2013",

"updated_at": "06/06/2014",

"created_by": [redacted],

"searchable_quick": false,

"searchable_advanced": null,

"searchable_batch": null,

"searchable": "",

"pii": "Yes",

"qtip": "Email or phone number",

"used_by": "Patient Review",

"used_by_display": "<a href='#' onClick='remoteJS({name:\"dialogs/AddSubjectTypeDlg\", obj_id:83 });'>Patient Review</a>"

},

{

"id": 1079,

"obj_id": 1079,

"display_name": "Contact Information - Email",

"name": "Contact Information - Email",

"type": "Text Field",

"values": "",

"created_at": "05/15/2013",

"updated_at": "07/19/2013",

"created_by": [redacted],

"searchable_quick": false,

"searchable_advanced": null,

"searchable_batch": null,

"searchable": "",

"pii": "Yes",

"qtip": "",

"used_by": "Sequencing Centers",

"used_by_display": "<a href='#' onClick='remoteJS({name:\"dialogs/AddSubjectTypeDlg\", obj_id:196 });'>Sequencing Centers</a>"

},

{

"id": 2057,

"obj_id": 2057,

"display_name": "Contact Information - Fax",

"name": "Contact Information - Fax",

"type": "Text Field",

"values": "",

"created_at": "12/04/2013",

"updated_at": "06/09/2014",

"created_by": [redacted],

"searchable_quick": false,

"searchable_advanced": null,

"searchable_batch": null,

"searchable": "",

"pii": "Yes",

"qtip": "",

"used_by": "Sequencing Centers",

"used_by_display": "<a href='#' onClick='remoteJS({name:\"dialogs/AddSubjectTypeDlg\", obj_id:196 });'>Sequencing Centers</a>"

},

{

"id": 1402,

"obj_id": 1402,

"display_name": "Contact Information - Phone",

"name": "Contact Information - Phone",

"type": "Text Field",

"values": "",

"created_at": "07/08/2013",

"updated_at": "07/19/2013",

"created_by": [redacted],

"searchable_quick": false,

"searchable_advanced": null,

"searchable_batch": null,

"searchable": "",

"pii": "Yes",

"qtip": "",

"used_by": "Sequencing Centers",

"used_by_display": "<a href='#' onClick='remoteJS({name:\"dialogs/AddSubjectTypeDlg\", obj_id:196 });'>Sequencing Centers</a>"

},

{

"id": 524,

"obj_id": 524,

"display_name": "Contact is Primary",

"name": "Contact is Primary",

"type": "Text Field",

"values": "",

"created_at": "05/07/2013",

"updated_at": "09/19/2013",

"created_by": [redacted],

"searchable_quick": false,

"searchable_advanced": null,

"searchable_batch": null,

"searchable": "",

"pii": "No",

"qtip": "",

"used_by": "Labmatrix Legacy - Persons Contact",

"used_by_display": "<a href='#' onClick='remoteJS({name:\"dialogs/AddSubjectTypeDlg\", obj_id:95 });'>Labmatrix Legacy - Persons Contact</a>"

},

{

"id": 525,

"obj_id": 525,

"display_name": "Contact is Subject",

"name": "Contact is Subject",

"type": "Text Field",

"values": "",

"created_at": "05/07/2013",

"updated_at": "09/19/2013",

"created_by": [redacted],

"searchable_quick": false,

"searchable_advanced": null,

"searchable_batch": null,

"searchable": "",

"pii": "No",

"qtip": "",

"used_by": "Labmatrix Legacy - Persons Contact",

"used_by_display": "<a href='#' onClick='remoteJS({name:\"dialogs/AddSubjectTypeDlg\", obj_id:95 });'>Labmatrix Legacy - Persons Contact</a>"

},

{

"id": 54,

"obj_id": 54,

"display_name": "Contact Name",

"name": "Contact Name",

"type": "Text Field",

"values": "",

"created_at": "02/05/2013",

"updated_at": "11/01/2013",

"created_by": [redacted],

"searchable_quick": true,

"searchable_advanced": true,

"searchable_batch": true,

"searchable": "Quick,Advanced,Batch",

"pii": "Yes",

"qtip": "",

"used_by": "Patient, UDP Inquiry, Patient Follow Up Visit",

"used_by_display": "<a href='#' onClick='remoteJS({name:\"dialogs/AddSubjectTypeDlg\", obj_id:1 });'>Patient</a>, <a href='#' onClick='remoteJS({name:\"dialogs/AddSubjectTypeDlg\", obj_id:61 });'>UDP Inquiry</a>, <a href='#' onClick='remoteJS({name:\"dialogs/AddSubjectTypeDlg\", obj_id:264 });'>Patient Follow Up Visit</a>"

},

{

"id": 1849,

"obj_id": 1849,

"display_name": "Contact Phone",

"name": "Contact Phone",

"type": "Text Field",

"values": "",

"created_at": "11/01/2013",

"updated_at": "11/01/2013",

"created_by": [redacted],

"searchable_quick": true,

"searchable_advanced": true,

"searchable_batch": true,

"searchable": "Quick,Advanced,Batch",

"pii": "Yes",

"qtip": "",

"used_by": "UDP Inquiry",

"used_by_display": "<a href='#' onClick='remoteJS({name:\"dialogs/AddSubjectTypeDlg\", obj_id:61 });'>UDP Inquiry</a>"

},

{

"id": 1853,

"obj_id": 1853,

"display_name": "Contact State",

"name": "Contact State",

"type": "Text Field",

"values": "",

"created_at": "11/01/2013",

"updated_at": "11/01/2013",

"created_by": [redacted],

"searchable_quick": true,

"searchable_advanced": true,

"searchable_batch": true,

"searchable": "Quick,Advanced,Batch",

"pii": "Yes",

"qtip": "",

"used_by": "UDP Inquiry",

"used_by_display": "<a href='#' onClick='remoteJS({name:\"dialogs/AddSubjectTypeDlg\", obj_id:61 });'>UDP Inquiry</a>"

},

{

"id": 1851,

"obj_id": 1851,

"display_name": "Contact Street Address",

"name": "Contact Street Address",

"type": "Text Field",

"values": "",

"created_at": "11/01/2013",

"updated_at": "11/01/2013",

"created_by": [redacted],

"searchable_quick": true,

"searchable_advanced": true,

"searchable_batch": true,

"searchable": "Quick,Advanced,Batch",

"pii": "Yes",

"qtip": "",

"used_by": "UDP Inquiry",

"used_by_display": "<a href='#' onClick='remoteJS({name:\"dialogs/AddSubjectTypeDlg\", obj_id:61 });'>UDP Inquiry</a>"

},

{

"id": 526,

"obj_id": 526,

"display_name": "Contact To From About",

"name": "Contact To From About",

"type": "Text Area",

"values": "",

"created_at": "05/07/2013",

"updated_at": "09/19/2013",

"created_by": [redacted],

"searchable_quick": false,

"searchable_advanced": null,

"searchable_batch": null,

"searchable": "",

"pii": "No",

"qtip": "",

"used_by": "Labmatrix Legacy - Communications",

"used_by_display": "<a href='#' onClick='remoteJS({name:\"dialogs/AddSubjectTypeDlg\", obj_id:92 });'>Labmatrix Legacy - Communications</a>"

},

{

"id": 2201,

"obj_id": 2201,

"display_name": "Contact type",

"name": "Contact type",

"type": "Text Field",

"values": "",

"created_at": "02/03/2014",

"updated_at": "06/09/2014",

"created_by": [redacted],

"searchable_quick": false,

"searchable_advanced": null,

"searchable_batch": null,

"searchable": "",

"pii": "Yes",

"qtip": "Labmatrix legacy field",

"used_by": "Labmatrix Legacy - Persons Contact",

"used_by_display": "<a href='#' onClick='remoteJS({name:\"dialogs/AddSubjectTypeDlg\", obj_id:95 });'>Labmatrix Legacy - Persons Contact</a>"

},

{

"id": 187,

"obj_id": 187,

"display_name": "Contact Type",

"name": "Contact Type",

"type": "Radio Buttons",

"values": "Telephone, Letter, Records",

"created_at": "03/20/2013",

"updated_at": "09/19/2013",

"created_by": [redacted],

"searchable_quick": false,

"searchable_advanced": null,

"searchable_batch": null,

"searchable": "",

"pii": "No",

"qtip": "",

"used_by": "Patient, Labmatrix Legacy - Persons Contact, Patient Follow Up Visit",

"used_by_display": "<a href='#' onClick='remoteJS({name:\"dialogs/AddSubjectTypeDlg\", obj_id:1 });'>Patient</a>, <a href='#' onClick='remoteJS({name:\"dialogs/AddSubjectTypeDlg\", obj_id:95 });'>Labmatrix Legacy - Persons Contact</a>, <a href='#' onClick='remoteJS({name:\"dialogs/AddSubjectTypeDlg\", obj_id:264 });'>Patient Follow Up Visit</a>"

},

{

"id": 1850,

"obj_id": 1850,

"display_name": "Contact Work Phone",

"name": "Contact Work Phone",

"type": "Text Field",

"values": "",

"created_at": "11/01/2013",

"updated_at": "11/01/2013",

"created_by": [redacted],

"searchable_quick": true,

"searchable_advanced": true,

"searchable_batch": true,

"searchable": "Quick,Advanced,Batch",

"pii": "Yes",

"qtip": "",

"used_by": "UDP Inquiry",

"used_by_display": "<a href='#' onClick='remoteJS({name:\"dialogs/AddSubjectTypeDlg\", obj_id:61 });'>UDP Inquiry</a>"

},

{

"id": 1854,

"obj_id": 1854,

"display_name": "Contact Zip Code",

"name": "Contact Zip Code",

"type": "Text Field",

"values": "",

"created_at": "11/01/2013",

"updated_at": "11/01/2013",

"created_by": [redacted],

"searchable_quick": true,

"searchable_advanced": true,

"searchable_batch": true,

"searchable": "Quick,Advanced,Batch",

"pii": "Yes",

"qtip": "",

"used_by": "UDP Inquiry",

"used_by_display": "<a href='#' onClick='remoteJS({name:\"dialogs/AddSubjectTypeDlg\", obj_id:61 });'>UDP Inquiry</a>"

},

{

"id": 527,

"obj_id": 527,

"display_name": "Content",

"name": "Content",

"type": "Text Area",

"values": "",

"created_at": "05/07/2013",

"updated_at": "09/19/2013",

"created_by": [redacted],

"searchable_quick": false,

"searchable_advanced": null,

"searchable_batch": null,

"searchable": "",

"pii": "No",

"qtip": "",

"used_by": "Labmatrix Legacy - Communications",

"used_by_display": "<a href='#' onClick='remoteJS({name:\"dialogs/AddSubjectTypeDlg\", obj_id:92 });'>Labmatrix Legacy - Communications</a>"

},

{

"id": 2587,

"obj_id": 2587,

"display_name": "Contracted?",

"name": "Contracted?",

"type": "Choice",

"values": "Yes, No",

"created_at": "05/02/2014",

"updated_at": "06/09/2014",

"created_by": [redacted],

"searchable_quick": false,

"searchable_advanced": null,

"searchable_batch": null,

"searchable": "",

"pii": "Yes",

"qtip": "",

"used_by": "Exome Analysis Results",

"used_by_display": "<a href='#' onClick='remoteJS({name:\"dialogs/AddSubjectTypeDlg\", obj_id:156 });'>Exome Analysis Results</a>"

},

{

"id": 2316,

"obj_id": 2316,

"display_name": "Control Subject",

"name": "Control Subject",

"type": "CheckBox",

"values": "",

"created_at": "03/04/2014",

"updated_at": "03/04/2014",

"created_by": [redacted],

"searchable_quick": true,

"searchable_advanced": true,

"searchable_batch": true,

"searchable": "Quick,Advanced,Batch",

"pii": "No",

"qtip": "",

"used_by": "Patient, Cell Culture, Rescue Cell Culture, Patient Follow Up Visit",

"used_by_display": "<a href='#' onClick='remoteJS({name:\"dialogs/AddSubjectTypeDlg\", obj_id:1 });'>Patient</a>, <a href='#' onClick='remoteJS({name:\"dialogs/AddSubjectTypeDlg\", obj_id:40 });'>Cell Culture</a>, <a href='#' onClick='remoteJS({name:\"dialogs/AddSubjectTypeDlg\", obj_id:247 });'>Rescue Cell Culture</a>, <a href='#' onClick='remoteJS({name:\"dialogs/AddSubjectTypeDlg\", obj_id:264 });'>Patient Follow Up Visit</a>"

},

{

"id": 1465,

"obj_id": 1465,

"display_name": "Copy Number",

"name": "Copy Number",

"type": "Text Field",

"values": "",

"created_at": "07/18/2013",

"updated_at": "09/19/2013",

"created_by": [redacted],

"searchable_quick": false,

"searchable_advanced": null,

"searchable_batch": null,

"searchable": "",

"pii": "No",

"qtip": "CN",

"used_by": "SNP Report",

"used_by_display": "<a href='#' onClick='remoteJS({name:\"dialogs/AddSubjectTypeDlg\", obj_id:155 });'>SNP Report</a>"

},

{

"id": 1265,

"obj_id": 1265,

"display_name": "Copy Scanned",

"name": "Copy Scanned",

"type": "Radio Buttons",

"values": "Yes, No",

"created_at": "06/03/2013",

"updated_at": "09/19/2013",

"created_by": [redacted],

"searchable_quick": false,

"searchable_advanced": null,

"searchable_batch": null,

"searchable": "",

"pii": "No",

"qtip": "",

"used_by": "NIH Visit",

"used_by_display": "<a href='#' onClick='remoteJS({name:\"dialogs/AddSubjectTypeDlg\", obj_id:140 });'>NIH Visit</a>"

},

{

"id": 1264,

"obj_id": 1264,

"display_name": "Copy sent to Medical Records",

"name": "Copy sent to Medical Records",

"type": "Radio Buttons",

"values": "Yes, No",

"created_at": "06/03/2013",

"updated_at": "09/19/2013",

"created_by": [redacted],

"searchable_quick": false,

"searchable_advanced": null,

"searchable_batch": null,

"searchable": "",

"pii": "No",

"qtip": "",

"used_by": "NIH Visit",

"used_by_display": "<a href='#' onClick='remoteJS({name:\"dialogs/AddSubjectTypeDlg\", obj_id:140 });'>NIH Visit</a>"

},

{

"id": 1765,

"obj_id": 1765,

"display_name": "Correction of Parental Relationship Approved",

"name": "Correction of Parental Relationship Approved",

"type": "CheckBox",

"values": "",

"created_at": "10/22/2013",

"updated_at": "12/04/2013",

"created_by": [redacted],

"searchable_quick": true,

"searchable_advanced": true,

"searchable_batch": true,

"searchable": "Quick,Advanced,Batch",

"pii": "No",

"qtip": "",

"used_by": "SNP Analysis, Exome Analysis",

"used_by_display": "<a href='#' onClick='remoteJS({name:\"dialogs/AddSubjectTypeDlg\", obj_id:59 });'>SNP Analysis</a>, <a href='#' onClick='remoteJS({name:\"dialogs/AddSubjectTypeDlg\", obj_id:60 });'>Exome Analysis</a>"

},

{

"id": 1138,

"obj_id": 1138,

"display_name": "Cough Assist and/or CF Vest?",

"name": "Cough Assist and/or CF Vest?",

"type": "Radio Buttons",

"values": "Yes, No, Unassessed",

"created_at": "05/28/2013",

"updated_at": "09/19/2013",

"created_by": [redacted],

"searchable_quick": false,

"searchable_advanced": null,

"searchable_batch": null,

"searchable": "",

"pii": "No",

"qtip": "If yes, change Safety Status to Moderate or High, create PIFUT for plan for Travel, NIH stay",

"used_by": "Patient Visit Tools, Patient Visit Planning for Version C",

"used_by_display": "<a href='#' onClick='remoteJS({name:\"dialogs/AddSubjectTypeDlg\", obj_id:163 });'>Patient Visit Tools</a>, <a href='#' onClick='remoteJS({name:\"dialogs/AddSubjectTypeDlg\", obj_id:192 });'>Patient Visit Planning for Version C</a>"

},

{

"id": 528,

"obj_id": 528,

"display_name": "Country",

"name": "Country",

"type": "Text Field",

"values": "",

"created_at": "05/07/2013",

"updated_at": "11/01/2013",

"created_by": [redacted],

"searchable_quick": true,

"searchable_advanced": true,

"searchable_batch": true,

"searchable": "Quick,Advanced,Batch",

"pii": "Yes",

"qtip": "",

"used_by": "Labmatrix Legacy - Organization Contacts, Patient Follow Up Visit, UDP Inquiry, Patient",

"used_by_display": "<a href='#' onClick='remoteJS({name:\"dialogs/AddSubjectTypeDlg\", obj_id:94 });'>Labmatrix Legacy - Organization Contacts</a>, <a href='#' onClick='remoteJS({name:\"dialogs/AddSubjectTypeDlg\", obj_id:264 });'>Patient Follow Up Visit</a>, <a href='#' onClick='remoteJS({name:\"dialogs/AddSubjectTypeDlg\", obj_id:61 });'>UDP Inquiry</a>, <a href='#' onClick='remoteJS({name:\"dialogs/AddSubjectTypeDlg\", obj_id:1 });'>Patient</a>"

},

{

"id": 2744,

"obj_id": 2744,

"display_name": "c.Position",

"name": "c.Position",

"type": "Text Field",

"values": "",

"created_at": "07/23/2014",

"updated_at": "11/19/2014",

"created_by": [redacted],

"searchable_quick": false,

"searchable_advanced": true,

"searchable_batch": false,

"searchable": "Advanced",

"pii": "No",

"qtip": "cDNA transcript position",

"used_by": "Exome Analysis Results, Variant Prioritization",

"used_by_display": "<a href='#' onClick='remoteJS({name:\"dialogs/AddSubjectTypeDlg\", obj_id:156 });'>Exome Analysis Results</a>, <a href='#' onClick='remoteJS({name:\"dialogs/AddSubjectTypeDlg\", obj_id:279 });'>Variant Prioritization</a>"

},

{

"id": 529,

"obj_id": 529,

"display_name": "Create Date",

"name": "Create Date",

"type": "Date",

"values": "",

"created_at": "05/07/2013",

"updated_at": "06/10/2014",

"created_by": [redacted],

"searchable_quick": false,

"searchable_advanced": null,

"searchable_batch": null,

"searchable": "",

"pii": "Yes",

"qtip": "",

"used_by": "Labmatrix Legacy - Communications",

"used_by_display": "<a href='#' onClick='remoteJS({name:\"dialogs/AddSubjectTypeDlg\", obj_id:92 });'>Labmatrix Legacy - Communications</a>"

},

{

"id": 934,

"obj_id": 934,

"display_name": "Created By",

"name": "Created By",

"type": "Text Field",

"values": "",

"created_at": "05/10/2013",

"updated_at": "09/19/2013",

"created_by": [redacted],

"searchable_quick": false,

"searchable_advanced": null,

"searchable_batch": null,

"searchable": "",

"pii": "No",

"qtip": "",

"used_by": "Sharepoint Legacy - Pediatric Patient, Sharepoint Legacy - Adult Patient Status, Sharepoint Legacy - Pediatric Task List",

"used_by_display": "<a href='#' onClick='remoteJS({name:\"dialogs/AddSubjectTypeDlg\", obj_id:114 });'>Sharepoint Legacy - Pediatric Patient</a>, <a href='#' onClick='remoteJS({name:\"dialogs/AddSubjectTypeDlg\", obj_id:115 });'>Sharepoint Legacy - Adult Patient Status</a>, <a href='#' onClick='remoteJS({name:\"dialogs/AddSubjectTypeDlg\", obj_id:159 });'>Sharepoint Legacy - Pediatric Task List</a>"

},

{

"id": 530,

"obj_id": 530,

"display_name": "Created by Organization ID",

"name": "Created by Organization ID",

"type": "Number",

"values": "",

"created_at": "05/07/2013",

"updated_at": "09/19/2013",

"created_by": [redacted],

"searchable_quick": false,

"searchable_advanced": null,

"searchable_batch": null,

"searchable": "",

"pii": "No",

"qtip": "",

"used_by": "Labmatrix Legacy - Biomaterials",

"used_by_display": "<a href='#' onClick='remoteJS({name:\"dialogs/AddSubjectTypeDlg\", obj_id:91 });'>Labmatrix Legacy - Biomaterials</a>"

},

{

"id": 531,

"obj_id": 531,

"display_name": "Created By Person ID",

"name": "Created By Person ID",

"type": "Number",

"values": "",

"created_at": "05/07/2013",

"updated_at": "07/29/2013",

"created_by": [redacted],

"searchable_quick": false,

"searchable_advanced": null,

"searchable_batch": null,

"searchable": "",

"pii": "No",

"qtip": "",

"used_by": "Labmatrix Legacy - Biomaterials",

"used_by_display": "<a href='#' onClick='remoteJS({name:\"dialogs/AddSubjectTypeDlg\", obj_id:91 });'>Labmatrix Legacy - Biomaterials</a>"

},

{

"id": 1375,

"obj_id": 1375,

"display_name": "Create Task/Consultation Request as Needed",

"name": "Create Task/Consultation Request as Needed",

"type": "List",

"values": "Ophthamology - email consult request(scheduled not required), Swallow/Speech eval, Photography (phone call), Nutrition (email [redacted], include special diets), Neurology, EEG/EMG (contact 7SW neuro testing), Schedule LP/Skin Biopsy if no sedation, Neuropsych (email), Audioology (5NW)",

"created_at": "06/24/2013",

"updated_at": "01/23/2015",

"created_by": [redacted],

"searchable_quick": true,

"searchable_advanced": true,

"searchable_batch": true,

"searchable": "Quick,Advanced,Batch",

"pii": "Yes",

"qtip": "",

"used_by": "Patient Visit Tools, Patient Visit Planning for Version C",

"used_by_display": "<a href='#' onClick='remoteJS({name:\"dialogs/AddSubjectTypeDlg\", obj_id:163 });'>Patient Visit Tools</a>, <a href='#' onClick='remoteJS({name:\"dialogs/AddSubjectTypeDlg\", obj_id:192 });'>Patient Visit Planning for Version C</a>"

},

{

"id": 532,

"obj_id": 532,

"display_name": "Creator",

"name": "Creator",

"type": "Text Field",

"values": "",

"created_at": "05/07/2013",

"updated_at": "09/19/2013",

"created_by": [redacted],

"searchable_quick": false,

"searchable_advanced": null,

"searchable_batch": null,

"searchable": "",

"pii": "No",

"qtip": "",

"used_by": "Labmatrix Legacy - Communications",

"used_by_display": "<a href='#' onClick='remoteJS({name:\"dialogs/AddSubjectTypeDlg\", obj_id:92 });'>Labmatrix Legacy - Communications</a>"

},

{

"id": 2738,

"obj_id": 2738,

"display_name": "CRISPR Design Notes",

"name": "CRISPR Design Notes",

"type": "Text Area",

"values": "",

"created_at": "07/18/2014",

"updated_at": "07/18/2014",

"created_by": [redacted],

"searchable_quick": false,

"searchable_advanced": false,

"searchable_batch": false,

"searchable": "",

"pii": "No",

"qtip": "Design and Synthesis",

"used_by": "Zebrafish Mutation Project",

"used_by_display": "<a href='#' onClick='remoteJS({name:\"dialogs/AddSubjectTypeDlg\", obj_id:186 });'>Zebrafish Mutation Project</a>"

},

{

"id": 1969,

"obj_id": 1969,

"display_name": "CRISPRs ",

"name": "CRISPRs ",

"type": "Text Area",

"values": "",

"created_at": "11/18/2013",

"updated_at": "11/18/2013",

"created_by": [redacted],

"searchable_quick": false,

"searchable_advanced": null,

"searchable_batch": null,

"searchable": "",

"pii": "No",

"qtip": "Primers",

"used_by": "Zebrafish Mutation Project",

"used_by_display": "<a href='#' onClick='remoteJS({name:\"dialogs/AddSubjectTypeDlg\", obj_id:186 });'>Zebrafish Mutation Project</a>"

},

{

"id": 2230,

"obj_id": 2230,

"display_name": "CRISPR target",

"name": "CRISPR target",

"type": "Text Area",

"values": "",

"created_at": "02/07/2014",

"updated_at": "02/27/2014",

"created_by": [redacted],

"searchable_quick": false,

"searchable_advanced": null,

"searchable_batch": null,

"searchable": "",

"pii": "No",

"qtip": "",

"used_by": "Zebrafish Mutation Project",

"used_by_display": "<a href='#' onClick='remoteJS({name:\"dialogs/AddSubjectTypeDlg\", obj_id:186 });'>Zebrafish Mutation Project</a>"

},

{

"id": 1308,

"obj_id": 1308,

"display_name": "CSF Cell Count",

"name": "CSF Cell Count",

"type": "Text Field",

"values": "",

"created_at": "06/10/2013",

"updated_at": "10/29/2014",

"created_by": [redacted],

"searchable_quick": false,

"searchable_advanced": true,

"searchable_batch": false,

"searchable": "Advanced",

"pii": "No",

"qtip": "",

"used_by": "Research Sample",

"used_by_display": "<a href='#' onClick='remoteJS({name:\"dialogs/AddSubjectTypeDlg\", obj_id:86 });'>Research Sample</a>"

},

{

"id": 2577,

"obj_id": 2577,

"display_name": "CSF collection and sharing",

"name": "CSF collection and sharing",

"type": "Choice",

"values": "Yes, No",

"created_at": "05/01/2014",

"updated_at": "05/01/2014",

"created_by": [redacted],

"searchable_quick": false,

"searchable_advanced": null,

"searchable_batch": null,

"searchable": "",

"pii": "No",

"qtip": "",

"used_by": "Consent Versions",

"used_by_display": "<a href='#' onClick='remoteJS({name:\"dialogs/AddSubjectTypeDlg\", obj_id:239 });'>Consent Versions</a>"

},

{

"id": 1307,

"obj_id": 1307,

"display_name": "CSF Glucose",

"name": "CSF Glucose",

"type": "Text Field",

"values": "",

"created_at": "06/10/2013",

"updated_at": "10/29/2014",

"created_by": [redacted],

"searchable_quick": false,

"searchable_advanced": true,

"searchable_batch": false,

"searchable": "Advanced",

"pii": "No",

"qtip": "",

"used_by": "Research Sample",

"used_by_display": "<a href='#' onClick='remoteJS({name:\"dialogs/AddSubjectTypeDlg\", obj_id:86 });'>Research Sample</a>"

},

{

"id": 2822,

"obj_id": 2822,

"display_name": "CSF N-linked Glycomics",

"name": "CSF N-linked Glycomics",

"type": "CheckBox",

"values": "",

"created_at": "09/09/2014",

"updated_at": "09/09/2014",

"created_by": [redacted],

"searchable_quick": false,

"searchable_advanced": false,

"searchable_batch": false,

"searchable": "",

"pii": "No",

"qtip": "",

"used_by": "Glycomics Collaborations",

"used_by_display": "<a href='#' onClick='remoteJS({name:\"dialogs/AddSubjectTypeDlg\", obj_id:244 });'>Glycomics Collaborations</a>"

},

{

"id": 2746,

"obj_id": 2746,

"display_name": "CSF N-linked Results",

"name": "CSF N-linked Results",

"type": "Text Field",

"values": "",

"created_at": "07/23/2014",

"updated_at": "07/30/2014",

"created_by": [redacted],

"searchable_quick": false,

"searchable_advanced": false,

"searchable_batch": false,

"searchable": "",

"pii": "No",

"qtip": "",

"used_by": "Glycome Patient",

"used_by_display": "<a href='#' onClick='remoteJS({name:\"dialogs/AddSubjectTypeDlg\", obj_id:224 });'>Glycome Patient</a>"

},

{

"id": 2855,

"obj_id": 2855,

"display_name": "CSF oligosaccharides Results",

"name": "CSF oligosaccharides Results",

"type": "Text Field",

"values": "",

"created_at": "10/07/2014",

"updated_at": "10/07/2014",

"created_by": [redacted],

"searchable_quick": false,

"searchable_advanced": true,

"searchable_batch": false,

"searchable": "Advanced",

"pii": "No",

"qtip": "",

"used_by": "Glycome Patient",

"used_by_display": "<a href='#' onClick='remoteJS({name:\"dialogs/AddSubjectTypeDlg\", obj_id:224 });'>Glycome Patient</a>"

},

{

"id": 2823,

"obj_id": 2823,

"display_name": "CSF O-linked Glycomics",

"name": "CSF O-linked Glycomics",

"type": "CheckBox",

"values": "",

"created_at": "09/09/2014",

"updated_at": "09/09/2014",

"created_by": [redacted],

"searchable_quick": false,

"searchable_advanced": false,

"searchable_batch": false,

"searchable": "",

"pii": "No",

"qtip": "",

"used_by": "Glycomics Collaborations",

"used_by_display": "<a href='#' onClick='remoteJS({name:\"dialogs/AddSubjectTypeDlg\", obj_id:244 });'>Glycomics Collaborations</a>"

},

{

"id": 2747,

"obj_id": 2747,

"display_name": "CSF O-linked Results",

"name": "CSF O-linked Results",

"type": "Text Field",

"values": "",

"created_at": "07/23/2014",

"updated_at": "07/23/2014",

"created_by": [redacted],

"searchable_quick": false,

"searchable_advanced": false,

"searchable_batch": false,

"searchable": "",

"pii": "No",

"qtip": "",

"used_by": "Glycome Patient",

"used_by_display": "<a href='#' onClick='remoteJS({name:\"dialogs/AddSubjectTypeDlg\", obj_id:224 });'>Glycome Patient</a>"

},

{

"id": 1306,

"obj_id": 1306,

"display_name": "CSF Protein",

"name": "CSF Protein",

"type": "Text Field",

"values": "",

"created_at": "06/10/2013",

"updated_at": "10/29/2014",

"created_by": [redacted],

"searchable_quick": false,

"searchable_advanced": true,

"searchable_batch": false,

"searchable": "Advanced",

"pii": "No",

"qtip": "",

"used_by": "Research Sample",

"used_by_display": "<a href='#' onClick='remoteJS({name:\"dialogs/AddSubjectTypeDlg\", obj_id:86 });'>Research Sample</a>"

},

{

"id": 2021,

"obj_id": 2021,

"display_name": "CS_freq_refallele",

"name": "CS_freq_refallele",

"type": "Number",

"values": "",

"created_at": "11/22/2013",

"updated_at": "11/22/2013",

"created_by": [redacted],

"searchable_quick": false,

"searchable_advanced": null,

"searchable_batch": null,

"searchable": "",

"pii": "No",

"qtip": "",

"used_by": "Exome Analysis Results",

"used_by_display": "<a href='#' onClick='remoteJS({name:\"dialogs/AddSubjectTypeDlg\", obj_id:156 });'>Exome Analysis Results</a>"

},

{

"id": 2022,

"obj_id": 2022,

"display_name": "CS_freq_varallele",

"name": "CS_freq_varallele",

"type": "Number",

"values": "",

"created_at": "11/22/2013",

"updated_at": "11/22/2013",

"created_by": [redacted],

"searchable_quick": false,

"searchable_advanced": null,

"searchable_batch": null,

"searchable": "",

"pii": "No",

"qtip": "",

"used_by": "Exome Analysis Results",

"used_by_display": "<a href='#' onClick='remoteJS({name:\"dialogs/AddSubjectTypeDlg\", obj_id:156 });'>Exome Analysis Results</a>"

},

{

"id": 1275,

"obj_id": 1275,

"display_name": "CSF Samples",

"name": "CSF Samples",

"type": "Subject Back Ref",

"values": "",

"created_at": "06/03/2013",

"updated_at": "10/29/2014",

"created_by": [redacted],

"searchable_quick": false,

"searchable_advanced": true,

"searchable_batch": false,

"searchable": "Advanced",

"pii": "No",

"qtip": "",

"used_by": "Patient, Research Sample, Patient Follow Up Visit",

"used_by_display": "<a href='#' onClick='remoteJS({name:\"dialogs/AddSubjectTypeDlg\", obj_id:1 });'>Patient</a>, <a href='#' onClick='remoteJS({name:\"dialogs/AddSubjectTypeDlg\", obj_id:86 });'>Research Sample</a>, <a href='#' onClick='remoteJS({name:\"dialogs/AddSubjectTypeDlg\", obj_id:264 });'>Patient Follow Up Visit</a>"

},

{

"id": 1005,

"obj_id": 1005,

"display_name": "CSF Spun Down",

"name": "CSF Spun Down",

"type": "CheckBox",

"values": "",

"created_at": "05/13/2013",

"updated_at": "09/19/2013",

"created_by": [redacted],

"searchable_quick": false,

"searchable_advanced": null,

"searchable_batch": null,

"searchable": "",

"pii": "No",

"qtip": "High for 3 minutes",

"used_by": "CSF Processing",

"used_by_display": "<a href='#' onClick='remoteJS({name:\"dialogs/AddSubjectTypeDlg\", obj_id:117 });'>CSF Processing</a>"

},

{

"id": 926,

"obj_id": 926,

"display_name": "CT_DA_Date",

"name": "CT_DA_Date",

"type": "Text Field",

"values": "",

"created_at": "05/10/2013",

"updated_at": "09/19/2013",

"created_by": [redacted],

"searchable_quick": false,

"searchable_advanced": null,

"searchable_batch": null,

"searchable": "",

"pii": "No",

"qtip": "",

"used_by": "Sharepoint Legacy - Pediatric Patient",

"used_by_display": "<a href='#' onClick='remoteJS({name:\"dialogs/AddSubjectTypeDlg\", obj_id:114 });'>Sharepoint Legacy - Pediatric Patient</a>"

},

{

"id": 2750,

"obj_id": 2750,

"display_name": "Culled Variant",

"name": "Culled Variant",

"type": "Choice",

"values": "Yes, No",

"created_at": "07/24/2014",

"updated_at": "07/24/2014",

"created_by": [redacted],

"searchable_quick": false,

"searchable_advanced": false,

"searchable_batch": false,

"searchable": "",

"pii": "No",

"qtip": "",

"used_by": "Exome Analysis Results",

"used_by_display": "<a href='#' onClick='remoteJS({name:\"dialogs/AddSubjectTypeDlg\", obj_id:156 });'>Exome Analysis Results</a>"

},

{

"id": 2484,

"obj_id": 2484,

"display_name": "Cultured or Frozen Fibroblast",

"name": "Cultured or Frozen Fibroblast",

"type": "Choice",

"values": "Cultured, Frozen",

"created_at": "04/02/2014",

"updated_at": "04/02/2014",

"created_by": [redacted],

"searchable_quick": false,

"searchable_advanced": null,

"searchable_batch": null,

"searchable": "",

"pii": "No",

"qtip": "Does the collaborator request a freshly grown culture or a frozen culture",

"used_by": "Collaborations",

"used_by_display": "<a href='#' onClick='remoteJS({name:\"dialogs/AddSubjectTypeDlg\", obj_id:147 });'>Collaborations</a>"

},

{

"id": 463,

"obj_id": 463,

"display_name": "Culture Fibroblast",

"name": "Culture Fibroblast",

"type": "Subject",

"values": "",

"created_at": "04/28/2013",

"updated_at": "09/19/2013",

"created_by": [redacted],

"searchable_quick": false,

"searchable_advanced": null,

"searchable_batch": null,

"searchable": "",

"pii": "No",

"qtip": "",

"used_by": "iPS Cells, Collaborations, Glycomics Collaborations",

"used_by_display": "<a href='#' onClick='remoteJS({name:\"dialogs/AddSubjectTypeDlg\", obj_id:41 });'>iPS Cells</a>, <a href='#' onClick='remoteJS({name:\"dialogs/AddSubjectTypeDlg\", obj_id:147 });'>Collaborations</a>, <a href='#' onClick='remoteJS({name:\"dialogs/AddSubjectTypeDlg\", obj_id:244 });'>Glycomics Collaborations</a>"

},

{

"id": 466,

"obj_id": 466,

"display_name": "Culture Fibroblasts",

"name": "Culture Fibroblasts",

"type": "Subject Back Ref",

"values": "",

"created_at": "04/30/2013",

"updated_at": "10/29/2014",

"created_by": [redacted],

"searchable_quick": false,

"searchable_advanced": true,

"searchable_batch": false,

"searchable": "Advanced",

"pii": "No",

"qtip": "",

"used_by": "Research Sample",

"used_by_display": "<a href='#' onClick='remoteJS({name:\"dialogs/AddSubjectTypeDlg\", obj_id:86 });'>Research Sample</a>"

},

{

"id": 2936,

"obj_id": 2936,

"display_name": "Culture Media",

"name": "Culture Media",

"type": "Text Field",

"values": "",

"created_at": "12/08/2014",

"updated_at": "12/08/2014",

"created_by": [redacted],

"searchable_quick": false,

"searchable_advanced": true,

"searchable_batch": false,

"searchable": "Advanced",

"pii": "No",

"qtip": "",

"used_by": "Rescue Cell Culture",

"used_by_display": "<a href='#' onClick='remoteJS({name:\"dialogs/AddSubjectTypeDlg\", obj_id:247 });'>Rescue Cell Culture</a>"

},

{

"id": 2560,

"obj_id": 2560,

"display_name": "Culture Type",

"name": "Culture Type",

"type": "Choice",

"values": "Fibroblast, Osteoblast, HELA cells, HEK293 cells, CHO, MEF, SH-SY5Y, iPS cells, Melanocyte, Lymphoblast, NTERA2, JURKAT",

"created_at": "04/24/2014",

"updated_at": "04/24/2014",

"created_by": [redacted],

"searchable_quick": false,

"searchable_advanced": null,

"searchable_batch": null,

"searchable": "",

"pii": "No",

"qtip": "",

"used_by": "",

"used_by_display": ""

},

{

"id": 2811,

"obj_id": 2811,

"display_name": "Curator Notes",

"name": "Curator Notes",

"type": "Text Area",

"values": "",

"created_at": "08/28/2014",

"updated_at": "12/09/2014",

"created_by": [redacted],

"searchable_quick": false,

"searchable_advanced": false,

"searchable_batch": false,

"searchable": "",

"pii": "No",

"qtip": "Curator notes from Phenote",

"used_by": "Zebrafish Phenotype, Drosophila Phenotypes",

"used_by_display": "<a href='#' onClick='remoteJS({name:\"dialogs/AddSubjectTypeDlg\", obj_id:173 });'>Zebrafish Phenotype</a>, <a href='#' onClick='remoteJS({name:\"dialogs/AddSubjectTypeDlg\", obj_id:265 });'>Drosophila Phenotypes</a>"

},

{

"id": 533,

"obj_id": 533,

"display_name": "Current",

"name": "Current",

"type": "Text Area",

"values": "",

"created_at": "05/07/2013",

"updated_at": "09/19/2013",

"created_by": [redacted],

"searchable_quick": false,

"searchable_advanced": null,

"searchable_batch": null,

"searchable": "",

"pii": "No",

"qtip": "",

"used_by": "Labmatrix Legacy - Phenotype Legacy Files",

"used_by_display": "<a href='#' onClick='remoteJS({name:\"dialogs/AddSubjectTypeDlg\", obj_id:96 });'>Labmatrix Legacy - Phenotype Legacy Files</a>"

},

{

"id": 534,

"obj_id": 534,

"display_name": "Current Mass",

"name": "Current Mass",

"type": "Text Area",

"values": "",

"created_at": "05/07/2013",

"updated_at": "09/19/2013",

"created_by": [redacted],

"searchable_quick": false,

"searchable_advanced": null,

"searchable_batch": null,

"searchable": "",

"pii": "No",

"qtip": "",

"used_by": "Labmatrix Legacy - Biomaterials",

"used_by_display": "<a href='#' onClick='remoteJS({name:\"dialogs/AddSubjectTypeDlg\", obj_id:91 });'>Labmatrix Legacy - Biomaterials</a>"

},

{

"id": 535,

"obj_id": 535,

"display_name": "Current Mass Units",

"name": "Current Mass Units",

"type": "Text Area",

"values": "",

"created_at": "05/07/2013",

"updated_at": "09/19/2013",

"created_by": [redacted],

"searchable_quick": false,

"searchable_advanced": null,

"searchable_batch": null,

"searchable": "",

"pii": "No",

"qtip": "",

"used_by": "Labmatrix Legacy - Biomaterials",

"used_by_display": "<a href='#' onClick='remoteJS({name:\"dialogs/AddSubjectTypeDlg\", obj_id:91 });'>Labmatrix Legacy - Biomaterials</a>"

},

{

"id": 2324,

"obj_id": 2324,

"display_name": "Current Medications",

"name": "Current Medications",

"type": "Text Area",

"values": "",

"created_at": "03/04/2014",

"updated_at": "03/04/2014",

"created_by": [redacted],

"searchable_quick": false,

"searchable_advanced": null,

"searchable_batch": null,

"searchable": "",

"pii": "Yes",

"qtip": "",

"used_by": "Patient Review",

"used_by_display": "<a href='#' onClick='remoteJS({name:\"dialogs/AddSubjectTypeDlg\", obj_id:83 });'>Patient Review</a>"

},

{

"id": 2660,

"obj_id": 2660,

"display_name": "Current password",

"name": "Current password",

"type": "Text Field",

"values": "",

"created_at": "06/03/2014",

"updated_at": "06/03/2014",

"created_by": [redacted],

"searchable_quick": false,

"searchable_advanced": null,

"searchable_batch": null,

"searchable": "",

"pii": "No",

"qtip": "",

"used_by": "Bioinformatics Hard Drives",

"used_by_display": "<a href='#' onClick='remoteJS({name:\"dialogs/AddSubjectTypeDlg\", obj_id:248 });'>Bioinformatics Hard Drives</a>"

},

{

"id": 330,

"obj_id": 330,

"display_name": "Current Review Status",

"name": "Current Review Status",

"type": "Choice",

"values": "Not Reviewed, Needs Re-Review, Enqueued Studies Pending, Hold for other Reason (see notes), Closed with Diagnosis (see notes), Closed, UDP Laboratory Follow-Up, Collaborated Out, Genetic Analysis Complete, No Plan, Interim Analysis Needed to Proceed, Samples sent for analysis, awaiting data, Genetic Studies Done, Analysis Pending, All requested samples added to current list, Returned from Queue",

"created_at": "04/08/2013",

"updated_at": "08/19/2013",

"created_by": [redacted],

"searchable_quick": false,

"searchable_advanced": null,

"searchable_batch": null,

"searchable": "",

"pii": "No",

"qtip": "",

"used_by": "Friday Meeting Notes, Sharepoint Legacy - Friday Meeting Notes",

"used_by_display": "<a href='#' onClick='remoteJS({name:\"dialogs/AddSubjectTypeDlg\", obj_id:82 });'>Friday Meeting Notes</a>, <a href='#' onClick='remoteJS({name:\"dialogs/AddSubjectTypeDlg\", obj_id:154 });'>Sharepoint Legacy - Friday Meeting Notes</a>"

},

{

"id": 536,

"obj_id": 536,

"display_name": "Current Status",

"name": "Current Status",

"type": "Text Field",

"values": "",

"created_at": "05/07/2013",

"updated_at": "09/19/2013",

"created_by": [redacted],

"searchable_quick": false,

"searchable_advanced": null,

"searchable_batch": null,

"searchable": "",

"pii": "No",

"qtip": "",

"used_by": "Labmatrix Legacy - Biomaterials",

"used_by_display": "<a href='#' onClick='remoteJS({name:\"dialogs/AddSubjectTypeDlg\", obj_id:91 });'>Labmatrix Legacy - Biomaterials</a>"

},

{

"id": 537,

"obj_id": 537,

"display_name": "Current Status Date",

"name": "Current Status Date",

"type": "Date",

"values": "",

"created_at": "05/07/2013",

"updated_at": "06/10/2014",

"created_by": [redacted],

"searchable_quick": false,

"searchable_advanced": null,

"searchable_batch": null,

"searchable": "",

"pii": "Yes",

"qtip": "",

"used_by": "Labmatrix Legacy - Biomaterials",

"used_by_display": "<a href='#' onClick='remoteJS({name:\"dialogs/AddSubjectTypeDlg\", obj_id:91 });'>Labmatrix Legacy - Biomaterials</a>"

},

{

"id": 538,

"obj_id": 538,

"display_name": "Current Volume",

"name": "Current Volume",

"type": "Text Area",

"values": "",

"created_at": "05/07/2013",

"updated_at": "09/19/2013",

"created_by": [redacted],

"searchable_quick": false,

"searchable_advanced": null,

"searchable_batch": null,

"searchable": "",

"pii": "No",

"qtip": "",

"used_by": "Labmatrix Legacy - Biomaterials",

"used_by_display": "<a href='#' onClick='remoteJS({name:\"dialogs/AddSubjectTypeDlg\", obj_id:91 });'>Labmatrix Legacy - Biomaterials</a>"

},

{

"id": 860,

"obj_id": 860,

"display_name": "Current Volume Units",

"name": "Current Volume Units",

"type": "Text Field",

"values": "",

"created_at": "05/08/2013",

"updated_at": "09/19/2013",

"created_by": [redacted],

"searchable_quick": false,

"searchable_advanced": null,

"searchable_batch": null,

"searchable": "",

"pii": "No",

"qtip": "",

"used_by": "Labmatrix Legacy - Biomaterials",

"used_by_display": "<a href='#' onClick='remoteJS({name:\"dialogs/AddSubjectTypeDlg\", obj_id:91 });'>Labmatrix Legacy - Biomaterials</a>"

},

{

"id": 539,

"obj_id": 539,

"display_name": "Custom ID",

"name": "Custom ID",

"type": "Text Area",

"values": "",

"created_at": "05/07/2013",

"updated_at": "09/19/2013",

"created_by": [redacted],

"searchable_quick": false,

"searchable_advanced": null,

"searchable_batch": null,

"searchable": "",

"pii": "No",

"qtip": "",

"used_by": "Labmatrix Legacy - Subjects",

"used_by_display": "<a href='#' onClick='remoteJS({name:\"dialogs/AddSubjectTypeDlg\", obj_id:112 });'>Labmatrix Legacy - Subjects</a>"

},

{

"id": 927,

"obj_id": 927,

"display_name": "DA_CT_Dispo",

"name": "DA_CT_Dispo",

"type": "Text Field",

"values": "",

"created_at": "05/10/2013",

"updated_at": "09/19/2013",

"created_by": [redacted],

"searchable_quick": false,

"searchable_advanced": null,

"searchable_batch": null,

"searchable": "",

"pii": "No",

"qtip": "",

"used_by": "Sharepoint Legacy - Pediatric Patient",

"used_by_display": "<a href='#' onClick='remoteJS({name:\"dialogs/AddSubjectTypeDlg\", obj_id:114 });'>Sharepoint Legacy - Pediatric Patient</a>"

},

{

"id": 1241,

"obj_id": 1241,

"display_name": "Database and schedule to",

"name": "Database and schedule to",

"type": "List",

"values": "Floor nurse manager, [redacted] if Peds <10kg, OR Team if using OR, Anesthesia Team if using Anesthesia, NP/Attending, [redacted] if having MRS",

"created_at": "06/03/2013",

"updated_at": "09/19/2013",

"created_by": [redacted],

"searchable_quick": false,

"searchable_advanced": null,

"searchable_batch": null,

"searchable": "",

"pii": "No",

"qtip": "",

"used_by": "Patient Visit Tools, Patient Visit Planning for Version C",

"used_by_display": "<a href='#' onClick='remoteJS({name:\"dialogs/AddSubjectTypeDlg\", obj_id:163 });'>Patient Visit Tools</a>, <a href='#' onClick='remoteJS({name:\"dialogs/AddSubjectTypeDlg\", obj_id:192 });'>Patient Visit Planning for Version C</a>"

},

{

"id": 2576,

"obj_id": 2576,

"display_name": "Database submission of genetic data",

"name": "Database submission of genetic data",

"type": "Choice",

"values": "Yes, No",

"created_at": "05/01/2014",

"updated_at": "05/01/2014",

"created_by": [redacted],

"searchable_quick": false,

"searchable_advanced": null,

"searchable_batch": null,

"searchable": "",

"pii": "No",

"qtip": "",

"used_by": "Consent Versions",

"used_by_display": "<a href='#' onClick='remoteJS({name:\"dialogs/AddSubjectTypeDlg\", obj_id:239 });'>Consent Versions</a>"

},

{

"id": 2926,

"obj_id": 2926,

"display_name": "Data Explanation File",

"name": "Data Explanation File",

"type": "File",

"values": "",

"created_at": "12/08/2014",

"updated_at": "12/08/2014",

"created_by": [redacted],

"searchable_quick": false,

"searchable_advanced": true,

"searchable_batch": false,

"searchable": "Advanced",

"pii": "No",

"qtip": "",

"used_by": "ChIP-Seq",

"used_by_display": "<a href='#' onClick='remoteJS({name:\"dialogs/AddSubjectTypeDlg\", obj_id:254 });'>ChIP-Seq</a>"

},

{

"id": 1747,

"obj_id": 1747,

"display_name": "Data Sent to Appistry",

"name": "Data Sent to Appistry",

"type": "CheckBox",

"values": "",

"created_at": "10/18/2013",

"updated_at": "10/18/2013",

"created_by": [redacted],

"searchable_quick": true,

"searchable_advanced": true,

"searchable_batch": true,

"searchable": "Quick,Advanced,Batch",

"pii": "No",

"qtip": "",

"used_by": "",

"used_by_display": ""

},

{

"id": 1746,

"obj_id": 1746,

"display_name": "Data Sent to Toronto",

"name": "Data Sent to Toronto",

"type": "CheckBox",

"values": "",

"created_at": "10/18/2013",

"updated_at": "10/18/2013",

"created_by": [redacted],

"searchable_quick": true,

"searchable_advanced": true,

"searchable_batch": true,

"searchable": "Quick,Advanced,Batch",

"pii": "No",

"qtip": "",

"used_by": "",

"used_by_display": ""

},

{

"id": 1748,

"obj_id": 1748,

"display_name": "Data Submitted to dbGaP",

"name": "Data Submitted to dbGaP",

"type": "CheckBox",

"values": "",

"created_at": "10/18/2013",

"updated_at": "03/04/2014",

"created_by": [redacted],

"searchable_quick": true,

"searchable_advanced": true,

"searchable_batch": true,

"searchable": "Quick,Advanced,Batch",

"pii": "No",

"qtip": "",

"used_by": "",

"used_by_display": ""

},

{

"id": 1263,

"obj_id": 1263,

"display_name": "Data to be Returned to",

"name": "Data to be Returned to",

"type": "Choice",

"values": [redacted],

"created_at": "06/03/2013",

"updated_at": "09/19/2013",

"created_by": [redacted],

"searchable_quick": false,

"searchable_advanced": null,

"searchable_batch": null,

"searchable": "",

"pii": "No",

"qtip": "",

"used_by": "NIH Visit",

"used_by_display": "<a href='#' onClick='remoteJS({name:\"dialogs/AddSubjectTypeDlg\", obj_id:140 });'>NIH Visit</a>"

},

{

"id": 540,

"obj_id": 540,

"display_name": "Date",

"name": "Date",

"type": "Date",

"values": "",

"created_at": "05/07/2013",

"updated_at": "07/19/2013",

"created_by": [redacted],

"searchable_quick": false,

"searchable_advanced": null,

"searchable_batch": null,

"searchable": "",

"pii": "Yes",

"qtip": "",

"used_by": "Labmatrix Legacy - Test Order Records, Patient Specific Gene List",

"used_by_display": "<a href='#' onClick='remoteJS({name:\"dialogs/AddSubjectTypeDlg\", obj_id:106 });'>Labmatrix Legacy - Test Order Records</a>, <a href='#' onClick='remoteJS({name:\"dialogs/AddSubjectTypeDlg\", obj_id:256 });'>Patient Specific Gene List</a>"

},

{

"id": 941,

"obj_id": 941,

"display_name": "Date 1st Review rec'd",

"name": "Date 1st Review rec'd",

"type": "Date",

"values": "",

"created_at": "05/10/2013",

"updated_at": "07/19/2013",

"created_by": [redacted],

"searchable_quick": false,

"searchable_advanced": null,

"searchable_batch": null,

"searchable": "",

"pii": "Yes",

"qtip": "",

"used_by": "Sharepoint Legacy - Adult Patient Status",

"used_by_display": "<a href='#' onClick='remoteJS({name:\"dialogs/AddSubjectTypeDlg\", obj_id:115 });'>Sharepoint Legacy - Adult Patient Status</a>"

},

{

"id": 942,

"obj_id": 942,

"display_name": "Date 2nd Acc/Rej Review",

"name": "Date 2nd Acc/Rej Review",

"type": "Date",

"values": "",

"created_at": "05/10/2013",

"updated_at": "07/19/2013",

"created_by": [redacted],

"searchable_quick": false,

"searchable_advanced": null,

"searchable_batch": null,

"searchable": "",

"pii": "Yes",

"qtip": "",

"used_by": "Sharepoint Legacy - Adult Patient Status",

"used_by_display": "<a href='#' onClick='remoteJS({name:\"dialogs/AddSubjectTypeDlg\", obj_id:115 });'>Sharepoint Legacy - Adult Patient Status</a>"

},

{

"id": 944,

"obj_id": 944,

"display_name": "Date Accept",

"name": "Date Accept",

"type": "Date",

"values": "",

"created_at": "05/10/2013",

"updated_at": "07/19/2013",

"created_by": [redacted],

"searchable_quick": false,

"searchable_advanced": null,

"searchable_batch": null,

"searchable": "",

"pii": "Yes",

"qtip": "",

"used_by": "Sharepoint Legacy - Adult Patient Status",

"used_by_display": "<a href='#' onClick='remoteJS({name:\"dialogs/AddSubjectTypeDlg\", obj_id:115 });'>Sharepoint Legacy - Adult Patient Status</a>"

},

{

"id": 1126,

"obj_id": 1126,

"display_name": "Date Acceptance Letter Mailed",

"name": "Date Acceptance Letter Mailed",

"type": "Date",

"values": "",

"created_at": "05/28/2013",

"updated_at": "07/19/2013",

"created_by": [redacted],

"searchable_quick": false,

"searchable_advanced": null,

"searchable_batch": null,

"searchable": "",

"pii": "Yes",

"qtip": "",

"used_by": "Patient Visit Tools, Patient Visit Planning for Version C",

"used_by_display": "<a href='#' onClick='remoteJS({name:\"dialogs/AddSubjectTypeDlg\", obj_id:163 });'>Patient Visit Tools</a>, <a href='#' onClick='remoteJS({name:\"dialogs/AddSubjectTypeDlg\", obj_id:192 });'>Patient Visit Planning for Version C</a>"

},

{

"id": 943,

"obj_id": 943,

"display_name": "Date Acc/Rej Review",

"name": "Date Acc/Rej Review",

"type": "Date",

"values": "",

"created_at": "05/10/2013",

"updated_at": "07/19/2013",

"created_by": [redacted],

"searchable_quick": false,

"searchable_advanced": null,

"searchable_batch": null,

"searchable": "",

"pii": "Yes",

"qtip": "",

"used_by": "Sharepoint Legacy - Adult Patient Status",

"used_by_display": "<a href='#' onClick='remoteJS({name:\"dialogs/AddSubjectTypeDlg\", obj_id:115 });'>Sharepoint Legacy - Adult Patient Status</a>"

},

{

"id": 1560,

"obj_id": 1560,

"display_name": "date_added",

"name": "date_added",

"type": "Date",

"values": "",

"created_at": "08/09/2013",

"updated_at": "08/09/2013",

"created_by": [redacted],

"searchable_quick": false,

"searchable_advanced": null,

"searchable_batch": null,

"searchable": "",

"pii": "No",

"qtip": "",

"used_by": "Labmatrix Legacy - Todo Form",

"used_by_display": "<a href='#' onClick='remoteJS({name:\"dialogs/AddSubjectTypeDlg\", obj_id:107 });'>Labmatrix Legacy - Todo Form</a>"

},

{

"id": 1557,

"obj_id": 1557,

"display_name": "Date Administered",

"name": "Date Administered",

"type": "Date",

"values": "",

"created_at": "08/09/2013",

"updated_at": "08/09/2013",

"created_by": [redacted],

"searchable_quick": false,

"searchable_advanced": null,

"searchable_batch": null,

"searchable": "",

"pii": "No",

"qtip": "",

"used_by": "",

"used_by_display": ""

},

{

"id": 945,

"obj_id": 945,

"display_name": "Date Admission",

"name": "Date Admission",

"type": "Date",

"values": "",

"created_at": "05/10/2013",

"updated_at": "07/19/2013",

"created_by": [redacted],

"searchable_quick": false,

"searchable_advanced": null,

"searchable_batch": null,

"searchable": "",

"pii": "Yes",

"qtip": "",

"used_by": "Sharepoint Legacy - Adult Patient Status, Patient Visit Tools, Patient Visit Planning for Version C",

"used_by_display": "<a href='#' onClick='remoteJS({name:\"dialogs/AddSubjectTypeDlg\", obj_id:115 });'>Sharepoint Legacy - Adult Patient Status</a>, <a href='#' onClick='remoteJS({name:\"dialogs/AddSubjectTypeDlg\", obj_id:163 });'>Patient Visit Tools</a>, <a href='#' onClick='remoteJS({name:\"dialogs/AddSubjectTypeDlg\", obj_id:192 });'>Patient Visit Planning for Version C</a>"

},

{

"id": 2128,

"obj_id": 2128,

"display_name": "Date Agreement Executed",

"name": "Date Agreement Executed",

"type": "Date",

"values": "",

"created_at": "01/03/2014",

"updated_at": "01/03/2014",

"created_by": [redacted],

"searchable_quick": false,

"searchable_advanced": null,

"searchable_batch": null,

"searchable": "",

"pii": "No",

"qtip": "",

"used_by": "Collaboration Projects",

"used_by_display": "<a href='#' onClick='remoteJS({name:\"dialogs/AddSubjectTypeDlg\", obj_id:204 });'>Collaboration Projects</a>"

},

{

"id": 1901,

"obj_id": 1901,

"display_name": "Date Alignment Complete",

"name": "Date Alignment Complete",

"type": "Date",

"values": "",

"created_at": "11/08/2013",

"updated_at": "11/08/2013",

"created_by": [redacted],

"searchable_quick": false,

"searchable_advanced": null,

"searchable_batch": null,

"searchable": "",

"pii": "No",

"qtip": "",

"used_by": "Diploid Alignment",

"used_by_display": "<a href='#' onClick='remoteJS({name:\"dialogs/AddSubjectTypeDlg\", obj_id:209 });'>Diploid Alignment</a>"

},

{

"id": 353,

"obj_id": 353,

"display_name": "Date Alignment Started",

"name": "Date Alignment Started",

"type": "Date",

"values": "",

"created_at": "04/12/2013",

"updated_at": "11/08/2013",

"created_by": [redacted],

"searchable_quick": false,

"searchable_advanced": null,

"searchable_batch": null,

"searchable": "",

"pii": "No",

"qtip": "",

"used_by": "Diploid Alignment",

"used_by_display": "<a href='#' onClick='remoteJS({name:\"dialogs/AddSubjectTypeDlg\", obj_id:209 });'>Diploid Alignment</a>"

},

{

"id": 2654,

"obj_id": 2654,

"display_name": "Date All File Transfers Complete for Collaboration",

"name": "Date All File Transfers Complete for Collaboration",

"type": "Date",

"values": "",

"created_at": "05/29/2014",

"updated_at": "05/29/2014",

"created_by": [redacted],

"searchable_quick": false,

"searchable_advanced": null,

"searchable_batch": null,

"searchable": "",

"pii": "No",

"qtip": "",

"used_by": "Information Technology Collaboration",

"used_by_display": "<a href='#' onClick='remoteJS({name:\"dialogs/AddSubjectTypeDlg\", obj_id:183 });'>Information Technology Collaboration</a>"

},

{

"id": 898,

"obj_id": 898,

"display_name": "Date Amplicon Sent to Macrogen",

"name": "Date Amplicon Sent to Macrogen",

"type": "Date",

"values": "",

"created_at": "05/10/2013",

"updated_at": "02/11/2014",

"created_by": [redacted],

"searchable_quick": false,

"searchable_advanced": null,

"searchable_batch": null,

"searchable": "",

"pii": "No",

"qtip": "",

"used_by": "Sanger Interpretation",

"used_by_display": "<a href='#' onClick='remoteJS({name:\"dialogs/AddSubjectTypeDlg\", obj_id:22 });'>Sanger Interpretation</a>"

},

{

"id": 2048,

"obj_id": 2048,

"display_name": "Date Annotation Complete",

"name": "Date Annotation Complete",

"type": "Date",

"values": "",

"created_at": "12/04/2013",

"updated_at": "12/04/2013",

"created_by": [redacted],

"searchable_quick": false,

"searchable_advanced": null,

"searchable_batch": null,

"searchable": "",

"pii": "No",

"qtip": "",

"used_by": "Cohort Variant Call Format File",

"used_by_display": "<a href='#' onClick='remoteJS({name:\"dialogs/AddSubjectTypeDlg\", obj_id:195 });'>Cohort Variant Call Format File</a>"

},

{

"id": 1001,

"obj_id": 1001,

"display_name": "Date Annotation Pipeline Complete",

"name": "Date Annotation Pipeline Complete",

"type": "Date",

"values": "",

"created_at": "05/10/2013",

"updated_at": "09/19/2013",

"created_by": [redacted],

"searchable_quick": false,

"searchable_advanced": null,

"searchable_batch": null,

"searchable": "",

"pii": "No",

"qtip": "",

"used_by": "",

"used_by_display": ""

},

{

"id": 946,

"obj_id": 946,

"display_name": "Date Applic",

"name": "Date Applic",

"type": "Date",

"values": "",

"created_at": "05/10/2013",

"updated_at": "06/10/2014",

"created_by": [redacted],

"searchable_quick": false,

"searchable_advanced": null,

"searchable_batch": null,

"searchable": "",

"pii": "Yes",

"qtip": "",

"used_by": "Sharepoint Legacy - Adult Patient Status",

"used_by_display": "<a href='#' onClick='remoteJS({name:\"dialogs/AddSubjectTypeDlg\", obj_id:115 });'>Sharepoint Legacy - Adult Patient Status</a>"

},

{

"id": 2504,

"obj_id": 2504,

"display_name": "Date BAM Files Sent",

"name": "Date BAM Files Sent",

"type": "Date",

"values": "",

"created_at": "04/09/2014",

"updated_at": "04/09/2014",

"created_by": [redacted],

"searchable_quick": false,

"searchable_advanced": null,

"searchable_batch": null,

"searchable": "",

"pii": "No",

"qtip": "Date sent to Toronto for Iteration 0 VCF generation",

"used_by": "PhenomeCentral Data Submission",

"used_by_display": "<a href='#' onClick='remoteJS({name:\"dialogs/AddSubjectTypeDlg\", obj_id:216 });'>PhenomeCentral Data Submission</a>"

},

{

"id": 1892,

"obj_id": 1892,

"display_name": "Date Calling Complete",

"name": "Date Calling Complete",

"type": "Date",

"values": "",

"created_at": "11/08/2013",

"updated_at": "11/08/2013",

"created_by": [redacted],

"searchable_quick": false,

"searchable_advanced": null,

"searchable_batch": null,

"searchable": "",

"pii": "No",

"qtip": "",

"used_by": "",

"used_by_display": ""

},

{

"id": 1165,

"obj_id": 1165,

"display_name": "Date cDNA Ordered",

"name": "Date cDNA Ordered",

"type": "Date",

"values": "",

"created_at": "05/29/2013",

"updated_at": "09/19/2013",

"created_by": [redacted],

"searchable_quick": false,

"searchable_advanced": null,

"searchable_batch": null,

"searchable": "",

"pii": "No",

"qtip": "",

"used_by": "Entry Vector, Clone",

"used_by_display": "<a href='#' onClick='remoteJS({name:\"dialogs/AddSubjectTypeDlg\", obj_id:193 });'>Entry Vector</a>, <a href='#' onClick='remoteJS({name:\"dialogs/AddSubjectTypeDlg\", obj_id:266 });'>Clone</a>"

},

{

"id": 2829,

"obj_id": 2829,

"display_name": "Date Chart created:",

"name": "Date Chart created:",

"type": "Date",

"values": "",

"created_at": "09/12/2014",

"updated_at": "09/12/2014",

"created_by": [redacted],

"searchable_quick": false,

"searchable_advanced": false,

"searchable_batch": false,

"searchable": "",

"pii": "Yes",

"qtip": "",

"used_by": "Clinical Notes",

"used_by_display": "<a href='#' onClick='remoteJS({name:\"dialogs/AddSubjectTypeDlg\", obj_id:267 });'>Clinical Notes</a>"

},

{

"id": 129,

"obj_id": 129,

"display_name": "Date CLIA Results Received",

"name": "Date CLIA Results Received",

"type": "Date",

"values": "",

"created_at": "02/13/2013",

"updated_at": "09/19/2013",

"created_by": [redacted],

"searchable_quick": false,

"searchable_advanced": null,

"searchable_batch": null,

"searchable": "",

"pii": "No",

"qtip": "",

"used_by": "CLIA Validation",

"used_by_display": "<a href='#' onClick='remoteJS({name:\"dialogs/AddSubjectTypeDlg\", obj_id:15 });'>CLIA Validation</a>"

},

{

"id": 404,

"obj_id": 404,

"display_name": "Date CNV Analysis Complete",

"name": "Date CNV Analysis Complete",

"type": "Date",

"values": "",

"created_at": "04/15/2013",

"updated_at": "09/19/2013",

"created_by": [redacted],

"searchable_quick": false,

"searchable_advanced": null,

"searchable_batch": null,

"searchable": "",

"pii": "No",

"qtip": "",

"used_by": "SNP Analysis",

"used_by_display": "<a href='#' onClick='remoteJS({name:\"dialogs/AddSubjectTypeDlg\", obj_id:59 });'>SNP Analysis</a>"

},

{

"id": 541,

"obj_id": 541,

"display_name": "Date Collected",

"name": "Date Collected",

"type": "Date",

"values": "",

"created_at": "05/07/2013",

"updated_at": "10/28/2014",

"created_by": [redacted],

"searchable_quick": false,

"searchable_advanced": true,

"searchable_batch": false,

"searchable": "Advanced",

"pii": "No",

"qtip": "",

"used_by": "Labmatrix Legacy - Example Record, Labmatrix Legacy - Adult UDP Entry, Labmatrix Legacy - Test Order Records, Labmatrix Legacy - Todo Form, Glycomics Results, Labmatrix Legacy - Send Out Tests",

"used_by_display": "<a href='#' onClick='remoteJS({name:\"dialogs/AddSubjectTypeDlg\", obj_id:102 });'>Labmatrix Legacy - Example Record</a>, <a href='#' onClick='remoteJS({name:\"dialogs/AddSubjectTypeDlg\", obj_id:101 });'>Labmatrix Legacy - Adult UDP Entry</a>, <a href='#' onClick='remoteJS({name:\"dialogs/AddSubjectTypeDlg\", obj_id:106 });'>Labmatrix Legacy - Test Order Records</a>, <a href='#' onClick='remoteJS({name:\"dialogs/AddSubjectTypeDlg\", obj_id:107 });'>Labmatrix Legacy - Todo Form</a>, <a href='#' onClick='remoteJS({name:\"dialogs/AddSubjectTypeDlg\", obj_id:176 });'>Glycomics Results</a>, <a href='#' onClick='remoteJS({name:\"dialogs/AddSubjectTypeDlg\", obj_id:104 });'>Labmatrix Legacy - Send Out Tests</a>"

},

{

"id": 2459,

"obj_id": 2459,

"display_name": "Date Completed",

"name": "Date Completed",

"type": "Date",

"values": "",

"created_at": "03/26/2014",

"updated_at": "03/28/2014",

"created_by": [redacted],

"searchable_quick": false,

"searchable_advanced": null,

"searchable_batch": null,

"searchable": "",

"pii": "No",

"qtip": "",

"used_by": "Assay, Drosophila Reports, Invoice Information",

"used_by_display": "<a href='#' onClick='remoteJS({name:\"dialogs/AddSubjectTypeDlg\", obj_id:225 });'>Assay</a>, <a href='#' onClick='remoteJS({name:\"dialogs/AddSubjectTypeDlg\", obj_id:237 });'>Drosophila Reports</a>, <a href='#' onClick='remoteJS({name:\"dialogs/AddSubjectTypeDlg\", obj_id:270 });'>Invoice Information</a>"

},

{

"id": 182,

"obj_id": 182,

"display_name": "Date Consent Signed",

"name": "Date Consent Signed",

"type": "Date",

"values": "",

"created_at": "02/26/2013",

"updated_at": "07/19/2013",

"created_by": [redacted],

"searchable_quick": false,

"searchable_advanced": null,

"searchable_batch": null,

"searchable": "",

"pii": "Yes",

"qtip": "",

"used_by": "Blood Kit, Consent Signed, Patient, Patient Follow Up Visit",

"used_by_display": "<a href='#' onClick='remoteJS({name:\"dialogs/AddSubjectTypeDlg\", obj_id:33 });'>Blood Kit</a>, <a href='#' onClick='remoteJS({name:\"dialogs/AddSubjectTypeDlg\", obj_id:141 });'>Consent Signed</a>, <a href='#' onClick='remoteJS({name:\"dialogs/AddSubjectTypeDlg\", obj_id:1 });'>Patient</a>, <a href='#' onClick='remoteJS({name:\"dialogs/AddSubjectTypeDlg\", obj_id:264 });'>Patient Follow Up Visit</a>"

},

{

"id": 947,

"obj_id": 947,

"display_name": "Date consultant review rec'd",

"name": "Date consultant review rec'd",

"type": "Date",

"values": "",

"created_at": "05/10/2013",

"updated_at": "09/19/2013",

"created_by": [redacted],

"searchable_quick": false,

"searchable_advanced": null,

"searchable_batch": null,

"searchable": "",

"pii": "No",

"qtip": "",

"used_by": "Sharepoint Legacy - Adult Patient Status",

"used_by_display": "<a href='#' onClick='remoteJS({name:\"dialogs/AddSubjectTypeDlg\", obj_id:115 });'>Sharepoint Legacy - Adult Patient Status</a>"

},

{

"id": 948,

"obj_id": 948,

"display_name": "Date consultant review sent",

"name": "Date consultant review sent",

"type": "Date",

"values": "",

"created_at": "05/10/2013",

"updated_at": "09/19/2013",

"created_by": [redacted],

"searchable_quick": false,

"searchable_advanced": null,

"searchable_batch": null,

"searchable": "",

"pii": "No",

"qtip": "",

"used_by": "Sharepoint Legacy - Adult Patient Status",

"used_by_display": "<a href='#' onClick='remoteJS({name:\"dialogs/AddSubjectTypeDlg\", obj_id:115 });'>Sharepoint Legacy - Adult Patient Status</a>"

},

{

"id": 1838,

"obj_id": 1838,

"display_name": "Date Consult Request Sent",

"name": "Date Consult Request Sent",

"type": "Date",

"values": "",

"created_at": "10/31/2013",

"updated_at": "10/31/2013",

"created_by": [redacted],

"searchable_quick": true,

"searchable_advanced": true,

"searchable_batch": true,

"searchable": "Quick,Advanced,Batch",

"pii": "No",

"qtip": "",

"used_by": "Consult Request",

"used_by_display": "<a href='#' onClick='remoteJS({name:\"dialogs/AddSubjectTypeDlg\", obj_id:180 });'>Consult Request</a>"

},

{

"id": 392,

"obj_id": 392,

"display_name": "Date Core Lab Contacted",

"name": "Date Core Lab Contacted",

"type": "Date",

"values": "",

"created_at": "04/15/2013",

"updated_at": "09/19/2013",

"created_by": [redacted],

"searchable_quick": false,

"searchable_advanced": null,

"searchable_batch": null,

"searchable": "",

"pii": "No",

"qtip": "",

"used_by": "SNP Analysis",

"used_by_display": "<a href='#' onClick='remoteJS({name:\"dialogs/AddSubjectTypeDlg\", obj_id:59 });'>SNP Analysis</a>"

},

{

"id": 542,

"obj_id": 542,

"display_name": "Date Created",

"name": "Date Created",

"type": "Date",

"values": "",

"created_at": "05/07/2013",

"updated_at": "07/19/2013",

"created_by": [redacted],

"searchable_quick": false,

"searchable_advanced": null,

"searchable_batch": null,

"searchable": "",

"pii": "Yes",

"qtip": "",

"used_by": "Sharepoint Legacy - Adult Patient Status, Drosophila Phenotypes, Labmatrix Legacy - Biomaterials, Zebrafish Phenotype",

"used_by_display": "<a href='#' onClick='remoteJS({name:\"dialogs/AddSubjectTypeDlg\", obj_id:115 });'>Sharepoint Legacy - Adult Patient Status</a>, <a href='#' onClick='remoteJS({name:\"dialogs/AddSubjectTypeDlg\", obj_id:265 });'>Drosophila Phenotypes</a>, <a href='#' onClick='remoteJS({name:\"dialogs/AddSubjectTypeDlg\", obj_id:91 });'>Labmatrix Legacy - Biomaterials</a>, <a href='#' onClick='remoteJS({name:\"dialogs/AddSubjectTypeDlg\", obj_id:173 });'>Zebrafish Phenotype</a>"

},

{

"id": 2424,

"obj_id": 2424,

"display_name": "Date CRISPRs Ordered",

"name": "Date CRISPRs Ordered",

"type": "Date",

"values": "",

"created_at": "03/20/2014",

"updated_at": "03/20/2014",

"created_by": [redacted],

"searchable_quick": false,

"searchable_advanced": null,

"searchable_batch": null,

"searchable": "",

"pii": "No",

"qtip": "",

"used_by": "Zebrafish Mutation Project",

"used_by_display": "<a href='#' onClick='remoteJS({name:\"dialogs/AddSubjectTypeDlg\", obj_id:186 });'>Zebrafish Mutation Project</a>"

},

{

"id": 1007,

"obj_id": 1007,

"display_name": "Date CSF processed",

"name": "Date CSF processed",

"type": "Date",

"values": "",

"created_at": "05/13/2013",

"updated_at": "08/08/2013",

"created_by": [redacted],

"searchable_quick": false,

"searchable_advanced": null,

"searchable_batch": null,

"searchable": "",

"pii": "No",

"qtip": "",

"used_by": "CSF Processing",

"used_by_display": "<a href='#' onClick='remoteJS({name:\"dialogs/AddSubjectTypeDlg\", obj_id:117 });'>CSF Processing</a>"

},

{

"id": 1035,

"obj_id": 1035,

"display_name": "Date Culture Started",

"name": "Date Culture Started",

"type": "Date",

"values": "",

"created_at": "05/15/2013",

"updated_at": "10/29/2014",

"created_by": [redacted],

"searchable_quick": false,

"searchable_advanced": true,

"searchable_batch": false,

"searchable": "Advanced",

"pii": "No",

"qtip": "",

"used_by": "Cell Culture, Research Sample, Zebrafish Culture, Rescue Cell Culture",

"used_by_display": "<a href='#' onClick='remoteJS({name:\"dialogs/AddSubjectTypeDlg\", obj_id:40 });'>Cell Culture</a>, <a href='#' onClick='remoteJS({name:\"dialogs/AddSubjectTypeDlg\", obj_id:86 });'>Research Sample</a>, <a href='#' onClick='remoteJS({name:\"dialogs/AddSubjectTypeDlg\", obj_id:234 });'>Zebrafish Culture</a>, <a href='#' onClick='remoteJS({name:\"dialogs/AddSubjectTypeDlg\", obj_id:247 });'>Rescue Cell Culture</a>"

},

{

"id": 367,

"obj_id": 367,

"display_name": "Date DNA Archived",

"name": "Date DNA Archived",

"type": "Date",

"values": "",

"created_at": "04/15/2013",

"updated_at": "09/19/2013",

"created_by": [redacted],

"searchable_quick": false,

"searchable_advanced": null,

"searchable_batch": null,

"searchable": "",

"pii": "No",

"qtip": "",

"used_by": "DNA Extraction",

"used_by_display": "<a href='#' onClick='remoteJS({name:\"dialogs/AddSubjectTypeDlg\", obj_id:122 });'>DNA Extraction</a>"

},

{

"id": 349,

"obj_id": 349,

"display_name": "Date DNA Sent for Exome",

"name": "Date DNA Sent for Exome",

"type": "Date",

"values": "",

"created_at": "04/12/2013",

"updated_at": "10/23/2014",

"created_by": [redacted],

"searchable_quick": false,

"searchable_advanced": true,

"searchable_batch": false,

"searchable": "Advanced",

"pii": "No",

"qtip": "Exome sequencing",

"used_by": "Exome Sequencing",

"used_by_display": "<a href='#' onClick='remoteJS({name:\"dialogs/AddSubjectTypeDlg\", obj_id:161 });'>Exome Sequencing</a>"

},

{

"id": 2281,

"obj_id": 2281,

"display_name": "Date DNA Sent for Genome",

"name": "Date DNA Sent for Genome",

"type": "Date",

"values": "",

"created_at": "02/18/2014",

"updated_at": "02/18/2014",

"created_by": [redacted],

"searchable_quick": false,

"searchable_advanced": null,

"searchable_batch": null,

"searchable": "",

"pii": "No",

"qtip": "",

"used_by": "Genome Sequencing",

"used_by_display": "<a href='#' onClick='remoteJS({name:\"dialogs/AddSubjectTypeDlg\", obj_id:212 });'>Genome Sequencing</a>"

},

{

"id": 71,

"obj_id": 71,

"display_name": "Date DNA Sent for SNP Chip",

"name": "Date DNA Sent for SNP Chip",

"type": "Date",

"values": "",

"created_at": "02/06/2013",

"updated_at": "10/21/2014",

"created_by": [redacted],

"searchable_quick": false,

"searchable_advanced": true,

"searchable_batch": false,

"searchable": "Advanced",

"pii": "No",

"qtip": "",

"used_by": "SNP Analysis, SNP Chip Sequencing",

"used_by_display": "<a href='#' onClick='remoteJS({name:\"dialogs/AddSubjectTypeDlg\", obj_id:59 });'>SNP Analysis</a>, <a href='#' onClick='remoteJS({name:\"dialogs/AddSubjectTypeDlg\", obj_id:203 });'>SNP Chip Sequencing</a>"

},

{

"id": 63,

"obj_id": 63,

"display_name": "Date DNA Sent for Validation",

"name": "Date DNA Sent for Validation",

"type": "Date",

"values": "",

"created_at": "02/06/2013",

"updated_at": "10/23/2014",

"created_by": [redacted],

"searchable_quick": false,

"searchable_advanced": true,

"searchable_batch": false,

"searchable": "Advanced",

"pii": "No",

"qtip": "",

"used_by": "CLIA Validation",

"used_by_display": "<a href='#' onClick='remoteJS({name:\"dialogs/AddSubjectTypeDlg\", obj_id:15 });'>CLIA Validation</a>"

},

{

"id": 547,

"obj_id": 547,

"display_name": "Date_DNA_submitted",

"name": "Date_DNA_submitted",

"type": "Date",

"values": "",

"created_at": "05/07/2013",

"updated_at": "09/19/2013",

"created_by": [redacted],

"searchable_quick": false,

"searchable_advanced": null,

"searchable_batch": null,

"searchable": "",

"pii": "No",

"qtip": "",

"used_by": "Labmatrix Legacy - SNP Chip Non UDP, Labmatrix Legacy - SNP Chip",

"used_by_display": "<a href='#' onClick='remoteJS({name:\"dialogs/AddSubjectTypeDlg\", obj_id:99 });'>Labmatrix Legacy - SNP Chip Non UDP</a>, <a href='#' onClick='remoteJS({name:\"dialogs/AddSubjectTypeDlg\", obj_id:105 });'>Labmatrix Legacy - SNP Chip</a>"

},

{

"id": 2753,

"obj_id": 2753,

"display_name": "Date [redacted] approved Missing Consent",

"name": "Date [redacted] approved Missing Consent",

"type": "Date",

"values": "",

"created_at": "08/01/2014",

"updated_at": "08/01/2014",

"created_by": [redacted],

"searchable_quick": true,

"searchable_advanced": true,

"searchable_batch": true,

"searchable": "Quick,Advanced,Batch",

"pii": "Yes",

"qtip": "",

"used_by": "Consent Signed",

"used_by_display": "<a href='#' onClick='remoteJS({name:\"dialogs/AddSubjectTypeDlg\", obj_id:141 });'>Consent Signed</a>"

},

{

"id": 2510,

"obj_id": 2510,

"display_name": "Date Entered into EZColony",

"name": "Date Entered into EZColony",

"type": "Date",

"values": "",

"created_at": "04/14/2014",

"updated_at": "04/14/2014",

"created_by": [redacted],

"searchable_quick": false,

"searchable_advanced": null,

"searchable_batch": null,

"searchable": "",

"pii": "No",

"qtip": "",

"used_by": "Zebrafish Culture",

"used_by_display": "<a href='#' onClick='remoteJS({name:\"dialogs/AddSubjectTypeDlg\", obj_id:234 });'>Zebrafish Culture</a>"

},

{

"id": 1764,

"obj_id": 1764,

"display_name": "Date Exome Analysis Complete",

"name": "Date Exome Analysis Complete",

"type": "Date",

"values": "",

"created_at": "10/22/2013",

"updated_at": "10/22/2013",

"created_by": [redacted],

"searchable_quick": true,

"searchable_advanced": true,

"searchable_batch": true,

"searchable": "Quick,Advanced,Batch",

"pii": "No",

"qtip": "",

"used_by": "Exome Analysis",

"used_by_display": "<a href='#' onClick='remoteJS({name:\"dialogs/AddSubjectTypeDlg\", obj_id:60 });'>Exome Analysis</a>"

},

{

"id": 360,

"obj_id": 360,

"display_name": "Date Extraction Complete",

"name": "Date Extraction Complete",

"type": "Date",

"values": "",

"created_at": "04/15/2013",

"updated_at": "09/19/2013",

"created_by": [redacted],

"searchable_quick": false,

"searchable_advanced": null,

"searchable_batch": null,

"searchable": "",

"pii": "No",

"qtip": "",

"used_by": "DNA Extraction",

"used_by_display": "<a href='#' onClick='remoteJS({name:\"dialogs/AddSubjectTypeDlg\", obj_id:122 });'>DNA Extraction</a>"

},

{

"id": 2430,

"obj_id": 2430,

"display_name": "Date F0 Culture Complete",

"name": "Date F0 Culture Complete",

"type": "Date",

"values": "",

"created_at": "03/24/2014",

"updated_at": "03/24/2014",

"created_by": [redacted],

"searchable_quick": false,

"searchable_advanced": null,

"searchable_batch": null,

"searchable": "",

"pii": "No",

"qtip": "Inject emrbyos reach maturity",

"used_by": "Zebrafish Mutation Project",

"used_by_display": "<a href='#' onClick='remoteJS({name:\"dialogs/AddSubjectTypeDlg\", obj_id:186 });'>Zebrafish Mutation Project</a>"

},

{

"id": 2403,

"obj_id": 2403,

"display_name": "Date F0 gDNA Isolated",

"name": "Date F0 gDNA Isolated",

"type": "Date",

"values": "",

"created_at": "03/14/2014",

"updated_at": "03/14/2014",

"created_by": [redacted],

"searchable_quick": false,

"searchable_advanced": null,

"searchable_batch": null,

"searchable": "",

"pii": "No",

"qtip": "",

"used_by": "Zebrafish Mutation Project",

"used_by_display": "<a href='#' onClick='remoteJS({name:\"dialogs/AddSubjectTypeDlg\", obj_id:186 });'>Zebrafish Mutation Project</a>"

},

{

"id": 2442,

"obj_id": 2442,

"display_name": "Date F0 Generation Entered into EZColony",

"name": "Date F0 Generation Entered into EZColony",

"type": "Date",

"values": "",

"created_at": "03/24/2014",

"updated_at": "03/24/2014",

"created_by": [redacted],

"searchable_quick": false,

"searchable_advanced": null,

"searchable_batch": null,

"searchable": "",

"pii": "No",

"qtip": "",

"used_by": "Zebrafish Mutation Project",

"used_by_display": "<a href='#' onClick='remoteJS({name:\"dialogs/AddSubjectTypeDlg\", obj_id:186 });'>Zebrafish Mutation Project</a>"

},

{

"id": 2404,

"obj_id": 2404,

"display_name": "Date F1 gDNA Extracted",

"name": "Date F1 gDNA Extracted",

"type": "Date",

"values": "",

"created_at": "03/14/2014",

"updated_at": "03/14/2014",

"created_by": [redacted],

"searchable_quick": false,

"searchable_advanced": null,

"searchable_batch": null,

"searchable": "",

"pii": "No",

"qtip": "",

"used_by": "Zebrafish Mutation Project, Zebrafish Breeding Information",

"used_by_display": "<a href='#' onClick='remoteJS({name:\"dialogs/AddSubjectTypeDlg\", obj_id:186 });'>Zebrafish Mutation Project</a>, <a href='#' onClick='remoteJS({name:\"dialogs/AddSubjectTypeDlg\", obj_id:233 });'>Zebrafish Breeding Information</a>"

},

{

"id": 2443,

"obj_id": 2443,

"display_name": "Date F1 Generation Entered into EZColony",

"name": "Date F1 Generation Entered into EZColony",

"type": "Date",

"values": "",

"created_at": "03/24/2014",

"updated_at": "03/24/2014",

"created_by": [redacted],

"searchable_quick": false,

"searchable_advanced": null,

"searchable_batch": null,

"searchable": "",

"pii": "No",

"qtip": "",

"used_by": "Zebrafish Mutation Project",

"used_by_display": "<a href='#' onClick='remoteJS({name:\"dialogs/AddSubjectTypeDlg\", obj_id:186 });'>Zebrafish Mutation Project</a>"

},

{

"id": 2055,

"obj_id": 2055,

"display_name": "Date File Extracted",

"name": "Date File Extracted",

"type": "Date",

"values": "",

"created_at": "12/04/2013",

"updated_at": "12/04/2013",

"created_by": [redacted],

"searchable_quick": false,

"searchable_advanced": null,

"searchable_batch": null,

"searchable": "",

"pii": "No",

"qtip": "",

"used_by": "Varsifter Files",

"used_by_display": "<a href='#' onClick='remoteJS({name:\"dialogs/AddSubjectTypeDlg\", obj_id:198 });'>Varsifter Files</a>"

},

{

"id": 2053,

"obj_id": 2053,

"display_name": "Date File Received/Made",

"name": "Date File Received/Made",

"type": "Date",

"values": "",

"created_at": "12/04/2013",

"updated_at": "12/04/2013",

"created_by": [redacted],

"searchable_quick": false,

"searchable_advanced": null,

"searchable_batch": null,

"searchable": "",

"pii": "No",

"qtip": "",

"used_by": "Cohort Variant Call Format File",

"used_by_display": "<a href='#' onClick='remoteJS({name:\"dialogs/AddSubjectTypeDlg\", obj_id:195 });'>Cohort Variant Call Format File</a>"

},

{

"id": 2653,

"obj_id": 2653,

"display_name": "Date FilesReceived",

"name": "Date FilesReceived",

"type": "Date",

"values": "",

"created_at": "05/29/2014",

"updated_at": "07/11/2014",

"created_by": [redacted],

"searchable_quick": false,

"searchable_advanced": false,

"searchable_batch": false,

"searchable": "",

"pii": "No",

"qtip": "",

"used_by": "",

"used_by_display": ""

},

{

"id": 1578,

"obj_id": 1578,

"display_name": "Date Files Received",

"name": "Date Files Received",

"type": "Date",

"values": "",

"created_at": "08/12/2013",

"updated_at": "08/12/2013",

"created_by": [redacted],

"searchable_quick": false,

"searchable_advanced": null,

"searchable_batch": null,

"searchable": "",

"pii": "No",

"qtip": "",

"used_by": "Information Technology Collaboration Files, Genome Sequencing, Patient Bioinformatics Files, Transcriptome",

"used_by_display": "<a href='#' onClick='remoteJS({name:\"dialogs/AddSubjectTypeDlg\", obj_id:184 });'>Information Technology Collaboration Files</a>, <a href='#' onClick='remoteJS({name:\"dialogs/AddSubjectTypeDlg\", obj_id:212 });'>Genome Sequencing</a>, <a href='#' onClick='remoteJS({name:\"dialogs/AddSubjectTypeDlg\", obj_id:235 });'>Patient Bioinformatics Files</a>, <a href='#' onClick='remoteJS({name:\"dialogs/AddSubjectTypeDlg\", obj_id:255 });'>Transcriptome</a>"

},

{

"id": 1862,

"obj_id": 1862,

"display_name": "Date Files Sent",

"name": "Date Files Sent",

"type": "Date",

"values": "",

"created_at": "11/01/2013",

"updated_at": "02/04/2014",

"created_by": [redacted],

"searchable_quick": false,

"searchable_advanced": null,

"searchable_batch": null,

"searchable": "",

"pii": "No",

"qtip": "",

"used_by": "Information Technology Collaboration Files",

"used_by_display": "<a href='#' onClick='remoteJS({name:\"dialogs/AddSubjectTypeDlg\", obj_id:184 });'>Information Technology Collaboration Files</a>"

},

{

"id": 1095,

"obj_id": 1095,

"display_name": "Date Files Sent to Toronto",

"name": "Date Files Sent to Toronto",

"type": "Date",

"values": "",

"created_at": "05/17/2013",

"updated_at": "08/20/2013",

"created_by": [redacted],

"searchable_quick": false,

"searchable_advanced": null,

"searchable_batch": null,

"searchable": "",

"pii": "No",

"qtip": "",

"used_by": "",

"used_by_display": ""

},

{

"id": 2208,

"obj_id": 2208,

"display_name": "Date Formated",

"name": "Date Formated",

"type": "Date",

"values": "",

"created_at": "02/06/2014",

"updated_at": "02/06/2014",

"created_by": [redacted],

"searchable_quick": false,

"searchable_advanced": null,

"searchable_batch": null,

"searchable": "",

"pii": "No",

"qtip": "",

"used_by": "dbGaP VCFs",

"used_by_display": "<a href='#' onClick='remoteJS({name:\"dialogs/AddSubjectTypeDlg\", obj_id:207 });'>dbGaP VCFs</a>"

},

{

"id": 2119,

"obj_id": 2119,

"display_name": "Date GS File Created",

"name": "Date GS File Created",

"type": "Date",

"values": "",

"created_at": "12/20/2013",

"updated_at": "08/29/2014",

"created_by": [redacted],

"searchable_quick": false,

"searchable_advanced": false,

"searchable_batch": false,

"searchable": "",

"pii": "No",

"qtip": "",

"used_by": "GenomeStudio Files",

"used_by_display": "<a href='#' onClick='remoteJS({name:\"dialogs/AddSubjectTypeDlg\", obj_id:202 });'>GenomeStudio Files</a>"

},

{

"id": 1891,

"obj_id": 1891,

"display_name": "Date Haplotype Caller Started",

"name": "Date Haplotype Caller Started",

"type": "Date",

"values": "",

"created_at": "11/08/2013",

"updated_at": "11/08/2013",

"created_by": [redacted],

"searchable_quick": false,

"searchable_advanced": null,

"searchable_batch": null,

"searchable": "",

"pii": "No",

"qtip": "",

"used_by": "",

"used_by_display": ""

},

{

"id": 390,

"obj_id": 390,

"display_name": "Date IDAT Files Received",

"name": "Date IDAT Files Received",

"type": "Date",

"values": "",

"created_at": "04/15/2013",

"updated_at": "10/29/2014",

"created_by": [redacted],

"searchable_quick": false,

"searchable_advanced": true,

"searchable_batch": false,

"searchable": "Advanced",

"pii": "No",

"qtip": "IDAT files from Core Lab",

"used_by": "SNP Analysis, SNP Chip Sequencing",

"used_by_display": "<a href='#' onClick='remoteJS({name:\"dialogs/AddSubjectTypeDlg\", obj_id:59 });'>SNP Analysis</a>, <a href='#' onClick='remoteJS({name:\"dialogs/AddSubjectTypeDlg\", obj_id:203 });'>SNP Chip Sequencing</a>"

},

{

"id": 2429,

"obj_id": 2429,

"display_name": "Date Injected Embryos Cultured",

"name": "Date Injected Embryos Cultured",

"type": "Date",

"values": "",

"created_at": "03/24/2014",

"updated_at": "03/24/2014",

"created_by": [redacted],

"searchable_quick": false,

"searchable_advanced": null,

"searchable_batch": null,

"searchable": "",

"pii": "No",

"qtip": "",

"used_by": "Zebrafish Mutation Project",

"used_by_display": "<a href='#' onClick='remoteJS({name:\"dialogs/AddSubjectTypeDlg\", obj_id:186 });'>Zebrafish Mutation Project</a>"

},

{

"id": 2015,

"obj_id": 2015,

"display_name": "Date Karyotyping Complete",

"name": "Date Karyotyping Complete",

"type": "Date",

"values": "",

"created_at": "11/20/2013",

"updated_at": "11/20/2013",

"created_by": [redacted],

"searchable_quick": false,

"searchable_advanced": null,

"searchable_batch": null,

"searchable": "",

"pii": "No",

"qtip": "",

"used_by": "iPS Cells",

"used_by_display": "<a href='#' onClick='remoteJS({name:\"dialogs/AddSubjectTypeDlg\", obj_id:41 });'>iPS Cells</a>"

},

{

"id": 120,

"obj_id": 120,

"display_name": "Date Kit Sent",

"name": "Date Kit Sent",

"type": "Date",

"values": "",

"created_at": "02/12/2013",

"updated_at": "07/19/2013",

"created_by": [redacted],

"searchable_quick": false,

"searchable_advanced": null,

"searchable_batch": null,

"searchable": "",

"pii": "Yes",

"qtip": "",

"used_by": "Blood Kit",

"used_by_display": "<a href='#' onClick='remoteJS({name:\"dialogs/AddSubjectTypeDlg\", obj_id:33 });'>Blood Kit</a>"

},

{

"id": 877,

"obj_id": 877,

"display_name": "Date Lane Files Received",

"name": "Date Lane Files Received",

"type": "Date",

"values": "",

"created_at": "05/08/2013",

"updated_at": "10/29/2014",

"created_by": [redacted],

"searchable_quick": false,

"searchable_advanced": true,

"searchable_batch": true,

"searchable": "Advanced,Batch",

"pii": "No",

"qtip": "FastQ Files/Lane Files",

"used_by": "Exome Analysis, Exome Sequencing, Genome Sequencing",

"used_by_display": "<a href='#' onClick='remoteJS({name:\"dialogs/AddSubjectTypeDlg\", obj_id:60 });'>Exome Analysis</a>, <a href='#' onClick='remoteJS({name:\"dialogs/AddSubjectTypeDlg\", obj_id:161 });'>Exome Sequencing</a>, <a href='#' onClick='remoteJS({name:\"dialogs/AddSubjectTypeDlg\", obj_id:212 });'>Genome Sequencing</a>"

},

{

"id": 2312,

"obj_id": 2312,

"display_name": "Date Last Discussed in Screening Meeting",

"name": "Date Last Discussed in Screening Meeting",

"type": "Date",

"values": "",

"created_at": "02/28/2014",

"updated_at": "06/10/2014",

"created_by": [redacted],

"searchable_quick": false,

"searchable_advanced": null,

"searchable_batch": null,

"searchable": "",

"pii": "Yes",

"qtip": "",

"used_by": "Patient, Patient Follow Up Visit, Clinical Notes",

"used_by_display": "<a href='#' onClick='remoteJS({name:\"dialogs/AddSubjectTypeDlg\", obj_id:1 });'>Patient</a>, <a href='#' onClick='remoteJS({name:\"dialogs/AddSubjectTypeDlg\", obj_id:264 });'>Patient Follow Up Visit</a>, <a href='#' onClick='remoteJS({name:\"dialogs/AddSubjectTypeDlg\", obj_id:267 });'>Clinical Notes</a>"

},

{

"id": 2830,

"obj_id": 2830,

"display_name": "Date Last Discussed in Screening Meeting:",

"name": "Date Last Discussed in Screening Meeting:",

"type": "Date",

"values": "",

"created_at": "09/12/2014",

"updated_at": "09/12/2014",

"created_by": [redacted],

"searchable_quick": false,

"searchable_advanced": false,

"searchable_batch": false,

"searchable": "",

"pii": "Yes",

"qtip": "",

"used_by": "",

"used_by_display": ""

},

{

"id": 932,

"obj_id": 932,

"display_name": "Date Last Review",

"name": "Date Last Review",

"type": "Date",

"values": "",

"created_at": "05/10/2013",

"updated_at": "07/19/2013",

"created_by": [redacted],

"searchable_quick": false,

"searchable_advanced": null,

"searchable_batch": null,

"searchable": "",

"pii": "Yes",

"qtip": "",

"used_by": "Sharepoint Legacy - Pediatric Patient",

"used_by_display": "<a href='#' onClick='remoteJS({name:\"dialogs/AddSubjectTypeDlg\", obj_id:114 });'>Sharepoint Legacy - Pediatric Patient</a>"

},

{

"id": 1338,

"obj_id": 1338,

"display_name": "Date Last Taken",

"name": "Date Last Taken",

"type": "Date",

"values": "",

"created_at": "06/20/2013",

"updated_at": "07/19/2013",

"created_by": [redacted],

"searchable_quick": false,

"searchable_advanced": null,

"searchable_batch": null,

"searchable": "",

"pii": "Yes",

"qtip": "",

"used_by": "Medication",

"used_by_display": "<a href='#' onClick='remoteJS({name:\"dialogs/AddSubjectTypeDlg\", obj_id:142 });'>Medication</a>"

},

{

"id": 221,

"obj_id": 221,

"display_name": "Date Medical Records Scanned",

"name": "Date Medical Records Scanned",

"type": "Date",

"values": "",

"created_at": "03/26/2013",

"updated_at": "07/19/2013",

"created_by": [redacted],

"searchable_quick": false,

"searchable_advanced": null,

"searchable_batch": null,

"searchable": "",

"pii": "Yes",

"qtip": "",

"used_by": "Patient Documents Scan & Sort",

"used_by_display": "<a href='#' onClick='remoteJS({name:\"dialogs/AddSubjectTypeDlg\", obj_id:88 });'>Patient Documents Scan & Sort</a>"

},

{

"id": 2733,

"obj_id": 2733,

"display_name": "Date of Admission",

"name": "Date of Admission",

"type": "Date",

"values": "",

"created_at": "07/18/2014",

"updated_at": "07/18/2014",

"created_by": [redacted],

"searchable_quick": true,

"searchable_advanced": true,

"searchable_batch": true,

"searchable": "Quick,Advanced,Batch",

"pii": "Yes",

"qtip": "",

"used_by": "Patient, Patient Follow Up Visit",

"used_by_display": "<a href='#' onClick='remoteJS({name:\"dialogs/AddSubjectTypeDlg\", obj_id:1 });'>Patient</a>, <a href='#' onClick='remoteJS({name:\"dialogs/AddSubjectTypeDlg\", obj_id:264 });'>Patient Follow Up Visit</a>"

},

{

"id": 2693,

"obj_id": 2693,

"display_name": "Date of Amendment",

"name": "Date of Amendment",

"type": "Date",

"values": "",

"created_at": "06/25/2014",

"updated_at": "06/25/2014",

"created_by": [redacted],

"searchable_quick": false,

"searchable_advanced": null,

"searchable_batch": null,

"searchable": "",

"pii": "No",

"qtip": "",

"used_by": "MTA/ITA Amendments",

"used_by_display": "<a href='#' onClick='remoteJS({name:\"dialogs/AddSubjectTypeDlg\", obj_id:251 });'>MTA/ITA Amendments</a>"

},

{

"id": 2776,

"obj_id": 2776,

"display_name": "Date of Amplification",

"name": "Date of Amplification",

"type": "Date",

"values": "",

"created_at": "08/04/2014",

"updated_at": "08/04/2014",

"created_by": [redacted],

"searchable_quick": false,

"searchable_advanced": false,

"searchable_batch": false,

"searchable": "",

"pii": "No",

"qtip": "",

"used_by": "Genome Amplification",

"used_by_display": "<a href='#' onClick='remoteJS({name:\"dialogs/AddSubjectTypeDlg\", obj_id:258 });'>Genome Amplification</a>"

},

{

"id": 4,

"obj_id": 4,

"display_name": "Date of Birth",

"name": "Date of Birth",

"type": "Date",

"values": "",

"created_at": "01/28/2013",

"updated_at": "01/23/2015",

"created_by": [redacted],

"searchable_quick": true,

"searchable_advanced": true,

"searchable_batch": true,

"searchable": "Quick,Advanced,Batch",

"pii": "Yes",

"qtip": "If unknown use \"01/01/1900\"",

"used_by": "UDP Inquiry, Labmatrix Legacy - Peds Sharepoint Seen, Labmatrix Legacy - Peds Sharepoint Seen List, Patient Follow Up Visit, Sharepoint Legacy - Pediatric Patient, Sharepoint Legacy - Adult Patient Status, Labmatrix Legacy - Subjects, Patient Visit Tools, Patient Review, Patient",

"used_by_display": "<a href='#' onClick='remoteJS({name:\"dialogs/AddSubjectTypeDlg\", obj_id:61 });'>UDP Inquiry</a>, <a href='#' onClick='remoteJS({name:\"dialogs/AddSubjectTypeDlg\", obj_id:97 });'>Labmatrix Legacy - Peds Sharepoint Seen</a>, <a href='#' onClick='remoteJS({name:\"dialogs/AddSubjectTypeDlg\", obj_id:98 });'>Labmatrix Legacy - Peds Sharepoint Seen List</a>, <a href='#' onClick='remoteJS({name:\"dialogs/AddSubjectTypeDlg\", obj_id:264 });'>Patient Follow Up Visit</a>, <a href='#' onClick='remoteJS({name:\"dialogs/AddSubjectTypeDlg\", obj_id:114 });'>Sharepoint Legacy - Pediatric Patient</a>, <a href='#' onClick='remoteJS({name:\"dialogs/AddSubjectTypeDlg\", obj_id:115 });'>Sharepoint Legacy - Adult Patient Status</a>, <a href='#' onClick='remoteJS({name:\"dialogs/AddSubjectTypeDlg\", obj_id:112 });'>Labmatrix Legacy - Subjects</a>, <a href='#' onClick='remoteJS({name:\"dialogs/AddSubjectTypeDlg\", obj_id:163 });'>Patient Visit Tools</a>, <a href='#' onClick='remoteJS({name:\"dialogs/AddSubjectTypeDlg\", obj_id:83 });'>Patient Review</a>, <a href='#' onClick='remoteJS({name:\"dialogs/AddSubjectTypeDlg\", obj_id:1 });'>Patient</a>"

},

{

"id": 543,

"obj_id": 543,

"display_name": "Date of Birth - Anticipated",

"name": "Date of Birth - Anticipated",

"type": "Date",

"values": "",

"created_at": "05/07/2013",

"updated_at": "07/19/2013",

"created_by": [redacted],

"searchable_quick": false,

"searchable_advanced": null,

"searchable_batch": null,

"searchable": "",

"pii": "Yes",

"qtip": "",

"used_by": "Labmatrix Legacy - Subjects",

"used_by_display": "<a href='#' onClick='remoteJS({name:\"dialogs/AddSubjectTypeDlg\", obj_id:112 });'>Labmatrix Legacy - Subjects</a>"

},

{

"id": 1269,

"obj_id": 1269,

"display_name": "Date of Contact",

"name": "Date of Contact",

"type": "Date",

"values": "",

"created_at": "06/03/2013",

"updated_at": "07/19/2013",

"created_by": [redacted],

"searchable_quick": false,

"searchable_advanced": null,

"searchable_batch": null,

"searchable": "",

"pii": "Yes",

"qtip": "",

"used_by": "NIH Visit, External Communication",

"used_by_display": "<a href='#' onClick='remoteJS({name:\"dialogs/AddSubjectTypeDlg\", obj_id:140 });'>NIH Visit</a>, <a href='#' onClick='remoteJS({name:\"dialogs/AddSubjectTypeDlg\", obj_id:190 });'>External Communication</a>"

},

{

"id": 1330,

"obj_id": 1330,

"display_name": "Date of Death",

"name": "Date of Death",

"type": "Date",

"values": "",

"created_at": "06/18/2013",

"updated_at": "07/19/2013",

"created_by": [redacted],

"searchable_quick": false,

"searchable_advanced": null,

"searchable_batch": null,

"searchable": "",

"pii": "Yes",

"qtip": "",

"used_by": "Patient, Labmatrix Legacy - Subjects, Patient Follow Up Visit",

"used_by_display": "<a href='#' onClick='remoteJS({name:\"dialogs/AddSubjectTypeDlg\", obj_id:1 });'>Patient</a>, <a href='#' onClick='remoteJS({name:\"dialogs/AddSubjectTypeDlg\", obj_id:112 });'>Labmatrix Legacy - Subjects</a>, <a href='#' onClick='remoteJS({name:\"dialogs/AddSubjectTypeDlg\", obj_id:264 });'>Patient Follow Up Visit</a>"

},

{

"id": 2014,

"obj_id": 2014,

"display_name": "Date of Embryoid Body Formation",

"name": "Date of Embryoid Body Formation",

"type": "Date",

"values": "",

"created_at": "11/20/2013",

"updated_at": "11/20/2013",

"created_by": [redacted],

"searchable_quick": false,

"searchable_advanced": null,

"searchable_batch": null,

"searchable": "",

"pii": "No",

"qtip": "",

"used_by": "iPS Cells",

"used_by_display": "<a href='#' onClick='remoteJS({name:\"dialogs/AddSubjectTypeDlg\", obj_id:41 });'>iPS Cells</a>"

},

{

"id": 1972,

"obj_id": 1972,

"display_name": "Date of F0 Breeding",

"name": "Date of F0 Breeding",

"type": "Date",

"values": "",

"created_at": "11/18/2013",

"updated_at": "03/24/2014",

"created_by": [redacted],

"searchable_quick": false,

"searchable_advanced": null,

"searchable_batch": null,

"searchable": "",

"pii": "No",

"qtip": "WT x Injected",

"used_by": "Zebrafish Mutation Project, Zebrafish Breeding Information",

"used_by_display": "<a href='#' onClick='remoteJS({name:\"dialogs/AddSubjectTypeDlg\", obj_id:186 });'>Zebrafish Mutation Project</a>, <a href='#' onClick='remoteJS({name:\"dialogs/AddSubjectTypeDlg\", obj_id:233 });'>Zebrafish Breeding Information</a>"

},

{

"id": 2405,

"obj_id": 2405,

"display_name": "Date of F1 Breeding",

"name": "Date of F1 Breeding",

"type": "Date",

"values": "",

"created_at": "03/14/2014",

"updated_at": "03/24/2014",

"created_by": [redacted],

"searchable_quick": false,

"searchable_advanced": null,

"searchable_batch": null,

"searchable": "",

"pii": "No",

"qtip": "",

"used_by": "Zebrafish Mutation Project",

"used_by_display": "<a href='#' onClick='remoteJS({name:\"dialogs/AddSubjectTypeDlg\", obj_id:186 });'>Zebrafish Mutation Project</a>"

},

{

"id": 1947,

"obj_id": 1947,

"display_name": "Date of Fifth Passage",

"name": "Date of Fifth Passage",

"type": "Date",

"values": "",

"created_at": "11/13/2013",

"updated_at": "11/13/2013",

"created_by": [redacted],

"searchable_quick": false,

"searchable_advanced": null,

"searchable_batch": null,

"searchable": "",

"pii": "No",

"qtip": "",

"used_by": "iPS Cells",

"used_by_display": "<a href='#' onClick='remoteJS({name:\"dialogs/AddSubjectTypeDlg\", obj_id:41 });'>iPS Cells</a>"

},

{

"id": 2986,

"obj_id": 2986,

"display_name": "Date of Final Sign Off or Revision",

"name": "Date of Final Sign Off or Revision",

"type": "Date",

"values": "",

"created_at": "01/22/2015",

"updated_at": "01/22/2015",

"created_by": [redacted],

"searchable_quick": false,

"searchable_advanced": true,

"searchable_batch": false,

"searchable": "Advanced",

"pii": "No",

"qtip": "",

"used_by": "Variant Prioritization",

"used_by_display": "<a href='#' onClick='remoteJS({name:\"dialogs/AddSubjectTypeDlg\", obj_id:279 });'>Variant Prioritization</a>"

},

{

"id": 1970,

"obj_id": 1970,

"display_name": "Date of First Injection",

"name": "Date of First Injection",

"type": "Date",

"values": "",

"created_at": "11/18/2013",

"updated_at": "03/24/2014",

"created_by": [redacted],

"searchable_quick": false,

"searchable_advanced": null,

"searchable_batch": null,

"searchable": "",

"pii": "No",

"qtip": "",

"used_by": "Zebrafish Mutation Project, Zebrafish Injections",

"used_by_display": "<a href='#' onClick='remoteJS({name:\"dialogs/AddSubjectTypeDlg\", obj_id:186 });'>Zebrafish Mutation Project</a>, <a href='#' onClick='remoteJS({name:\"dialogs/AddSubjectTypeDlg\", obj_id:232 });'>Zebrafish Injections</a>"

},

{

"id": 1943,

"obj_id": 1943,

"display_name": "Date of First Passage",

"name": "Date of First Passage",

"type": "Date",

"values": "",

"created_at": "11/13/2013",

"updated_at": "11/13/2013",

"created_by": [redacted],

"searchable_quick": false,

"searchable_advanced": null,

"searchable_batch": null,

"searchable": "",

"pii": "No",

"qtip": "",

"used_by": "iPS Cells",

"used_by_display": "<a href='#' onClick='remoteJS({name:\"dialogs/AddSubjectTypeDlg\", obj_id:41 });'>iPS Cells</a>"

},

{

"id": 548,

"obj_id": 548,

"display_name": "Date_of_follow-up_phone_call",

"name": "Date_of_follow-up_phone_call",

"type": "Date",

"values": "",

"created_at": "05/07/2013",

"updated_at": "07/19/2013",

"created_by": [redacted],

"searchable_quick": false,

"searchable_advanced": null,

"searchable_batch": null,

"searchable": "",

"pii": "Yes",

"qtip": "",

"used_by": "Labmatrix Legacy - FEDEX Specimen Kit Tracking",

"used_by_display": "<a href='#' onClick='remoteJS({name:\"dialogs/AddSubjectTypeDlg\", obj_id:103 });'>Labmatrix Legacy - FEDEX Specimen Kit Tracking</a>"

},

{

"id": 1946,

"obj_id": 1946,

"display_name": "Date of Fourth Passage",

"name": "Date of Fourth Passage",

"type": "Date",

"values": "",

"created_at": "11/13/2013",

"updated_at": "11/13/2013",

"created_by": [redacted],

"searchable_quick": false,

"searchable_advanced": null,

"searchable_batch": null,

"searchable": "",

"pii": "No",

"qtip": "",

"used_by": "iPS Cells",

"used_by_display": "<a href='#' onClick='remoteJS({name:\"dialogs/AddSubjectTypeDlg\", obj_id:41 });'>iPS Cells</a>"

},

{

"id": 2765,

"obj_id": 2765,

"display_name": "Date of Gene List",

"name": "Date of Gene List",

"type": "Date",

"values": "",

"created_at": "08/04/2014",

"updated_at": "08/04/2014",

"created_by": [redacted],

"searchable_quick": false,

"searchable_advanced": false,

"searchable_batch": false,

"searchable": "",

"pii": "No",

"qtip": "(Versioning of Gene Lists)",

"used_by": "Established Gene List",

"used_by_display": "<a href='#' onClick='remoteJS({name:\"dialogs/AddSubjectTypeDlg\", obj_id:257 });'>Established Gene List</a>"

},

{

"id": 1998,

"obj_id": 1998,

"display_name": "Date of Infection",

"name": "Date of Infection",

"type": "Date",

"values": "",

"created_at": "11/20/2013",

"updated_at": "11/20/2013",

"created_by": [redacted],

"searchable_quick": false,

"searchable_advanced": null,

"searchable_batch": null,

"searchable": "",

"pii": "No",

"qtip": "",

"used_by": "iPS Cells",

"used_by_display": "<a href='#' onClick='remoteJS({name:\"dialogs/AddSubjectTypeDlg\", obj_id:41 });'>iPS Cells</a>"

},

{

"id": 949,

"obj_id": 949,

"display_name": "Date of Inq",

"name": "Date of Inq",

"type": "Date",

"values": "",

"created_at": "05/10/2013",

"updated_at": "07/19/2013",

"created_by": [redacted],

"searchable_quick": false,

"searchable_advanced": null,

"searchable_batch": null,

"searchable": "",

"pii": "Yes",

"qtip": "",

"used_by": "Sharepoint Legacy - Adult Patient Status",

"used_by_display": "<a href='#' onClick='remoteJS({name:\"dialogs/AddSubjectTypeDlg\", obj_id:115 });'>Sharepoint Legacy - Adult Patient Status</a>"

},

{

"id": 2780,

"obj_id": 2780,

"display_name": "Date of Meeting",

"name": "Date of Meeting",

"type": "Date",

"values": "",

"created_at": "08/06/2014",

"updated_at": "08/06/2014",

"created_by": [redacted],

"searchable_quick": false,

"searchable_advanced": false,

"searchable_batch": false,

"searchable": "",

"pii": "No",

"qtip": "",

"used_by": "\"-omics\" Collaboration Meeting Notes",

"used_by_display": "<a href='#' onClick='remoteJS({name:\"dialogs/AddSubjectTypeDlg\", obj_id:260 });'>\"-omics\" Collaboration Meeting Notes</a>"

},

{

"id": 893,

"obj_id": 893,

"display_name": "Date of PCR Amplification",

"name": "Date of PCR Amplification",

"type": "Date",

"values": "",

"created_at": "05/10/2013",

"updated_at": "02/11/2014",

"created_by": [redacted],

"searchable_quick": false,

"searchable_advanced": null,

"searchable_batch": null,

"searchable": "",

"pii": "No",

"qtip": "",

"used_by": "Sanger Interpretation",

"used_by_display": "<a href='#' onClick='remoteJS({name:\"dialogs/AddSubjectTypeDlg\", obj_id:22 });'>Sanger Interpretation</a>"

},

{

"id": 2615,

"obj_id": 2615,

"display_name": "Date of Release/Consent ",

"name": "Date of Release/Consent ",

"type": "Date",

"values": "",

"created_at": "05/13/2014",

"updated_at": "05/13/2014",

"created_by": [redacted],

"searchable_quick": true,

"searchable_advanced": true,

"searchable_batch": true,

"searchable": "Quick,Advanced,Batch",

"pii": "Yes",

"qtip": "Recording, Filming, Photographing",

"used_by": "Photography Release",

"used_by_display": "<a href='#' onClick='remoteJS({name:\"dialogs/AddSubjectTypeDlg\", obj_id:242 });'>Photography Release</a>"

},

{

"id": 1744,

"obj_id": 1744,

"display_name": "Date of Report",

"name": "Date of Report",

"type": "Date",

"values": "",

"created_at": "10/18/2013",

"updated_at": "06/10/2014",

"created_by": [redacted],

"searchable_quick": true,

"searchable_advanced": true,

"searchable_batch": true,

"searchable": "Quick,Advanced,Batch",

"pii": "Yes",

"qtip": "",

"used_by": "PII - Patient File Uploads, Non - PII Patient File Uploads, ChIP-Seq",

"used_by_display": "<a href='#' onClick='remoteJS({name:\"dialogs/AddSubjectTypeDlg\", obj_id:172 });'>PII - Patient File Uploads</a>, <a href='#' onClick='remoteJS({name:\"dialogs/AddSubjectTypeDlg\", obj_id:201 });'>Non - PII Patient File Uploads</a>, <a href='#' onClick='remoteJS({name:\"dialogs/AddSubjectTypeDlg\", obj_id:254 });'>ChIP-Seq</a>"

},

{

"id": 1944,

"obj_id": 1944,

"display_name": "Date of Second Passage",

"name": "Date of Second Passage",

"type": "Date",

"values": "",

"created_at": "11/13/2013",

"updated_at": "11/13/2013",

"created_by": [redacted],

"searchable_quick": false,

"searchable_advanced": null,

"searchable_batch": null,

"searchable": "",

"pii": "No",

"qtip": "",

"used_by": "iPS Cells",

"used_by_display": "<a href='#' onClick='remoteJS({name:\"dialogs/AddSubjectTypeDlg\", obj_id:41 });'>iPS Cells</a>"

},

{

"id": 1945,

"obj_id": 1945,

"display_name": "Date of Third Passage",

"name": "Date of Third Passage",

"type": "Date",

"values": "",

"created_at": "11/13/2013",

"updated_at": "11/13/2013",

"created_by": [redacted],

"searchable_quick": false,

"searchable_advanced": null,

"searchable_batch": null,

"searchable": "",

"pii": "No",

"qtip": "",

"used_by": "iPS Cells",

"used_by_display": "<a href='#' onClick='remoteJS({name:\"dialogs/AddSubjectTypeDlg\", obj_id:41 });'>iPS Cells</a>"

},

{

"id": 2865,

"obj_id": 2865,

"display_name": "Date of UDP Request",

"name": "Date of UDP Request",

"type": "Date",

"values": "",

"created_at": "10/29/2014",

"updated_at": "10/29/2014",

"created_by": [redacted],

"searchable_quick": false,

"searchable_advanced": true,

"searchable_batch": false,

"searchable": "Advanced",

"pii": "No",

"qtip": "",

"used_by": "",

"used_by_display": ""

},

{

"id": 1364,

"obj_id": 1364,

"display_name": "Date of Visit Assigned and Recorded on Calendar",

"name": "Date of Visit Assigned and Recorded on Calendar",

"type": "Radio Buttons",

"values": "Yes, No",

"created_at": "06/24/2013",

"updated_at": "07/19/2013",

"created_by": [redacted],

"searchable_quick": false,

"searchable_advanced": null,

"searchable_batch": null,

"searchable": "",

"pii": "Yes",

"qtip": "",

"used_by": "Patient Visit Tools, Patient Visit Planning for Version C",

"used_by_display": "<a href='#' onClick='remoteJS({name:\"dialogs/AddSubjectTypeDlg\", obj_id:163 });'>Patient Visit Tools</a>, <a href='#' onClick='remoteJS({name:\"dialogs/AddSubjectTypeDlg\", obj_id:192 });'>Patient Visit Planning for Version C</a>"

},

{

"id": 1472,

"obj_id": 1472,

"display_name": "Date Ordered",

"name": "Date Ordered",

"type": "Date",

"values": "",

"created_at": "07/19/2013",

"updated_at": "07/19/2013",

"created_by": [redacted],

"searchable_quick": false,

"searchable_advanced": null,

"searchable_batch": null,

"searchable": "",

"pii": "No",

"qtip": "",

"used_by": "Sanger Interpretation, Zebrafish Mutation Project, Primer Order, Medication, Overnight Collaboration Shipment",

"used_by_display": "<a href='#' onClick='remoteJS({name:\"dialogs/AddSubjectTypeDlg\", obj_id:22 });'>Sanger Interpretation</a>, <a href='#' onClick='remoteJS({name:\"dialogs/AddSubjectTypeDlg\", obj_id:186 });'>Zebrafish Mutation Project</a>, <a href='#' onClick='remoteJS({name:\"dialogs/AddSubjectTypeDlg\", obj_id:217 });'>Primer Order</a>, <a href='#' onClick='remoteJS({name:\"dialogs/AddSubjectTypeDlg\", obj_id:142 });'>Medication</a>, <a href='#' onClick='remoteJS({name:\"dialogs/AddSubjectTypeDlg\", obj_id:273 });'>Overnight Collaboration Shipment</a>"

},

{

"id": 2861,

"obj_id": 2861,

"display_name": "Date Patient Letter Created",

"name": "Date Patient Letter Created",

"type": "Date",

"values": "",

"created_at": "10/27/2014",

"updated_at": "10/27/2014",

"created_by": [redacted],

"searchable_quick": true,

"searchable_advanced": true,

"searchable_batch": true,

"searchable": "Quick,Advanced,Batch",

"pii": "Yes",

"qtip": "",

"used_by": "Patient",

"used_by_display": "<a href='#' onClick='remoteJS({name:\"dialogs/AddSubjectTypeDlg\", obj_id:1 });'>Patient</a>"

},

{

"id": 154,

"obj_id": 154,

"display_name": "Date PennCNV Run",

"name": "Date PennCNV Run",

"type": "Date",

"values": "",

"created_at": "02/20/2013",

"updated_at": "09/19/2013",

"created_by": [redacted],

"searchable_quick": false,

"searchable_advanced": null,

"searchable_batch": null,

"searchable": "",

"pii": "No",

"qtip": "",

"used_by": "SNP Analysis",

"used_by_display": "<a href='#' onClick='remoteJS({name:\"dialogs/AddSubjectTypeDlg\", obj_id:59 });'>SNP Analysis</a>"

},

{

"id": 1899,

"obj_id": 1899,

"display_name": "Date Phased VCF File Created",

"name": "Date Phased VCF File Created",

"type": "Date",

"values": "",

"created_at": "11/08/2013",

"updated_at": "11/08/2013",

"created_by": [redacted],

"searchable_quick": false,

"searchable_advanced": null,

"searchable_batch": null,

"searchable": "",

"pii": "No",

"qtip": "",

"used_by": "Phased and Imputed VCF",

"used_by_display": "<a href='#' onClick='remoteJS({name:\"dialogs/AddSubjectTypeDlg\", obj_id:188 });'>Phased and Imputed VCF</a>"

},

{

"id": 2681,

"obj_id": 2681,

"display_name": "Date Phenotype Data Updated",

"name": "Date Phenotype Data Updated",

"type": "Date",

"values": "",

"created_at": "06/17/2014",

"updated_at": "06/17/2014",

"created_by": [redacted],

"searchable_quick": false,

"searchable_advanced": null,

"searchable_batch": null,

"searchable": "",

"pii": "No",

"qtip": "",

"used_by": "PhenomeCentral Data Submission",

"used_by_display": "<a href='#' onClick='remoteJS({name:\"dialogs/AddSubjectTypeDlg\", obj_id:216 });'>PhenomeCentral Data Submission</a>"

},

{

"id": 1021,

"obj_id": 1021,

"display_name": "Date Plasma Extracted",

"name": "Date Plasma Extracted",

"type": "Date",

"values": "",

"created_at": "05/14/2013",

"updated_at": "09/19/2013",

"created_by": [redacted],

"searchable_quick": false,

"searchable_advanced": null,

"searchable_batch": null,

"searchable": "",

"pii": "No",

"qtip": "",

"used_by": "Plasma Extraction",

"used_by_display": "<a href='#' onClick='remoteJS({name:\"dialogs/AddSubjectTypeDlg\", obj_id:47 });'>Plasma Extraction</a>"

},

{

"id": 2583,

"obj_id": 2583,

"display_name": "Date Posted to Web",

"name": "Date Posted to Web",

"type": "Subject",

"values": "",

"created_at": "05/01/2014",

"updated_at": "05/01/2014",

"created_by": [redacted],

"searchable_quick": true,

"searchable_advanced": true,

"searchable_batch": true,

"searchable": "Quick,Advanced,Batch",

"pii": "No",

"qtip": "",

"used_by": "Consent Signed",

"used_by_display": "<a href='#' onClick='remoteJS({name:\"dialogs/AddSubjectTypeDlg\", obj_id:141 });'>Consent Signed</a>"

},

{

"id": 2423,

"obj_id": 2423,

"display_name": "Date Primers Ordered",

"name": "Date Primers Ordered",

"type": "Date",

"values": "",

"created_at": "03/20/2014",

"updated_at": "03/20/2014",

"created_by": [redacted],

"searchable_quick": false,

"searchable_advanced": null,

"searchable_batch": null,

"searchable": "",

"pii": "No",

"qtip": "",

"used_by": "Zebrafish Mutation Project",

"used_by_display": "<a href='#' onClick='remoteJS({name:\"dialogs/AddSubjectTypeDlg\", obj_id:186 });'>Zebrafish Mutation Project</a>"

},

{

"id": 114,

"obj_id": 114,

"display_name": "Date Received",

"name": "Date Received",

"type": "Date",

"values": "",

"created_at": "02/12/2013",

"updated_at": "10/28/2014",

"created_by": [redacted],

"searchable_quick": false,

"searchable_advanced": true,

"searchable_batch": false,

"searchable": "Advanced",

"pii": "No",

"qtip": "",

"used_by": "Collaboration Reports, Patient Medical Material, DNA Extraction, Collaborations, Glycomics Results, Blood Kit, Labmatrix Legacy - Biomaterials, Primer Order, Patient Letter, AB1 Sequence Files, Letters, Alignment Collaboration Files, Lymphoblast Culture Request",

"used_by_display": "<a href='#' onClick='remoteJS({name:\"dialogs/AddSubjectTypeDlg\", obj_id:178 });'>Collaboration Reports</a>, <a href='#' onClick='remoteJS({name:\"dialogs/AddSubjectTypeDlg\", obj_id:32 });'>Patient Medical Material</a>, <a href='#' onClick='remoteJS({name:\"dialogs/AddSubjectTypeDlg\", obj_id:122 });'>DNA Extraction</a>, <a href='#' onClick='remoteJS({name:\"dialogs/AddSubjectTypeDlg\", obj_id:147 });'>Collaborations</a>, <a href='#' onClick='remoteJS({name:\"dialogs/AddSubjectTypeDlg\", obj_id:176 });'>Glycomics Results</a>, <a href='#' onClick='remoteJS({name:\"dialogs/AddSubjectTypeDlg\", obj_id:33 });'>Blood Kit</a>, <a href='#' onClick='remoteJS({name:\"dialogs/AddSubjectTypeDlg\", obj_id:91 });'>Labmatrix Legacy - Biomaterials</a>, <a href='#' onClick='remoteJS({name:\"dialogs/AddSubjectTypeDlg\", obj_id:217 });'>Primer Order</a>, <a href='#' onClick='remoteJS({name:\"dialogs/AddSubjectTypeDlg\", obj_id:87 });'>Patient Letter</a>, <a href='#' onClick='remoteJS({name:\"dialogs/AddSubjectTypeDlg\", obj_id:250 });'>AB1 Sequence Files</a>, <a href='#' onClick='remoteJS({name:\"dialogs/AddSubjectTypeDlg\", obj_id:253 });'>Letters</a>, <a href='#' onClick='remoteJS({name:\"dialogs/AddSubjectTypeDlg\", obj_id:194 });'>Alignment Collaboration Files</a>, <a href='#' onClick='remoteJS({name:\"dialogs/AddSubjectTypeDlg\", obj_id:272 });'>Lymphoblast Culture Request</a>"

},

{

"id": 950,

"obj_id": 950,

"display_name": "Date Reject",

"name": "Date Reject",

"type": "Date",

"values": "",

"created_at": "05/10/2013",

"updated_at": "07/19/2013",

"created_by": [redacted],

"searchable_quick": false,

"searchable_advanced": null,

"searchable_batch": null,

"searchable": "",

"pii": "Yes",

"qtip": "",

"used_by": "Sharepoint Legacy - Adult Patient Status",

"used_by_display": "<a href='#' onClick='remoteJS({name:\"dialogs/AddSubjectTypeDlg\", obj_id:115 });'>Sharepoint Legacy - Adult Patient Status</a>"

},

{

"id": 1644,

"obj_id": 1644,

"display_name": "Date Reported",

"name": "Date Reported",

"type": "Date",

"values": "",

"created_at": "08/12/2013",

"updated_at": "10/28/2014",

"created_by": [redacted],

"searchable_quick": false,

"searchable_advanced": true,

"searchable_batch": false,

"searchable": "Advanced",

"pii": "No",

"qtip": "",

"used_by": "Glycomics Results",

"used_by_display": "<a href='#' onClick='remoteJS({name:\"dialogs/AddSubjectTypeDlg\", obj_id:176 });'>Glycomics Results</a>"

},

{

"id": 2866,

"obj_id": 2866,

"display_name": "Date Requested",

"name": "Date Requested",

"type": "Date",

"values": "",

"created_at": "10/29/2014",

"updated_at": "10/29/2014",

"created_by": [redacted],

"searchable_quick": false,

"searchable_advanced": true,

"searchable_batch": false,

"searchable": "Advanced",

"pii": "No",

"qtip": "",

"used_by": "Lymphoblast Culture Request",

"used_by_display": "<a href='#' onClick='remoteJS({name:\"dialogs/AddSubjectTypeDlg\", obj_id:272 });'>Lymphoblast Culture Request</a>"

},

{

"id": 1043,

"obj_id": 1043,

"display_name": "Date RNA Processed",

"name": "Date RNA Processed",

"type": "Date",

"values": "",

"created_at": "05/15/2013",

"updated_at": "09/19/2013",

"created_by": [redacted],

"searchable_quick": false,

"searchable_advanced": null,

"searchable_batch": null,

"searchable": "",

"pii": "No",

"qtip": "",

"used_by": "RNA Extraction",

"used_by_display": "<a href='#' onClick='remoteJS({name:\"dialogs/AddSubjectTypeDlg\", obj_id:43 });'>RNA Extraction</a>"

},

{

"id": 1672,

"obj_id": 1672,

"display_name": "Date Sample Sent",

"name": "Date Sample Sent",

"type": "Date",

"values": "",

"created_at": "08/27/2013",

"updated_at": "08/27/2013",

"created_by": [redacted],

"searchable_quick": false,

"searchable_advanced": null,

"searchable_batch": null,

"searchable": "",

"pii": "No",

"qtip": "",

"used_by": "Collaborations, Requisition, Glycomics Collaborations, Send Out Testing",

"used_by_display": "<a href='#' onClick='remoteJS({name:\"dialogs/AddSubjectTypeDlg\", obj_id:147 });'>Collaborations</a>, <a href='#' onClick='remoteJS({name:\"dialogs/AddSubjectTypeDlg\", obj_id:167 });'>Requisition</a>, <a href='#' onClick='remoteJS({name:\"dialogs/AddSubjectTypeDlg\", obj_id:244 });'>Glycomics Collaborations</a>, <a href='#' onClick='remoteJS({name:\"dialogs/AddSubjectTypeDlg\", obj_id:278 });'>Send Out Testing</a>"

},

{

"id": 2065,

"obj_id": 2065,

"display_name": "Date Samples Received at Axeq",

"name": "Date Samples Received at Axeq",

"type": "Date",

"values": "",

"created_at": "12/09/2013",

"updated_at": "12/09/2013",

"created_by": [redacted],

"searchable_quick": false,

"searchable_advanced": null,

"searchable_batch": null,

"searchable": "",

"pii": "No",

"qtip": "",

"used_by": "Exome Sequencing, Genome Sequencing",

"used_by_display": "<a href='#' onClick='remoteJS({name:\"dialogs/AddSubjectTypeDlg\", obj_id:161 });'>Exome Sequencing</a>, <a href='#' onClick='remoteJS({name:\"dialogs/AddSubjectTypeDlg\", obj_id:212 });'>Genome Sequencing</a>"

},

{

"id": 80,

"obj_id": 80,

"display_name": "Date Sample(s) Stored",

"name": "Date Sample(s) Stored",

"type": "Date",

"values": "",

"created_at": "02/06/2013",

"updated_at": "09/19/2013",

"created_by": [redacted],

"searchable_quick": false,

"searchable_advanced": null,

"searchable_batch": null,

"searchable": "",

"pii": "No",

"qtip": "",

"used_by": "DNA Extraction",

"used_by_display": "<a href='#' onClick='remoteJS({name:\"dialogs/AddSubjectTypeDlg\", obj_id:122 });'>DNA Extraction</a>"

},

{

"id": 545,

"obj_id": 545,

"display_name": "Date Seen",

"name": "Date Seen",

"type": "Date",

"values": "",

"created_at": "05/07/2013",

"updated_at": "03/07/2014",

"created_by": [redacted],

"searchable_quick": true,

"searchable_advanced": true,

"searchable_batch": true,

"searchable": "Quick,Advanced,Batch",

"pii": "Yes",

"qtip": "",

"used_by": "Labmatrix Legacy - Peds Sharepoint Seen, Labmatrix Legacy - Peds Sharepoint Seen List, Sharepoint Legacy - Pediatric Patient, Sharepoint Legacy - Adult Patient Status, Patient Follow Up Visit, NIH Visit, Seen, Patient",

"used_by_display": "<a href='#' onClick='remoteJS({name:\"dialogs/AddSubjectTypeDlg\", obj_id:97 });'>Labmatrix Legacy - Peds Sharepoint Seen</a>, <a href='#' onClick='remoteJS({name:\"dialogs/AddSubjectTypeDlg\", obj_id:98 });'>Labmatrix Legacy - Peds Sharepoint Seen List</a>, <a href='#' onClick='remoteJS({name:\"dialogs/AddSubjectTypeDlg\", obj_id:114 });'>Sharepoint Legacy - Pediatric Patient</a>, <a href='#' onClick='remoteJS({name:\"dialogs/AddSubjectTypeDlg\", obj_id:115 });'>Sharepoint Legacy - Adult Patient Status</a>, <a href='#' onClick='remoteJS({name:\"dialogs/AddSubjectTypeDlg\", obj_id:264 });'>Patient Follow Up Visit</a>, <a href='#' onClick='remoteJS({name:\"dialogs/AddSubjectTypeDlg\", obj_id:140 });'>NIH Visit</a>, <a href='#' onClick='remoteJS({name:\"dialogs/AddSubjectTypeDlg\", obj_id:262 });'>Seen</a>, <a href='#' onClick='remoteJS({name:\"dialogs/AddSubjectTypeDlg\", obj_id:1 });'>Patient</a>"

},

{

"id": 121,

"obj_id": 121,

"display_name": "Date Sent",

"name": "Date Sent",

"type": "Date",

"values": "",

"created_at": "02/12/2013",

"updated_at": "07/19/2013",

"created_by": [redacted],

"searchable_quick": false,

"searchable_advanced": null,

"searchable_batch": null,

"searchable": "",

"pii": "Yes",

"qtip": "",

"used_by": "Information Technology Collaboration, NIH Visit, Transcriptome, ChIP-Seq, Alignment Collaboration Files",

"used_by_display": "<a href='#' onClick='remoteJS({name:\"dialogs/AddSubjectTypeDlg\", obj_id:183 });'>Information Technology Collaboration</a>, <a href='#' onClick='remoteJS({name:\"dialogs/AddSubjectTypeDlg\", obj_id:140 });'>NIH Visit</a>, <a href='#' onClick='remoteJS({name:\"dialogs/AddSubjectTypeDlg\", obj_id:255 });'>Transcriptome</a>, <a href='#' onClick='remoteJS({name:\"dialogs/AddSubjectTypeDlg\", obj_id:254 });'>ChIP-Seq</a>, <a href='#' onClick='remoteJS({name:\"dialogs/AddSubjectTypeDlg\", obj_id:194 });'>Alignment Collaboration Files</a>"

},

{

"id": 951,

"obj_id": 951,

"display_name": "Date sent 1st Review",

"name": "Date sent 1st Review",

"type": "Date",

"values": "",

"created_at": "05/10/2013",

"updated_at": "07/19/2013",

"created_by": [redacted],

"searchable_quick": false,

"searchable_advanced": null,

"searchable_batch": null,

"searchable": "",

"pii": "Yes",

"qtip": "",

"used_by": "Sharepoint Legacy - Adult Patient Status",

"used_by_display": "<a href='#' onClick='remoteJS({name:\"dialogs/AddSubjectTypeDlg\", obj_id:115 });'>Sharepoint Legacy - Adult Patient Status</a>"

},

{

"id": 1166,

"obj_id": 1166,

"display_name": "Date Sent for Sanger Validation",

"name": "Date Sent for Sanger Validation",

"type": "Date",

"values": "",

"created_at": "05/29/2013",

"updated_at": "09/19/2013",

"created_by": [redacted],

"searchable_quick": false,

"searchable_advanced": null,

"searchable_batch": null,

"searchable": "",

"pii": "No",

"qtip": "",

"used_by": "Sanger Interpretation",

"used_by_display": "<a href='#' onClick='remoteJS({name:\"dialogs/AddSubjectTypeDlg\", obj_id:22 });'>Sanger Interpretation</a>"

},

{

"id": 1711,

"obj_id": 1711,

"display_name": "Date Sent to Appistry",

"name": "Date Sent to Appistry",

"type": "Date",

"values": "",

"created_at": "09/26/2013",

"updated_at": "09/26/2013",

"created_by": [redacted],

"searchable_quick": false,

"searchable_advanced": null,

"searchable_batch": null,

"searchable": "",

"pii": "No",

"qtip": "",

"used_by": "",

"used_by_display": ""

},

{

"id": 1389,

"obj_id": 1389,

"display_name": "Date Sent to Collaborator",

"name": "Date Sent to Collaborator",

"type": "Date",

"values": "",

"created_at": "06/28/2013",

"updated_at": "10/29/2014",

"created_by": [redacted],

"searchable_quick": false,

"searchable_advanced": true,

"searchable_batch": false,

"searchable": "Advanced",

"pii": "No",

"qtip": "",

"used_by": "Exome Sequencing, Lymphoblast Culture Request, Overnight Collaboration Shipment",

"used_by_display": "<a href='#' onClick='remoteJS({name:\"dialogs/AddSubjectTypeDlg\", obj_id:161 });'>Exome Sequencing</a>, <a href='#' onClick='remoteJS({name:\"dialogs/AddSubjectTypeDlg\", obj_id:272 });'>Lymphoblast Culture Request</a>, <a href='#' onClick='remoteJS({name:\"dialogs/AddSubjectTypeDlg\", obj_id:273 });'>Overnight Collaboration Shipment</a>"

},

{

"id": 900,

"obj_id": 900,

"display_name": "Date Sequence Files Received",

"name": "Date Sequence Files Received",

"type": "Date",

"values": "",

"created_at": "05/10/2013",

"updated_at": "02/11/2014",

"created_by": [redacted],

"searchable_quick": false,

"searchable_advanced": null,

"searchable_batch": null,

"searchable": "",

"pii": "No",

"qtip": "",

"used_by": "Sanger Interpretation",

"used_by_display": "<a href='#' onClick='remoteJS({name:\"dialogs/AddSubjectTypeDlg\", obj_id:22 });'>Sanger Interpretation</a>"

},

{

"id": 1025,

"obj_id": 1025,

"display_name": "Date Serum Extracted",

"name": "Date Serum Extracted",

"type": "Date",

"values": "",

"created_at": "05/14/2013",

"updated_at": "09/19/2013",

"created_by": [redacted],

"searchable_quick": false,

"searchable_advanced": null,

"searchable_batch": null,

"searchable": "",

"pii": "No",

"qtip": "",

"used_by": "Serum Extraction",

"used_by_display": "<a href='#' onClick='remoteJS({name:\"dialogs/AddSubjectTypeDlg\", obj_id:119 });'>Serum Extraction</a>"

},

{

"id": 866,

"obj_id": 866,

"display_name": "Date SNP Analysis Complete",

"name": "Date SNP Analysis Complete",

"type": "Date",

"values": "",

"created_at": "05/08/2013",

"updated_at": "09/19/2013",

"created_by": [redacted],

"searchable_quick": false,

"searchable_advanced": null,

"searchable_batch": null,

"searchable": "",

"pii": "No",

"qtip": "",

"used_by": "SNP Analysis",

"used_by_display": "<a href='#' onClick='remoteJS({name:\"dialogs/AddSubjectTypeDlg\", obj_id:59 });'>SNP Analysis</a>"

},

{

"id": 444,

"obj_id": 444,

"display_name": "Date Sorted",

"name": "Date Sorted",

"type": "Date",

"values": "",

"created_at": "04/26/2013",

"updated_at": "07/19/2013",

"created_by": [redacted],

"searchable_quick": false,

"searchable_advanced": null,

"searchable_batch": null,

"searchable": "",

"pii": "Yes",

"qtip": "",

"used_by": "Patient Documents Scan & Sort",

"used_by_display": "<a href='#' onClick='remoteJS({name:\"dialogs/AddSubjectTypeDlg\", obj_id:88 });'>Patient Documents Scan & Sort</a>"

},

{

"id": 549,

"obj_id": 549,

"display_name": "Date_specimen_kit_FEDEXd",

"name": "Date_specimen_kit_FEDEXd",

"type": "Date",

"values": "",

"created_at": "05/07/2013",

"updated_at": "07/19/2013",

"created_by": [redacted],

"searchable_quick": false,

"searchable_advanced": null,

"searchable_batch": null,

"searchable": "",

"pii": "Yes",

"qtip": "",

"used_by": "Labmatrix Legacy - FEDEX Specimen Kit Tracking",

"used_by_display": "<a href='#' onClick='remoteJS({name:\"dialogs/AddSubjectTypeDlg\", obj_id:103 });'>Labmatrix Legacy - FEDEX Specimen Kit Tracking</a>"

},

{

"id": 550,

"obj_id": 550,

"display_name": "Date_specimen_kit_received_from_Subject",

"name": "Date_specimen_kit_received_from_Subject",

"type": "Date",

"values": "",

"created_at": "05/07/2013",

"updated_at": "07/19/2013",

"created_by": [redacted],

"searchable_quick": false,

"searchable_advanced": null,

"searchable_batch": null,

"searchable": "",

"pii": "Yes",

"qtip": "",

"used_by": "Labmatrix Legacy - FEDEX Specimen Kit Tracking",

"used_by_display": "<a href='#' onClick='remoteJS({name:\"dialogs/AddSubjectTypeDlg\", obj_id:103 });'>Labmatrix Legacy - FEDEX Specimen Kit Tracking</a>"

},

{

"id": 83,

"obj_id": 83,

"display_name": "Date Specimens Received",

"name": "Date Specimens Received",

"type": "Date",

"values": "",

"created_at": "02/06/2013",

"updated_at": "07/19/2013",

"created_by": [redacted],

"searchable_quick": false,

"searchable_advanced": null,

"searchable_batch": null,

"searchable": "",

"pii": "Yes",

"qtip": "",

"used_by": "DNA Extraction",

"used_by_display": "<a href='#' onClick='remoteJS({name:\"dialogs/AddSubjectTypeDlg\", obj_id:122 });'>DNA Extraction</a>"

},

{

"id": 1260,

"obj_id": 1260,

"display_name": "Date Specimen to be collected",

"name": "Date Specimen to be collected",

"type": "Date",

"values": "",

"created_at": "06/03/2013",

"updated_at": "07/19/2013",

"created_by": [redacted],

"searchable_quick": false,

"searchable_advanced": null,

"searchable_batch": null,

"searchable": "",

"pii": "Yes",

"qtip": "",

"used_by": "NIH Visit",

"used_by_display": "<a href='#' onClick='remoteJS({name:\"dialogs/AddSubjectTypeDlg\", obj_id:140 });'>NIH Visit</a>"

},

{

"id": 2788,

"obj_id": 2788,

"display_name": "Date(s) Seen",

"name": "Date(s) Seen",

"type": "Subject Back Ref",

"values": "",

"created_at": "08/20/2014",

"updated_at": "08/20/2014",

"created_by": [redacted],

"searchable_quick": false,

"searchable_advanced": false,

"searchable_batch": false,

"searchable": "",

"pii": "No",

"qtip": null,

"used_by": "Patient Follow Up Visit",

"used_by_display": "<a href='#' onClick='remoteJS({name:\"dialogs/AddSubjectTypeDlg\", obj_id:264 });'>Patient Follow Up Visit</a>"

},

{

"id": 67,

"obj_id": 67,

"display_name": "Date Started",

"name": "Date Started",

"type": "Date",

"values": "",

"created_at": "02/06/2013",

"updated_at": "11/13/2014",

"created_by": [redacted],

"searchable_quick": false,

"searchable_advanced": false,

"searchable_batch": false,

"searchable": "",

"pii": "No",

"qtip": "",

"used_by": "iPS Cells, Assay, Invoice Information",

"used_by_display": "<a href='#' onClick='remoteJS({name:\"dialogs/AddSubjectTypeDlg\", obj_id:41 });'>iPS Cells</a>, <a href='#' onClick='remoteJS({name:\"dialogs/AddSubjectTypeDlg\", obj_id:225 });'>Assay</a>, <a href='#' onClick='remoteJS({name:\"dialogs/AddSubjectTypeDlg\", obj_id:270 });'>Invoice Information</a>"

},

{

"id": 1099,

"obj_id": 1099,

"display_name": "Date Submitted to dbGaP",

"name": "Date Submitted to dbGaP",

"type": "Date",

"values": "",

"created_at": "05/17/2013",

"updated_at": "03/04/2014",

"created_by": [redacted],

"searchable_quick": false,

"searchable_advanced": null,

"searchable_batch": null,

"searchable": "",

"pii": "No",

"qtip": "",

"used_by": "dbGaP Submission",

"used_by_display": "<a href='#' onClick='remoteJS({name:\"dialogs/AddSubjectTypeDlg\", obj_id:213 });'>dbGaP Submission</a>"

},

{

"id": 2376,

"obj_id": 2376,

"display_name": "Date Submitted to PhenomeCentral",

"name": "Date Submitted to PhenomeCentral",

"type": "Date",

"values": "",

"created_at": "03/10/2014",

"updated_at": "03/10/2014",

"created_by": [redacted],

"searchable_quick": false,

"searchable_advanced": null,

"searchable_batch": null,

"searchable": "",

"pii": "No",

"qtip": "",

"used_by": "PhenomeCentral Data Submission",

"used_by_display": "<a href='#' onClick='remoteJS({name:\"dialogs/AddSubjectTypeDlg\", obj_id:216 });'>PhenomeCentral Data Submission</a>"

},

{

"id": 457,

"obj_id": 457,

"display_name": "Date Treatment Started",

"name": "Date Treatment Started",

"type": "Date",

"values": "",

"created_at": "04/28/2013",

"updated_at": "08/26/2013",

"created_by": [redacted],

"searchable_quick": false,

"searchable_advanced": null,

"searchable_batch": null,

"searchable": "",

"pii": "No",

"qtip": "",

"used_by": "Cell Culture, Rescue Cell Culture",

"used_by_display": "<a href='#' onClick='remoteJS({name:\"dialogs/AddSubjectTypeDlg\", obj_id:40 });'>Cell Culture</a>, <a href='#' onClick='remoteJS({name:\"dialogs/AddSubjectTypeDlg\", obj_id:247 });'>Rescue Cell Culture</a>"

},

{

"id": 1940,

"obj_id": 1940,

"display_name": "Date Trypsinized",

"name": "Date Trypsinized",

"type": "Date",

"values": "",

"created_at": "11/13/2013",

"updated_at": "11/13/2013",

"created_by": [redacted],

"searchable_quick": false,

"searchable_advanced": null,

"searchable_batch": null,

"searchable": "",

"pii": "No",

"qtip": "",

"used_by": "iPS Cells",

"used_by_display": "<a href='#' onClick='remoteJS({name:\"dialogs/AddSubjectTypeDlg\", obj_id:41 });'>iPS Cells</a>"

},

{

"id": 1033,

"obj_id": 1033,

"display_name": "Date Urine Processed",

"name": "Date Urine Processed",

"type": "Date",

"values": "",

"created_at": "05/14/2013",

"updated_at": "07/19/2013",

"created_by": [redacted],

"searchable_quick": false,

"searchable_advanced": null,

"searchable_batch": null,

"searchable": "",

"pii": "Yes",

"qtip": "",

"used_by": "Urine",

"used_by_display": "<a href='#' onClick='remoteJS({name:\"dialogs/AddSubjectTypeDlg\", obj_id:120 });'>Urine</a>"

},

{

"id": 2498,

"obj_id": 2498,

"display_name": "dbGaP BAM File",

"name": "dbGaP BAM File",

"type": "Subject",

"values": "",

"created_at": "04/09/2014",

"updated_at": "04/09/2014",

"created_by": [redacted],

"searchable_quick": false,

"searchable_advanced": null,

"searchable_batch": null,

"searchable": "",

"pii": "No",

"qtip": "",

"used_by": "dbGaP Submission",

"used_by_display": "<a href='#' onClick='remoteJS({name:\"dialogs/AddSubjectTypeDlg\", obj_id:213 });'>dbGaP Submission</a>"

},

{

"id": 2199,

"obj_id": 2199,

"display_name": "dbGAP ID",

"name": "dbGAP ID",

"type": "Formula",

"values": "",

"created_at": "01/30/2014",

"updated_at": "01/30/2014",

"created_by": [redacted],

"searchable_quick": false,

"searchable_advanced": null,

"searchable_batch": null,

"searchable": "",

"pii": "No",

"qtip": null,

"used_by": "Patient, Patient Follow Up Visit",

"used_by_display": "<a href='#' onClick='remoteJS({name:\"dialogs/AddSubjectTypeDlg\", obj_id:1 });'>Patient</a>, <a href='#' onClick='remoteJS({name:\"dialogs/AddSubjectTypeDlg\", obj_id:264 });'>Patient Follow Up Visit</a>"

},

{

"id": 2286,

"obj_id": 2286,

"display_name": "dbGaP VCF",

"name": "dbGaP VCF",

"type": "Subject",

"values": "",

"created_at": "02/20/2014",

"updated_at": "02/20/2014",

"created_by": [redacted],

"searchable_quick": true,

"searchable_advanced": true,

"searchable_batch": true,

"searchable": "Quick,Advanced,Batch",

"pii": "No",

"qtip": "",

"used_by": "dbGaP Submission",

"used_by_display": "<a href='#' onClick='remoteJS({name:\"dialogs/AddSubjectTypeDlg\", obj_id:213 });'>dbGaP Submission</a>"

},

{

"id": 2166,

"obj_id": 2166,

"display_name": "dbSNP ID",

"name": "dbSNP ID",

"type": "Text Field",

"values": "",

"created_at": "01/17/2014",

"updated_at": "04/21/2014",

"created_by": [redacted],

"searchable_quick": false,

"searchable_advanced": null,

"searchable_batch": null,

"searchable": "",

"pii": "No",

"qtip": "",

"used_by": "Exome Analysis Results",

"used_by_display": "<a href='#' onClick='remoteJS({name:\"dialogs/AddSubjectTypeDlg\", obj_id:156 });'>Exome Analysis Results</a>"

},

{

"id": 1881,

"obj_id": 1881,

"display_name": "dbSNP_MAF",

"name": "dbSNP_MAF",

"type": "Text Field",

"values": "",

"created_at": "11/07/2013",

"updated_at": "11/07/2013",

"created_by": [redacted],

"searchable_quick": false,

"searchable_advanced": null,

"searchable_batch": null,

"searchable": "",

"pii": "No",

"qtip": "",

"used_by": "Exome Analysis Results",

"used_by_display": "<a href='#' onClick='remoteJS({name:\"dialogs/AddSubjectTypeDlg\", obj_id:156 });'>Exome Analysis Results</a>"

},

{

"id": 2331,

"obj_id": 2331,

"display_name": "DDx:",

"name": "DDx:",

"type": "Text Area",

"values": "",

"created_at": "03/04/2014",

"updated_at": "03/04/2014",

"created_by": [redacted],

"searchable_quick": false,

"searchable_advanced": null,

"searchable_batch": null,

"searchable": "",

"pii": "Yes",

"qtip": "",

"used_by": "Patient Review",

"used_by_display": "<a href='#' onClick='remoteJS({name:\"dialogs/AddSubjectTypeDlg\", obj_id:83 });'>Patient Review</a>"

},

{

"id": 1651,

"obj_id": 1651,

"display_name": "Decreased m/z",

"name": "Decreased m/z",

"type": "Text Field",

"values": "",

"created_at": "08/12/2013",

"updated_at": "10/28/2014",

"created_by": [redacted],

"searchable_quick": false,

"searchable_advanced": true,

"searchable_batch": false,

"searchable": "Advanced",

"pii": "No",

"qtip": "",

"used_by": "Glycomics Results",

"used_by_display": "<a href='#' onClick='remoteJS({name:\"dialogs/AddSubjectTypeDlg\", obj_id:176 });'>Glycomics Results</a>"

},

{

"id": 2178,

"obj_id": 2178,

"display_name": "Deleterious Changes for Whole Locus",

"name": "Deleterious Changes for Whole Locus",

"type": "Text Field",

"values": "",

"created_at": "01/17/2014",

"updated_at": "01/17/2014",

"created_by": [redacted],

"searchable_quick": false,

"searchable_advanced": null,

"searchable_batch": null,

"searchable": "",

"pii": "No",

"qtip": "",

"used_by": "Exome Analysis Results",

"used_by_display": "<a href='#' onClick='remoteJS({name:\"dialogs/AddSubjectTypeDlg\", obj_id:156 });'>Exome Analysis Results</a>"

},

{

"id": 1488,

"obj_id": 1488,

"display_name": "DELETE - Sample(s)",

"name": "DELETE - Sample(s)",

"type": "Text Field",

"values": "",

"created_at": "07/23/2013",

"updated_at": "06/06/2014",

"created_by": [redacted],

"searchable_quick": false,

"searchable_advanced": null,

"searchable_batch": null,

"searchable": "",

"pii": "No",

"qtip": "",

"used_by": "",

"used_by_display": ""

},

{

"id": 1403,

"obj_id": 1403,

"display_name": "DELETE - Subject of Collaborations",

"name": "DELETE - Subject of Collaborations",

"type": "Choice",

"values": "Fibroblast pellet glycomics, Zebrafish study, Yeast interactome study, Drosophila study, Omics testing, Other",

"created_at": "07/08/2013",

"updated_at": "06/06/2014",

"created_by": [redacted],

"searchable_quick": false,

"searchable_advanced": null,

"searchable_batch": null,

"searchable": "",

"pii": "No",

"qtip": "",

"used_by": "",

"used_by_display": ""

},

{

"id": 1415,

"obj_id": 1415,

"display_name": "DELETE - Unknown Significance - Does Not Segregate",

"name": "DELETE - Unknown Significance - Does Not Segregate",

"type": "Text Area",

"values": "",

"created_at": "07/08/2013",

"updated_at": "06/06/2014",

"created_by": [redacted],

"searchable_quick": false,

"searchable_advanced": null,

"searchable_batch": null,

"searchable": "",

"pii": "No",

"qtip": "Min 3 SNPs; does not segregate with disease; does not qualify for reported or unreported pathogenic",

"used_by": "",

"used_by_display": ""

},

{

"id": 1413,

"obj_id": 1413,

"display_name": "DELETE - Unknown Significance - Segregates",

"name": "DELETE - Unknown Significance - Segregates",

"type": "Text Area",

"values": "",

"created_at": "07/08/2013",

"updated_at": "06/06/2014",

"created_by": [redacted],

"searchable_quick": false,

"searchable_advanced": null,

"searchable_batch": null,

"searchable": "",

"pii": "No",

"qtip": "Min 3 SNPs; segregates with disease; does not qualify for reported or unreported pathogenic",

"used_by": "",

"used_by_display": ""

},

{

"id": 1412,

"obj_id": 1412,

"display_name": "DELETE - Unreported Possibly Pathogenic Regions",

"name": "DELETE - Unreported Possibly Pathogenic Regions",

"type": "Text Area",

"values": "",

"created_at": "07/08/2013",

"updated_at": "06/06/2014",

"created_by": [redacted],

"searchable_quick": false,

"searchable_advanced": null,

"searchable_batch": null,

"searchable": "",

"pii": "No",

"qtip": "Min 3 SNPs; within coding region or within 5000bp of a coding region; segregates with disease; not reported pathogenic in OMIM or DECIPHER",

"used_by": "",

"used_by_display": ""

},

{

"id": 1424,

"obj_id": 1424,

"display_name": "DELETE - UNUSED Age of onset",

"name": "DELETE - UNUSED Age of onset",

"type": "Radio Buttons",

"values": "Unknown, Congenital onset, Embryonal onset, Fetal onset, Neonatal onset, Infantile onset, Childhood onset, Juvenile onset, Adult onset, Young adult onset, Middle age onset, Late onset",

"created_at": "07/10/2013",

"updated_at": "02/20/2014",

"created_by": [redacted],

"searchable_quick": false,

"searchable_advanced": null,

"searchable_batch": null,

"searchable": "",

"pii": "No",

"qtip": "",

"used_by": "",

"used_by_display": ""

},

{

"id": 2241,

"obj_id": 2241,

"display_name": "DELETE - UNUSED Diploid Align Family Members",

"name": "DELETE - UNUSED Diploid Align Family Members",

"type": "Text Field",

"values": "",

"created_at": "02/10/2014",

"updated_at": "02/26/2014",

"created_by": [redacted],

"searchable_quick": false,

"searchable_advanced": null,

"searchable_batch": null,

"searchable": "",

"pii": "No",

"qtip": "Placeholder field -- list family members to be DA",

"used_by": "",

"used_by_display": ""

},

{

"id": 2205,

"obj_id": 2205,

"display_name": "DELETE - UNUSED Global Age of Onset",

"name": "DELETE - UNUSED Global Age of Onset",

"type": "Text Field",

"values": "",

"created_at": "02/04/2014",

"updated_at": "02/20/2014",

"created_by": [redacted],

"searchable_quick": false,

"searchable_advanced": null,

"searchable_batch": null,

"searchable": "",

"pii": "No",

"qtip": "",

"used_by": "",

"used_by_display": ""

},

{

"id": 2242,

"obj_id": 2242,

"display_name": "DELETE - UNUSED Iteration 0 Family Members",

"name": "DELETE - UNUSED Iteration 0 Family Members",

"type": "Text Field",

"values": "",

"created_at": "02/10/2014",

"updated_at": "02/26/2014",

"created_by": [redacted],

"searchable_quick": false,

"searchable_advanced": null,

"searchable_batch": null,

"searchable": "",

"pii": "No",

"qtip": "Placeholder field -- list family members for It0",

"used_by": "",

"used_by_display": ""

},

{

"id": 2186,

"obj_id": 2186,

"display_name": "Deletion Bedfile",

"name": "Deletion Bedfile",

"type": "File",

"values": "",

"created_at": "01/24/2014",

"updated_at": "01/24/2014",

"created_by": [redacted],

"searchable_quick": false,

"searchable_advanced": null,

"searchable_batch": null,

"searchable": "",

"pii": "No",

"qtip": "",

"used_by": "SNP Analysis",

"used_by_display": "<a href='#' onClick='remoteJS({name:\"dialogs/AddSubjectTypeDlg\", obj_id:59 });'>SNP Analysis</a>"

},

{

"id": 1214,

"obj_id": 1214,

"display_name": "Deletion Bedfile Created",

"name": "Deletion Bedfile Created",

"type": "CheckBox",

"values": "",

"created_at": "05/29/2013",

"updated_at": "09/19/2013",

"created_by": [redacted],

"searchable_quick": false,

"searchable_advanced": null,

"searchable_batch": null,

"searchable": "",

"pii": "No",

"qtip": "",

"used_by": "SNP Analysis",

"used_by_display": "<a href='#' onClick='remoteJS({name:\"dialogs/AddSubjectTypeDlg\", obj_id:59 });'>SNP Analysis</a>"

},

{

"id": 2889,

"obj_id": 2889,

"display_name": "Deletion BED file filtered Exomiser Output file",

"name": "Deletion BED file filtered Exomiser Output file",

"type": "File",

"values": "",

"created_at": "11/12/2014",

"updated_at": "11/12/2014",

"created_by": [redacted],

"searchable_quick": false,

"searchable_advanced": true,

"searchable_batch": false,

"searchable": "Advanced",

"pii": "No",

"qtip": "",

"used_by": "Exome Analysis",

"used_by_display": "<a href='#' onClick='remoteJS({name:\"dialogs/AddSubjectTypeDlg\", obj_id:60 });'>Exome Analysis</a>"

},

{

"id": 2881,

"obj_id": 2881,

"display_name": "Deletion BED file filtered Exomiser Output File",

"name": "Deletion BED file filtered Exomiser Output File",

"type": "Text Field",

"values": "",

"created_at": "11/11/2014",

"updated_at": "11/11/2014",

"created_by": [redacted],

"searchable_quick": false,

"searchable_advanced": true,

"searchable_batch": false,

"searchable": "Advanced",

"pii": "No",

"qtip": "",

"used_by": "",

"used_by_display": ""

},

{

"id": 1584,

"obj_id": 1584,

"display_name": "Demographic Information obtained for Proband/Family",

"name": "Demographic Information obtained for Proband/Family",

"type": "Radio Buttons",

"values": "Yes, No",

"created_at": "08/12/2013",

"updated_at": "08/12/2013",

"created_by": [redacted],

"searchable_quick": false,

"searchable_advanced": null,

"searchable_batch": null,

"searchable": "",

"pii": "No",

"qtip": "",

"used_by": "Patient Visit Tools, Patient Visit Planning for Version C",

"used_by_display": "<a href='#' onClick='remoteJS({name:\"dialogs/AddSubjectTypeDlg\", obj_id:163 });'>Patient Visit Tools</a>, <a href='#' onClick='remoteJS({name:\"dialogs/AddSubjectTypeDlg\", obj_id:192 });'>Patient Visit Planning for Version C</a>"

},

{

"id": 383,

"obj_id": 383,

"display_name": "De Novo",

"name": "De Novo",

"type": "CheckBox",

"values": "",

"created_at": "04/15/2013",

"updated_at": "06/06/2014",

"created_by": [redacted],

"searchable_quick": false,

"searchable_advanced": null,

"searchable_batch": null,

"searchable": "",

"pii": "No",

"qtip": "Boolean filter",

"used_by": "Exome Analysis",

"used_by_display": "<a href='#' onClick='remoteJS({name:\"dialogs/AddSubjectTypeDlg\", obj_id:60 });'>Exome Analysis</a>"

},

{

"id": 2886,

"obj_id": 2886,

"display_name": "De Novo Exomiser Output file",

"name": "De Novo Exomiser Output file",

"type": "File",

"values": "",

"created_at": "11/12/2014",

"updated_at": "11/12/2014",

"created_by": [redacted],

"searchable_quick": false,

"searchable_advanced": true,

"searchable_batch": false,

"searchable": "Advanced",

"pii": "No",

"qtip": "",

"used_by": "Exome Analysis",

"used_by_display": "<a href='#' onClick='remoteJS({name:\"dialogs/AddSubjectTypeDlg\", obj_id:60 });'>Exome Analysis</a>"

},

{

"id": 2878,

"obj_id": 2878,

"display_name": "De Novo Exomiser Output File",

"name": "De Novo Exomiser Output File",

"type": "Text Field",

"values": "",

"created_at": "11/11/2014",

"updated_at": "11/11/2014",

"created_by": [redacted],

"searchable_quick": false,

"searchable_advanced": true,

"searchable_batch": false,

"searchable": "Advanced",

"pii": "No",

"qtip": "",

"used_by": "",

"used_by_display": ""

},

{

"id": 1656,

"obj_id": 1656,

"display_name": "Description",

"name": "Description",

"type": "Text Area",

"values": "",

"created_at": "08/14/2013",

"updated_at": "01/23/2014",

"created_by": [redacted],

"searchable_quick": false,

"searchable_advanced": null,

"searchable_batch": null,

"searchable": "",

"pii": "No",

"qtip": "",

"used_by": "Cohort Variant Call Format File, Non - PII Patient File Uploads, Destination Vector Construction, Entry Vector, Information Technology Collaboration Files, Vial, PII - Patient File Uploads, Bioinformatics Hard Drives, Clone, Glycerol Stock, Alignment Collaboration Files",

"used_by_display": "<a href='#' onClick='remoteJS({name:\"dialogs/AddSubjectTypeDlg\", obj_id:195 });'>Cohort Variant Call Format File</a>, <a href='#' onClick='remoteJS({name:\"dialogs/AddSubjectTypeDlg\", obj_id:201 });'>Non - PII Patient File Uploads</a>, <a href='#' onClick='remoteJS({name:\"dialogs/AddSubjectTypeDlg\", obj_id:168 });'>Destination Vector Construction</a>, <a href='#' onClick='remoteJS({name:\"dialogs/AddSubjectTypeDlg\", obj_id:193 });'>Entry Vector</a>, <a href='#' onClick='remoteJS({name:\"dialogs/AddSubjectTypeDlg\", obj_id:184 });'>Information Technology Collaboration Files</a>, <a href='#' onClick='remoteJS({name:\"dialogs/AddSubjectTypeDlg\", obj_id:152 });'>Vial</a>, <a href='#' onClick='remoteJS({name:\"dialogs/AddSubjectTypeDlg\", obj_id:172 });'>PII - Patient File Uploads</a>, <a href='#' onClick='remoteJS({name:\"dialogs/AddSubjectTypeDlg\", obj_id:248 });'>Bioinformatics Hard Drives</a>, <a href='#' onClick='remoteJS({name:\"dialogs/AddSubjectTypeDlg\", obj_id:266 });'>Clone</a>, <a href='#' onClick='remoteJS({name:\"dialogs/AddSubjectTypeDlg\", obj_id:171 });'>Glycerol Stock</a>, <a href='#' onClick='remoteJS({name:\"dialogs/AddSubjectTypeDlg\", obj_id:194 });'>Alignment Collaboration Files</a>"

},

{

"id": 2136,

"obj_id": 2136,

"display_name": "Description of Data or Materials to be Transferred",

"name": "Description of Data or Materials to be Transferred",

"type": "Text Area",

"values": "",

"created_at": "01/13/2014",

"updated_at": "12/18/2014",

"created_by": [redacted],

"searchable_quick": false,

"searchable_advanced": false,

"searchable_batch": false,

"searchable": "",

"pii": "No",

"qtip": "Details about what is being collaborated",

"used_by": "Collaborations, Collaboration Projects",

"used_by_display": "<a href='#' onClick='remoteJS({name:\"dialogs/AddSubjectTypeDlg\", obj_id:147 });'>Collaborations</a>, <a href='#' onClick='remoteJS({name:\"dialogs/AddSubjectTypeDlg\", obj_id:204 });'>Collaboration Projects</a>"

},

{

"id": 1236,

"obj_id": 1236,

"display_name": "Description of issue",

"name": "Description of issue",

"type": "Text Area",

"values": "",

"created_at": "06/03/2013",

"updated_at": "06/10/2014",

"created_by": [redacted],

"searchable_quick": false,

"searchable_advanced": null,

"searchable_batch": null,

"searchable": "",

"pii": "Yes",

"qtip": "",

"used_by": "NIH Visit, Patient Visit Tools, Patient Visit Planning for Version C",

"used_by_display": "<a href='#' onClick='remoteJS({name:\"dialogs/AddSubjectTypeDlg\", obj_id:140 });'>NIH Visit</a>, <a href='#' onClick='remoteJS({name:\"dialogs/AddSubjectTypeDlg\", obj_id:163 });'>Patient Visit Tools</a>, <a href='#' onClick='remoteJS({name:\"dialogs/AddSubjectTypeDlg\", obj_id:192 });'>Patient Visit Planning for Version C</a>"

},

{

"id": 314,

"obj_id": 314,

"display_name": "Destination Address - DELETE",

"name": "Destination Address - DELETE",

"type": "Text Area",

"values": "",

"created_at": "04/05/2013",

"updated_at": "06/09/2014",

"created_by": [redacted],

"searchable_quick": false,

"searchable_advanced": null,

"searchable_batch": null,

"searchable": "",

"pii": "No",

"qtip": "Address where specimen is being shipped",

"used_by": "",

"used_by_display": ""

},

{

"id": 1559,

"obj_id": 1559,

"display_name": "Destination_Lab_or_Researcher",

"name": "Destination_Lab_or_Researcher",

"type": "Text Field",

"values": "",

"created_at": "08/09/2013",

"updated_at": "08/09/2013",

"created_by": [redacted],

"searchable_quick": false,

"searchable_advanced": null,

"searchable_batch": null,

"searchable": "",

"pii": "No",

"qtip": "",

"used_by": "Labmatrix Legacy - Send Out Tests",

"used_by_display": "<a href='#' onClick='remoteJS({name:\"dialogs/AddSubjectTypeDlg\", obj_id:104 });'>Labmatrix Legacy - Send Out Tests</a>"

},

{

"id": 1925,

"obj_id": 1925,

"display_name": "Destination Vector",

"name": "Destination Vector",

"type": "Subject",

"values": "",

"created_at": "11/11/2013",

"updated_at": "01/13/2015",

"created_by": [redacted],

"searchable_quick": false,

"searchable_advanced": false,

"searchable_batch": false,

"searchable": "",

"pii": "No",

"qtip": "",

"used_by": "Destination Vector Construction, Mutagenized Vectors",

"used_by_display": "<a href='#' onClick='remoteJS({name:\"dialogs/AddSubjectTypeDlg\", obj_id:168 });'>Destination Vector Construction</a>, <a href='#' onClick='remoteJS({name:\"dialogs/AddSubjectTypeDlg\", obj_id:228 });'>Mutagenized Vectors</a>"

},

{

"id": 1928,

"obj_id": 1928,

"display_name": "Destination Vector Confirmed by Macrogen Sequencing",

"name": "Destination Vector Confirmed by Macrogen Sequencing",

"type": "CheckBox",

"values": "",

"created_at": "11/11/2013",

"updated_at": "11/11/2013",

"created_by": [redacted],

"searchable_quick": false,

"searchable_advanced": null,

"searchable_batch": null,

"searchable": "",

"pii": "No",

"qtip": "",

"used_by": "Destination Vector Construction",

"used_by_display": "<a href='#' onClick='remoteJS({name:\"dialogs/AddSubjectTypeDlg\", obj_id:168 });'>Destination Vector Construction</a>"

},

{

"id": 2251,

"obj_id": 2251,

"display_name": "Detection of Mutation in F1 Embryos",

"name": "Detection of Mutation in F1 Embryos",

"type": "Choice",

"values": "Yes, No",

"created_at": "02/10/2014",

"updated_at": "03/13/2014",

"created_by": [redacted],

"searchable_quick": false,

"searchable_advanced": null,

"searchable_batch": null,

"searchable": "",

"pii": "No",

"qtip": "",

"used_by": "Zebrafish Mutation Project, Zebrafish Breeding Information",

"used_by_display": "<a href='#' onClick='remoteJS({name:\"dialogs/AddSubjectTypeDlg\", obj_id:186 });'>Zebrafish Mutation Project</a>, <a href='#' onClick='remoteJS({name:\"dialogs/AddSubjectTypeDlg\", obj_id:233 });'>Zebrafish Breeding Information</a>"

},

{

"id": 1878,

"obj_id": 1878,

"display_name": "Diagnoses",

"name": "Diagnoses",

"type": "Subject Back Ref",

"values": "",

"created_at": "11/07/2013",

"updated_at": "11/24/2014",

"created_by": [redacted],

"searchable_quick": true,

"searchable_advanced": true,

"searchable_batch": true,

"searchable": "Quick,Advanced,Batch",

"pii": "No",

"qtip": "",

"used_by": "Patient, Patient Review, UCSC Gene List, Patient Follow Up Visit",

"used_by_display": "<a href='#' onClick='remoteJS({name:\"dialogs/AddSubjectTypeDlg\", obj_id:1 });'>Patient</a>, <a href='#' onClick='remoteJS({name:\"dialogs/AddSubjectTypeDlg\", obj_id:83 });'>Patient Review</a>, <a href='#' onClick='remoteJS({name:\"dialogs/AddSubjectTypeDlg\", obj_id:199 });'>UCSC Gene List</a>, <a href='#' onClick='remoteJS({name:\"dialogs/AddSubjectTypeDlg\", obj_id:264 });'>Patient Follow Up Visit</a>"

},

{

"id": 552,

"obj_id": 552,

"display_name": "Diagnosis",

"name": "Diagnosis",

"type": "Text Area",

"values": "",

"created_at": "05/07/2013",

"updated_at": "11/13/2014",

"created_by": [redacted],

"searchable_quick": false,

"searchable_advanced": false,

"searchable_batch": false,

"searchable": "",

"pii": "Yes",

"qtip": "",

"used_by": "Labmatrix Legacy - Peds Sharepoint Seen, Labmatrix Legacy - Peds Sharepoint Seen List, Sharepoint Legacy - Pediatric Patient, Sharepoint Legacy - Adult Patient Status",

"used_by_display": "<a href='#' onClick='remoteJS({name:\"dialogs/AddSubjectTypeDlg\", obj_id:97 });'>Labmatrix Legacy - Peds Sharepoint Seen</a>, <a href='#' onClick='remoteJS({name:\"dialogs/AddSubjectTypeDlg\", obj_id:98 });'>Labmatrix Legacy - Peds Sharepoint Seen List</a>, <a href='#' onClick='remoteJS({name:\"dialogs/AddSubjectTypeDlg\", obj_id:114 });'>Sharepoint Legacy - Pediatric Patient</a>, <a href='#' onClick='remoteJS({name:\"dialogs/AddSubjectTypeDlg\", obj_id:115 });'>Sharepoint Legacy - Adult Patient Status</a>"

},

{

"id": 2710,

"obj_id": 2710,

"display_name": "Diagnosis Disproved",

"name": "Diagnosis Disproved",

"type": "Choice",

"values": "Yes, No",

"created_at": "07/17/2014",

"updated_at": "11/13/2014",

"created_by": [redacted],

"searchable_quick": false,

"searchable_advanced": true,

"searchable_batch": false,

"searchable": "Advanced",

"pii": "No",

"qtip": "",

"used_by": "Diagnosis",

"used_by_display": "<a href='#' onClick='remoteJS({name:\"dialogs/AddSubjectTypeDlg\", obj_id:185 });'>Diagnosis</a>"

},

{

"id": 1875,

"obj_id": 1875,

"display_name": "Diagnosis Name",

"name": "Diagnosis Name",

"type": "Text Field",

"values": "",

"created_at": "11/07/2013",

"updated_at": "11/13/2014",

"created_by": [redacted],

"searchable_quick": false,

"searchable_advanced": true,

"searchable_batch": false,

"searchable": "Advanced",

"pii": "No",

"qtip": "",

"used_by": "Diagnosis",

"used_by_display": "<a href='#' onClick='remoteJS({name:\"dialogs/AddSubjectTypeDlg\", obj_id:185 });'>Diagnosis</a>"

},

{

"id": 2579,

"obj_id": 2579,

"display_name": "Dietary therapy",

"name": "Dietary therapy",

"type": "Choice",

"values": "Yes, No",

"created_at": "05/01/2014",

"updated_at": "05/01/2014",

"created_by": [redacted],

"searchable_quick": false,

"searchable_advanced": null,

"searchable_batch": null,

"searchable": "",

"pii": "No",

"qtip": "",

"used_by": "Consent Versions",

"used_by_display": "<a href='#' onClick='remoteJS({name:\"dialogs/AddSubjectTypeDlg\", obj_id:239 });'>Consent Versions</a>"

},

{

"id": 1914,

"obj_id": 1914,

"display_name": "Dilution for ELISA",

"name": "Dilution for ELISA",

"type": "Text Field",

"values": "",

"created_at": "11/08/2013",

"updated_at": "11/08/2013",

"created_by": [redacted],

"searchable_quick": false,

"searchable_advanced": null,

"searchable_batch": null,

"searchable": "",

"pii": "No",

"qtip": "",

"used_by": "Antibody",

"used_by_display": "<a href='#' onClick='remoteJS({name:\"dialogs/AddSubjectTypeDlg\", obj_id:189 });'>Antibody</a>"

},

{

"id": 1912,

"obj_id": 1912,

"display_name": "Dilution for Immuno Cytochemistry",

"name": "Dilution for Immuno Cytochemistry",

"type": "Text Field",

"values": "",

"created_at": "11/08/2013",

"updated_at": "11/08/2013",

"created_by": [redacted],

"searchable_quick": false,

"searchable_advanced": null,

"searchable_batch": null,

"searchable": "",

"pii": "No",

"qtip": "",

"used_by": "Antibody",

"used_by_display": "<a href='#' onClick='remoteJS({name:\"dialogs/AddSubjectTypeDlg\", obj_id:189 });'>Antibody</a>"

},

{

"id": 1911,

"obj_id": 1911,

"display_name": "Dilution for Immuno Histochemistry",

"name": "Dilution for Immuno Histochemistry",

"type": "Text Field",

"values": "",

"created_at": "11/08/2013",

"updated_at": "11/08/2013",

"created_by": [redacted],

"searchable_quick": false,

"searchable_advanced": null,

"searchable_batch": null,

"searchable": "",

"pii": "No",

"qtip": "",

"used_by": "Antibody",

"used_by_display": "<a href='#' onClick='remoteJS({name:\"dialogs/AddSubjectTypeDlg\", obj_id:189 });'>Antibody</a>"

},

{

"id": 1913,

"obj_id": 1913,

"display_name": "Dilution for IP",

"name": "Dilution for IP",

"type": "Text Field",

"values": "",

"created_at": "11/08/2013",

"updated_at": "11/08/2013",

"created_by": [redacted],

"searchable_quick": false,

"searchable_advanced": null,

"searchable_batch": null,

"searchable": "",

"pii": "No",

"qtip": "",

"used_by": "Antibody",

"used_by_display": "<a href='#' onClick='remoteJS({name:\"dialogs/AddSubjectTypeDlg\", obj_id:189 });'>Antibody</a>"

},

{

"id": 1910,

"obj_id": 1910,

"display_name": "Dilution for Western Blot",

"name": "Dilution for Western Blot",

"type": "Text Field",

"values": "",

"created_at": "11/08/2013",

"updated_at": "11/08/2013",

"created_by": [redacted],

"searchable_quick": false,

"searchable_advanced": null,

"searchable_batch": null,

"searchable": "",

"pii": "No",

"qtip": "",

"used_by": "Antibody",

"used_by_display": "<a href='#' onClick='remoteJS({name:\"dialogs/AddSubjectTypeDlg\", obj_id:189 });'>Antibody</a>"

},

{

"id": 2239,

"obj_id": 2239,

"display_name": "Diploid Alignment",

"name": "Diploid Alignment",

"type": "Subject",

"values": "",

"created_at": "02/10/2014",

"updated_at": "02/10/2014",

"created_by": [redacted],

"searchable_quick": false,

"searchable_advanced": null,

"searchable_batch": null,

"searchable": "",

"pii": "No",

"qtip": "",

"used_by": "Alignment Collaboration Files",

"used_by_display": "<a href='#' onClick='remoteJS({name:\"dialogs/AddSubjectTypeDlg\", obj_id:194 });'>Alignment Collaboration Files</a>"

},

{

"id": 2479,

"obj_id": 2479,

"display_name": "Diploid Alignments",

"name": "Diploid Alignments",

"type": "Subject Back Ref",

"values": "",

"created_at": "03/31/2014",

"updated_at": "01/23/2015",

"created_by": [redacted],

"searchable_quick": false,

"searchable_advanced": false,

"searchable_batch": false,

"searchable": "",

"pii": "No",

"qtip": "",

"used_by": "Patient, Information Technology Collaboration, Patient Follow Up Visit",

"used_by_display": "<a href='#' onClick='remoteJS({name:\"dialogs/AddSubjectTypeDlg\", obj_id:1 });'>Patient</a>, <a href='#' onClick='remoteJS({name:\"dialogs/AddSubjectTypeDlg\", obj_id:183 });'>Information Technology Collaboration</a>, <a href='#' onClick='remoteJS({name:\"dialogs/AddSubjectTypeDlg\", obj_id:264 });'>Patient Follow Up Visit</a>"

},

{

"id": 1863,

"obj_id": 1863,

"display_name": "Direction of File Transfer",

"name": "Direction of File Transfer",

"type": "Choice",

"values": "Sent, Received",

"created_at": "11/01/2013",

"updated_at": "11/01/2013",

"created_by": [redacted],

"searchable_quick": false,

"searchable_advanced": null,

"searchable_batch": null,

"searchable": "",

"pii": "No",

"qtip": "",

"used_by": "Information Technology Collaboration Files, Alignment Collaboration Files",

"used_by_display": "<a href='#' onClick='remoteJS({name:\"dialogs/AddSubjectTypeDlg\", obj_id:184 });'>Information Technology Collaboration Files</a>, <a href='#' onClick='remoteJS({name:\"dialogs/AddSubjectTypeDlg\", obj_id:194 });'>Alignment Collaboration Files</a>"

},

{

"id": 2311,

"obj_id": 2311,

"display_name": "Discussed in Screening Meeting",

"name": "Discussed in Screening Meeting",

"type": "Radio Buttons",

"values": "Yes, No",

"created_at": "02/28/2014",

"updated_at": "02/28/2014",

"created_by": [redacted],

"searchable_quick": false,

"searchable_advanced": null,

"searchable_batch": null,

"searchable": "",

"pii": "No",

"qtip": "",

"used_by": "Patient, Patient Follow Up Visit",

"used_by_display": "<a href='#' onClick='remoteJS({name:\"dialogs/AddSubjectTypeDlg\", obj_id:1 });'>Patient</a>, <a href='#' onClick='remoteJS({name:\"dialogs/AddSubjectTypeDlg\", obj_id:264 });'>Patient Follow Up Visit</a>"

},

{

"id": 1370,

"obj_id": 1370,

"display_name": "Discuss mode of transportatiion, lodging, persons traveling with family",

"name": "Discuss mode of transportatiion, lodging, persons traveling with family",

"type": "Radio Buttons",

"values": "Yes, No, Complete",

"created_at": "06/24/2013",

"updated_at": "09/19/2013",

"created_by": [redacted],

"searchable_quick": false,

"searchable_advanced": null,

"searchable_batch": null,

"searchable": "",

"pii": "No",

"qtip": "",

"used_by": "Patient Visit Tools, Patient Visit Planning for Version C",

"used_by_display": "<a href='#' onClick='remoteJS({name:\"dialogs/AddSubjectTypeDlg\", obj_id:163 });'>Patient Visit Tools</a>, <a href='#' onClick='remoteJS({name:\"dialogs/AddSubjectTypeDlg\", obj_id:192 });'>Patient Visit Planning for Version C</a>"

},

{

"id": 317,

"obj_id": 317,

"display_name": "Disposition Notes",

"name": "Disposition Notes",

"type": "Text Area",

"values": "",

"created_at": "04/08/2013",

"updated_at": "06/09/2014",

"created_by": [redacted],

"searchable_quick": false,

"searchable_advanced": null,

"searchable_batch": null,

"searchable": "",

"pii": "Yes",

"qtip": "",

"used_by": "Friday Meeting Notes, Sharepoint Legacy - Friday Meeting Notes",

"used_by_display": "<a href='#' onClick='remoteJS({name:\"dialogs/AddSubjectTypeDlg\", obj_id:82 });'>Friday Meeting Notes</a>, <a href='#' onClick='remoteJS({name:\"dialogs/AddSubjectTypeDlg\", obj_id:154 });'>Sharepoint Legacy - Friday Meeting Notes</a>"

},

{

"id": 553,

"obj_id": 553,

"display_name": "Disposition_Recommendation",

"name": "Disposition_Recommendation",

"type": "Text Area",

"values": "",

"created_at": "05/07/2013",

"updated_at": "06/10/2014",

"created_by": [redacted],

"searchable_quick": false,

"searchable_advanced": null,

"searchable_batch": null,

"searchable": "",

"pii": "Yes",

"qtip": "",

"used_by": "Labmatrix Legacy - Adult UDP Entry",

"used_by_display": "<a href='#' onClick='remoteJS({name:\"dialogs/AddSubjectTypeDlg\", obj_id:101 });'>Labmatrix Legacy - Adult UDP Entry</a>"

},

{

"id": 325,

"obj_id": 325,

"display_name": "Disposition Review Date",

"name": "Disposition Review Date",

"type": "Date",

"values": "",

"created_at": "04/08/2013",

"updated_at": "09/19/2013",

"created_by": [redacted],

"searchable_quick": false,

"searchable_advanced": null,

"searchable_batch": null,

"searchable": "",

"pii": "No",

"qtip": "",

"used_by": "Friday Meeting Notes, Sharepoint Legacy - Friday Meeting Notes",

"used_by_display": "<a href='#' onClick='remoteJS({name:\"dialogs/AddSubjectTypeDlg\", obj_id:82 });'>Friday Meeting Notes</a>, <a href='#' onClick='remoteJS({name:\"dialogs/AddSubjectTypeDlg\", obj_id:154 });'>Sharepoint Legacy - Friday Meeting Notes</a>"

},

{

"id": 366,

"obj_id": 366,

"display_name": "DNA Archive Samples",

"name": "DNA Archive Samples",

"type": "FreezerPro Barcode",

"values": "",

"created_at": "04/15/2013",

"updated_at": "09/19/2013",

"created_by": [redacted],

"searchable_quick": false,

"searchable_advanced": null,

"searchable_batch": null,

"searchable": "",

"pii": "No",

"qtip": "8 x 20ug samples on 96 well plate",

"used_by": "DNA Extraction",

"used_by_display": "<a href='#' onClick='remoteJS({name:\"dialogs/AddSubjectTypeDlg\", obj_id:122 });'>DNA Extraction</a>"

},

{

"id": 311,

"obj_id": 311,

"display_name": "DNA Barcode",

"name": "DNA Barcode",

"type": "FreezerPro Barcode",

"values": "",

"created_at": "04/05/2013",

"updated_at": "09/19/2013",

"created_by": [redacted],

"searchable_quick": false,

"searchable_advanced": null,

"searchable_batch": null,

"searchable": "",

"pii": "No",

"qtip": "",

"used_by": "",

"used_by_display": ""

},

{

"id": 467,

"obj_id": 467,

"display_name": "DNA Extractions",

"name": "DNA Extractions",

"type": "Subject Back Ref",

"values": "",

"created_at": "04/30/2013",

"updated_at": "10/29/2014",

"created_by": [redacted],

"searchable_quick": false,

"searchable_advanced": true,

"searchable_batch": false,

"searchable": "Advanced",

"pii": "No",

"qtip": "",

"used_by": "Patient, Research Sample, Patient Follow Up Visit",

"used_by_display": "<a href='#' onClick='remoteJS({name:\"dialogs/AddSubjectTypeDlg\", obj_id:1 });'>Patient</a>, <a href='#' onClick='remoteJS({name:\"dialogs/AddSubjectTypeDlg\", obj_id:86 });'>Research Sample</a>, <a href='#' onClick='remoteJS({name:\"dialogs/AddSubjectTypeDlg\", obj_id:264 });'>Patient Follow Up Visit</a>"

},

{

"id": 1334,

"obj_id": 1334,

"display_name": "DNA Extractions for FreezerPro",

"name": "DNA Extractions for FreezerPro",

"type": "Subjects",

"values": "",

"created_at": "06/19/2013",

"updated_at": "09/19/2013",

"created_by": [redacted],

"searchable_quick": false,

"searchable_advanced": null,

"searchable_batch": null,

"searchable": "",

"pii": "No",

"qtip": null,

"used_by": "LIMS Helper",

"used_by_display": "<a href='#' onClick='remoteJS({name:\"dialogs/AddSubjectTypeDlg\", obj_id:144 });'>LIMS Helper</a>"

},

{

"id": 554,

"obj_id": 554,
[truncated: 973,444 more chars]
